# Does physical activity-based intervention decrease repetitive negative thinking? A systematic review

**DOI:** 10.1371/journal.pone.0319806

**Published:** 2025-04-01

**Authors:** Shimeng Wang, Mingyang Lu, Xiaoxiao Dong, Yifan Xu

**Affiliations:** 1 Institute of Sports Science, Nantong University, Nantong, China; 2 Department of Physical Education, Dankook University, Yongyin, South Korea; 3 Nanjing Sport Institute, Nanjing, China; 4 Gdansk University of Physical Education and Sport, Gdansk, Poland; University of Zanjan, IRAN, ISLAMIC REPUBLIC OF

## Abstract

**Background:**

Repetitive negative thinking (RNT) is characterized by its persistence, difficulty in control, and the tendency to focus on negative thoughts and past events. It is recognized as a key factor in the development and maintenance of mental health issues such as depression and anxiety. A growing body of research suggests that physical activity-based interventions may effectively reduce RNT. However, the extent of this effect and the mechanisms behind it remain inconsistent across studies.

**Methods:**

This systematic review synthesized evidence from 19 peer-reviewed studies retrieved from PubMed, Web of Science, and other relevant databases up to December 2024. The objective was to investigate the efficacy of physical activity-based interventions in reducing RNT, with a particular focus on the influence of intervention type, duration, frequency, and intensity.

**Results:**

The review found that physical activity interventions effectively reduce RNT, particularly when combined with psychological training. Combined interventions yielded greater reductions than physical activity alone. Moderate-to-high intensity exercise (30-60 min/session, 3-5 times/week) over an extended period was most effective, likely due to physiological, psychological, and social mechanisms. Single-session interventions showed limited effects, emphasizing the need for sustained engagement. Notably, interventions were more effective in individuals with depression, anxiety, or chronic stress, whereas effects in healthy individuals were smaller and more variable, suggesting that baseline symptomatology enhances intervention benefits.

**Conclusions:**

This review underscores the importance of designing intervention protocols that integrate both physical and psychological components to achieve greater reductions in RNT. The findings provide empirical support for the use of combined interventions involving physical activity and psychological training as an effective strategy for managing RNT. Additionally, future research should prioritize identifying optimal intervention characteristics (e.g., type, frequency, intensity) and addressing methodological limitations, such as the inclusion of diverse participant samples and broader language coverage, to provide more comprehensive insights into effective intervention strategies. These findings have important implications for mental health interventions and offer practical guidance for developing evidence-based approaches to reduce RNT.

## Introduction

Depression and anxiety are common mental disorders characterized by high prevalence, high rates of recurrence, increased risk of suicide, and significant disability. These conditions pose a major public health challenge worldwide[1]. According to the latest data from the Global Burden of Disease (https://vizhub.healthdata.org/gbd-results/), approximately 280 million people worldwide suffer from depression, while 301 million are affected by anxiety. Central to these mental health issues is repetitive negative thinking (RNT), a cognitive pattern involving repetitive, ineffective, and uncontrollable contemplation of negative events[2–3]. RNT includes two primary forms: rumination and worry[4]. Rumination involves persistent reflection and overanalysis of past negative experiences[5], which is closely associated with depression[6]. Conversely, worry is characterized by excessive concern about potential future negative events[7] and is strongly linked to anxiety disorders[8]. Given that RNT predicts the onset and maintenance of depression and anxiety[9], interventions targeting RNT may offer significant benefits for mental health outcomes.

The etiology of RNT is multifaceted, with both biological and environmental factors playing influential roles[10]. For instance, biological factors like genetic predispositions and hormonal imbalances have been implicated in RNT development, while environmental factors such as chronic stress and adverse childhood experiences contribute significantly to its persistence. These diverse influences highlight the need for holistic approaches to intervention[10]. Current treatments for RNT include pharmacological and psychological interventions. Pharmacological approaches, such as the use of selective serotonin reuptake inhibitors (SSRIs) and mood stabilizers, have demonstrated effectiveness in reducing RNT by addressing physiological mechanisms[11]. However, long-term use of pharmacological treatments can result in adverse effects such as weight gain, sedation, and cognitive impairment[12–13]. On the other hand, psychological interventions like Metacognitive Therapy and Mindfulness-Based Therapy emphasize cognitive and emotional regulation, with promising results[14]. Nevertheless, these interventions often lack a direct physiological impact, highlighting the need for more comprehensive, side-effect-free approaches.

Emerging evidence supports the potential of physical activity-based interventions to reduce RNT. Physical activity, unlike pharmacological and psychological treatments, offers a unique triadic effect on physiological, psychological, and social dimensions. Studies suggest that physical activity enhances cognitive control, reduces emotional reactivity, and mitigates the impact of rumination and worry[15–16]. Moreover, physical activity-based interventions have been shown to reduce sensitivity to anxiety[17–18], thereby impacting RNT pathways. These mechanisms include enhanced neuroplasticity, endorphin release, and improved cognitive control, which facilitate the regulation of negative thought patterns[19–20].

Research indicates that physical activity-based interventions positively affect a range of psychological disorders, including worry, rumination, and memory bias. However, their influence on other critical psychological conditions like anxiety, PTSD, and persecutory delusions remains underexplored. Given that these conditions share RNT as a transdiagnostic feature, the potential for physical activity to target this common mechanism across multiple disorders warrants further investigation. Furthermore, while the positive impact of physical activity on various psychological outcomes is well-documented, the mechanisms through which these effects occur remain unclear. It is uncertain whether the observed reductions in worry, rumination, and other RNT-related symptoms can be solely attributed to reductions in RNT or if additional mechanisms are at play.

Despite the existing literature, gaps remain in the understanding of how physical activity influences RNT. The rationale for linking diverse psychological disorders with distinct symptom profiles and risk factors to a singular variable—RNT—requires further clarification. While cognitive control deficits are a known precursor to RNT[15], it is essential to understand how physical activity uniquely impacts these deficits compared to other interventions. Moreover, the current body of research has not sufficiently justified the study’s novelty or the significance of investigating the direct effects of physical activity on RNT.

Physical activity interventions offer multiple benefits, including low cost, ease of implementation, and minimal side effects. Nonetheless, inconsistencies in findings highlight the need for further research. Some studies, for example, report no significant change in worry levels following physical activity interventions[21], while others show significant reductions in worry and rumination[20,22]. These mixed outcomes could be due to variations in participant characteristics, intervention design, and measurement tools. To address these gaps, this study aims to conduct a systematic review of the impact of physical activity-based interventions on RNT. By synthesizing current research, the study seeks to identify unresolved issues, clarify the conditions under which physical activity is most effective, and provide guidance for future research and practical applications. The novelty of this study lies in its systematic evaluation of heterogeneous findings, with a focus on elucidating the specific conditions that yield optimal intervention outcomes.

## Materials & methods

This systematic review adhered to the PRISMA 2020 statement: an updated guideline for reporting systematic reviews (https://www.prisma-statement.org/). The protocol for this systematic review was registered on INPLASY (202440095) and is accessible in its entirety on inplasy.com (https://doi.org/10.37766/inplasy2024.4.0095).

### Search strategy

A comprehensive search was conducted across PubMed, Web of Science, Cochrane Library, and APA PsycNET databases. The search covered studies published from database inception to December 31, 2024. The inclusion criteria for selecting studies were as follows: (1) studies that utilized physical activity as the primary intervention; (2) studies that measured RNT-related outcomes such as rumination, worry, or perseverative thinking; (3) peer-reviewed, full-text articles published in English; and (4) studies with clear pre-test and post-test measures. Exclusion criteria included review articles, conference abstracts, case reports, and studies without accessible full texts or usable data.

To ensure transparency, the search strategy included the following key terms: “physical activity,” “exercise,” “aerobic exercise,” “resistance training,” “acute exercise,” “exercise training,” “repetitive negative thinking,” “rumination,” “worry,” and “perseverative thinking.” This strategy was applied across all databases to maximize coverage. It is worth noting that “resistance training” was included as a search term, addressing a prior discrepancy where it was listed in Table 1 but not the search strategy.

**Table 1:** Characteristics of selected studies.

First Author (year)	Country	Participants	Design	Sample Size	Characteristics of intervention					Outcome	Scale	Main result
					Program	Min	Times (week)	Intensity	Duration			
Aalsm et al., 2022	USA	Adolescents with Depression Age: 15.2 ± 1.3 Gender: 9male, 34female	SAT	43	mind-Body Skills	90	1	Moderate intensity	10	Worry	PSWQ	Mind-Body Skills intervention reduced worry and depressive symptoms.
Alderman et al., 2016	USA	clinical depression and Health adults Age: 21.0 ± 3.2 Gender: 15male,37female	mixed design‌‌	52	mental training and physical training	80	2	Moderate intensity	8	Rumination	RRS	MAP training decreased rumination and improved cognitive control.
Basso et al., 2022	USA	moderately fit healthy adults Age: 25-59 82.9%female) 17.1%male)	RCT	80	cycling	45	7	NR	12	Rumination	RRS	Exercise intervention showed no direct effect on worry reduction.
Craft, 2005	USA	clinical depression Age: 43.21 ± 13.23 Female	QE	19	physical activity	30	3	Moderate intensity	9	Rumination	RRS	Exercise reduced depressive symptoms but had limited impact on worry.
de Bruin, 2016	Netherland	Stressed Young Adults Age: 18-40 Female 55, Male 20	RCT	75	physical exercise	20	7	NR	5	Worry	PSWQ	All groups exhibited reductions in worry levels, with the physical exercise group showing the strongest intervention effect.
Gordon et al., 2020	Ireland	healthy adults Age: 28.4 ± 6.6 5 males, 9 females	RCT	28	Resistance exercise training	NR	2	NR	8	Worry	PSWQ	Worry levels decreased in the experimental group and increased in the control group.
Gordon et al., 2021	Ireland	analogue generalized anxiety disorder Age: E(26.5 ± 5.8) C(26.7 ± 4.9) 10males 17females	RCT	27	Resistance exercise training	NR	2	NR	8	Worry	PSWQ	The experimental group saw a significant drop in worry levels, in contrast to the control group, which showed an increase.
Herring et al., 2011	Ireland	generalized anxiety disorder Age:20.8 ± 1.4 20females	RCT	30	Resistance exercise training	NR	2	Moderate intensity	6	Worry	PSWQ	The experimental group experienced a significant reduction in worry levels, while the control group remained unchanged.
Herring et al., 2017	Ireland	generalized anxiety disorder Age:20.8 ± 1.4 17females	Pilot study	17	Acute exercise	30	1	High intensity	6	Worry	PSWQ	Worry levels significantly decreased in the experimental group, with no change in the control group.
Herring et al., 2019	Ireland	analogue generalized anxiety disorder Age:21.4 ± 2.3 19males 16females	RCT	35	Acute exercise	30	1	High intensity	6	Worry	PSWQ	A significant reduction in worry levels was observed in the experimental group, with the control group showing no change.
Jacoby et al., 2024	USA	Adults with Generalized Anxiety Disorder Age: 33.37 70%female,30%male	RCT	226	Kundalini yoga	60	12	NR	1	Worry	PSWQ	CBT and yoga both improved sleep and reduced worry, but no significant difference between groups.
La Rocque et al., 2021	Canada	Depression in Women. Age:E(34.85 ± 15.15) C(29.40 ± 13.08)	RCT	35	exercise	60	2	NR	8	Rumination	RRS	The experimental group reported a significant reduction in rumination, whereas the control group experienced a slight decrease.
McDowell et al., 2016	Ireland	Health young adult Age:21.2 ± 1.5	Repeated Measures Design	27	Acute exercise	30	1	High intensity	1	Worry	PSWQ	In the experimental group, worry levels decreased in males but increased in females; the opposite trend was observed in the control group.
Plag et al., 2020	Germany	Young adults with Generalized Anxiety Disorder Age: 40.18 ± 12.4 24female, 9male	RCT	33	HIIT	12 sessions over 12 day		High intensity	12 day	Worry	PSWQ	HIIT training effectively reduced worry and anxiety symptoms.
Schuver et al., 2016	USA	Depression in Women Age:42.68 ± 4.95	RCT	34	yoga	60	0.5/1	NR	12	Rumination	RRS	The experimental group demonstrated a significant decrease in rumination, while the control group showed a slight decrease.
Shors et al., 2018	USA	Adult women Age:18-32	QE	105	Mental training and physical training	60	2	Moderate intensity	6	Rumination	RRS	The experimental group showed a significant decrease in rumination, with rumination levels slightly increasing in the control group.
Shrimal et al., 2024	India	Healthcare professionals Age: E(34 ± 10) C(32 ± 7) 53female, 38male	QE	110	Yoga	30-minute self-practice three times a week/ a weekly one-hour Yoga		Low intensity	4	Perseverative Thinking	PTQ	Yoga intervention reduced stress-related perseverative thinking among healthcare professionals.
van Aalst et al., 2021	Belgium	Healthy female individuals Age:E(31.8 ± 9.8) C(24.9 ± 5.1)	QE	30	Yoga	60	2	NR	12	Worry	PSWQ	The experimental group experienced a decrease in worry levels, while the control group showed a significant increase.
Vollbehr et al., 2021	Netherlands	Adults with chronic mood disorders Age: 49 ± 13.81	RCT	11		60	1	Moderate intensity	9	Worry and Rumination	RRS and PSWQ	Mindful yoga intervention showed potential in reducing worry and rumination.

SAT: single-arm clinical trial; RCT:Randomized Controlled Trial; QE: Quasi-experimental; NR: Not reports; RRS:Ruminative Response Scale; PSWQ:Penn State Worry Questionnaire;PTQ: Perseverative Thinking Questionnaire.

### Eligibility criteria

#### Types of studies.

The study selects English-language literature published in peer-reviewed journals. The research type must be either a randomized controlled trial (RCT) or a non-randomized controlled trial that reports sufficient statistical details (such as means, standard deviations, sample sizes, etc.). Literature reviews, case reports, conference abstracts, study protocols, duplicate publications, studies with inaccessible full texts or unavailable data extraction, as well as studies without access to original data, will be excluded.

#### Types of interventions.

Among the selected studies, the experimental group in controlled trials received interventions based solely on physical activity (physical activity programs are not limited), while the control group received standard interventions or no intervention.

#### Types of outcomes measures.

The research findings must be assessed using validated instruments and report indices measuring RNT (rumination, worry, perseverative thinking) outcomes.

### Study selection and data extraction

To minimize the possibility of omission or misjudgment, two researchers independently conducted literature screening, followed by cross-validation. In case of discrepancies during the screening process, they were resolved through discussion; if consensus could not be reached, a third party made the final adjudication. Due to the large number of literature items to be screened, multiple rounds of screening will be conducted, supplemented by relevant systematic reviews or meta-analyses to avoid literature omissions.

The included studies are managed using EndNote 21 software (https://endnote.com/product-details/). This software is used to store, organize, and eliminate duplicate records, ensuring efficiency and accuracy in study selection and data extraction. In addition to its role in duplicate removal, EndNote 21 is used to organize references, categorize studies by relevance, and track changes made during each round of screening. After removing duplicate studies, titles and abstracts are reviewed, and preliminary screening is conducted based on inclusion and exclusion criteria. Subsequently, relevant full-text articles are obtained and thoroughly reviewed. The remaining studies from the initial screening undergo a second round of screening to identify experimental studies that can be included in this research. The software facilitates multiple rounds of screening and assists in organizing references for systematic reviews or meta-analyses, thereby ensuring that no relevant literature is overlooked.

The research will utilize standardized forms for data recording. These forms ensure consistency in data extraction and documentation, a key addition that was not explicitly stated in the original version. The extracted data will encompass various aspects of the included studies. Firstly, it will capture the fundamental details of each study, such as the title, authors, and publication date. Secondly, it will compile basic information about the study participants, including their average age, gender distribution, and the sample sizes of both the experimental and control groups. Additionally, it will document the intervention measures undertaken, specifying the interventions’ frequency, timing, and duration. Moreover, it will detail the control measures employed, including their frequency, timing, and characteristics of the control group. These details are recorded in a standardized data extraction form, which promotes uniformity and transparency in data collection.

Furthermore, the data extraction process will include information about the content and measurement tools of the indicators used to assess RNT, focusing on the type of tool, its validity, reliability, and application context within each study. The extracted information will include the type of RNT assessment tool, its validity, reliability, and application context within each study. Finally, the study design will be meticulously documented, with a focus on key elements evaluating bias risk, such as the type of study design (randomized controlled trial or quasi-experimental design), blinding, allocation concealment, and risk of bias assessment criteria. Information on how each of these elements was addressed in the included studies will be documented in the standardized data extraction forms, providing a detailed account of how bias-related elements are tracked and recorded. This methodological enhancement ensures greater transparency and traceability in the assessment of study quality. Similarly, two researchers will independently extract data and cross-check the extracted data. In case of discrepancies, a third party will be involved to resolve them. This systematic and transparent approach reduces the risk of errors and omissions, ensuring a comprehensive and reproducible data extraction process.

### Risk of bias assessment

The methodological quality of the included studies was assessed using the Physiotherapy Evidence Database (PEDro) scale, which consists of 11 evaluation criteria. Each criterion is scored 1 point for compliance with the standard, while 0 points are assigned for criteria that are not applicable or not met. Studies with a total score ranging from 0 to 3 points are classified as low-quality, 4 to 7 points as medium-quality, and 8 to 11 points as high-quality. This section on ‘Risk of Bias Assessment’ represents a new addition to the methodology, introducing a clear and standardized approach for assessing study quality and risk of bias. The evaluation was independently conducted by two researchers, with any disagreements resolved through discussion or adjudicated by a third party. This process ensures objectivity and reliability in quality assessment and provides a clear basis for determining the quality of the evidence included in this research.

## Results

### Selection process and results

**Fig 1:**
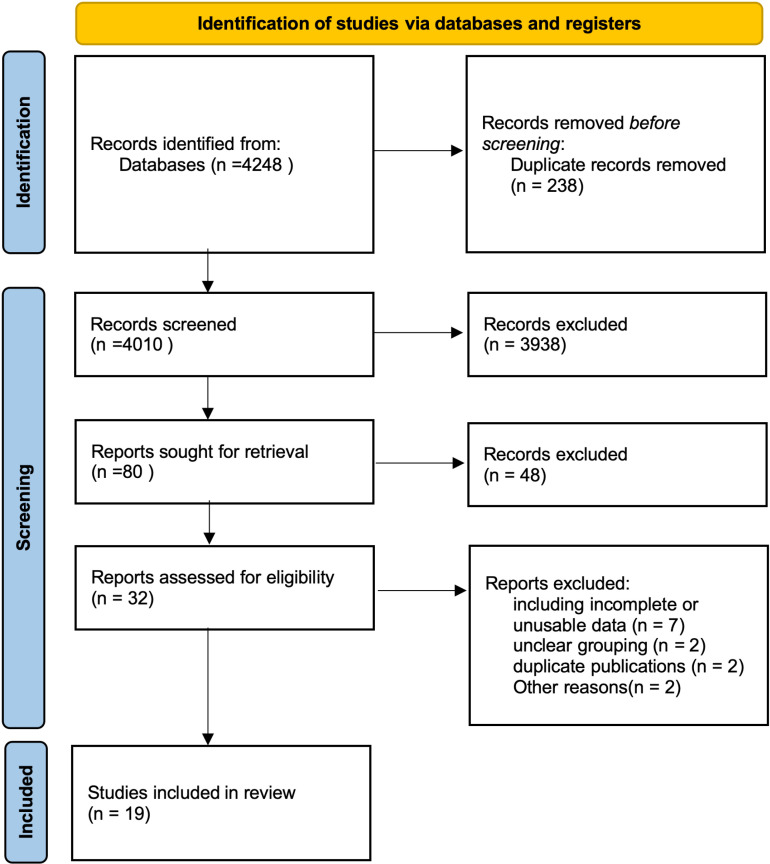
PRISMA(2020) flowchart of record identification, screening, and inclusion.

The study selection process is illustrated in Fig 1. A total of 4,248 studies were identified from electronic databases. After removing 238 duplicate records, 4,010 studies remained for title and abstract screening. During this phase, 3,938 records were excluded due to irrelevance, leaving 80 studies for full-text retrieval. Among these, 48 reports were excluded for not meeting the research objectives, resulting in 32 studies being assessed for eligibility. After further evaluation, 13 studies were excluded for various reasons, including incomplete or unusable data (n =  7), unclear grouping (n =  2), duplicate publications (n =  2), and other reasons (n =  2). Ultimately, 19 studies met the inclusion criteria and were included in the systematic review. The selection criteria required studies to have a randomized controlled trial (RCT), quasi-experimental, or pre-post design, etc. with physical activity as the intervention and pre-post outcome measurements related to RNT.

### Study characteristics

Table 1 provides an overview of the 19 included studies, summarizing details such as the first author, publication country, participant characteristics, study design, sample size, intervention protocol, outcome description, outcome measures, and measurement tools. The studies were conducted between 2005 and 2024 across the United States, the Netherlands, Ireland, Canada, India, Germany, and Belgium. The study designs included 11 randomized controlled trials (RCTs)(Basso et al., 2022[23]; de Bruin, 2016[24]; Gordon et al., 2020[25]; Gordon et al., 2021[26]; Herring et al., 2011[27]; Herring et al., 2017[28]; Herring et al., 2019[29]; Jacoby et al., 2024[30]; La Rocque et al., 2021[31]; Plag et al., 2020[32]; Schuver et al., 2016[33]; Vollbehr et al., 2021[34]), 4 quasi-experimental studies(Craft, 2005[35]; Shors et al., 2018[36]; Shrimal et al., 2024[37]; van Aalst et al., 2021[21]), 1 pilot study(Herring et al., 2017[28]), 1 repeated measures study(McDowell et al., 2016[38]), 1 mixed design study(Alderman et al., 2016[39]), and 1 single-arm clinical trial(Aalsm et al., 2022[40]). The total sample size was 1,084 participants, comprising a diverse population of healthy individuals, individuals with depression, generalized anxiety disorder (GAD), stress-related disorders, and healthcare professionals. The participants’ ages ranged from 13 to 65 years, with some studies reporting gender distributions, while others did not specify participant demographics. The interventions investigated in these studies included mind-body interventions (e.g., mindfulness, yoga, and MAP training), resistance training, aerobic exercise, high-intensity interval training (HIIT), and combined approaches integrating both physical activity and psychological training. The intensity of these interventions ranged from low to high, with session durations varying from 30 to 90 minutes per session and frequencies ranging from 1 to 7 sessions per week. The primary outcomes measured across the studies focused on worry and rumination, with one study (Shrimal et al., 2024[37]) specifically assessing perseverative thinking. Validated assessment tools used included the Ruminative Response Scale (RRS), Penn State Worry Questionnaire (PSWQ), and Perseverative Thinking Questionnaire (PTQ).

### Risk of bias assessment

**Fig 2:**
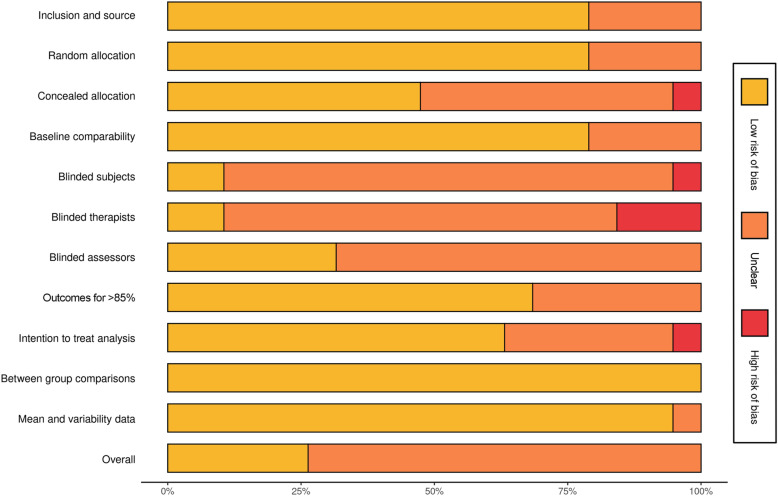
Histogram of Risk of Bias Assessment results of the included studies.

**Fig 3:**
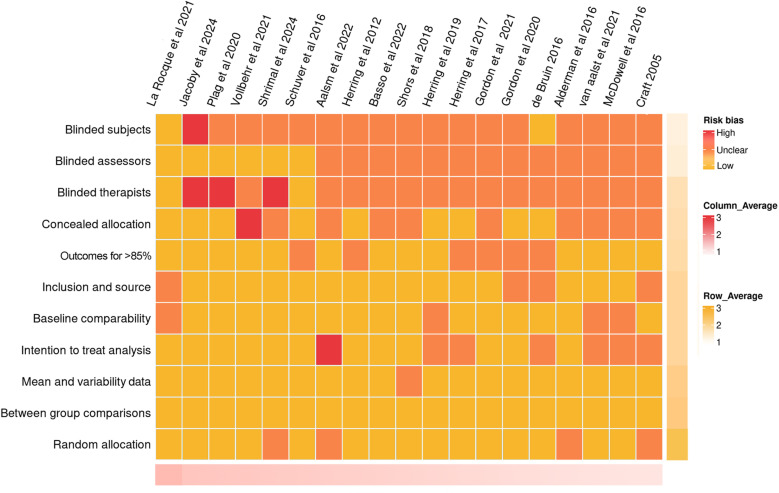
Heatmap of Risk of Bias Assessment results for included studies.

The methodological quality of the 19 included studies was evaluated using the Physiotherapy Evidence Database (PEDro) scale. The overall quality was categorized as moderate to high. Specifically, 14 studies were classified as moderate quality (Aalsm et al., 2022[40]; Alderman et al., 2016[39]; Basso et al., 2022[23]; Craft, 2005[35]; de Bruin, 2016[24]; Gordon et al., 2020[25]; Gordon et al., 2021[26]; Herring et al., 2011[27]; Herring et al., 2017[28]; Herring et al., 2019[29]; McDowell et al., 2016[38]; Shors et al., 2018[36]; Shrimal et al., 2024[37]; van Aalst et al., 2021[21]), and 5 studies were classified as high quality (Jacoby et al., 2024[30]; La Rocque et al., 2021[31]; Plag et al., 2020[32]; Schuyer et al., 2016[33]; Vollbehr et al., 2021[34]). The lowest scoring items on the PEDro scale were blinding of subjects, blinding of therapists, and blinding of assessors. The absence of blinding for these roles could introduce performance and detection bias, which may influence the observed intervention effects on RNT. The overall quality assessment results are presented in Fig 2, while Fig 3 presents the quality assessment results for each included study. Fig 2 illustrates the distribution of PEDro scores for each included study, highlighting the proportion of high- and moderate-quality studies. Fig 3 provides a visual heatmap of the quality assessment for each included study, detailing the compliance of each study with 11 PEDro criteria.

### Measurement tools and participant characteristics

The primary tools for assessing repetitive negative thinking (RNT) in the included studies were the Ruminative Responses Scale (RRS), the Penn State Worry Questionnaire (PSWQ), and the Perseverative Thinking Questionnaire (PTQ). The RRS focuses on measuring the tendency to engage in repetitive, self-focused negative thoughts, typically linked to depressive symptoms. The PSWQ assesses the intensity and frequency of worry, a cognitive process often associated with anxiety disorders. The PTQ was specifically used to measure perseverative thinking in one study (Shrimal et al., 2024[37]). These tools were applied to assess RNT across healthy individuals, individuals with depression, anxiety, and stress-related disorders. Participant characteristics varied, with 6 studies focusing on individuals with depression (Aalsma et al., 2022[40]; Alderman et al., 2016[39]; Craft, 2005[35]; La Rocque et al., 2021[31]; Schuver et al., 2016[33]; Vollbehr et al., 2021[34]), 6 studies focusing on individuals with anxiety (Gordon et al., 2021[26]; Herring et al., 2011[27]; Herring et al., 2017[28]; Herring et al., 2019[29]; Jacoby et al., 2024[30]; Plag et al., 2020[32]), 5 studies targeting healthy individuals (Basso et al., 2022[23]; Gordon et al., 2020[25]; McDowell et al., 2016[38]; Shors et al., 2018[36]; van Aalst et al., 2021[21]), and 2 studies targeting individuals under stress (de Bruin, 2016[24]; Shrimal et al., 2024[37]).

### Overcomes

Of the included studies, 6 focused on physical activity’s impact on rumination (Alderman et al., 2016[39]; Basso et al., 2022[23]; Craft, 2005[35]; La Rocque et al., 2021[31]; Schuver et al., 2016[33]; Shors et al., 2018[36]), with 5 studies reporting positive effects on reducing rumination. Only 1 study (Shors et al., 2018[36]) found that physical activity alone was insufficient, but combining it with psychological training yielded positive results. For worry, 8 studies (de Bruin, 2016[24]; Gordon et al., 2020[25]; Gordon et al., 2021[26]; Herring et al., 2011[27]; Herring et al., 2017[28]; Herring et al., 2019[29]; McDowell et al., 2016[38]; van Aalst et al., 2021[21]) examined its outcomes. Of these, 7 studies found positive results, while only 1 (van Aalst et al., 2021[21]) found no significant change following a yoga intervention. Additionally, 3 studies (Aalsma et al., 2022[40]; Jacoby et al., 2024[30]; Plag et al., 2020[32]) investigated interventions targeting both worry and rumination in clinical populations, with results indicating moderate to strong effects in reducing repetitive negative thinking. Finally, 2 studies examined broader aspects of repetitive negative thinking (RNT). Shrimal et al., 2024 focused on stress-related perseverative thinking, using the Perseverative Thinking Questionnaire (PTQ) to assess the impact of a yoga-based intervention on repetitive thought patterns, reporting a significant reduction in stress-related negative thinking[37]. Vollbehr et al., 2021 on the other hand, evaluated worry and rumination in adults with chronic mood disorders, finding that mindfulness interventions showed potential in reducing both cognitive patterns[34],.

### Key findings

#### Impact of physical activity-based on RNT.

Physical activity-based interventions led to significant reductions in repetitive negative thinking (RNT), particularly in rumination and worry. The most pronounced effects were observed in multimodal interventions combining aerobic and resistance training, suggesting a synergistic effect between different forms of physical activity. Additionally, mindfulness-based interventions, such as yoga and meditation, demonstrated effectiveness, particularly in reducing stress-related perseverative thinking. These findings suggest that both structured exercise and mindfulness-based movement practices can be beneficial in mitigating RNT.

#### Effectiveness of different intervention types.

The effectiveness of physical activity interventions varied based on intervention characteristics and participant profiles. Higher intensity and longer-duration interventions generally resulted in greater reductions in RNT-related measures, supporting a dose-response relationship. Notably, studies conducted in clinical populations (e.g., individuals with depression, anxiety, or chronic stress) reported larger effect sizes, whereas interventions in healthy individuals yielded smaller or more variable effects. This suggests that physical activity may exert a stronger impact on RNT when baseline symptomatology is present, underscoring the potential for targeted interventions in at-risk populations.

### Summary

These findings provide new insights into the role of physical activity-based in mitigating RNT, particularly rumination and worry. Among various interventions, combined aerobic and resistance training yielded the most substantial reductions, reinforcing the synergistic effects of multimodal approaches. Furthermore, the analysis supports a dose-response relationship, with higher intensity and longer-duration interventions leading to greater improvements in RNT-related measures. Importantly, the differential effects observed between clinical and healthy populations suggest that physical activity -based may be particularly effective as an intervention for individuals experiencing heightened baseline levels of RNT. These findings highlight the potential of physical activity as a non-pharmacological intervention and emphasize the importance of tailored intervention designs to maximize therapeutic benefits across diverse populations.

## Discussion

The present study aims to comprehensively analyze whether physical activity-based interventions can reduce RNT. Therefore, searches were conducted in databases such as PubMed, Web of Science, etc., from inception to December 2024. Through screening, a total of 19 studies relevant to the research topic were included for systematic review. Based on the findings of included the studies, the following discussion is presented:

Firstly, physical activity-based interventions consistently demonstrate a positive trend and conclusion in reducing RNT, which aligns with previous research findings[39]. However, studies involving single-session physical activity interventions showed no significant reduction in worry and rumination. This is consistent with findings indicating that short-term interventions may not induce sufficient physiological and psychological changes to significantly affect RNT. One possible reason is that single-session activities do not provide sufficient stimulation for long-term neuroplasticity, which requires repetitive training to induce stable changes in brain structure[41]. Additionally, single-session interventions may trigger cognitive biases, such as negative memory biases, especially in individuals with high levels of rumination[42]. As a result, short-term interventions may exacerbate certain negative thinking patterns rather than reduce them. Therefore, sustained and repeated interventions are recommended to achieve lasting effects on RNT. Instead, single-session interventions may exacerbate certain cognitive biases, such as negative memory biases, particularly in individuals with high levels of rumination[42]. RNT is the result of multiple factors, mechanisms, and targets interacting with each other, making its underlying mechanisms quite complex[4]. Although identifying the mechanisms is beyond the scope of this study, several potential physiological, psychological, and social mechanisms can explain why there are positive outcomes in RNT following participation in physical activity-based interventions. Firstly, there are physiological mechanisms at play. Physical activity is believed to influence the brain’s physiological processes, including neurotransmitter release, neurogenesis, and enhancement of brain function[43–44]. For instance, physical activity promotes the release of endorphins, which are endogenous chemicals associated with pleasure and relaxation, thus alleviating anxiety and depressive symptoms and reducing the tendency for RNT [45–46]. Endorphins bind to opioid receptors in the brain, which are linked to feelings of pleasure and relaxation[47]. This process helps reduce cognitive processes such as worry and rumination by alleviating negative emotional arousal, which has been associated with a decrease in RNT. The release of endorphins triggers activation of the opioid receptors in the brain, which are linked to feelings of pleasure and relaxation[47]. This mechanism may contribute to a reduction in cognitive processes such as worry and rumination, as the reduction of negative emotional arousal has been associated with lower RNT. Therefore, the role of endorphin release serves as a crucial physiological mechanism in the relationship between physical activity and RNT. These improvements at the physiological level are closely linked to mental health. Additionally, research suggests that individuals who engage in regular physical activity may be less susceptible to the impact of rumination on the hypothalamic-pituitary-adrenal axis reactivity, thereby mitigating the effects of rumination on the cortisol output pathway[48]. Therefore, attaining these physiological effects may help reduce negative emotions in individuals, thereby inhibiting the onset of RNT.

Secondly, there are psychological mechanisms at play. Physical activity can promote the release of endogenous chemicals such as endorphins, leading to feelings of pleasure and relaxation, alleviating anxiety and depressive symptoms, and reducing the tendency for RNT[45–46]. Moreover, research evidence suggests a close association between cognitive control deficits and RNT[15]. Physical activity, on the other hand, contributes to enhancing cognitive control abilities, improving cognitive reappraisal[49], and regulating self-monitoring and thought regulation abilities. This helps individuals avoid getting trapped in meaningless thought loops, thereby reducing RNT[10]. Furthermore, physical activity can promote a sense of agency and self-efficacy, which are critical for mitigating repetitive and negative thought patterns. These psychological benefits may vary depending on participants’ adherence to physical activity protocols. Research indicates that voluntary engagement in physical activity may yield greater psychological benefits compared to participation as research subjects, as it reflects intrinsic motivation and long-term commitment[50]. Voluntary engagement fosters a sense of autonomy, competence, and relatedness, which are key components of intrinsic motivation[51]. This suggests that interventions encouraging voluntary participation may result in stronger and more sustainable reductions in RNT. Intrinsic motivation, driven by the satisfaction of one’s psychological needs for autonomy, competence, and relatedness, is a key determinant of sustained engagement in physical activity[51]. By fostering a sense of voluntary engagement, physical activity programs may promote higher levels of adherence, thereby enhancing the effectiveness of interventions targeting RNT.

Thirdly, there are social effects mechanisms at play. Physical activity is often a social behavior, such as participating in team sports, fitness classes, and outdoor activities, which can promote communication and interaction with others, fostering positive social relationships[52]. Positive interactions and social engagement in the social environment can increase individuals’ self-esteem, self-confidence, and sense of belonging, thereby reducing the occurrence of RNT[53]. Additionally, the social support system also plays a crucial role in the impact of physical activity on mental health. Research evidence suggests that individuals with strong social support networks are more likely to actively engage in physical activity and have better coping abilities when facing stress and difficulties[54]. This form of social support can originate from various sources such as family, friends, colleagues, and the community. It provides individuals with emotional and informational support as well as tangible assistance, which can mitigate the risk of mental health issues, including the propensity for RNT. Furthermore, when examining the social factors influencing the relationship between physical activity and RNT, the integrated role emphasized in the Biopsychosocial Model must not be overlooked. This model posits that physiological, psychological, and social factors interact to collectively shape an individual’s overall health status[55]. Therefore, considering the combined influence of physiological, psychological, and social factors can provide a more comprehensive understanding of the impact of physical activity on RNT and offer a stronger basis for developing targeted interventions. Furthermore, compared to single physical activity interventions, combined interventions that include meditation, mindfulness, and other psychological interventions have shown significant positive effects in reducing RNT. These research findings support previous studies[56]. The study suggests that the positive effects of combined interventions can be discussed from multiple perspectives. Firstly, combined interventions simultaneously regulate both the emotional and cognitive aspects of individuals. Physical activity reduces depressive and anxious emotions [57], while psychological training helps individuals establish healthier psychological mechanisms [3,58]. Therefore, combined interventions can effectively intervene in RNT by addressing both emotional and cognitive regulation simultaneously. Secondly, combined interventions provide additional support and resources, enriching the avenues through which individuals benefit and enhancing the effectiveness of interventions. Research evidence indicates that following combined meditation and aerobic exercise interventions, there was a significant reduction in rumination in the experimental group, while the control group, which engaged solely in physical activity, showed no significant change[36]. This suggests that combined interventions, which target both emotional regulation and cognitive processes, are more effective than single interventions in reducing RNT[36]. Such results may be attributed to the fact that in combined interventions involving physical activity and psychological training, individuals not only benefit from the exercise but also gain additional psychological support, emotional guidance, and cognitive-behavioral skill practice through the psychological training component. These factors contribute to individuals’ better understanding and management of their emotions and thoughts, thereby enhancing control over the allocation of limited brain resources[59]. This enables them to address the negative issues associated with RNT more effectively. Thirdly, combined intervention strategies can enhance overall physical and mental health by improving physical health levels[60]. By integrating physical activity with psychological training, individuals may achieve a better balance at both physiological and psychological levels. Such a holistic mind-body intervention approach can assist individuals in developing healthy lifestyle habits and establishing robust psychological mechanisms, thereby better equipping them to counter the effects of RNT. Finally, it should be noted that this combined intervention approach falls under the category of mind-body exercises, which integrate physical activity with psychological training. Therefore, it is recommended to consider mind-body exercise programs such as tai chi, health qigong, yoga, etc., when addressing RNT. These mind-body exercise programs not only enhance physical health but also improve cognitive control and emotional regulation abilities[61,62], thereby improving mental well-being and providing effective strategies for addressing RNT issues.

Finally, interventions characterized by moderate duration, moderate frequency, moderate intensity, and longer duration demonstrate a predominance of positive effects on RNT in the studies included. The study suggests that the continuity and frequency of interventions, timing, as well as the adaptability of both physical and psychological aspects are important factors in explaining these research results. Firstly, continuity and frequency play crucial roles. The sustained and moderate frequency of exercise interventions significantly influences their effectiveness. Moderate-frequency exercise interventions ensure sufficient continuity, allowing participants to continuously benefit over a period of time. Additionally, intervals between sessions help the body adapt to the exercise load, preventing fatigue and overtraining. Additionally, studies have found that individuals with high levels of rumination may experience more negative memory biases during acute exercise, exacerbating rumination[42]. These findings support the notion that the short-term impact of exercise on rumination may be relatively limited, indicating that short-term exercise interventions may not be sufficient to trigger lasting physiological and psychological changes. Instead, they may have a noticeable adverse effect on RNT. Secondly, the time window is crucial. Research indicates that both psychological and physiological changes require time to stabilize and consolidate[63]. Long-term exercise interventions allow individuals to gradually adapt to exercise and derive benefits from it. Moreover, prolonged exercise interventions may require more time to induce changes in neuroplasticity[41], thereby influencing cognitive and emotional patterns. Thirdly, physical and psychological adaptability. Sustained moderate-intensity exercise may provide the body with sufficient opportunities to adapt to the exercise load, promoting cardiovascular health, enhancing muscle endurance, and more. This physical adaptation may have a positive impact on emotional and cognitive states, reducing the tendency to engage in RNT. Thirdly, physical and psychological adaptability. Sustained moderate to moderate-intensity exercise provides ample opportunities for the body to adapt to the exercise load. This adaptation optimizes the effects of exercise and consequently exerts positive influences on emotional and cognitive states, reducing the tendency for RNT. Hillman et al[64] proposed that at least 30 minutes of moderate-intensity physical activity is necessary to induce cellular changes in the brain, which subsequently affect cognition. This process has been linked to changes in neuroplasticity, particularly in the prefrontal cortex, which is responsible for cognitive control and executive function. The findings in this study align with this notion, as interventions lasting 30-60 minutes demonstrated improvements in cognitive reappraisal and reductions in RNT. The intervention effects of programs lasting 25 to 60 minutes, as observed in the included studies, support this proposition, that physical activity over a certain duration can disperse attention, reduce the focus on negative thinking, and thereby alleviate the occurrence of RNT. The most significant point is that this finding aligns with the American College of Sports Medicine’s (ACSM) Exercise Testing and Prescription Guidelines, which recommend engaging in moderate-intensity exercise for at least 30 minutes, 3-5 times per week, to promote and maintain health. This alignment highlights the relevance and practical significance of the current findings for public health initiatives and mental health intervention strategies[65]. This indicates the relevance and applicability of the results to existing guidelines.

## Conclusions

Based on a systematic review and analysis of 14 studies, the following conclusions have been drawn: Physical activity-based interventions have the potential to reduce RNT, with combined interventions that integrate physical activity and psychological training showing even greater effectiveness. This suggests that a holistic approach that simultaneously targets emotional and cognitive components may yield better outcomes. Moreover, intervention protocols characterized by longer durations, moderate frequency, moderate intensity, and extended intervention cycles demonstrate superior improvements in RNT. These findings contribute to a more comprehensive understanding of the mechanisms through which physical activity influences RNT and provide crucial theoretical references for the development of evidence-based intervention strategies.

### Limitations and future directions

While this study provides valuable insights into the potential positive effects of physical activity-based interventions on RNT, certain limitations must be acknowledged. Firstly, the selection criteria used in this study excluded unpublished or non-peer-reviewed materials. Although this approach ensured the inclusion of high-quality, peer-reviewed research, it may have introduced selection bias that could impact the generalizability of the findings. Secondly, the search strategy was limited to English-language publications, potentially overlooking relevant studies in other languages. Expanding future searches to include non-English literature and grey literature could provide a more comprehensive and globally relevant analysis of the impact of physical activity-based interventions on RNT.

Additionally, the limited quantity and diversity of study samples warrant attention. Increasing the number and diversity of participants in future studies will enhance the generalizability of findings. Moreover, the incorporation of meta-analytic techniques will allow for a more robust synthesis of evidence and provide stronger statistical support for the observed intervention effects. Future research should also aim to identify the optimal intervention parameters, such as the most effective types, frequencies, and intensities of physical activity to reduce RNT. Comparative studies focusing on these parameters will provide practical guidance for designing effective intervention protocols.

Lastly, future studies should explore alternative research methodologies, such as empirical and experimental designs, to provide deeper insights into the intervention effects of physical activity on RNT. Empirical studies can offer a more nuanced understanding of causal relationships, thereby strengthening the evidence base for future interventions. By addressing these limitations, future research can contribute to a more comprehensive understanding of how physical activity influences RNT, paving the way for the development of targeted and effective intervention strategies.

## Supporting information

S1 File Data extract and included. https://doi.org/10.6084/m9.figshare.25711734.
(ZIP)

S1 Fig The flowchart summarizes the study selection process based on PRISMA (2020) guidelines. Detailed criteria and methods are described in the main text. (TIF)

S2 Fig This figure was generated using https://smuonco.shinyapps.io/Onlinemeta/
(TIF)

S3 Fig This figure was generated using https://smuonco.shinyapps.io/Onlinemeta/. (TIF)
