# Supplementary material for: Does physical activity-based intervention decrease repetitive negative thinking? A systematic review
Source: PLoS One. 2025 Apr 1;20(4):e0319806. doi: 10.1371/journal.pone.0319806 (PMC11960971; doi:10.1371/journal.pone.0319806)
Supplement: S1 File — https://doi.org/10.6084/m9.figshare.25711734. (ZIP) [file pone.0319806.s001.zip › supporting information/paper file/Gordon 2020.pdf]

scientific reports

Check for updates

OPEN

Resistance exercise training for anxiety and worry symptoms among young adults: a randomized controlled trial

Brett R. Gordon[1,2]✉, Cillian P. McDowell[1,2,3,4], Mark Lyons[1] & Matthew P. Herring[1,2]

This trial quantified the effects of ecologically-valid resistance exercise training (RET) on anxiety and worry symptoms among young adults. Young adults not meeting criteria for subclinical, or analogue Generalized Anxiety Disorder (AGAD) were randomized to an eight-week RET intervention, or eight-week wait-list. AGAD status was determined using validated cut-scores for both the Psychiatric Diagnostic Screening Questionnaire-Generalized Anxiety Disorder subscale (≥ 6) and Penn State Worry Questionnaire (≥ 45). The primary outcome was anxiety symptoms measured with the Trait Anxiety subscale of the State-Trait Anxiety Inventory. The RET was designed according to World Health Organization and American College of Sports Medicine guidelines. RM-ANCOVA examined differences between RET and wait-list over time. Significant interactions were decomposed with simple effects analysis. Hedges' *d* effect sizes quantified magnitude of differences in change between RET and wait-list. Twenty-eight participants (64% female) fully engaged in the trial (mean age: 26.0 ± 6.2y, RET: n = 14; Wait-list: n = 14). A significant group X time interaction was found for anxiety symptoms ($F_{(3,66)} = 3.60$, $p \leq 0.019$; $d = 0.85$, 95%CI: 0.06 to 1.63). RET significantly reduced anxiety symptoms from baseline to post-intervention (mean difference = − 7.89, $p \leq 0.001$). No significant interaction was found for worry ($F_{(3,69)} = 0.79$, $p \geq 0.50$; $d = -0.22$, 95%CI: − 0.96 to 0.53). Ecologically-valid RET significantly improves anxiety symptoms among young adults.
Trial Registration: Clinicaltrials.gov Identifier: NCT04116944, 07/10/2019.

Exercise, a subset of physical activity that is planned, structured, and repetitive, for the purpose of enhancing or maintaining or more components of fitness[1], has well-established effects on anxiety among otherwise healthy adults[2], chronically-ill adults[3], and adults with anxiety disorders[4]. Recent meta-analytic evidence indicated that RET, though understudied compared to aerobic exercise, significantly reduces anxiety among both healthy adults ($\Delta = 0.50$) and those with a physical/mental illness ($\Delta = 0.19$)[5]. However, there is a lack of rigorously designed investigations of ecologically-valid resistance exercise training (RET) that have reported adherence and compliance among young adult males and females. Ecologically-valid RET programs generalize to settings outside of a laboratory setting, are comprised of RET frequency, composition, intensity, progression, and use of standard movements that can be performed at home or at a gym. The only two trials that have randomized healthy young adults to RET[6,7] showed positive effects on anxiety of magnitudes ranging from small ($d = 0.32$)[6] to large ($d = 1.17$)[7]. However, these trials included homogenous samples of males and did not report attendance or compliance to RET, a frequent limitation in this literature[5]. Few trials have examined response to RET designed in accordance with World Health Organization (WHO)[8] and American College of Sports Medicine (ACSM) guidelines[9]. WHO recommends muscle strengthening activities involving major muscle groups two or more days a week[8]; ACSM recommends progressive RET a minimum of two non-consecutive days each week, with 1–3 sets of 8–12 repetitions for muscular strength benefits in novices[9].

The only randomized controlled trial (RCT) of RET among people with an anxiety disorder to date reported improved clinical severity[10], sleep quality and quantity[11], quality of life[12], and associated symptoms[13] among young adult women with GAD. However, the extent to which ecologically-valid RET designed in accordance with WHO and ACSM guidelines improves anxiety and worry symptoms among young adults without an anxiety

[1]Department of Physical Education and Sport Sciences, University of Limerick, Limerick, Ireland. [2]Physical Activity for Health Research Cluster, Health Research Institute, University of Limerick, Limerick, Ireland. [3]The Irish Longitudinal Study On Ageing, Trinity College Dublin, Dublin, Ireland. [4]School of Medicine, Trinity College Dublin, Dublin, Ireland. ✉email: brg5334@psu.edu

disorder is understudied. Examining the potential benefits of guidelines-based RET among those who do not present with clinically-relevant anxiety symptoms provides proof of principle to examine among samples with clinically-relevant anxiety symptoms; based on reductions in symptoms among those without clinically-relevant symptom elevations, larger magnitude reductions among those with more severe symptoms would be anticipated. Thus, the RCT reported here quantified the effects of RET on anxiety and worry symptoms among young adults without AGAD. Based on previous evidence, the authors hypothesized that RET would elicit small magnitude reductions in anxiety symptoms and small magnitude reductions in worry symptoms.

Materials and methods

This trial has adhered to the Consolidated Standards of Reporting Trials (CONSORT) Checklist[14].

Trial design. This manuscript presents findings from one of two parallel RCTs (ClinicalTrials.gov Identifier: NCT04116944, 07/10/2019). The full methods of these RCTs were reported previously[15]. The research protocol was approved by the University of Limerick's Education and Health Sciences Research Ethics Committee (EHSREC No: 2017_03_18_EHS), performed in accordance with the ethical standards as laid down in the 1964 Declaration of Helsinki and its later amendments, and informed consent was obtained from all individual participants included in the study. This trial had rolling recruitment; data collection began January 18th, 2018 and concluded June 26th, 2019.

Participants. Participants were recruited from the surrounding area via posters, emails, and word of mouth. Potential participants initially completed an electronic battery of physical activity and mood questionnaires to establish eligibility; Fig. 1 presents a flowchart of participant recruitment. At baseline, participants completed a battery of online questionnaires, including the 10-item Psychiatric Diagnostic Screening Questionnaire-GAD subscale (PDSQ-GAD)[16] and the 16-item Penn State Worry Questionnaire (PSWQ)[17], followed by several other questionnaires that assessed signs and symptoms of GAD, e.g., state anxiety, feelings of energy and fatigue, irritability, and depressive symptoms. Participants were categorized as either AGAD (PDSQ-GAD ≥ 6 and PSWQ ≥ 45) or non-AGAD. Randomization was conducted utilizing www.randomizer.org to generate a randomized list of codes to indicate assignment to either the RET or wait-list control condition. Participants who did not meet or exceed both cut scores were considered non-AGAD. Participants with AGAD were diverted to the parallel RCT. Following AGAD categorization, participants were randomized, stratified by sex, to RET or a wait-list control condition.

Inclusion criteria were: (1) age 18–40y; (2) not meeting criteria for AGAD; (3) no medical contraindication to safe participation in RET; and, (4) no current pregnancy or lactation. Young adults were recruited because the median age of onset of GAD is 30 years[18]. Current RET involvement at baseline was not an exclusion criterion. Participants were not excluded if they were in treatment for anxiety or other mental health disorders. Participants' previous self-reported RET involvement was measured in weeks. Participants in both groups were advised to maintain their current levels of physical activity throughout the trial. Participants were not compensated. Based on previous meta-analytic evidence of the small-to-moderate effect of RET on anxiety ($\Delta = 0.31$)[5], a priori power analysis with G*Power 3.1 indicated a sample size of 24 (12 in each group) would provide > 80% statistical power (two-tailed $\alpha = 0.05$, four repeated measures) to detect a small-to-moderate effect of RET on anxiety symptoms.

RET intervention. The RET was designed in accordance with WHO and ACSM guidelines[8,9]. The eight-week, twice-weekly intervention increased resistance progressively, such that the participant could complete two sets of between 8 and 12 repetitions of eight exercises before experiencing either fatigue, a deterioration in lifting form noted by the investigator, or failure to complete a repetition. The resistance was stipulated by the investigator in accordance with guidelines rather than self-selected by participants. RET sessions were fully supervised, conducted privately on a one-to-one basis in a RET facility, with no other people in the facility besides the investigator and participant. When necessary, an additional investigator was in the facility for spotting, or investigator training purposes. The eight exercises were barbell squat, barbell bench press, hexagon bar deadlift, seated dumbbell shoulder lateral raise, barbell bent over rows, dumbbell lunges, seated dumbbell curls, and abdominal crunches. Participants randomized to the immediate start RET completed a three-week, twice-weekly, familiarization process to ensure safety, correct lifting technique, and that the entirety of the eight-week intervention was delivered at the correct resistance starting at week one. Further specifics of the RET intervention have been previously published[15].

Control condition. Participants randomized to the wait-list completed questionnaires electronically once a week. Participants that completed the eight-week wait-list condition were subsequently offered the RET intervention, but no data were collected.

Outcomes. Anxiety symptoms were the primary outcome of the trial, and were measured with the trait subscale of the State Trait Anxiety Inventory (STAI-Y2)[19]. The 20-item STAI-Y2 is the most widely-used anxiety measure in the RET for anxiety literature[5], has strong internal consistency[20], and has shown sensitivity to change in response to even short-term RET[13]. Anxiety symptoms were measured at baseline, week one, week four, and post-intervention. Cronbach's α assessed internal consistency of outcome measures. Internal consistency for anxiety symptoms was $\alpha = 0.90$ (ICC = 0.88, 95%CI: 0.81 to 0.94). Correlations between repeated measures were 0.83 ($p \leq 0.001$) and 0.73 ($p \leq 0.003$) for the RET and wait-list groups, respectively.

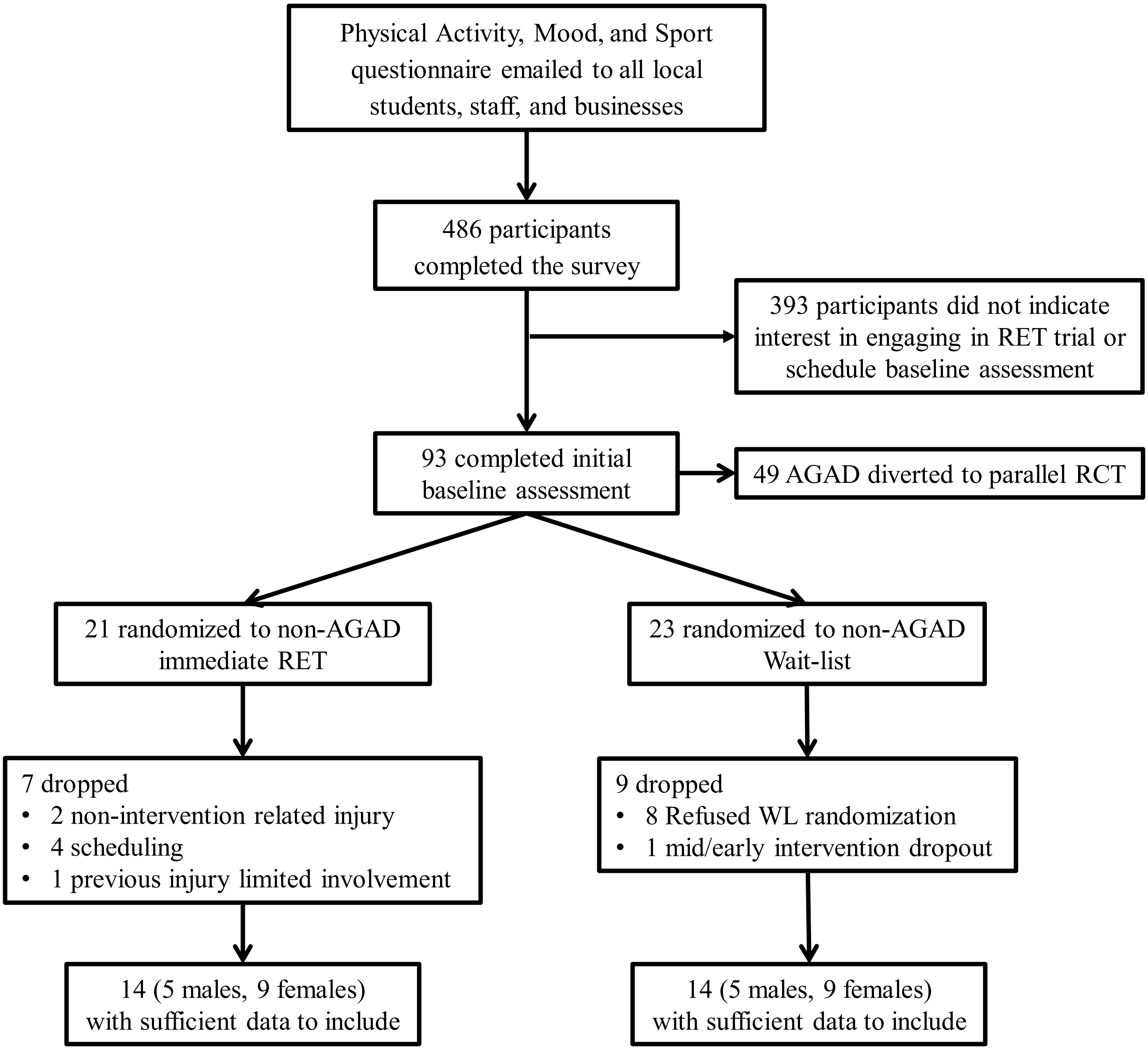

Figure 1. Flow chart of included participants.

Worry symptoms were a secondary outcome assessed using the 16-item PSWQ. Total worry, worry engagement, and absence of worry scores were calculated according to recommendations[21]. The PSWQ has a test–retest reliability of 0.92, strong internal consistency 0.95, and, a score of 45 has shown sensitivity/specificity for GAD of 99/98%[17,22]. Worry was measured at baseline, week one, week four, and post-intervention. Internal consistency for the PSWQ was $\alpha = 0.92$ (ICC = 0.88, 95%CI: 0.80 to 0.94). Correlations between repeated measures were 0.90 ($p \le 0.001$) and 0.89 ($p \le 0.001$) for the RET and wait-list group respectively. Internal consistency for worry engagement was $\alpha = 0.90$ (ICC = 0.87, 95%CI: 0.78 to 0.93). Correlations between repeated measures were 0.86 ($p \le 0.001$) and 0.89 ($p \le 0.001$) for the RET and wait-list group respectively. Internal consistency for absence of worry was $\alpha = 0.84$ (ICC = 0.76, 95%CI: 0.54 to 0.88). Correlations between repeated measures were 0.92 ($p \le 0.001$) and 0.51 ($p \ge 0.06$) for the RET and wait-list group respectively.

Covariates. Baseline physical activity was assessed using an online, self-report version of the seven-day Physical Activity Recall[23]. Participants reported time engaged in sleep, moderate, hard, and very hard activities during the prior week. Estimated energy expenditure was calculated as kilocalories per week.

Intervention fidelity and manipulation check. Attendance was calculated by dividing the number of RET bouts attended by 16 (2 sessions per week × 8 weeks). Compliance was calculated by dividing the number of sets in which at least eight repetitions were completed by 256 (2 sets × 8 exercises × 2 sessions per week × 8 weeks), which represents the prescribed number of repetitions. To quantify anticipated changes in objective muscular

Variable	RET (n = 14) Mean (SD)	WL (n = 14) Mean (SD)	*t*	*p*	Cohen's d
% Female	64	64			
Age (y)	25.2 (5.7)	28.4 (6.6)	− 1.39	0.18	− 0.53 (− 1.28 to 0.23)
Body mass index	24.9 (4.3)	22.9 (2.9)	1.40	0.16	0.54 (− 0.22 to 1.28)
Symptoms of GAD (PDSQ-GAD)	2.7 (1.7)	1.1 (1.9)	0.95	0.35	0.36 (− 0.39 to 1.11)
Worry symptoms (PSWQ)	47.8 (11.0)	47.3 (11.3)	0.18	0.91	0.07 (− 0.67 to 0.81)
Worry-engagement (PSWQ-WE)	30.7 (8.6)	29.8 (7.9)	0.30	0.77	0.01 (− 0.63 to 0.85)
Absence of worry (PSWQ-AW)	17.1 (3.5)	17.5 (4.5)	− 0.28	0.78	− 0.11 (− 0.85 to 0.64)
Anxiety symptoms (STAI-Y2)	39.9 (7.9)	36.7 (9.1)	0.98	0.34	0.38 (− 0.38 to 1.12)
Physical activity (kcals/week)	270.7 (31.2)	264.8 (28.8)	0.47	0.65	0.18 (− 0.56 to 0.92)

Table 1. Baseline differences among resistance exercise training and wait-list. *SD* standard deviation, *PDSQ-GAD* psychiatric diagnostic screening questionnaire-generalized anxiety disorder subscale, *PSWQ* Penn state worry questionnaire, *PSWQ-WE* Penn state worry questionnaire-worry engagement, *PSWQ-AW* Penn state worry questionnaire-absence of worry, *RET* resistance exercise training, *STAI-Y2* trait anxiety inventory, *WL* wait-list, *y* years.

strength as a manipulation check, and to facilitate setting of load, participants completed a five repetition maximum (5RM) assessment for the barbell squat, barbell bench press, and hexagon bar deadlift at baseline and post-intervention. During the six familiarization sessions, participants completed two familiarizations with the 5RM process, and one maximal 5RM assessment. Rate of perceived exertion (6–20)[24] and a muscle soreness scale (1–10) were measured following the completion of each exercise.

Statistical analyses. Data analyses were performed using SPSS 26.0. Missing data for STAI-Y2 (n = 5), and worry (n = 4) were imputed: gender and time-variant responses for each variable were entered as predictors into separate multiple linear regression models for condition, and predicted values were retained. Participants (n = 10) were excluded if they were missing primary outcome data at > 1 time point. Independent samples *t*-tests examined baseline differences in age, body mass index, physical activity, anxiety symptoms, and worry symptoms between groups and based on sex. The magnitude of baseline differences were quantified using Cohen's d effect sizes[25]. Two group (RET/wait-list) x four time (baseline/week one/week four/week eight) RM-ANCOVA examined differences between RET and wait-list, controlling for age, sex, and baseline physical activity. Significant interactions were decomposed using simple effects analysis. The magnitude of within-condition change was quantified using standardized mean difference (SMD). Associations between changes in strength and changes in anxiety and worry symptoms were quantified using Pearson correlation coefficients of associations between change scores. The magnitude of difference in outcome change between groups was quantified by Hedges' *d* effect sizes and associated 95%Cs[25]. Intention-to-treat analyses, analyses of complete cases only, and analyses without controlling for baseline physical activity are reported as sensitivity analyses. Hedges' *d* effect sizes were calculated by subtracting the mean change in the wait-list from the mean change in the RET condition, and dividing this difference by the pooled standard deviation of baseline scores, and adjusted for small sample size bias. Effect sizes are calculated such that improved outcomes in each condition and larger improvements among RET compared to wait-list resulted in positive effect sizes. Changes in strength were examined with paired-sample *t*-tests. The NNT and associated 95%CIs were converted from Hedges' *d* and calculated as the inverse of the absolute risk reduction for RET compared with the wait-list condition. NNT was rounded up to the nearest whole number[26]. De-identified individual participant data for primary outcomes measures analysed during the current study will be made available for five years at six months following publication of primary outcome measures summary data.

Ethics statement. The research protocol was approved by the University's Research Ethics Committee (2017_03_18_EHS), and performed in accordance with the ethical standards as laid down in the 1964 Declaration of Helsinki and its later amendments.

Results

Table 1 presents baseline participant characteristics and differences between groups. There were no baseline differences between groups on any outcomes, supporting successful randomization. There were no baseline differences between sexes on any outcomes. Although not an exclusion criterion, all participants included in analyses had a training age of zero, meaning they were not engaged in structured resistance exercise training. One participant currently receiving treatment for depression withdrew from the wait-list. No participants reported receiving treatment for GAD.

Intervention fidelity and manipulation check. The average attendance to the RET intervention was 85% (13 out of 16 sessions). The average compliance with RET was 83% (212 out of 256 repetitions). The average rate of perceived exertion was 14 ± 1 (in between somewhat hard and hard), and average muscle soreness was

Outcome	Group	Baseline	Week 1	SMD	Hedges' *d*	Week 4	SMD	Hedges' *d*	Week 8	SMD	Hedges' *d*
Anxiety Symptoms (STAI-Y2)	RET	39.9 (7.9)	36.3 (10.6)	− 0.45	0.51 (− 0.25 to 1.28)	36.2 (10.5)	− 0.46	− 0.35 (− 0.41 to 1.11)	31.8 (8.0)*	1.03	0.85 (0.06 to 1.63)
	WL	36.7 (9.1)	37.6 (8.6)	0.10		36.2 (9.8)	− 0.06		36.0 (9.5)	− 0.08	
Worry Symptoms (PSWQ)	RET	47.8 (11.0)	45.2 (11.0)	− 0.23	0.02 (− 0.72 to 0.76)	46.1 (10.5)	− 0.15	− 0.19 (− 0.93 to 0.56)	44.4 (11.8)	− 0.31	− 0.22 (− 0.96 to 0.53)
	WL	47.3 (11.3)	44.9 (11.8)	− 0.21		43.5 (15.3)	− 0.33		41.4 (16.0)*	− 0.52	
Worry-Engagement (PSWQ-WE)	RET	30.7 (8.6)	30.1 (8.9)	− 0.08	− 0.19 (− 0.94 to 0.55)	29.8 (7.0)	− 0.11	− 0.28 (− 1.02 to 0.47)	28.4 (8.8)	− 0.27	− 0.20 (− 0.94 to 0.54)
	WL	29.8 (7.9)	27.5 (8.6)	− 0.29		26.5 (10.9)	− 0.42		25.8 (10.3)*	− 0.51	
Absence of Worry (PSWQ-AW)	RET	17.1 (3.5)	15.1 (3.9)	− 0.58	0.46 (− 0.29 to 1.21)	15.7 (4.6)	− 0.40	0.23 (− 0.51 to 0.97)	15.9 (5.0)	− 0.36	− 0.18 (− 0.92 to 0.57)
	WL	17.5 (4.5)	17.4 (4.5)	− 0.02		17.1 (5.1)	− 0.10		15.6 (6.2)	− 0.43	

Table 2. Changes in anxiety and worry symptoms. RM-ANCOVA controlling for sex, age, and non-intervention physical activity; *indicates a significant difference from the baseline score in simple effects analyse. *SMD* standardized mean difference, *RET* resistance exercise training, *WL* wait-list, *STAI-Y2* trait anxiety inventory, *PSWQ* Penn state worry questionnaire, *PSWQ-WE* Penn state worry questionnaire-worry engagement, *PSWQ-AW* Penn state worry questionnaire-absence of worry.

	Hedges' *d*	Hedges' *d*	Hedges' *d*
Outcome	Baseline-Week 1	Week 1-Week 4	Week 4-Week 8
Anxiety symptoms (STAI-Y2)	0.51 (− 0.25 to 1.28)	− 0.14 (− 0.90 to 0.61)	0.42 (− 0.35 to 1.18)
Worry symptoms (PSWQ)	0.02 (− 0.72 to 0.76)	− 0.20 (− 0.94 to 0.54)	− 0.03 (− 0.77 to 0.71)
Worry-engagement (PSWQ-WE)	− 0.19 (− 0.94 to 0.55)	− 0.08 (− 0.82 to 0.66)	0.07 (− 0.67 to 0.81)
Absence of worry (PSWQ-AW)	0.46 (− 0.29 to 1.21)	− 0.21 (− 0.96 to 0.53)	− 0.33 (− 1.07 to 0.42)

Table 3. Changes in outcomes by time-points of intervention. *STAI-Y2* trait anxiety inventory, *PSWQ* Penn state worry questionnaire, *PSWQ-WE* Penn state worry questionnaire-worry engagement, *PSWQ-AW* Penn state worry questionnaire-absence of worry.

4 ± 2 out of 10. As anticipated, participants in the RET intervention significantly increased their strength ($t_{(13)}$ = − 6.75, $p \leq 0.001$, Cohen's d = 2.04, mean increase: 23.4% ± 14.7).

Anxiety and worry severity. Table 2 presents descriptives, SMD, and Hedges' *d* (95%CI) for outcomes. Based on the magnitude of worry reduction, there was a NNT of 3 (95%CI: 2–37). There was a significant group X time interaction for anxiety symptoms ($F_{(3,66)} = 3.60$, $p \leq 0.019$; $d = 0.85$, 95%CI: 0.06–1.63). RET significantly reduced anxiety symptoms from baseline to post-intervention (mean difference = − 7.89, $p \leq 0.001$). There was no significant interaction for worry ($F_{(3,69)=}$0.79, $p \geq 0.51$, $d = -0.22$, 95%CI: − 0.96 to 0.53), worry engagement ($F_{(3,69)} = 0.37$, $p \geq 0.79$, $d = -0.20$, 95%CI: − 0.94 to 0.54), or absence of worry ($F_{(3,69)} = 1.81$, $p \geq 0.16$, $d = -0.18$, 95%CI: − 0.92 to 0.57). RM-ANCOVA findings did not differ upon removal of baseline physical activity as a covariate for all outcomes. There was a significant group X time interaction for anxiety symptoms ($F_{(3,69)} = 3.41$, $p \leq 0.02$), and no significant interaction for worry, worry-engagement, or absence of worry (all $p \geq 0.16$). The magnitude of outcome change did not materially differ in intention-to-treat or complete case analyses for all outcomes (Supplement 1). Although both the RET and wait-list reduced worry, Hedges' *d* effect sizes were negative, as greater reductions in worry occurred in the wait-list. Table 3 presents Hedges' *d* (95%CI) for outcomes through time-points of the intervention. Changes in strength were not significantly associated with changes in anxiety symptoms ($r_{(14)} = 0.07$, $p = 0.83$), worry symptoms ($r_{(14)} = -0.19$, $p = 0.55$), worry engagement ($r_{(14)} = 0.16$, $p = 0.63$), or absence of worry ($r_{(14)} = -0.39$, $p = 0.21$).

Discussion

Compared to an eight-week wait-list control condition, ecologically-valid RET, designed according to WHO and ACSM guidelines, significantly reduced anxiety symptoms in a non-anxiety disordered young adult sample. This RCT specifically addressed recent calls from the United States Physical Activity Guidelines Advisory Committee Scientific Report to conduct RCTs among individuals at different stages or severity of impairment (i.e., AGAD) to examine whether physical activity delays or prevents disease onset and progression[27]. Disease onset and progression is particularly relevant to young adults with AGAD, as those who display elevated subclinical symptoms are more likely to develop clinically significant psychopathology[28], and intervening at this point in the severity spectrum could alleviate future burden through the preventative capacity of exercise training. The large magnitude reductions in anxiety found here ($d = 0.85$) are larger than previous meta-analytic evidence of the effects of RET on anxiety symptoms among healthy adults of all ages ($\Delta = 0.50$)[5]. The large reductions in anxiety symptoms here are clinically meaningful, based on a frequently used response threshold of a 50% or greater

reduction in baseline scores, or on a minimally important difference threshold of 0.5 standard deviation units[29]. The magnitude of anxiety reductions is larger than the effect of aerobic-based physical activity interventions in healthy adults ($\Delta = 0.45$)[2], and larger than the effect of RET among young adult women with GAD ($d = 0.52$)[13]. Even though participants were deliberately screened for lower levels of worry, both RET and wait-list exhibited small magnitude reductions in worry.

Greater magnitude reductions in anxiety symptoms occurred following initial familiarization to week one ($d = 0.51$, 95%CI: − 0.25 to 1.28), and subsequently from week four to post-intervention ($d = 0.42$, 95%CI: 0.35 to 1.18), than from week one to week four ($d = -0.14$ 95%CI: − 0.90 to 0.61). These stepped reductions indicate that participants have immediate improvements in anxiety symptoms upon beginning an RET intervention, and even if reductions briefly plateau, the reductions continue with further RET engagement[13]. As predicted, there were large reductions in anxiety symptoms in the RET group, and relatively little change in the wait-list. A non-significant, negative effect for worry ($d = -0.22$, 95%CI − 0.96 to 0.53) was not expected. However, the negative Hedges' *d* effect size occurred due to worry improvements in both groups, with non-significantly larger (albeit both small magnitude) improvements in the wait-list. Ideally, participants assigned to the wait-list control condition experience no change in anxiety symptoms or worry. However, small, non-significant changes in either direction were expected, as meta-analytic evidence has shown that wait-lists may result in improvement[30] and worsening of outcomes[31] among those with anxiety disorders. In the context of exercise interventions for mental health, placebo/sham exercise interventions have positive effects on subjective outcomes[32]. Although the magnitude of worry reduction was affected by improvements in the wait-list in this trial, moderate-to-large magnitude improvements in worry in either group were not expected, as participants were deliberately screened for reduced worry.

Adherence of 85% and compliance to RET of 83% supports that the RET intervention was feasible and tolerable. These attendance and compliance rates indicate that participants missed between 2–3 sessions over the intervention, but were compliant when they attended. Furthermore, no adverse events to the RET occurred. Physical activity participation at the population level is low[33], and even lower in individuals with mental health issues[34]. However, participants who completed the trial (n = 28) had significantly greater worry ($t_{(43)} = 2.42$, $p \leq 0.02$), and PDSQ-GAD symptoms ($t_{(43)} = 2.01$ $p \leq 0.0504$) at baseline than those who dropped out (n = 16), supporting the feasibility of interventions in those with even higher levels of worry.

Although the participants in this trial did not meet criteria for AGAD, quantified by scores at or above cut scores for both the PSWQ and PDSQ-GAD subscale, the mean PSWQ for both groups was ≥ 45, indicating some elevated worry. Very little information exists regarding the prevalence of elevated worry among young adults. Although recruitment for these trials targeted individuals with and without AGAD, and, as much as possible, aimed to shield the overall hypothesis and focus of the trial from participants, recruitment for the trial may have attracted a larger proportion of worried individuals. Of the 93 participants who completed the baseline screening, 49 (53 %) met AGAD criteria, indicating a prevalence of worry in the small population of potential participants. However, among the non-AGAD participants, the mean PDSQ-GAD scores in both conditions were well below established cut scores sensitive and specific to GAD.

Although meta-analytic evidence has not supported that significant improvements in strength are required for mental health benefit[5,35], as anticipated, participants showed large significant improvements in strength (all $d \geq 1.53$). These associated strength changes may have important implications. For example, physical health and performance variables explain significant variation in physical activity among older adults with probable GAD beyond variation explained by sociodemographics and other health behaviours[36], and increased grip strength is associated with lower odds of developing GAD[37]. Given that GAD risk typically arises during the teen years and progresses relatively linearly[38], RET interventions targeting these age groups, who may otherwise not be engaging in any muscle strengthening activity, are particularly important. In the United States, 70% of adults do not meet WHO muscle strengthening guidelines[39].

Due to the progressive increase in weight of the RET protocol, participants engaged in the largest dose of RET at the end of the intervention, when their improvements in strengths allowed them to engage in more intense RET. Meta-analytic evidence supports a dose–response relationship between physical activity and anxiety epidemiologically[40] and experimentally[5]; consistent with this previous evidence, effect sizes in this trial were largest at the end of the intervention ($d = 0.85$) when the dose was largest. Previous meta-analytic evidence indicated that the anxiolytic effects of RET were not moderated by features of the RET stimulus[5]. There currently are no RET guidelines for mental health. As such, this investigation focused on pre-existing WHO and ACSM guidelines for RET for physical health. To further examine dose, future trials should rigorously report details of the RET stimulus, and randomize participants to RET of low, moderate, and vigorous intensity to explore intensity and dose–response as moderators of the anxiolytic effect of RET. RET trials of varying intensities and frequencies may also identify the minimum effective dose, and maximal tolerated dose of RET for anxiety and worry symptoms among healthy individuals and individuals with a physical or mental illness.

Though identifying mechanisms of action was beyond the scope of the present investigation, there are several potential social and psychobiological mechanisms that may explain why these large magnitude reductions in anxiety occurred in non-anxiety disordered young adults engaging in an RET intervention. Although social interaction was rigorously controlled[14], RET participants may have benefited from the increased amount of social interaction during the exercise bouts, as every bout was supervised. Other putative mechanisms include the expectancy of improved mental health following exercise[41], or feelings of mastery following the progressive increases in strength[42]. Due to the progressive nature of the RET protocol, participants continuously achieved goals set by themselves relative to weight/reps on individual exercises. These achievements could have improved self-efficacy, which possibly mediated the relationship between exercise and anxiety reductions through producing mastery experiences. It is important to note that although progress on lifts was documented to appropriately increase resistance, no RET goals were set by investigators, or formalized in any capacity. Psychobiological

mechanisms involve systems that are involved in both how anxiety develops and how exercise affects the brain[43]. More research is needed regarding the specific mechanisms underlying exercise effects on anxiety and worry, particularly those that may be unique to RET. Potential RET specific mechanisms include increases in IGF-1)[44] and reductions in inflammation during RET[45]. Evidence from animal models for RET effects on anxiety-like behaviour are limited, as there are several design issues related to how to most appropriately model RET in rodents. Common models, such as tail-weighted ladder climbs, may be anxiogenic, as they often involve shock during training[46]. Unlike aerobic exercise, in which wheel-running fairly accurately reflects aerobic exercise engagement, there are less homologous RET models for rodents. However, new models, such as burrowing, or unweighted tower climbing, are emerging[47].

The anxiety and worry reductions found here are generalizable to the larger population. Compared to wait-list, the anxiety reductions for RET expressed as a function of absolute risk reduction[26] resulted in a NNT of three, such that anxiety reductions would be expected to occur for at least one of every three participants who would engage in this ecologically-valid RET. The population of young adults age 18–40 y in Ireland is ~ 1.5 million[48]. If the entirety of the population were compliant with guidelines-based RET, anxiety reductions demonstrated here would be expected to occur in approximately 500,000 young adults. Although it would be logistically impossible to provide one-on-one RET at such a large scale, there are innumerable ways to engage in ACSM and WHO guidelines-based RET at minimal cost and equipment needs.

Although this trial was sufficiently powered to detect small-to-moderate reductions in worry, future trials would benefit from larger sample sizes to explore potential sex-related response differences to RET, and plausible mediators/moderators of response. To ensure safety, compliance, rigorous delivery of the RET intervention, and control for social interaction, each session had to be coordinated between the participant, supervisor, and facility availability. For all missed sessions, an attempt was made to make-up the sessions at a later time/day in the week to ensure maximum compliance. This can be particularly burdensome with a limited staff of investigators continuously available to adjust to participant schedules. Participants who could not fit a session into their schedule that week were not asked to engage in the RET on their own; however, at the expense of controlling for social interaction, future trials could incorporate a home-based, guidelines-based resistance-band/body weight supplemented exercise protocol to maximize RET compliance when sessions must be missed. Additionally, although pain was assessed with a 10-point Likert scale following each exercise as a component of trial fidelity and safety, GAD often co-occurs with pain[49], and other dimensions of pain were not assessed. Physical activity is associated with pain indirectly via symptoms of panic and somatization disorder[50]; failing to measure and examine prevalence of persistent pain and pain response to RET is a limitation.

Conclusions

Ecologically-valid, guidelines-based RET significantly improved anxiety symptoms among young adults. Future trials should replicate and expand these findings to explore sex-related differences, examine putative biological, cognitive, and psychological mechanisms for the anxiolytic effects of RET, and augment other established treatments for anxiety, such as cognitive behavioural therapy and pharmacotherapy, with RET.

Data availability

De-identified individual participant data for primary outcomes measures analysed during the current study will be made available for five years at six months following publication of primary outcome measures summary data.

Received: 26 April 2020; Accepted: 29 September 2020
Published online: 16 October 2020

References

1. Caspersen, C. J., Powell, K. E. & Christenson, G. M. Physical activity, exercise, and physical fitness: definitions and distinctions for health-related research. *Public Health Rep.* **100**(2), 126 (1985).
2. Conn, V. S. Anxiety outcomes after physical activity interventions: meta-analysis findings. *Nurs. Res.* **59**(3), 224. https://doi.org/10.1097/NNR.0b013e3181dbb2f8 (2010).
3. Herring, M. P., O'Connor, P. J. & Dishman, R. K. The effect of exercise training on anxiety symptoms among patients: a systematic review. *AMA Arch. Int. Med.* **170**(4), 321–331. https://doi.org/10.1001/archinternmed.2009.530 (2010).
4. Stubbs, B. *et al.* An examination of the anxiolytic effects of exercise for people with anxiety and stress-related disorders: a meta-analysis. *Psychiatry Res.* **249**, 102–108. https://doi.org/10.1016/j.psychres.2016.12.020 (2017).
5. Gordon, B. R., McDowell, C. P., Lyons, M. & Herring, M. P. The effects of resistance exercise training on anxiety: a meta-analysis and meta-regression analysis of randomized controlled trials. *Sports Med.* **47**(12), 2521–2532. https://doi.org/10.1007/s40279-017-0769-0 (2017).
6. Rahmani-Nia, F., Arazi, H., Rahimi, R., Piri-Kurd, K. & Hossaini, K. Effects of an eight-week circuit strength training program on the body images and anxiety in untrained college students. *Medicina Dello Sport* **64**(3), 297–308 (2011).
7. Norvell, N. & Belles, D. Psychological and physical benefits of circuit weight training in law enforcement personnel. *J. Consult. Clin. Psychol.* **61**(3), 520–527. https://doi.org/10.1037//0022-006x.61.3.520 (1993).
8. World Health Organization. *Global Recommendations on Physical Activity for Health*. 2010. https://www.who.int/dietphysicalactivity/global-PA-recs-2010.pdf. Accessed November 15th, 2019.
9. American College of Sports Medicine. American College of Sports Medicine position stand. Progression models in resistance training for healthy adults. *Med. Sci. Sports Exerc.* **41**(3), 687–708. https://doi.org/10.1249/MSS.0b013e3181915670 (2009).
10. Herring, M. P., Jacob, M. L., Suveg, C., Diskman, R. K. & O'Connor, P. J. Feasibility of exercise training for the short-term treatment of generalized anxiety disorder: a randomized controlled trial. *Psychother. Psychosom.* **81**(1), 21–28. https://doi.org/10.1159/000327898 (2012).
11. Herring, M. P., Kline, C. E. & O'Connor, P. J. Effects of exercise training on self-reported sleep among young women with generalized anxiety disorder. *Ment. Health Phys. Act.* **9**, 59–66. https://doi.org/10.1016/j.mhpa.2015.09.002 (2015).
12. Herring, M. P., Johnson, K. E. & O'Connor, P. J. Exercise training and health-related quality of life in generalized anxiety disorder. *Psychol. Sport Exerc.* **27**, 138–141. https://doi.org/10.1016/j.psychsport.2016.08.011 (2016).

13. Herring, M. P., Jacob, M. L., Suveg, C., Dishman, R. K. & O'Connor, P. J. Effects of short-term exercise training on signs and symptoms of generalized anxiety disorder. *Ment. Health Phys. Act.* **4**(2), 71–77. https://doi.org/10.1016/j.mhpa.2011.07.002 (2011).
14. Schulz, K. F., Altman, D. G. & Moher, D. CONSORT 2010 statement: updated guidelines for reporting parallel group randomised trials. *BMC Med.* **8**, 18. https://doi.org/10.1186/1741-7015-8-18 (2010).
15. Gordon, B. R., McDowell, C. P., Lyons, M. & Herring, M. P. Acute and chronic effects of resistance exercise training among young adults with and without analogue generalized anxiety disorder: a protocol for pilot randomized controlled trials. *Men. Health Phys. Act.* **18**, 100321. https://doi.org/10.1016/j.mhpa.2020.100321 (2020).
16. Zimmerman, M. & Mattia, J. I. A self-report scale to help make psychiatric diagnoses: the psychiatric diagnostic screening questionnaire. *JAMA Psychiatry* **58**(8), 787–794. https://doi.org/10.1001/archpsyc.58.8.787 (2001).
17. Meyer, T. J., Miller, M. L., Metzger, R. L. & Borkovec, T. D. Development and validation of the Penn state worry questionnaire. *Behav. Res Ther.* **28**(6), 487–495. https://doi.org/10.1016/0005-7967(90)90135-6 (1990).
18. Bandelow, B. & Michaelis, S. Epidemiology of anxiety disorders in the 21st century. *Dialogues Clin. Neurosci.* **17**(3), 327 (2015) (**PMID: 26487813**).
19. Spielberger, C. D. *State-Trait Anxiety Inventory for Adults* 2nd edn. (Consulting Psychologists Press, Palo Alto, 1983).
20. Barnes, L. L., Harp, D. & Jung, W. S. Reliability generalization of scores on the Spielberger state-trait anxiety inventory. *Educ. Psychol. Meas.* **62**(4), 603–618. https://doi.org/10.1177/0013164402062004005 (2002).
21. Fresco, D. M., Heimberg, R. G., Mennin, D. S., Turk, C. L. & Heimberg, R. G. Confirmatory factor analysis of the Penn state worry questionnaire. *Behav. Res. Ther.* **40**(3), 313–323. https://doi.org/10.1016/s0005-7967(00)00113-3 (2002).
22. Behar, E., Alcaine, O., Zuellig, A. R. & Borkovec, T. D. Screening for generalized anxiety disorder using the Penn state worry questionnaire: a receiver operating characteristic analysis. *J. Behav. Ther. Exp. Psychiatry* **34**(1), 25–43. https://doi.org/10.1016/s0005-7916(03)00004-1 (2003).
23. Blair, S. N. *et al.* Assessment of habitual physical activity by a seven day recall in a community survey and controlled experiments . *Am. J. Epidemiol.* **122**(5), 794–804. https://doi.org/10.1093/oxfordjournals.aje.a114163 (1985).
24. Borg, G. & Ottoson, D. *Perception of Exertion in Physical Exercise* (Macmillan Press Limited, London, 1986).
25. Hedges, L. V. & Olkin, I. *Statistical Methods for Meta-Analysis* (Academic Press, New York, 1985).
26. Cook, R. J. & Sackett, D. L. The number needed to treat: a clinically useful measure of treatment effect. *BMJ* **310**(6977), 452–454. https://doi.org/10.1136/bmj.310.6977.452 (1995).
27. United States Physical Activity Guidelines Advisory Committee. 2018 *Physical Activity Guidelines Advisory Committee Scientific Report*. Department of Health and Human Services; 2018. https://health.gov/paguidelines/second-edition/report/. Accessed December 03th, 2019.
28. Wolitzky-Taylor, K. *et al.* Experiencing core symptoms of anxiety and unipolar mood disorders in late adolescence predicts disorder onset in early adulthood. *Depress Anxiety* **31**(3), 207–213. https://doi.org/10.1002/da.22250 (2014).
29. Rush, A. J. *et al.* The 16-item quick inventory of depressive symptomatology (qids), clinician rating (qids-c), and self-report (qids-sr): a psychometric evaluation in patients with chronic major depression. *Bio Psychiatr.* **54**(5), 573–583. https://doi.org/10.1016/S0006-3223(02)01866-8 (2003).
30. Devilly, G. J. & McFarlane, A. C. When wait lists are not feasible, nothing is a thing that does not need to be done. *J. Consult. Clin. Psychol.* **77**(6), 1159. https://doi.org/10.1037/a0016878 (2009).
31. Patterson, B., Boyle, M. H., Kivlenieks, M. & Van Ameringen, M. The use of waitlists as control conditions in anxiety disorders research. *J. Psychiatr. Res.* **83**, 112–120. https://doi.org/10.1016/j.jpsychires.2016.08.015 (2016).
32. Lindheimer, J. B., O'Connor, P. J. & Dishman, R. K. Quantifying the placebo effect in psychological outcomes of exercise training: a meta-analysis of randomized trials. *Sports Med.* **45**(5), 693–711. https://doi.org/10.1007/s40279-015-0303-1 (2015).
33. Bauman, A. E. *et al.* Correlates of physical activity: why are some people physically active and others not?. *Lancet* **380**(9838), 258–271. https://doi.org/10.1016/S0140-6736(12)60735-1 (2012).
34. McDowell, C. P. *et al.* Physical activity and generalized anxiety disorder: results from the Irish longitudinal study on ageing (TILDA). *Int. J. Epidemiol.* **47**(5), 1443–1453. https://doi.org/10.1093/ije/dyy141 (2018).
35. Gordon, B. R. *et al.* Association of efficacy of resistance exercise training with depressive symptoms: meta-analysis and meta-regression analysis of randomized clinical trials. *JAMA Psychiatry* **75**(6), 566–576. https://doi.org/10.1001/jamapsychiatry.2018.0572 (2018).
36. McDowell, C. P., Gordon, B. R., MacDonncha, C. & Herring, M. P. Physical activity correlates among older adults with probable generalized anxiety disorder: results from the Irish longitudinal study on ageing. *Gen. Hosp. Psychiatry* **59**, 30–36. https://doi.org/10.1016/j.genhosppsych.2019.04.012 (2019).
37. Gordon, B. R., McDowell, C. P., Lyons, M. & Herring, M. P. Associations between grip strength and generalized anxiety disorder in older adults: results from the Irish longitudinal study on ageing. *J. Affect Disord.* **255**, 136–141. https://doi.org/10.1016/j.jad.2019.05.043 (2019).
38. Lieb, R., Becker, E. & Altamura, C. The epidemiology of generalized anxiety disorder in Europe. *Eur. Neuropsychopharmacol.* **15**(4), 445–452. https://doi.org/10.1016/j.euroneuro.2005.04.010 (2005).
39. Bennie, J. A. *et al.* Muscle strengthening, aerobic exercise, and obesity: a pooled analysis of 1.7 million us adults. *Obesity* **28**(2), 371–378. https://doi.org/10.1002/oby.22673 (2020).
40. McDowell, C. P., Dishman, R. K., Gordon, B. R. & Herring, M. P. Physical activity and anxiety: a systematic review and meta-analysis of prospective cohort studies. *Am. J. Prev. Med.* **57**(4), 545–556. https://doi.org/10.1016/j.amepre.2019.05.012 (2019).
41. Lindheimer, J. B., Szabo, A., Raglin, J. S. & Beedie, C. Advancing the understanding of placebo effects in psychological outcomes of exercise: lessons learned and future directions. *Eur. J. Sport Sci.* **9**, 1–12. https://doi.org/10.1080/17461391.2019.1632937 (2019).
42. Petruzzello, S. J., Landers, D. M., Harfield, B. D., Kubitz, K. A. & Salazar, W. A meta-analysis on the anxiety-reducing effects of acute and chronic exercise. *Sports Med.* **11**(3), 143–182. https://doi.org/10.2165/00007256-199111030-00002 (1991).
43. Dishman, R. K. *et al.* Neurobiology of exercise. *Obesity* **14**(3), 345–356. https://doi.org/10.1038/oby.2006.46 (2006).
44. Cassilhas, R. C. *et al.* Spatial memory is improved by aerobic and resistance exercise through divergent molecular mechanisms. *Neuroscience* **202**, 309–317. https://doi.org/10.1016/j.neuroscience.2011.11.029 (2012).
45. Olson, T. P., Dengel, D., Leon, A. & Schmitz, K. H. Changes in inflammatory biomarkers following one-year of moderate resistance training in overweight women. *Int. J. Obes.* **31**(6), 996. https://doi.org/10.1038/sj.ijo.0803534 (2007).
46. Strickland, J. C. & Smith, M. A. Animal models of resistance exercise and their application to neuroscience research. *J. Neurosci. Methods.* **273**, 191–200. https://doi.org/10.1016/j.jneumeth.2016.08.003 (2016).
47. Roemers, P. *et al.* Burrowing as a novel voluntary strength training method for mice: a comparison of various voluntary strength or resistance exercise methods. *J. Neurosci. Methods* **300**, 112–126. https://doi.org/10.1016/j.jneumeth.2017.05.027 (2018).
48. Central Statistics Office. Census 2016 Summary Results. 2016. https://www.cso.ie/en/csolatestnews/presspages/2017/census2016summaryresults-part1/. Accessed November 15th, 2019.
49. Csupak, B., Sommer, J. L., Jacobsohn, E. & El-Gabalawy, R. A. A population-based examination of the co-occurrence and functional correlates of chronic pain and generalized anxiety disorder. *J. Anxiety Disord.* **56**, 74–80. https://doi.org/10.1016/j.janxdis.2018.04.005 (2018).
50. O'Connor, P. J., Herring, M. P., McDowell, C. P. & Dishman, R. K. Physical activity is indirectly associated with pain in college women through associations with somatization and panic disorder symptoms: a cross-sectional study. *Ann. Epidemiol.* **33**, 37–43. https://doi.org/10.1016/j.annepidem.2019.02.008 (2019).

Author contributions
All authors made substantial contributions to conception and design, acquisition of data, and preparation of the manuscript. All authors have read and approved the final version of the manuscript, and agree with the order of presentation of the authors.

Funding
Brett R. Gordon was funded by the Irish Research Council under the Government of Ireland Postgraduate Programme. Cillian P. McDowell is funded by the Irish Research Council under the Government of Ireland Postdoctoral Programme.

Competing interests
The authors declare no competing interests.

Additional information
Supplementary information is available for this paper at https://doi.org/10.1038/s41598-020-74608-6.

Correspondence and requests for materials should be addressed to B.R.G.

Reprints and permissions information is available at www.nature.com/reprints.

Publisher's note Springer Nature remains neutral with regard to jurisdictional claims in published maps and institutional affiliations.

Open Access This article is licensed under a Creative Commons Attribution 4.0 International License, which permits use, sharing, adaptation, distribution and reproduction in any medium or format, as long as you give appropriate credit to the original author(s) and the source, provide a link to the Creative Commons licence, and indicate if changes were made. The images or other third party material in this article are included in the article's Creative Commons licence, unless indicated otherwise in a credit line to the material. If material is not included in the article's Creative Commons licence and your intended use is not permitted by statutory regulation or exceeds the permitted use, you will need to obtain permission directly from the copyright holder. To view a copy of this licence, visit http://creativecommons.org/licenses/by/4.0/.

© The Author(s) 2020
