# Supplementary material for: Does physical activity-based intervention decrease repetitive negative thinking? A systematic review
Source: PLoS One. 2025 Apr 1;20(4):e0319806. doi: 10.1371/journal.pone.0319806 (PMC11960971; doi:10.1371/journal.pone.0319806)
Supplement: S1 File — https://doi.org/10.6084/m9.figshare.25711734. (ZIP) [file pone.0319806.s001.zip › supporting information/paper file/Aalsma 2022.pdf]

Mind-Body Skills Groups for Adolescents with Depression in Primary Care: A Pilot Study

Matthew C. Aalsma, PhD [a], Lindsey D. Jones, BA [b], Julie K. Staples, PhD [c], Jennifer M. Garabrant, BSW[b], James S. Gordon, MD [c], Lynda Richtsmeier Cyr, PhD, LP [c], Eduardo Salgado, BS[b], Michelle P. Salyers, PhD [b]

[a]Department of Pediatrics – Adolescent Behavioral Health Research Program, Indiana University School of Medicine – Indianapolis, Indiana
[b] Department of Psychology, Indiana University-Purdue University Indianapolis – Indianapolis, Indiana
[c] The Center for Mind-Body Medicine –Washington, D.C.

Corresponding Author:

Matthew C. Aalsma, PhD
Professor, Department of Pediatrics
Director, Adolescent Behavioral Health Research Program
Indiana University School of Medicine
410 W. 10th Street, Suite 2025
Indianapolis, IN 46204
Office: 317-274-8812
Desk: 317-278-7797
Email: maalsma@iu.edu

Acknowledgments: The writing of this paper was supported by the Sandra Eskenazi Mental Health Center and the Herbert Simon Family Foundation (070241-00002B). A special thanks goes to all Eskenazi staff who led the study intervention and who assisted with recruitment, scheduling, and other study logistics.

Disclosures: Dr. Gordon is the founder and executive director of The Center for Mind-Body Medicine (CMBM), a non-profit that developed the intervention being discussed in this paper. Dr. Julie K. Staples is the research director at CMBM. Dr. Lynda Richtsmeier Cyr is the associate clinical director at CMBM. All other authors have no conflicts to report.

Funding: Supported by the Sandra Eskenazi Mental Health Center and the Herbert Simon Family Foundation (070241-00002B).

Keywords: Adolescents, Depression, Mind-Body Intervention, Primary Care

Clinical Trials Registry: ClinicalTrials.gov Identifier NCT03363750

Abbreviations: MBSG, Mind-Body Skills Group; CDI-2, CMBM, The Center for Mind-Body Medicine; Children's Depression Inventory-2; SIQ, Suicidal Ideation Questionnaire; (SIQ-JR) Suicide Ideation Questionnaire-Junior High School Version; MAAS-A, Mindful Attention Awareness Scale; SEQ-DA, Self-Efficacy for Depressed Adolescents; CRSQ-R, rumination subscale of the Children's Response Style Questionnaire; MBSR, mindfulness-based stress reduction

Abstract

Objective: To determine acceptability and preliminary effectiveness of Mind-Body Skills Groups (MBSGs) as a treatment for depressed adolescents in primary care.

Methods: A single arm clinical trial was conducted. A 10-week MBSG program was implemented in primary care. Participants completed self-report measures at baseline, post-intervention, and 3-months following the MBSGs. Measures included the Children's Depression Inventory-2, Suicidal Ideation Questionnaire, Mindful Attention Awareness Scale, Self-Efficacy for Depressed Adolescents, rumination subscale of the Children's Response Style Questionnaire, and a short acceptability questionnaire.

Results: Participants included 43 adolescents. The total depression scores significantly improved following the MBSG intervention and continued to improve significantly from post-treatment to follow-up. Mindfulness, self-efficacy, rumination, and suicidal ideation all had significant improvement following the intervention. Acceptability of the program was strong, and attendance was excellent.

Discussion: Preliminary evidence suggests that MBSGs are an acceptable treatment for primary care settings and lead to improved depression symptoms in adolescents.

INTRODUCTION

Depression affects nearly 14% of US adolescents (Hedden et al., n.d.) and is associated with impaired social functioning, (Carrellas, Biederman, & Uchida, 2017) poor academic performance, (Fletcher, 2009) and increased comorbid diagnoses such as anxiety disorder, substance abuse disorder, and suicidal behavior. (Avenevoli, Swendsen, He, Burstein, & Merikangas, 2015) The most common treatment for adolescents with depression includes psychiatric medication and cognitive-behavioral therapy (Thapar, Collishaw, Pine, & Thapar, 2012); however, adherence to psychiatric medications for adolescents is poor (Fontanella, Bridge, Marcus, & Campo, 2011) and linking adolescents to needed outpatient care is difficult for families facing multiple barriers to engaging in evidence-based depression treatment.

One approach to increase engagement in outpatient depression treatment is providing behavioral health services in primary care settings through collaborative behavioral healthcare. (Collins, Hewson, Munger, & Wade, 2010) Given that the majority of adolescents receive treatment for depression by a healthcare provider in their primary care clinic - rather than by an outpatient mental health specialist (Anderson, Chen, Perrin, & Van Cleave, 2015; Olfson, Blanco, Wang, Laje, & Correll, 2014) - providing behavioral health services in primary care settings can eliminate common barriers to service utilization such as additional costs, transportation issues, and perceived stigma related to mental health treatment. (Collins et al., 2010) Moreover, providing behavioral health treatment in primary care allows for improved communication between medical and behavioral health staff regarding co-occurring health problems and medication management. (Asarnow, Rozenman, Wiblin, & Zeltzer, 2015; Crowley & Kirschner, 2015) Integrating behavioral health services like depression screening, psychotherapy, and prescription medication is encouraged by professional agencies such as the

American Academy of Pediatrics (A. H. Cheung et al., 2007; Amy H. Cheung, Zuckerbrot, Jensen, Laraque, & Stein, 2018), and collaborative care models have shown to be associated with improved health outcomes; (Kodish, Richardson, & Schlesinger, 2019) however, research indicates that a substantial amount of youth do not utilize treatment services. (O'Connor et al., 2016) Thus, innovative programming for adolescent depression treatment in primary care is needed.

One approach encompasses mind-body modalities (MBM) to reduce depression with adolescents. Defined as a collection of healthcare practices aimed at bringing control over the mind, body, brain and behavior ("National Center for Complementary and Integrative Health. Complementary, Alternative, or Integrative Health: What's in a name?," 2018), MBM treatments teach a variety of mind-body techniques in one bundled approach; therefore, allowing individuals to experiment and choose techniques that work best for their individualized needs. Common practices of MBMs include techniques of yoga and meditation, as well as mindfulness and deep breathing. Mindfulness-based treatments is one element within MBMs that has gained popularity in recent years. For example, in a randomized controlled study with outpatient adolescents, 8 weeks of a mindfulness-based stress reduction (MBSR) in addition to usual treatment had a significant improvement in depression symptoms compared to usual treatment alone. (Biegel, Brown, Shapiro, & Schubert, 2009) In another study with adolescent girls at risk for diabetes with mild to moderate depression symptoms, those receiving a mindfulness intervention, which included breath awareness, body scanning, mindful eating, and mindful movement, had a significantly greater decrease in depression symptoms than those receiving cognitive behavioral therapy. (Shomaker et al., 2019) Thus, mindfulness interventions for adolescents struggling with depression are promising, but less is known about the effects of

MBM treatments - as a whole - on adolescents with depression; particularly in healthcare settings.

The Center for Mind-Body Medicine (CMBM) has developed a MBM program called Mind-Body Skills Groups (MBSGs). (J. S. Gordon et al., 2000) This program aims to restore psychological wellbeing by teaching diverse, evidence-based mind-body techniques that balance physiological functioning, stimulate self-awareness and emotional regulation, and enhance imagination and intuition. These techniques include a variety of concentrative MBM practices such as mindfulness, and active meditations including slow, deep breathing, mindful eating, and movement (i.e. shaking and dancing). Other techniques include biofeedback and autogenic training, guided imagery, and self-expression through written words and drawings. A key element of this program is the social support provided by the groups; social support has a strong association with the absence of depression among adolescents and children. (Gariépy, Honkaniemi, & Quesnel-Vallée, 2016) The MBSGs have been shown to be successful in treating posttraumatic stress symptoms in adolescents in Kosovo and Gaza (J. S. Gordon, Staples, Blyta, Bytyqi, & Wilson, 2008; Staples, Abdel Atti, & Gordon, 2011) as well as depression symptoms in war-traumatized children and adolescents in Gaza (Staples et al., 2011); however, less is known about this program's effectiveness and acceptability for treating adolescent depression within a primary care setting in the United States.

The purpose of the current research was to evaluate an open-trial pilot study aimed to: 1) establish acceptability of incorporating MBSGs into primary care settings, 2) garner preliminary evidence for effectiveness of the intervention in addressing adolescent depression symptoms and,

3) investigate the program's effects on related outcomes such as suicidal ideation, self-efficacy, rumination, and mindfulness.

METHODS

Clinic Setting

Adolescent participants were seen for care at one of 3 participating primary care clinics in an urban hospital system in central Indiana. Clinic staff consisted of primary care doctors, psychiatric consultants (e.g., psychiatrist or psychiatric nurse practitioner), registered nurses and onsite masters level clinicians who provide collaborative behavioral healthcare services.

Recruitment and Procedures

Eligible participants were first identified and approached by behavioral health clinicians who provided information regarding the study. If a participant and their parent (or guardian) expressed interest, a research assistant later contacted the parent by telephone to answer questions and schedule a screening appointment. After obtaining parental informed consent and adolescent assent, eligibility was assessed, and baseline measures were collected. Eligibility criteria required that the adolescent (1) was a primary care patient of the participating hospital system, (2) screened positive for depression through a structured diagnostic interview, (Duncan et al., 2018) (3) was 13 to 17 years old, (4) spoke English, and (5) was willing to attend the MBSGs for 10 weeks. Adolescents were excluded if they (1) had a history of bipolar disorder or psychosis, (2) were at an acute and immediate risk for suicide at the time of screening, (3) lacked capacity to provide assent, or (4) had previously participated in a MBSG.

Recruitment was conducted in waves over the study period, with up to 12 participants recruited per group. Once a MBSG ended, a new wave of participants were recruited. The MBSGs were held once per week for 10 weeks and sessions lasted 1.5 hours. Group sessions took place during after school hours (e.g., 3 or 4 PM) and were delivered in conference rooms at the primary care clinics. Group sessions were facilitated by 2 CMBM-trained behavioral health clinicians. The behavioral health clinicians were provided with a manual for delivery instructions on the intervention and supervised weekly by CMBM clinical faculty to ensure fidelity to the model. During the 10-week series, participants were taught a variety of tools and techniques to foster self-awareness and emotional regulation (see Table 1). Participants were asked to complete surveys after the MBSG was completed and again approximately 3 months later. Adolescents were compensated travel costs for attending each MBSG session ($10), completing the screen/baseline procedures ($60), and completing the 2 post-intervention visits ($40 each). Additionally, participants could earn chances to draw rewards from a “fishbowl” for participating in the treatment (attending group meetings and practicing mind-body skills as homework). Prizes included $5 gift cards, $10 gift cards, and one $50 gift card. This project was approved by Indiana University’s Institutional Review Board and the participating hospital system’s research committee.

Measures

Participant demographics were collected during the screening appointment (see Table 2), and attendance was recorded at each session. In addition, all measures below were administered at baseline, post-intervention, and 3 months later unless otherwise noted.

Acceptability

Acceptability was assessed at both time points after the intervention using items developed by the research team. Using a 5-point rating scale (1 = Do Not Agree; 5 = Completely Agree) participants responded to the following questions: (1) the MBSGs were useful, (2) I had to force myself to attend the MBSGs, (3) I apply what I learned in my everyday life, (4) the MBSGs were enjoyable, (5) I would recommend the MBSGs to others, (6) I am certain I will benefit from the skills I learn in the long run, and (7) my group facilitators were understanding. Acceptability was calculated by computing an average score based on each time point (baseline alpha=0.90; 3-month alpha=0.85).

Depression

The main outcome of depression was assessed with the Children's Depression Inventory (CDI-2) (Kovacs, 2003) which consists of 28 self-report items rated from 0 (no depressive symptom) to 2 (significant depressive symptom). The CDI-2 instrument has two primary indices that measure symptoms of *emotional problems* and *functional problems*, and 4 subscale items (2 for each index) that measure symptoms of *negative mood/physical symptoms, negative self-esteem, interpersonal problems, and ineffectiveness*. Items are summed into raw scores and then transformed into T-scores, which are used to determine depression classification.

Suicidal Ideation

Suicidal ideation was measured using the Suicidal Ideation Questionnaire (SIQ; grades 10th -12th) and junior high school version (SIQ-JR; grades 7th - 9th). (Reynolds, William M. Mazza, 1988) Both versions of the SIQ assess the frequency in which adolescents cognitively

engaged in thoughts of suicide or death in the past month. The SIQ consists of 30 items and the SIQ-JR consists of 15 items. All items are rated on a 7-point Likert scale (0 = I never had this thought; 6 = Almost every day). A cutoff score of ≥ 41 on the SIQ, or ≥ 31 on the SIQ-JR, indicates a person is at acute risk for suicide. Both SIQ measures also contain critical content items which can postulate severe risk for suicide (SIQ = 8 critical items; SIQ-JR = 6 critical items). If a participant met the cutoff score, and/or scored high (i.e., 5-6) on at least 3 of the critical content items, they were considered at acute risk of suicide and received a clinical assessment by a behavioral health clinician.

Mindfulness

Adolescent mindfulness was measured using the Mindful Attention Awareness Scale – Adolescents (MAAS-A), (Brown, West, Loverich, & Biegel, 2011) a 14-item assessment designed to measure enhanced attention and awareness of present reality. The MAAS-A uses 6-point Likert scale where higher scores reflect greater mindfulness.

Self-Efficacy

The Self-Efficacy Questionnaire for Depressed Adolescents (SEQ-DA) is a 12-item self-report measure of effectiveness in coping with depression. (M. S. Gordon, Tonge, & Melvin, 2012) Adapted specifically for adolescent depression, the SEQ-DA includes items that measure effectiveness in coping with mood and irritability, sleeping patterns, eating behaviors, concentration, social interactions, negative thoughts, and thoughts of self-harm. The SEQ-DA is evaluated on a 5-point Likert scale where higher total scores indicate greater self-efficacy.

Rumination

Rumination was assessed using the Children's Response Style Questionnaire - Rumination (CRSQ-R), a self-report 13-item measure that assesses youth response styles to depressed mood. (Abela, Aydin, & Auerbach, 2007) Participants are asked to rate on a 4-point Likert scale (0 = almost never to 3 = almost always) the extent to which they adopt a certain coping strategy when they feel sad. Total scores are computed by summing the overall score of the 13 items. Scores can range from 0-39, with higher total scores indicating higher levels of rumination.

Statistical Analysis

All outcome analyses only included data from participants who attended at least one MBSG. Missing data at the question level were imputed with SYSTAT (v.12.02) using the Expectation/Maximization (EM) algorithm except for the SIQ and SIQ-JR*,* which used the author recommendations for prorated scoring. (Reynolds, William M. Mazza, 1988) Data analysis was performed using SPSS version 26 (IBM, SPSS). To test change over time, the MIXED procedure in SPSS was used to run a linear mixed model to account for clustering of the participants within each MBSG and repeated measures over time for individuals. Although this 3-level model accounted for all the possible levels of variance in the design, for the SIQ, SIQ-JR, CRSQ-R and the ineffectiveness and negative self-esteem subscales of the CDI-2, there was not enough variance at the clustered MGSG level to give a stable estimate for the variance parameters. For these outcomes, a marginal model was used to account for the repeated measures of individuals over time. An unstructured covariance matrix of the residuals was used in this model. Time was the fixed factor in all models. In addition, the following aspects of participants'

status were included as fixed factors as well as their interactions with time: sex, age, ethnicity, and MBSG attendance. If any of these fixed factors or their interactions were significant, they were included in the final model. Pairwise comparisons of differences between outcomes at each time point were performed using a Sidak correction to control for Type I errors.

RESULTS

Demographics

Overall, our study delivered 5 independent MBSGs in the participating primary care clinics. A total of 49 adolescents were recruited for the study; of those, 2 participants were ineligible due to depression screen failure. Of the remaining 47 participants, 4 adolescents' data were excluded because they did not attend any MBSG sessions. The final sample included 43 adolescents who were primarily female (79%), Hispanic (67%) and in Junior High (58%; see Table 2).

Acceptability of Intervention

Acceptability scores were positive throughout the study (post-intervention = 4.24; 3-month follow-up visit = 4.26). Attendance for the MBSGs was excellent: only 14% (n = 6) of participants attended less than half of the sessions and 86% (n = 37) of participants attended 6 or more sessions. In total, 25% (n = 11) had perfect attendance (all 10 sessions).

Depression Primary Outcome

The estimated marginal mean scores and the overall level of significance of the changes over time are shown in Table 3. There was significant improvement over time in the total CDI-2

score as well as for functional and emotional problems and their subscales of negative mood/physical symptoms, negative self-esteem, ineffectiveness, and interpersonal problems. Pairwise analysis showed significant improvement at the post-intervention time point for the total CDI-2 score and all of the scales/subscales (Table 3). In addition, for the total CDI-2 score and ineffectiveness subscale, the follow-up time point was significantly lower than the post-intervention time point (total CDI-2: -2.1 [95% CI, -402 to -0.02]; $p = .048$) (ineffectiveness: -1.1 [95% CI, -2.1 to -0.01]; $p = .048$) showing continued improvement at follow-up. For the other scales/subscales there was no statistically significant difference between the post intervention and follow-up time points indicating that the improvements were maintained at follow-up. While the overall improvement was the same for males and females, males had significantly higher ineffectiveness score than females throughout the study (1.8 [95% CI, 0.29 to 3.5]; $p = .022$ across all points). For emotional problems and its negative self-esteem subscale, scores from Hispanic participants were higher than non-Hispanic participants at all time points, indicating more severe emotional problems (2.7 [95% CI, 0.29 to 5.3]; $p = .030$ across all time points) and negative self-esteem (2.4 [95% CI, 0.96 to 3.8]; $p = .002$ across all time points). Depression classifications based on T-Scores are shown in Table 4.

Secondary Outcomes

In addition to improved depression, there was significant improvement post-intervention in secondary outcomes of mindfulness (MAAS-A), self-efficacy (SEQ-DA), rumination (CRSQ-R) and suicidal ideation (SIQ and SIQ-JR; see Table 3). There was no statistically significant difference between the post-intervention and follow-up time points indicating that the improvement was maintained at follow-up.

DISCUSSION

Adolescent depression is frequently identified and diagnosed by primary care providers, yet engagement in mental health treatment is very low in primary care (O'Connor et al., 2016) and is often limited to antidepressant medication or therapy. (Merikangas et al., 2010) Given that the prevalence of adolescent depression is increasingly high, (Mojtabai, Olfson, & Han, 2016; Twenge, Joiner, Rogers, & Martin, 2018) innovative treatment models that are effective and keep youth engaged are needed in primary care. Results from our study suggest that MBSGs could be feasible for reducing depression in adolescents seeking mental health treatment in primary care. In addition, participants reported significant improvements in mindfulness, self-efficacy, rumination and suicidal ideation following the MBSG. These results were maintained at follow-up. Below we discuss the findings of this work and implications for depression treatment for adolescents.

Acceptability of MBSGs in Primary Care

Acceptability of MBSGs was strong, with the majority of adolescents attending more than half of the sessions and <10% not attending any. Only 4 participants did not attend any sessions. Previous studies have shown that integrating behavioral health services into primary care is feasible and effective for treating depressed youth as compared to providing usual care (Kodish et al., 2019; Richardson et al., 2014); however, collaborative care models primarily focus on providing traditional forms of treatment (e.g., psychotherapy or psychiatric medication) rather than holistic approaches. Thus, the current study extends the current collaborative care model by the integration of a holistic treatment approach in a collaborative care setting.

Depression Outcomes

The primary outcome of interest in the current project was symptoms of depression. Following the intervention, overall depression scores (as well as subscales) decreased significantly. Decreased scores were well maintained at the 3-month follow-up visit, and overall depression as well as one subscale showed increased improvement. Given high rates of adolescent depression and suicide (Cha et al., 2018), the finding that suicidal ideation also improved significantly over time is important. Further research with a control group is needed, but the current findings suggest that MBSGs may be an additional intervention to consider for adolescents coping with depression. In addition, given higher subscale scores for males (ineffectiveness) and for Hispanics (emotional problems and negative self-esteem), future studies will need increased variation in gender and ethnicity to study potential differential effectiveness for different populations. However, the success of this intervention for Hispanic youth who have increased rates of depression diagnoses and are less likely to receive mental health treatment as compared to Non-Hispanic youth (Control & Prevention, 2017; Merikangas et al., 2011) is encouraging and important.

Secondary Outcomes

In addition to depression and suicidal thoughts, we included other secondary outcomes to identify possible mechanisms of change. Although our sample is too small to test mediational mechanisms, participants in our study showed significant improvements in mindfulness and self-efficacy, as well as diminished rumination. Other studies suggest these could be important mediators. For example, enhanced awareness of reality (i.e., mindfulness) (Kearns et al., 2016), greater self-efficacy (M. S. Gordon, Tonge, & Melvin, 2011) and decreased rumination

(Michalak, Hölz, & Teismann, 2011) have been shown to be predictive of reductions in depression relapse. Youth in our study demonstrated significant improvement in their depressive symptoms and a decrease in ruminating thoughts and reported enhanced mindfulness and self-efficacy behaviors. Thus, findings suggest that MBSGs may contribute in a variety of ways to decreasing depression in youth and can be effectively integrated into primary care.

Limitations

Our primary study limitation is the lack of a control group. While promising, the effects of the intervention need to be tested in a rigorous design. In addition, participants were primarily Hispanic/Latinx (67%), female (79%) and were not representative of all adolescents who seek mental health treatment in primary care. Finally, although the intervention was acceptable from the participants' point of view, we did not assess acceptability from clinic providers or at the healthcare system level. This particular organization has invested heavily in training behavioral health clinicians to use the MBSGs, but future studies would be needed to ensure feasibility of implementation in other settings as well.

CONCLUSION

Adolescence is a period in which health behaviors and states of mind, including depression, may shape future health outcomes. Depression is often first recognized and treated by primary care providers, yet youth engagement in mental health treatment is poor. (Abuse, 2018; Anderson et al., 2015) Early recognition and intervention in adolescent depression can help alleviate health problems in adulthood. (Naicker, Galambos, Zeng, Senthilselvan, & Colman, 2013; Rohde, Lewinsohn, Klein, Seeley, & Gau, 2013) It is important, therefore, to

provide effective early interventions in a setting that is easily accessible, and in a way that is acceptable to youth. MBSGs in primary care fulfill these criteria and appear to be a promising treatment option for youth with depression. Future randomized trials will be important to assess the value of including this model of depression treatment in primary care settings.

HIGHLIGHTS

- Adolescent depression is a serious and growing concern that can lead to co-morbid diagnoses and negative health outcomes.
- Primary care settings are an ideal home for receiving depression treatment; however, treatments services are often limited in this setting.
- Preliminary data suggests that a new intervention, Mind-Body Skills Groups, could be an effective and feasible treatment for depressed adolescents seeking services in primary care.
- Additional research is needed to understand the full effectiveness, feasibility and acceptability of Mind-Body Skills Groups as a depression treatment for adolescents in primary care.

REFERENCES

Abela, J. R. Z., Aydin, C. M., & Auerbach, R. P. (2007). Responses to Depression in Children: Reconceptualizing the Relation Among Response Styles. *Journal of Abnormal Child Psychology*, *35*(6), 913–927. https://doi.org/10.1007/s10802-007-9143-2

Abuse, S. (2018). Mental Health Services Administration. Key substance use and mental health indicators in the United States: Results from the 2017 National Survey on Drug Use and Health. *Center for Behavioral Health Statistics and Quality.*, *HHS Public*.

Anderson, L. E., Chen, M. L., Perrin, J. M., & Van Cleave, J. (2015). Outpatient Visits and Medication Prescribing for US Children With Mental Health Conditions. *PEDIATRICS*, *136*(5), e1178–e1185. https://doi.org/10.1542/peds.2015-0807

Asarnow, J. R., Rozenman, M., Wiblin, J., & Zeltzer, L. (2015). Integrated Medical-Behavioral Care Compared With Usual Primary Care for Child and Adolescent Behavioral Health. *JAMA Pediatrics*, *169*(10), 929. https://doi.org/10.1001/jamapediatrics.2015.1141

Avenevoli, S., Swendsen, J., He, J.-P., Burstein, M., & Merikangas, K. R. (2015). Major Depression in the National Comorbidity Survey–Adolescent Supplement: Prevalence, Correlates, and Treatment. *Journal of the American Academy of Child & Adolescent Psychiatry*, *54*(1), 37-44.e2. https://doi.org/10.1016/j.jaac.2014.10.010

Biegel, G. M., Brown, K. W., Shapiro, S. L., & Schubert, C. M. (2009). Mindfulness-based stress reduction for the treatment of adolescent psychiatric outpatients: A randomized clinical trial. *Journal of Consulting and Clinical Psychology*, *77*(5), 855.

Brown, K. W., West, A. M., Loverich, T. M., & Biegel, G. M. (2011). Assessing Adolescent Mindfulness: Validation of an Adapted Mindful Attention Awareness Scale in Adolescent Normative and Psychiatric Populations. *Psychological Assessment*, *23*(4), 1023–1033. https://doi.org/10.1037/a0021338

Carrellas, N. W., Biederman, J., & Uchida, M. (2017). How prevalent and morbid are subthreshold manifestations of major depression in adolescents? A literature review. *Journal of Affective Disorders*, *210*, 166–173. Retrieved from http://www.sciencedirect.com/science/article/pii/S0165032716316238

Cha, C. B., Franz, P. J., M. Guzmán, E., Glenn, C. R., Kleiman, E. M., & Nock, M. K. (2018). Annual Research Review: Suicide among youth - epidemiology, (potential) etiology, and treatment. *Journal of Child Psychology and Psychiatry*, *59*(4), 460–482. https://doi.org/10.1111/jcpp.12831

Cheung, A. H., Zuckerbrot, R. A., Jensen, P. S., Ghalib, K., Laraque, D., & Stein, R. E. K. (2007). Guidelines for Adolescent Depression in Primary Care (GLAD-PC): II. Treatment and Ongoing Management. *PEDIATRICS*, *120*(5), e1313–e1326. https://doi.org/10.1542/peds.2006-1395

Cheung, Amy H., Zuckerbrot, R. A., Jensen, P. S., Laraque, D., & Stein, R. E. K. (2018). Guidelines for Adolescent Depression in Primary Care (GLAD-PC): Part II. Treatment and Ongoing Management. *Pediatrics*, *141*(3), e20174082. https://doi.org/10.1542/peds.2017-4082

Collins, C., Hewson, D. L., Munger, R., & Wade, T. (2010). Evolving Models of Behavioral Health Integration in Primary Care. *New York: Millbank Memorial Fund*, *504*.

Control, C. for D., & Prevention. (2017). YRBSS results. Retrieved from outh risk behavior

surveillance survey (YRBSS) results website: https://www.cdc.gov/healthyyouth/data/yrbs/results.htm

Crowley, R. A., & Kirschner, N. (2015). The Integration of Care for Mental Health, Substance Abuse, and Other Behavioral Health Conditions into Primary Care: Executive Summary of an American College of Physicians Position Paper. *Annals of Internal Medicine*, *163*(4), 298. https://doi.org/10.7326/M15-0510

Duncan, L., Georgiades, K., Wang, L., Van Lieshout, R. J., MacMillan, H. L., Ferro, M. A., … Boyle, M. H. (2018). Psychometric evaluation of the Mini International Neuropsychiatric Interview for Children and Adolescents (MINI-KID). *Psychological Assessment*, *30*(7), 916–928. https://doi.org/10.1037/pas0000541

Fletcher, J. M. (2009). Adolescent depression and educational attainment: results using sibling fixed effects. *Health Economics*, *19*(7), 855–871. https://doi.org/10.1002/hec.1526

Fontanella, C. A., Bridge, J. A., Marcus, S. C., & Campo, J. V. (2011). Factors Associated with Antidepressant Adherence for Medicaid-Enrolled Children and Adolescents. *Annals of Pharmacotherapy*, *45*(7–8), 898–909. https://doi.org/10.1345/aph.1Q020

Gariépy, G., Honkaniemi, H., & Quesnel-Vallée, A. (2016). Social support and protection from depression: systematic review of current findings in Western countries. *British Journal of Psychiatry*, *209*(4), 284–293. https://doi.org/10.1192/bjp.bp.115.169094

Gordon, J. S., Fisher, T., Lord, S., Buckley, R., Kimmel, J., & Shinal, A. (2000). *The Advanced Traning Manual*. Washington, DC: The Center for Mind-Body Medicine.

Gordon, J. S., Staples, J. K., Blyta, A., Bytyqi, M., & Wilson, A. T. (2008). Treatment of Posttraumatic Stress Disorder in Postwar Kosovar Adolescents Using Mind-Body Skills Groups. *The Journal of Clinical Psychiatry*, *69*(9), 1469–1476. https://doi.org/10.4088/JCP.v69n0915

Gordon, M. S., Tonge, B., & Melvin, G. A. (2011). Outcome of Adolescent Depression: 6 Months After Treatment. *Australian & New Zealand Journal of Psychiatry*, *45*(3), 232–239. https://doi.org/10.3109/00048674.2010.538838

Gordon, M. S., Tonge, B., & Melvin, G. A. (2012). The Self-Efficacy Questionnaire for Depressed Adolescents: A measure to predict the course of depression in depressed youth. *Australian and New Zealand Journal of Psychiatry*, *46*(1), 47–54. https://doi.org/10.1177/0004867411428390

Hedden, S. L., Kennet, J., Lipari, R., Medley, G., Tice, P., P Copello, E. A., & Kroutil, L. A. (n.d.). *Key Substance Use and Mental Health Indicators in the United States: Results from the 2015 National Survey on Drug Use and Health*. Retrieved from http://www.samhsa.gov/data/

Kearns, N. P., Shawyer, F., Brooker, J. E., Graham, A. L., Enticott, J. C., Martin, P. R., & Meadows, G. N. (2016). Does rumination mediate the relationship between mindfulness and depressive relapse? *Psychology and Psychotherapy: Theory, Research and Practice*, *89*(1), 33–49. https://doi.org/10.1111/papt.12064

Kodish, I., Richardson, L., & Schlesinger, A. (2019). Collaborative and Integrated Care for Adolescent Depression. *Child and Adolescent Psychiatric Clinics of North America*, *28*(3), 315–325. https://doi.org/10.1016/j.chc.2019.02.003

Kovacs, M. (2003). *Children's Depression Inventory Technical Manual Update*. Toronto, Ontario, Canada: Multi-Health Systems.

Merikangas, K. R., He, J., Burstein, M., Swendsen, J., Avenevoli, S., Case, B., … Olfson, M. (2011). Service Utilization for Lifetime Mental Disorders in U.S. Adolescents: Results of

the National Comorbidity Survey–Adolescent Supplement (NCS-A). *Journal of the American Academy of Child & Adolescent Psychiatry*, *50*(1), 32–45. https://doi.org/10.1016/j.jaac.2010.10.006

Merikangas, K. R., He, J. P., Burstein, M., Swanson, S. A., Avenevoli, S., Cui, L., … Swendsen, J. (2010). Lifetime prevalence of mental disorders in U.S. adolescents: Results from the national comorbidity survey replication-adolescent supplement (NCS-A). *Journal of the American Academy of Child and Adolescent Psychiatry*, *49*(10), 980–989. https://doi.org/10.1016/j.jaac.2010.05.017

Michalak, J., Hölz, A., & Teismann, T. (2011). Rumination as a predictor of relapse in mindfulness-based cognitive therapy for depression. *Psychology and Psychotherapy: Theory, Research and Practice*, *84*(2), 230–236. https://doi.org/10.1348/147608310X520166

Mojtabai, R., Olfson, M., & Han, B. (2016). National trends in the prevalence and treatment of depression in adolescents and young adults. *Pediatrics*, *138*(6), e20161878. https://doi.org/10.1542/peds.2016-1878

Naicker, K., Galambos, N. L., Zeng, Y., Senthilselvan, A., & Colman, I. (2013). Social, demographic, and health outcomes in the 10 years following adolescent depression. *Journal of Adolescent Health*, *52*(5), 533–538. Retrieved from https://www.sciencedirect.com/science/article/pii/S1054139X13000451?via%3Dihub

National Center for Complementary and Integrative Health. Complementary, Alternative, or Integrative Health: What's in a name? (2018). Retrieved April 27, 2020, from National Institute of Health website: https://nccih.nih.gov/health/integrative-health

O'Connor, B. C., Lewandowski, R. E., Rodriguez, S., Tinoco, A., Gardner, W., Hoagwood, K., & Scholle, S. H. (2016). Usual Care for Adolescent Depression From Symptom Identification Through Treatment Initiation. *JAMA Pediatrics*, *170*(4), 373. https://doi.org/10.1001/jamapediatrics.2015.4158

Olfson, M., Blanco, C., Wang, S., Laje, G., & Correll, C. U. (2014). National Trends in the Mental Health Care of Children, Adolescents, and Adults by Office-Based Physicians. *JAMA Psychiatry*, *71*(1), 81. https://doi.org/10.1001/jamapsychiatry.2013.3074

Reynolds, William M. Mazza, J. J. (1988). *Suicidal Ideation Questionnaire Professional Manuel*. Florida: Psychological Assessment Resources.

Richardson, L. P., Ludman, E., McCauley, E., Lindenbaum, J., Larison, C., Zhou, C., … Katon, W. (2014). Collaborative Care for Adolescents With Depression in Primary Care. *JAMA*, *312*(8), 809. https://doi.org/10.1001/jama.2014.9259

Rohde, P., Lewinsohn, P. M., Klein, D. N., Seeley, J. R., & Gau, J. M. (2013). Key characteristics of major depressive disorder occurring in childhood, adolescence, emerging adulthood, and adulthood. *Clinical Psychological Science*, *1*(1), 41–53.

Shomaker, L. B., Pivarunas, B., Annameier, S. K., Gulley, L., Quaglia, J., Brown, K. W., … Bell, C. (2019). One-Year Follow-Up of a Randomized Controlled Trial Piloting a Mindfulness-Based Group Intervention for Adolescent Insulin Resistance. *Frontiers in Psychology*, *10*. https://doi.org/10.3389/fpsyg.2019.01040

Staples, J. K., Abdel Atti, J. A., & Gordon, J. S. (2011). Mind-Body Skills Groups for Posttraumatic Stress Disorder and Depression Symptoms in Palestinian Children and Adolescents in Gaza. *International Journal of Stress Management*, *18*(3), 246–262. https://doi.org/10.1037/a0024015

Thapar, A., Collishaw, S., Pine, D. S., & Thapar, A. K. (2012). Depression in adolescence. *The*

Lancet, *379*(9820), 1056–1067.

Twenge, J. M., Joiner, T. E., Rogers, M. L., & Martin, G. N. (2018). Increases in Depressive Symptoms, Suicide-Related Outcomes, and Suicide Rates Among U.S. Adolescents After 2010 and Links to Increased New Media Screen Time. *Clinical Psychological Science*, *6*(1), 3–17. https://doi.org/10.1177/2167702617723376

Table 1. Center for Mind-Body Medicine Model and Techniques *

Each session is structured with: (1) opening meditation (soft belly breathing); (2) check-in; (3) didactic: explanation of a mind-body skill technique (one for each session); (4) experiential exercise: experimentation using the mind-body skill technique; (5) sharing of personal experience using the mind-body skill technique; and (5)closing meditation (soft belling breathing).

Session I: Overview of Mind-Body Medicine and Opening Drawings

Draw yourself – as you see yourself today.
Draw yourself – with your biggest problem, challenge or issue.
Draw yourself – with your biggest problem, challenge or issue solved.

Session II: Autogenic Training and Biofeedback

Discuss the biological underpinnings of "mind-body medicine" and use biofeedback skin thermometers to demonstrate we can control and affect out physiology (stress response).

Session III: Meditation

Discuss the three different types of meditation and how they are useful. Experience an active meditation (shaking and dancing or walking meditation).

Session IV: Guided Imagery

Use "safe place" and "wise-guide" guided imagery to invoke relaxation and intuition.

Session V: Dialogue with a Symptom

Acknowledge the power of writing as a tool for accessing the unconscious and solving problems. Practice writing as an exercise for self-expression and insight.

Session VI: Genograms

Create genograms to explore family patterns, identify sources of support, and increase our self-awareness.

Session VII: Genograms

Continue sharing genograms from session VII.

Session VIII: Relationship with Food and Mindful Eating

Introduce the concept of "food as medicine" and experience mindful eating and share its benefits.

Session IX: Spirituality

Practice forgiveness meditation or loving kindness meditation.

Session X: Closing Drawings and Closing Ritual

Draw yourself – as you are now.
Draw yourself – as you'd like to be (personally, professionally, emotionally, etc.).
Draw – how you are going to get there. What is the road map?

**All techniques from the model were covered during the 10-week series; however, the order and structure of some sessions may have varied depending on time, level of participation, group preference.*

Table 2. Participant Characteristics (N = 43)

Characteristics		Frequency	Percent
**Gender**			
	Female	34	79.1%
	Male	9	20.9 %
**Race**			
	White or Caucasian	16	37.2%
	Black or African American	8	18.6%
	Multiracial	2	4.7%
	Refused	17	39.5%
**Ethnicity**			
	Hispanic or Latinx	29	67.4%
	Not Hispanic or Latinx	13	30.2%
	Participant unsure	1	2.3%
**Education Level**			
	8th -9th grade	25	58.2%
	10th – 12th grade	18	41.8%
**Age (M = 15.2; SD = 1.3)**			

Table 3. Effect of the MBSGs over time

	Mean Score[a] (95% Confidence Interval)				
**Outcome Measure**	**Baseline (n = 43)[b]**	**Post Treatment (n=39)[c]**	**Follow-up (n=39)[d]**	**Pairwise comparisons over time[e]**	***p* value[f]**
CDI-2 Total Score	27.5 (23.3-31.8)	17.5 (13.2-21.8)	15.4 (11.2-19.5)	T1 > T2, T3; T2 >T3	< .001
Emotional Problems	13.7 (11.9- 15.5)	8.3 (6.6-10.0)	7.4 (5.6-9.1)	T1 > T2, T3	<.001
Negative Mood/Physical Symptoms	8.9 (7.4-10.5)	5.5 (4.0-7.1)	5.2 (3.7-6.7)	T1 > T2, T3	< .001
Negative Self-Esteem	4.9 (4.1-5.8)	3.1 (2.2-3.9)	2.4 (1.7-3.1)	T1 > T2, T3	< .001
Functional Problems	12.8 (11.1-14.4)	8.5 (6.7-10.2)	7.2 (5.5-8.9)	T1 > T2, T3	< .001
Ineffectiveness	9.6 (8.7-10.6)	6.5 (5.4-7.6)	5.4 (4.3-6.5)	T1 > T2, T3; T2 > T3	< .001
Interpersonal Problems	3.7 (2.9-4.6)	2.3 (1.4-3.2)	1.9(1.0-2.8)	T1 > T2, T3	< .001
SIQ	49.8 (31.1-68.4)	28.2 (12.2-44.2)	27.2 (14.2-40.2)	T1 > T2, T3	.007
SIQ-JR	32.1 (24.0-40.2)	20.2 (11.9-28.4)	19.3 (10.9-27.6)	T1 > T2, T3	.006
Mindfulness	45.1 (39.3-50.8)	52.2 (46.4-58.1)	57.3 (51.7-63.0)	T1 < T2, T3	.001
Self-Efficacy	33.2 (28.2-38.2)	41.8 (36.8-46.7)	42.8 (37.9-47.7)	T1 < T2, T3	< .001
Rumination	39.4 (37.1-41.6)	30.9 (28.0-33.9)	31.5 (28.7-34.3)	T1 > T2, T3	< .001

Abbreviations: T1= Baseline, T2= Post Treatment, T3 = Follow-up
[a]Mean scores are Estimated Marginal Means from the repeated measures linear mixed models.
[b] for Mindfulness and Rumination, n = 42, for SIQ n = 18, for SIQ-JR n = 25
[c] for Ineffectiveness and Functional Problems and Total CDI-2, n = 38, for SIQ n = 15, for SIQ-JR n = 24
[d] for SIQ n = 16 , for SIQ-JR n = 23
[e] $p < .001$ for all except CID-2 Total and Ineffectiveness T2 >T3, SIQ and SIQ-JR T1>T3, SIQ-JR T1>T2, and Mindfulness T1<T2 where $p < .05$; Mindfulness T1<T3 and SIQ T1>T2 where $p < .01$;
[f] p value is for the overall change across time.

Table 4. Depression Classification Changes based on CDI-2 T-Scores

	**Baseline**		**Post Treatment**		**Follow-up**	
	**T-score Mean (SD)**	**Depression Classification[a]**	**T-Score Mean (SD)**	**Depression Classification**	**T-Score Mean (SD)**	**Depression Classification**
Male	84.7 (6.4)	Very Elevated	75.8 (9.8)	Very Elevated	69.4 (12.4)	Elevated
Female	73.3 (9.0)	Very Elevated	61.9 (11.5)	High Average	58.3 (10.3)	Average

[a]Depression classification categories are as follows: Very Elevated (≥ 70), Elevated (65-69), High Average (60-64), Average or Lower (≤59).
