# Supplementary material for: Does physical activity-based intervention decrease repetitive negative thinking? A systematic review
Source: PLoS One. 2025 Apr 1;20(4):e0319806. doi: 10.1371/journal.pone.0319806 (PMC11960971; doi:10.1371/journal.pone.0319806)
Supplement: S1 File — https://doi.org/10.6084/m9.figshare.25711734. (ZIP) [file pone.0319806.s001.zip › supporting information/paper file/Gordon 2021.pdf]

Journal of Affective Disorders 281 (2021) 153–159

Contents lists available at ScienceDirect

Journal of Affective Disorders

journal homepage: www.elsevier.com/locate/jad

Research paper

Resistance exercise training among young adults with analogue generalized anxiety disorder

Brett R. Gordon [a,b,c,*], Cillian P. McDowell [a,b,c,d], Mark Lyons [a], Matthew P. Herring [a,b,e]

[a] Department of Physical Education and Sport Sciences, University of Limerick, Limerick, Ireland
[b] Physical Activity for Health Research Cluster, Health Research Institute, University of Limerick, Limerick, Ireland
[c] Public Health Sciences, Penn State College of Medicine, Pennsylvania, United States of America
[d] The Irish Longitudinal Study on Ageing, Trinity College Dublin, Dublin, Ireland
[e] School of Medicine, Trinity College Dublin, Dublin, Ireland

A R T I C L E I N F O

Keywords:
Worry
Strength training
Anxious
Muscle Strengthening Activity

A B S T R A C T

Introduction: The objective of this randomized controlled trial (RCT) was to quantify the effects of eight weeks of World Health Organization and American College of Sports Medicine guidelines-based resistance exercise training (RET) among participants meeting criteria for subclinical, or analogue-GAD (AGAD)
Methods: Forty-four participants (mean age (y): 25.4.6±4.9.2) were randomized to either an eight-week, fully-supervised, one-on-one RET intervention or wait-list control. AGAD status was determined using validated cut-scores for both the Psychiatric Diagnostic Screening Questionnaire-GAD subscale (≥6) and Penn State Worry Questionnaire (≥45). Remission, based on change in AGAD status, was assessed post-intervention, and quantified with number needed to treat (NNT). Primary analyses focused on participants missing outcome data at ≤1 time point (RET: n=12, Wait-list: n=15). RM-ANCOVA examined differences between RET and wait-list across time. Simple effects analysis decomposed significant interactions. Hedges' *d* quantified magnitude of differences in change between conditions over time.
Results: Attendance was 81% and compliance to the RET was 77%. Participants significantly increased strength (all d≥1.24, $p \leq 0.006$) with no adverse events. RET improved AGAD status (NNT=3, 95%CI: 2 to 7). Significant group X time interactions were found for worry ($F_{(3,66)} = 3.12$, $p \leq 0.043$; d=0.93, 95%CI: 0.13 to 1.73) and anxiety symptoms ($F_{(3,57.84)}$=2.91, ε=0.88, $p \leq 0.045$; d=0.71, 95%CI: -0.08 to 1.49). RET significantly reduced worry (mean difference=-6.49, $p \leq 0.045$) and anxiety symptoms (mean difference=-10.50, $p \leq 0.001$).
Limitations: Limitations include a small sample size, and lack of attention-matched control condition.
Conclusion: RET significantly improved AGAD severity, and elicited large, clinically meaningful improvements in worry and anxiety symptoms among young adults with AGAD.

Introduction

Anxiety disorders are prevalent (Bandelow & Michaelis, 2015), the sixth leading cause of disability in terms of years of life lived with disability (Baxter et al., 2014), and associated with poor health (Kessler et al., 2009). Anxiety disorders are economically burdensome (Andlin-Sobocki & Wittchen, 2005) and poorly treated; 60% of patients with anxiety disorders do not positively respond to front-line interventions (i.e., cognitive behavioural therapy or pharmacotherapy) and many maintain residual symptoms following treatment (Bystritsky, 2006). Alternative or augmented treatments, such as physical activity and exercise, protect against anxiety symptoms and disorders (McDowell et al., 2019).

Physical activity is any bodily movement produced by skeletal muscles that results in energy expenditure; exercise is a subset of physical activity that is planned, structured, and repetitive, for the purpose of enhancing or maintaining one or more components of fitness (Caspersen et al., 1985). Exercise has well established effects on anxiety among otherwise healthy adults (Conn, 2010), chronically-ill adults (Herring et al., 2010), and anxiety disorder patients (Herring et al., 2012). However, people with Generalized Anxiety Disorder (GAD) are less likely to meet the minimum recommended levels of physical activity than those without GAD (McDowell et al., 2018). Understudied compared to aerobic exercise, recent meta-analytic evidence showed

* Corresponding author at: 500 University Drive, Public Health Sciences, Penn State College of Medicine, Hershey, Pennsylvania, United States of America.
E-mail address: brg5334@psu.edu (B.R. Gordon).

https://doi.org/10.1016/j.jad.2020.12.020
Received 4 June 2020; Received in revised form 18 November 2020; Accepted 5 December 2020
Available online 8 December 2020
0165-0327/© 2021 The Authors. Published by Elsevier B.V. This is an open access article under the CC BY license (http://creativecommons.org/licenses/by/4.0/).

that resistance exercise training (RET) significantly reduces anxiety symptoms among both healthy adults (Δ=0.50) and those with a physical/mental illness (Δ=0.19) (Gordon et al., 2017). However, only two included RET trials randomized participants with any mental illness (Vlachopoulo et al., 2005; Herring et al., 2011), and only one randomized participants with clinically relevant anxiety pathology (Herring et al., 2011). These trials were neither designed in accordance with World Health Organization (WHO) (WHO, 2011) and American College of Sports Medicine (ACSM) (ACSM, 2009) guidelines for muscle strengthening exercise, nor designed to be ecologically-valid. The WHO recommends partaking in muscle-strengthening activities involving major muscle groups on two or more days a week (WHO, 2011); ACSM recommends progressive strength training a minimum of two non-consecutive days each week, with 1-3 sets of 8 to 12 repetitions for muscular strength benefits in novices (ACSM, 2009).

The only randomized controlled trial (RCT) of RET among people with an anxiety disorder to date reported improved clinical severity (Herring et al., 2012) and associated symptoms (Herring et al., 2011) among young adult women with GAD. However, this trial featured only three lower-body, machine-based exercises, and was not designed in accordance with ACSM guidelines. The extent to which ecologically-valid RET (i.e., RET following currently recommended guidelines, and using standard movements that can be done at home or at a gym, including the RET frequency, composition, intensity, and progression) designed in accordance with WHO and ACSM guidelines improves these signs and symptoms of GAD, particularly among adults with prodromal, or analogue GAD (AGAD), is unknown (Gordon et al., 2017). The median age of first onset of GAD is 30 (Kessler et al., 2012), and individuals who display elevated subclinical symptoms are more likely to develop clinically significant psychopathology (Ruscio et al., 2007; Wolitzky-Taylor et al., 2014).

Thus, the aim of this RCT was to quantify the effects of ecologically-valid RET on AGAD status and worry and anxiety symptoms among young adults with AGAD. Based on previous evidence (Herring et al., 2012; Gordon et al., 2017), the authors hypothesized that RET would improve AGAD status, and elicit moderate magnitude reductions in worry and anxiety symptoms.

Methods

This pilot efficacy trial has adhered to the Consolidated Standards of Reporting Trials (CONSORT) Checklist (Schulz et al., 2010).

Trial Design

This manuscript presents findings from one of two parallel eight-week RCTs (ClinicalTrials.gov Identifier: NCT04116944). The full methods of these RCTs were reported previously (Gordon et al., 2020). The research protocol was approved by the University's Research Ethics Committee (EHSREC No: 2017_03_18_EHS); all participants provided written informed consent prior to participation. This trial had rolling recruitment; data collection began January 18th, 2018 and concluded June 26th, 2019.

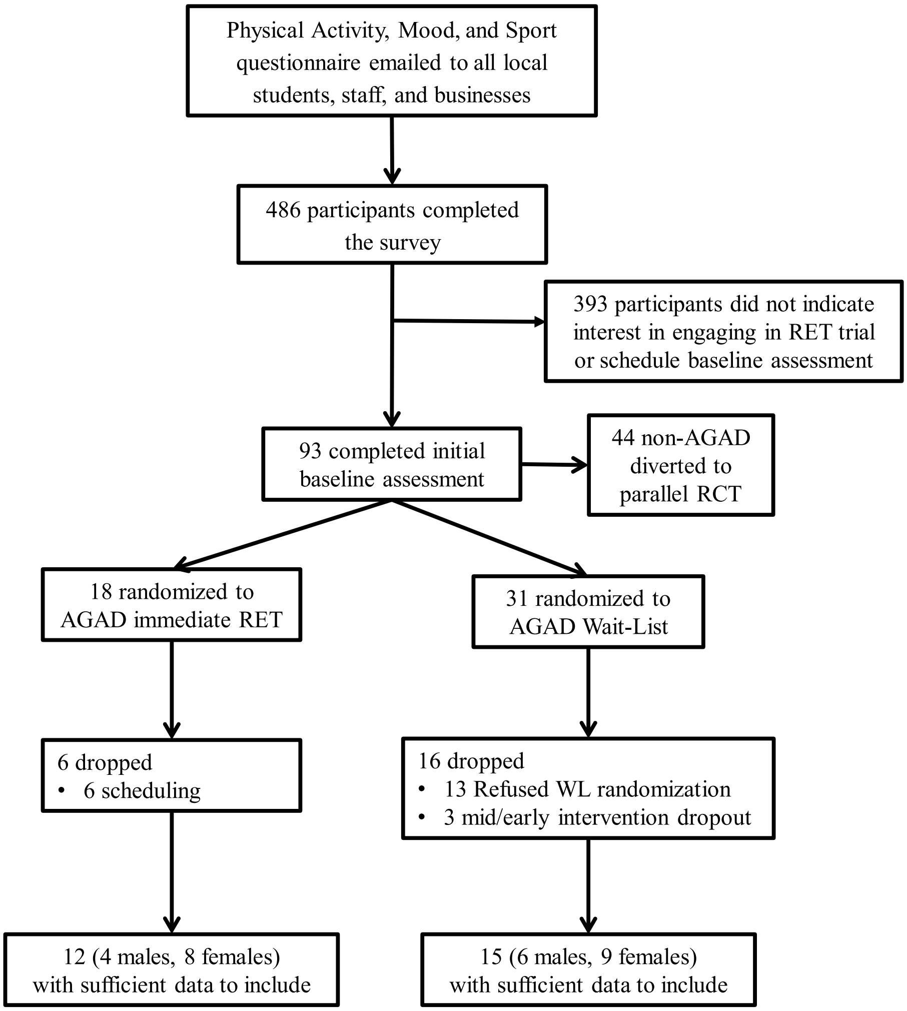

Fig. 1. Flow chart of included participants.

Participants

Participants were recruited from the surrounding area via posters, emails, and word of mouth. This trial had rolling recruitment; data collection for this trial began January 18th, 2018, and concluded June 26th, 2019. Potential participants initially completed an electronic battery of questionnaires to establish eligibility; Fig. 1 presents a flowchart of participant recruitment.

At baseline, participants completed a battery of online questionnaires, including the 10-item GAD subscale of the Psychiatric Diagnostic Screening Questionnaire (PDSQ-GAD) (Zimmerman & Mattia, 2001a), 16-item Penn State Worry Questionnaire (PSWQ) (Meyer et al., 1990) and several other measures of signs and symptoms of GAD (i.e., depressive symptoms, irritability) (Rush et al., 2003; Craig et al., 2008). Participants were classified as either AGAD (PDSQ-GAD ≥6 *and* PSWQ ≥45) or non-AGAD. Participants who did not meet or exceed both cut-off scores were considered non-AGAD, and diverted to a parallel RCT. Following AGAD categorization, participants were randomized, stratified by sex, to RET or a wait-list control.

Inclusion criteria were: i) age 18-40y; ii) scores that met criteria for AGAD (PDSQ-GAD ≥6 *and* PSWQ ≥45); iii) no medical contraindication to safe participation in RET; and, iv) no current pregnancy or lactation. Current RET involvement at baseline was not an exclusion criterion. Participants currently engaging in any form of resistance exercise training or physical activity were eligible for this study. To account for RET-specific familiarity/baseline physical activity levels, participants were asked, in reference to the time of the baseline assessment, how long they had currently been involved in a formalized resistance exercise training program. Participants' previous self-reported RET involvement was measured in weeks to quantify participant training age. Although not an exclusion criterion, all participants had a training age of zero, meaning they were not currently involved in RET.

Participants were not excluded if they were in treatment for anxiety, depression, or other mental health disorders (Rebar et al., 2017). Four participants currently receiving treatment for depression completed the trial (RET n=2, wait-list n=2). Treatment included pharmacotherapy (RET n=1, wait-list n=1), psychotherapy (wait-list n=1), or both (RET n=1). Six participants receiving treatment for depression withdrew (RET n=2, wait-list n=4). No participants reported receiving treatment for GAD.

Participants randomized to both groups were advised to maintain their current levels of physical activity throughout the trial. Based on previous meta-analytic evidence of the small-to-moderate effect of RET on anxiety (Δ=0.31) (Gordon et al., 2017), a priori power analysis with G*Power 3.1 indicated a sample size of 24 (12 in each group) would provide >80% statistical power (two-tailed α=0.05, four repeated measures) to detect a small-to-moderate effect of exercise on worry symptoms (the hallmark symptom of GAD). As this study was considered a pilot efficacy trial for a larger more expansive trial, and attrition is common among physical activity interventions, recruitment continued until at least 10 participants were allocated to each group. At this point, 12 participants were included in the RET condition, and 15 participants were included in the wait-list condition.

RET Intervention

RET was designed in accordance with WHO and ACSM guidelines (WHO, 2011; ACSM, 2009). The eight-week, twice-weekly intervention increased resistance progressively, such that the participant could complete between 8-12 repetitions before experiencing either fatigue, a deterioration in lifting form, or failure to complete a repetition. The eight exercises were barbell squat, barbell bench press, hexagon bar deadlift, seated dumbbell shoulder lateral raise, barbell bent over rows, dumbbell lunges, seated dumbbell curls, and abdominal crunches. Participants randomized to RET completed a three-week, twice-weekly, familiarization process to ensure safety, correct lifting technique, and that the entirety of the eight-week intervention was delivered at the correct resistance starting at week one. Fully supervised exercise sessions were approximately 25 minutes, on a one-to-one basis in a RET facility. All investigators were fully trained in delivering the exercise protocol consistently, as well as identifying proper and improper lifting mechanics. Participants completed primary and secondary outcome questionnaires supervised in the RET facility prior to RET bouts. Further specifics of the RET intervention have been previously published (Gordon et al., 2020).

Control Condition

Participants randomized to the wait-list completed primary and secondary outcome questionnaires supervised in the RET facility at baseline, week one, and week eight. Week four questionnaires were e-mailed to participants. Participants that completed the eight-week wait-list condition were subsequently offered the RET intervention, but no data were collected.

Primary Outcome

The primary outcome dependent variable of the trial was AGAD status, determined by scoring above or below validated cut-scores for both the PDSQ-GAD (≥6) and PSWQ (≥45). The PDSQ-GAD subscale has a sensitivity/specificity of 94/41%, strong internal consistency (α=0.89), positive and negative predictive value (25/98%), and test-retest reliability (α=0.58) (Zimmerman & Mattia, 2001a; 2001b). The PSWQ has a strong test-retest reliability (α=0.92) and internal consistency (α=0.95). A cut score of 45 has shown sensitivity/specificity for GAD of 99/98% (Meyer et al., 1990). As RET can elicit rapid reductions in worry and anxiety symptoms (Herring et al., 2012), The PDSQ-GAD and PSWQ were assessed at baseline, week one, week four, and week eight.

Secondary Outcome

Secondary outcome dependent variables included worry, worry-engagement, and absence of worry assessed with the 16-item PSWQ. Total worry, worry-engagement, and absence of worry scores were calculated according to recommendations (Fresco et al., 2002). Worry symptoms were measured at baseline, week one, week four, and post-intervention. The PSWQ demonstrated good internal consistency α=0.84 (ICC=0.82, 95%CI: 0.70 to 0.90). Correlations between repeated measures at baseline and week one were 0.70 ($p \leq 0.01$) and 0.78 ($p \leq 0.001$) for the RET and wait-list groups, respectively. Worry-engagement demonstrated good internal consistency α=0.81 (ICC=0.78, 95%CI: 0.64 to 0.89). Correlations between repeated measures were 0.60 ($p \leq 0.04$) and 0.76 ($p \leq 0.001$) for RET and wait-list groups, respectively. Absence of worry demonstrated acceptable internal consistency α=0.65 (ICC=0.59, 95%CI: 0.30 to 0.79). Correlations between repeated measures were 0.33 ($p \geq 0.29$) and 0.72 ($p \leq 0.002$) for RET and wait-list groups, respectively.

Anxiety symptoms were measured with the trait subscale of the State-Trait anxiety inventory (STAI-Y2) (Spielberger & Gorsuch, 1983). The 20-item STAI-Y2 is the most widely-used anxiety measure in the literature (Gordon et al., 2017), and has shown sensitivity to change in response to even short-term RET (Herring et al., 2011). Anxiety symptoms were measured in the same battery as the PDSQ-GAD and PSWQ at baseline, week one, week four, and week eight. The STAI-Y2 demonstrated good internal consistency α=0.81 (ICC=0.89, 95%CI: 0.81 to 0.94). Correlations between repeated measures were 0.64 ($p \leq 0.02$) and 0.87 ($p \leq 0.001$) for RET and wait-list groups, respectively.

Covariates

Baseline physical activity was assessed using an online, self-report

version of the seven-day Physical Activity Recall (Blair et al., 1985). Participants reported time engaged in sleep, moderate, hard, and very hard activities during the prior week. Estimated energy expenditure was calculated as kilocalories per week. According to thresholds validated by Dishman and Steinhardt (1988), participants can be considered inactive (<245 kcals/week) to highly active (≥280 kcals/week).

Intervention Fidelity and Manipulation Check

Attendance was calculated by dividing the number of RET bouts attended by 16 (two sessions per week x eight weeks). Compliance to the RET protocol was calculated by dividing the number of sets in which at least eight repetitions were completed by 256 (two sets x eight exercises x two sessions per week x eight weeks), which represents the minimum prescribed number of repetitions.

To quantify anticipated changes in strength as a manipulation check, and to facilitate setting of load, participants completed a five-repetition max (5RM) assessment for the barbell squat, barbell bench press, and hexagon bar deadlift at baseline and post-intervention. During the six familiarization sessions, participants completed two familiarizations with the 5RM process, and one maximal 5RM assessment.

Statistical analyses

Data analyses were performed using SPSS 25.0. Missing data for both the STAI-Y2 and PSWQ were 3.7%. Missing data for STAI-Y2 (n=5) and PSWQ (n=5) were imputed: sex and time-variant responses for each variable were entered as predictors into separate multiple linear regression models for condition, and predicted values were retained. Participants (n=16) were excluded if they were missing primary outcome data at >1 time point. Intention-to-treat analyses, and analyses of complete cases only are reported as sensitivity analyses. Changes in AGAD status, considered remission herein, were analysed using the number needed to treat (NNT) (Cook & Sackett, 1995). The NNT and associated 95%CIs were calculated as the inverse of the absolute risk reduction for RET compared with the wait-list condition. NNT was rounded up to the nearest whole number, and 95%CIs were converted from Hedges' *d* effect size for worry symptoms (Cook & Sackett, 1995).

Independent samples *t*-tests examined baseline differences between groups and sexes. The magnitude of baseline differences were quantified using Cohen's d effect sizes (Rosenthal, 1994). Two group (RET/wait--list) x four time (baseline/week one/week four/post-intervention) repeated measures ANCOVA examined differences between RET and wait-list; age, sex, and baseline physical activity were covariates. The Huynh-Feldt adjustment was applied when sphericity was violated. Significant interactions were decomposed using simple effects analysis. Standardized mean difference (SMD) quantified the magnitude of within-condition change. The magnitude of difference in outcome change between groups was quantified by Hedges' *d* effect sizes and associated 95%CIs (Hedges & Olkin, 1985). Hedges' *d* effect sizes were calculated by subtracting the mean change in the wait-list from the mean change in the RET condition, and dividing this difference by the pooled standard deviation of baseline scores; effect sizes were adjusted for small sample size bias and calculated such that improved outcomes in each condition and larger improvements among RET compared to wait-list resulted in positive effect sizes. Changes in strength were examined with paired-samples *t*-tests. Associations between changes in strength and changes in worry and anxiety symptoms were quantified using Pearson correlation coefficients of associations between change scores

Results

Table 1 presents baseline participant characteristics and differences between groups among included participants. There were no baseline differences between groups among included participants on any outcomes, supporting successful randomization. There were no baseline differences between sexes among included participants on any outcomes.

Table 1
Baseline differences among included participants.

Variable	RET (n=12) Mean (SD)	WL (n=15) Mean (SD)	*t*	*p*	Cohen's d
% Female	66.67	66.67			
Age(y)	26.5(5.8)	26.7(4.9)	-0.081	0.94	-0.03(-0.79 to 0.73)
Body Mass Index	24.4(4.1)	26.0(4.5)	-0.97	0.34	-0.37(-1.14 to 0.39)
Symptoms of GAD (PDSQ-GAD)	7.8(1.3)	7.7(1.3)	0.20	0.84	0.08(-0.68 to 0.84)
Worry Symptoms (PSWQ)	63.5(9.5)	61.2(8.3)	0.67	0.51	0.26(-0.50 to 1.02)
Worry-Engagement (PSWQ-WE)	42.7(7.4)	40.7(5.9)	0.76	0.46	0.29(-0.47 to 1.06)
Absence of Worry (PSWQ-AW)	20.8(2.7)	21.1(4.3)	-0.22	0.83	-0.08(-0.84 to 0.67)
Anxiety Symptoms (STAI-Y2)	51.5 (10.7)	55.1(9.1)	-0.96	0.35	0.14(-1.14 to 0.39)
Physical Activity (kcals/week)	254.4 (16.8)	277.0 (54.8)	-1.51	0.15	-0.58(-1.36 to 0.19)

SD=Standard Deviation; RET=Resistance Exercise Training; WL=Wait-list; PDSQ-GAD=Psychiatric Diagnostic Screening Questionnaire-Generalized Anxiety Disorder subscale; PSWQ=Penn State Worry Questionnaire; PSWQ-WE=Penn State Worry Questionnaire-Worry Engagement; PSWQ-AW=Penn State Worry Questionnaire-Absence of Worry; STAI-Y2=Trait Anxiety Inventory.

Intervention Fidelity and Manipulation Check

The average attendance to the RET intervention was 81% (13 out of 16 sessions). The average compliance with RET was 77% (197 out of 256 repetitions). These attendance and compliance rates indicate that participants missed approximately three sessions over the intervention, but were compliant when they attended. No adverse events arose from trial participation. However, one participant randomized to RET reported a headache during an exercise bout. The investigator supervising the bout immediately stopped the session, and the participant subsequently withdrew from the trial after consulting with a physician. The average rating of perceived exertion was 14±2 (between somewhat hard and hard, range 6 to 20) (Heath, 1998); average muscle soreness was 4±2 out of 10 (between mild soreness and some soreness present). As anticipated, participants in the RET intervention significantly increased their total strength ($t_{(8)}$=-6.86, $p \leq 0.001$, Cohen's d=2.29, mean increase: 23.4%±11.8).

Number Needed to Treat

Based on AGAD remission, there was a NNT of 3 (95%CI: 2 to 17). For transparency, Table 2 indicates NNT values through alternative operational definitions of AGAD status.

Worry Severity and Anxiety Symptoms

Table 3 presents descriptives, standardized mean differences, and Hedges' *d* (95%CI) for outcomes. Significant group X time interactions were found for worry symptoms ($F_{(3,66)}$=3.60, $p \leq 0.04$; d=0.93, 95%CI: 0.13 to 1.73), worry-engagement ($F_{(3,66)}$=3.31, $p \leq 0.03$; d=0.98, 95%CI: 0.18 to 1.79), and anxiety symptoms ($F_{(3,57.84)}$=2.91, ε=0.88, $p \leq 0.045$; d=0.71, 95%CI: -0.08 to 1.49). RET significantly reduced worry symptoms (mean difference=-6.49, $p \leq 0.04$), non-significantly reduced worry engagement (mean difference=-5.49, $p \geq 0.059$), and significantly reduced anxiety symptoms (mean difference=-10.50, $p \leq 0.001$) from

Table 2
Number needed to treat values of varying operational definitions of Analogue Generalized Anxiety Disorder status.

AGAD-Status categorizations	Resistance Exercise Training Remission	Wait-list Remission	Number Needed to Treat
PDSQ-GAD <6, **OR** PSWQ <45	6	2	3
PDSQ-GAD <6, **AND** PSWQ <45	2	1	10
PDSQ-GAD <6	6	2	3
PSWQ <45	2	1	10
PSWQ Worry symptom reduction magnitude (Hedges' *d*)			3
STAI-Y2 Anxiety symptom reduction magnitude (Hedges' *d*)			4

PDSQ-GAD=Psychiatric Diagnostic Screening Questionnaire-Generalized Anxiety Disorder subscale; PSWQ=Penn State Worry Questionnaire; STAI-Y2=Trait Anxiety Inventory.

baseline to post-intervention. There was no significant interaction for absence of worry ($F_{(3,69)}$=1.16, $p \geq 0.33$; *d*=-0.58, 95%CI: -1.36 to 0.19). Changes in strength were not significantly associated with changes in worry symptoms ($r_{(9)}$=0.20, $p \leq 0.60$), worry engagement ($r_{(9)}$=0.29, $p \leq 0.46$), absence of worry ($r_{(9)}$=-0.54, $p \leq 0.13$), or anxiety symptoms ($r_{(9)}$=0.39, $p \leq 0.30$). Table 4 presents Hedges' *d* (95%CI) for outcomes at each measurement time-point.

The magnitude of reductions for worry symptoms (*d*=1.07, 95%CI: 0.45 to 1.68), worry engagement (*d*=1.10, 95%CI: 0.48 to 1.72), absence of worry (*d*=-0.55, 95%CI: -1.15 to 0.04), and anxiety symptoms (*d*=0.87, 95%CI: 0.27 to 1.48) with intention-to-treat analysis did not differ from primary analyses. The magnitude of reductions also did not differ for worry symptoms (*d*=1.04, 95%CI: 0.19 to 1.89), worry engagement (*d*=1.13, 95%CI: 0.27 to 1.99), absence of worry (*d*=-0.32, 95%CI:-1.23 to 0.49), and anxiety symptoms (*d*=0.64, 95%CI: -0.18 to 1.46) using only complete cases.

Discussion

This RCT specifically addressed recent calls from the United States Physical Activity Guidelines Advisory Committee Scientific Report to conduct RCTs in individuals at different stages or severity of impairment (i.e., AGAD), to examine whether physical activity, in this case RET, delays or prevents disease onset and progression (United States Physical Activity Guidelines Advisory Committee, 2018). This is critically important, because intervention at early points on the severity spectrum may significantly reduce the probability of future clinical psychopathology (Wolitzky-Taylor et al., 2014). The cumulative evidence supports the recommendations for prescription and end-user engagement in guidelines-based aerobic and RET as an efficacious alternative or augmented therapy for worry and anxiety symptoms.

Ecologically-valid RET, designed according to WHO and ACSM guidelines, significantly improved AGAD status among young adults with AGAD. The NNT of three indicates that AGAD remission would be expected to occur for at least one of every three participants who would engage in this guidelines-based RET. This NNT of three is consistent with previous evidence among young women with GAD (Herring et al., 2012). The NNT is smaller than previous meta-analytic evidence for antidepressant treatment in GAD (NNT=5.15) (Kapczinski et al., 2003), and consistent with the NNT for cognitive behavioural therapy in GAD (NNT=2.23) (Cuijpers et al., 2014). The consistency in NNT between RET and other frontline treatments for GAD directly supports RET as an alternative or augmentation therapy for AGAD and GAD.

The large magnitude reductions in worry symptoms found here (*d*=0.93) are larger than previous evidence for RET for worry among young adult women with GAD (*d*=0.45) (Herring et al., 2012). The large magnitude reductions in anxiety symptoms (*d*=0.71) and worry symptoms are clinically meaningful, based on a frequently used response threshold of a 50% or greater reduction in baseline scores, or on a minimally important difference threshold of 0.5 standard deviation units (Rush et al., 2003). The magnitude of reductions in anxiety symptoms are larger than previous meta-analytic evidence for RET for anxiety among those with physical/mental illness (Δ=0.19) (Gordon et al., 2017). The magnitude of reductions in anxiety symptoms are also larger than previous meta-analytic evidence for all/diverse types of exercise for otherwise healthy adults (Δ=0.22) (Conn, 2010) and chronically-ill adults (Δ=0.29) (Herring et al., 2012), and experimental

Table 4
Changes in outcomes by time-points of intervention.

Outcome	Hedges' *d* Baseline to Week 1	Hedges' *d* Week 1 to Week 4	Hedges' *d* Week 4 to Week 8
Worry Symptoms (PSWQ)	0.69 (-0.09 to 1.47)	0.02 (-0.74 to 0.78)	0.19 (-0.58 to 0.95)
Worry-Engagement (PSWQ-WE)	0.73 (-0.04 to 1.52)	0.19 (-0.57 to 0.95)	0.004 (-0.76 to 0.76)
Absence of Worry (PSWQ-AW)	0.15 (-0.62 to 0.91)	-0.28 (-1.04 to 0.49)	-0.58 (1.35 to 0.20)
Anxiety Symptoms (STAI-Y2)	0.17(-0.59 to 0.93)	0.34 (-0.42 to 1.11)	0.21 (-0.55 to 0.97)

STAI-Y2=Trait Anxiety Inventory; PSWQ=Penn State Worry Questionnaire; PSWQ-WE=Penn State Worry Questionnaire-Worry Engagement; PSWQ-AW; Penn State Worry Questionnaire-Absence of Worry.

Table 3
Changes in worry and anxiety symptoms from baseline.

Outcome	Group	Baseline	Week 1	SMD	Hedges' *d* from Baseline	Week 4	SMD	Hedges' *d* from Baseline	Week 8	SMD	Hedges' *d* from Baseline
Worry Symptoms (PSWQ)	RET	63.5(9.5)	59.3 (13.1)	0.45	0.69 (-0.09 to 1.47)	59.0(9.9)	0.48	0.71 (-0.07 to 1.50)	56.2(11.1) *	0.78	0.93 (0.13 to 1.73)
	WL	61.2(8.3)	63.2(7.4)	-0.24		63.2 (10.6)	-0.24		62.3(8.9)	-0.14	
Worry-Engagement (PSWQ-WE)	RET	42.7(7.4)	39.5 (11.1)	0.43	0.73 (-0.04 to 1.52)	38.3(8.4)	0.59	0.98 (0.18 to 1.78)	36.5(10.4)	0.83	0.98 (0.18 to 1.79)
	WL	40.7(5.9)	42.6(5.3)	-0.32		43.1(8.0)	-0.39		41.3(6.9)	-0.26	
Absence of Worry (PSWQ-AW)	RET	20.8(2.7)	19.8(3.0)	0.40	0.15 (-0.62 to 0.91)	20.3(2.3)	0.22	-0.07 (-0.83 to 0.68)	22.8(8.9)	-0.69	-0.58 (-1.36 to 0.19)
	WL	21.1(4.3)	20.6(2.9)	0.12		20.3(3.8)	-0.2		20.9(3.2)	-0.15	
Anxiety Symptoms (STAI-Y2)	RET	51.5 (10.7)	48.8 (10.8)	0.26	0.17 (-0.59 to 0.93)	44.9 (11.3)	0.62	0.49 (-0.28 to 1.26)	40.4(10.2) *	1.04	0.71 (-0.08 to 1.49)
	WL	55.1(9.1)	54.1(7.8)	0.12		53.8(9.6)	0.18		51.2(8.5)	0.43	

RM-ANCOVA controlling for sex, age, and non-intervention physical activity; *indicates a significant difference from the baseline score in simple effects analyse. SMD=Standardized Mean Difference; RET=Resistance Exercise Training; WL=Wait-list; STAI-Y2=Trait Anxiety Inventory; PSWQ=Penn State Worry Questionnaire; PSWQ-WE=Penn State Worry Questionnaire-Worry Engagement; PSWQ-AW= Penn State Worry Questionnaire-Absence of Worry.

evidence for RET for anxiety among young adult women with diagnosed GAD (d=0.52) (Herring et al., 2011). This is the second RET trial to demonstrate large reductions in worry and anxiety symptoms among young adults with GAD or AGAD (Herring et al., 2012; Herring et al., 2011), and the first ecologically-valid RET trial, designed in accordance with WHO and ACSM guidelines, to demonstrate such large reductions.

The reductions in worry symptoms in the RET group were larger than predicted; specifically, the rapid, moderate-to-large magnitude improvement in worry symptoms across the familiarization sessions from baseline to week one of training were larger than predicted. Although the dose of RET during the three-week familiarization process was minimal, the reductions in worry symptoms that occurred in the three weeks following baseline to week one (d=0.69) are consistent with evidence that RET can elicit reductions in worry symptoms among young adult women with GAD in two weeks (d=0.33) (Herring et al., 2012). The reductions in anxiety symptoms from baseline to week one (d=0.17) are consistent with the reductions in anxiety symptoms among young adult women with GAD in two weeks (d=0.14) (Herring et al., 2011).

Adherence of 81% and compliance to RET of 77% supports that the RET intervention was feasible and tolerable among young adults with AGAD. These attendance and compliance rates indicate that participants missed approximately three sessions over the intervention, but were compliant when they attended. The high attendance and compliance reported here, as well as exceedingly high attendance (100%) and compliance (99.1%) rates reported for the lone previous RCT of RET among young adult women with GAD (Herring et al., 2012), provides promising support for RET as an alternative/augmentation therapy.

Changes in strength were not correlated with changes in worry or anxiety symptoms. Recent meta-analytic evidence supports significant improvements in strength are not required for mental health benefits (Gordon et al., 2017; 2018). However, as anticipated, participants showed large significant improvements in strength (all $d \geq 1.24$, $p \leq 0.006$). These associated strength changes may have important implications specifically for young adults with AGAD. Given that GAD risk typically arises during the teen years and progresses relatively linearly into adulthood (Lieb, Becker, & Altamura, 2005), RET interventions targeting these adolescent and young adult populations, who may otherwise not be engaging in any muscle strengthening activity, are particularly important. Additionally, future trials including larger samples should investigate the relationship between changes in strength and changes in mental health outcomes in maximum tolerable doses and minimal effective doses of guidelines-based RET in healthy and clinical populations less willing to comply to progressive RET interventions.

There are several potential mechanisms that may help to explain why large magnitude reductions in anxiety occurred in response to RET among young adults with AGAD. Although social interaction was rigorously controlled (Gordon et al., 2020), RET participants may have benefited from the increased amount of social interaction during the exercise bouts. Neurobiological theories involve systems that are involved in both how anxiety develops, and how exercise affects the brain (Dishman et al., 2006). Potential RET-specific mechanisms include increases in insulin-like growth factor 1 (IGF-1). Recent animal models showed that hippocampal IGF-1was increased in rats after RET, and not in wheel running (Cassilhas et al., 2012). Increase in serum IGF-1 is associated with neurogenesis, and reduced anxiety-like behaviours in mice. (Ding et al., 2006). More research is needed regarding the specific mechanisms underlying RET effects on anxiety and worry symptoms, particularly those that may be unique to RET, and unique to individuals with AGAD or anxiety disorders.

Generalizability

Although findings are based on a small sample of young adults, the anxiety and worry symptom reductions found here are generalizable other populations of young adults, and may have implications for middle-aged and older adults. Although the worldwide prevalence of GAD among those 15-45y ranges from 1.4-4.1% (Bandelow & Michaelis, 2015), there is no information regarding the prevalence of AGAD in this population. However, of the 93 participants who completed the baseline screening, 51 (55%) met AGAD criteria, indicating high prevalence of elevated worry symptoms in the small population of potential participants. The population of young adults aged 18-40y in Ireland is ~1.5 million (Central Statistics Office, 2016); based on the prevalence rate assessed here, 810,000 young adults in Ireland would be expected to meet criteria for AGAD. If the entirety of this population were compliant with guidelines-based RET, based on the AGAD remission demonstrated here (NNT=3), remission would be expected to occur in approximately 270,000 young adults. Although it would be logistically impossible to provide one-on-one RET at such a large scale, there are several ways to engage in ACSM and WHO guidelines-based RET at minimal cost and equipment needs, or at home using only body-weight exercises. Therefore, policy makers, universities, and organizations should devise ways to make RET accessible, and encourage all individuals not meeting guidelines to engage in aerobic activity and RET consistent with the guidelines of the WHO and ACSM for physical and mental health benefit.

This RCT has clear implications for the efficacy of RET as an alternative or augmentation therapy for anxiety disorders among young adults, and the development of guidelines for treatment among those most at risk for developing anxiety disorders, particularly the prescription of RET as an alternative or augmentation therapy among young adults with elevated worry. Clinicians should evaluate and monitor patients' physical activity involvement, and encourage patients to engage in guidelines-based RET. Further, although the RET exercises are generalizable to the general population, there are myriad alternative RET options as well, including home-based RET, equipment free RET, or RET focused on muscular hypertrophy or power (ACSM, 2009). Clinicians should encourage patients to engage in guidelines-based RET as an alternative treatment for GAD, and augment other empirically-supported treatments for anxiety, such as cognitive behavioural therapy and pharmacotherapy, with RET.

Limitations

Limitations include a lack of a time-matched, attention-control condition. Participants in the RET intervention may have benefited from the attention and social interaction provided to them not provided to the wait-list condition. Further, the lack of follow-up assessment limits the study's ability to determine if reductions in symptoms are sustained. As this was a pilot efficacy trial, the sample size was small. Although this trial was sufficiently powered to detect small-to-moderate reductions in worry symptoms, future trials would benefit from larger sample sizes to explore potential sex-related response differences to RET and plausible mediators/moderators of response. Additionally, although participants met or exceeded established cut scores sensitive and specific to GAD, the use of blinded clinical interviews assessing the presence or absence of anxiety and related disorders would more rigorously assess baseline AGAD status.

Conclusion

Ecologically-valid, guidelines-based RET significantly improved AGAD status and resulted in moderate-to-large magnitude, clinically meaningful improvements in symptoms of worry and anxiety among young adults with elevated worry indicative of AGAD. Future trials should replicate and expand these findings to explore sex-related differences, examine putative psychobiological mechanisms of the anxiolytic effects of RET, and augment other established treatments for anxiety, such as psychotherapy and pharmacotherapy, with RET.

Authors' Contributions

All authors made substantial contributions to conception and design, acquisition of data, and preparation of the manuscript. All authors have read and approved the final version of the manuscript, and agree with the order of presentation of the authors.

Statement of Ethics

The research protocol was approved by the University's Research Ethics Committee, and all participants provided written informed consent prior to participation.

Conflicts of Interests and Source of Funding

Brett R. Gordon was funded by the Irish Research Council under the Government of Ireland Postgraduate Programme. Cillian P. McDowell is funded by the Irish Research Council under the Government of Ireland Postdoctoral Programme. No authors declare any conflicts of interest. The results of the present study do not constitute endorsement by ACSM. The results of the study are presented clearly, honestly, and without fabrication, falsification, or inappropriate data manipulation.

Data Availability

De-identified individual participant data for primary outcomes measures analysed during the current study will be made available for five years at six months following publication of primary outcome measures summary data.

References

American College of Sports Medicine, 2009. American college of sports medicine position stand. progression models in resistance training for healthy adults. Med Sci Sports Exerc 41, 687–608, doi:10.1249/MSS.0b013e3181915670.

Andlin-Sobocki, P., Wittchen, H.U., 2005. Cost of anxiety disorders in Europe. Eur J Neurol 12, 39–44. https://doi.org/10.1111/j.1468-1331.2005.01196.x.

Baxter, A.J., Vos, T., Skott, K.M., et al., 2014. The global burden of anxiety disorders in 2010. Psychol Med 44, 2363–2374. https://doi.org/10.1017/S0033291713003243.

Blair, S.N., Haskell, W.L., Ho, P., et al., 1985. Assessment of habitual physical activity by a sevenday recall in a community survey and controlled experiments. Am J Epidemiol 122, 794–804. https://doi.org/10.1093/oxfordjournals.aje.a114163.

Bystritsky, A., 2006. Treatment-resistant anxiety disorders. Mol Psychiatry 11, 805–814. https://doi.org/10.1038/sj.mp.4001852.

Caspersen, C.J., Powell, K.E., Christenson, G.M., 1985. Physical activity, exercise, and physical fitness: definitions and distinctions for health-related research. Public Health Rep 100 (2), 126–131. PMID: 3920711; PMCID: PMC1424733.

Cassilhas, R.C., Lee, K.S., Fernandes, J., et al., 2012. Spatial memory is improved by aerobic and resistance exercise through divergent molecular mechanisms. Neuroscience 27, 309–317. https://doi.org/10.1016/j.neuroscience.2011.11.029.

Central Statistics Office, 2016. In: Census 2016 Summary Results. https://www.cso.ie/en/csolatestnews/presspages/2017/census2016summaryresults-part1/. Accessed 15/11/19.

Conn, V.S., 2010. Anxiety outcomes after physical activity interventions: meta-analysis findings. Nurs Res 59, 224–231. https://doi.org/10.1097/NNR.0b013e3181dbb2f8.

Cook, R.L., Sackett, D.L., 1995. The number needed to treat: a clinically useful measure of treatment effect. BMJ 310, 452–454. https://doi.org/10.1136/bmj.310.6977.452.

Cuijpers, P., Sijbrandij, M., Koole, S., et al., 2014. Psychological treatment of generalized anxiety disorder: a meta-analysis. Clin Psychol Rev 34, 130–140. https://doi.org/10.1016/j.cpr.2014.01.002.

Ding, Q., Vaynman, S., Akhavan, M., et al., 2006. Insulin-like growth factor I interfaces with brain-derived neurotrophic factor-mediated synaptic plasticity to modulate aspects of exercise-induced cognitive function. Neurosci 140, 823–833. https://doi.org/10.1016/j.neuroscience.2006.02.084.

Dishman, R.K., Berthoud, H.R., Booth, F.W., et al., 2006. Neurobiology of exercise. Obesity 14, 345–356. https://doi.org/10.1038/oby.2006.46.

Dishman, R.K., Steinhardt, M., 1988. Reliability and concurrent validity for a 7-d re-call of physical activity in college sutdents. Med Sci Sports Exerc 20 (1), 14–25. https://doi.org/10.1249/00005768-198802000-00003.

Fresco, D.M., Frankel, A.N., Mennin, D.S., et al., 2002. Confirmatory factor analysis of the Penn state worry questionnaire. Behav Res Ther 40, 313–323. https://doi.org/10.1016/s0005-7967(00)00113-3.

Gordon, B.R., McDowell, C.P., Hallgren, M., Meyer, J.D., Lyons, M., Herring, M.P., 2018. Association of efficacy of resistance exercise training with depressive symptoms: meta-analysis and meta-regression analysis of randomized clinical trials. JAMA Psychiatr 75 (6), 566–576. https://doi.org/10.1001/jamapsychiatry.2018.0572.

Gordon, B.R., McDowell, C.P., Lyons, M., et al., 2017. The effects of resistance exercise training on anxiety: a meta-analysis and meta-regression analysis of randomized controlled trials. Sports Med 47, 2521–2532. https://doi.org/10.1007/s40279-017-0769-0.

Gordon, B.R., McDowell, C.P., Lyons, M., et al., 2020. Acute and chronic effects of resistance exercise among young adults with and without analogue generalized anxiety disorder: a protocol for pilot randomized controlled trials. Ment Health Phys Act 18, 100321. https://doi.org/10.1016/j.mhpa.2020.100321.

Heath, E.M., 1998. Borg's perceived exertion and pain scales. Med Sci Sports Exerc 30, 1461.

Hedges, L.V., Olkin, I.., 1985. Statistical methods for meta-analysis. Academic Press, New York, pp. 76–104.

Herring, M.P., Jacob, M.L., Suveg, C., et al., 2011. Effects of short-term exercise training on signs and symptoms of generalized anxiety disorder. Ment Health Phys Act 4, 71–77. https://doi.org/10.1016/j.mhpa.2011.07.002.

Herring, M.P., Jacob, M.L., Suveg, C., et al., 2012. Feasibility of exercise training for the short-term treatment of generalized anxiety disorder: a randomized controlled trial. Psychother Psychosom 81, 21–28. https://doi.org/10.1159/000327898.

Herring, M.P., O'Connor, P.J., Dishman, R.K., 2010. The effect of exercise training on anxiety symptoms among patients: a systematic review. Arch Intern Med 170, 321–331. https://doi.org/10.1001/archinternmed.2009.530.

Kapczinski, F., dos Santos-Souza, J.J., da Cunha, A.A., et al., 2003. Antidepressants for generalized anxiety disorder. Cochrane Database Syst Rev, CD003592. https://doi.org/10.1002/14651858.CD003592.

Kessler, R.C., Aguilar-Gaxiola, S., Alonso, J., et al., 2009. The global burden of mental disorders: an update from the WHO World Mental Health (WMH) surveys. Epidemiol Psichiatr Soc 28, 23–33. https://doi.org/10.1017/s1121189X00001421.

Kessler, R.C., Petukhova, M., Sampson, N.A., et al., 2012. Twelve-month and lifetime prevalence and lifetime morbid risk of anxiety and mood disorders in the United States. Int J Methods Psychiatr Res 21, 169–184. https://doi.org/10.1002/mpr.1359.

Lieb, R., Becker, E., Altamura, C., 2005. The epidemiology of generalized anxiety disorder in Europe. Eur Neuropsychopharmacol 15, 445–452. https://doi.org/10.1016/j.euroneuro.2005.04.010.

McDowell, C.P., Dishman, R.K., Gordon, B.R., et al., 2019. Physical activity and anxiety: a systematic review and meta-analysis of prospective cohort studies. Am J Prev Med 57, 545–556. https://doi.org/10.1016/j.amepre.2019.05.012.

McDowell, C.P., Dishman, R.K., Vancampfort, D., et al., 2018. Physical activity and generalized anxiety disorder: results from The Irish Longitudinal Study on Ageing (TILDA). Int J Epidemiol 47, 1443–1453. https://doi.org/10.1093/ije/dyy141.

Meyer, T.J., Miller, M.L., Metzger, R.L., et al., 1990. Development and validation of the Penn state worry questionnaire. Behav Res Ther 28, 487–495. https://doi.org/10.1016/0005-7967(90)90135-6.

Rebar, A.L., Stanton, R., Rosenbaum, S., 2017. Comorbidity of depression and anxiety in exercise research. Lancet 4 (7), 519. https://doi.org/10.1016/S2215-0366(17)30164-5.

Rosenthal, R., 1994. Parametric Measures of Effect Size. The Handbook of Research Synthesis. Russell Sage Foundation, New York, pp. 231–244.

Ruscio, A.M., Chiu, W.T., Roy-Byrne, P., et al., 2007. Broadening the definition of generalized anxiety disorder: effects on prevalence and associations with other disorders in the National Comorbidity Survey Replication. J Anxiety Disord 21, 662–676. https://doi.org/10.1016/j.janxdis.2006.10.004.

Rush, A.J., Trivedi, M.H., Ibrahim, H.M., et al., 2003. The 16-item quick inventory of depressive symptomatology (qids), clinician rating (qids-c), and self-report (qids-sr): a psychometric evaluation in patients with chronic major depression. Bio Psychiatr 54 (5), 573–583. https://doi.org/10.1016/S0006-3223(02)01866-8.

Schulz, K.F., Altman, D.G., Moher, D., 2010. CONSORT 2010 statement: updated guidelines for reporting parallel group randomised trials. BMC Med 8, 18. https://doi.org/10.1186/1741-7015-8-18.

Spielberger, C.D., Gorsuch, R.L., 1983. Manual for the state-trait anxiety inventory (form Y): self-evaluation questionnaire, 1st ed. Palo Alto:Consulting Psychologists Press.

United States Physical Activity Guidelines Advisory Committee, 2018. In: 2018 physical activity guidelines advisory committee scientific report. Department of Health and Human Services. Available here. https://health.gov/paguidelines/second-edition/report/. Accessed 03/12/2019.

Vlachopoulo, T., Tsalis, G., Fokas, K., et al., 2005. Efficacy of a weight training program as a treatment for chemically dependent. J Hum Mov Stud 49, 373–388.

Wolitzky-Taylor, K., Dour, H., Zinbarg, R., et al., 2014. Experiencing core symptoms of anxiety and unipolar mood disorders in late adolescence predicts disorder onset in early adulthood. Depress Anxiety 31, 207–213. https://doi.org/10.1002/da.22250.

World Health Organization, 2011. In: Global recommendations on physical activity for health. Available here: https://www.who.int/dietphysicalactivity/global-PA-recs-2010.pdf. Accessed 03/12/2019.

Zimmerman, M., Mattia, J.I., 2001. A self-report scale to help make psychiatric diagnoses: the psychiatric diagnostic screening questionnaire. Arch Gen Psychiatr 58, 787–794. https://doi.org/10.1001/archpsyc.58.8.787.

Zimmerman, M., Mattia, J.I., 2001. The psychiatric diagnostic screening questionnaire: development, reliability and validity. Compr Psychiatry 42, 175–189. https://doi.org/10.1053/comp.2001.23126.
