# Supplementary material for: Does physical activity-based intervention decrease repetitive negative thinking? A systematic review
Source: PLoS One. 2025 Apr 1;20(4):e0319806. doi: 10.1371/journal.pone.0319806 (PMC11960971; doi:10.1371/journal.pone.0319806)
Supplement: S1 File — https://doi.org/10.6084/m9.figshare.25711734. (ZIP) [file pone.0319806.s001.zip › supporting information/paper file/Craft 2015.pdf]

Psychology of Sport and Exercise 6 (2005) 151–171

Psychology
OF SPORT AND EXERCISE

www.elsevier.com/locate/psychsport

Exercise and clinical depression: examining two psychological mechanisms

Lynette L. Craft*

Department of Kinesiology, Michigan State University, East Lansing, MI,USA

Received 17 February 2003; received in revised form 1 June 2003; accepted 28 November 2003

Available online 28 January 2004

Abstract

Objectives: To examine the antidepressant effects of exercise, and two previously proposed psychological mechanisms for this effect: self-efficacy and distraction.

Method: A quasi-experimental design was utilized with participants ($N = 19$) self-selecting to either a control or a 9-week exercise group. Dependent variables included severity of depression, coping self-efficacy, rumination, and distraction and were measured at study entry, 3 and 9 weeks later.

Results: Exercisers reported lower depression scores than controls at Week 3 [9.3 (4.8) vs. 24.7 (12.0), $p < 0.05$] and Week 9 [6.4 (5.4) vs. 21.8 (16.4), $p < 0.05$]. Coping self-efficacy was higher among exercisers than controls at Week 3 [7.3 (0.9) vs. 5.2 (1.3), $p < 0.05$] and Week 9 [7.5 (0.9) vs. 5.3 (1.8), $p < 0.05$]. Exercisers ruminated less than controls at Week 3 [38.9 (1.8) vs. 46.5 (8.2), $p < 0.05$] and at Week 9 [33.9 (1.2) vs. 44.0 (10.9), $p < 0.05$]. Distraction was higher for exercisers at Week 3 [28.6 (1.2) vs. 23.6 (3.0), $p < 0.05$] but not at Week 9 [27.7 (2.1) vs. 23.7 (5.3), $p > 0.05$]. Partial correlations, controlling for baseline values of the independent variables, were conducted and a significant negative relationship between coping self-efficacy and depression was found at both Weeks 3 and 9.

Conclusions: Exercise was associated with a reduction in the symptoms of depression. Support was found for coping self-efficacy as a potential mechanism; however, there was limited support for distraction as a potential mechanism.

© 2003 Elsevier Ltd. All rights reserved.

Keywords: Mental health; Women; Physical activity

* Division of Psychiatry, Boston University, School of Medicine, Room M-619, 85 E. Newton Street, Boston, MA 02118, USA. Tel.: +1-617-414-1938; fax: +1-617-414-1937.

E-mail address: craftlyn@bu.edu

1469-0292/$ - see front matter © 2003 Elsevier Ltd. All rights reserved.
doi:10.1016/j.psychsport.2003.11.003

Introduction

It is estimated that clinical depression affects roughly 9.5% of the United States (US) adult population each year (National Institutes of Mental Health, 2001). Depression is characterized by a variety of symptoms including feelings of sadness, irritability, changes in sleep and appetite, feelings of worthlessness, loss of pleasure from enjoyable activities, and psychomotor retardation (American Psychiatric Association, 1994). Depression is twice as prevalent in females as males and the risk of a recurrence can be as high as 50–90% (Preskorn, 1999; Stahl, 1996). This chronic illness is very costly to the US health care system and each year over 40 billion dollars are spent on lost productivity and medical treatment related to depression (Zerihun, 2001).

Traditional treatments for clinical depression have primarily included psychotherapy and pharmacological interventions (Johnson & Miller, 1994; Preskorn, 1999). Unfortunately, these therapies are not always effective and, for some, may have side effects (Byrne & Byrne, 1993; Leith, 1994; Mutrie, 2000; Raglin, 1990; Stahl, 1996). Furthermore, health care changes in the US have led to time constraints for therapy and limits in payment for mental health services (Mirin & Sederer, 1994). Therefore, practitioners and researchers have begun to examine possible alternatives in the treatment of this disorder.

Exercise has been proposed as one plausible adjunct or alternative to the traditional treatments for depression. The first studies on exercise and depression were cross-sectional, comparing the physical activity and physical capacity levels of depressed and non-depressed individuals (Martinsen, Strand, Paulsson, & Kaggestad, 1989; Morgan, 1969, 1970). Exercise intervention studies followed, examining the efficacy of exercise in alleviating depression (e.g. Brown, Welsh, Labbe, Vitulli, & Kulkarni, 1992; Dimeo, Baurer, Varahm, Proest, & Halter, 2001; Doyne, Chambless, & Beutler, 1983; McNeil, LeBlanc, & Joyner, 1991; Veale et al., 1992). Researchers have investigated whether certain types of exercise are more effective in alleviating depression than others (Doyne et al., 1987; Martinsen, Hoffart, & Solberg, 1989; Singh, Clements, & Fiatarone, 1997). Exercise has also been compared to more traditional treatments such as psychotherapy, behavioral modification, and medication (Babyak et al., 2000; Blumenthal et al., 1999; Greist et al., 1979; Klein et al., 1985). Meta-analytic studies suggest that chronic exercise is effective in alleviating symptoms of depression, that exercise is as effective as most traditional treatments, that exercise is effective across genders, and that mode, frequency, and intensity of exercise do not appear to moderate the effect (Craft & Landers, 1998; North, McCullagh, & Tran, 1990).

While the relationship between exercise and a reduction in depression is a consistent finding, this research has been fraught with methodological problems that need to be considered. For example, many studies have utilized small samples or failed to employ control groups. Further, many studies have not used random assignment of subjects or intention to treat analyses. While these methodological limitations can be difficult to avoid, they may impact the generalizability and strength of study findings. Previous research has found that studies employing strict methodological control (i.e. randomized control trials) generally produce effects that are quite similar to less well controlled studies (Craft & Landers, 1998; Lawlor & Hopker, 2001). This may indicate that study quality is not an important moderator of the effect. However, others contend that the similar effect sizes found between clinical trials less well controlled studies may actually represent the fact that the benefit of randomization was mediated by other methodological flaws such as lack of adequate concealment to treatment group and lack of intention to treat analysis (Lawlor & Hopker, 2001). Therefore, while the research on this

relationship appears very positive, findings should be interpreted with these methodological considerations in mind.

Mechanisms for the antidepressant effects of exercise

There is general support that a relationship between exercise involvement and a reduction in depression exists; however, the mechanisms by which exercise exerts its effect have not been studied extensively and are not well understood. Nonetheless, there are researchers who contend that exercise causes a reduction in depression (Mutrie, 2000), while others argue that more information is needed before a causal association can be confirmed (Landers & Arent, 2001). Research to date has not extensively examined the plausible mechanisms suggested in the literature. A better understanding of the mechanisms behind the antidepressant effects of exercise would not only provide additional insight into a possible causal association, but should also be helpful in the future use of exercise as an adjunct therapy in the treatment of depression. Therefore, studies are needed to examine the potential physiological and psychological mechanisms (Dunn, Trivedi, & O'Neil, 2001; Fox, 1999; Landers & Arent, 2001; Leith, 1994). Two psychological explanations that have received attention in the literature and warrant further examination are self-efficacy and the concept of distraction. These two explanations are based on existing theories of depression and conceptually appear to be plausible mechanisms.

Self-efficacy

Self-efficacy, or the level of confidence one feels to meet the challenge at hand, has been proposed as one mechanism by which exercise may help reduce symptoms of clinical depression. Self-efficacy refers to the belief that one possesses the necessary skills to complete a task as well as the confidence that the task can actually be completed with the desired outcome obtained. Efficacy beliefs direct choice of behavior, goals that are chosen, persistence at a task, motivation and effort, expectations of success, and how one responds affectively (Bandura, 1997). Bandura (1997) contends that healthy individuals are able to regulate goal directed behaviors and are also able to use regulatory strategies for their thoughts, feelings, and emotions. This idea of self-regulation has led to the investigation of the concept of coping self-efficacy. People's beliefs about their ability to control a stressful situation and regulate their response to the situation is a primary determinant of how they will respond. A low sense of self-control or coping efficacy can cause people to approach situations with anxiety and with little confidence to obtain the desired outcome (Bandura, 1997).

Bandura (1997), utilizing self-efficacy theory, describes how the low sense of efficacy of those with depression often leads to negative self-evaluations, negative ruminations, and faulty styles of thinking. Further, depressed people often blame themselves for negative life events and this negative explanatory style can worsen and prolong their depression (Peterson & Seligman, 1984). Individuals with low efficacy to stop or restructure negative thoughts have also been shown to have higher levels of depression (Kavanagh & Wilson, 1989).

Several different factors influence the development of efficacy beliefs (Bandura, 1997). However, the best source of efficacy information appears to come from mastery experiences. When an

individual can repeatedly derive a course of action, follow a plan, and meet the desired outcome in the face of changing situations and obstacles then he or she is likely to feel highly efficacious. On the contrary, experiencing repeated failures at mastery attempts can lead to decreased feelings of self-efficacy.

Exercise may provide an effective mode to enhance efficacy beliefs based on its ability to provide a meaningful mastery experience. According to Bandura (1997), in order for an intervention to lead to enhanced coping self-efficacy it must teach the individual how to self-monitor behaviors, set goals, and utilize social support to maintain the desired behaviors. Learning how to monitor exercise behaviors, set short and long-term exercise goals, and positive support from the exercise instructor and significant others can all contribute to feelings of mastery. Therefore, it is possible that the antidepressant properties of exercise are related to enhanced feelings of efficacy that result from this mastery experience.

Research examining the relationship between physical activity and self-efficacy has predominately focused on the enhancement of physical self-efficacy and efficacy to regulate exercise behaviors (Beniamini, Rubenstein, Zaichkowksy, & Crim, 1997; Ewart, Stewart, Gillilan, & Kelemen, 1986; McAuley, 1992; McAuley & Courneya, 1992; McAuley, Lox, & Duncan, 1993). The relationship between exercise and self-efficacy in depressed patients has not been studied extensively and findings have been equivocal (Brown et al., 1992; Singh et al., 1997). Furthermore, generalized feelings of efficacy rather than coping self-efficacy have been the focus of the research to date. Enhanced feelings of coping have been associated with exercise participation in anxious adults (Steptoe, Edwards, Moses, & Mathews, 1989), however, as mentioned previously, this relationship has not been examined with the clinically depressed. Therefore, the role that exercise can play in augmenting the coping self-efficacy of depressed patients needs further investigation.

Distraction

The concept of distraction has also been promoted as a possible explanation for the antidepressant effects of exercise. Some researchers (Bahrke & Morgan, 1978; Gleser & Mendleberg, 1990; Johnsgard, 1989; Leith, 1994) have suggested that physical activity serves as a distraction from worries, anxiety, and depressing thoughts. A theory of depression that is conceptually related is the response styles theory (Nolen-Hoeksema, 1991). This theory discusses two different dispositional ways in which individuals typically respond to feelings of depression and how these differing responses can affect the severity and length of a depressive episode. Rumination is a response style that involves the tendency to passively and repeatedly focus on one's negative feelings and the consequences of those feelings. Distraction refers to a response style in which the individual busies oneself in an engaging activity (i.e. a hobby, work) in an attempt to focus on something other than the depressed mood.

Rumination is thought to have a negative influence on the course of depression. Rumination may lead to negative thoughts and attributions about the self, past, present, and future, which can contribute to a continued depressed mood (Lyubomirsky & Nolen-Hoeksema, 1995). Those who focus on their negative thoughts rather than engage in activities to solve their problems are at risk for prolonged bouts of depression (Nolen-Hoeksema, Parker, & Larson, 1994). Further, ruminating on

one's depression may prevent the individual from behaving in a manner that elicits positive reinforcement and enhances one's sense of control (Lyubomirsky & Nolen-Hoeksema, 1993). Without such opportunities, the depressed person may experience feelings of helplessness, lowered self-esteem, lowered expectancies for the future, and a lack of motivation (Nolen-Hoeksema, 1991). Research related to response styles theory has shown that women spend more time ruminating than men, that men tend to use distraction more than women, and that individuals who ruminate generally have more severe and longer lasting episodes of depression than those who use distraction as a response style (Just & Alloy, 1997; Morrow & Nolen-Hoeksema, 1990; Nolen-Hoeksema and Morrow, 1991; Nolen-Hoeksema, Morrow, & Fredrickson, 1993). Therefore, based on the current research, a distraction response style appears to be associated with a more positive outcome for those suffering from depression (Nolen-Hoeksema, 1991, 1998).

Nolen-Hoeksema (1991) cautions that not all activities are distracting. An effective distracting activity engages the individual and has a high probability of providing opportunities for positive reinforcement (Nolen-Hoeksema, 1991). The very nature of exercise makes it a potentially distracting activity worthy of investigation. When working out, people are often focused on training goals or attending to somatic changes such as their breathing, heart rate, fatigue, or sore muscles (Leith, 1994). Furthermore, exercise offers the opportunity for positive reinforcement as exercise goals are met, daily workouts become less painful and fatiguing, and significant others reward the individual for taking a proactive role in the management of her symptoms. Therefore, the antidepressant effects of exercise may result from the ability of exercise to provide periodic distraction from negative thoughts and feelings of depression. As mentioned previously, this temporary attenuation of depressed mood may allow for more effective problem-solving behaviors (Nolen-Hoeksema, 1991).

Response styles theory has not yet been tested with depressed participants who engage in an exercise program as a distracting activity. Exercise has been compared to other distracting activities such as relaxation, assertiveness training, health education, and social contact (Doyne et al., 1983; Klein et al., 1985; McNeil et al., 1991; Singh et al., 1997). Results of these studies have been inconclusive with exercise being more effective than some activities and similar to others in its ability to aid in the reduction of depression. This line of research, however, has only examined the overall effect of these activities on symptoms of depression. It is proposed in the present study that an exercise program may lead to an alteration in response style. Exercising individuals may spend less time in rumination and, instead, engage in distracting activities as a means to elevate their mood.

Therefore, the purpose of this study was to examine two previously proposed mechanisms for the antidepressant effects of exercise, self-efficacy and distraction. The relationships between self-efficacy, response style (rumination and distraction), exercise, and clinical depression were studied. The following hypotheses were proposed: (a) a chronic exercise intervention will be associated with a reduction in the symptoms of clinical depression, (b) depressed participants in an exercise training intervention will report increased levels of coping self-efficacy, (c) depressed individuals who engage in a chronic exercise intervention will report a reduced tendency to ruminate on their depression and an increased use of distraction across a 9-week intervention, and (d) obtained levels of coping self-efficacy, rumination, and distraction will be related to current levels of depression among exercise group participants.

Method

Participants and design

Prior to data collection, study approval was obtained from the Institutional Review Board. Further, participants in this study signed an informed consent. All women in this study were Caucasian and the average age of the participants was 43.21 years (SD = 13.23).

Initially, an experimental design with random assignment of participants to study groups was planned and all participants who met study inclusion criteria indicated their desire to be in the exercise intervention group. However, there were multiple problems with subject retention during the recruitment period. Therefore, a non-equivalent control group design (quasi-experimental) was utilized with participants assigned to their choice of either the control group or the exercise intervention group.

Subsequently, 10 women chose to participate in the control group and 11 women chose the exercise intervention group. There were two women who dropped out of the study, both in the exercise intervention group. These participants attended the first exercise session, which consisted primarily of information regarding stretching, monitoring heart rate, and familiarization with the exercise equipment. Neither of these women returned to the study after this initial session and neither of these women completed baseline questionnaire data. One participant stated that she was too busy to take part in the study and the other woman elected to enter psychotherapy and was therefore no longer eligible for the study. There were no further dropouts in either the control or exercise group, leaving a total of 10 in the control group and nine in the exercise group.

All women were suffering from physician diagnosed (e.g. DSM-IV, ICD-9) clinical depression. Further, participants were receiving pharmacological treatment for their depression (for at least 4 weeks prior to study entry) but were not receiving any additional psychological treatments. Participants reported current episodes of depression lasting a mean of 17.47 months (SD = 28.75). Further, they had been taking their current medications for a mean of 47.26 months (SD = 47.55). Participants were excluded if they were currently involved in a regular exercise program or if they had been involved in an exercise program at any time in the past year. That is, all participants were asked if they were currently (or at any time in the past year) involved in exercise activities consisting of at least 3 bouts per week, for at least 20–30 min per session, at a moderate intensity. A "brisk" walk was provided as an example of moderate intensity. Two participants indicated that they occasionally (approximately 1–2 times/month) took a leisurely walk but all others indicated no current (or in past year) physical activity. Participants were also deemed ineligible for the study if they were concurrently suffering from another mental illness, were currently undergoing any treatment other than pharmacological interventions for their depression, or if they had any physical illness that would contraindicate exercise.

Participants were recruited from area physicians, advertisements placed in local newspapers, and posted flyers. Participants were informed that the purpose of the study was to examine various treatments for clinical depression. All participants maintained normal contact with their physician and continued pharmacological treatment during the length of the study. Further, they were asked to report to the primary investigator any change in antidepressant medication or medication dosage during the course of the study. All participants who completed the 9-week study received $25.00 for their participation.

Questionnaires

Demographic

A questionnaire assessing a variety of variables such as ethnicity, age of onset of first depressive episode, number of hospitalizations for depression, current antidepressant medication, current physical health problems, etc. was administered to all participants at study entry.

Exercise program readiness questionnaire

Participants assigned to the exercise intervention group completed this 10-item questionnaire. This inventory assessed health status and risk factors (i.e. dizziness, history of heart attack, excessively overweight) that would indicate the need for medical evaluation prior to beginning an exercise program. Any woman who answered "yes" to at least one of the questions and all women over the age of 40 were required to consult with their physician and provide a physician's signature indicating the participant's suitability to engage in the moderate intensity exercise program.

Beck Depression Inventory-II (Beck, Steer, & Brown, 1996)

The BDI-II is a 21-item self-report inventory designed to assess the behavioral and cognitive symptoms of depression. Each item consists of several statements describing symptoms of depression. Participants were asked to circle the statement that best described their symptoms related to that particular aspect of depression (i.e. feelings of guilt, changes in sleep). The statements are numbered from "0" to "3", with higher numbers indicating more severe symptoms. Scores for responses are summed to obtain an overall score. The overall sum can range from 0 to 63, with higher scores indicating more severe depression. Beck et al. suggest the following cut score guidelines: scores of 0–13 indicate "minimal" depression, 14–19 indicate "mild" depression, 20–28 indicate "moderate" depression, and 29–63 indicate "severe" depression. This inventory has demonstrated acceptable internal consistency with the authors reporting Cronbach's alphas ranging from 0.92 to 0.93. Internal consistency for this study was examined using Cronbach's alpha and was deemed acceptable with $\alpha = 0.91$.

Depression Coping Self-Efficacy Scale (Perraud, 2000)

The DCSES is a 24-item inventory designed to assess the coping self-efficacy of depressed patients. The inventory asks participants to rate their confidence in their ability to engage in a variety of coping responses from 0 (not at all confident) to 10 (confident). The ratings for all items are summed and a mean score is calculated. This inventory has demonstrated acceptable internal consistency with the author reporting Cronbach alphas ranging from 0.93 to 0.96. Internal consistency was also acceptable for the DCSES in this study ($\alpha = 0.82$).

Responses style questionnaire (Nolen-Hoeksema & Morrow, 1991)

The RSQ assesses how an individual generally responds to feeling sad, down, or depressed. The inventory has two subscales: the Ruminative Response Scale, which has 22 items and assesses the tendency to respond to depression with self-focused behaviors and the Distractive Response Scale, which has 11 items and assesses the tendency to engage in distracting activities as a response to depression. The participant rates each response item on a scale from 1 (almost never) to 4 (almost always). An overall score for each subscale is obtained by summing responses. This inventory has demonstrated acceptable internal consistency (Ruminative Response Scale: $\alpha = 0.89$ and Distractive

Response Scale: $\alpha = 0.80$) (Nolen-Hoeksema & Morrow, 1991). In this study, the two subscales of the RSQ were found to be internally consistent (Ruminative Response Scale, $\alpha = 0.88$; Distractive Response Scale, $\alpha = 0.71$).

Rating of perceived exertion (Borg, 1982)

In an attempt to check the participants' perception of the exercise intensity and as a means to instruct participants on an accepted method of self-monitoring exercise intensity, participants rated their perception of effort exerted during exercise using Borg's Rating of Perceived Exertion (RPE) scale. This scale ranges from the numbers six to 20 with adjectives such as "very light", "hard", and "very, very hard" anchored along the numerical scale. Participants were asked to select the number that best described the intensity at which they felt they were working.

Procedure

All participants completed the demographic questionnaire, BDI-II, DCSES, and RSQ at study entry, prior to the beginning of the exercise intervention. A 9-week intervention was planned based upon the results of the Craft and Landers' (1998) meta-analysis which indicated that exercise interventions of 9-weeks or longer were most effective in alleviating symptoms of depression. Therefore, participants in the control group were followed for 9 weeks. Members of this group completed the BDI-II, the DCSES, and the RSQ again after 3 and 9 weeks. These three questionnaires were counterbalanced in terms of order of administration over the three time periods. Questionnaires were mailed to participants, which allowed them to complete the inventories privately and conveniently. Individuals in this group were instructed not to begin an exercise program during the 9 weeks of the study.

Those in the exercise group began an exercise intervention consisting of 3 days per week of moderate intensity physical activity. Participants exercised in groups of 2–4 twice per week, in a laboratory setting, and also completed one home-based exercise session per week on their own. Laboratory based sessions were conducted in an exercise physiology instructional classroom space at a Midwestern University in the USA. The room was well lit, contained several treadmills and cycle ergometers, ample free space for stretching, and a restroom/changing area with lockers. Participants were also able to park free of charge directly outside the facility.

Each exercise session consisted of a brief (5 min) session of stretching followed by exercise on stationary cycle ergometers and treadmills. The first week of the study consisted of familiarizing participants with stretching techniques, monitoring heart rate via radial or carotid pulse, and how to properly use the exercise equipment. At study entry, based upon self-report, the participants were currently physically inactive. As such, Weeks 2–3 of the study were conducted at a "comfortable" pace for a total of 20 min. For the remaining 6 weeks of the study, participants were asked to ride or walk at 50–75% of HRR for the 30-min bout with heart rate monitored throughout to ensure the appropriate exercise intensity was maintained for the entire bout. The exercise bout was followed by each participant rating her perceived exertion for the exercise session and a brief (5 min) cool down period.

For the home-based exercise sessions, participants were free to engage in whatever type of exercise activity they chose. Aerobics video-tapes were provided to participants to assist them in their home based session. Participants were instructed to follow the general exercise protocol of stretching, monitoring heart rate throughout exercise, cool-down, post-exercise rating of perceived exertion, stretch,

etc. Each week, participants reported to the investigator the date of her home based session, the type of activity performed, the length of time engaged in the activity, and the RPE associated with the exercise bout.

This intervention lasted 9 weeks with the participants completing the previously described questionnaire battery (BDI-II, DCSES, RSQ) at study entry, 3, and 9 weeks. These questionnaires were counterbalanced in terms of order of administration across the three time periods. The participants in the exercise group also completed the questionnaires at home and then returned them to the study investigator.

The researcher attempted to facilitate a mastery experience during this 9-week exercise intervention. During Weeks 1–3, the researcher guided the participants through their stretching, assisted them in monitoring their heart rates, provided them with a target heart rate range based on HRR, helped them regulate the intensity of their exercise session, etc. During Weeks 4–6, the participants took a more active role in the process. They stretched on their own, monitored their own heart rate, and attempted to adjust their exercise intensity to remain within their target heart rate range. They were also given information on how to calculate their target heart rate range. During the final 3 weeks of the intervention, the participants were responsible for the entire exercise session. They stretched on their own, computed their target heart rate range, monitored their heart rates and exercise intensity, completed the 30-min exercise bout, and cool down period without assistance from the researcher. The researcher was present to answer questions but encouraged the participants to complete the workout session on their own. Furthermore, having the participants exercise one day per week on their own should have assisted in the development of a mastery experience.

Analyses

Descriptive statistics were computed for subject characteristics and study variables. To check for potential group (i.e. control, exercise) differences at study entry, subject characteristics were examined using student's *t* tests for continuous variables and Chi square analyses for categorical variables. A one-way analysis of variance (ANOVA) was conducted to check for pre-existing group differences on study dependent variables (depression score, coping self-efficacy, and response style).

To test the first three study hypotheses, four separate repeated measures multivariate analysis of variance (MANOVAs) were utilized to examine group (control, exercise) differences across time on BDI-II (depression), DCSES (coping self-efficacy), and RSQ (rumination and distraction). Follow-up univariate analysis of variance (ANOVA) was utilized to determine group differences at each time point (i.e. whether the exercise or control group had higher mean scores). Within subjects repeated measures MANOVA was then conducted to determine at what time points these group differences occurred (i.e. if change occurred from study entry to Week 3 or from Week 3 to Week 9). For each analysis, tests of assumptions for multivariate analyses were met: normality, homogeneity of variance-covariance matrices (Box's M test), linearity, and sphericity (Greenhouse-Geisser and Huyon-Feldt). Effect sizes (Cohen's *d*) were also calculated for dependent variables at Week 3 and Week 9 using a pooled standard deviation as described by Hedges and Olkin (1985).

To test the fourth hypothesis that coping self-efficacy, rumination, and distraction would be related to depression score, partial correlations were calculated between independent variables and depression

score (dependent variable) at Week 3 and Week 9 controlling for baseline levels of each independent variable at each time point.

Results

At study entry, the two groups did not differ significantly ($p > 0.05$) on age, marital status, level of education, age that depression was first diagnosed, numbers of prior hospitalizations for depression, severity of depression, or type of antidepressant medication. Subject characteristics are presented in Table 1. Overall, adherence to the exercise program was good among the members of the exercise group. Most participants who missed sessions scheduled make-up sessions in order to complete the study protocol of 3 sessions total per week. Participants in the exercise group completed a mean of 23.3 of the possible 27.0 sessions. This was 86.3% of the scheduled sessions.

Scores for dependent variables (severity of depression, coping self-efficacy, and use of rumination and distraction) at study entry were examined using ANOVA and results showed that the two groups did not differ significantly [BDI-II: $F(1, 18) = 0.55$, $p = 0.47$; DCSES: $F(1, 18) = 2.28$, $p = 0.15$; rumination: $F(1, 18) = 1.03$, $p = 0.33$; distraction: $F(1, 18) = 3.45$, $p = 0.08$]. Means and standard deviations for these study variables for all time points are presented in Table 2.

To test the first hypothesis that a chronic exercise intervention would be associated with a reduction in depression, a Group(control, exercise) × Time (study entry, 3 weeks, 9 weeks) repeated measures MANOVA was conducted. The Group × Time interaction was significant (Wilks' $\lambda = 0.66$, $F(2, 16) = 4.2$, $p < 0.05$) as was the within subjects main effect for Time (Wilks' $\lambda = 0.56$,

Table 1
Subject characteristics

	Control ($N = 10$) mean (SD)	Exercise ($N = 9$) mean (SD)	t or X^2	p
Age (years)	42.9 (13.3)	43.6 (13.9)	$t = -0.11$	0.92
Age first diagnosed (years)	28.8 (12.5)	31.1 (13.3)	$t = -0.39$	0.70
Number of prior hospitalizations for depression	0.1 (0.3)	0.6 (1.1)	$t = -1.17$	0.24
BDI-II score (Severity of depression)	24.9 (12.6)	21.2 (8.4)	$t = 0.74$	0.47
Marital status			$X^2 = 2.04$	0.57
Single	3.0	3.0		
Married	5.0	6.0		
Widowed	1.0	0.0		
Separated	1.0	0.0		
Highest level of education achieved			$X^2 = 2.25$	0.32
High School	4.0	1.0		
College	3.0	5.0		
Graduate School	3.0	3.0		
Type of antidepressant medication			$X^2 = 0.57$	0.75
SSRIs	6.0	5.0		
Atypical SSRIs	5.0	5.0		

Table 2
Means and standard deviations across time on BDI-II, DCSES, and RSQ

	Study entry		3 weeks		9 weeks	
	Control	Exercise	Control	Exercise	Control	Exercise
BDI-II	24.9 (12.6)	21.2 (8.4)	24.7 (12.0)<br>ES = −0.02	9.3 (4.8)<br>ES = −1.42	21.8 (16.4)<br>ES = −0.24	6.4 (5.4)<br>ES = −0.60
DCSES	6.0 (1.2)	6.8 (1.0)	5.2 (1.3)<br>ES = −0.67	7.3 (0.9)<br>ES = 0.50	5.3 (1.8)<br>ES = 0.08	7.5 (0.9)<br>ES = 0.22
Rumination	48.0 (9.1)	43.8 (9.0)	46.5 (8.2)<br>ES = −0.16	38.9 (1.8)<br>ES = 0.54	44.0 (10.9)<br>ES = −0.30	33.9 (1.2)<br>ES = −2.8
Distraction	23.4 (3.1)	26.6 (4.3)	23.6 (3.0)<br>ES = 0.06	28.6 (1.2)<br>ES = 0.47	23.7 (5.3)<br>ES = 0.03	27.7 (2.1)<br>ES = −0.75

$F(2, 16) = 5.5$, $p < 0.05$). The between subjects main effect for Time was also significant, $F(1, 17) = 7.0$, $p < 0.05$. Follow-up tests for the interaction using univariate ANOVA indicated that the exercise group was less depressed than the control group at 3 weeks, $F(1, 18) = 12.8$, $p < 0.01$, and at 9 weeks, $F(1, 18) = 7.1$, $p < 0.05$. Within subjects repeated measures MANOVA indicated that the control group did not significantly reduce their depression scores from study entry to 3 weeks, Wilks' $\lambda = 0.10$, $F(1, 9) = 0.01$, $p > 0.05$, or from 3 weeks to 9 weeks, Wilks' $\lambda = 0.92$, $F(1, 9) = 0.80$, $p > 0.05$. Further, the exercise group had a significant reduction in depression from study entry to 3 weeks, Wilks' $\lambda = 0.41$, $F(1, 8) = 11.4$, $p < 0.01$, but no further reduction from 3 weeks to 9 weeks, Wilks' $\lambda = 0.72$, $F(1, 8) = 3.1$, $p > 0.05$. This indicates that exercise was associated with a reduction in depression by 3 weeks time and this reduction in depression in the exercise group was maintained to the end of the 9-week study. Means, standard deviations, and effect sizes for depression scores across time are presented in Table 2. Fig. 1 represents the significant Group × Time interaction.

The second hypothesis predicted that coping self-efficacy would increase across time in members of the exercise intervention. Repeated measures MANOVA, using coping self-efficacy as the dependent variable, showed that there was a significant Group × Time interaction [Wilks' $\lambda = 0.50$, $F(2, 16) = 8.2$, $p < 0.01$]. The within subjects main effect for Time was not significant, Wilks' $\lambda = 0.90$, $F(2, 16) = 0.93$, $p > 0.05$. The between subjects main effect for Group was also significant, $F(1, 17) = 17.2$, $p < 0.01$. Results of follow-up univariate ANOVA for the interaction indicated that the exercise group had higher coping efficacy at 3 weeks [$F(1, 18) = 16.6$, $p < 0.01$] as well as at 9 weeks [$F(1, 18) = 10.1$, $p < 0.01$]. Within subjects repeated measures MANOVA indicated that both the control group and the exercise group had a significant change in efficacy score from study entry to 3 weeks [control group: Wilks' $\lambda = 0.52$, $F(1, 9) = 8.5$, $p < 0.05$; exercise group: Wilks' $\lambda = 0.60$, $F(1, 8) = 5.3$, $p < 0.05$] but neither group showed further improvement from 3 weeks to 9 weeks [control group: Wilks' $\lambda = 0.10$, $F(1, 9) = 0.02$, $p > 0.05$; exercise group: Wilks' $\lambda = 0.96$, $F(1, 8) = 0.33$, $p > 0.05$]. The two groups both demonstrated changes in efficacy score across the 9-week study. The exercise group increased their self-efficacy score, while efficacy decreased in the control group. This may have contributed to the significant Group X Time interaction. However, those in the exercise intervention showed significantly greater increases in coping self-efficacy than those in the control group. This increased efficacy emerged by 3 weeks time and was maintained throughout

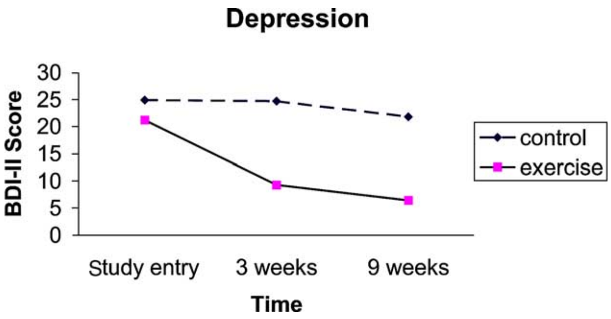

Fig. 1. Group × Time interaction for BDI-II score.

the remainder of the study. Means, standard deviations, and effect sizes across time for coping self-efficacy are presented in Table 2. Fig. 2 represents the significant Group × Time interaction.

The third hypothesis predicted that those in the exercise intervention would report a decreased tendency to use ruminative strategies and an increased use of distraction techniques across time. Repeated measures MANOVA was first conducted with rumination as the dependent variable. The Group × Time interaction was not significant [Wilks' $\lambda = 0.89$, $F(2, 16) = 1.0$, $p > 0.05$], but the main effect for time was significant [Wilks' $\lambda = 0.56$, $F(2, 16) = 6.3$, $p < 0.01$]. The test of between subjects main effect for Group was also significant [$F(1, 17) = 4.9$, $p < 0.05$]. These findings suggest that, regardless of group (control, exercise), all participants decreased their use of ruminative strategies across time. Because group means appeared different and low statistical power was suspected for the non-significant Group × Time interaction, a follow-up univariate ANOVA was conducted. Results showed that the exercise group had significantly lower scores for use of rumination than the control group at 3 weeks [$F(1, 18) = 5.5$, $p < 0.05$] and at 9 weeks [$F(1, 18) = 7.0$, $p < 0.001$]. Means, standard deviations, and effect sizes across time are presented in Table 2.

To determine whether the use of distraction increased across time more in the exercise group than in the control group, a repeated measures MANOVA using distraction as the dependent variable was conducted. The Group × Time interaction was not significant [Wilks' $\lambda = 0.93$, $F(2, 18) = 0.61$, $p > 0.05$] and neither was the main effect for Time [Wilks' $\lambda = 0.90$, $F(2, 18) = 0.88$, $p > 0.05$]. The between subjects main effect for Group was significant [$F(1, 17) = 7.8$, $p < 0.05$]. These findings suggest that there was not an increase in use of distraction across the 9 weeks of the study for either

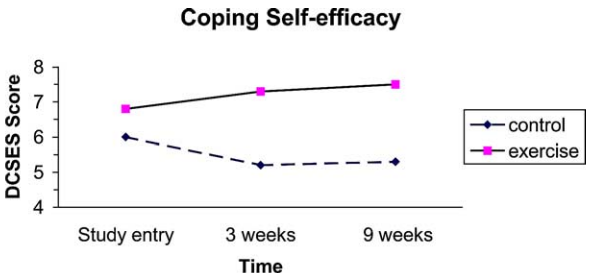

Fig. 2. Group × Time interaction for DCSES score.

Table 3
Partial correlations among IVs and DV at Week 3 and Week 9 controlling for baseline values of IV

Independent variable	Depression Week 3	Week 9
Coping self-efficacy	−0.79*	−0.77*
Rumination	0.06	−0.02
Distraction	−0.36	−0.61

* Correlation significant at $p < 0.05$.

the control group or the exercise group. Again, because the group means appeared different and low power was suspected for the non-significant Group × Time interaction, univariate ANOVA was conducted for each time point to determine if the groups were different. Results indicated that exercise group members had a higher use of distraction techniques at 3 weeks [$F(1, 18) = 10.6, p < 0.01$] but not at 9 weeks [$F(1, 18) = 2.2, p > 0.05$]. Means, standard deviations, and effect sizes for distraction scores across time are presented in Table 2.

Next, to test the hypothesis predicting that levels of coping self-efficacy, rumination, and distraction would be related to current levels of depression, partial correlations were calculated among independent variables and the dependent variable (depression) at Week 3 and Week 9. After controlling for baseline values of each independent variable, the only significant correlations that emerged were between coping self-efficacy and depression at Week 3 ($r = -0.79$, $p < 0.05$) and Week 9 ($r = -0.77$, $p < 0.05$). Partial correlations are presented in Table 3.

Discussion

Overall effect of exercise on depression

The first hypothesis predicted that an exercise intervention would be associated with a reduction in depression among clinically depressed women. This hypothesis was supported by the data. All women began the study with "moderate" levels of depression based on their scores on the BDI-II. At the conclusion of the study, the women in the control group were still moderately depressed whereas the women in the exercise intervention group were only minimally depressed. Therefore, a moderate intensity (50–75% HRR) exercise program consisting of 3 exercise sessions per week was associated with a reduction in the symptoms of clinical depression. This finding mirrors the results of numerous previous studies demonstrating a reduction in depression following involvement in an exercise program (e.g. Blumenthal et al., 1999; Brown et al., 1992; Dimeo et al., 2001; Doyne et al., 1983; Martinsen et al., 1989; Martinsen, Medhus, & Sandvik, 1985). Furthermore, in light of the fact that the women in this study had been taking antidepressant medications for a mean of 47.3 months and likely reflect a sample of individuals with chronic depression that have not adequately responded to antidepressant therapy, such response to an exercise intervention is a notable result.

Participants in the exercise group experienced a significant reduction in their depression by the third week of the study. Ewart (1995) contends that one obstacle to understanding the mechanisms mediating

the relationship between exercise and depression is the insistence that mood changes following exercise must be driven by physiological changes such as maximal aerobic capacity, muscular strength, or heart rate. Several studies, such as this one, have found psychological benefits of exercise in short time periods (e.g. Brown et al., 1992; Doyne et al., 1987). Others have also shown small relationships between psychological benefits and physiological changes (Blumenthal et al., 1999; Doyne et al., 1987; Martinsen et al., 1989; Veale et al., 1992). However, physiological parameters were not assessed in this study and therefore cannot be ruled out as potential mediators of this relationship. Research indicates that physiological adaptations to training, such as decreased heart rate, reductions in catecholamine response, and attenuation of the slow component of peak O_2 uptake, can occur within the first 2–3 weeks of exercise training (Winder, Hagberg, Hickson, Ehsani, & McLane, 1978; Womack et al., 1995). Therefore, while the reduction in symptoms of depression occurred very rapidly and the focus of this study is on psychological mechanisms, physiological mechanisms remain plausible and should not be discredited.

Given the large effect size that emerged at 3 weeks for those in the exercise group, there is evidence to suggest that interventions of shorter length may be very effective in reducing symptoms of depression. While this is appealing given current concerns related to promoting interventions that are efficacious and cost-effective, it may be premature to recommend exercise programs of such short duration. It remains unclear whether 3 weeks are long enough for depressed individuals to adopt and maintain exercise involvement as a behavioral lifestyle change. Shorter duration interventions may be effective in reducing symptoms of depression; however, it is not known whether symptom reduction would persist if the individual failed to adhere to a regular exercise program.

Women in this study chose the group in which they would participate. As such, it is possible that women in the exercise group expected to experience improvements in their depression. Women in the control group had a qualitatively different experience than exercise group members and a placebo control group was not utilized. Therefore, while the findings related to reductions in depression are very encouraging and were sustained across the 9 weeks of the study, these results should be interpreted with this limitation in mind.

Exercise, coping self-efficacy, and depression

The second hypothesis examined one of the previously proposed psychological mechanisms, coping self-efficacy. It was predicted that the participants in the exercise intervention group would report increased levels of coping self-efficacy. It was also hypothesized that obtained levels of coping self-efficacy would be related to current levels of depression. Both hypotheses were supported by the data. Women in the exercise group had significantly higher coping self-efficacy by the third week of the study and this heightened level of efficacy was maintained throughout the remainder of the study.

The relationship between exercise and coping self-efficacy (rather than generalized feelings of efficacy or efficacy to engage in exercise) has not been previously tested in women suffering from clinical depression. However, research conducted on adults with anxiety disorders supports this finding. Steptoe and colleagues (1989) compared the perceived coping ability of anxious adults following involvement in either an aerobic exercise program or a placebo-control strength and flexibility program. The authors reported that their moderate intensity aerobic exercise program was associated with reductions in anxiety and increases in coping self-efficacy. These psychological benefits were not significantly related to initial fitness level, changes in fitness, or age.

There are several ways in which mastering an important health behavior such as exercise could lead to enhanced feelings to cope with one's depression. First, all the participants, based upon self-reported exercise behavior, were not exercising on a regular basis. Therefore, making the commitment to engage in exercise three times per week was a very proactive step in the maintenance of their physical health. Adherence to this program may have increased the women's awareness that committing themselves to the management of other health behaviors (e.g. proper nutrition, appropriate sleep schedules) could help control the symptoms of their depression. Second, the women were very unfamiliar with the "process" of engaging in an exercise session. During the study, they learned to stretch, monitor heart rate, use exercise equipment, etc. Again, learning and mastering new health-related skills may have given them the confidence necessary to learn to master new techniques to deal with their symptoms. Third, goal setting was promoted throughout the exercise program. As goals were achieved and new goals set, confidence to achieve desired outcomes likely increased. Goal setting may have become viewed as a beneficial self-regulatory strategy to manage depressive symptoms. Fourth, the exercise participants were required to complete one exercise session per week on their own at home. The ability to engage in exercise independent of the group and exercise instructor could have transferred to feelings of confidence to take a more self-managed approach to the control of depressive symptoms. Finally, as the participants began to notice their depression subsiding, they verbalized that their improved affect was due to their involvement in the exercise program. Again, this may have led to the belief that they could, in fact, do something behaviorally that would impact their symptoms and that they weren't simply "victims" of depression.

In this study, the strongest relationship that emerged during Weeks 3 and 9 for exercisers was between coping self-efficacy and depression scores (after controlling for baseline coping self-efficacy). Coping self-efficacy demonstrated a strong negative relationship with depression indicating that higher levels of efficacy were associated with lower levels of depression. While this does not provide definitive evidence that coping self-efficacy is the psychological mechanism by which exercise exerts a positive influence on depression, it does allude to that possibility and indicates that researchers should continue to study self-efficacy theory in relation to exercise and depression. This finding is in accordance with Bandura's (1997) contention that individuals who feel inefficacious to control their thoughts, feelings, emotions, and reactions are generally more depressed than those with high efficacy.

It can be argued that learning any new skill could lead to enhanced coping self-efficacy and a reduction in depression. Bandura (1997) suggests that mastery experiences that enhance one's sense of self-control are vital for enhancing feelings of coping self-efficacy. As stated previously, he argues that an intervention must teach the individual how to self-monitor behaviors, set goals, and utilize social support to maintain the desired behaviors if an increase in coping self-efficacy is to occur. Therefore, while learning some types of new skills (such as hobbies) may engage the individual in a "mastery" experience, they may not lead to an enhanced sense of self-control. Further, it is easy to conceive that mastering other "health related" skills (e.g. health education, nutrition, weight management) could also lead to an enhanced sense of self-control and ultimately a reduction in depression. However, that does not negate the potential role of mastery in the relationship between exercise involvement and symptoms of depression. Research is needed to determine if the mastery experience associated with starting an exercise program is superior in reducing symptoms of depression compared to the mastery of other health related behaviors that also enhance feelings of self-control.

Therefore, the findings from this study suggest that the enhancement of coping self-efficacy should be an important component in the treatment of depression. Involvement in an exercise program can

provided a meaningful mastery experience through which coping self-efficacy can be augmented. If exercise is used as an adjunct therapy in the treatment of this disorder, opportunities for mastery should be emphasized and positive feedback provided.

Exercise, response style, and depression

The third study hypothesis predicted that involvement in an exercise program would be associated with a decreased tendency to use rumination as a response style and an increased use of distraction techniques. Further, it was hypothesized that response style would be associated with symptoms of depression. These hypotheses were partially supported by the data. All women reported a reduced use of ruminative strategies across time, with the exercisers having significantly lower mean scores at Week 3 and at Week 9. Results also indicated that the exercise group members had a higher use of distraction techniques at Week 3 but not at Week 9. Therefore, exercise was associated with a reduction in the use of rumination across the 9-week study and an increase in the use of a distraction response style at Week 3. However, data did not support the prediction that a response style would be associated with depression among exercise group participants.

No previous studies have examined whether an exercise intervention is associated with a change in response style. Prior research has consistently found that rumination predicts the duration and severity of depressed mood, however, the findings for distraction have been mixed with distraction predicting severity of depression in some studies but not others (Nolen-Hoeksema, 1991). While a positive side effect of exercise may be a more beneficial response style, it does not appear that either rumination or distraction is the psychological mechanism explaining the antidepressant effects of exercise. There are several potential explanations for why response style did not predict depression in the exercise group. With respect to rumination, it is possible that other psychological constructs, such as coping-self-efficacy or social support, may buffer the effects of a ruminative response style in individuals who exercise. Thus, while the person may still have a tendency to use ruminative strategies, if exercise results in increased confidence and a supportive social network, the use of rumination may not have such a negative impact on his/her depression. Nolen-Hoeksema (1991) argues that social support may mediate the relationship between rumination and depression if the social support encourages the depressed individual to stop ruminating and start engaging in distracting activities. Therefore, it is conceivable that exercise group participants may provide this type of social support to each other and help attenuate the effects of rumination on depression. Future research should assess the perceptions of social support among participants.

As for distraction, it remains highly possible that distraction is occurring during exercise activities. Participants may have been temporarily distracted from their depression during each acute exercise bout. If so, that temporary distraction might be responsible for the transient mood elevation that occurred following exercise. Response styles theory predicts that exercise, if an effective distracter, would lead to a temporary elevation in mood which could provide time for more effective problem-solving (Nolen-Hoeksema et al., 1993). Therefore, while there was not strong evidence to support a change in response style as the mechanism for reductions in depression, distraction during each exercise bout cannot be ruled out.

It is also possible that an increased use of distracting activities is an important predictor of depression but that such changes were not measured adequately in this study. For example, perhaps exercise leads to an increased use of a variety of distracting activities such as hobbies or various

physical activities that were not assessed by the inventory. Or, possibly, a person chooses to use one type of activity that she enjoys as her primary distracting activity. These types of changes would not necessarily be reflected in her distraction score. As Nolen-Hoeksema (1991) discusses, research related to a distractive response style must next find a way to measure the variety of tasks an individual uses to distract him/herself and the degree to which the task demands cognitive involvement. A depressed person may utilize one or two tasks that require much concentration and attention. These would be effective distractors that lead to mood elevation but would not necessarily result in a noticeable change in score on the Distractive Response Style (DRS) subscale. Therefore, an alteration in the use of a distractive response style also remains a possible explanation for the antidepressant effects of exercise. Future researchers should examine whether participants experience distraction from their thoughts and worries during the exercise bout and whether exercise leads to a change in the type of distracting activities used or the amount of involvement invested in distracting activities. Previous research has only compared the effect of exercise to other distracting activities (Doyne et al., 1983; Klein et al., 1985; McNeil et al., 1991), and, as such, findings do not answer those types of questions.

Therefore, an exercise intervention program appears associated with a positive change in response style. If exercise is utilized as an adjunct therapy, participants should be discouraged from discussing their depression during an exercise bout and encouraged to focus on aspects of the program that might provide distraction. Activities such as monitoring heart rate and exercise intensity and engaging in social interaction with other participants should provide distraction from worrisome thoughts and feelings.

Limitations

There are several limitations to the current study that must be addressed. First, a quasi-experimental design was utilized with participants choosing to be in either the control or exercise group. Therefore, it is possible that these two groups were not equivalent at study entry. Analysis of subject characteristics and study dependent variables indicated that there were no significant differences between groups on variables deemed important. However, there may have been relevant subject characteristics, such as exercise stage of change (Prochaska & DeClemente, 1983), which differed between the two groups that were not assessed in this study.

Second, reductions in depression among exercise group members may have been a result of expectancy effects. Perhaps women in that group expected to feel better because they were engaging in an exercise program. This cannot be excluded as a potential cause for the reductions in depression. However, it seems unlikely that the other positive benefits of exercise that emerged (i.e. increases in coping self-efficacy, reductions in rumination) would have been expected by the participants.

Third, this study did not utilize a placebo-control group. Therefore, the positive benefits associated with the exercise program may have resulted from a “group” effect. That is, from interaction with the investigator or social support from other exercise group members. Previous studies that have utilized placebo-control groups have typically found that exercise is still superior in reducing symptoms of depression and not likely due to group effects (Brown et al., 1992; Doyne et al., 1983; Doyne et al., 1987; Singh et al., 1997). However, results of this study should be interpreted with this limitation in mind. Future research should compare exercise groups to other groups involved in “mastery” oriented activities.

While it was not the intent of this study to evaluate the long-term effects of an exercise intervention, longitudinal studies are needed to determine whether the positive results of this study are long lasting. Other researchers have reported continued exercise involvement and remission of depression during follow-up to exercise intervention studies (e.g. Babyak et al., 2000; Martinsen & Medhus, 1989; Sime, 1987). However, it remains unclear whether the changes in coping self-efficacy and response style that occurred in this study are lasting effects or a temporary result of exercise involvement during a 9-week program.

While some support was found for the psychological mechanisms investigated in this study, it is important to emphasize that these are not the only potential mechanisms for the relationship between exercise and depression. It is highly likely that there are biological, psychological, and sociological factors influencing this relationship. There may also be individual variation in the mechanisms or combination of mechanisms mediating this relationship (Fox, 1999). Further, exercise program characteristics (e.g. increase in physical fitness, goal achievement, social experiences) likely exert an influence and need further examination (Sonstroem, 1984). As such, this relationship may be best studied utilizing a biopsychosocial approach.

Finally, the small sample size of this study resulted in low statistical power. This could explain several of the non-significant findings. Therefore, before future researchers disregard the theories explored here, it is important to replicate these findings with larger samples. Due to low statistical power, the testing of mediational relationships via path analysis techniques was not possible. While the data provided mixed support for the potential mechanisms examined in this study, each of these mechanisms should be further tested using a larger sample and statistical analyses to address potential mediating relationships.

Conclusions

There were many important findings that resulted from this study. A moderate intensity exercise program was associated with a reduction in depression among chronically depressed women taking antidepressant medications. Further, data provided support for coping self-efficacy as a psychological mechanism for the antidepressant effects of exercise. It also appears that involvement in an exercise program is associated with positive changes in response style. That is, a reduced tendency to ruminate and increased use of a distractive response style. These changes in self-efficacy and response style tendency happened rather quickly (in the first 3 weeks of the study) and are consistent with the findings of other studies reporting such changes in these variables following relatively short interventions (e.g. Burns & Nolen-Hoeksema, 1991; Ozer & Bandura, 1991). Therefore, findings support the use of exercise as an adjunct therapy in the treatment of clinical depression and exercise program aspects related to mastery and distraction should be emphasized.

References

American Psychiatric Association (1994). *Diagnostic and statistical manual of mental disorders* (4th ed.). Washington, DC: American Psychiatric Association.

Babyak, M., Blumenthal, J. A., Herman, S., Khatri, P., Doraiswamy, M., Moore, K., Craighead, W. E., Baldewicz, T. T., & Krishnan, K. R. (2000). Exercise treatment for major depression: maintenance of therapeutic benefit at 10 months. *Psychosomatic Medicine*, *62*, 633–638.

Bahrke, M. S., & Morgan, W. P. (1978). Anxiety reduction following exercise and meditation. *Cognitive Therapy Research*, *2*, 323–333.

Bandura, A. (1997). *Self-efficacy: The exercise of control*. New York, NY: W.H. Freeman and Company.

Beck, A. T., Steer, R. A., & Brown, K. (1996). *Beck depression inventory* (2nd ed.). SanAntonio, TX: Harcourt Brace.

Beniamini, Y., Rubenstein, J. J., Zaichkowsky, L. D., & Crim, M. C. (1997). Effects of high-intensity strength training on quality-of-life parameters in cardiac rehabilitation patients. *American Journal of Cardiology*, *80*, 841–846.

Blumenthal, J. A., Babyak, M. A., Moore, K. A., Craighead, W. E., Herman, S., Khatri, P., Waugh, R., Napolitano, M. A., Forman, L. M., Appelbaum, M., Doraiswamy, M., & Krishnan, K. R. (1999). Effects of exercise training on older patients with major depression. *Archives of Internal Medicine*, *159*, 2349–2356.

Borg, G. (1982). The rating of perceived exertion scale. *Medicine and Science in Sports and Exercise*, *14*, 377–387.

Brown, S. W., Welsh, M. C., Labbe, E. E., Vitulli, W. F., & Kulkarni, P. (1992). Aerobic exercise in the psychological treatment of adolescents. *Perceptual and Motor Skills*, *74*, 555–560.

Byrne, A., & Byrne, D. G. (1993). The effect of exercise on depression, anxiety and other mood states: a review. *Journal of Psychosomatic Research*, *37*, 565–574.

Burns, D., & Nolen-Hoeksema, S. K. (1991). Coping styles, homework assignments and the effectiveness of cognitive-behavioral therapy. *Journal of Consulting and Clinical Psychology*, *59*, 305–311.

Craft, L. L., & Landers, D. M. (1998). The effect of exercise on clinical depression and depression resulting from mental illness: a meta-analysis. *Journal of Sport and Exercise Psychology*, *20*, 339–357.

Dimeo, F., Baurer, M., Varahram, I., Proest, G., & Halter, U. (2001). Benefits from aerobic exercise in patients with major depression: a pilot study. *British Journal of Sports Medicine*, *35*, 114–117.

Doyne, E. J., Chambless, D. L., & Beutler, L. E. (1983). Aerobic exercise as a treatment for depression in women. *Behavior Therapy*, *14*, 434–440.

Doyne, E. J., Ossip-Klein, D. J., Bowman, E. D., Osborn, K. M., McDougall-Wilson, I. B., & Neimeyer, R. A. (1987). Running versus weight lifting in the treatment of depression. *Journal of Consulting and Clinical Psychology*, *55*, 748–754.

Dunn, A. L., Trivedi, M. H., & O'Neil, H. A. (2001). Physical activity dose-response effects on outcomes of depression and anxiety. *Medicine and Science in Sports and Exercise*, *33*, s587–s597.

Ewart, C. K. (1995). Self-efficacy and recovery from heart attack: implications for a social cognitive analysis of exercise and emotion. In J. E. Maddux (Ed.), *Self-efficacy, adaptation, and adjustment: Theory, research, and application*. New York, NY: Plenium Press.

Ewart, C. K., Stewart, K. J., Gillilan, R. E., & Kelemen, M. H. (1986). Self-efficacy mediates strength gains during circuit weight training in men with coronary artery disease. *Medicine and Science in Sports and Exercise*, *18*, 531–540.

Fox, K. R. (1999). The influence of physical activity on mental well-being. *Public Health Nutrition*, *2*, 411–418.

Gleser, J., & Mendelberg, H. (1990). Exercise and sport in mental health: a review of the literature. *Israel Journal of Psychiatry and Related Sciences*, *27*, 99–112.

Greist, J. H., Klein, M. H., Eischens, R. R., Faris, J., Gurman, A. S., & Morgan, W. P. (1979). Running as treatment for depression. *Comprehensive Psychiatry*, *20*, 41–54.

Hedges, L. V., & Olkin, I. (1985). *Statistical methods for meta-analysis*. New York: Academic Press.

Johnson, L. D., & Miller, S. D. (1994). Modification of depression risk factors: a solution-focused approach. *Psychotherapy*, *31*, 244–253.

Johnsgard, K. W. (1989). *The exercise prescription for anxiety and depression*. New York: Plenum Publishing.

Just, N., & Alloy, L. B. (1997). The response styles theory of depression: tests and an extension of the theory. *Journal of Abnormal Psychology*, *106*, 221–229.

Kavanagh, D. J., & Wilson, P. H. (1989). Prediction of outcome with a group version of cognitive therapy for depression. *Behaviour Research and Therapy*, *27*, 333–347.

Klein, M. H., Greist, J. H., Gurman, A. S., Neimeyer, R. A., Lesser, D. P., Bushnell, N. J., & Smith, R. E. (1985). A comparative outcome study of group psychotherapy vs. exercise treatments for depression. *International Journal of Mental Health*, *13*, 148–177.

Landers, D. M., & Arent, S. M. (2001). Physical activity and mental health. In R. N. Singer, H. A. Hausenblaus, & C. M. Janelle (Eds.), *Handbook of sport psychology* (2nd ed.). New York, NY: John Wiley and Sons.

Lawlor, D. A., & Hopker, S. W. (2001). The effectiveness of exercise as an intervention in the management of depression: systematic review and meta-regression analysis of randomized controlled trials. *British Medical Journal*, *322*, 763–766.

Leith, L. M. (1994). *Foundations of exercise and mental health*. Morgantown, WV: Fitness Information Technology.

Lyubomirsky, S., & Nolen-Hoeksema, S. (1993). Self-perpetuating properties of depressive rumination. *Journal of Personality and Social Psychology*, *65*, 339–349.

Lyubomirsky, S., & Nolen-Hoeksema, S. (1995). Effects of self-focused rumination on negative thinking and interpersonal problem-solving. *Journal of Personality and Social Psychology*, *69*, 176–190.

Martinsen, E. W., Hoffart, A., & Solberg, O. (1989). Comparing aerobic and nonaerobic forms of exercise in the treatment of clinical depression: a randomized trial. *Comprehensive Psychiatry*, *30*, 324–331.

Martinsen, E. W., & Medhus, A. (1989). Exercise adherence and patients' evaluation of exercise in a comprehensive treatment programme for depression. *Nordic Journal of Psychiatry*, *43*, 521–529.

Martinsen, E. W., Medhus, A., & Sandvik, L. (1985). Effects of aerobic exercise on depression: a controlled study. *British Medical Journal*, *291*, 109.

Martinsen, E. W., Strand, J., Paulsson, G., & Kaggestad, J. (1989). Physical fitness levels in patients with anxiety and depressive disorders. *International Journal of Sports Medicine*, *10*, 58–61.

McAuley, E. (1992). Understanding exercise behavior: a self-efficacy perspective. In G. C. Roberts (Ed.), *Motivation in sport and exercise*. Champaign, IL: Human Kinetics.

McAuley, E., & Courneya, K. S. (1992). Self-efficacy relationships with affective and exertion responses to exercise. *Journal of Applied Social Psychology*, *22*, 312–326.

McAuley, E., Lox, C., & Duncan, T. E. (1993). Long-term maintenance of exercise, self-efficacy, and physiological change in older adults. *Journal of Gerontology: Psychological Sciences*, *48*, 218–224.

McNeil, J. K., LeBlanc, E. M., & Joyner, M. (1991). The effect of exercise on depressive symptoms in the moderately depressed elderly. *Psychology and Aging*, *6*, 487–488.

Mirin, S. M., & Sederer, L. I. (1994). Mental health care: current realities, future directions. *Psychiatric Quarterly*, *65*, 161–175.

Morgan, W. P. (1969). A pilot investigation of physical working capacity in depressed and nondepressed males. *Research Quarterly*, *40*, 859–861.

Morgan, W. P. (1970). Physical working capacity in depressed and non-depressed psychiatric females: a preliminary study. *American Corrective Therapy Journal*, *24*, 14–16.

Morrow, J., & Nolen-Hoeksema, S. (1990). Effects of responses to depression on the remediation of depressive affect. *Journal of Personality and Social Psychology*, *58*, 519–527.

Mutrie, N. (2000). The relationship between physical activity and clinically-defined depression. In S. J. H. Biddle, K. R. Fox, & S. H. Boutcher (Eds.), *Physical activity and psychological well-being*. London: Routledge.

National Institutes of Mental Health (2001). The numbers count: mental disorders in America. Retrieved December 20, 2001, from http://www.nimh.gov.

Nolen-Hoeksema, S. (1991). Responses to depression and their effects on the duration of depressive episodes. *Journal of Abnormal Psychology*, *100*, 569–582.

Nolen-Hoeksema, S. (1998). Ruminative coping with depression. In J. Heckhausen, & C. S. Dweck (Eds.), *Motivation and self-regulation across the life span*. New York, NY: Cambridge University Press.

Nolen-Hoeksema, S., & Morrow, J. (1991). A prospective study of depression and posttraumatic stress symptoms after a natural disaster: the 1989 Loma Prieta earthquake. *Journal of Personality and Social Psychology*, *61*, 115–121.

Nolen-Hoeksema, S., Morrow, J., & Fredrickson, B. L. (1993). Response styles and the duration of episodes of depressed mood. *Journal of Abnormal Psychology*, *102*, 20–28.

Nolen-Hoeksema, S., Parker, L., & Larson, J. (1994). Ruminative coping with depressed mood following loss. *Journal of Personality and Social Psychology*, *67*, 92–104.

North, T. C., McCullagh, P., & Tran, Z. V. (1990). Effects of exercise on depression. *Exercise and Sport Science Reviews*, *18*, 379–415.

Ozer, E. M., & Bandura, A. (1991). Mechanisms governing empowerment effects: a self-efficacy analysis. *Journal of Personality and Social Psychology*, *58*, 472–486.

Perraud, S. (2000). Development of the Depression Coping Self-Efficacy Scale (DCSES). *Archives of Psychiatric Nursing*, *14*, 276–284.

Peterson, C., & Seligman, M. E. P. (1984). Causal explanations as a risk factor for depression: theory and evidence. *Psychological Review*, *91*, 347–374.

Preskorn, S. H. (1999). *Outpatient management of depression: A guide for the practioner* (2nd ed.). Caddo, OK: Professional Communications, Inc.

Prochaske, J. O., & DiClemente, C. C. (1983). Stages and processes of self-change in smoking: towards an integrative model of change. *Journal of Consulting and Clinical Psychology*, *51*, 390–395.

Raglin, J. S. (1990). Exercise and mental health: beneficial and detrimental effects. *Sports Medicine*, *9*, 323–329.

Sime, W. E. (1987). Exercise in the treatment and prevention of depression. In Morgan, & Goldston (Eds.), *Exercise and mental health*. Washington, DC: Hemisphere.

Singh, N. A., Clements, K. M., & Fiatarone, M. A. (1997). A randomized controlled trial of progressive resistance training in depressed elders. *Journal of Gerontology*, *52*, M27–M35.

Sonstroem, R. J. (1984). Exercise and self-esteem. *Exercise and Sport Science Reviews*, *12*, 123–155.

Stahl, S. M. (1996). *Essential psychopharmacology: Neuroscientific basis and practical applications*. New York, NY: Cambridge University Press.

Steptoe, A., Edwards, S., Moses, J., & Mathews, A. (1989). The effects of exercise training on mood and perceived coping ability in anxious adults from the general population. *Journal of Psychosomatic Research*, *33*, 537–547.

Veale, D., LeFevre, K., Pantelis, C., deSouza, V., Mann, A., & Sargent, A. (1992). Aerobic exercise in the adjunctive treatment of depression: a randomized controlled trial. *Journal of the Royal Society of Medicine*, *85*, 541–544.

Winder, W. W., Hagberg, J. M., Hickson, R. C., Ehsani, A. A., & McLane, J. A. (1978). Time course of sympathoadrenal adaptation to endurance exercise training in man. *Journal of Applied Physiology*, *45*, 370–374.

Womack, C. J., Davis, S. E., Blumer, J. L., Barrett, E., Weltman, A. L., & Gaesser, G. A. (1995). Slow component of O_2 uptake during heavy exercise: adaptation to endurance training. *Journal of Applied Physiology*, *79*, 838–845.

Zerihun, M (2001). Depression drains workplace productivity. Retrieved October 31, 2001, from http://www.depressionnet.org.
