# Supplementary material for: Does physical activity-based intervention decrease repetitive negative thinking? A systematic review
Source: PLoS One. 2025 Apr 1;20(4):e0319806. doi: 10.1371/journal.pone.0319806 (PMC11960971; doi:10.1371/journal.pone.0319806)
Supplement: S1 File — https://doi.org/10.6084/m9.figshare.25711734. (ZIP) [file pone.0319806.s001.zip › supporting information/paper file/Herring 2017.pdf]

Accepted Manuscript

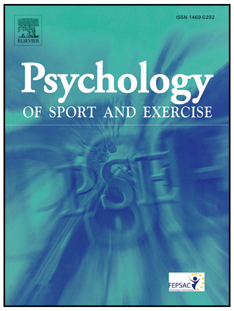

Acute exercise effects on worry, state anxiety, and feelings of energy and fatigue among young women with probable Generalized Anxiety Disorder: A pilot study

Matthew P. Herring, Mats Hallgren, Mark J. Campbell

PII: S1469-0292(16)30179-0

DOI: 10.1016/j.psychsport.2017.07.009

Reference: PSYSPO 1244

To appear in: *Psychology of Sport & Exercise*

Received Date: 3 October 2016

Revised Date: 12 June 2017

Accepted Date: 27 July 2017

Please cite this article as: Herring, M.P., Hallgren, M., Campbell, M.J., Acute exercise effects on worry, state anxiety, and feelings of energy and fatigue among young women with probable Generalized Anxiety Disorder: A pilot study, *Psychology of Sport & Exercise* (2017), doi: 10.1016/j.psychsport.2017.07.009.

This is a PDF file of an unedited manuscript that has been accepted for publication. As a service to our customers we are providing this early version of the manuscript. The manuscript will undergo copyediting, typesetting, and review of the resulting proof before it is published in its final form. Please note that during the production process errors may be discovered which could affect the content, and all legal disclaimers that apply to the journal pertain.

ACCEPTED MANUSCRIPT

Acute Exercise Effects on Worry, State Anxiety, and Feelings of Energy and Fatigue Among Young Women with Probable Generalized Anxiety Disorder: A Pilot Study

Matthew P. Herring,[a,b] Mats Hallgren,[c] and Mark J. Campbell[a]

[a]Department of Physical Education and Sport Sciences, University of Limerick, Ireland

[b]Health Research Institute, University of Limerick, Ireland

[c]Division of Epidemiology and Public Health Intervention Research (EPHIR), Department of Public Health Sciences, Karolinska Institutet, Solna 171 77, Sweden

Corresponding Author:
Matthew P. Herring, PhD
PESS 1045
Department of Physical Education and Sport Sciences
University of Limerick
Limerick, Ireland
Phone: +353 061 23 4762
Email: matthew.herring@ul.ie

Word Count: 3,507

ACCEPTED MANUSCRIPT

ACCEPTED MANUSCRIPT

Acute Exercise Effects on Worry, State Anxiety, and Feelings of Energy and Fatigue Among Young Women with Probable Generalized Anxiety Disorder: A Pilot Study

Date of Submission: June 12, 2017

Word Count: 3,507

ACCEPTED MANUSCRIPT

ACCEPTED MANUSCRIPT

Background

Little is known about the acute effects of exercise among individuals with clinical or subclinical Generalized Anxiety Disorder (GAD).

Purpose

Thus, this study examined worry, state anxiety, and feelings of energy and fatigue responses to acute aerobic exercise and quiet rest, and explored potential moderators of response among young adult women with worry scores indicative of GAD.

Methods

Seventeen young women with Penn State Worry Questionnaire scores ≥45 (60±8) completed 30-min treadmill running at 65%-85% heart rate reserve (%HRR) and 30-min seated quiet rest in counterbalanced order. Outcomes included worry, state anxiety, and feelings of energy and fatigue. Two condition X two time repeated measures ANOVA examined differences across condition and time. Hedges' *d* effect sizes (95%CI) were calculated to quantify and compare the magnitude of change. Independent-samples *t*-tests explored potential moderators of outcome response.

Results

Total exercise time was 35.8±3.4min with a mean 30.3±0.16 in-zone minutes (65%-85%HRR); participants exercised at ~72.9±0.03 %HRR (range 66%-79%). Compared with quiet rest, acute exercise significantly improved worry engagement, state anxiety, and feelings of energy and fatigue (all $p \leq 0.031$). Moderate-to-large (d=0.44 to 1.69) improvements in state anxiety and feelings of energy and fatigue were found. Exercise-induced reductions in worry engagement were significantly larger among non-high trait anxious participants. Compared to normal sleepers, quiet rest significantly increased feelings of fatigue among poor sleepers.

ACCEPTED MANUSCRIPT

ACCEPTED MANUSCRIPT

Conclusion

Findings provide initial support for the positive effects of acute aerobic exercise on worry, state anxiety, and feelings of energy and fatigue among young women with worry indicative of GAD.

Keywords: Acute exercise; Worry; Anxiety; Fatigue; Energy; Generalized Anxiety Disorder

ACCEPTED MANUSCRIPT

ACCEPTED MANUSCRIPT

Introduction

Among young adult women with Generalized Anxiety Disorder (GAD), evidence supports the effects of exercise training on clinical severity (Herring, Jacob, Suveg, Dishman, & O’Connor, 2012), worry, anxiety, and feelings of energy and fatigue, (Herring, Jacob, Suveg, & O’Connor, 2011), dimensions of sleep quality and quantity (Herring, Kline, & O'Connor, 2015), and health-related quality of life (Herring, Johnson, & O'Connor, 2016). Improved signs and symptoms of GAD, including anxiety, worry, and feelings of energy and fatigue, have been reported following as few as two weeks of exercise training involving the collective effects of three bouts of exercise (Herring et al., 2011). However, less is known about the effects of a single bout of exercise among individuals with GAD and, in particular, subclinical levels of GAD (i.e., elevated worry, the hallmark of GAD). Given that individuals who display elevated symptom scores are more likely to develop clinically significant psychopathology (Ruscio et al., 2007; Wolitzky□Taylor et al., 2014), investigating exercise effects on worry and other key outcomes in the symptom profile of GAD, including state anxiety and feelings of energy and fatigue, among individuals with emerging symptoms of GAD may be particularly important. However, the effects of a single bout of exercise among individuals with elevated worry scores indicative of GAD are not yet known.

GAD is more prevalent among women (Bandelow & Michaelis, 2015; Remes, Brayne, Linde, & Lafortune, 2016), and women have a higher likelihood of reporting GAD symptoms shown to be improved by exercise (i.e., fatigue, irritability, muscle tension, and somatic symptoms) (Herring et al., 2011; Steiner et al., 2005; Vesga-López et al., 2008). The limited available evidence of exercise effects among individuals with GAD has supported positive benefits for young women with GAD. Recent evidence also supported moderate-to-large

ACCEPTED MANUSCRIPT

ACCEPTED MANUSCRIPT

improvements in feelings of energy and fatigue, total mood disturbance, and state anxiety following acute exercise in young adult women (McDowell, Campbell, & Herring, 2016). Thus, the authors' reasoned that females with elevated worry indicative of GAD may be particularly likely to benefit from a single bout of vigorous intensity aerobic exercise.

Additionally, very little is known about potential moderators of response to acute exercise. Identifying predictors/correlates of outcome responses to acute exercise has implications for the future development of both acute and chronic exercise protocols. Thus, the objectives of this study were to: (1) test the effects of acute aerobic exercise compared to quiet rest on worry, state anxiety, and feelings of energy and fatigue, and, (2) explore potential moderators of response among young women with worry scores indicative of GAD. The authors hypothesized that, compared to quiet rest, an acute bout of aerobic exercise would significantly improve worry, state anxiety, and feelings of energy and fatigue among young women with worry indicative of GAD.

Methods

Design & Participants. The study protocol was approved by the University's Research Ethics Board. Prior to participation, interested potential participants provided written informed consent and completed a medical history screening questionnaire that included the Physical Activity Readiness Questionnaire. Seventeen young adult women, aged 20.8±1.4y, were recruited from the university and surrounding populations as part of ongoing recruitment for a series of acute exercise studies. Of 85 consecutively recruited individuals, 53 were included in a previously reported study using the same standard protocols (McDowell et al., 2016), seven did not provide adequate data to be included in the previously published study or the study reported here, and 8 potentially eligible male participants were excluded based on the current focus on female

ACCEPTED MANUSCRIPT

ACCEPTED MANUSCRIPT

participants with probable GAD. Based on power analysis performed with G*Power, the resulting sample size of 17 would provide >80% statistical power to detect differences in worry assuming a two-tailed α=0.05, a correlation between repeated measures of r=0.8, and a moderate effect of exercise on worry (f=0.23, d=0.46) based on previous evidence (Herring et al., 2012; McDowell et al., 2016). Potential participants were screened based on the Penn State Worry Questionnaire (Meyer, Miller, Metzger, & Borkovec, 1990). Inclusion criteria were : i) Penn State Worry Questionnaire (PSWQ) score ≥45, which has demonstrated high sensitivity and specificity as a cut-score to identify individuals with elevated worry indicative of GAD (Behar, Alcaine, Zuellig, & Borkovec, 2003); ii) age 18-35y; iii) no medical contraindication to safe participation in vigorous aerobic exercise; and, iv) no current pregnancy or lactation. Potential participants who met inclusion criteria were randomized using random number generation (www.randomizer.org) to complete two conditions in counterbalanced order at approximately the same time of day, with approximately 48h between conditions: i) 30-min of vigorous running on a treadmill, or ii) 30-min seated quiet rest, a well-established control condition in studies of acute exercise and mood (Ensari, Greenlee, Motl, & Petruzzello, 2015; McDowell et al., 2016).

Baseline Measures. Before testing on day 1, each participant completed electronic versions (www.surveymonkey.com) of a Seven Day Physical Activity Recall (Blair et al., 1985), the trait subscale of the State-Trait Anxiety Inventory, (STAI-Y2) (Spielberger, 1983), the Quick Inventory of Depressive Symptoms (QIDS) (Rush et al., 2003a), and the Pittsburgh Sleep Quality Index (PSQI) (Buysse, Reynolds, Monk, Berman, & Kupfer, 1989). Established cut-scores were used to classify high trait anxious status, depression status, and poor sleep status. STAI-Y2 scores >1SD above the age-related norm (~50) indicated high trait anxious status (Speilberger, Gorsuch, Lushene, Vagg, & Jacobs, 1983). QIDS scores ≥6 indicated depression

ACCEPTED MANUSCRIPT

(Rush et al., 2003b). PSQI scores >5 indicated poor sleep (Buysse, Reynolds III, Monk, Berman, & Kupfer, 1989).

Outcomes. Using laboratory desktop PCs, immediately before and 10 minutes following exercise or quiet rest participants completed electronic assessments (www.surveymonkey.com) of worry, state anxiety, and the intensity of feelings of energy and fatigue. This process required approximately 10-15 minutes for participants.

Worry. Worry was assessed using the 16-item PSWQ (Meyer et al., 1990). Each participant rated 16 statements using a 5-item Likert scale from 1 “Not at all typical of me” to 5 “Very typical of me;” item responses are summed after reverse scoring such that total score ranges from 16-80. Worry engagement (11 items worded in the direction of pathological worry) and absence of worry (5 items reverse-worded to combat acquiescence) subscales were also calculated and examined (Fresco, Heimberg, Mennin, & Turk, 2002; Meyer et al., 1990). The PSWQ demonstrated adequate internal consistency in the current sample (α=0.86), and correlations between repeated measures were r=0.90 and r=0.93 for exercise and quiet rest, respectively. Worry engagement demonstrated similar internal consistency (α=0.83), and correlations between repeated measures were r=0.97 and r=0.87 for exercise and quiet rest, respectively. However, internal consistency for absence of worry was poor (α=0.44), and correlations between repeated measures were low for both exercise (r=0.40) and quiet rest (r=0.49). Recent evidence has suggested that the PSWQ is sensitive to change in response to acute aerobic exercise (McDowell et al., 2016).

State Anxiety. State anxiety, conceptualized as transient, fluctuating feelings of tension and apprehension, was measured with the 20-item state subscale of the State-Trait Anxiety Inventory (STAI-Y1) (Speilberger et al., 1983). Participants rated each item using a 4-item Likert scale

ACCEPTED MANUSCRIPT

from 1 “Not at all” to 4 “Very much so;” item responses are summed after reverse scoring such that total score ranges from 20-80. The STAI-Y1 demonstrated adequate internal consistency in the current sample (α=0.90), and correlations between repeated measures were r=0.91 and r=0.86 for exercise and control, respectively.

Feelings of Energy and Fatigue. Feelings of energy and fatigue, conceptualized as subjective perceptions of the capacity or reduced capacity to complete mental and physical activities, were measured with the vigor and fatigue subscales of the Profile of Mood States – Brief Form (POMS-B). These subscales reliably measure the intensity of feelings of energy and fatigue (McNair, Droppleman, & Lorr, 1992; O'Connor, 2004). Participants were instructed to respond to the POMS-B “based on how you feel RIGHT NOW,” and for each subscale rated 5 adjectives using a 5-item Likert scale from 0 “Not at all” to 4 “Extremely.” Subscale scores range from 0-20. Internal consistency was adequate in the current sample for fatigue (α=0.89) and vigor (α=0.90). For the fatigue subscale, correlations between repeated measures were r=0.52 and r=0.90 for exercise and control, respectively. For the vigor subscale, correlations between repeated measures were r=0.56 and r=0.83, respectively.

Conditions

Acute Aerobic Exercise. Participants completed a vigorous (Committee, 2008) 30-min supervised bout of running on a Woodway Pro® treadmill at 65%-85% of maximal heart rate reserve (%HRR). Intensity (%HRR) was estimated as a function of predicted maximal heart rate and an age-related norm resting heart rate for females of 73bpm. In the absence of incremental exercise testing to determine aerobic capacity, this method has demonstrated validity for estimating vigorous intensity exercise dose in field-based exercise studies (Miller et al., 2014). Polar FT60® HR monitors were used to set the heart rate training zone and to continuously

ACCEPTED MANUSCRIPT

monitor HR and in-zone exercise minutes during exercise. Each participant was instructed to perform a 5-min warm-up, progressively increasing the treadmill speed and/or grade to achieve 65%HRR, and to then complete 30 min at a heart rate between 65%-85%HRR. However, most participants achieved 65%HRR within five minutes; therefore, warm-up periods ranged from 1-5min. When 30 min at 65%-85%HRR had been completed, each participant walked until she felt sufficiently cooled down. Again, some participants cooled down more rapidly than others; therefore, the cool-down period ranged from 1-5min. As shown later in the Results section, all participants completed >30 min at 65%-85%HRR, there was little variability in in-zone exercise minutes, and differences in both total exercise time that resulted from differing warm-up and cooldown time and the exact %HRR at which each participant exercised were not significantly associated with outcomes or outcome response to exercise. Each participant provided a session Rating of Perceived Exertion (RPE) using Borg's 6-20 RPE scale (Borg, 1982). Standard instructions for RPE were provided to each individual. Given the potential mood effects of social interaction (McNeil, LeBlanc, & Joyner, 1991), extraneous conversation was minimized during the exercise session.

Control: Seated Quiet Rest. Each participant completed 30 min of seated quiet rest in the same location as exercise testing. Procedures were consistent with the exercise session except that each participant sat in an upright chair in a quiet area of the lab for 30 min. Extraneous conversation with each participant was kept to an absolute minimum during the quiet rest session. Participants were not permitted to read or listen to music given the potential for altered mood responses.

ACCEPTED MANUSCRIPT

Analyses

Data analyses were performed using SPSS 22.0. Pearson's correlation coefficients quantified associations among participant characteristics and outcomes. Independent samples *t*-tests examined baseline differences in outcomes based on high trait anxious, depression, and poor sleep status. Paired samples *t*-tests quantified within-condition change for exercise and quiet rest. The magnitude of within-condition change was quantified with standardized mean difference (d); effects were calculated such that pre-post improvements resulted in a positive effect size. Two condition (exercise/quiet rest) X two time (pre/post) repeated measures ANOVA examined differences between exercise and quiet rest. However, given increased calls to move beyond null-hypothesis significance testing in favor of effect sizes and confidence intervals (Cumming, 2013), we also quantified and compared the magnitude of change in outcome responses using Hedges' *d* effect sizes and associated 95% confidence intervals (95%CI). For each outcome measure, the mean pre-condition to post-condition change for the quiet rest condition was subtracted from the mean pre-condition to post-condition change for the exercise condition and divided by the pooled pre-condition standard deviation (Hedges & Olkin, 1985). Effect sizes were adjusted for small sample bias and calculated such that improved moods resulted in positive effect sizes (Hedges & Olkin, 1985). Independent samples *t*-tests of pre-post exercise and pre-post quiet rest change scores examined within-condition differences in outcome responses based on high trait anxious status, depression status, and poor sleep status.

Results

Baseline Characteristics, Associations, and Differences Based on High Trait Anxious, Depression, and Poor Sleep Status. Baseline participant characteristics are presented in Table 1, and Table 2 presents baseline associations between participant characteristics and outcomes.

ACCEPTED MANUSCRIPT

Significant moderate-to-large, positive associations were found between worry and worry engagement, absence of worry, trait anxiety, depressive symptoms, and sleep quality (all p<0.05), between worry engagement and depressive symptoms, absence of worry, state anxiety, and sleep quality (all p<0.05), between absence of worry and depressive symptoms and sleep quality (all p<0.05), between trait anxiety and depressive symptoms, state anxiety, and sleep quality (all p<0.05), between state anxiety and depressive symptoms (p<0.05), and between sleep quality and depressive symptoms (p<0.05). A significant negative association was found between feelings of energy and fatigue (p<0.05).

Baseline worry was significantly greater among participants with poor sleep (t_{15}=2.70, p≤0.017). Baseline state anxiety was significantly greater among high trait anxious participants (t_{15}=2.74, p≤0.015) and among depressed participants (t_{15}=2.55, p≤0.023).

Exercise Session Variables. Participants averaged 35.8±3.4 min of exercise, 30.3±0.16 min of which were in-zone (65%-85%HRR). Participants exercised at ~72.9±0.03 %HRR (range: 66%-79%), approximating an average session heart rate of 164.5±4.9 bpm and an average session RPE of 13±2 (range: 9 to 17). All participants completed >30min at 65%-85%HRR; there was little variability in in-zone exercise minutes, speed (~4-6 mph), or gradient (0-3%), and differences in total exercise time and exact %HRR at which participants exercised were not significantly associated with outcome responses to exercise (all p>0.10).

Within-Condition Outcome Responses. Pre- and post-condition means and standard deviations and standardized mean differences are presented for exercise and quiet rest in Table 3. Exercise significantly reduced worry (t_{16}=-2.26, p<0.04; d=0.28), worry engagement (t_{16}=-4.67, p<0.001; d=0.34), state anxiety (t_{16}=-4.01, p≤0.001; d=0.40), and feelings of fatigue (t_{16}=-3.60, p≤0.003;

ACCEPTED MANUSCRIPT

d=0.77), and significantly increased feelings of energy (t_{16}=4.16, $p \leq 0.001$; d=1.11). No significant change was found for absence of worry (t_{16}=-0.08, $p > 0.93$; d=0.00).

Quiet rest significantly increased feelings of fatigue (t_{16}=2.20, $p \leq 0.044$; d=0.19) and significantly decreased feelings of energy (t_{16}=-4.60, $p < 0.001$; d=-0.68). No significant changes were found for worry (t_{16}=-0.22, $p > 0.82$; d=0.01), worry engagement (t_{16}=0.35, $p > 0.73$; d=-0.04), absence of worry (t_{16}=-0.57, $p > 0.57$; d=0.17), or state anxiety (t_{16}=0.63, $p > 0.53$; d=-0.08).

Effects of Exercise Compared to Quiet Rest. Compared with quiet rest, acute aerobic exercise significantly improved worry engagement ($F_{(1, 16)}$=5.63, $p \leq 0.031$; η_p^2=0.27), state anxiety ($F_{(1, 16)}$=8.32, $p \leq 0.011$; η_p^2=0.34), and feelings of energy ($F_{(1, 16)}$=41.7 $p < 0.001$; η_p^2=0.75) and fatigue ($F_{(1,16)}$=17.1, $p \leq 0.001$, η_p^2=0.55). Condition X time interactions were not significant for worry (total PSWQ score) ($F_{(1, 16)}$=3.00 $p > 0.10$; η_p^2=0.17) or absence of worry ($F_{(1, 16)}$=0.02, $p > 0.88$; η_p^2=0.001). A significant main effect for time was found for worry ($F_{(1, 16)}$=4.92, $p \leq 0.042$; η_p^2=0.25).

As shown in Table 3, compared with quiet rest, acute exercise resulted in large improvements in feelings of fatigue (*d*=0.76, 95%CI: 0.06, 1.46) and energy (*d*=1.69, 95%CI: 0.90, 2.47). Small-to-moderate improvements were found for worry (*d*=0.25), worry engagement (*d*=0.37), and state anxiety (*d*=0.44). Quiet rest resulted in a small decrease in absence of worry (*d*=0.17).

Moderators of Exercise Effects

Within-Condition Differences & Potential Moderators. Significantly larger reductions in worry engagement in response to exercise were found among participants who were not high trait anxious (t_{15}=-2.56, $p \leq 0.022$). For quiet rest, compared to normal sleepers feelings of fatigue were significantly increased among poor sleepers (t_{15}=-3.39, $p \leq 0.004$).

ACCEPTED MANUSCRIPT

Discussion

To the authors' knowledge, this is the first experimental study to examine the effects of acute vigorous exercise on worry, state anxiety, and feelings of energy and fatigue among young women with indications of GAD. The primary findings of this pilot study were that, compared to 30 minutes of quiet rest, a 30-minute bout of aerobic exercise completed at an average intensity of 73%HRR significantly improved worry engagement, state anxiety, and feelings of energy and fatigue. Statistically nonsignificant improvements were also observed for total PSWQ worry score. The magnitude of improvements ranged from moderate-to-large, and is consistent with previously reported effects of acute exercise on mood states among healthy adults (Ensari et al., 2015; Loy, O'Connor, & Dishman, 2013; McDowell et al., 2016), young adults with persistent fatigue (Herring & O'Connor, 2009), adults with Multiple Sclerosis (Ensari, Sandroff, & Motl, 2016), and women with Major Depressive Disorder (Meyer, Koltyn, Stegner, Kim, & Cook, 2016b).

The small magnitude improvement in total worry score (d=0.25) is comparable to the previously reported effect of three bouts of moderate-intensity cycling among young women with GAD (d=0.23) (Herring et al., 2012). This finding is notable given slightly lower baseline worry scores in the current study (59.7±8.3 vs. 62.1±6.4) and potential differences that could be anticipated between the effects of a single bout of exercise and the accumulated benefit of multiple exercise bouts. Though the magnitude of change for worry of 0.25 approximated a 3.5% change, it is plausible that the PSWQ total score is less sensitive to acute changes in worry due partly to the poor psychometrics and limited sensitivity of the absence of worry subscale.

Acute exercise significantly improved the worry engagement subscale score, resulting in a significant, small-to-moderate improvement (d=0.37). Controversy regarding the

ACCEPTED MANUSCRIPT

appropriateness of a two-factor solution for the PSWQ has focused on whether or not the absence of worry factor has substantive meaning or is simply an irrelevant method effect arising from response styles associated with reverse-worded items (Brown, 2003). Some evidence has demonstrated that worry engagement has explained the majority of variance in symptom measures, but has also ascribed meaning to the absence of worry subscale as more than a response bias (Fresco et al., 2002). Similar to previous findings (Fresco et al., 2002), compared to PSWQ total score, worry engagement had similar internal consistency and comparable or greater correlations with other outcomes; absence of worry did not perform well psychometrically, had weaker correlations with other outcomes, and appeared less sensitive to acute change. Thus, the present findings suggest that worry engagement may provide a psychometrically sound, more sensitive measure of worry response to exercise when considered independent of absence of worry.

The magnitude of outcome improvements were influenced by differential responses to quiet rest. Within-condition analyses demonstrated moderate-to-large magnitude improvements in feelings of energy (d=1.11) and fatigue (d=0.77) following acute aerobic exercise; however, the large magnitude improvements compared to quiet rest resulted partly due to a significant worsening of feelings of energy (d=-0.68) and fatigue (d=0.19) following quiet rest. As a widely used control condition in studies of acute exercise, mood, and affect (Ensari et al., 2015; McDowell et al., 2016), it is somewhat surprising that ‘imposed’ quiet rest would negatively impact state anxiety and feelings of energy and fatigue. However, these findings are consistent with recent evidence showing elevated anxiety following ‘imposed’ sedentary behavior among active adults (Edwards & Loprinzi, 2016). It is plausible that quiet rest may serve as an imposed bout of sedentary behavior that acutely worsens mood states in some individuals.

ACCEPTED MANUSCRIPT

Notwithstanding the need for continued research into the plausible mechanisms of exercise effects on mood outcomes, the present findings encourage future investigation of factors associated with worsening of mood states during quiet rest, including sedentary behavior, rumination, and poor sleep quality, the time course of changes in response to quiet rest, and the most appropriate comparison condition for acute exercise.

The exercise protocol reported herein was selected for several reasons. Compared to low and moderate intensity exercise, less is known about mood or affective responses to bouts of vigorous intensity exercise, and even less evidence is available regarding self-selected intensities within a range of vigorous intensities. Participants were instructed to maintain an exercising heart rate between 65%-85%HRR, but were free to exercise within the prescribed range. This ensured both that a standardized vigorous intensity was achieved and that participants were allotted some preference regarding intensity within the prescribed vigorous range. Though preferred intensity has resulted in better compliance rates compared to prescribed intensity (Callaghan, Khalil, Morres, & Carter, 2011), prescribed intensity recently resulted in a significant, almost 4-fold larger improvement in depressed mood among women with Major Depressive Disorder (Meyer et al., 2016a). The present findings showed no significant differences between participants based on exercise intensity within the prescribed range. Thus, it may be particularly advantageous to allow participants to manipulate level of intensity within a prescribed intensity range.

The small sample size and somewhat favorable baseline mood profile among the sample studied here are potential limitations, though participants with more favorable trait anxiety profiles demonstrated significantly larger improvements in worry engagement following exercise. Additionally, a larger sample size could have provided sufficient power for the

ACCEPTED MANUSCRIPT

measurement of additional plausible moderators that could have provided further context for the present findings, including objective measures of attentional biases, plausible biomarkers, and sleep. Nonetheless, these findings represent the first investigation to show improvements in worry, state anxiety, and feelings of energy and fatigue in response to a single bout of vigorous intensity aerobic exercise among individuals with probable GAD.

Conclusions

The present findings provide initial support for the positive effects of acute aerobic exercise on worry, state anxiety, and feelings of energy and fatigue among young women with elevated worry indicative of GAD. Future research can benefit from larger sample sizes that compare equal distributions of female and male participants, comparisons of varying degrees of subclinical and clinical GAD severity, and the comparison of multiple exercise modes. The examination of plausible cognitive or biological mechanisms which may underlie exercise effects, including neurobiological and neurotrophic effects and reduced attentional biases, are also warranted.

ACCEPTED MANUSCRIPT

ACCEPTED MANUSCRIPT

References

Bandelow, B., & Michaelis, S. (2015). Epidemiology of anxiety disorders in the 21st century. *Dialogues in clinical neuroscience, 17*(3), 327.

Behar, E., Alcaine, O., Zuellig, A. R., & Borkovec, T. (2003). Screening for generalized anxiety disorder using the Penn State Worry Questionnaire: A receiver operating characteristic analysis. *Journal of behavior therapy and experimental psychiatry, 34*(1), 25-43.

Blair, S. N., Haskell, W. L., Ho, P., Paffenbarger, R. S., Vranizan, K. M., Farquhar, J. W., & Wood, P. D. (1985). Assessment of habitual physical activity by a sevenday recall in a community survey and controlled experiments. *American journal of Epidemiology, 122*(5), 794-804.

Borg, G. A. (1982). Psychophysical bases of perceived exertion. *Med sci sports exerc, 14*(5), 377-381.

Brown, T. A. (2003). Confirmatory factor analysis of the Penn State Worry Questionnaire: Multiple factors or method effects? *Behaviour Research and Therapy, 41*, 1411-1426.

Buysse, D. J., Reynolds, C. F., Monk, T. H., Berman, S. R., & Kupfer, D. J. (1989). The Pittsburgh Sleep Quality Index: a new instrument for psychiatric practice and research. *Psychiatry research, 28*(2), 193-213.

Buysse, D. J., Reynolds III, C. F., Monk, T. H., Berman, S. R., & Kupfer, D. J. (1989). The Pittsburgh Sleep Quality Index: a new instrument for psychiatric practice and research. *Psychiatry Research, 28*(2), 193-213.

Callaghan, P., Khalil, E., Morres, I., & Carter, T. (2011). Pragmatic randomised controlled trial of preferred intensity exercise in women living with depression. *BMC Public Health, 11*(1), 465.

Committee, P. A. G. A. (2008). Physical activity guidelines advisory committee report, 2008. *Washington, DC: US Department of Health and Human Services, 2008*.

Cumming, G. (2013). The new statistics why and how. *Psychological science*, 0956797613504966.

Edwards, M. K., & Loprinzi, P. D. (2016). Experimentally increasing sedentary behavior results in increased anxiety in an active young adult population. *Journal of Affective Disorders, 204*, 166-173.

Ensari, I., Greenlee, T. A., Motl, R. W., & Petruzzello, S. J. (2015). META-ANALYSIS OF ACUTE EXERCISE EFFECTS ON STATE ANXIETY: AN UPDATE OF RANDOMIZED CONTROLLED TRIALS OVER THE PAST 25 YEARS. *Depression and anxiety*.

Ensari, I., Sandroff, B. M., & Motl, R. W. (2016). Intensity of treadmill walking exercise on acute mood symptoms in persons with multiple sclerosis. *Anxiety, Stress, & Coping*, 1-11.

Fresco, D. M., Heimberg, R. G., Mennin, D. S., & Turk, C. L. (2002). Confirmatory factor analysis of the Penn State worry questionnaire. *Behaviour Research and Therapy, 40*(3), 313-323.

Hedges, L. V., & Olkin, I. (1985). Statistical methods for meta-analysis: Academic Press New York.

Herring, M. P., Jacob, M. L., Suveg, C., Dishman, R. K., & O'Connor, P. J. (2012). Feasibility of exercise training for the short-term treatment of generalized anxiety disorder: a randomized controlled trial. *Psychotherapy and Psychosomatics, 81*(1), 21-28.

Herring, M. P., Jacob, M. L., Suveg, C., & O'Connor, P. J. (2011). Effects of short-term exercise training on signs and symptoms of generalized anxiety disorder. *Mental health and physical activity, 4*(2), 71-77.

Herring, M. P., Johnson, K. E., & O'Connor, P. J. (2016). Exercise training and health-related quality of life in generalized anxiety disorder. *Psychology of Sport and Exercise, 27*, 138-141.

ACCEPTED MANUSCRIPT

ACCEPTED MANUSCRIPT

Herring, M. P., Kline, C. E., & O'Connor, P. J. (2015). Effects of exercise on sleep among young women with Generalized Anxiety Disorder. *Mental health and physical activity, 9*, 59-66.

Herring, M. P., & O'Connor, P. J. (2009). The effect of acute resistance exercise on feelings of energy and fatigue. *Journal of sports sciences, 27*(7), 701-709.

Loy, B. D., O'Connor, P. J., & Dishman, R. K. (2013). The effect of a single bout of exercise on energy and fatigue states: a systematic review and meta-analysis. *Fatigue: Biomedicine, Health & Behavior, 1*(4), 223-242.

McDowell, C. P., Campbell, M. J., & Herring, M. P. (2016). Sex-related differences in mood responses to acute aerobic exercise. *Medicine and Science in Sports and Exercise, 48*(9), 1798-1802.

McNair, D. M., Droppleman, L. F., & Lorr, M. (1992). *Edits manual for the profile of mood states: POMS*: Edits.

McNeil, J. K., LeBlanc, E. M., & Joyner, M. (1991). The effect of exercise on depressive symptoms in the moderately depressed elderly. *Psychology and Aging, 6*(3), 487.

Meyer, J. D., Ellingson, L. D., Koltyn, K. F., Stegner, A. J., Kim, J.-S., & Cook, D. B. (2016a). Psychobiological responses to preferred-and prescribed-intensity exercise in MDD. *Med. Sci. Sports Exerc.*

Meyer, J. D., Koltyn, K. F., Stegner, A. J., Kim, J.-S., & Cook, D. B. (2016b). Influence of Exercise Intensity for Improving Depressed Mood in Depression: A Dose-Response Study. *Behavior Therapy, 47*(4), 527-537.

Meyer, T. J., Miller, M. L., Metzger, R. L., & Borkovec, T. D. (1990). Development and validation of the penn state worry questionnaire. *Behaviour research and therapy, 28*(6), 487-495.

Miller, F. L., O'Connor, D. P., Herring, M. P., Sailors, M. H., Jackson, A. S., Dishman, R. K., & Bray, M. S. (2014). Exercise Dose, Exercise Adherence, and Associated Health Outcomes in the TIGER Study. *Medicine and science in sports and exercise, 46*(1), 69.

O'Connor, P. J. (2004). Evaluation of four highly cited energy and fatigue mood measures. *Journal of psychosomatic research, 57*(5), 435-441.

Remes, O., Brayne, C., Linde, R., & Lafortune, L. (2016). A systematic review of reviews on the prevalence of anxiety disorders in adult populations. *Brain and Behavior, 6*(7).

Ruscio, A. M., Chiu, W. T., Roy-Byrne, P., Stang, P. E., Stein, D. J., Wittchen, H.-U., & Kessler, R. C. (2007). Broadening the definition of generalized anxiety disorder: effects on prevalence and associations with other disorders in the National Comorbidity Survey Replication. *Journal of anxiety disorders, 21*(5), 662-676.

Rush, A. J., Trivedi, M. H., Ibrahim, H. M., Carmody, T. J., Arnow, B., Klein, D. N., . . . Manber, R. (2003a). The 16-Item Quick Inventory of Depressive Symptomatology (QIDS), clinician rating (QIDS-C), and self-report (QIDS-SR): a psychometric evaluation in patients with chronic major depression. *Biological psychiatry, 54*(5), 573-583.

Rush, A. J., Trivedi, M. H., Ibrahim, H. M., Carmody, T. J., Arnow, B., Klein, D. N., . . . Manber, R. (2003b). The 16-Item Quick Inventory of Depressive Symptomatology (QIDS), clinician rating (QIDS-C), and self-report (QIDS-SR): a psychometric evaluation in patients with chronic major depression. *Biological psychiatry*.

Speilberger, C. D., Gorsuch, R., Lushene, R., Vagg, P., & Jacobs, G. (1983). Manual for the state-trait anxiety inventory. *Palo Alto, CA: Consulting Psychologists*.

Spielberger, C. D. (1983). Manual for the State-Trait Anxiety Inventory STAI (form Y)(" self-evaluation questionnaire").

Steiner, M., Allgulander, C., Ravindran, A., Kosar, H., Burt, T., & Austin, C. (2005). Gender differences in clinical presentation and response to sertraline treatment of generalized anxiety disorder. *Human Psychopharmacology Clinical and Experimental, 20*(1), 3-13.

Vesga-López, O., Schneier, F., Wang, S., Heimberg, R., Liu, S.-M., Hasin, D. S., & Blanco, C. (2008). Gender differences in generalized anxiety disorder: results from the National

ACCEPTED MANUSCRIPT

ACCEPTED MANUSCRIPT

Epidemiologic Survey on Alcohol and Related Conditions (NESARC). *The Journal of clinical psychiatry, 69*(10), 1606.

Wolitzky-Taylor, K., Dour, H., Zinbarg, R., Mineka, S., Vrshek-Schallhorn, S., Epstein, A., . . . Nazarian, M. (2014). Experiencing core symptoms of anxiety and unipolar mood disorders in late adolescence predicts disorder onset in early adulthood. *Depression and anxiety, 31*(3), 207-213.

ACCEPTED MANUSCRIPT

ACCEPTED MANUSCRIPT

Table 1. Baseline Participant Characteristics

**Age (y)**	20.8±1.4
**Weight (kg)**	62.9±6.2
**BMI (kg/m^2)**	22.2±1.8
**Underweight (%)**	1 (5.9%)
**Normal (%)**	16 (94.1%)
**Overweight (%)**	0 (0%)
**Smoker (%)**	0 (0%)
**Pain (%)**	0 (0%)
**Contraceptive (%)**	6 (35.3%)
**Physical Activity (kcal/wk)**	328.1±51.2
**Sleep (PSQI)**	5.0±1.9
**Poor sleeper (PSQI>5; %)**	8 (47.1%)
**High trait anxious (%)**	7 (41.2%)
**Depressed (QIDS>5; %)**	8 (47.1%)
**None (%)**	8 (47.1%)
**Mild depression (%)**	7 (41.2%)
**Moderate depression (%)**	1 (5.9%)
**Trait anxiety (STAI-Y2)**	45.9±8.6
**State anxiety (STAI-Y1)**	35.8±9.0
**Feelings of Energy (POMS-B)**	6.9±4.2
**Feelings of Fatigue (POMS-B)**	5.6±4.2
**Depression (QIDS)**	5.5±3.4
**Worry (PSWQ)**	59.7±8.3
**Worry Engagement (PSWQ)**	40.0±6.2
**Absence of Worry (PSWQ)**	19.7±2.8

Abbreviations: y: years; kg: kilograms; BMI: Body Mass Index; kg/m^2: kilograms per meter-squared; kcal/wk: kilocalories per week; PSQI: Pittsburgh Sleep Quality Index; QIDS: Quick Inventory of Depressive Symptomatology; STAI-Y2: Trait Subscale of the State-Trait Anxiety Inventory; STAI-Y1: State Subscale of the State-Trait Anxiety Inventory; POMS-B: Profile of Mood States-Brief; PSWQ: Penn State Worry Questionnaire

ACCEPTED MANUSCRIPT

ACCEPTED MANUSCRIPT

Table 2. Baseline Associations Between Participant Characteristics and Outcome Measures

	Age (y)	Weight (kg)	BMI (kg/m^2)	Physical Activity (kcals)	Trait Anxiety (STAI-Y2)	Depressive Symptoms (QIDS)	Worry (PSWQ)	Worry Engagement (PSWQ)	Absence of Worry (PSWQ)	State Anxiety (STAI-Y1)	Sleep Quality (PSQI)	Energy (POMS-B)	Fatigue (POMS-B)
Age (y)	1												
Weight (kg)	0.14	1											
BMI (kg/m^2)	0.42	0.59*	1										
Physical Activity (kcals)	0.01	0.48	0.18	1									
Trait Anxiety (STAI-Y2)	0.18	-0.04	0.08	0.16	1								
Depressive Symptoms (QIDS)	0.02	0.05	0.08	0.37	0.83****	1							
Worry (PSWQ)	0.01	-0.23	-0.09	-0.14	0.50*	0.64**	1						
Worry Engagement (PSWQ)	0.13	-0.28	-0.13	-0.24	0.44	0.56*	0.96****	1					
Absence of Worry (PSWQ)	-0.26	-0.04	0.03	0.12	0.48	0.64**	0.80****	0.61**	1				
State Anxiety (STAI-Y1)	0.29	-0.04	0.13	-0.05	0.65***	0.56*	0.46	0.54*	0.14	1			
Sleep Quality (PSQI)	0.35	0.15	0.15	0.25	0.65***	0.58*	0.61**	0.52*	0.62**	0.48	1		
Energy (POMS-B)	-0.10	-0.44	-0.14	-0.19	0.14	0.12	0.32	0.30	0.26	0.32	0.18	1	
Fatigue (POMS-B)	0.12	0.08	0.22	-0.11	0.28	0.16	0.28	0.26	0.26	0.10	0.19	-0.56*	1

*$p<0.05$
**$p\leq0.01$
***$p\leq0.005$
****$p\leq0.001$

Abbreviations: y, years; kg, kilograms; BMI, body mass index; kg/m^2, kilograms per meter squared; kcals, kilocalories; STAI-Y2, State-Trait Anxiety Inventory – Trait; QIDS, Quick Inventory of Depressive Symptomatology; PSWQ, Penn State Worry Questionnaire; POMS-B, Profile of Mood States-Brief; STAI-Y1, State-Trait Anxiety Inventory – State; PSQI, Pittsburgh Sleep Quality Index

ACCEPTED MANUSCRIPT

ACCEPTED MANUSCRIPT

443 Table 3. Pre- and Post-Condition Means (SD), Standardized Mean Differences (d), and Hedges' *d* Effect Sizes (95%CI)

	Pre-EX	Post-EX	SMD (d)	Pre-CON	Post-CON	SMD (d)	Hedges' *d* (95% CI)
**Worry (PSWQ)**	60.2 (8.3)	57.9 (9.5)	0.28	58.6 (9.2)	58.5 (8.7)	0.01	0.25 (-0.43, 0.92)
**Worry Engagement (PSWQ)**	39.8 (6.7)	37.5 (7.6)	0.34	39.1 (7.2)	39.4 (6.8)	-0.04	0.37 (-0.31, 1.04)
**Absence of Worry (PSWQ)**	20.4 (2.9)	20.4 (2.7)	0	19.6 (3.0)	19.1 (3.8)	0.17	-0.17 (-0.84, 0.51)
**State anxiety (STAI-Y1)**	36.0 (8.2)	32.7 (7.9)	0.40	36.4 (9.9)	37.2 (9.9)	-0.08	0.44 (-0.24, 1.12)
**Energy (POMS-B)**	5.1 (3.7)	9.2 (4.3)	1.11	7.4 (4.7)	4.2 (3.7)	-0.68	1.69 (0.90, 2.47)*
**Fatigue (POMS-B)**	5.4 (3.0)	3.1 (3.4)	0.77	5.8 (5.2)	6.8 (5.7)	0.19	0.76 (0.06, 1.46)*

444 * $p < 0.05$; significant improvement for exercise compared to quiet rest based on 95%CI not encompassing 0.

445 **Abbreviations:** STAI-Y1: State Subscale of the State-Trait Anxiety Inventory; POMS: Profile of Mood States-Brief; PSWQ: Penn State Worry Questionnaire

ACCEPTED MANUSCRIPT

ACCEPTED MANUSCRIPT

Highlights

- Acute exercise effects in adults with clinical or subclinical GAD are unstudied
- Seventeen young women with probable GAD completed acute exercise and quiet rest
- Exercise significantly improved worry engagement, state anxiety, and energy and fatigue
- Moderate-to-large improvements were found for state anxiety and energy and fatigue
- Quiet rest appeared to worsen outcomes, particularly feelings of energy and fatigue

ACCEPTED MANUSCRIPT
