# Supplementary material for: Does physical activity-based intervention decrease repetitive negative thinking? A systematic review
Source: PLoS One. 2025 Apr 1;20(4):e0319806. doi: 10.1371/journal.pone.0319806 (PMC11960971; doi:10.1371/journal.pone.0319806)
Supplement: S1 File — https://doi.org/10.6084/m9.figshare.25711734. (ZIP) [file pone.0319806.s001.zip › supporting information/paper file/Jacoby2024.pdf]

Check for updates

Received: 6 April 2023 | Revised: 6 June 2023 | Accepted: 23 June 2023

DOI: 10.1111/jsr.13992

RESEARCH ARTICLE

Journal of Sleep Research ESRS

Effect of cognitive behavioural therapy and yoga for generalised anxiety disorder on sleep quality in a randomised controlled trial: the role of worry, mindfulness, and perceived stress as mediators

Ryan J. Jacoby[1] | Mackenzie L. Brown[2] | Sarah T. Wieman[3] | David Rosenfield[4] | Susanne S. Hoeppner[1] | Eric Bui[5] | Elizabeth A. Hoge[6] | Sat Bir S. Khalsa[7] | Stefan G. Hofmann[8] | Naomi M. Simon[9]

[1]Massachusetts General Hospital/Harvard Medical School, Department of Psychiatry, Boston, Massachusetts, USA

[2]University of Louisville, Department of Psychology, Louisville, Kentucky, USA

[3]Suffolk University, Department of Psychology, Boston, Massachusetts, USA

[4]Southern Methodist University, Department of Psychology, Dallas, Texas, USA

[5]Normandie Univ, UNICAEN, INSERM, U1237, PhIND 'Physiopathology and Imaging of Neurological Disorders', NEUROPRESAGE Team, Institut Blood and Brain at Caen-Normandie, GIP Cyceron, Caen, France and Centre Hospitalier Universitaire Caen Normandie, Caen, France

[6]Georgetown University Medical Center, Department of Psychiatry, Washington, DC, USA

[7]Brigham and Women's Hospital/Harvard Medical School, Division of Sleep and Circadian Disorders, Department of Medicine, Boston, Massachusetts, USA

[8]Philipps-University Marburg, Department of Clinical Psychology, Marburg/Lahn, Germany

[9]New York University Grossman School of Medicine, Department of Psychiatry, New York, New York, USA

Correspondence
Ryan J. Jacoby, Massachusetts General Hospital/Harvard Medical School, Department of Psychiatry, 185 Cambridge St, Suite 2000, Boston, MA 02114, USA.
Email: rjjacoby@mgh.harvard.edu

Funding information
National Center for Complementary and Integrative Health, Grant/Award Numbers: R01AT007257, R01AT007258; National Institute of Mental Health, Grant/Award Number: K23MH120351

Summary

Sleep disturbances are present in ~65% of individuals with generalised anxiety disorder (GAD). Although both Kundalini yoga (KY) and cognitive behavioural therapy (CBT) are effective treatment options for GAD, little is known about how these treatments compare in improving sleep for GAD and what drives these changes. Accordingly, we examined the effects of CBT, KY, and stress education (SEdu; an attention control condition) on subjective sleep quality (as measured by the Pittsburgh Sleep Quality Index [PSQI] and Insomnia Severity Index [ISI]) in a randomised controlled trial of 226 adults with GAD (mean age 33.37 years; 70% female; 79% White). We hypothesised that both CBT and KY would outperform SEdu in improving sleep disturbances. Three potential mediators of sleep improvement (worry, mindfulness, perceived stress) were also examined. In line with hypotheses, PSQI and ISI scores significantly improved from pre- to post-treatment for all three treatment groups (all $p < 0.001$, all $d > 0.97$). However, contrary to predictions, sleep changes were not significantly greater for CBT or KY compared to SEdu. In mediation analyses, within-person deviations in worry, mindfulness, and stress each significantly mediated the effect of time on sleep outcomes. Degree of change in sleep

Sat Bir S. Khalsa, Stefan G. Hofmann and Naomi M. Simon are co-senior authors: khalsa@hms.harvard.edu, stefan.hofmann@uni-marburg.de, naomi.simon@nyulangone.org.

Clinical Trials Registration: GATE: Generalized Anxiety—A Treatment Evaluation, ClinicalTrials.gov Identifier: NCT01912287 (https://clinicaltrials.gov/ct2/show/NCT01912287).

© 2023 European Sleep Research Society.

attributable to worry (CBT > KY > SEdu) and perceived stress (CBT, KY > SEdu) was moderated by treatment group. Personalised medicine as well as combined treatment approaches should be studied to help reduce sleep difficulties for patients with GAD who do not respond.

KEYWORDS
anxiety, cognitive behavioural therapy, insomnia, mechanisms, sleep, stress, yoga

1 | INTRODUCTION

Generalised anxiety disorder (GAD) is a common anxiety disorder characterised by excessive anxiety and worry about varied topics affecting 5.7% of Americans in their lifetime (Ruscio et al., 2017). GAD is also associated with physical symptoms and is the only anxiety disorder that includes sleep disturbance in its diagnostic criteria (e.g., difficulty falling or staying asleep; American Psychiatric Association, 2013). Of patients with GAD 50%–70% endorse insomnia symptoms (i.e., persistent trouble falling asleep, staying asleep, and/or poor quality sleep associated with significant distress or impairment; American Psychiatric Association, 2013) that are as severe as their anxiety symptoms (e.g., Boland & Ross, 2015). Furthermore, sleep disturbances in GAD are associated with a wide range of impairments (Bélanger et al., 2004; Brenes et al., 2009; Glidewell et al., 2015), including impaired concentration and memory, decreased occupational functioning, increased absenteeism, and increased use of medical services (Benz et al., 2020; Harvey et al., 2009). Chronic insomnia may also be a risk factor for treatment relapse (Ohayon & Roth, 2003). Therefore, the additive clinical effects of anxiety and sleep problems warrant further exploration of treatment approaches that may address sleep in the context of GAD.

Cognitive behavioural therapy (CBT) is the 'gold standard' psychological treatment for anxiety (e.g., Carpenter et al., 2018). Clinical trials of CBT for GAD and elevated worry have shown positive effects on sleep (Belleville et al., 2010; Brenes et al., 2020; Bush et al., 2012; Ramsawh et al., 2016). However, many trials have found insomnia to be a residual symptom following anxiety disorder treatment (Glidewell et al., 2015), and the mechanisms by which CBT for GAD improves sleep are not well understood. There is evidence that the core symptom of GAD, pathological worry, may be a key mechanism in the development of sleep problems (Åkerstedt et al., 2007; Harvey et al., 2009). Elevated worry before bed is consistently associated with sleep interference (e.g., lower sleep efficiency) in healthy controls (e.g., Åkerstedt et al., 2007; Brosschot et al., 2007). Moreover, pathological worries about sleep (e.g., ruminative thoughts about the consequences of lost sleep, unrealistic expectations about sleep) may contribute to the maintenance of insomnia (Cousineau et al., 2016). Accordingly, the positive effects of CBT for GAD on sleep may be in part due to targeting pathological worries (Belleville et al., 2010; Sánchez-Ortuño & Edinger, 2012), although this has not yet been directly tested.

Complementary and integrative mind–body practices, such as yoga, are increasingly popular interventions for GAD and anxiety (Belleville et al., 2010; Jeter et al., 2015; Sánchez-Ortuño & Edinger, 2012). Yoga includes physical exercises, breath regulation, relaxation, and meditation, which are believed to facilitate reductions in psychophysiological arousal and increases in mindfulness (Hofmann et al., 2015; Hoge et al., 2013). There is also evidence for improvements in sleep/insomnia with yoga and mindfulness practices (Khalsa & Goldstein, 2021; Rusch et al., 2019; Wang et al., 2016; Wang et al., 2019). However, there is limited research investigating whether mind body interventions improve sleep among patients with GAD specifically (Hoge et al., 2013; Khalsa et al., 2015; Vøllestad et al., 2011). More research is also needed to clarify the mechanisms, such as mindfulness, by which these interventions have their effect on sleep in GAD. Research suggests that mindfulness likely improves insomnia symptoms due to reducing hyperarousal as well as improving self-regulation of thought processes (Ong & Kalmbach, 2023), suggesting that mindfulness is a promising mechanism for further study.

In summary, although evidence supports the efficacy of CBT and mind–body interventions for improving sleep among patients with GAD, there is a dearth of research directly comparing these treatments and testing potential mechanisms. Accordingly, in this secondary data analysis, we examined the efficacy of both group CBT and Kundalini yoga (KY), compared with a stress education (SEdu) attention control treatment (that involved psychoeducation about the effects of stress on the body and addressing factors that can alleviate stress), on subjective sleep quality among patients with GAD enrolled in a randomised controlled trial (RCT; Hofmann et al., 2015; Simon et al., 2021). We hypothesised that scores on the Pittsburgh Sleep Quality Index (PSQI) and Insomnia Severity Index (ISI) would significantly decrease from baseline to post-treatment (regardless of intervention; Hypothesis 1A). We also examined whether these changes were moderated by treatment group (CBT, KY, SEdu) and hypothesised that CBT and KY would both outperform SEdu in improving sleep quality (CBT and KY > SEdu; Hypothesis 1B).

We additionally examined three potential mediators of sleep quality improvements (Aim 2) including worry and mindfulness, the previously mentioned targets of CBT and KY respectively. We also examined general perceived stress as a non-specific mediator because in previous research although the frequency of daily stressors did not differ between participants with insomnia and those who reported satisfaction with their sleep, individuals with insomnia perceive daily

13652869, 2024, 1, Downloaded from https://onlinelibrary.wiley.com/doi/10.1111/jsr.13992 by University Of British Columbia, Wiley Online Library on [30/01/2025]. See the Terms and Conditions (https://onlinelibrary.wiley.com/terms-and-conditions) on Wiley Online Library for rules of use; OA articles are governed by the applicable Creative Commons License

events to be more stressful (Morin et al., 2003), suggesting that sensitivity to stress may contribute to sleep difficulties. We hypothesised that all three mediators (worry, mindfulness, perceived stress) would be related to sleep quality outcomes (i.e., ISI and PSQI scores as a multivariate outcome) over and above the effects of the other mediators (Hypothesis 2). In moderated mediation analyses (Aim 3), we also examined whether mediated pathways were different across the three treatment groups, because some treatments might be more effective at changing certain mediators. Specifically, we predicted that CBT would change worry more than the other treatments (Hypothesis 3a) and that KY would change mindfulness more than the other treatments (Hypothesis 3b).

2 | METHODS

2.1 | Participants

Participants ($N = 226$; mean age 33.37 years; 70% female; 79% White; 12.4% Hispanic) with a primary diagnosis of GAD as determined by the Structured Diagnostic Interview for Diagnostic and Statistical Manual of Mental Disorders, Fourth Edition (DSM-IV; First et al., 2002) or the Anxiety Disorders Interview Schedule-5 (Brown & Barlow, 2014) were enrolled in a two-site RCT (Hofmann et al., 2015; Simon et al., 2021). Participants were randomly assigned to 12 weeks of treatment in one of three groups (2:2:1 ratio): CBT, $n = 90$; KY, $n = 93$; SEdu, $n = 43$. Demographics of the sample and primary study outcomes can be found in Simon et al. (2021). Institutional Review Board approvals and written informed consent were obtained.

Inclusion criteria included adults with a primary diagnosis of GAD and a Clinical Global Impression Scale-Severity (Guy, 1976) score of at least 'moderately ill' for GAD symptom severity who were free of or on stable psychotropic medication. Exclusion criteria included: (a) currently practicing mind–body techniques (e.g., yoga, meditation, Tai Chi) or enrolled in psychotherapy for GAD, (b) more than five yoga classes or CBT sessions in the last 3 years, (c) lifetime history of a psychotic, bipolar, or developmental disorder, (d) diagnosis of post-traumatic stress disorder, substance use disorder, eating disorder, or organic mental disorder within the past 6 months, (e) significant suicidal ideation or suicidal behaviours within the past 6 months, (f) women who were pregnant, planning to become pregnant, or on an unreliable form of birth control, (g) cognitive impairment, or (h) serious medical illness or instability or history of head trauma. See Hofmann et al. (2015) for more details.

2.2 | Measures

The PSQI (Buysse et al., 1989) is a 19-item self-report measure that evaluates past month sleep quality. The PSQI has seven sub-scores with items rated on a 4-point scale: (a) subjective sleep quality (i.e., 'For the past month, how would you rate your sleep quality overall'), (b) sleep latency (i.e., how long it takes to fall asleep), (c) sleep duration (i.e., hours of actual sleep), (d) habitual sleep efficiency (i.e., ratio of number of hours slept divided by number of hours spent in bed), (e) sleep disturbance (i.e., reasons for sleep troubles; e.g., pain, trouble breathing, bad dreams), (f) use of sleep medications, and (g) daytime dysfunction (i.e., trouble staying awake and maintaining enthusiasm for daily activities). A global score ranging from 0 to 21 is calculated with higher scores indicating greater sleep disturbance and poorer sleep quality. A cut-off of >8 was established in clinical populations for poor sleep quality (Carpenter & Andrykowski, 1998; Ibáñez del Prado & Cruzado, 2020). The PSQI has been shown to have good test–retest reliability and validity (Backhaus et al., 2002); $\alpha = 0.66$–0.67 in the present study.

The ISI (Bastien et al., 2001) is a seven-item self-report measure of DSM-IV insomnia in the past 2 weeks, which measures severity (e.g., difficulty falling and staying asleep), distress (e.g., worry about sleep problems), and interference of sleep problems (e.g., self-perception of interference with daily functioning). The ISI uses a 5-point Likert scale, total scores range from 0 to 28, and higher scores represent more severe insomnia (cut-off score ≥10 indicate cases of insomnia in community samples; Morin et al., 2011). The ISI has demonstrated good reliability and validity (Bastien et al., 2001); $\alpha = 0.86$–0.88 in the present study.

The Penn State Worry Questionnaire-Past Week (PSWQ-PW; Stöber & Bittencourt, 1998) is a self-report measure of pathological worry severity over the past week. The PSWQ-PW includes 15-items rated on a 7-point Likert scale from 0 (*Never*) to 6 (*Almost always*, e.g., 'My worries overwhelmed me'). Items worded in the positive direction are reverse scored, with total scores from 0 to 90 (higher scores indicate greater worry severity). The PSWQ-PW has good test–retest reliability and high internal consistency (Stöber & Bittencourt, 1998); $\alpha = 0.89$–0.96 in the present study.

The Five-Facet Mindfulness Questionnaire (FFMQ; Baer et al., 2006) is a 39-item self-report measure of five key components of mindfulness: (a) observing (e.g., 'I pay attention to sensation, such as the wind in my hair or sun on my face'), (b) describing (e.g., 'I'm good at finding words to describe my feelings'), (c) acting with awareness (e.g., 'I find it difficult to stay focused on what's happening in the present'), (d) non-judging of inner experience (e.g., 'I make judgments about whether my thoughts are good or bad'), and (e) non-reactivity to inner experience (e.g., 'When I have distressing thoughts or images I am able just to notice them without reacting'). Items are rated on a 5-point Likert scale from 1 (*Never or rarely true*) to 5 (*Very often or always true*); items worded in the negative direction are reverse scored. The overall score ranges from 39 to 195 (higher scores indicate more mindfulness). The five-factor structure has been validated in meditating and non-meditating samples (Baer et al., 2008); α's $= 0.80$–0.92 in the present study.

The Perceived Stress Scale (PSS; Cohen et al., 1983) is a 10-item self-report measure of the degree to which situations in one's life are appraised as stressful over the last month (e.g., 'In the last month, how often have you found that you could not cope with all the things that you had to do'). Items are rated on a 5-point Likert scale from 0 (*Never*) to 4 (*Very often*). Items worded in the positive direction are

13652869, 2024, 1, Downloaded from https://onlinelibrary.wiley.com/doi/10.1111/jsr.13992 by University Of British Columbia, Wiley Online Library on [30/01/2025]. See the Terms and Conditions (https://onlinelibrary.wiley.com/terms-and-conditions) on Wiley Online Library for rules of use; OA articles are governed by the applicable Creative Commons License

reverse scored; total scores range from 0 to 40, with higher scores indicating more perceived stress. The PSS has been shown to have adequate internal and test–retest reliability (Cohen et al., 1983); $\alpha = 0.80–0.89$ in the present study.

2.3 | Procedure

Participants were randomised to a group (CBT, KY, or SEdu) for 12 consecutive weekly 60-min sessions and daily 20-min homework practices between sessions. Each group type was led by two trained instructors with three to six patients per group. The assessments in this secondary analysis occurred at baseline and after Week 12 of treatment.

2.3.1 | Treatments

Group CBT consisted of psychoeducation, cognitive restructuring, progressive muscle relaxation, worry exposures, and in vivo exposure exercises (mindfulness was not addressed). Group KY (as taught by Yogi Bhajan) included breathing techniques, meditation, yoga theory, philosophy and psychology, physical postures/exercises, and deep relaxation. Group SEdu received psychoeducation about the stress response, the negative stress cycle, stress and health/illness, and the contribution of lifestyle behaviours such as caffeine and alcohol intake. This included providing general psychoeducation about sleep physiology (e.g., the stages of sleep), misconceptions about sleep, and sleep hygiene, but this was neither prescriptive nor personalised. For additional details on the treatment manual, see Hofmann et al. (2015).

2.4 | Statistical analysis

Our primary analyses included the full sample of study participants (regardless of whether they scored above the clinical cut-off for insomnia). Sleep quality outcomes for Aim 1 (PSQI and ISI) were analysed using multi-level modelling (MLM). The predictors were Time (weeks from baseline), Treatment Group (dummy coded to represent comparisons between CBT and SEdu and between KY and SEdu), and Time × Treatment Group. MLM includes all participants who complete at least one assessment (all 226 participants in the present study), regardless of other missing data. The MLMs had three levels: the two repeated measurements of sleep quality were nested within participants, who were nested within their treatment cohort. We followed with exploratory analyses to investigate whether specific aspects of sleep disturbance were impacted differently by the treatments by examining the seven PSQI subscales separately (correcting for multiple tests using the Benjamini–Hochberg correction).

For Aims 2–3, we conducted a longitudinal moderated mediation analysis using MLM. As we had two separate, strongly correlated measures of sleep quality, we performed a single multivariate mediation analysis (i.e., PSQI and ISI comprised the multivariate outcome) using multivariate MLM (MMLM; Hox et al., 2018), rather than perform a separate mediation analysis for each outcome. Instead of calculating the regression coefficients to maximise the fit of the predictors to a single outcome (as in MLM), MMLM maximises the fit to both outcomes simultaneously, calculating the regression coefficients to maximise the overall probability across both outcomes. So, the regression coefficients in MMLM will be similar to (but not exactly equal) the average of the regression coefficients if the outcomes had been analysed separately.

For the mediation analysis, we examined whether PSWQ, FFMQ, and/or PSS mediated the effect of Time on the outcomes (Figure 1). Measures were assessed at baseline and Week 12. We examined the within-person, longitudinal relations between the variables in our model because between-person relations can be confounded by between-subjects third variable confounds (Wang & Maxwell, 2015). The effect of Time on the mediators and on the outcome inherently calculate the within-subjects changes in those variables from baseline to post-treatment. Specifically, we followed Wang and Maxwell (2015) and disaggregated each mediator into its between-subjects component (the average level of the mediator over baseline and post-treatment), and its within-subjects component (the within-person deviation for each mediator at each time point from its own mean; Wang & Maxwell, 2015). Both the within-person deviations of each mediator at each timepoint, and the between-subjects mean on the mediator, were included in the model (Figure 1). All three mediators were included in a single mediation model (see Data S1 for more details).

The sleep quality outcomes were the PSQI and ISI (as the multivariate outcome). The deviations in each mediator at each timepoint was a time-varying predictor of outcome at each timepoint and reflected the within-person relation between each mediator and the sleep outcomes over time. As the change over time in the different mediators may vary between treatment groups (e.g., FFMQ may change more in KY than in SEdu), we allowed the effect of Time on the mediators to be moderated by Treatment Group (dummy coded as described in Aim 1; Figure 1). The 'a' paths in the mediation analysis were the effect of Time (i.e., the change over time) on each mediator using MLM. The 'b' paths estimated the within-person relationships between the repeated measurements of the mediators (as time-varying predictors) with the repeated measurements of the outcomes (Wang & Maxwell, 2015) using MMLM (as we had a multivariate outcome). Mediated pathways were tested for significance using the program *RMediation* (Tofighi & MacKinnon, 2011). As an effect size measure of the mediated pathways, we report P_M, which is the proportion of the effect of Time on sleep quality that was mediated by each particular mediator ($P_M = a \times b/c$). In additional exploratory analyses (See Data S1) we also examined two specific subscales of the FFMQ (acting with awareness and non-judging of inner experience) as mediators because a previous meta-analysis demonstrated that these two FFMQ subscales had the strongest relationships with affective symptoms (Carpenter et al., 2019). Finally, to determine if

13652869, 2024, 1, Downloaded from https://onlinelibrary.wiley.com/doi/10.1111/jsr.13992 by University Of British Columbia, Wiley Online Library on [30/01/2025]. See the Terms and Conditions (https://onlinelibrary.wiley.com/terms-and-conditions) on Wiley Online Library for rules of use; OA articles are governed by the applicable Creative Commons License

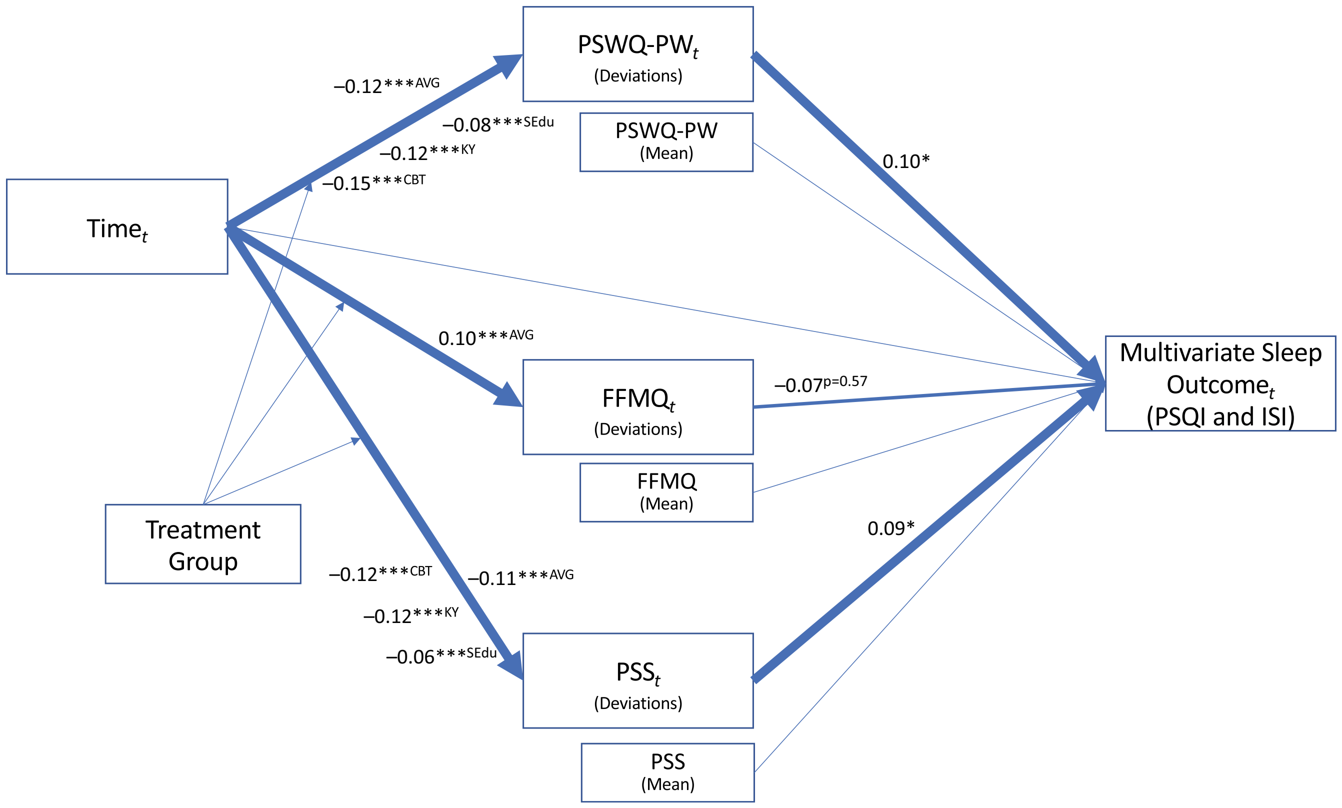

FIGURE 1 The PSWQ, FFMQ, and PSS as mediators of the change over time in the multivariate sleep outcome. CBT, cognitive behavioural therapy; KY, Kundalini yoga; SEdu, stress education; FFMQ, Five-Facet Mindfulness Questionnaire; PSWQ-PW, Penn State Worry Questionnaire-Past Week; PSS, Perceived Stress Scale; PSQI, Pittsburgh Sleep Quality Index; ISI, Insomnia Severity Index. The mediator boxes in the model represent the two separate components of each mediator: the mean level of the mediator across assessments, and the deviations at each timepoint between the value of the mediator at that timepoint and the mean level of the mediator for that participant. The path coefficients reported in the figure are for the deviation component of that mediator, as the mean component of the mediator may simply represent third variable confounds between participants. Paths in bold are significant. The non-bolded path from Time to outcome was not significant. ***p < 0.001, **p < 0.01, *p < 0.05, ^+p < 0.1. The subscript 't' indicates that the variable was measured repeatedly over time. Treatment Group was tested as a moderator of the change in the PSWQ, FFMQ, and PSS over time. Path coefficients that appear above the path line represent the average (AVG) change over time (per week) in the mediator across the three treatment groups (indicated by the 'AVG' superscript). Treatment Group was a significant moderator of the effect of time on the PSWQ and PSS, so the path coefficient for each treatment group is presented below the path line, with superscripts for each group. Treatment group was not a significant moderator of the change over time in the FFMQ, so separate path coefficients for each treatment group are not presented. Paths from the mean of each mediator to the outcome are not shown because they cannot mediate change over time given that they do not change over time.

our findings applied to the subset of participants reporting substantial insomnia, we re-calculated our analyses using the subgroup with an ISI score of ≥ 10 at baseline (See Data S1). Additional details for the statistical analytical plan can be found in the Data S1.

2.4.1 | Power analyses

Power analyses using the power analysis program *RMASS2* indicted that, given the number of participants in each treatment at each time point, we have >0.90 power to detect a medium effect size (Cohen's $d = 0.50$) difference between treatments on sleep quality outcomes for this secondary analysis. For the mediation analysis, Fritz and Mackinnon (2007) showed that sample sizes >75 are sufficient to have power >0.80 to detect mediation when the mediated pathways were of 'half-medium' effect size ($\beta = 0.26$).

3 | RESULTS

3.1 | Preliminary analyses

Descriptive statistics for outcome and mediator measures by intervention group and time point can be found in Table 1. Post-treatment assessments were completed by 155 participants (68.6%). As reported in the primary outcomes paper (Simon et al., 2021), dropouts did not differ from completers on baseline characteristics.

13652869, 2024, 1, Downloaded from https://onlinelibrary.wiley.com/doi/10.1111/jsr.13992 by University Of British Columbia, Wiley Online Library on [30/01/2025]. See the Terms and Conditions (https://onlinelibrary.wiley.com/terms-and-conditions) on Wiley Online Library for rules of use; OA articles are governed by the applicable Creative Commons License

TABLE 1 Raw means and standard deviations for outcome and mediator measures by intervention group and time point.

	Kundalini yoga		CBT		Stress education	
Variable, mean (SD)	Pre ($n = 93$)[a]	Post ($n = 59$)[b]	Pre ($n = 90$)[c]	Post ($n = 67$)	Pre ($n = 43$)[d]	Post ($n = 29$)[e]
PSWQ-PW	64.22 (11.73)	45.80 (19.00)	64.25 (12.70)	40.96 (17.13)	64.21 (10.38)	52.04 (17.52)
FFMQ Total	114.30 (15.75)	128.93 (18.64)	113.61 (18.94)	122.14 (20.49)	112.62 (15.42)	122.24 (17.45)
Observe	24.91 (6.28)	26.34 (7.61)	23.53 (6.00)	23.81 (6.18)	24.86 (5.47)	25.07 (5.60)
Describe	26.40 (6.43)	27.99 (6.53)	25.18 (7.38)	25.85 (6.95)	24.78 (6.35)	26.34 (6.44)
Acting with awareness	22.54 (6.22)	26.97 (5.98)	24.30 (5.86)	25.81 (6.31)	21.92 (5.68)	24.59 (5.09)
Non-judgement	23.32 (7.44)	28.74 (6.96)	23.87 (7.89)	27.77 (7.73)	24.02 (7.24)	26.52 (8.67)
Non-reactivity	17.13 (4.34)	18.90 (4.59)	16.73 (4.53)	18.90 (4.78)	17.04 (3.64)	19.72 (3.63)
PSS	23.67 (5.20)	18.17 (6.57)	23.84 (5.87)	17.86 (6.37)	23.83 (4.57)	20.45 (7.40)
PSQI	8.71 (3.19)	6.12 (3.41)	8.08 (3.93)	6.46 (3.25)	8.33 (3.98)	7.27 (3.40)
ISI	13.08 (6.65)	8.56 (5.67)	12.53 (6.71)	8.60 (5.33)	12.26 (6.15)	10.01 (5.84)

Abbreviations: CBT, cognitive behavioural therapy; FFMQ, Five-Facet Mindfulness Questionnaire; ISI, Insomnia Severity Index; Post, post-treatment; Pre, pre-treatment; PSS, Perceived Stress Scale; PSQI, Pittsburgh Sleep Quality Index; PSWQ-PW, Penn State Worry Questionnaire-Past Week.
[a]Kundalini Yoga Group—Pre: $n = 92$ for FFMQ, PSS, and ISI; $n = 85$ for PSQI.
[b]Kundalini Yoga Group—Post: $n = 57$ for PSQI.
[c]CBT Group—Pre: $n = 85$ for ISI.
[d]Stress Education Group—Pre: $n = 42$ for PSWQ-PW; $n = 41$ for FFMQ, PSS, and ISI; $n = 40$ for PSQI.
[e]Stress Education Group—Post: $n = 26$ for PSQI.

3.1.1 | Baseline characteristics

In the full sample, 47.6% ($n = 100$) were above the clinical cut-off of the PSQI at baseline (Carpenter & Andrykowski, 1998) and 63.7% ($n = 142$) were above the clinical cut-off on the ISI for insomnia (Morin et al., 2011). There were no site differences in baseline PSQI levels ($t[212] = -0.97$, $p = 0.33$, $d = 0.13$); one site had worse baseline insomnia as measured by the ISI (mean [SD] = 13.60 [7.08] versus 11.84 [5.93]; $t[208] = -2.00$, $p = 0.046$, $d = 0.28$). Additional baseline characteristics and baseline correlations between mediators and outcomes can be found in the Data S1.

3.2 | Aim 1: impact of treatment for GAD on sleep quality

At post-treatment, 32.0% ($n = 48$) were above the clinical cut-off of the PSQI and 41.3% ($n = 64$) were above the cut-off on the ISI for insomnia. PSQI scores decreased (improved) across all three treatment groups from pre-treatment (estimated marginal mean [EMM] [SE] = 8.38 [0.12]) to post-treatment (EMM [SE] = 6.66 [0.15]) with a large effect size ($b = -0.14$, 95% confidence interval [CI] = −0.17 to −0.11; $t = -9.03$, $df = 350$, $p < 0.001$, $d = 0.97$; Figure 2). The Time by Treatment Group interactions, testing change over time in CBT versus SEdu ($p = 0.73$) and change over time in KY versus SEdu ($p = 0.16$), were not significant, suggesting that the three treatment groups did not differentially improve overall sleep quality as measured by the PSQI.

The MLMs examining changes in the seven PSQI subscales showed that four subscales improved significantly over time after correction for multiple tests using the Benjamini–Hochberg adjustment: (a) subjective sleep quality, $t(184) = 5.03$, $p < 0.001$, $d = 0.74$, (b) sleep latency, $t(168) = 5.05$, $p < 0.001$, $d = 0.78$, (c) sleep duration, $t(167) = 2.29$, $p = 0.02$, $d = 0.35$, and (d) daytime dysfunction, $t(182) = 4.84$, $p < 0.001$, $d = 0.72$. The other three subscales demonstrated false discovery rates >0.05: habitual sleep efficiency ($p = 0.04$, $d = 0.31$), sleep disturbances ($p = 0.05$, $d = 0.30$), and use of sleep medications ($p = 0.10$, $d = 0.26$). There were no significant Treatment Group × Time interactions (all $p > 0.05$, all $d < 0.28$).

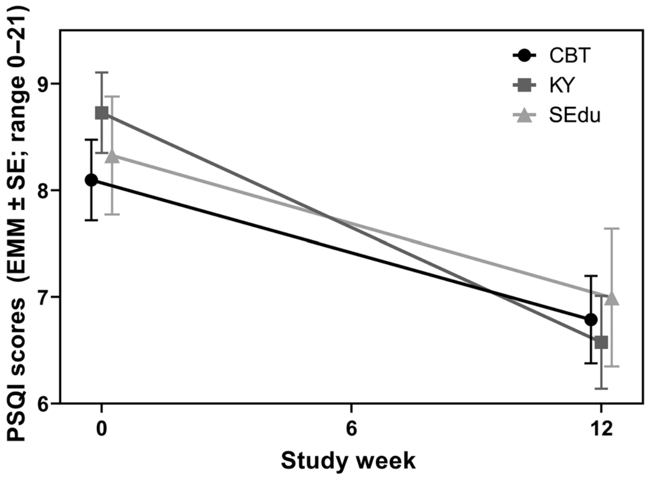

FIGURE 2 Changes in PSQI scores over time by treatment group. Figure depicts estimated marginal means (EMM) and standard errors (SEs). PSQI, Pittsburgh Sleep Quality Index; CBT, cognitive behavioural therapy; KY, Kundalini yoga; SEdu, stress education. We scaled the graph to clearly show the interaction (full PSQI range 0–21; cut-off score >8 indicates poor sleep quality).

13652869, 2024, 1, Downloaded from https://onlinelibrary.wiley.com/doi/10.1111/jsr.13992 by University Of British Columbia, Wiley Online Library on [30/01/2025]. See the Terms and Conditions (https://onlinelibrary.wiley.com/terms-and-conditions) on Wiley Online Library for rules of use; OA articles are governed by the applicable Creative Commons License

The ISI scores also decreased (i.e., improved) across the three treatment groups from pre- (EMM [SE] = 12.71 [0.19]) to post-treatment (EMM [SE] = 9.26 [0.23]) with a large effect size ($b = -0.29$, 95% CI = −0.34 to −0.24; $t = -11.40$, $df = 376$, $p < 0.001$, $d = 1.18$; Figure 3). The Time × Group interactions testing change in CBT versus SEdu ($p = 0.34$) and change in KY versus SEdu ($p = 0.09$) were not significant, suggesting that the three treatment groups did not differentially improve insomnia symptoms.

3.3 | Aim 2: mediation of treatment effects

The MMLM analyses showed that, on average across treatment groups, the 'a' paths from Time to deviations in the PSWQ, FFMQ, and PSS were all significant, indicating that all three mediators improved significantly over time (Figure 1). The 'b' paths from deviations in the PSWQ and PSS to sleep quality outcomes were also significant, indicating that lower PSWQ and PSS deviations (lower within-person worry and perceived stress) were related to lower (better) scores on sleep quality outcomes within-subjects over time (Figure 1). Higher FFMQ deviations (greater mindfulness) were marginally related to lower (better) sleep quality (Figure 1). As a result, the PSWQ, FFMQ, and PSS deviations were all significant mediators of the effect of Time on sleep quality outcomes (PSWQ: $a \times b = 0.012$, 95% CI = 0.0002–0.023, $p < 0.05$, percent mediated [P_M] = 32.4%; FFMQ: $a \times b$ = 0.007, 95% CI = 0.001–0.013, $p < 0.05$, $P_M = 18.9\%$; PSS: $a \times b = 0.010$, 95% CI = 0.002–0.019, $p < 0.05$, $P_M = 27.0\%$). Thus, together the three mediators accounted for >78% of the total effect of time on sleep quality.

3.4 | Aim 3: moderated mediation

The changes in the PSWQ and PSS (but not FFMQ) were moderated by Treatment Group. The PSWQ decreased significantly from pre- to post-treatment for all three treatments (Figure 1), but the decrease was: (a) greater in CBT than SEdu (difference = −0.06, 95% CI = −0.09 to −0.03; $t = -3.76$, $df = 310$, $p < 0.001$, $d = 0.43$), (b) greater in KY than SEdu (difference = −0.04, 95% CI = −0.07 to −0.003; $t = -2.11$, $df = 310$, $p = 0.035$, $d = 0.24$), and (c) greater in CBT than KY (difference = −0.03, 95% CI = −0.05 to −0.0003; $t = -1.99$, $df = 310$, $p = 0.047$, $d = 0.23$). Hence, the PSWQ was a significant mediator of change in sleep quality in all three treatment groups, but the change attributable to the PSWQ was strongest in CBT ($a \times b$ = 0.015, 95% CI = −0.029 to −0.001, $p < 0.05$, $P_M = 40.5\%$), followed by KY ($a \times b$ = 0.012, 95% CI = −0.024 to −0.01, $p < 0.05$, $P_M = 32.3\%$), and then SEdu ($a \times b$ = 0.008, 95% CI = −0.017 to −0.0001, $p < 0.05$, $P_M = 21.6\%$).

The PSS also decreased significantly from pre- to post-treatment for all three treatments (Figure 1), but the decrease was: (a) greater in CBT than SEdu (difference = −0.06, 95% CI = −0.10 to −0.02, $t = -3.00$, $df = 308$, $p < 0.001$, $d = 0.34$), and (b) greater in KY than SEdu (difference = −0.05, 95% CI = −0.09 to −0.01, $t = -2.65$, $df = 308$, $p = 0.009$, $d = 0.30$), but (c) not different in CBT versus KY. Hence, the PSS was a significant mediator of change in sleep quality in all three treatment groups, but the change attributable to PSS was strongest in CBT ($a \times b$ = 0.011, 95% CI = −0.016 to −0.007, $p < 0.05$, $P_M = 29.7\%$) and KY ($a \times b$ = 0.011, 95% CI = −0.015 to −0.008, $p < 0.05$, $P_M = 29.6\%$) than in SEdu ($a \times b$ = 0.006, 95% CI = −0.013 to −0.001, $p < 0.05$, $P_M = 16.2\%$). See Data S1 for exploratory mediation analyses of two FFMQ subscales (non-judging and the acting with awareness) as alternatives to the FFMQ total score as mediators of sleep quality outcomes.

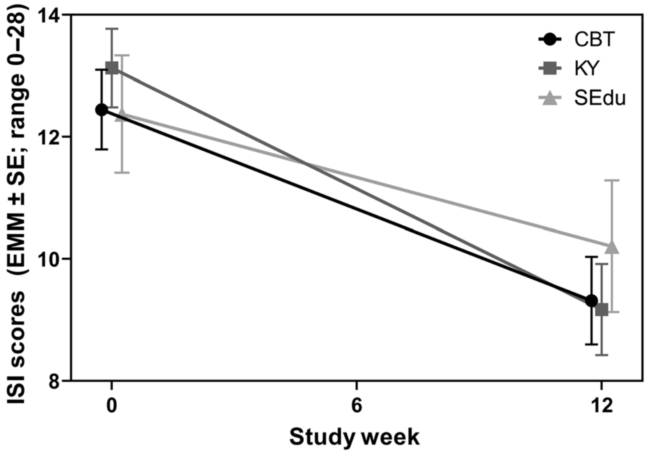

FIGURE 3 Changes in ISI scores over time by treatment group. Figure depicts estimated marginal means (EMM) and standard errors (SEs). CBT, cognitive behavioural therapy; ISI, insomnia severity index; KY, Kundalini yoga; SEdu, stress education. We scaled the graph to clearly show the interaction (full ISI range 0–28; cut-off score = 15 indicates moderate insomnia).

3.5 | Sensitivity analyses

Sensitivity analyses using the subgroup of participants with an ISI score of ≥ 10 at baseline ($n = 142$, 62.8% of the full sample; Morin et al., 2011) were very similar to the results for the full sample (See Data S1).

4 | DISCUSSION

The present secondary data analysis investigation directly compared the efficacy of two group interventions for GAD (CBT and KY) versus an attention control group (SEdu) in improving sleep quality. The sample reported high rates of insomnia (64%, as measured by the ISI), consistent with existing literature (Bélanger et al., 2004). In line with hypotheses, all three interventions, including the SEdu control, led to significant improvements in sleep quality with large effect sizes. These findings are consistent with existing literature demonstrating the efficacy of CBT (Belleville et al., 2010; Bush et al., 2012; Ramsawh et al., 2016), mind–body interventions (e.g., Hoge et al., 2013; Vøllestad et al., 2011), and SEdu (Hoge et al., 2013), for addressing sleep disturbance in GAD, and suggest that each effectively improves co-occurring sleep difficulties in GAD. Nonetheless,

13652869, 2024, 1, Downloaded from https://onlinelibrary.wiley.com/doi/10.1111/jsr.13992 by University Of British Columbia, Wiley Online Library on [30/01/2025]. See the Terms and Conditions (https://onlinelibrary.wiley.com/terms-and-conditions) on Wiley Online Library for rules of use; OA articles are governed by the applicable Creative Commons License

there remained a substantial percentage of patients (32%–41%) who remained above the clinical cut-offs of our sleep quality measures, indicating that more work is needed.

The SEdu control condition may have showed efficacy in improving sleep quality because psychoeducation about sleep and sleep hygiene was taught in the curriculum. Sleep hygiene includes behavioural and environmental recommendations intended to promote sleep and has been associated with improvements in sleep quality (Khalsa & Goldstein, 2021; O'Donnell & Driller, 2017). This information was provided in an educational format and was not personalised to participants, nor were participants instructed to change their sleep habits. And yet, while CBT and KY outperformed SEdu in decreasing symptoms of GAD (Simon et al., 2021), the inclusion of sleep hygiene and other non-specific factors in the SEdu curriculum may have contributed to similar improvements in sleep quality across the three interventions.

When examining specific components of sleep quality (as measured by the PSQI), subjective sleep quality, sleep latency, sleep duration, and daytime dysfunction due to sleep disturbances were the components that specifically improved during treatment. Two past trials examining CBT for GAD also showed improvements in subjective sleep quality and sleep latency (Bush et al., 2012; Ramsawh et al., 2016), but these studies failed to show improvements in sleep duration or daytime dysfunction. Sleep efficiency, sleep disturbances (e.g., due to things like pain or trouble breathing), and frequency of use of sleep medications did not improve significantly in the present study. However, at baseline, use of sleep medications (24.8%) and certain sleep disturbances (e.g., being unable to breathe comfortably, 25.1%) were not frequently reported, perhaps limiting the ability of these aspects to improve.

In line with our mediation hypotheses, reductions in all three constructs, worry ($P_M = 32.4\%$), mindfulness ($P_M = 18.9\%$), and perceived stress ($P_M = 27.0\%$), mediated the effects of change over time in sleep quality outcomes (Total $P_M = 78.3\%$). In line with the hypotheses, the amount of change in sleep quality outcomes attributable to worry was greater in CBT than KY, which was greater than SEdu. This is in line with findings suggesting that: (a) worry is a key mechanism in the formation of sleep problems and pathological worries about sleep maintain insomnia (Åkerstedt et al., 2007; Cousineau et al., 2016; Harvey et al., 2009), and (b) mindfulness meditation reduces emotional reactivity and ruminative thoughts, which contribute to poor sleep quality (Rusch et al., 2019). Contrary to the hypotheses, changes in the FFMQ were not moderated by treatment group. We also found that the amount of change attributable to perceived stress was greater in CBT and KY than in SEdu. Of note, the mechanisms tested are all constructs related to negative affect, and the interventions have overlapping components (e.g., relaxation in CBT, breathing and meditation in yoga), which complicate the interpretation of our moderated mediation analyses.

For patients whose sleep quality did not improve, an important next step would be to continue to test integrative interventions such as Mindfulness Based Therapy for Insomnia (Perini et al., 2021) that includes psychoeducation about sleep physiology and sleep hygiene, targets worry beliefs about sleep via cognitive/mindfulness exercises and addresses perceived stress through meditation and yoga-based practices. Similarly, research has also suggested that treatment of GAD with CBT enhanced with KY may improve sleep (Khalsa et al., 2015). Thus, combined approaches are promising future directions for addressing GAD and sleep comorbidities. Alternatively, meta-analytical findings show that CBT for insomnia demonstrates a moderate effect on comorbid anxiety symptoms (effect size $= 0.41$; Belleville et al., 2011); however, none of the studies included enrolled individuals with GAD specifically, which is a direction for future research. Finally, personalised medicine approaches could determine which treatment approaches (e.g., CBT versus KY) are best for certain patients.

Although a strength of the present study is the longitudinal design, our meditational analyses are correlational (e.g., we cannot infer that reductions in worry cause improvements in sleep quality because we were not able to perform a meaningful time lagged analysis with measures that were only assessed at baseline and post-treatment). Moreover, while we were powered to detect medium effects in this secondary data analysis, smaller effect sizes may have been missed. Another limitation was that sleep quality measures were not administered mid-treatment, which limited our ability to look at more complex mediation models. The relationship between anxiety and sleep disturbance is likely bidirectional (Glidewell et al., 2015; Harvey et al., 2009), such that anxiety/worry may interfere with sleep but also lack of sleep may lead to impaired mood regulation (Babson et al., 2009; Jansson-Fröjmark & Lindblom, 2008). Thus, future studies should include multiple mid-treatment measures of sleep quality in addition to the mediators in order to explore these relationships.

Additionally, the primary outcome measures used (the ISI and PSQI) are both retrospective self-report measures. Although these measures are common, daily diary or sleep logs are the 'gold standard' self-report method for sleep quality outcome data (e.g., Halpern et al., 2014), and a combination of these methods provides an excellent assessment of one's sleep quality (Harvey et al., 2009). Thus, future studies might utilise a daily diary method to obtain information on sleep onset, number of awakenings, total time spent asleep, and subjective sleep quality as it is likely that results from the current trial would have differed across measurement types. Moreover, ecological momentary assessment (EMA) could assess how the relationships between anxiety and sleep quality change pre- to post-treatment. Furthermore, future studies should include objective measures of sleep disturbance such as actigraphy or polysomnography to objectively capture sleep disturbance improvements via behavioural interventions for GAD.

In conclusion, the present study sought to examine the differential efficacy of group CBT and KY compared with a SEdu control for improving sleep quality in GAD and whether worry, mindfulness, and/or perceived stress mediated the effects of treatment on sleep difficulties. Findings suggest that each of these interventions are efficacious for improving sleep quality in individuals with GAD, and that worry, mindfulness, and stress each significantly mediate these effects. The amount of change in sleep outcomes attributable to

13652869, 2024, 1, Downloaded from https://onlinelibrary.wiley.com/doi/10.1111/jsr.13992 by University Of British Columbia, Wiley Online Library on [30/01/2025]. See the Terms and Conditions (https://onlinelibrary.wiley.com/terms-and-conditions) on Wiley Online Library for rules of use; OA articles are governed by the applicable Creative Commons License

worry (CBT > KY > SEdu) and perceived stress (CBT, KY > SEdu) was moderated by treatment group. Future treatment studies should include intensive time series methods (e.g., daily sleep diaries, EMA) and objective measures (e.g., actigraphy, polysomnography).

AUTHOR CONTRIBUTIONS

Ryan Jane Jacoby: Conceptualization; writing – original draft; writing – review and editing. **Mackenzie L Brown:** Conceptualization; investigation; writing – original draft; writing – review and editing. **Sarah T Wieman:** Conceptualization; investigation; project administration; writing – original draft; writing – review and editing. **David Rosenfield:** Formal analysis; methodology; visualization; writing – original draft; writing – review and editing. **Susanne S Hoeppner:** Methodology; visualization; writing – review and editing. **Eric Bui:** Investigation; supervision; writing – review and editing. **Elizabeth A Hoge:** Conceptualization; funding acquisition; methodology; supervision; writing – review and editing. **Sat Bir S Khalsa:** Conceptualization; funding acquisition; methodology; supervision; writing – review and editing. **Stefan G Hofmann:** Conceptualization; funding acquisition; investigation; methodology; resources; supervision; writing – review and editing. **Naomi M Simon:** Conceptualization; funding acquisition; investigation; methodology; resources; supervision; writing – review and editing.

CONFLICT OF INTEREST STATEMENT

Research reported in this publication was supported by the National Center for Complementary and Integrative Health of the National Institutes of Health (NIH) awarded to the principal investigators Drs Hofmann and Simon under award numbers R01AT007257 and R01AT007258. The content is solely the responsibility of the authors and does not necessarily represent the official views of the NIH. Dr Jacoby has received salary and research support from the National Institute of Mental Health (NIMH; K23MH120351). She is also paid as a faculty member of the Massachusetts General Hospital Psychiatry Academy. She receives book royalties from Hogrefe Publishing. Dr Rosenfield received grants and/or consulting fees from the National Center for Complementary and Integrative Health (NCCIH), NIH, the Department of Defence, and Rosenfield Analytics during the conduct of the study. Dr Bui received grants from the NIH during the conduct of the study and received royalties from Springer and from Wolters Kluwer, personal fees from Cerevel Therapeutics LLC, and grants from the US Department of Defence, Osher Center for Integrative Medicine, Patient-Centred Outcomes Research Institute, and Elizabeth Dole Foundation outside the submitted work. Dr Khalsa received grants from the NCCIH and NIH during the conduct of the study; received grants and personal fees from Kundalini Research Institute and grants from Kripalu Center for Yoga and Health outside the submitted work; and is a practitioner and certified instructor in KY as taught by Yogi Bhajan. He receives book royalties from Handspring Publishing. Dr Hofmann receives financial support by the Alexander von Humboldt Foundation (as part of the Alexander von Humboldt Professor), the Hessische Ministerium für Wissenschaft und Kunst (as part of the LOEWE Spitzenprofessur), NIH/NIMH R01MH128377, NIH/NIMHU01MH108168, Broderick Foundation/MIT, and the James S. McDonnell Foundation 21st Century Science Initiative in Understanding Human Cognition—Special Initiative. He receives compensation for his work as editor from Springer Nature. He also receives royalties and payments for his work from various publishers. Dr Simon in the past 2 years received grants from the NIH during the conduct of the study; received grant funding from the American Foundation for Suicide Prevention, Patient-Centred Outcomes Research Institute, Ananda Scientific, and support from the Cohen Veteran's Network; received personal fees from Cerevel, Vanda Pharmaceuticals, Engrail, Praxis Therapeutics, Bionomics Limited, Genomind, and Wiley; performed grant reviews for American Foundation for Suicide Prevention; receiving royalties for UpToDate contributions from Wolters Kluwer and APA Publishing (Textbook of Anxiety, Trauma and OCD Related Disorders 2020); and has spousal stock from G1 Therapeutics and Zentalis outside the submitted work. All other authors report no conflicts of interest.

DATA AVAILABILITY STATEMENT

The data underlying this article cannot be shared publicly due to the privacy of individuals that participated in the study. At the time of data collection, the IRB consent form did not allow for release of individual patient data.

ORCID

Ryan J. Jacoby https://orcid.org/0000-0001-9154-5580

REFERENCES

Åkerstedt, T., Kecklund, G., & Axelsson, J. (2007). Impaired sleep after bedtime stress and worries. *Biological Psychology*, *76*(3), 170–173. https://doi.org/10.1016/j.biopsycho.2007.07.010

American Psychiatric Association. (2013). *Diagnostic and statistical manual of mental disorders* (5th ed.). https://doi.org/10.1176/appi.books.9780890425596

Babson, K. A., Feldner, M. T., Trainor, C. D., & Smith, R. C. (2009). An experimental investigation of the effects of acute sleep deprivation on panic-relevant biological challenge responding. *Behavior Therapy*, *40*(3), 239–250. https://doi.org/10.1016/j.beth.2008.06.001

Backhaus, J., Junghanns, K., Broocks, A., Riemann, D., & Hohagen, F. (2002). Test–retest reliability and validity of the Pittsburgh sleep quality index in primary insomnia. *Journal of Psychosomatic Research*, *4*, 737–740.

Baer, R. A., Smith, G. T., Hopkins, J., Krietemeyer, J., & Toney, L. (2006). Using self-report assessment methods to explore facets of mindfulness. *Assessment*, *13*(1), 27–45. https://doi.org/10.1177/1073191105283504

Baer, R. A., Smith, G. T., Lykins, E., Button, D., Krietemeyer, J., Sauer, S., Walsh, E., Duggan, D., & Williams, J. M. G. (2008). Construct validity of the five facet mindfulness questionnaire in meditating and nonmeditating samples. *Assessment*, *15*(3), 329–342. https://doi.org/10.1177/1073191107313003

Bastien, C. H., Vallières, A., & Morin, C. M. (2001). Validation of the insomnia severity index as an outcome measure for insomnia research. *Sleep Medicine*, *2*(4), 297–307. https://doi.org/10.1016/s1389-9457(00)00065-4

Bélanger, L., Morin, C. M., Langlois, F., & Ladouceur, R. (2004). Insomnia and generalized anxiety disorder: Effects of cognitive behavior therapy for GAD on insomnia symptoms. *Journal of Anxiety Disorders*, *18*(4), 561–571. https://doi.org/10.1016/S0887-6185(03)00031-8

Belleville, G., Cousineau, H., Levrier, K., & St-Pierre-Delorme, M.-È. (2011). Meta-analytic review of the impact of cognitive-behavior therapy for

13652869, 2024, 1, Downloaded from https://onlinelibrary.wiley.com/doi/10.1111/jsr.13992 by University Of British Columbia, Wiley Online Library on [30/01/2025]. See the Terms and Conditions (https://onlinelibrary.wiley.com/terms-and-conditions) on Wiley Online Library for rules of use; OA articles are governed by the applicable Creative Commons License

insomnia on concomitant anxiety. *Clinical Psychology Review*, *31*(4), 638–652. https://doi.org/10.1016/j.cpr.2011.02.004

Belleville, G., Cousineau, H., Levrier, K., St-Pierre-Delorme, M.-E., & Marchand, A. (2010). The impact of cognitive-behavior therapy for anxiety disorders on concomitant sleep disturbances: A meta-analysis. *Journal of Anxiety Disorders*, *24*(4), 379–386. https://doi.org/10.1016/j.janxdis.2010.02.010

Benz, F., Knoop, T., Ballesio, A., Bacaro, V., Johann, A. F., Rücker, G., Feige, B., Riemann, D., & Baglioni, C. (2020). The efficacy of cognitive and behavior therapies for insomnia on daytime symptoms: A systematic review and network meta-analysis. *Clinical Psychology Review*, *80*, 101873. https://doi.org/10.1016/j.cpr.2020.101873

Boland, E. M., & Ross, R. J. (2015). Recent advances in the study of sleep in the anxiety disorders, obsessive-compulsive sisorder, and posttraumatic stress disorder. *The Psychiatric Clinics of North America*, *38*(4), 761–776. https://doi.org/10.1016/j.psc.2015.07.005

Brenes, G. A., Divers, J., Miller, M. E., Anderson, A., Hargis, G., & Danhauer, S. C. (2020). Comparison of cognitive-behavioral therapy and yoga for the treatment of late-life worry: A randomized preference trial. *Depression and Anxiety*, *37*(12), 1194–1207. https://doi.org/10.1002/da.23107

Brenes, G. A., Miller, M. E., Stanley, M. A., Williamson, J. D., Knudson, M., & McCall, W. V. (2009). Insomnia in older adults with generalized anxiety disorder. *The American Journal of Geriatric Psychiatry*, *17*(6), 465–472. https://doi.org/10.1097/JGP.0b013e3181987747

Brosschot, J. F., Van Dijk, E., & Thayer, J. F. (2007). Daily worry is related to low heart rate variability during waking and the subsequent nocturnal sleep period. *International Journal of Psychophysiology*, *63*, 39–47. https://doi.org/10.1016/j.ijpsycho.2006.07.016

Brown, T. A., & Barlow, D. H. (2014). *Anxiety and related disorders interview schedule for DSM-5 (ADIS-5).*— OUP USA Treatments That Work.

Bush, A. L., Armento, M. E. A., Weiss, B. J., Rhoades, H. M., Novy, D. M., Wilson, N. L., Kunik, M. E., & Stanley, M. A. (2012). The Pittsburgh sleep quality index in older primary care patients with generalized anxiety disorder: Psychometrics and outcomes following cognitive behavioral therapy. *Psychiatry Research*, *199*(1), 24–30. https://doi.org/10.1016/j.psychres.2012.03.045

Buysse, D. J., Reynolds, C. F., III, Monk, T. H., Berman, S. R., & Kupfer, D. J. (1989). The Pittsburgh sleep quality index: A new instrument for psychiatric practice and research. *Psychiatry Research*, *28*(2), 193–213.

Carpenter, J. K., Andrews, L. A., Witcraft, S. M., Powers, M. B., Smits, J. A. J., & Hofmann, S. G. (2018). Cognitive behavioral therapy for anxiety and related disorders: A meta-analysis of randomized placebo-controlled trials. *Depression and Anxiety*, *35*(6), 502–514. https://doi.org/10.1002/da.22728

Carpenter, J. K., Conroy, K., Gomez, A. F., Curren, L. C., & Hofmann, S. G. (2019). The relationship between trait mindfulness and affective symptoms: A metaanalysis of the five facet mindfulness questionnaire (FFMQ). *Clinical Psychology Review*, *74*, 101785. https://doi.org/10.1016/j.cpr.2019.101785

Carpenter, J. S., & Andrykowski, M. A. (1998). Psychometric evaluation of the Pittsburgh sleep quality index. *Journal of Psychosomatic Research*, *45*(1), 5–13. https://doi.org/10.1016/S0022-3999(97)00298-5

Cohen, S., Kamarck, T., & Mermelstein, R. (1983). A global measure of perceived stress. *Journal of Health and Social Behavior*, *24*(4), 385–396. https://doi.org/10.2307/2136404

Cousineau, H., Marchand, A., Bouchard, S., Bélanger, C., Gosselin, P., Langlois, F., Labrecque, J., Dugas, M. J., & Belleville, G. (2016). Insomnia symptoms following treatment for comorbid panic disorder with agoraphobia and generalized anxiety disorder. *Journal of Nervous and Mental Disease*, *204*, 267–273. https://doi.org/10.1097/NMD.0000000000000466

First, M., Spitzer, R., Gibbon, M., & Williams, J. (2002). *Structured clinical interview for DSM-IV-TR Axis I disorders, research version, patient edition (SCID-I/P)*. Biometrics Research.

Fritz, M. S., & MacKinnon, D. P. (2007). Required sample size to detect the mediated effect. *Psychological Science*, *18*(3), 233–239. https://doi.org/10.1111/j.1467-9280.2007.01882.x

Glidewell, R. N., McPherson Botts, E., & Orr, W. C. (2015). Insomnia and anxiety: Diagnostic and management implications of complex interactions. *Sleep Medicine Clinics*, *10*(1), 93–99. https://doi.org/10.1016/j.jsmc.2014.11.008

Guy, W. (1976). Clinical Global Impressions. In *ECDEU assessment manual for psychopharmacology—Revised* (pp. 218–222). National Institute of Mental Health. DHEW Pub No. ADM 76-338.

Halpern, J., Cohen, M., Kennedy, G., Reece, J., Cahan, C., & Baharav, A. (2014). Yoga for improving sleep quality and quality of life for older adults. *Alternative Therapies in Health and Medicine*, *20*(3), 37–46.

Harvey, A. G., Hairston, I. S., Gruber, J., & Gershon, A. (2009). Anxiety and sleep. In M. M. Antony & M. B. Stein (Eds.), *Oxford handbook of anxiety and related disorders* (pp. 611–621). Oxford University Press; APA PsycInfo.

Hofmann, S. G., Curtiss, J., Khalsa, S. B. S., Hoge, E., Rosenfield, D., Bui, E., Keshaviah, A., & Simon, N. (2015). Yoga for generalized anxiety disorder: Design of a randomized controlled clinical trial. *Contemporary Clinical Trials*, *44*, 70–76. https://doi.org/10.1016/j.cct.2015.08.003

Hoge, E. A., Bui, E., Marques, L., Metcalf, C. A., Morris, L. K., Robinaugh, D. J., Worthington, J. J., Pollack, M. H., & Simon, N. M. (2013). Randomized controlled trial of mindfulness meditation for generalized anxiety disorder: Effects on anxiety and stress reactivity. *The Journal of Clinical Psychiatry*, *74*(8), 786–792. https://doi.org/10.4088/JCP.12m08083

Hox, J. J., Moerbeek, M., & Schoot, R. v. d. (2018). *Multilevel analysis: Techniques and applications* (3rd ed.). Routledge.

Ibáñez del Prado, C., & Cruzado, J. A. (2020). A screening method for sleep disturbances at the end-of-life. *Palliative and Supportive Care*, *18*(4), 468–472. https://doi.org/10.1017/S1478951520000024

Jansson-Fröjmark, M., & Lindblom, K. (2008). A bidirectional relationship between anxiety and depression, and insomnia? A prospective study in the general population. *Journal of Psychosomatic Research*, *64*(4), 443–449. https://doi.org/10.1016/j.jpsychores.2007.10.016

Jeter, P. E., Slutsky, J., Singh, N., & Khalsa, S. B. S. (2015). Yoga as a therapeutic intervention: A bibliometric analysis of published research studies from 1967 to 2013. *The Journal of Alternative and Complementary Medicine*, *21*(10), 586–592. https://doi.org/10.1089/acm.2015.0057

Khalsa, M. K., Greiner-Ferris, J. M., Hofmann, S. G., & Khalsa, S. B. S. (2015). Yoga-enhanced cognitive behavioural therapy (Y-CBT) for anxiety management: A pilot study. *Clinical Psychology & Psychotherapy*, *22*(4), 364–371. https://doi.org/10.1002/cpp.1902

Khalsa, S. B. S., & Goldstein, M. R. (2021). Treatment of chronic primary sleep onset insomnia with kundalini yoga: A randomized controlled trial with active sleep hygiene comparison. *Journal of Clinical Sleep Medicine*, *17*(9), 1841–1852. https://doi.org/10.5664/jcsm.9320

Morin, C. M., Belleville, G., Bélanger, L., & Ivers, H. (2011). The insomnia severity index: Psychometric indicators to detect insomnia cases and evaluate treatment response. *Sleep*, *34*(5), 601–608. https://doi.org/10.1093/sleep/34.5.601

Morin, C. M., Rodrigue, S., & Ivers, H. (2003). Role of stress, arousal, and coping skills in primary insomnia. *Psychosomatic Medicine*, *65*(2), 259–267.

O'Donnell, S., & Driller, M. W. (2017). Sleep-hygiene education improves sleep indices in elite female athletes. *International Journal of Exercise Science*, *10*(4), 522–530.

Ohayon, M. M., & Roth, T. (2003). Place of chronic insomnia in the course of depressive and anxiety disorders. *Journal of Psychiatric Research*, *37*(1), 9–15. https://doi.org/10.1016/S0022-3956(02)00052-3

Ong, J., & Kalmbach, D. (2023). Mindfulness as an adjunct or alternative to CBT-I. *Sleep Medicine Clinics*, *18*(1), 59–71. https://doi.org/10.1016/j.jsmc.2022.09.002

Perini, F., Wong, K. F., Lin, J., Hassirim, Z., Ong, J. L., Lo, J., Ong, J. C., Doshi, K., & Lim, J. (2021). Mindfulness-based therapy for insomnia for

13652869, 2024, 1, Downloaded from https://onlinelibrary.wiley.com/doi/10.1111/jsr.13992 by University Of British Columbia, Wiley Online Library on [30/01/2025]. See the Terms and Conditions (https://onlinelibrary.wiley.com/terms-and-conditions) on Wiley Online Library for rules of use; OA articles are governed by the applicable Creative Commons License

older adults with sleep difficulties: A randomized clinical trial. *Psychological Medicine*, *1–11*, 1038–1048. https://doi.org/10.1017/S0033291721002476

Ramsawh, H. J., Bomyea, J., Stein, M. B., Cissell, S. H., & Lang, A. J. (2016). Sleep quality improvement during cognitive behavioral therapy for anxiety disorders. *Behavioral Sleep Medicine*, *14*(3), 267–278. https://doi.org/10.1080/15402002.2014.981819

Rusch, H. L., Rosario, M., Levison, L. M., Olivera, A., Livingston, W. S., Wu, T., & Gill, J. M. (2019). The effect of mindfulness meditation on sleep quality: A systematic review and meta-analysis of randomized controlled trials. *Annals of the new York Academy of Sciences*, *1445*(1), 5–16. https://doi.org/10.1111/nyas.13996

Ruscio, A. M., Hallion, L. S., Lim, C. C. W., Aguilar-Gaxiola, S., Al-Hamzawi, A., Alonso, J., Andrade, L. H., Borges, G., Bromet, E. J., Bunting, B., Caldas de Almeida, J. M., Demyttenaere, K., Florescu, S., de Girolamo, G., Gureje, O., Haro, J. M., He, Y., Hinkov, H., Hu, C., … Scott, K. M. (2017). Cross-sectional comparison of the epidemiology of DSM-5 generalized anxiety disorder across the globe. *JAMA Psychiatry*, *74*(5), 465–475. https://doi.org/10.1001/jamapsychiatry.2017.0056

Sánchez-Ortuño, M. M., & Edinger, J. D. (2012). Internight sleep variability: Its clinical significance and responsiveness to treatment in primary and comorbid insomnia. *Journal of Sleep Research*, *21*, 527–534. https://doi.org/10.1111/j.1365-2869.2012.01010.x

Simon, N. M., Hofmann, S. G., Rosenfield, D., Hoeppner, S. S., Hoge, E. A., Bui, E., & Khalsa, S. B. S. (2021). Efficacy of yoga vs cognitive behavioral therapy vs stress education for the treatment of generalized anxiety disorder: A randomized clinical trial. *JAMA Psychiatry*, *78*(1), 13–20. https://doi.org/10.1001/jamapsychiatry.2020.2496

Stöber, J., & Bittencourt, J. (1998). Weekly assessment of worry: An adaptation of the Penn State worry questionnaire for monitoring changes during treatment. *Behaviour Research and Therapy*, *36*(6), 645–656. https://doi.org/10.1016/s0005-7967(98)00031-x

Tofighi, D., & MacKinnon, D. P. (2011). RMediation: An R package for mediation analysis confidence intervals. *Behavior Research Methods*, *43*(3), 692–700. https://doi.org/10.3758/s13428-011-0076-x

Vøllestad, J., Sivertsen, B., & Nielsen, G. H. (2011). Mindfulness-based stress reduction for patients with anxiety disorders: Evaluation in a randomized controlled trial. *Behaviour Research and Therapy*, *49*(4), 281–288. https://doi.org/10.1016/j.brat.2011.01.007

Wang, F., Eun-Kyoung Lee, O., Feng, F., Vitiello, M. V., Wang, W., Benson, H., Fricchione, G. L., & Denninger, J. W. (2016). The effect of meditative movement on sleep quality: A systematic review. *Sleep Medicine Reviews*, *30*, 43–52. https://doi.org/10.1016/j.smrv.2015.12.001

Wang, L., & Maxwell, S. E. (2015). On disaggregating between-person and within-person effects with longitudinal data using multilevel models. *Psychological Methods*, *20*(1), 63–83. https://doi.org/10.1037/met0000030

Wang, X., Li, P., Pan, C., Dai, L., Wu, Y., & Deng, Y. (2019). The effect of mind-body therapies on insomnia: A systematic review and meta-analysis. *Evidence-Based Complementary and Alternative Medicine*, *17*, 9359807. https://doi.org/10.1155/2019/9359807

SUPPORTING INFORMATION

Additional supporting information can be found online in the Supporting Information section at the end of this article.

How to cite this article: Jacoby, R. J., Brown, M. L., Wieman, S. T., Rosenfield, D., Hoeppner, S. S., Bui, E., Hoge, E. A., Khalsa, S. B. S., Hofmann, S. G., & Simon, N. M. (2024). Effect of cognitive behavioural therapy and yoga for generalised anxiety disorder on sleep quality in a randomised controlled trial: the role of worry, mindfulness, and perceived stress as mediators. *Journal of Sleep Research*, *33*(1), e13992. https://doi.org/10.1111/jsr.13992

13652869, 2024, 1, Downloaded from https://onlinelibrary.wiley.com/doi/10.1111/jsr.13992 by University Of British Columbia, Wiley Online Library on [30/01/2025]. See the Terms and Conditions (https://onlinelibrary.wiley.com/terms-and-conditions) on Wiley Online Library for rules of use; OA articles are governed by the applicable Creative Commons License
