# Supplementary material for: Does physical activity-based intervention decrease repetitive negative thinking? A systematic review
Source: PLoS One. 2025 Apr 1;20(4):e0319806. doi: 10.1371/journal.pone.0319806 (PMC11960971; doi:10.1371/journal.pone.0319806)
Supplement: S1 File — https://doi.org/10.6084/m9.figshare.25711734. (ZIP) [file pone.0319806.s001.zip › supporting information/paper file/de Bruin 2016.pdf]

Mindfulness (2016) 7:1182–1192
DOI 10.1007/s12671-016-0561-5

ORIGINAL PAPER

A RCT Comparing Daily Mindfulness Meditations, Biofeedback Exercises, and Daily Physical Exercise on Attention Control, Executive Functioning, Mindful Awareness, Self-Compassion, and Worrying in Stressed Young Adults

Esther I. de Bruin[1] **· J. Esi van der Zwan**[2] **· Susan M. Bögels**[1,3]

Published online: 2 July 2016
© The Author(s) 2016. This article is published with open access at Springerlink.com

Abstract Our Western society is characterized by multitasking, competition, and constant time pressure. Negative effects of stress for the individual (anxiety, depression, somatic complaints) and for organizations and society (costs due to work absence) are very high. Thus, time-efficient self-help interventions to address these issues are necessary. This study assessed the effects of daily mindfulness meditations (MM) versus daily heart rate variability biofeedback (HRV-BF) and daily physical exercise (PE) on attention control, executive functioning, mindful awareness, self-compassion, and worrying. Young adults ($n = 75$, age range 18 to 40) with elevated stress levels were randomized to MM, HRV-BF, or PE, and measurements were taken at pre-test, post-test, and follow-up. Interventions in all three groups were self-guided and lasted for 5 weeks. Generalized estimating equation analyses showed that overall, all three interventions were effective and did not differ from each other. However, practice time differed between groups, with participants in the PE group practicing much more than participants in the other two groups. Therefore, additional analyses were carried out in two subsamples. The optimal dose sample included only those participants who practiced for at least 70 % of the total prescribed time. In the equal dose sample, home practice intensity was equal for all three groups. Again, the effects of the three interventions did not differ. In conclusion, MM, HRV-BF, and PE are all effective self-help methods to improve attention control, executive functioning, mindful awareness, self-compassion, and worrying, and mindfulness meditation was not found to be more effective than HRV-biofeedback or physical exercise for these cognitive processes.

Keywords Mindfulness meditation · HRV-biofeedback · Physical exercise · Attention control · Executive functioning · Mindful awareness · Self-compassion · Worrying

Esther I. de Bruin and J. Esi van der Zwan contributed equally to this work.

✉ Esther I. de Bruin
e.i.debruin@uva.nl

1 University of Amsterdam, Research Institute of Child Development and Education (RICDE), Research Priority Area Yield, Nieuwe Achtergracht 127, 1018, WS Amsterdam, The Netherlands

2 Department of Clinical Developmental Psychology and EMGO Institute for Health and Care Research, Vrije Universiteit Amsterdam, Van der Boechorststraat 1, 1081, BT Amsterdam, The Netherlands

3 UvA Minds, Academic Outpatient Child and Adolescent Treatment Center of the University of Amsterdam, Plantage Muidergracht 14, 1018, TV Amsterdam, The Netherlands

Introduction

Our Western society is characterized by multitasking, speed, competition, and constant time pressure. Although these aspects of life might be inspiring, they also lead to stress. Being exposed to brief stressful periods may lead to complaints such as headaches, muscle pains, sleep problems, mood instability, and flu-like symptoms, which may affect daily functioning. Moreover, being exposed to chronic stress or being unable to effectively cope with stress may ultimately lead to, for instance, states of depression and anxiety, burnout, suicide attempts, poor immune functioning, and cardiovascular disorders (e.g., Cohen et al. 2012; Netterstrøm et al. 2008; Stansfeld and Candy 2006).

Besides these high "personal costs," organizational and societal costs of stress (e.g., costs due to work absenteeism and health care costs), are extremely high. In the USA, annual estimated societal costs of stress are around $300 billion (e.g.,

Rosch 2001). As for Europe, costs of work-related depression for instance were estimated to be around €617 billion per year (European Agency for Safety and Health at Work 2009).

Since stress seems to be an integral part of our current Western society, and negative health consequences are enormous, cost-effective interventions that reduce stress and its effect on daily functioning are of great value, particularly methods that support self-regulatory healing capacities of the mind-body system.

Mindfulness-based interventions (MBIs) are widely applied across the world, and the evidence for the positive effects is rising. This can, for instance, be seen by the 40-fold increase in mindfulness publications between the 1990s and 2010 (Harnett and Dawe 2012). Mindfulness originally stems from the 2500-year-old Buddhist tradition (Kabat-Zinn 2003). Mindfulness in this study is defined as non-reactive and non-judgmental, present-moment awareness and attention for internal and external stimuli as they arise. Mindfulness has been shown to have positive effects in the treatment of chronic pain patients (Kabat-Zinn et al. 1985), leads to a better immune system (Davidson et al. 2003), is positively related to mental well-being (Carmody and Baer 2008), inversely related to stress and mood disorders (Brown and Ryan 2003), and decreases depression and anxiety in clinical populations (Hoffman et al. 2010). Further, in meta-analyses in non-clinical groups, it was found that meditation had its largest effects in the reduction of negative emotion (Sedlmeier et al. 2012).

In addition to positive effects on stress and (symptoms of) anxiety and depression, MBIs may also alter cognitive processes, such as attention control, executive functioning, mindful awareness, self-compassion, and rumination or worrying. Such cognitive improvements may be important in its own right, but may also explain why MBIs reduce stress, anxiety, and depression, as they may be caused and maintained by these cognitive processes (e.g., Caspi et al. 2014). There is evidence to suggest that MBIs improve attention control and executive functioning (e.g., shifting, initiating, monitoring, and behavior regulation) in children as well as in adults (Chiesa et al. 2011; Flook et al. 2010; Slagter et al. 2007). MBIs were also found to increase mindful awareness and self-compassion and reduce rumination and worrying (Chiesa and Serretti 2009; Kuyken et al. 2010; Robins et al. 2012).

Positive effects on stress-related symptoms are not only found for MBIs; for instance, another promising intervention is heart rate variability (HRV)-biofeedback. With HRV-biofeedback, one tries to maximize the variability of one's heart rate during exercises, by adjusting the breathing pace to approximately six breaths per minute, i.e., the resonance frequency (Vaschillo et al. 2006). Some aspects of HRV-biofeedback overlap with mindfulness meditation, namely, focusing your attention on the breathing, here and now, while breathing in a slow pace.

HRV-biofeedback has been found to decrease stress, anxiety, and depression in non-clinical populations (Henriques et al. 2011; Ratanasiripong et al. 2012) as well as in clinical samples with anxiety disorders or depression (Reiner 2008; Siepmann et al. 2008) and to reduce post-traumatic stress disorder (PTSD) and insomnia symptoms (Zucker et al. 2009). Furthermore, HRV-biofeedback has a positive effect on cardiovascular problems such as pre-hypertension (Lin et al. 2012), and it reduces asthmatic symptoms (Lehrer et al. 2004, 2006). Relatively little is known about the effect of HRV-biofeedback on cognitive processes, although research has shown that a single short session of HRV-biofeedback improves cognitive performance on a modified Stroop task (Prinsloo et al. 2011). One may expect that HRV-biofeedback could also have beneficial effects on cognitive processes such as executive functioning and mindful awareness because HRV-biofeedback requires an attentional focus and therefore bears similarities to mindfulness meditations.

Further, physical exercise has also been shown to reduce symptoms of anxiety and depression among non-clinical groups (Conn 2010a, b) and also in, for instance, individuals suffering from depression or anxiety disorders (Jazaieri et al. 2012; Lawlor and Hopker 2001; Stathopoulous et al. 2006). Additionally, exercise has a positive effect on physical well-being, such as reductions in the risk for breast cancer, obesity, and heart disease (Powell et al. 2011), and it improves cognitive and executive functioning (Best 2010). A recent randomized controlled trial (RCT) showed that regular physical exercise also increased dispositional mindfulness in healthy men (Mothes et al. 2014). As physical exercise "forces" one to focus attention on the body and on the here and now, and leaves little space for rumination, physical exercise may have similar effects to meditation.

Mindfulness meditation, HRV-biofeedback, and physical exercise thus all seem to have beneficial effects with respect to stress, anxiety, and depression and possible positive effects on cognitive processes. However, to the best of the authors' knowledge, effects of these interventions on cognitive functioning have not been compared yet. Therefore, the main aim of the current RCT was to compare, in a sample of at least mildly stressed adults, the effects of daily meditations (a self-help format of MBI) to those of daily HRV-biofeedback and daily physical exercise (both also delivered in a self-help format) on a series of cognitive processes, that is, attention control, executive functioning, mindful awareness, self-compassion, and worrying. We hypothesized that mindfulness meditations would result in stronger effects, given that these cognitive processes are explicitly targeted in MBIs.

Method

Participants

Participants in this study were eligible when they were aged between 18 and 40 years and had a score on the Perceived Stress Scale (PSS; Cohen et al. 1983), which was higher than at least one standard deviation below the normative mean (17). This cutoff score is based on the article of Cohen and Janicki-Deverts (2012). Participants in the mindfulness meditation (MM) group ($n = 27$, of whom $n = 20$ females) had a mean PSS score of 24.22 (SD = 4.69), those in the HRV-biofeedback (HRV-BF) group ($n = 25$, of whom $n = 17$ females) had a mean PSS score of 23.24 (SD = 3.43), and those in the physical exercise (PE) group ($n = 23$, of whom $n = 18$ females) had a mean PSS score of 23.61 (SD = 4.31). Groups did not differ in stress level as measured by the PSS, $F(2,72) = 0.37$, $p = 0.70$, and the number of males and females did not differ between groups, χ^2 (2) = 0.66, $p = 0.72$. Additionally, age in the MM group ($M = 26.32$, SD = 5.03), the HRV-BF group ($M = 26.99$, SD = 6.53), and the PE group ($M = 25.28$, SD = 4.42) did not differ significantly, $H(2) = 0.43$, $p = 0.81$. Fifty-one participants reported exercising regularly before the start of the study, with an average exercise time of 205.26 min/week in the MM group, 162.81 min/week in the HRV-BF group, and 143.93 min/week in the PE group. No differences were found between the MM, HRV-BF, and PE groups in whether participants exercised or not, χ^2 (2) = 0.72, $p = 0.70$, and for how long they exercised if they did, $H(2) = 1.98$, $p = 0.37$. Thirty-eight participants reported having meditated at least once before, of whom four reported to meditate more than once a month, and four reported to meditate regularly, but less than once a month. No differences were found between groups for having meditated before, χ^2 (2) = 3.19, $p = 0.20$, or meditating regularly, χ^2 (4) = 4.25, $p = 0.37$. Only two participants reported ever having tried HRV-biofeedback, and one of them used a biofeedback device regularly, but neither of them was allocated to the HRV-BF group. Lastly, participants in the three groups did not differ on any of the cognitive processes at pre-test (all p values >0.34). Highest attained school levels were the following: 6.7 % higher general secondary education, 21.3 % pre-university education, 8 % intermediate vocational education, 21.3 % higher vocational education, 40 % university, and 2.7 % other education. Groups did not differ in educational level, χ^2 (10) = 10.20, $p = 0.42$. Participants were excluded if they were pregnant or had insufficient understanding of the Dutch language.

Procedure

Potential participants ($n = 126$) were randomized to either MM, HRV-BF, or PE upon signing up. After receiving additional information on the study, people who were interested in participating ($n = 95$) signed informed consent. The cognitive processes of attention control, executive functioning, mindful awareness, self-compassion, and worrying were assessed at pre-test, post-test, and follow-up 6 weeks later. Results on the main outcome measures (stress-related symptoms) of this trial are described elsewhere (Van der Zwan et al. 2015). Participants were kept blind to which intervention they were allocated until after pre-test. See Fig. 1 for a CONSORT diagram of participant flow.

Out of the 95 participants, 19 (20 %) dropped out of the study after pre-test. Dropouts did not differ significantly from the other participants with respect to stress level, age, attentional control, executive functioning, mindful awareness, self-compassion, or worrying at pre-test (all p values >0.06). Only one participant reported not being satisfied with the allocated treatment as a main reason to drop out; therefore, we do not expect that dropout was affected by dissatisfaction with the allocated treatment. In fact, most dropouts ($n = 15$) reported either having time issues as the main reason for dropping out, or they could not attend the obligatory orientation session, which could also imply time constraints.

Participants in all three conditions started with a 2-h group orientation session which consisted of three parts. First, general information about stress and how stress affects our mental well-being was provided. Second, the specific effects of MM, HRV-BF, or PE on well-being were explained. The third part of the session consisted of experiential practice. In the MM group, participants practiced meditations (e.g., the raisin exercise, body scan, sitting with the breath, walking meditation, plus short inquiries), guided by a licensed mindfulness trainer, in a meditation room of UvA minds, the outpatient mental health care clinic for parents and children, adjacent to the University of Amsterdam. In the HRV-BF group, participants practiced using a handheld HRV-biofeedback device, the StressEraser (a 510[k] premarket notification exempt, class II medical device; Helicor, NY). Furthermore, they practiced abdominal breathing, which is recommended for applying HRV-biofeedback. The orientation session was guided by two experts in HRV-biofeedback, in a meeting room of the University of Amsterdam. In the PE group, participants practiced spinning (indoor cycling on a home trainer), with a professional spinning teacher, in a sport center of the University of Amsterdam. With each participant, an individual practice plan for the following week was made with one of the research assistants, and practice started the day after the orientation sessions. In the following weeks, practice plans were filled out online per week by each individual participant.

After the last measurement, 50-euro vouchers were randomly distributed among the participants as a reward. The Ethics Committee of the University of Amsterdam approved this study (number COP-2503).

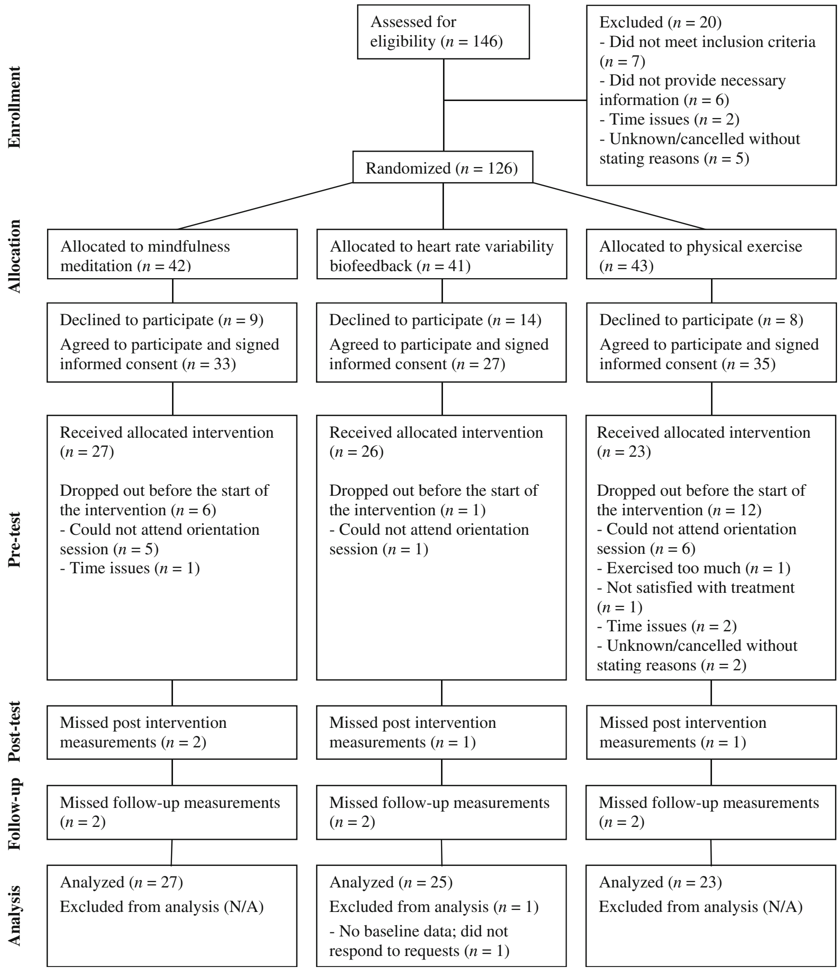

Fig. 1 Consolidated Standards of Reporting Trial (CONSORT) diagram for a RCT of mindfulness meditation, heart rate variability biofeedback, and physical exercise. Adapted with permission from "Physical activity, mindfulness meditation, or heart rate variability biofeedback for stress reduction: a randomized controlled trial" by Van der Zwan et al. (2015), Applied Psychophysiology and Biofeedback. Copyright 2015 by the authors

All participants practiced daily for a 5-week period. In the first week, they practiced 10 min a day; in the second week, this was increased to 15 min; and during the last 3 weeks, participants practiced 20 min a day, so practice time was kept the same across the three interventions. All participants kept a daily log of how long they practiced and received daily reminders by WhatsApp or text message to optimize motivation and compliance. They further received a weekly phone call to see how they were doing and to answer any questions if necessary.

In the MM group, participants were given a CD with the meditations for their daily practice. The homework followed a standardized protocol over the following 5-week intervention: week 1, breathing meditation, mindful eating (beginner's mind), and routine activity with full awareness; week 2, body scan; week 3, breathing meditation, body meditation, mindful movement, and 3-min breathing space; week 4, breathing meditation, body meditation, sound meditation, and thought meditation; and week 5, participant's own choice mediations. The rationale and meditations were based on the book of Williams and Penman (2011).

In the HRV-BF group, participants were asked to breathe in their own resonance frequency during the daily practice, guided by the StressEraser. Someone's resonance frequency is the breathing pace that yields the largest heart rate variability, which is usually close to six breaths per minute (Vaschillo et al. 2006). Participants estimated their personal resonance frequency during the orientation session, as a part of the StressEraser instructions. The StressEraser has an infrared sensor for the index finger which measures heart rate and uses this to guide participants in keeping the optimal breathing pace.

In the PE group, participants could choose their preferred intense exercises that increased heart rate, temperature, and breathing pace. Activities were allowed to vary between weeks and within individuals. For instance, one could run 10 min every day the first week and cycle fast to work 15 min a day the next week. This way, the intervention was tailored to the individual's abilities.

Measures

Attention Control Scale

The Attention Control Scale (ACS) consists of 20 items and assesses attention control (Derryberry and Reed 2001). Items

assess the ability to focus, shift, and divide attention (e.g., “It’s very hard for me to concentrate on a difficult task when there are noises around,” “It is easy for me to read or write while I’m also talking on the phone”). Internal consistency of the ACS at pre-test in the current sample was 0.88.

Behavior Rating Inventory of Executive Function

The Behavior Rating Inventory of Executive Function–Adult version (BRIEF-A) is a self-report questionnaire about executive functions (Roth et al. 2005) and consists of 75 items (e.g., “I can concentrate only briefly,” “I am not a good organizer”) divided over the following nine subscales: inhibition, shifting, emotional control, self-monitoring, initiate, working memory, plan/organize, task monitoring, and organization of materials. Internal consistency of the total BRIEF-A at pre-test was very high in this study ($\alpha = 0.95$).

Five Facet Mindfulness Questionnaire–Short Form

The short version of the Five Facet Mindfulness Questionnaire (FFMQ-SF) is a 24-item self-report questionnaire that consists of the following five domains of mindful awareness: observing, describing, acting with awareness, non-judging, and non-reactivity with items such as “When I’m walking, I deliberately notice the sensations of my body moving,” “I criticize myself for having irrational or inappropriate emotions,” or “I perceive my feelings and emotions without having to react to them” (Bohlmeijer et al. 2011). Internal consistency of the FFMQ ranges from 0.75 to 0.90 for the Dutch population (De Bruin et al. 2012). In the current sample, internal consistency of the FFMQ total score at pre-test was good, i.e., $\alpha = 0.86$.

Self-Compassion Scale

The short version of the Self-Compassion Scale (SCS-SF) is a 12-item self-report questionnaire which assesses self-compassion by using the following six subscales: self-kindness, self-judgment, common humanity, isolation, mindfulness, and overidentification (Neff 2003; Raes et al. 2011). Self-compassion is a way of being kind to oneself, to observe one’s experience as being part of humanity (a sense of togetherness, connectedness), and to be mindfully aware of difficult experiences instead of getting overly caught up in them (Neff 2003). Example items are “When I’m going through a very hard time, I give myself the caring and tenderness I need” or “I’m disapproving and judgmental about my own flaws and inadequacies.” Internal consistency of the Dutch version is high, $\alpha = 0.87$ (Raes et al. 2011). In the current sample, alpha for SCS total score at pre-test was high as well with $\alpha = 0.87$.

Penn State Worry Questionnaire

The Penn State Worry Questionnaire (PSWQ) measures the tendency, intensity, and uncontrollability of worry and consists of 16 items. Example items are “My worries overwhelm me” or “I am always worrying about something.” The PSWQ has shown high internal consistencies in clinical and non-clinical samples with α from 0.86 to 0.95 (Meyer et al. 1990; Van Rijsoort et al. 1999). In this sample, Cronbach’s alpha was 0.94 at pre-test.

Data Analyses

Generalized estimating equation (GEE) analyses (Zeger and Liang 1986) were carried out to examine whether the interventions differed in effects on the dependent variables. Based on three measurement occasions, a total sample size of 75, three groups, and a correlation among repeated measures ranging from 0.6 to 0.9 for the different measures, the minimal effect sizes that can be detected range from $f = 0.31$ to $f = 0.35$. This indicates that we should be able to detect at least a medium effect (medium effect is $f = 0.39$).

In addition, post hoc GEE analyses were carried out for “optimal dose” and for “equal dose” participants. “Optimal dose participants” were people that adhered to at least 70 % (>416.5 min) of the homework time and practices. For “equal dose participants,” a comparison was made between interventions for a selection of participants who were exposed to the same number of minutes of training. This comparison was included because even though the duration of the intervention was 34 days in all groups, participants in the PE group exercised significantly more minutes, $p < 0.01$, than those in the MM and the HRV-BF groups (mean training times 634.58, 364.04, and 370.27 min, respectively).

Within-group Cohen’s *d* was calculated as a measure of effect by dividing the mean difference (e.g., post-test minus pre-test) by the SD of these differences. An effect size of 0.3 was considered small, 0.5 medium, and 0.8 large (Cohen 1988).

Results

Eleven participants did not fill out the complete post-test and/or follow-up questionnaires. To assess whether these participants differed from those without missing values, a *t* test was carried out on the pre-test variables age and socioeconomic status (SES) and a chi-squared test to compare the groups on gender and allocated intervention. Furthermore, participants with missing values were compared to those without on the outcome variables at pre-test. Groups showed no differences on any of these variables (all *p* values >0.11), with the exception of self-compassion; participants with missing values scored lower on self-compassion ($p = 0.01$). All *Z* scores for

skewness and kurtosis fell within the normal range. Averages and standard deviations of the three groups on all outcome measures are displayed in Table 1.

GEE analyses showed that all interventions were effective over time with respect to attention control, executive functioning, mindful awareness, self-compassion, and worrying (all p values <0.03). MM, HRV-BF, and PE did not differ significantly from each other in effects on attention control, ACS, Wald χ^2 (2) = 7.25, $p = 0.33$; executive functioning, BRIEF-A, Wald χ^2 (2) = 4.12, $p = 0.13$; mindful awareness, FFMQ, Wald χ^2 (2) = 3.28, $p = 0.19$; self-compassion, SCS, Wald χ^2 (2) = 1.02, $p = 0.60$; and worrying, PSWQ, Wald χ^2 (2) = 0.45, $p = 0.80$. Since no main differences were found between groups, no further post hoc testing was carried out. Overall, effect sizes in the MM and PE group ranged from small to large (mean effect sizes are 0.38 and 0.52, respectively), whereas in the HRV-BF group, effect sizes were small to medium (mean effect size = 0.29; see Table 1).

Two additional analyses with respect to intervention effects were carried out. As we compared self-guided interventions, there was no guarantee that people practiced as much as in instructor-guided interventions. Therefore, first, optimal dose participants were selected as those who practiced at least 70 % of the homework time ($n = 12$ in the MM group, $n = 10$ in the HRV-BF group, and $n = 14$ in the PE group). Again, GEE analyses showed that all interventions were effective over time with respect to attention control, executive functioning, mindful awareness, self-compassion, and worrying (all p values <0.05). Participants in the "MM optimal dose," the "HRV-BF optimal dose," and the "PE optimal dose" groups did not differ significantly from each other with respect to attention control, ACS, Wald χ^2 (2) = 5.42, $p = 0.07$; executive functioning, BRIEF-A, Wald χ^2 (2) = 5.10, $p = 0.08$; mindful awareness, FFMQ, Wald χ^2 (2) = 0.30, $p = 0.86$; self-compassion, SCS, Wald χ^2 (2) = 3.29, $p = 0.19$; and worrying, PSWQ, Wald χ^2 (2) = 0.81, $p = 0.67$. Effect sizes in all optimal dose groups were somewhat higher than compared to those in the total sample, now ranging from small to large, with mean effect sizes of 0.59, 0.55, and 0.55 for MM, HRV-BF, and PE, respectively (see further Table 1).

Second, although number of days of practice did not differ between groups (23, 25, and 26 days of practice on average for the MM, HRV-BF, and PE groups, respectively), we found that participants in the PE group practiced significantly longer (more minutes) in total than participants in the MM group and the HRV-BF group, $F(2,47) = 8.89$, $p < 0.01$. More specifically, PE participants exercised for an average of 10.58 h (SD = 6.25), whereas MM and HRV-BF participants practiced meditations and HRV-biofeedback for an average of 6.07 h (SD = 2.31) and 6.17 h (SD = 2.62), respectively. Participants in the MM and the HRV-BF groups practiced one third less than prescribed in this study ([1 week × 10 min] + [1 week × 15 min] + [3 weeks × 20 min] = 9.92 h), whereas PE participants appeared to have no difficulty following the prescribed exercise time. Analyses were therefore repeated in a subgroup where practice dose did not differ significantly between the groups ($n = 18$ for MM with on average 7.23 h of practice, $n = 18$ for HRV-BF with on average 7.43 h of practice, and $n = 14$ for PE with on average 7.48 h of practice). To create these adjusted subgroups, the six participants who practiced most were removed from the PE group, and the six participants who practiced least were removed from both the MM and HRV-BF groups. GEE analyses showed that all interventions were effective over time with respect to attention control, executive functioning, mindful awareness, self-compassion, and worrying (all p values <0.01). Furthermore, GEE analyses showed that participants in the equal dose groups did not significantly differ in effects on attention control, ACS, Wald χ^2 (2) = 4.15, $p = 0.13$; executive functioning, BRIEF-A, Wald χ^2 (2) = 4.46, $p = 0.11$; mindful awareness, FFMQ, Wald χ^2 (2) = 1.01, $p = 0.60$; self-compassion, SCS, Wald χ^2 (2) = 1.05, $p = 0.59$; and worrying, PSWQ, Wald χ^2 (2) = 0.65, $p = 0.72$. Effect sizes were examined, and again effects in all groups varied from small to large, with mean effect sizes of 0.54, 0.38, and 0.61, for the MM group, the HRV-BF group, and the PE group, respectively. Note the large effect on mindful awareness in the PE group (1.24 pre-post and 0.79 pre-follow-up) as compared to the MM (0.48 pre-post and 0.52 pre-follow-up) and the HRV-BF groups (0.81 pre-post and 0.78 pre-follow-up). Furthermore, HRV-biofeedback showed very little effect at follow-up for attention control (effect size of 0.02 versus 0.88 in the MM group and 0.64 in the PE group) and executive functioning (effect size of 0.06 versus 0.88 in the MM group and 0.34 in the PE group; see further Table 1 and Fig. 2a–e).

Discussion

This study assessed the differential effects of three brief self-help interventions of daily meditations, daily HRV-biofeedback, and daily physical exercise on attention control, executive functioning, mindful awareness, self-compassion, and worrying. Core elements of mindfulness training (parallel with this study's MM group) are mindful awareness and (self-) compassion, as well as gaining control over one's attention, and distancing from thinking and thereby reducing worrying, which was not the case in PE group or the HRV-BF group. We therefore expected a larger improvement in these cognitive processes in the MM group compared to the PE or HRV-BF groups. Overall, the self-help interventions were found to be effective, but, unexpectedly, effects did not differ between the groups. As control over the amount of practice is limited in a self-guided intervention, and participants in the PE group exercised substantially more than in the MM and HRV-BF groups, post hoc group comparisons were

Table 1 Means (SDs) and within-group effect sizes of change in the mindfulness meditation, heart rate variability biofeedback, and physical exercise groups for attention control, executive functioning, mindful awareness, self-compassion, and worrying, at pre-test, post-test, and follow-up, in the total sample, the optimal dose sample, and the equal dose sample

		Total sample					Optimal dose sample		Equal dose sample	
		M (SD) pre-test	*M* (SD) post-test	*M* (SD) FU	*ES* pre-post	*ES* pre-FU	*ES* pre-post	*ES* pre-FU	*ES* pre-post	*ES* pre-FU
ACS	MM	50.11 (9.43)	51.76 (8.33)	53.75 (7.85)	0.13	0.47	0.34	0.95	0.34	0.88
	HRV-BF	52.72 (7.84)	53.67 (8.88)	52.48 (8.17)	0.16	0.01	0.28	0.33	0.24	0.02
	PE	52.22 (9.66)	55.09 (8.18)	54.90 (8.68)	0.39	0.52	0.25	0.38	0.45	0.64
BRIEF-A	MM	127.89 (25.30)	123.76 (22.42)	118.20 (24.47)	0.14	0.68	0.19	0.73	0.36	0.88
	HRV-BF	119.52 (15.88)	121.58 (17.15)	119.57 (17.15)	0.19	0.02	0.17	0.29	0.03	0.06
	PE	122.35 (19.49)	120.23 (25.10)	119.05 (28.46)	0.10	0.25	0.07	0.46	0.24	0.34
FFMQ	MM	77.85 (9.81)	80.80 (7.82)	81.17 (9.95)	0.27	0.35	0.61	0.89	0.48	0.52
	HRV-BF	77.28 (11.09)	83.67 (8.89)	83.30 (8.44)	0.68	0.58	0.88	1.22	0.81	0.78
	PE	74.57 (10.50)	82.82 (10.17)	80.35 (10.48)	0.99	0.61	1.02	0.62	1.24	0.79
SCS	MM	3.90 (1.13)	4.32 (0.98)	4.31 (1.10)	0.39	0.39	0.50	0.62	0.44	0.49
	HRV-BF	4.07 (1.08)	4.35 (1.12)	4.21 (1.10)	0.44	0.16	0.45	0.12	0.53	0.14
	PE	3.76 (0.76)	4.21 (0.87)	4.12 (0.99)	0.78	0.36	0.68	0.73	0.72	0.58
PSWQ	MM	56.78 (13.87)	53.12 (13.42)	53.08 (15.89)	0.46	0.51	0.46	0.62	0.46	0.59
	HRV-BF	51.40 (14.32)	48.42 (14.28)	48.70 (14.31)	0.36	0.32	0.77	0.99	0.58	0.56
	PE	53.91 (13.56)	49.73 (14.65)	50.35 (14.37)	0.69	0.47	0.84	0.47	0.56	0.51

ACS Attention Control Scale, *BRIEF-A* Behavior Rating Inventory of Executive Function–Adult version, *ES* effect size, *FFMQ* Five Facet Mindfulness Questionnaire, *FU* follow-up, *HRV-BF* heart rate variability biofeedback, *MM* mindfulness meditation, *PE* physical exercise, *PSWQ* Penn State Worry Questionnaire, *SCS* Self-Compassion Scale

Optimal dose: participants with >70% of the prescribed exercise time

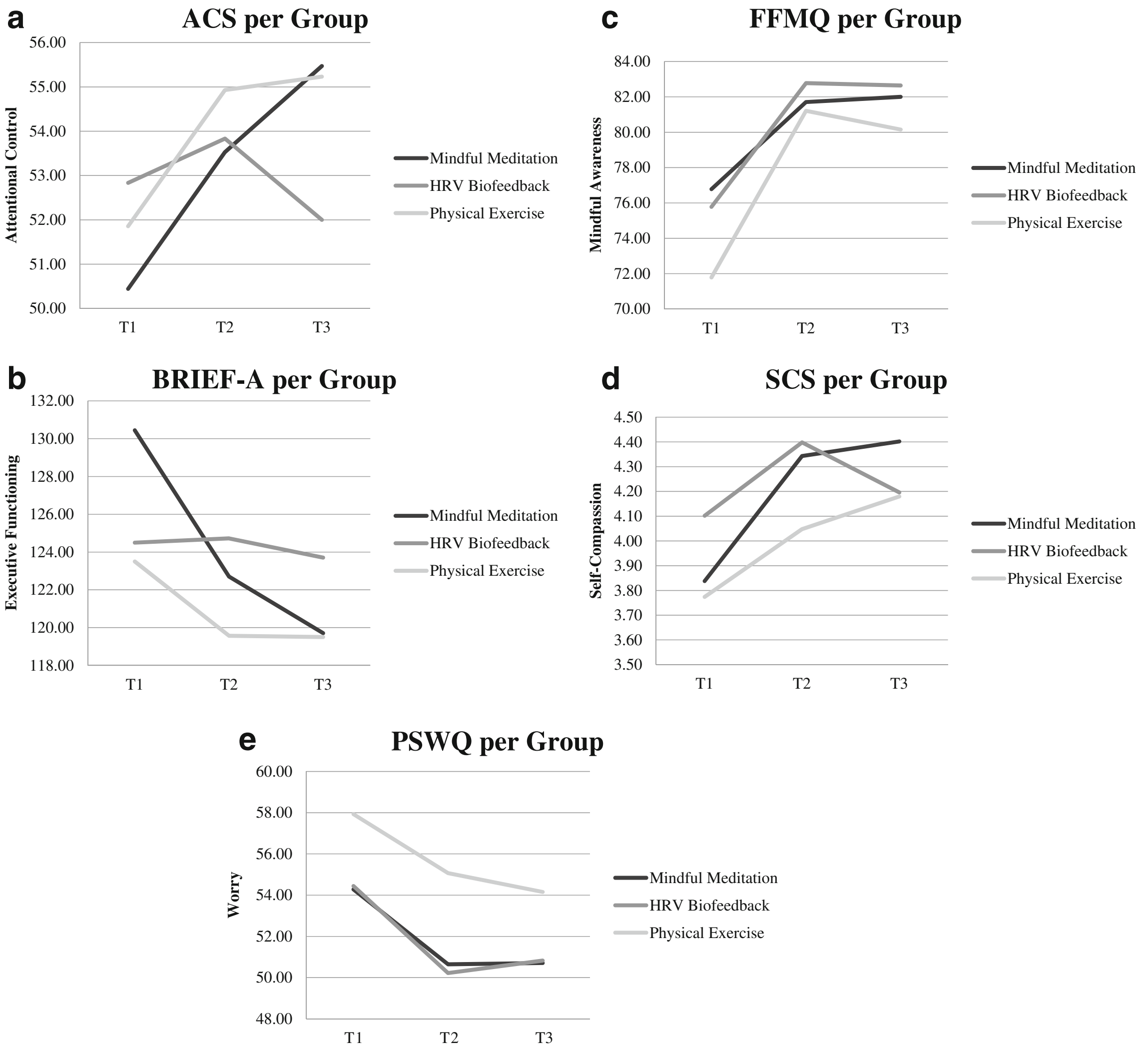

Fig. 2 **a** Attention Control Scale (ACS) **b** Behavior Rating Inventory of Executive Function–Adult version (BRIEF-A) **c** Five Facet Mindfulness Questionnaire (FFMQ) **d** Self-Compassion Scale (SCS) and **e** Penn State Worry Questionnaire (PSWQ) at pre-test (T1), post-test (T2), and follow-up (T3) for the equal dose group mindfulness meditation, HRV-biofeedback, and physical exercise

carried out correcting for optimal dose and equal dose. The lack of difference between the three groups however remained, and therefore, results appear robust.

Unexpected and most remarkable were the large benefits for mindful awareness in the PE and, although to a lesser extent, HRV-BF groups. This is interesting since mindful awareness is not explained or practiced during physical exercise or HRV-biofeedback exercises, whereas mindful awareness is the central target of a mindfulness training including the daily meditations. Mothes et al. (2014) hypothesize that as a result of physical exercise (and thus increased breathing, heart rate, etc.), body awareness may be enhanced, which in turn leads to increased mindful awareness. They further reason that physical exercise may lead to improved self-regulation of attention, which also leads to enhanced mindful awareness. We also expect that during physical activity, there is little attention space for thinking and rumination and thus greater here and now attention. Furthermore, physical exercise in this study sometimes took place outside, and contact with nature and simply the physical sensations of warmth and cold, humidity and dry, etc., may have enhanced present moment awareness. Positive effects of nature of on stress-related symptoms and well-being have been described in systematic reviews before (e.g., Annerstedt and Währborg 2011). As for HRV-biofeedback, one can imagine that some similarities may exist between meditation and HRV-biofeedback exercises (e.g., focus on the breathing), which could explain the improved mindful awareness. In addition, both HRV-biofeedback and physical exercise have a stress-reducing effect (e.g., Ratanasiripong et al. 2012; Salmon 2001), which may in turn be related to the increased mindful awareness.

Further, all three interventions showed a positive effect on self-compassion, indicating that they can be valuable tools for people with difficulties being gentle towards themselves and

understanding towards their own shortcomings. Self-compassion and depression, as well as self-compassion and anxiety, are inversely related (Raes 2010). This might hold some relation to why all three interventions have an effect on self-compassion, because meditation, HRV-biofeedback, and physical activity have all shown to reduce depression and anxiety symptoms (e.g., Conn 2010a, b; Henriques et al. 2011; Hoffman et al. 2010).

From neuro-scientific studies, it is further known that both physical exercise as well as meditation lead to improved functioning in brain regions involved in the regulation of attention and executive functioning (i.e., Hillman et al. 2008; Tang and Posner 2009), which might explain the lack of difference between the MM and PE groups in attention control and executive functioning in the current study. Although no significant differences between groups were found, effect sizes for attention control and executive functioning were very low in the HRV-biofeedback group. Considering the relatively low p value for attention control and executive functioning in the equal dose sample, it is possible that the effect of the HRV-BF intervention actually differs from the effect of the MM and PE interventions. With the current sample size, however, only medium effects could be detected; therefore, it is possible that a smaller effect did not show up.

Finally, with respect to worrying, findings of no larger effects in the mindfulness group as compared to the other groups were also surprising. Although it was previously shown that meditating, exercising, and applying HRV-biofeedback all reduce symptoms of anxiety, and worrying is considered to be part of anxiety, only in mindfulness meditation one specifically practices with decentering from one's (worrisome and ruminating) thoughts. Perhaps this is related to the very brief nature of the intervention in this study. Throughout a regular 8-week trainer-guided MBI, participants practice repeatedly with not getting too attached to thoughts, being able to see thoughts simply as thoughts and not as facts, practice with letting thoughts pass, and observing them from a distance which in turn lowers worrying and rumination. However, the theme of "thoughts are just thoughts" is not something that one starts with at the beginning of an MBI. This is usually covered in the more advanced stages. Since in this study only the more basic practices were covered (i.e., awareness of the breath and the body), it is possible that practicing with decentering from one's thoughts was hardly attended to, and therefore, effect on worrying was not larger than in the other two groups.

The fact that only medium effects could be detected in this study is a limitation. However, it can be argued that differences between interventions like the ones studied here are only meaningful if they are at least medium in effect, because it may only be worthwhile to participate in another intervention than the one that the participant prefers if the benefits of that intervention are detectably larger. In this study, the equal/optimal dose analyses were carried out in a smaller subsample and were therefore slightly underpowered. Other limitations of the current study were the reliance on self-reports only, and the participant's educational level being somewhat skewed towards the higher levels, limiting generalizability to a broader population. A further limitation was that the intensity of exercise ("treatment dose") was nearly double in the physical exercise group as compared to the other two groups, which made group comparisons more complex. In this study, multiple comparisons were made and results should therefore be interpreted with caution. Note however that there seemed to be a clear pattern of overall effectiveness of the three interventions on the cognitive processes and no evidence that mindfulness outperformed physical exercise or HRV-biofeedback.

For future studies, objective outcome measures that do not rely on self-report need to be included, as well as a larger sample. Further, the current sample consisted of nearly 50 % university students, and although it is known that students suffer from high rates of stress (e.g., Eisenberg et al. 2007), it would be interesting to apply these trainings to other groups as well. Finally, replication is needed using guided groups, since some of these interventions might be more effective in a guided format.

Conclusion

This study demonstrated that overall self-regulated daily meditations, daily HRV-biofeedback, and daily physical exercises lead to improved attention control, executive functioning, increased mindful awareness and self-compassion, and to less worrying. Mindful meditation did not outperform HRV-biofeedback or daily physical exercise with respect to these cognitive processes.

Acknowledgments We would like to thank Silke de Klerk, Kirsten Laheij, Kirsten Timmermans, Charlotte van Dijk, and particularly Michelle Azarhoosh for their valuable assistance in this project. This research is supported by Philips and Technology Foundation STW, Nationaal Initiatief Hersenen en Cognitie (NIHC), under the partnership program Healthy Lifestyle Solutions (project number 12001).

Open Access This article is distributed under the terms of the Creative Commons Attribution 4.0 International License (http://creativecommons.org/licenses/by/4.0/), which permits unrestricted use, distribution, and reproduction in any medium, provided you give appropriate credit to the original author(s) and the source, provide a link to the Creative Commons license, and indicate if changes were made.

References

Annerstedt, M., & Währborg, P. (2011). Nature-assisted therapy: systematic review of controlled and observational studies. *Scandinavian Journal of Public Health, 39*, 371–388.

Best, J. R. (2010). Effects of physical activity on children's executive function: contributions of experimental research on aerobic exercise. *Developmental Review, 30*, 331–351.

Bohlmeijer, E., ten Klooster, P. M., Fledderus, M., Veehof, M., & Baer, R. (2011). Psychometric properties of the Five Facet Mindfulness Questionnaire in depressed adults and development of a short form. *Assessment, 18*, 308–320.

Brown, K. W., & Ryan, R. M. (2003). The benefits of being present: mindfulness and its role in psychological well-being. *Journal of Personality and Social Psychology, 84*, 822–848.

Carmody, J., & Baer, R. A. (2008). Relationships between mindfulness practice and levels of mindfulness, medical and psychological symptoms and well-being in a mindfulness-based stress reduction program. *Journal of Behavioral Medicine, 31*, 23–33.

Caspi, A., Houts, R. M., Belsky, D. W., Goldman-Mellor, S. J., Harrington, H., Israel, S., et al. (2014). The p factor: one general psychopathology factor in the structure of psychiatric disorders? *Clinical Psychological Science, 2*, 119–137.

Chiesa, A., & Serretti, A. (2009). Mindfulness-based stress reduction for stress management in healthy people: a review and meta-analysis. *Journal of Alternative and Complementary Medicine, 15*, 593–600.

Chiesa, A., Calati, R., & Serretti, A. (2011). Does mindfulness training improve cognitive abilities? A systematic review of neuropsychological findings. *Clinical Psychology Review, 31*, 449–464.

Cohen, J. (Ed.) (1988). *Statistical power analysis for the behavioural sciences (2nd ed.)*. New York: Academic Press.

Cohen, S., & Janicki-Deverts, D. (2012). Who's stressed? Distributions of psychological stress in the United States in probability samples from 1983, 2006, and 2009. *Journal of Applied Social Psychology, 42*, 1320–1334.

Cohen, S., Kamarck, T., & Mermelstein, R. (1983). A global measure of perceived stress. *Journal of Health and Social Behavior, 24*(4), 385–396.

Cohen, S., Janicki-Deverts, D., Doyle, W. J., Miller, G. E., Frank, E., Rabin, B. S., et al. (2012). Chronic stress, glucocorticoid receptor resistance, inflammation, and disease risk. *Proceedings of the National Academy of Sciences of the USA, 109*, 5995–5999.

Conn, V. S. (2010a). Anxiety outcomes after physical activity interventions: meta-analysis findings. *Nursing Research, 59*, 224–231.

Conn, V. S. (2010b). Depressive symptom outcomes of physical activity interventions: meta-analysis findings. *Annals of Behavioral Medicine, 39*, 128–138.

Davidson, R. J., Kabat-Zinn, J., Schumacher, J., Rosenkranz, M., Muller, D., Santorelli, S. F., et al. (2003). Alterations in brain and immune function produced by mindfulness meditation. *Psychosomatic Medicine, 65*, 564–570.

De Bruin, E. I., Topper, M., Muskens, J. G. A. M., Bögels, S. M., & Kamphuis, J. H. (2012). Psychometric properties of the Dutch Five Facets Mindfulness Questionnaire (FFMQ-NL) in a meditating and a non-meditating sample. *Assessment, 19*, 187–197.

Derryberry, D., & Reed, M. A. (2001). Attention control, trait anxiety, and the regulation of irrelevant response information. *Journal of Abnormal Psychology, 111*, 225–236.

Eisenberg, D., Gollust, S. E., Golberstein, E., & Hefner, J. L. (2007). Prevalence and correlated of depression, anxiety, and suicidality among university students. *American Journal of Orthopsychiatry, 77*, 534–542.

European Agency for Safety and health at Work (2009). *OSH in figures: stress at work—facts and figures*.

Flook, L., Smalley, S. L., Kitil, M. J., Galla, B. M., Kaiser-Greenland, S., Locke, J., et al. (2010). Effects of mindful awareness practices on executive functions in elementary school children. *Journal of Applied School Psychology, 26*, 70–95.

Harnett, P. H., & Dawe, S. (2012). The contribution of mindfulness-based therapies for children and families and proposed conceptual integration. *Child and Adolescent Mental Health, 17*, 195–208.

Henriques, G., Keffer, S., Abrahamson, C., & Horst, S. J. (2011). Exploring the effectiveness of a computer-based heart rate variability biofeedback program in reducing anxiety in college students. *Applied Psychophysiology and Biofeedback, 36*, 101–112.

Hillman, C. H., Erickson, K. I., & Kramer, A. F. (2008). Be smart, exercise your heart: exercise effects on brain and cognition. *Nature Reviews Neuroscience, 9*, 58–65.

Hoffman, S. G., Sawyer, A. T., Witt, A. A., & Oh, D. (2010). The effect of mindfulness-based therapy on anxiety and depression: a meta-analytic review. *Journal of Consulting Clinical Psychology, 78*, 169–183.

Jazaieri, H., Goldin, P. R., Werner, K., Ziv, M., & Gross, J. J. (2012). A randomized trial of MBSR versus aerobic exercise for social anxiety disorder. *Journal of Clinical Psychology, 68*, 715–731.

Kabat-Zinn, J. (2003). Mindfulness-based interventions in context: past, present, and future. *Clinical Psychology: Science and Practice, 10*, 144–156.

Kabat-Zinn, J., Lipworth, L., & Burney, R. (1985). The clinical use of mindfulness meditation for the self-regulation of chronic pain. *Journal of Behavioral Medicine, 8*, 163–190.

Kuyken, W., Watkins, E., Holden, E., White, K., Taylor, R. S., Byford, S., et al. (2010). How does mindfulness-based cognitive therapy work? *Behavior Research and Therapy, 48*, 1105–1112.

Lawlor, D., & Hopker, S. W. (2001). The effectiveness of exercise as an intervention in the management of depression: systematic review and meta-regression analysis of randomized controlled trials. *British Medical Journal, 322*, 1–8.

Lehrer, P. M., Vaschillo, E., Vaschillo, B., Lu, S. E., Scardella, A., Siddique, M., et al. (2004). Biofeedback treatment for asthma. *Chest, 126*, 352–361.

Lehrer, F., Vaschillo, E., Lu, S. E., Eckberg, D., Vaschillo, B., Scardella, A., et al. (2006). Heart rate variability biofeedback—effects of age on heart rate variability, baroreflex gain, and asthma. *Chest, 129*, 278–284.

Lin, G., Xiang, Q., Fu, X., Wang, S., Wang, S., Chen, S., et al. (2012). Heart rate variability biofeedback decreases blood pressure in prehypertensive subjects by improving autonomic function and baroreflex. *Journal of Alternative and Complementary Medicine, 18*, 143–152.

Meyer, T. J., Miller, M. L., Metzger, R. L., & Borkovec, T. D. (1990). Development and validation of the Penn State Worry Questionnaire. *Behavior Research and Therapy, 28*, 487–495.

Mothes, H., Klaperski, S., Seelig, H., Schmidt, S., & Fuchs, R. (2014). Regular aerobic exercise increases dispositional mindfulness in men: a randomized controlled trial. *Mental Health and Physical Activity, 7*, 111–119.

Neff, K. D. (2003). Development and validation of a scale to measure self-compassion. *Self and Identity, 2*, 223–250.

Netterstrøm, B., Conrad, N., Bech, P., Fink, P., Olsen, O., Rugulies, R., et al. (2008). The relation between work-related psychosocial factors and the development of depression. *Epidemiologic Reviews, 30*, 118–132.

Powell, K. E., Paluch, A. E., & Blair, S. N. (2011). Physical activity for health: what kind? How much? How intense? On top of what? *Public Health, 32*, 349–365.

Prinsloo, G. E., Rauch, H. G. L., Lambert, M. I., Muench, F., Noakes, T. D., & Derman, W. E. (2011). The effect of short duration heart rate variability (HRV) biofeedback on cognitive performance during laboratory induced cognitive stress. *Applied Cognitive Psychology, 25*, 792–801.

Raes, F. (2010). Rumination and worrying as mediators of the relationship between self-compassion and depression and anxiety. *Personality and Individual Differences, 48*, 757–761.

Raes, F., Pommier, E., Neff, K. D., & Van Gucht, D. (2011). Construction and factorial validation of a short form of the Self-Compassion Scale. *Clinical Psychology & Psychotherapy, 18*, 250–255.

Ratanasiripong, P., Ratanasiripong, N., & Kathalae, D. (2012). Biofeedback intervention for stress and anxiety among nursing

students: a randomized controlled trial. *ISRN Nursing*. doi:10.5402/2012/827972. 2090-5491 (Electronic).

Reiner, R. (2008). Integrating a portable biofeedback device into clinical practice for patients with anxiety disorders: results of a pilot study. *Applied Psychophysiology and Biofeedback, 33*, 55–61.

Robins, C., Keng, S. L., Ekblad, A. G., & Brantley, J. G. (2012). Effects of mindfulness-based stress reduction on emotional experience and expression: a randomized controlled trial. *Journal of Clinical Psychology, 68*, 117–131.

Rosch, P. J. (2001). The quandary of job stress compensation. *Health and Stress, 3*, 1–4.

Roth, R. M., Isquith, P. K., & Gioia, G. A. (2005). *BRIEF-A: Behavior Rating Inventory of Executive Function–Adult version: professional manual*. Odessa, FL: Psychological Assessment Resources.

Salmon, P. (2001). Effects of physical exercise on anxiety, depression, and sensitivity to stress: a unifying theory. *Clinical Psychology Review, 21*, 33–61.

Sedlmeier, P., Eberth, J., Schwarz, M., Zimmerman, D., Haarig, F., Jaeger, S., et al. (2012). The psychological effects of meditation: a meta-analysis. *Psychological Bulletin, 138*, 1139–1171.

Siepmann, M., Aykac, V., Unterdoerfer, J., Petrowski, K., & Mueck-Weymann, M. (2008). A pilot study on the effects of heart rate variability biofeedback in patients with depression and in healthy subjects. *Applied Psychophysiology and Biofeedback, 33*, 195–201.

Slagter, H. A., Lutz, A., Greischar, L. L., Francis, A. D., Nieuwenhuis, S., Davis, J. M., et al. (2007). Mental training affects distribution of limited brain resources. *PLoS Biology, 5*, e138.

Stansfeld, S., & Candy, B. (2006). Psychosocial work environment and mental health—a meta analytic review. *Scandinavian Journal of Work, Environment & Health, 32*, 443–462.

Stathopoulous, G., Powers, M. B., Berry, A. C., Smits, J. A. J., & Otto, M. W. (2006). Exercise interventions for mental health: a quantitative and qualitative review. *Clinical Psychology: Science and Practice, 13*, 179–193.

Tang, Y. Y., & Posner, M. I. (2009). Attention training and attention state training. *Trends in Cognitive Sciences, 13*, 222–227.

Van der Zwan, J. E., de Vente, W., Huizink, A. C., Bögels, S. M., & de Bruin, E. I. (2015). Physical activity, mindfulness meditation, or heart rate variability biofeedback for stress reduction: a randomized controlled trial. *Applied Psychophysiology and Biofeedback, 40*, 257–68.

Van Rijsoort, S., Emmelkamp, P., & Vervaeke, G. (1999). The Penn State Worry Questionnaire and the Worry Domains Questionnaire. *Clinical Psychology and Psychotherapy, 6*, 297–307.

Vaschillo, E. G. V., Vaschillo, B., & Lehrer, P. M. (2006). Characteristics of resonance in heart rate variability stimulated by biofeedback. *Applied Psychophysiology and Biofeedback, 31*, 129–142.

Williams, M., & Penman, D. (2011). *Mindfulness: finding peace in a frantic world*. London: Piatkus.

Zeger, S. L., & Liang, K. Y. (1986). Longitudinal data analysis for discrete and continuous outcomes. *Biometrics, 42*, 121–130.

Zucker, T. L., Samuelson, K. W., Muench, F., Greenberg, M. A., & Gevirtz, R. N. (2009). The effects of respiratory sinus arrhythmia biofeedback on heart rate variability and posttraumatic stress disorder symptoms: a pilot study. *Applied Psychophysiology and Biofeedback, 34*, 135–143.
