# Supplementary material for: Does physical activity-based intervention decrease repetitive negative thinking? A systematic review
Source: PLoS One. 2025 Apr 1;20(4):e0319806. doi: 10.1371/journal.pone.0319806 (PMC11960971; doi:10.1371/journal.pone.0319806)
Supplement: S1 File — https://doi.org/10.6084/m9.figshare.25711734. (ZIP) [file pone.0319806.s001.zip › supporting information/paper file/Herring 2011.pdf]

Regular Article

Psychotherapy and Psychosomatics

Psychother Psychosom 2012;81:21–28
DOI: 10.1159/000327898

Received: October 20, 2010
Accepted after revision: March 25, 2011
Published online: November 22, 2011

Feasibility of Exercise Training for the Short-Term Treatment of Generalized Anxiety Disorder: A Randomized Controlled Trial

Matthew P. Herring[a, c] Marni L. Jacob[b] Cynthia Suveg[b] Rodney K. Dishman[a] Patrick J. O'Connor[a]

Departments of [a]Kinesiology and [b]Psychology, The University of Georgia, Athens, Ga., and [c]Department of Exercise Science, University of South Carolina, Columbia, S.C., USA

Key Words
Aerobic Exercise Training · Anxiety · Resistance Exercise Training · Worry

Abstract
Background: Exercise training may be especially helpful for patients with generalized anxiety disorder (GAD). We conducted a randomized controlled trial to quantify the effects of 6 weeks of resistance (RET) or aerobic exercise training (AET) on remission and worry symptoms among sedentary patients with GAD. ***Methods:*** Thirty sedentary women aged 18–37 years, diagnosed by clinicians blinded to treatment allocation with a primary DSM-IV diagnosis of GAD and not engaged in any treatment other than pharmacotherapy, were randomly allocated to RET, AET, or a wait list (WL). RET involved 2 weekly sessions of lower-body weightlifting. AET involved 2 weekly sessions of leg cycling matched with RET for body region, positive work, time actively engaged in exercise, and load progression. Remission was measured by the number needed to treat (NNT). Worry symptoms were measured by the Penn State Worry Questionnaire. ***Results:*** There were no adverse events. Remission rates were 60%, 40%, and 30% for RET, AET, and WL, respectively. The NNT was 3 (95% CI 2 to 56) for RET and 10 (95% CI –7 to 3) for AET. A significant condition-by-time interaction was found for worry symptoms. A follow-up contrast showed significant reductions in worry symptoms for combined exercise conditions versus the WL. ***Conclusions:*** Exercise training, including RET, is a feasible, low-risk treatment that can potentially reduce worry symptoms among GAD patients and may be an effective adjuvant, short-term treatment or augmentation for GAD. Preliminary findings warrant further investigation.

Copyright © 2011 S. Karger AG, Basel

Introduction

At least 25 randomized controlled trials have documented positive effects of exercise training on patients with depressive disorders, but only 2 have focused on anxiety disorder patients [1, 2]. In those trials, the influence of exercise training per se on anxiety symptoms was uncertain because research design weaknesses allowed nuisance factors to potentially confound the anxiety symptom reductions.

Several types of indirect evidence suggest that exercise training may be especially helpful for generalized anxiety disorder (GAD) patients. The evidence includes: (1) GAD patients tend to be physically inactive [3]; (2) exercise

KARGER
Fax +41 61 306 12 34
E-Mail karger@karger.ch
www.karger.com

© 2011 S. Karger AG, Basel
0033–3190/12/0811–0021$38.00/0

Accessible online at:
www.karger.com/pps

Matthew P. Herring
Department of Exercise Science, University of South Carolina
1300 Wheat Street
Columbia, SC 29208 (USA)
Tel. +1 803 777 7296, E-Mail mattpherring@gmail.com

Downloaded by:
Claude Moore Health Sciences Lib - Univ of Virginia
128.143.23.241 - 9/23/2013 3:57:31 AM

training reduces anxiety symptoms among healthy adults and patients with a chronic illness [4]; (3) exercise training benefits patients with major depressive disorder, which is highly comorbid with GAD and may be influenced by similar genetic factors [5]; and, (4) exercise has salutary effects on other signs and symptoms that characterize GAD patients, including fatigue [6] and poor concentration [7].

There is also a need to better understand the psychological consequences of resistance exercise training (RET). RET, though infrequently investigated compared to aerobic exercise training (AET) [4], had favorable effects in an early trial of anxiety patients [8]. Prior investigations comparing the psychological consequences of RET versus AET have not matched the 2 modes on more than 1 feature of the exercise stimulus.

The purpose of the randomized controlled trial reported here was to quantify the effects of 6 weeks of RET and AET, matched for the body area exercised, positive work, total time actively engaged in exercise, and weekly progression, on remission and worry symptoms among sedentary GAD patients. We hypothesized that, compared to a wait list (WL) control, both RET and AET would result in higher remission rates and larger improvements in worry symptoms.

Materials and Methods

Design and Patients

The study protocol for the trial was approved by an institutional review board. All volunteers provided written informed consent. Inclusion criteria were: (1) age of 18–39 years, (2) no concurrent psychiatric or psychological therapy other than medication, and (3) a primary DSM-IV diagnosis of GAD. Potential participants assigned an Anxiety Disorders Interview Schedule (ADIS-IV) [9] clinician severity rating ≥4 were diagnosed with GAD. Eligible patients were then enrolled into the intervention 1–15 days following ADIS-IV administration. Exclusion criteria included: (1) too few worry symptoms, defined by both a Psychiatric Diagnostic Screening Questionnaire (PDSQ) [10] GAD subscale score <6 and a Penn State Worry Questionnaire (PSWQ) [11] score <45; (2) too high a level of physical activity, defined by energy expenditure estimates using a 7-day physical activity recall (7PAR) [12] value >260 kcal/kg of body weight/week; (3) pregnancy; and, (4) contraindications to moderate-intensity exercise.

Random Allocation to Conditions

After screening and baseline assessment, one investigator (M.P.H.) allocated 30 patients in equal numbers to 3 conditions using blocked randomization (http://www.randomizer.org). Patients were blocked in blocks of 3 on the intervention condition (RET, AET, and WL) and stratified on psychoactive medication use (no medication or medication use) to ensure 3 similar groups of equal size. No patient refused randomization.

Baseline Strength Assessments

Following baseline outcome assessments, 4-repetition maximum (4-RM) was obtained on leg press, leg curl, and leg extension exercises using Cybex Eagle equipment. Estimated 1-repetition maximums (1-RM) were calculated as follows: 1-RM = 4-RM weight × 1.13.

Intervention Conditions

Both exercise training protocols involved 2 weekly sessions for 6 weeks. Exercise sessions were conducted with at least a 48-hour interval between each weekly session. Because mood improvements can result from social interaction, each session was supervised by 1 of 6 exercise specialists who purposefully avoided unnecessary conversation.

Resistance Exercise Training

RET sessions lasted approximately 46 min and 40 s and required 16 min of resistance exercise. Seven sets of 10 repetitions each were performed of leg press, leg curl and leg extension exercises beginning at 50% of the predicted 1-RM during week 1 and progressing by 5% of the predicted 1-RM weekly. Each exercise was preceded by a warm-up set of 10 repetitions beginning at 35% of the predicted 1-RM during week 1 and progressing by 5% of the predicted 1-RM weekly. Each eccentric and concentric action was performed for 2 s so that each set required 40 s. A rest interval of 80 s separated each set and each exercise. Heart rate, which was assessed using a Polar Vantage XL heart rate monitor, and ratings of perceived exertion (RPE) [13] and leg muscle pain intensity [14] were obtained within the first 15 s following the completion of the final set of each exercise. The session RPE was obtained following the workout.

Aerobic Exercise Training

The AET protocol was matched to the RET protocol for: (1) time actively engaged in exercise, (2) positive work, (3) a weekly 5% progression in load, and (4) body region. Two weekly sessions of 16 min of continuous leg cycling were performed. Heart rate, RPE, and leg muscle pain intensity were obtained during the last 10 s of the 2nd, 7th, and 15th min of each session. The session RPE was obtained following the workout.

Wait List Control

Patients assigned to the WL delayed entry into an exercise program for 6 weeks but completed weekly outcome assessments.

Outcomes

Clinicians blinded to allocation determined GAD diagnoses 1–16 days post-intervention using the ADIS-IV [9]. The ADIS-IV assesses for the presence of anxiety and related disorders using a semi-structured interview according to DSM-IV diagnostic criteria. Based on a 0–8 Likert severity scale, clinicians assign severity ratings to each disorder, thus allowing the delineation of principal and comorbid diagnoses. The psychometric properties of the ADIS-IV are well-established [9, 15]; its use as the diagnostic interview permitted a formal, careful, and thorough assessment of psychopathology.

Worry symptoms were assessed at baseline and at the beginning of the second weekly session during weeks 2, 4, and 6 with the PSWQ [11]. The PSWQ is a 16-item self-report questionnaire of the tendency to worry excessively. Patients responded on a

Downloaded by:
Claude Moore Health Sciences Lib - Univ of Virginia
128.143.23.241 - 9/23/2013 3:57:31 AM

5-point Likert scale for which higher scores indicated greater levels of worry. Completion of the scale required approximately 5 min. Favorable psychometric data support the PSWQ [16]. In the present investigation PSWQ scores (using all trials) demonstrated appropriate internal consistency (Chronbach's α = 0.92) and stability [ICC (2, 4) = 0.73; 95% CI 0.59 to 0.85].

Using the 9th item of the Beck Depression Inventory-II (Suicidal Thoughts or Wishes) [17], suicidal ideation was examined for safety monitoring, as a potential adverse event, and for intervention tolerability. Each patient completed a 24-hour prescription and nonprescription medication and supplement recall. Nonintervention physical activity also was estimated [12].

Preliminary Analyses

Descriptive statistics are presented in the text and tables as means [standard deviation (SD)] and in the figures as means (SE). χ^2 tests, Bonferroni-corrected for multiple comparisons, were used to evaluate baseline differences in the number of comorbid psychiatric diagnoses and psychoactive medication use. Baseline comparisons of other patient characteristics were performed using univariate ANOVA. Intervention intensity variables were averaged across 12 sessions and compared using independent samples t-tests.

Outcome Analyses

Clinician diagnoses of GAD were analyzed using the number needed to treat (NNT) [18]. The NNT and associated 95% CI were calculated as the inverse of the absolute risk reduction for each exercise condition compared with the WL condition.

Worry symptom scores were analyzed using a mixed-model 3 (condition: RET, AET, and WL) $\times$ 3 (time: weeks 2, 4, and 6) ANCOVA adjusted for baseline scores. Bonferroni-corrected pairwise comparisons were conducted to assess group differences. Because only 30% (n = 9) of patients were tested during the first 4 months (August to December), a 1-way intraclass correlation coefficient [ICC(1)] was calculated to examine the percentage of variance accounted for by the testing period (August to December and January to April). The testing period accounted for 5% of the variance, so a follow-up mixed-model 3 $\times$ 3 ANCOVA adjusted for baseline and the testing period was conducted. Because no differences between exercise conditions were hypothesized, a follow-up contrast (adjusted for the testing period) comparing WL with the combined exercise conditions on week 6 scores was computed.

At each time point, Hedges' d effect sizes and associated 95% CIs were calculated for each exercise condition [19]. Effect sizes were adjusted for small sample bias and calculated so that symptom improvement resulted in a positive effect size [19].

Results

Patient Flow

Patients were recruited from August 2009 through March 2010. Figure 1 illustrates the flow of patients through the trial. There were no musculoskeletal injuries or adverse events reported by the patients. The mean baseline BDI-II suicidal ideation item scores were 0.10, 0, and 0.50 for RET, AET, and WL, respectively, and did not increase for any condition across the investigation. No patient discontinued the intervention, and all patient data were used in analyses.

Baseline Patient Characteristics

Table 1 presents baseline demographic, medical, physical activity, and psychiatric characteristics of the sample. Corrected χ^2 tests showed a significantly larger number of comorbid psychiatric diagnoses for WL compared to RET.

Intervention Fidelity

WL patients completed 100% of the outcome assessments. Patients in the RET condition attended 100% (120/120) of sessions and complied with 99.1% of the RET protocol, completing 28,550 of 28,800 repetitions at the prescribed intensity. One RET session was not completed due to illness. Patients in the AET condition attended 100% (120/120) of sessions and complied with 100% of the AET protocol, each completing 12 bouts of 16 min of cycling exercise at the required power output. Thus, the average total minutes of exercise for the AET and RET patients was 192 and 190, respectively, out of a total of 192 possible minutes. Five total exercise bouts, i.e. 4 RET bouts and 1 AET bout, were completed away from the testing facility but were documented via phone calls in which exercise session duration and RPE were provided.

During the exercise sessions, RET was characterized by an overall mean (SD) RPE and heart rate (beats per minute) of 14 (1) and 125 (12). The overall means for AET were 8 (1) and 122 (8). There was not a significant difference in heart rate between exercise conditions [$t_{(18)} = 0.81$, p = 0.429]. The mean RPE during exercise was significantly higher for RET compared with AET [$t_{(18)} = 9.52$, $p < 0.0001$]. The session RPE was significantly higher for RET compared to AET [$t_{(18)} = 8.74$, $p < 0.001$]. As planned, RET resulted in larger strength increases across 6 weeks than did AET and WL (all Hedges' d $\geq$ 0.64).

Patients were asked to refrain from participating in other therapy programs during the intervention. However, after the trial 4 patients (1 RET, 2 AET, and 1 WL) reported that during the intervention they had minimal engagement (2 sessions) in an additional form of psychotherapy. A sensitivity analysis with these 4 patients removed did not change the statistical significance of the outcomes.

Outcomes

Remission rates were 60%, 40%, and 30% for RET, AET, and WL, respectively. The absolute risk reduction

Downloaded by:
Claude Moore Health Sciences Lib - Univ of Virginia
128.143.23.241 - 9/23/2013 3:57:31 AM

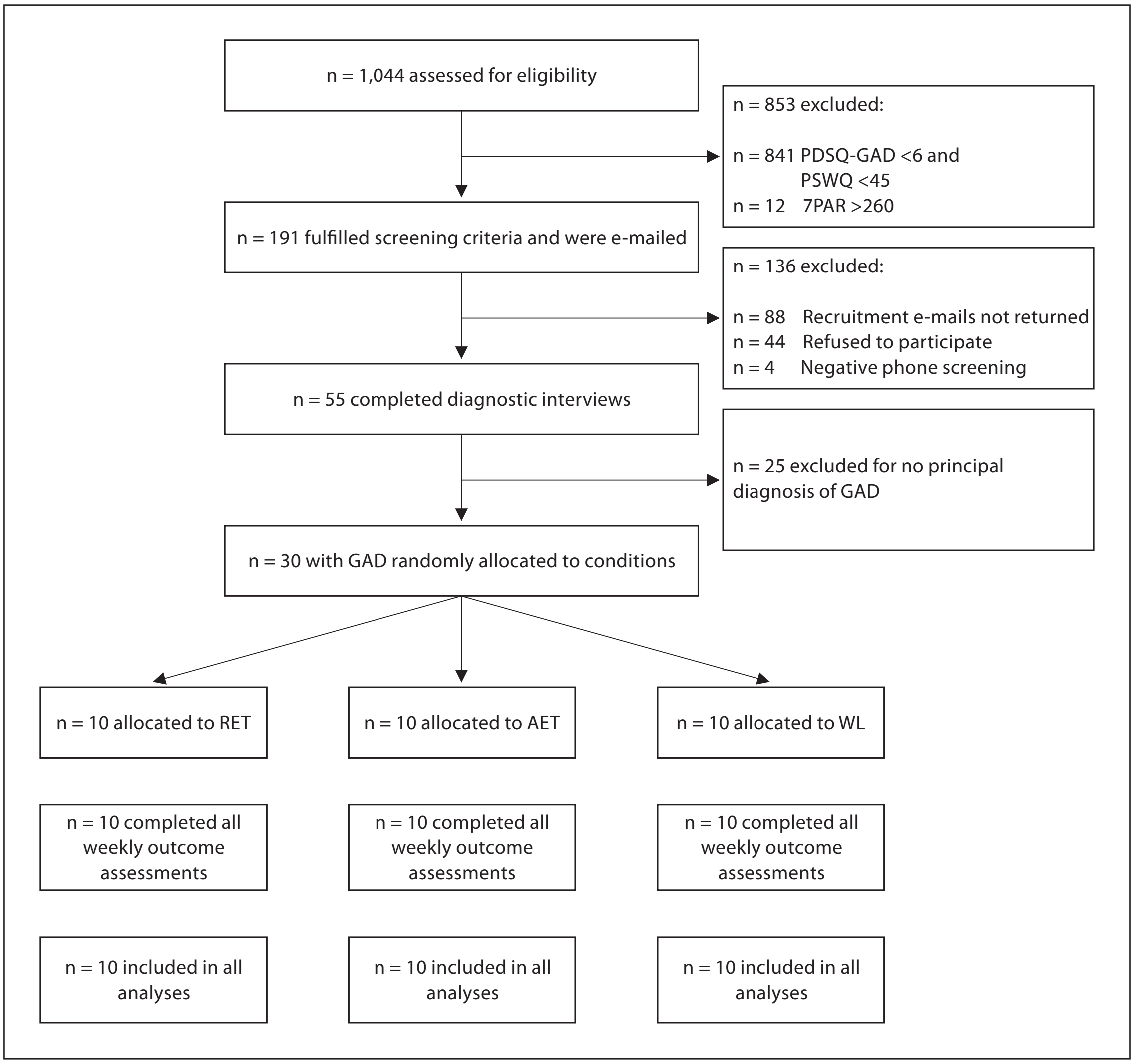

Fig. 1. Patient flow through the 6-week randomized, controlled trial.

for RET was 0.30 (95% CI 0.02 to 0.58) and the NNT was 3 (95% CI 2 to 56). The absolute risk reduction for AET was 0.10 (95% CI –0.15 to 0.35) and the NNT was 10 (95% CI –7 to 3). Psychoactive medication use did not moderate remission.

Table 2 presents the descriptive data for PSWQ scores. There was a significant condition-by-time interaction [$F_{(3.72, 48.4)} = 2.74$, $p = 0.042$, $\varepsilon = 0.931$]. Bonferroni-corrected pairwise comparisons of week 6 scores for RET [$t_{(18)} = 1.106$, $p = 0.28$] and AET [$t_{(18)} = 1.845$, $p = 0.081$] compared with WL were not significant; however, moderately large reductions in worry symptoms were found for both exercise conditions (Hedges' d = 0.45). A follow-up model adjusted for baseline scores and the testing period showed a significant condition-by-time interaction [$F_{(3.962, 49.529)} = 2.815$, $p = 0.035$, $\varepsilon = 0.991$]. A follow-up contrast of week 6 scores (fig. 2) showed larger reductions for the combined exercise conditions compared to WL [$t_{(25.943)} = 2.168$, $p = 0.039$]. Worry symptoms were not moderated by psychoactive medication use.

Weekly extra-intervention energy expenditure at weeks 2, 4, and 6 did not change significantly from baseline for RET, AET, or WL groups [$F_{(2, 52)} = 0.056$, $p = 0.946$]. These data suggest that symptom improvements were not confounded by nonintervention physical activity.

Downloaded by:
Claude Moore Health Sciences Lib - Univ of Virginia
128.143.23.241 - 9/23/2013 3:57:31 AM

Table 1. Baseline demographic, physical activity, medical, and psychiatric characteristics

Variable	Overall (n = 30)		RET (n = 10)		AET (n = 10)		WL (n = 10)	
	patients	%	patients	%	patients	%	patients	%
Age, years								
Mean ± SD	23.5 ± 5.9		25.6 ± 7.1		20.7 ± 3.0		24.2 ± 6.3	
Range	18 – 37		19 – 37		18 – 26		18 – 36	
College graduate	8	26.7	3	30.0	1	10.0	4	40.0
Married	3	10.0	1	10.0	0	0	2	20.0
Race/ethnicity								
Caucasian	19	63.3	5	50.0	8	80.0	6	60.0
African-American	3	10.0	0	0	1	10.0	2	20.0
Hispanic	3	10.0	2	20.0	1	10.0	0	0
Middle Eastern	2	6.7	2	20.0	0	0	0	0
Asian	2	6.7	1	10.0	0	0	1	10.0
Indian	1	3.3	0	0	0	0	1	10.0
Weight, kg								
Mean ± SD	65.7 ± 12.2		60.6 ± 9.1		70.0 ± 15.5		66.4 ± 8.1	
Height, cm								
Mean ± SD	164.7 ± 6.6		162.6 ± 6.2		165.0 ± 7.6		166.5 ± 4.6	
BMI, kg/m^2								
Mean ± SD	24.2 ± 5.8		22.8 ± 2.8		25.7 ± 5.2		24.0 ± 3.0	
7PAR, kcal/kg/week								
Mean ± SD	253.5 ± 27.7		263.3 ± 36.6		249.2 ± 21.5		248.0 ± 22.6	
Medication								
Contraceptive	15	50.0	5	50.0	5	50.0	5	50.0
Psychoactive								
SSRI	7	23.3	2	20.0	2	20.0	3	30.0
SNRI	2	6.7	1	10.0	1	10.0	0	0
NDRI	2	6.7	0	0	1	10.0	1	10.0
Muscle relaxant	2	6.7	1	10.0	1	10.0	0	0
Psychostimulant	1	3.3	0	0	0	0	1	10.0
Psychiatric comorbidity (cases[b])	21 (50)	70.0	5 (9)	50.0	6 (14)	60.0	10 (27)[a]	100.0
Social phobia	12	24.0	2	22.2	5	35.7	5	18.5
Specific phobia	19	38.0	2	22.2	5	35.7	12	44.4
OCD	5	10.0	1	11.1	1	7.1	3	11.1
PTSD	2	4.0	0	0	0	0	2	7.4
MDD	7	14.0	1	11.1	3	21.4	3	11.1
Dysthymia	4	8.0	2	22.2	0	0	2	7.4
Substance abuse	1	2.0	1	11.1	0	0	0	0

BMI = Body mass index; SSRI = selective serotonin reuptake inhibitor; SNRI = serotonin-norepinephrine reuptake inhibitor; NDRI = norepinephrine-dopamine reuptake inhibitor; OCD = obsessive compulsive disorder; PTSD = posttraumatic stress disorder; MDD = major depressive disorder.

[a] WL vs. RET [$\chi^2_{(1)} = 6.107$, p = 0.01]. [b] Actual number of cases, not percentage.

Discussion

The findings of this preliminary trial, including remission, significant worry symptom reduction, near-perfect adherence and compliance to the exercise intervention, and the absence of musculoskeletal injuries and adverse events, suggest that exercise training is a feasible, safe, and well-tolerated short-term treatment option or potential adjuvant therapy for sedentary women diagnosed with GAD. These results may be of particular importance given the evidence that exercise is as cost-effective as cognitive behavioral therapy (CBT) in treating GAD symptoms [20] and that CBT is more cost-effective than pharmacotherapy [21]. Widespread access to CBT is

Downloaded by:
Claude Moore Health Sciences Lib - Univ of Virginia
128.143.23.241 - 9/23/2013 3:57:31 AM

Table 2. Worry symptom scores (PSWQ) and effects of RET and AET

Outcome PSWQ	Baseline	Week 2		Week 4		Week 6	
	mean ± SD	mean ± SD	Hedges' d 95% CI	mean ± SD	Hedges' d 95% CI	mean ± SD	Hedges' d 95% CI
RET	63.80 ± 9.78	59.83 ± 9.93	0.33<br>–0.56 to 1.21	60.30 ± 8.29	0.30<br>–0.58 to 1.19	61.10 ± 10.01	0.45<br>–0.45 to 1.33
AET	62.10 ± 6.40	63.20 ± 4.16	0.23<br>–1.10 to 0.65	58.80 ± 5.20	0.28<br>–0.60 to 1.16	59.30 ± 7.38	0.45<br>–0.44 to 1.34
WL	64.30 ± 7.01	63.40 ± 8.91		63.50 ± 7.04		65.50 ± 7.62	

currently unavailable and would require pervasive policy changes [20].

Moderate-intensity exercise training, as quantified by the RPE ratings for each session, was successful in eliciting remission and improvements in worry symptoms. These and related findings suggest that exercise training is an accessible treatment or adjuvant therapy for GAD that is adoptable, implementable, and characterized by a low risk of adverse events.

The remission rate for RET was significantly higher than that for WL. The WL remission rate was consistent with prior research documenting that only about one third of GAD patients show partial or full remission over a 6-month period [22]. The NNT of 3 suggests that, on average, for every 10 GAD patients who perform 6 weeks of RET 3 additional remissions would be expected to occur compared to the expected number of spontaneous remissions among untreated patients. The NNT for RET compares favorably to the effects of 4–8 weeks of treatment with other empirically-supported GAD treatments including antidepressants (NNT = 2.38–3.23) [23], benzodiazepines (NNT = 2.56) [23], and azapirones (NNT = 4.4) [24].

The finding that AET did not elicit remission suggests that the therapeutic effect of exercise training for this outcome was likely linked to the relative exercise intensity as revealed by the RPE. The AET sessions were perceived as significantly less intense (RPE = 9; 'very light') than the RET sessions (RPE = 14; 'somewhat hard' and 'hard'). These findings are consistent with larger effects of higher-intensity exercise training on depression symptoms [25].

Worry symptoms were significantly reduced following 6 weeks of exercise training. Both exercise conditions produced moderate reductions in worry symptoms (Hedges' d = 0.45). These findings are comparable to moderate-sized effects reported in reviews of empirically-supported GAD treatments including relaxation therapy, cognitive therapy, and CBT [26, 27]. These findings also are consistent with the mean effect of short-duration exercise training programs on anxiety symptoms among patients with a chronic illness [4]. Given the continued interest in knowing the minimum exercise stimulus necessary to elicit mental health benefits, it is important to note that moderate-sized effects resulted from 6-week training protocols in which patients were exposed to the active ingredient of the exercise stimulus for a total of 3 h and 12 min.

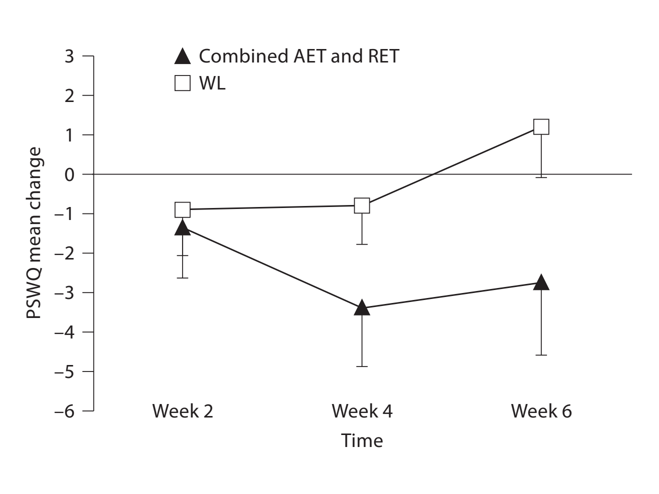

Fig. 2. Changes in worry symptom scores (PSWQ) in the combined AET and RET conditions compared to the WL condition across time.

Downloaded by:
Claude Moore Health Sciences Lib - Univ of Virginia
128.143.23.241 - 9/23/2013 3:57:31 AM

Although the research design was sufficiently powered to detect a statistically significant effect for the primary outcome, the small sample size, short treatment duration, and predominantly young-adult sample limit the generalizability of the present findings. Another potential limitation is that, because of initial strength differences, the exercise training conditions were not ideally equated on positive work; consequently, differences between AET and RET cannot be completely ruled out. Nonetheless, favorable effects on remission and worry symptoms were found regardless of the exercise condition.

A better understanding of the efficacy of exercise as a potential treatment for GAD could be realized through well-designed investigations that: (1) use large samples sizes to compare exercise effects to empirically-supported treatments for GAD, (2) compare the effects of different types of exercise that use different intensities and durations matched for perceptual responses during the time actively engaged in exercise to better understand the minimal and optimal dose necessary to improve symptoms, and (3) block randomize patients to conditions based on potential confounding variables including comorbid psychiatric diagnoses. Notwithstanding the need for more and better clinical trials of exercise training among anxiety patients, these initial findings encourage continued investigation of plausible cognitive or biological mechanisms that might explain antianxiety effects of exercise [28], including a reduction in the allostatic load and neurotrophic or neuroprotective effects [29].

Preliminary findings suggest that exercise training, including RET, is a feasible, well-tolerated intervention that can reduce worry symptoms among GAD patients and may be an effective adjuvant, short-term treatment, or augmentation [30] for GAD. Further investigation of exercise training effects on patients is warranted.

Acknowledgments

This research was supported by a grant from The University of Georgia's College of Education. The authors would like to thank Olivia Barkett, Kristin Espiau, Darren Gillman, Sean Hesson, Robert Leibman, Allyce Naeger, Akil Piggot, Brad Schwartz, Kristel Thomassin, Matthew Weintraub, and Lina Vayner for their assistance with data collection.

References

1 Broocks A, Bandelow B, Pekrun G, George A, Meyer T, Bartmann U, Hillmer-Vogel U, Ruther E: Comparison of aerobic exercise, clomipramine, and placebo in the treatment of panic disorder. Am J Psychiatry 1998;155: 603–609.

2 Merom D, Phongsavan P, Wagner R, Chey T, Marnane C, Steel Z, Silove D, Bauman A: Promoting walking as an adjunct intervention to group cognitive behavioral therapy for anxiety disorders – a pilot group randomized trial. J Anxiety Disord 2008;22: 959–968.

3 Goodwin RD: Association between physical activity and mental disorders among adults in the United States. Prev Med 2003;36:698–703.

4 Herring MP, O'Connor PJ, Dishman RK: Exercise training reduces anxiety symptoms among patients: a systematic review. Arch Intern Med 2010;170:321–331.

5 Kendler KS, Neale MC, Kessler RC, Heath AC, Eaves LJ: Generalized anxiety disorder in women: a population-based twin study. Arch Gen Psychiatry 1992;49:267–272.

6 Puetz TW, O'Connor PJ, Dishman RK: Effects of chronic exercise on feelings of energy and fatigue: a quantitative synthesis. Psychol Bull 2006;132:866–876.

7 Colcombe S, Kramer AF: Fitness effects on the cognitive function of older adults: a meta-analytic study. Psychol Sci 2003;14: 125–130.

8 Martinsen EW, Hoffart A, Solberg OV: Aerobic and non-aerobic forms of exercise in the treatment of anxiety disorders. Stress Med 1989;5:115–120.

9 Brown T, Di Nardo P, Barlow DH: Anxiety Disorders Interview Schedule Adult Version (ADIS-IV): Client Interview Schedule. Albany, Graywind Publications, 1994.

10 Zimmerman M, Mattia, JI: A self-report scale to help make psychiatric diagnoses: the Psychiatric Diagnostic Screening Questionnaire. Arch Gen Psychiatry 2001;58:787–794.

11 Meyer TJ, Miller ML, Metzger RL, Borkovec TD: Development and validation of the Penn State Worry Questionnaire. Behav Res Ther 1990;28:487–495.

12 Blair S, Haskell W, Ho P, Paffenbarger RS, Vranizan KM, Farquhar JW, Wood PD: Assessment of habitual physical activity by a seven-day recall in a community survey and controlled experiments. Am J Epidemiol 1985;122:794–804.

13 Borg G: Borg's Perceived Exertion and Pain Scales. Champaign, Human Kinetics, 1983.

14 Cook D, O'Connor P, Eubanks S, Smith J, Lee M: Naturally occurring muscle pain during exercise: assessment and experimental evidence. Med Sci Sports Exerc 1997;29:999–1012.

15 Brown TA, Di Nardo PA, Lehman CL, Campbell LA: Reliability of DSM-IV anxiety and mood disorders: Implications for the classification of emotional disorders. J Abnorm Psychol 2001;110:49–58.

16 Brown TA, Antony MM, Barlow DH: Psychometric properties of the Penn State Worry Questionnaire in a clinical anxiety disorders sample. Behav Res Ther 1992;30: 33–37.

17 Beck AT, Steer RA, Brown GK: Manual for the Beck Depression Inventory-II. San Antonio, Psychological Corporation, 1992.

18 Cook RJ, Sackett DL: The number needed to treat: a clinically useful measure of treatment effect. BMJ 1995;310:452–454.

19 Hedges LV, Olkin I: Statistical Methods for Meta-Analysis. New York, Academic Press, 1985.

20 McCrone P, Ridsdale L, Darbishire L, Seed P: Cost-effectiveness of cognitive behavioral therapy, graded exercise and usual care for patients with chronic fatigue in primary care. Psychol Med 2004;34:991–999.

Downloaded by:
Claude Moore Health Sciences Lib - Univ of Virginia
128.143.23.241 - 9/23/2013 3:57:31 AM

21 Heuzenroeder L, Donnelly M, Haby MM, Mihalopoulos C, Rossell R, Carter R, Andrews G, Vos T: Cost-effectiveness of psychological and pharmacological interventions for generalized anxiety disorder and panic disorder. Aust N Z J Psychiatry 2004; 38:602–612.
22 Wittchen H-U, Zhao S, Kessler R, Eaton WW: DSM-III-R generalized anxiety disorder in the National Comorbidity Survey. Arch Gen Psychiatry 1994;51:355–364.
23 Hackett D, Haudiquet V, Salinas E: A method for controlling for a high placebo response rate in a comparison of venlafaxine XR and diazepam in the short-term treatment of patients with generalised anxiety disorder. Eur Psychiatry 2003;18:182–187.
24 Chessick CA, Allen MH, Thase M, Batista Miralha da Cunha AB, Kapczinski FF, de Lima MS, dos Santos Souza JJ: Azapirones for generalized anxiety disorder. Cochrane Database Syst Rev 2006;3:CD006115.
25 Singh NA, Stavrinos TM, Scarbek Y, Galambos G, Liber C, Singh MAF: A randomized controlled trial of high versus low intensity weight training versus general practitioner care for clinical depression in older adults. J Gerontol A Biol Sci Med Sci 2005;60:768–776.
26 Gould RA, Safren SA, Washington DO, Otto MW: A meta-analytic review of cognitive behavioral treatments; in Heimber R, Turk CL, Mennin DS (eds): Generalized Anxiety Disorder: Advances in Research and Practice. New York, Guilford Press, 2005, pp 248–264.
27 Hofmann SG, Sawyer AT, Witt AA, Oh D: The effect of mindfulness-based therapy on anxiety and depression: a meta-analytic review. J Consult Clin Psychol 2010;78:169–183.
28 Dishman RK, Berthoud HR, Booth FW, Cotman CW, Edgerton VR, Fleshner MR, Gandevia SC, Gomez-Pinilla F, Greenwood BN, Hillman CH, Kramer AF, Levin BE, Moran TH, Russo-Neustadt AA, Salamone JD, Van Hoomissen JD, Wade CE, York DA, Zigmond MJ: Neurobiology of exercise. Obesity 2006;14:345–356.
29 Sylvia LG, Ametrano RM, Neirenberg AA: Exercise treatment for bipolar disorder: potential mechanisms of action mediated through increased neurogenesis and decreased allostatic load. Psychother Psychosom 2010;79:87–96.
30 Trivedi MH, Greer TL, Grannemann BD, Church TS, Galper DI, Sunderajan P, Wisniewski SR, Chambliss HO, Jordan AN, Finley C, Carmody TJ: TREAD: TReatment with Exercise Augmentation for Depression: study rationale and design. Clin Trials 2006; 3:291–305.

Downloaded by:
Claude Moore Health Sciences Lib - Univ of Virginia
128.143.23.241 - 9/23/2013 3:57:31 AM
