# Supplementary material for: Does physical activity-based intervention decrease repetitive negative thinking? A systematic review
Source: PLoS One. 2025 Apr 1;20(4):e0319806. doi: 10.1371/journal.pone.0319806 (PMC11960971; doi:10.1371/journal.pone.0319806)
Supplement: S1 File — https://doi.org/10.6084/m9.figshare.25711734. (ZIP) [file pone.0319806.s001.zip › supporting information/paper file/Shrimal2024.pdf]

Work 79 (2024) 73–82
DOI:10.3233/WOR-230061
IOS Press

Impact of Yoga on anxiety, stress and sleep quality among health care professionals during a public health crisis

Pragya Jain Shrimal[a], Satyapriya Maharana[b,*], Anupama Dave[c], Kashinath G. Metri[d,1], Nagarathna Raghuram[e] and Shivendra Shrimal[f]
[a]*Department of Yoga & Life Science, SVYASA Deemed to be University, Bengaluru, India*
[b]*Division of Yoga & Life Sciences, SVYASA Deemed to be University, Bengaluru, India*
[c]*Department of Obstetrics & Gynaecology, M.Y. Hospital & MGM Medical College, Indore, India*
[d]*Department of Yoga, Central University of Rajasthan, Ajmer, India*
[e]*Arogyadhama, SVYASA Deemed to be University, Bengaluru, India*
[f]*ESIC Model Hospital Indore, Ministry of Labour and Employment, Government of India, Indore, India*

Received 9 February 2023
Accepted 26 January 2024

Abstract.
BACKGROUND: Health care professionals (HCPs) working on the frontlines of health care emergencies/pandemics often experience elevated anxiety, stress and reduced quality of sleep. Yoga is a mind-body practice that has been proven to effective in improving physical and mental health.
OBJECTIVE: To evaluate the effectiveness of integrated Yoga intervention on mental health measures and sleep quality among HCPs working during the COVID-19 pandemic.
METHODS: This quasi-experimental study was conducted on 110 HCPs, including Doctors and nursing staff, with an age range of 20 to 65 years who were recruited through a convenience sampling method and assigned to a Yoga group ($n = 55$) or a control group ($n = 55$). The Yoga group received a weekly one-hour Yoga session followed by a 30-minute self-practice three times a week at home for one month. The control group followed routine activity. Heart rate (HR), Blood Pressure, stress measures (Perceived Stress and visual analogue Scale for Stress), COVID-related anxiety, Sleep Quality, Quality of life and Preservative Thinking were assessed before and after the intervention.
RESULTS: A significant decrease in stress measures, COVID-19-related anxiety and fear, and perseverative thinking was noticed in the Yoga group. Sleep quality and quality of life also improved significantly. The control group showed no significant change. At one month, the Yoga group showed significantly greater improvement in stress measures, COVID-19-related anxiety and fear, perseverative thinking, sleep quality and quality of life compared to the control group.
CONCLUSION: Yoga practice during the public health crisis would help reduce anxiety and stress and improve sleep among HCPs. Further randomized controlled trials are warranted.

Keywords: Mental health, healthcare, sleep, mind-body medicine

[1]E-mail: kashinath@curaj.ac.in.
*Address for correspondence: Dr. Satyapriya Maharana, Ph.D, SVYASA Deemed to be University, #19 EknathBhavan, Gavipuram Circle, Kempegowda Nagar, Bengaluru 560019, India. E-mail: trisatyapriya77@gmail.com.

ISSN 1051-9815/$35.00 © 2024 – IOS Press. All rights reserved.

1. Introduction

The medical profession is a noblework. Health care professionals (HCPs) in hospitals must have tremendous patience, dedication and a friendly attitude. HCPs working on the front lines of critical care during community infectious diseases/pandemic situations, in the community etc., are working hard and more additional hours than usual. Furthermore, such emergencies are often demanding and lead to an increase in stress and anxiety among healthcare professionals. Emergencies, such as a pandemic, sometimes last several months to years and significantly affect healthcare professionals' mental and physical health [1, 2].

Recent survey reports during the COVID-19 pandemic have shown that anxiety and stress among HCPs have increased during the pandemic.In a survey study, among 153 HCPs, 43.13% had anxiety, 47.05% had fear, and 30.17% had psychological stress during COVID-19 [3]. A systematic review and meta-analysis of 38 studies found that the global prevalence of mental health problems during the COVID-19 pandemic among healthcare professionals was 49% (anxiety - 40%, depression - 37% and despair - 37%) [4]. Another systematic review and meta-analysis of 239 studies ($n = 271{,}319$) showed that 33% of HCPs suffered from depressive symptoms, 42% from anxiety, 40% from acute stress, 32% from post-traumatic symptoms, and 42% from insomnia burnout [5]. Further, Studies among nurses reported high levels of stress and low sleep quality during the pandemic [6]. These studies suggest that healthcare professionals are at increased risk of anxiety, depression and stress in pandemic situations. In addition to this, studies have strongly recommended implementing effective interventions [7].

Psychopathologies such as anxiety and stress have deleterious effects on physiology and immune functions [8]. Anxiety, depression and stress affect the immune system and increase the risk of infections and other chronic health problems such as high blood pressure, heart disease, autoimmune diseases, etc. Stress and anxiety are associated with an increased risk of an acute cardiac event in healthcare professionals. Therefore, taking some effective measures to minimize stress and anxiety among healthcare professionals, especially those working in critical care units/during pandemic situations, is crucial. Medical professionals responding to healthcare emergencies require mental and emotional support. Furthermore, effective intervention to prevent psychopathology is crucial for HCPs in challenging situations [9]. Many complementary therapies such as music, massage, Yoga and exercise have been tried in addition to pharmacological interventions to reduce psychological problems in HCPs [10].

Yoga is a mind-body intervention that includes Yoga poses, breathing exercises, cleansing techniques, and meditation [11]. Yoga is an effective intervention to improve various mental health measures, including stress, depression, and anxiety [12]. Yoga has positive effects on the mental health of nurses [13]. There is evidence that Yoga improves the mental health of caregivers and nurses. It also helps improve cardiac risk factors. Yoga-based breathing techniques have been shown to be feasible and acceptable in HCPs [14, 15]. However, the literature indicates that no study has evaluated the effects of Yoga on the mental health of HCPs working during emergencies such asa pandemic.

This study intended to evaluate the effects of Yoga on mental health measures among HCPs during the COVID-19 pandemic.

2. Methodology

2.1. Study design

Quasi-experimental study. Although a randomized controlled trial would be the best design, this study adopted a quasi-experimental design, given the ongoing pandemic. Furthermore, these healthcare professionals had limited time to undergo intervention, and some were unwilling to undergo Yoga intervention.

2.2. Participants

Participants in this study were HCPs (physicians, nursing staff and nursing orderly) working in tertiary care COVID-19 hospitals.

2.2.1. Sample source

The study was conducted in a tertiary care hospital in North India. A notice was posted on the notice boards of the hospital and sent via email and messages to the HCP at the hospital, requesting volunteer participation. A virtual interaction session was organized to increase participation.

2.2.2. Sample size

A sample size of 100 was determined using the "G Power" software (alpha = 0.05, power = 0.95 and

effect size = 1.10). The sample size was calculated from a previous intervention study [16] comparing the effect of progressive muscle relaxation on anxiety.

2.3. Inclusion and exclusion criteria

The inclusion criteria for the study were: HCPs working in the COVID-19 healthcare facility, aged between 20 and 65 years, of any gender.

Participants were excluded if they had tested positive for COVID-19, had a psychiatric diagnosis, were taking long-term medications, had compensatory cardio-respiratory diseases, had recently undergone surgery, and were pregnant.

One-hundred-forty potential participants were screened for eligibility criteria. Twenty-two participants had exclusion criteria, and eight were transferred to another hospital before recruitment. A total of 110 participants completed the baseline assessment. Fifty-five participants were in the Yoga group, and 55 were in the waitlist control group. No randomization was performed.

2.4. Intervention

The Yoga group received a 60-minute structured Yoga session every Sunday. In addition, participants were provided flowcharts, diagrams, and audio-visual aids of 30-minute Yoga sessions and were encouraged to practice on their own at home at least three days a week. They were asked to keep a logbook to record the frequency of self-exercise. The integrated Yoga intervention consisted of breathing exercises, *Surya Namaskara, asanas, pranayama*, relaxation techniques and meditation [17] (see in Table 1). Participants in the waitlist control group were asked to follow their daily routine as usual. The waitlist control group received no intervention. The control group was monitored for COVID-19 infection.

2.5. Outcomes

The assessment was done at the baseline and one month later for both groups. The psychological outcomes, including stress, anxiety, fear, repetitive negative thinking, quality of life and sleep were assessed through self-administered questionnaires. Additionally, trained nursing staff measured physiological outcomes such as heart rate and systolic and diastolic blood pressure.

2.6. Assessment tools

2.6.1. Primary outcome measures (psychological outcomes)

Perceived Stress Scale (PSS) was used to assess stress. The PSS has 10 items which measure the extent to which a person perceives life situations as stressful during the last month [18].

Visual Analog Scale (VAS) was used to assess stress. VAS is used for the clinical assessment of self-reported stress. This scale ranges from 0 to 100, where 0 indicating ’no stress’ and 100 designating stress as ‘as bad as possible’ [19].

The Corona virus Anxiety Scale (CAS) is a 5-item scale to measure COVID-19-related anxiety. This scale demonstrated solid reliability (0.93) and validity [20].

The Fear of COVID-19 Scale (FCV-19S) is a self-administered questionnaire. It consists of seven items that measure fear related to COVID-19. Its strong

Table 1
Details of the practice done by the yoga group

Type of activity	List of practices
Loosening exercise (Shithilikarana Vyayama)	Spinal twisting (1 min), Forward and backward bending (1 min), Mukha Dhouti (1/2 min), Surya Namaskar (3 rounds, 3 min)
Breathing Practices	Hands in and out Breathing (1 min), Hands stretch breathing (1 min each variation), Tiger Breathing (1 min)
Asanas	**Standing:** Padahastasana (1 min)<br>**Sitting:** Vakrasäna (1 min)<br>**Prone:** Bhujangäsana (1 min)<br>**Supine:** Sulabha Matsyasana (1 min)
Relaxation techniques	Quick relaxation technique/ Deep relaxation technique (3 mins)
Pranayama	Abdominal breathing (2 min), Bhrastrika (30 strokes, 2 rounds, 2 min), Nadishodhanä (3 min)
Meditation	Sun meditation (3 min)

content and construct validity featured a Cronbach's alpha of 0.82. Its test-retest reliability was substantiated by an intra-class Correlation Coefficient (ICC) of 0.72 [21].

The Pittsburgh Sleep Quality Index (PSQI) consists of 19 items that produce a global sleep quality score and 7 component scores: sleep quality, sleep latency, sleep duration, habitual sleep efficiency, sleep disturbance, use of sleeping medications, and daytime dysfunction [22]. These seven component scores yield a global score of subjective sleep quality (range 0–21).PSQI is a widely used tool for assessing sleep quality, with a Cronbach's alpha of 0.80, indicating strong internal consistency. The PSQI has demonstrated moderate to substantial test-retest reliability (ranging from 0.69 to 0.77) [23].

The quality of life (QoL5) questionnaire is a five-scale questionnaire. QoL5 has acceptable construct validity, external reliability, sensitivity, and internal reliability, with Cronbach's alpha of 0.69 and test-retest correlation of 0.82 [24].

Perseverative Thinking Questionnaire (PTQ) has 15 items that measure an individual's repetitive negative thinking on a 4 Likert scale from 0–4, with 0 being never and four being almost always [25]. PTQ was utilized to measure preservative thinking patterns, characterized by high internal consistency (Cronbach's alpha = 0.95). The questionnaire has demonstrated acceptable test-retest reliability at 0.69 [25].

2.6.2. Physiological outcomes

Systolic and diastolic blood pressure and heart rate were measured using the Omron Automatic Blood Pressure Monitor (HEM-7120), an electronic blood pressure measuring instrument.

2.7. Statistical analysis

The data was presented in mean and standard deviation. A *p*-value of less than 0.05 was considered a statistically significant change. SPSS Version 21 was used for data analysis. The chi-squared and Independent samples *t*-test was used for baseline comparison between groups. The Mann-Whitney test (between groups) was used for statistical analysis as the data were not normally distributed.

3. Results

3.1. Participation

Ninety-one participants completed the study. Thirteen participants (5 in the Yoga group and 8 in the control group) tested positive for COVID-19 midway through the study. Three participants did not do self-practice, and three participants in the control group were lost to follow-up. Figure 1 shows the model in which the study was conducted.

3.2. Demographic characteristics

At baseline, both groups were comparable in terms of age range, gender and health worker categories (see Table 2).

3.3. Changes in physiological outcomes

After one month, there was a significant decrease in heart rate and systolic blood pressure compared to baseline in the Yoga group. The control group showed no change. In between-group comparison, the one-month Yoga group showed a significantly greater reduction in heart rate and SBP than the control group. No significant change in the DBP was seen (see Table 3).

3.4. Changes in psychological outcomes

Compared to the baseline, the Yoga group showed a significant decrease in perseverative thinking, Corona-related fear, anxiety and stress after one month. Sleep quality and quality of life also improved significantly. The control group showed no change (see Table 4 and Figs. 2–7).

At the one-month between-group comparison, the Yoga group showed a significantly greater reduction in fear, perceived stress, perseverative thinking, and corona-related anxiety compared to the control group. In addition, a significant improvement in sleep quality and quality of life was observed in the Yoga group compared to the control group.

4. Discussion

HCPs often suffer from work-related stress. Emergencies and critical situations, such as pandemics, often increase stress and anxiety. Recent survey reports suggest that healthcare professionals experi-

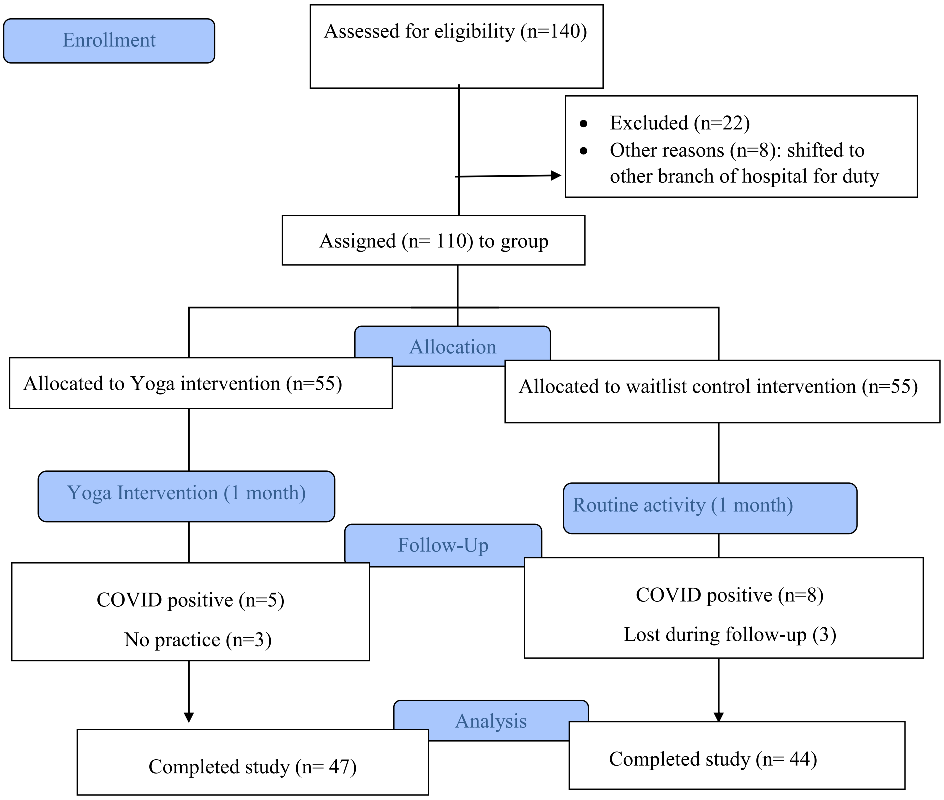

Fig. 1. Flow diagram for study design.

Table 2
Demographic characteristics of the Yoga and control groups

	Variables	Yoga $n = 47$	Control $n = 44$	P values (Chi-square test)
Gender*	Male	21(42%)	17 (34%)	0.016
	Female	23 (58%)	30 (66%)	
Age range* (Years)	25–35	33 (33%)	34 (68%)	0.148
	36–45	9 (18%)	14 (28%)	
	46–60	8 (16%)	2 (4%)	
Age	Age (Mean/SD)	34 ± 10 years	32 ± 7 years	0.148
Category*	Doctors	25	27	0.179
	Nursing staff	15	15	
	Nursing orderly	4	5	

Note. Non-significant ($p > 0.05$) difference between groups in all variables. Gender, age range, category of health care professionals. *Chi-squared test; other variables – Independent samples t-test.

Table 3
Changes in physiological outcomes in the two groups after one month

Variable	Group	Baseline	At 1-month	Sig- P between group	
		Mean ± SD	Mean ± S.D	Pre/pre	Post/post
HR	Y	76.96 ± 5.86	72.84 ± 3.89*	0.196	0.001^{∞}
	C	75.38 ± 5.54	76.84 ± 4.70		
BP_SYS	Y	120.36 ± 8.97	116.28 ± 8.03*	0.404	0.001^{∞}
	C	120.76 ± 6.79	122.76 ± 4.71		
BP_DIA	Y	76.24 ± 4.45	72.92 ± 3.70	0.340	0.08^{∞}
	C	75.12 ± 4.48	76.72 ± 4.69		

*$P < 0.001$: Wilcocson's Signed Rank test; $^{\infty}$Mann-Whitney Test. Abbreviations: HR-Heart Rate, BP-Blood Pressure, SYS-Systolic, DIA-Diastolic.

Table 4
Changes in psychological outcomes in the two groups after one month

Variable	Group	Baseline	At 1-month	Between groups (*P* value)	
		Mean ± SD	Mean ± SD	Pre/pre	Post/post
VAS for stress	Y	55.20 ± 10.15	18.20 ± 7.19*	0.60	0.001$^{\infty}$
	C	55.60 ± 12.31	48.40 ± 7.91		
PTQ	Y	32.12 ± 3.03	8.46 ± 1.69*	0.96	0.001$^{\infty}$
	C	32.08 ± 1.99	26.14 ± 2.84		
CAS	Y	12.76 ± 1.76	2.16 ± 0.99*	0.107	0.001$^{\infty}$
	C	12.24 ± 1.33	9.58 ± 1.16		
FCS	Y	22.04 ± 2.79	7.86 ± 0.78*	0.68	0.001$^{\infty}$
	C	22.12 ± 1.89	19.46 ± 1.72		
	C	56.60 ± 8.15	67.86 ± 3.79		
PSS	Y	23.98 ± 2.91	4.96 ± 1.55*	0.54	0.001$^{\infty}$
	C	24.22 ± 2.66	22.14 ± 1.72		
PSQI	Y	11.32 ± 2.66	8.76 ± 1.97*	0.72	0.001$^{\infty}$
	C	11.22 ± 2.15	10.38 ± 1.88		
QoL	Y	55.26 ± 8.85	87.33 ± 3.08*	0.64	0.001$^{\infty}$
	C	56.60 ± 8.15	67.86 ± 3.79		

*$P < 0.001$: Wilcocson's Signed Rank test; $^{\infty}$Mann-Whitney Test. Abbreviations: VAS (Stress): Visual Analogue Scale, PTQ: Perseverative Thinking Questionnaire, CAS: Coronavirus Anxiety Scale, FCS: Fear of Covid-19 Scale, QoL: Quality of Life, PSS: Perceived Stress Scale, PSQI: Pittsburgh Sleep Quality Index, Y: Yoga, C: Control.

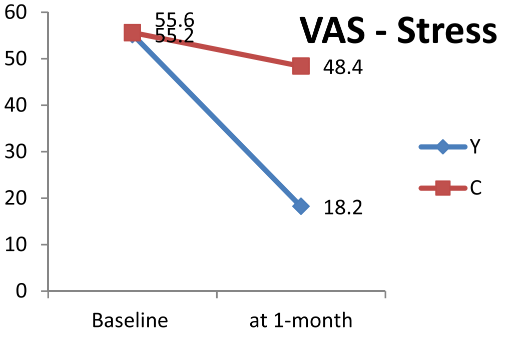

Fig. 2. Significant decreased in Visual Analogue Scale in the Yoga group. No significant change in the control group.

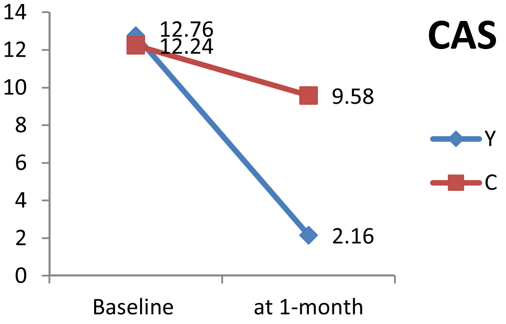

Fig. 3. Significant decrease in anxiety-related COVID-19 in the Yoga group at 1-month compared to baseline. The control group showed no significant change.

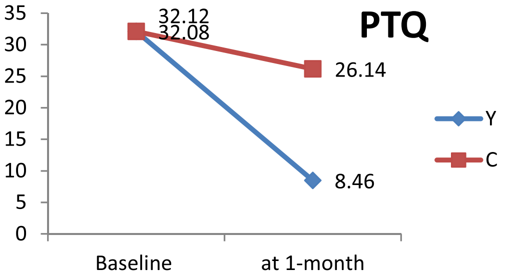

Fig. 4. Significant decrease in perseverative thinking in the Yoga group at 1-month compared to baseline. No change in the control group noted.

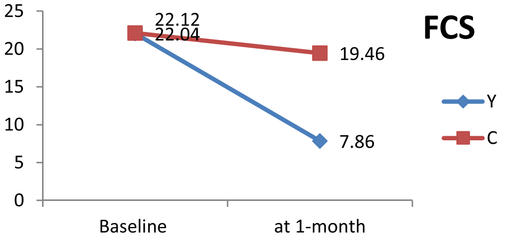

Fig. 5. Significant decrease in fear of COVID-19 in the Yoga group at 1-month compared to baseline. No change in the control group noted.

enced higher rates of anxiety and depression during the COVID-19 pandemic. At the beginning of the study, all participants in both groups had significant sleep disorders as well as increased levels of anxiety and stress.

The present study demonstrated the role of Yoga interventions in reducing anxiety and stress among healthcare professionals during the COVID-19 pan-

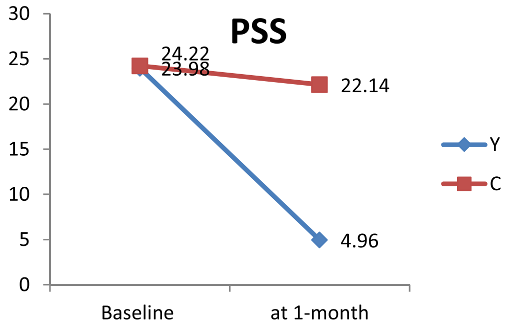

Fig. 6. Significant decrease in perceived stress was noted in the Yoga group at 1-month compared to baseline. No change in the control group noted.

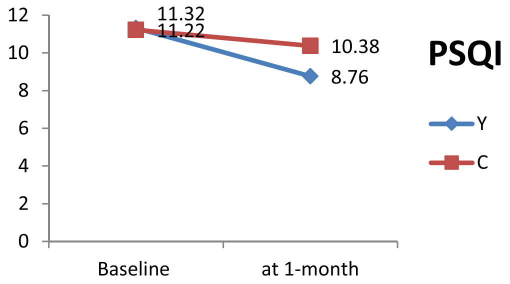

Fig. 7. Significant improvement in sleep quality in the Yoga group at 1-month compared to baseline. No change in the control group noted.

demic. This study suggests that Yoga helps reduce anxiety and stress and improves sleep quality in healthcare providers during healthcare emergencies. In addition to anxiety and depression, a significant reduction in blood pressure and heart rate was also noted. Furthermore, the significant improvement in quality of life was also observed in the Yoga group.

The COVID-19 pandemic and the imposed lockdown affected the quality of life of healthcare workers who had to carry out their duties in COVID-19 hospitals. The studies have reported, the effectiveness of yoga in managing stress and improving quality of life [26]. The results of the present study showed a significant improvement in quality of life after a Yoga intervention. A study by Rostami et al. 2019 on nurses working in intensive care units (ICU) showed that Yoga interventions improved nurses' quality of life [27]. However, this study was not conducted during an ongoing pandemic or crisis.Whereas the present study was during the COVID-19 pandemic. In a study by Pizzoli et al. from 2020, web-based interventions were also offered to the general population. The result showed that web-based interventions are effective, cost-effective and help reduce worry and stress [28]. Our intervention was also effective and could be carried out despite the restrictions caused by COVID-19. Yoga is effective in reducing anxiety and stress [29]. Previous studies on Yoga have shown that it positively affects the mental health of healthcare professionals [26]. Previously, in a study by La Torre et al. in 2020, a month-long Yoga and mindfulness program showed a significant reduction in anxiety and stress among healthcare workers [12]. The results of the present study are consistent with the previous study (La Torre). However, one study by La Torre was a single-arm study, and the intervention was performed once a week. Furthermore, this study was not conducted during a pandemic situation. It also helps improve sleep quality.

The results of the present study are also consistent with previous studies. Yoga is effective in improving sleep. In a single-arm study by Samuel et al., 43 healthcare professionals received eight weeks of supervised, mindfulness-based workplace activities. After eight weeks, significant improvement was observed in personal performance, depression, anxiety, stress, perceived resilience and compassion compared to baseline [30, 31]. The results of the present study are similar to those of previous studies. However, the present study is a two-arm study. Additionally, a reduction in objective physiological measures such as blood pressure and heart rate is the objective evidence of a change in subjective measures such as anxiety, depression, stress and sleep quality.

Yoga is a mind-body technique. It includes mindful practice of various Yoga postures, breathing techniques and meditation. This helps practitioners to be mindful and non-judgmental with their thoughts, emotions and behaviours. It also helps reduce the number of thoughts and intensity of negative thoughts and emotions and promotes relaxation. This helps to reduce anxiety, stress and depression. Additionally, Yoga poses involve stretching voluntary muscles, increasing parasympathetic activity as a stretch response [32]. Deep and slow breathing during pranayama practice helps down-regulate the HPA axis which is associated with reduced stress response and anxiety [33, 34].

In order to achieve the desired effects, the intervention should include slow movements and switching from one asana to another, as well as slow breathing techniques, relaxation techniques and meditation practice.

Although the mechanism of Yoga is not examined in the present study, the authors can speculate that improved psychological measures after Yoga

intervention may be due to decreased HPA axis hyperactivity and sympathetic tone [35, 36]. Additionally, Yoga interventions are associated with increased production of serotonin and melatonin, [37, 38] which help improve mood and sleep quality. Yoga helps improve awareness and mindfulness in everyday activities, which can help with self-awareness and prevent self-harming feelings and thoughts.

Although this study was performed during the COVID-19 pandemic and the world has moved out of COVID-19 at present, the results of the present study signify the usefulness of Yoga in reducing stress, anxiety and depression and improving sleep quality of healthcare care professionals who work in the emergency care unit. Furthermore, the results of the present study also signify the importance of Yoga among frontline workers in other situations, such as natural disasters.

The limitations of this study are that it is a non-randomized controlled trial (RCT) study (an RCT study with an active control group would have demonstrated more confirmative results); the lack of objective measurements; objective measures such of stress/anxiety/depression such as salivary cortisol level, heart rate variability were not included; and the small sample size.

5. Conclusion

Long-term emergencies such as pandemics often increase stress and anxiety levels in healthcare professionals, affecting their quality of sleep and quality of life. The present study shows that Yoga practice during hectic work/pandemic situations would help reduce anxietyand depression and improve healthcare professionals' sleep and quality of life. Further, RCT studies with longer-term interventions are warranted.

Ethics statement

The study was approved by the Institutional Ethics Committee of SVYASA University, Bengaluru (IEC No. RES/IECSVYASA/180/2020/C).

Informed consent was obtained from all participant's before baseline assessment.

The study was registered in the Clinical Trails Registry - India (CTRI registration number: CTRI/2020/10/028485, date: 20/10/2020; http://www.ctri.nic.in/).

Acknowledgments

We are thankful to all participants of this study for their cooperation during the project. We express our sincere gratitude to the Doctors, management, and nursing staff of MTH, Hospital, Indore, Madhya Pradesh, India.

Author contributions

PJS, SM, and RN conceived the conception and study idea. AD, SS contributed to data collections. KM, SM, and RN performed the data analysis. PJS, KM, AD, SM and SS wrote the manuscript. RN, KM and SS performed the final proofreading and corrections. All authors read and approved the manuscript.

Conflict of interest

There is no conflict of interest among the authors of this research trial.

Funding

No funding in any form was received for this research.

References

[1] Wu W, Zhang Y, Wang P, Zhang L, Wang G, Lei G, Xiao Q, Cao X, Bian Y, Xie S, Huang F. Psychological stress of medical staffs during outbreak of COVID-19 and adjustment strategy. Journal of medical virology. 2020;92(10): 1962-70.

[2] Ozamiz-Etxebarria N, Dosil-Santamaria M, Picaza-Gorrochategui M, Idoiaga-Mondragon N. Niveles de estrès, ansiedad y depresión en la primera fase del brote del COVID-19 en una muestra recogida en el norte de España. Cadernos de saude Publica. 2020;36(4):e00054020.

[3] Fadhel FH, Alqahtani MM, Arnout BA. Working with patients and the mental health of health care workers during the COVID-19 pandemic. Work. 2022;72(1):27-38.

[4] Saragih ID, Tonapa SI, Saragih IS, Advani S, Batubara SO, Suarilah I, Lin CJ. Global prevalence of mental health problems among healthcare workers during the Covid-19 pandemic: A systematic review and meta-analysis. International Journal of Nursing Studies. 2021;121:104002.

[5] Aymerich C, Pedruzo B, Pèrez JL, Laborda M, Herrero J, Blanco J, Mancebo G, Andrès L, Estèvez O, Fernandez M, de Pablo GS. COVID-19 pandemic effects on health worker's mental health: Systematic review and meta-analysis. European Psychiatry. 2022;65(1):e10.

[6] Bilgiç Ş, Çelikkalp Ü, Mısırlı C. Stress level and sleep quality of nurses during the COVID-19 pandemic. Work. 2021;70(4):1021-9.

[7] Zhang Y, Mo Q, Tan C, Hu J, Zhao M, Xiong X, Zhang J. Analysis of the mental health status of hospital staff during the COVID-19 pandemic. Work. 2023 May 20(Preprint):1-0.

[8] Sharma K, Anand A, Kumar R. The role of Yoga in working from home during the COVID-19 global lockdown. Work. 2020;66(4):731-7.

[9] Yesse M, Muze M, Kedir S, Argaw B, Dengo M, Nesre T, Hamdalla F, Saliha A, Mussa T, Kasim I, Kedir A. Assessment of knowledge, attitude and practice toward COVID-19 and associated factors among health care workers in Silte Zone, Southern Ethiopia. PloS one. 2021;16(10): e0257058.

[10] Vajpeyee M, Tiwari S, Jain K, Modi P, Bhandari P, Monga G, Yadav LB, Bhardwaj H, Shroti AK, Singh S, Vajpeyee A. Yoga and music intervention to reduce depression, anxiety, and stress during COVID-19 outbreak on healthcare workers. International Journal of Social Psychiatry. 2022;68(4):798-807.

[11] Sawant RS, Zinjurke BD, Binorkar SV. Preventive aspect of ayurveda and Yoga towards newly emerging disease COVID-19. Journal of Complementary and Integrative Medicine. 2021;18(4):667-78.

[12] La Torre G, Raffone A, Peruzzo M, Calabrese L, Cocchiara RA, D'Egidio V, Leggieri PF, Dorelli B, Zaffina S, Mannocci A, Yomin Collaborative Group. Yoga and mindfulness as a tool for influencing affectivity, anxiety, mental health, and stress among healthcare workers: Results of a single-arm clinical trial. Journal of Clinical Medicine. 2020;9(4):1037.

[13] Behan C. The benefits of meditation and mindfulness practices during times of crisis such as COVID-19. Irish Journal of Psychological Medicine. 2020;37(4):256-8.

[14] Narayanan S, Tennison J, Cohen L, Urso C, Subramaniam B, Bruera E. Yoga-based breathing techniques for health care workers during COVID-19 pandemic: Interests, feasibility, and acceptance. The Journal of Alternative and Complementary Medicine. 2021;27(8):706-9.

[15] Kanchibhotla D, Saisudha B, Ramrakhyani S, Mehta DH. Impact of a yogic breathing technique on the well-being of healthcare professionals during the COVID-19 pandemic. Global Advances in Health and Medicine. 2021;10:2164956120982956.

[16] Liu K, Chen Y, Wu D, Lin R, Wang Z, Pan L. Effects of progressive muscle relaxation on anxiety and sleep quality in patients with COVID-19. Complementary Therapies in Clinical Practice. 2020;39:101132.

[17] Nagarathna R, Nagendra HR, Majumdar V. A perspective on Yoga as a preventive strategy for coronavirus disease 2019. International Journal of Yoga. 2020;13(2):89.

[18] Gamonal Limcaoco RS, Mateos EM, Fernàndez JM, Roncero C. Anxiety, worry and perceived stress in the world due to the COVID-19 pandemic, March 2020. Preliminary results. MedRxiv. 2020:2020-04.19. Lesage FX, Berjot S. Validity of occupational stress assessment using a visual analogue scale. Occupational Medicine. 2011;61(6): 434-6.

[19] Lee SA. Coronavirus Anxiety Scale: A brief mental health screener for COVID-19 related anxiety. Death Studies. 2020;44(7):393-401.

[20] Ahorsu DK, Lin CY, Imani V, Saffari M, Griffiths MD, Pakpour AH. The fear of COVID-19 scale: Development and initial validation. International Journal of Mental Health and Addiction. 2020:1-9.

[21] Hinz A, Glaesmer H, Brähler E, Löffler M, Engel C, Enzenbach C, Hegerl U, Sander C. Sleep quality in the general population: Psychometric properties of the Pittsburgh Sleep Quality Index, derived from a German community sample of 9284 people. Sleep Medicine. 2017;30:57-63.

[22] Carpenter JS, Andrykowski MA. Psychometric evaluation of the Pittsburgh sleep quality index. Journal of Psychosomatic Research. 1998;45(1):5-13.

[23] Lindholt JS, Ventegodt S, Henneberg EW. Development and validation of QoL5 for clinical databases. A short, global and generic questionnaire based on an integrated theory of the quality of life. European Journal of Surgery. 2002;168(2):107-13.

[24] Ehring T, Zetsche U, Weidacker K, Wahl K, Schönfeld S, Ehlers A. The Perseverative Thinking Questionnaire (PTQ): Validation of a content-independent measure of repetitive negative thinking. Journal of Behavior Therapy and Experimental Psychiatry. 2011;42(2):225-32.

[25] Cocchiara RA, Peruzzo M, Mannocci A, Ottolenghi L, Villari P, Polimeni A, Guerra F, La Torre G. The use of Yoga to manage stress and burnout in healthcare workers: A systematic review. Journal of Clinical Medicine. 2019;8(3): 284.

[26] Rostami K, Ghodsbin F. Effect of Yoga on the quality of life of nurses working in intensive care units. Randomized controlled clinical trial. Investigación y educación en enfermería. 2019;37(3).

[27] Pizzoli SF, Marzorati C, Mazzoni D, Pravettoni G. Web-based relaxation intervention for stress during social isolation: Randomized controlled trial. JMIR Mental Health. 2020;7(12):e22757.

[28] Li AW, Goldsmith CA. The effects of Yoga on anxiety and stress. Alternative Medicine Review. 2012;17(1).

[29] Ofei-Dodoo S, Cleland-Leighton A, Nilsen K, Cloward JL, Casey E. Impact of a mindfulness-based, workplace group Yoga intervention on burnout, self-care, and compassion in health care professionals: A pilot study. Journal of Occupational and Environmental Medicine. 2020;62(8): 581-7.

[30] Livingston E, Collette-Merrill K. Effectiveness of integrative restoration (iRest) Yoga nidra on mindfulness, sleep, and pain in health care workers. Holistic Nursing Practice. 2018;32(3):160-6.

[31] Wongwilairat K, Buranruk O, Eungpinichpong W, Puntumetakul R, Kantharadussadee-Triamchaisri S. Muscle stretching with deep and slow breathing patterns: A pilot study for therapeutic development. Journal of Complementary and Integrative Medicine. 2018;16(2):20170167.

[32] Yılmaz ME. The effect of progressive breathing relaxation training on preoperative anxiety and surgical stress response. International Journal of Caring Sciences. 2020;13(2).

[33] Kim SH. Mindfulness-based stretching and deep breathing exercises normalize serum cortisol levels and reverse symptoms of PTSD: A prospective randomized-controlled trial. The University of New Mexico; 2012.

[34] Vempati RP, Telles S. Yoga-based guided relaxation reduces sympathetic activity judged from baseline levels. Psychological Reports. 2002;90(2):487-94.

[35] Sarubin N, Nothdurfter C, Schüle C, Lieb M, Uhr M, Born C, Zimmermannc R, Bühner M, Konopka K, Rupprecht R, Baghai TC. The influence of Hatha Yoga as an add-on treatment in major depression on

hypothalamic–pituitary–adrenal-axis activity: A randomized trial. Journal of Psychiatric Research. 2014;53: 76-83.
[36] Harinath K, Malhotra AS, Pal K, Prasad R, Kumar R, Kain TC, Rai L, Sawhney RC. Effects of Hatha Yoga and Omkar meditation on cardiorespiratory performance, psychologic profile, and melatonin secretion. The Journal of Alternative & Complementary Medicine. 2004;10(2):261-8.
[37] Tooley GA, Armstrong SM, Norman TR, Sali A. Acute increases in night-time plasma melatonin levels following a period of meditation. Biological Psychology. 2000;53(1):69-78.
