# Supplementary material for: Does physical activity-based intervention decrease repetitive negative thinking? A systematic review
Source: PLoS One. 2025 Apr 1;20(4):e0319806. doi: 10.1371/journal.pone.0319806 (PMC11960971; doi:10.1371/journal.pone.0319806)
Supplement: S1 File — https://doi.org/10.6084/m9.figshare.25711734. (ZIP) [file pone.0319806.s001.zip › supporting information/paper file/plag2020.pdf]

Journal of Anxiety Disorders 76 (2020) 102311

Contents lists available at ScienceDirect

Journal of Anxiety Disorders

journal homepage: www.elsevier.com/locate/janxdis

Working out the worries: A randomized controlled trial of high intensity interval training in generalized anxiety disorder

Jens Plag [a,*], Paul Schmidt-Hellinger [b], Theresa Klippstein [a], Jennifer L.M. Mumm [a], Bernd Wolfarth [b], Moritz B. Petzold [a,1], Andreas Ströhle [a,1]

[a] *Charité – Universitätsmedizin Berlin, Corporate Member of Freie Universität Berlin, Humboldt-Universität zu Berlin, and Berlin Institute of Health, Department of Psychiatry and Psychotherapy, Charité Campus Mitte, Berlin, Germany*

[b] *Charité - Universitätsmedizin Berlin, Corporate Member of Freie Universität Berlin, Humboldt-Universität zu Berlin, and Berlin Institute of Health, Department of Sports Medicine, Berlin, Germany*

A R T I C L E I N F O

Keywords:
Generalized anxiety disorder
GAD
Exercise
High intensity interval training

A B S T R A C T

Background: Aerobic exercise (AE) demonstrated an overall medium treatment effect in anxiety disorders (AD) but there is evidence for an “intensity-response” relationship. High intensity interval training (HIIT) was highly effective on a range of (mental) health parameters. However, so far no randomised-controlled trial (RCT) investigated the efficacy of HIIT in AD.

Methods: 33 patients with generalized anxiety disorder (GAD) were randomly assigned to 12-day HIIT or a training of lower intensity (LIT). Anxiety, comorbid depression, stress-related bodily symptoms and perceived control over anxiety related stimuli (PC) were assessed at baseline, post-training and 30 days after baseline by using the Penn State Worry Questionnaire (PSWQ), the Hamilton Inventories for Anxiety and Depression (Ham-A, Ham-D), the Screening for Somatoform Symptoms-7 (SOMS-7) and the Anxiety Control Questionnaire (ACQ-R).

Results: Both interventions showed moderate or large effects on all clinical measures. However, effects for HIIT were generally about twice as high as for LIT. PC negatively correlated with GAD severity in the whole sample at baseline but an association of training-induced changes in PC and worrying were exclusively detectable in HIIT.

Conclusion: HIIT was highly effective and fast acting in GAD. Therefore, it may complement first-line treatment approaches in this condition.

1. Introduction

In recent years, research has progressively focused on the efficacy of physical activity in anxiety disorders. In this context, several randomised controlled trials (RCT) demonstrated that aerobic exercise (AE) is an effective treatment approach for social anxiety disorder, panic disorder (PD), specific phobia and generalized anxiety disorder (GAD) (Bischoff et al., 2018; Broocks et al., 1998; Gaudlitz, Plag, Dimeo, & Ströhle, 2015; Herring, Jacob, Suveg, Dishman, & O’Connor, 2012; Lindenberger et al., 2017; Merom et al., 2008). Although studies in this field differed to some extent regarding type, intensity, frequency and total duration of exercise, recent meta-analyses found an overall medium effect of AE on disorder-specific symptomatology as well as significantly lower drop-out rates in the exercise groups compared to different control conditions (Aylett, Small, & Bower, 2018; Stubbs et al., 2017). Former studies usually established a several-week moderate intensity training (around 70 % of the maximal oxygen uptake [VO_{2max}]) consisting of up to seven units a week (Bischoff et al., 2018; Merom et al., 2008) and a maximum of 30 min per session (e.g. (Gaudlitz et al., 2015)). Interestingly, findings quite consistently show that aerobic training of this intensity led to a significantly larger anxiolytic effect not only in comparison to non-active control groups but also to exercise programs of lower physical strain (e.g. 30 % VO_{2max} or less) (Bischoff et al., 2018; Gaudlitz et al., 2015; Lindenberger et al., 2017). Significant

* Corresponding author at: Department of Psychiatry and Psychotherapy, Campus Charité Mitte Charité - Universitätsmedizin Berlin, Charitéplatz 1, 10117, Berlin, Germany.

E-mail addresses: jens.plag@charite.de (J. Plag), paul-juergen.schmidt@charite.de (P. Schmidt-Hellinger), theresa.klippstein@charite.de (T. Klippstein), jennifer.mumm@charite.de (J.L.M. Mumm), bernd.wolfarth@charite.de (B. Wolfarth), moritz.petzold@charite.de (M.B. Petzold), andreas.stroehle@charite.de (A. Ströhle).

[1] Shared senior authorship.

https://doi.org/10.1016/j.janxdis.2020.102311
Received 1 April 2020; Received in revised form 14 July 2020; Accepted 8 September 2020
Available online 24 September 2020
0887-6185/© 2020 Elsevier Ltd. All rights reserved.

evidence for an "intensity-response relationship" of aerobe exercise in anxiety disorders is currently provided by a meta-analysis that included nine trials focusing on different entities. In this analysis, exercise of higher intensity was found to be superior to "low-intensity exercise" for reducing disorder-specific anxiety with at least a small effect size (Aylett et al., 2018).

Considering these data, we suspected that high-intensity interval training (HIIT) might be a promising tool to further improve the efficacy of AE in anxiety disorders. HIIT is a popular form of exercise that is characterized by short bouts of (sub)maximum physical strain interspersed with brief periods of lower intensity or rest (Laursen & Jenkins, 2002). Although this type of training was originally developed to optimize training programs for endurance athletes, nowadays it is also frequently applied in the general population and was found to be well tolerated by previously sedentary individuals (Reljic et al., 2019). Several clinical trials demonstrated that HIIT led to similar or stronger effects on a range of health-related parameters such as cardiorespiratory fitness (CRF), body mass, glucose utilization or blood pressure compared to a moderate-intensity continuous training. These effects were also already evident within a significantly shorter period of time (Nardi, Tolves, Lenzi, Signori, & Silva, 2018; Way, Sultana, Sabag, Baker, & Johnson, 2019; Wen et al., 2019; Wewege, van den Berg, Ward, & Keech, 2017). With respect to mental conditions, a number of RCT reported HIIT's efficacy and good tolerability particularly in patients with depression. In this context, meta-analytic data also demonstrated that HIIT led to a significantly stronger improvement of symptoms than continuous moderately intense AE (Korman et al., 2019). To get an impression of both the therapeutic properties and the feasibility of HIIT in anxiety disorders, we recently conducted an open trial with patients suffering from PD with and without agoraphobia (AG) (Plag, Ergec, Fydrich, & Ströhle, 2019). As a result, a 12-day HIIT was associated with moderate or large effects on PD severity, agoraphobic avoidance, comorbid depression and endurance performance. Interestingly, no patient dropped out during the study period even though the training was rated as significantly exhausting by the participants (Plag et al., 2019).

The present study therefore aimed to investigate the efficacy and acceptance of HIIT in GAD within a RCT. We decided to focus on GAD because this condition was found to be associated with relatively smaller effects in guideline-based treatments compared to other anxiety disorders (Bandelow et al., 2015; Cuijpers et al., 2016). Since preliminary data also provide some evidence for intensity as an important factor for the impact of exercise on GAD (Herring et al., 2012), we hypothesized that HIIT will cause a faster and larger reduction of disorder-related worrying as well as further illness-related symptoms than a control condition of less intense exercise. Moreover, previous trials found a negative association between symptom severity and CRF in some "stress-related disorders" such as major depressive disorder (MDD) or posttraumatic stress disorder (PTSD) (Papasavvas, Bonow, Alhashemi, & Micklewright, 2016; Whitworth et al., 2020) and other studies nearly unanimously reported a significant improvement of CRF through different forms of HIIT (Wen et al., 2019). We therefore further seeked to assess the relationship between HIIT-induced changes in CRF and worrying as well as disorder-unspecific psychopathology. Therefore, we focused on pre-post effects of VO_{2max} as the most established objective parameter of CRF (Wen et al., 2019). Finally, we also aimed to explore the role of a specific pathogenetic factor of GAD in our intervention. Individual's perceived level of control (PC) over anxiety related stimuli as a distinct cognitive function was shown to be negatively correlated to symptom severity in several anxiety disorders but particularly to worrying in GAD (Gallagher, Bentley, & Barlow, 2014). Moreover, PC was also identified as a relevant factor for recovery in this condition since it was found to be indirectly associated with the outcome of cognitive behavioural therapy (CBT) (Gallagher, Naragon-Gainey, & Brown, 2013). Since further pathogenetic factors of anxiety disorders (e. g. anxiety sensitivity) were demonstrated to be changeable by AE (LeBouthillier & Asmundson, 2015), we therefore further intended to investigate the relevance of PC for both symptom severity and disorder-specific efficacy of AE in the present sample.

2. Method

2.1. Design and patients

The present study is a parallel-group, assessor-blinded RCT and was conducted at the outpatient center for anxiety disorders of the Department of Psychiatry and Psychotherapy of the Charité, Universitätsmedizin Berlin, Germany. We initially planned to assess 40 participants, however, due to reasons of recruitment we were only able to evaluate 33 patients. Participants were told that the goal of the study is to investigate the treatment effects of two different forms of exercise. Therefore, they were not blinded to the intervention but to the hypotheses of the trial. The study was approved by the local ethics committee (registration number: EA1/192/14), registered (Identifier: NCT02662803) and did not receive any specific funding.

Diagnosis of GAD was established according to criteria of the Diagnostic and Statistical Manual of Mental Disorders, 5th revision (DSM-5; (American Psychiatric Association, 2013)) and GAD severity at baseline was measured by the German version of the Penn State Worry Questionnaire (PSWQ; (Meyer, Miller, Metzger, & Borkovec, 1990). Comorbid mental disorders were investigated using the M.I.N.I. for DSM-IV and ICD-10 disorders (Sheehan et al., 1998). Inclusion criteria encompassed a primary diagnosis of GAD, a minimum age of at least 18 years, sufficient German language skills, written informed consent and the ability to attend regularly on the study interventions. Patients were excluded from this trial if they suffered from distinct mental or somatic comorbidities (severe depression, schizophrenia, borderline personality disorder, substance use disorder, suicidality, epilepsy and severe respiratory or cardiovascular diseases) and in case of current psychotherapy. Preexisting psychopharmacotherapy with antidepressants or pregabalin was allowed but the daily dosage had to be stable for at least four weeks before the beginning of the trial and during the whole study period. For additional diagnostics all patients underwent a comprehensive laboratory examination including hepatic, renal and hematological parameters as well as urinary screening including toxicology. Furthermore, CRF was assessed via cardiopulmonary exercise testing (CPET) immediately prior to the study interventions.

2.2. Randomization

Allocation to the intervention or control group was performed by biased coin minimization using the randomization software MinimPy (Saghaei & Saghaei, 2011). Marginal balance was used as a distance measure; the allocation ratio was 1:1. In order to achieve equal distribution of gender, sex and severity of GAD, these variables were integrated into the randomization procedure.

2.3. Determination of the individual training parameters

To determine the optimum training range for HIIT and to rule out any somatic contraindications for physical exercise, each patient underwent a CPET in the Department of Sports Medicine of the Charité, Universitätsmedizin Berlin, Germany. CPET was performed on an upright electronically braked cycle ergometer (Ergoselect 100k, Ergoline GmbH, Bitz, Germany). Breath-by-breath expired gas analysis under continuous 12-lead electrocardiogram monitoring started after a 3 min warm up period with 25 W and was increased by 25 W every 3 min until exhaustion. Subjects were advised to pedal at 70–80 rpm. In the last 15 s of every stage patients had measurement of non-invasive blood pressure as well as capillary blood lactate levels and they had to give their rate of perceived exertion (RPE (Borg, 1998). Measurements were continued during a 5 min recovery period. Maximal heart rate (HR_{max}) and VO_{2max} were determined as the highest average values over 30 s at

the final stage. Individual anaerobic threshold was determined using the first increase of lactate from baseline +1,5 mmol/l (Dickhuth et al., 1991). In order to assess training-related changes in VO_{2max}, all patients performed another CPET immediately after the end of the training period.

2.4. HIIT

According to the recommendations of Gillen and Gibala (Gillen & Gibala, 2014), HIIT was performed every second day within a period of 12 days on a bicycle ergometer. Each session lasted about 20 min and consisted of alternating one-minute bouts of 77–95 % and less than 70 % HR_{max}. The training units were instructed by trained staff and a heart rate monitor (V800 Polar Electro Oy, Finland) was used to ensure that patients exercise within the optimum range. In order to improve the safety of the training, a “warm-up” and “cool-down” period of five minutes was established before and after the training sessions, respectively. Patients were instructed to abstain from any physical exercise from baseline to follow-up in order to estimate the medium-term effects of the intervention.

2.5. Control condition

An aerobic training protocol of lower intensity exercise (LIT) that was already used in former studies served as a control condition (Gaudlitz et al., 2015). The program included separated units of stretching and adapted yoga figures which were performed on a training mat. LIT was matched to HIIT with respect to frequency and duration and therefore six 30-min sessions (including “warm-up” and “cool-down”) were performed within 12 days. The training was also supervised by our team members and a heart rate monitor was used to ensure that patients in the LIT group did not experience high physical strain and were continuously below 70 % HR_{max}. Patients of the control group also did not perform any exercise from post to follow-up.

2.6. Assessment

Assessments were performed before (baseline) and after (post) the training as well as 30 days after baseline (follow-up). Worrying was measured by using the Penn-State-Worry-Questionnaire (PSWQ-D; (Meyer et al., 1990)) that served as the primary outcome. Particularly to investigate changes of GAD symptoms that already occur during the intervention, we additionally applied the Penn-State-Worry-Questionnaire - past week (PSWQ-pw; (Stöber & Bittencourt, 1998)) because the reference period of this questionnaire is the last seven days before measurement. The Hamilton Anxiety Rating Scale (Ham-A; (Hamilton, 1959)) and the Hamilton Depression Rating Scale (Ham-D; (Hamilton, 1960)) were used to assess unspecific anxiety and comorbid depression. We further administered the revised version of the Anxiety Control Questionnaire (ACQ-R; (Brown, White, Forsyth, & Barlow, 2004) to investigate PC in the present sample. Since (stress-related) somatic symptoms are further central clinical characteristics of GAD according to DSM-5, we used the Screening for Somatoform Symptoms-7 (SOMS-7; (Rief & Hiller, 2003) in order to estimate this symptomatology during the last week before measurement. A higher score of the PSWQ-D, PSWQ-pw, Ham-A, Ham-D and SOMS-7 indicates a more pronounced symptomatology whereas the score of the ACQ-R is negatively correlated to the level of PC. The third-party assessment (Ham-A and Ham-D) was performed by J.P. who was blinded to the treatment allocation.

2.7. Data analysis

Including dropouts, the average percentage of missing data on item basis was 10.90 % (maximum: 33.33 %). Missing values were replaced by multiple imputation on scale basis, one of the state-of-the art procedures to handle missing data (Enders, 2010). Baseline data were integrated into all imputation steps. Imputation of post and follow-up data was carried out separately for IG and CG to preserve interaction effects. All analyses were carried out using SPSS v23.0 and reported values are pooled results from a set of five imputations ($n = 33$ if not stated elsewhere). Repeated measures ANOVAs with time (T1, T2) as the within subject factor and group (HIIT, LIT) as the between-subject factor were calculated for all outcome measures, using ηp^2 as the effect size. Post-Hoc tests using Bonferroni correction were calculated for the factor time and Cohen’s d was used as the effect size. To explore potential associations between changes in VO_{2max} as well as ACQ-R and primary/secondary clinical outcomes, Pearsons correlations were used. Significance level was set to .05 (two-tailed).

3. Results

3.1. Sample characteristics

Participants were recruited from 2015 to 2018. Fig. 1 demonstrates the flow of patients. Adherence rate was 82 % for HIIT and 94 % % for the control group indicating no significant differences between the conditions. No patient dropped out for reasons related to exercise. The sample consisted of 24 women and 9 men, with 13 women (72.73 %) in HIIT and 11 women (68.76 %) in LIT. See Table 1 for baseline characteristics.

3.2. Primary clinical outcome

For HIIT and LIT, the PSWQ-D scores at baseline were higher than 60 demonstrating that both groups are considered to be “high worriers” (Behar, Alcaine, Zuellig, & Borkovec, 2003; Meyer et al., 1990). A repeated measurements ANOVA from baseline to post showed a significant effect of time ($F(1,31) = 9.96$, $p < .01$, $\eta p2 = .24$) for PSWQ-D, indicating a reduction in worrying in both groups with a large effect size. There was no significant effect of group ($F(1,31) = 1.75$, $p = .19$). There was a significant time*group interaction ($F(1,31) = 5.90$, $p = .02$, $\eta p2 = .17$), indicating a larger decrease in worrying from baseline to post in HIIT compared to LIT with a large effect size. Post-hoc-tests showed significant reductions of medium effect sizes in symptom severity in HIIT from baseline to post ($p < .01$; $d = .68$) and to follow-up ($p < .01$; $d = .62$) but no significant differences in LIT (baseline to post: $p = .99$; baseline to follow-up: $p = .99$). Fig. 2 shows means for PSWQ-D for both groups and all points of measurement.

3.3. Secondary clinical outcomes

Repeated measurement ANOVAs for Ham-A and Ham-D showed significant group*time interaction effects with large effect sizes indicating a stronger reduction in unspecific anxiety and depression in HIIT compared to LIT (see Table 2). Post-hoc tests showed significant improvements with medium to very large effect sizes in all clinical measures in HIIT from baseline to post that remained stable or improved further to follow-up. For LIT, post-hoc tests showed significant differences with medium to large effects only in Ham-A, Ham-D and SOMS-7 from baseline to post or follow-up, respectively. Generally, effect sizes from baseline to post and follow-up within the groups were about double the size in HIIT compared to LIT. Values are displayed in Table 3.

3.4. ACQ-R and correlation to clinical outcomes

At Baseline, ACQ-R showed significant negative correlations with PSWQ-D (($r = -.74$, $p < .01$) and PSQW-pw ($r = -.59$, $p < .01$) and a significant positive correlation with SOMS-7 ($r = .34$, $p = 05$) but not Ham-A ($r = .00$, $p = .99$) or Ham-D ($r = -.28$, $p = .12$). For the whole sample, changes in ACQ-R from baseline to post showed significant associations with changes in PSWQ-pw ($r = -.57$, $p < .01$), PSWQ-D ($r = -.59$, $p < .01$) and Ham-A ($r = -.38$, $p = .03$) but not Ham-D

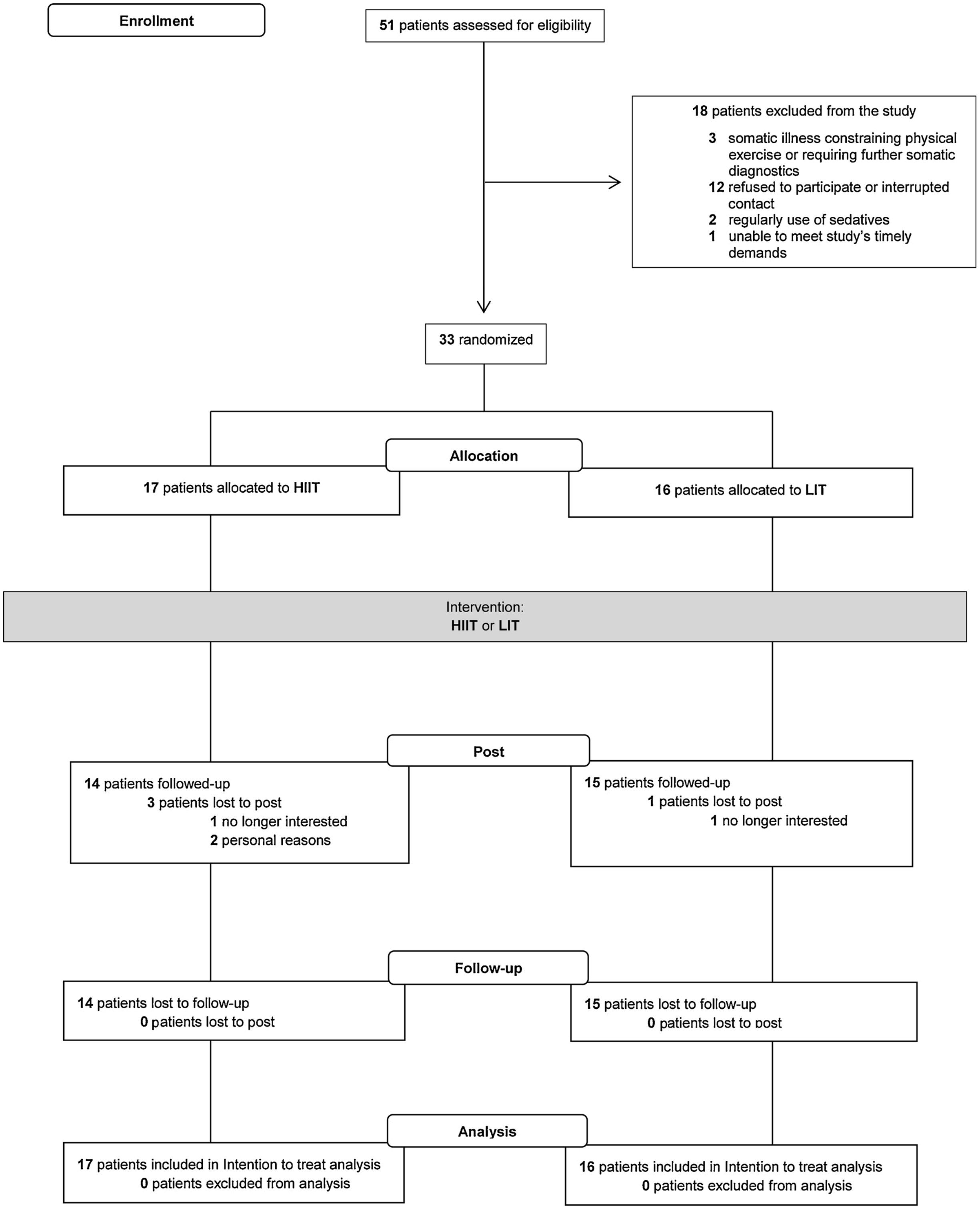

Fig. 1. Participant flow.

($r = -.25$, $p = .17$) or SOMS-7 ($r = -.19$, $p = .31$). If calculated separately for both groups, in HIIT, changes in ACQ from baseline to post showed significant correlations only with PSWQ-pw ($r = -.66$, $p < .01$) and PSWQ-D ($r = -.67$, $p < .01$), while the correlation with Ham-A was no longer significant ($r = -.40$, $p = .13$). In LIT, there were no significant correlations between any of the above measures (PSWQ-pw: $r = -.28$, $p = .30$; PSWQ-D: $r = -.14$, $p = .62$; Ham-A: $r = -.22$, $p = .43$).

3.5. VO_{2max} and correlation to clinical outcomes

Repeated measurement ANVOAS revealed a significant group*time interaction effect with a large effect size for VO_{2max} from baseline to post. Post-hoc tests showed a significant increase with a large effect size in VO_{2max} in HIIT but no significant differences in LIT as showed in Table 2. However, changes in VO_{2max} in HIIT did not significantly correlate with changes in PSWQ-D ($r = .07$, $p = .72$), PSWQ-pw ($r = -.07$, $p = .94$), Ham-A ($r = -.27$, $p = .16$), Ham-D ($r = -.21$, $p = .25$),

Table 1
Baseline characteristics of the sample (n = 33, if not stated elsewhere).

	All		HIIT		LIT	
Parameter	Mean	*SD*	Mean	*SD*	Mean	*SD*
Demographic and physical characteristics						
Age (years)	41.03	12.26	40.18	12.24	41.94	12.62
Height (cm)[a]	170.72	8.40	168.65	8.11	172.92	8.40
Weight (kg)[b]	67.94	14.15	65.98	10.67	70.03	17.27
BMI	23.05	3.29	23.02	2.53	23.08	4.02
Body Fat Content (%)	22.46	4.30	23.21	4.74	21.67	3.78
sBP at rest	121.01	13.10	119.52	9.23	122.58	16.44
dBP at rest[c]	77.50	8.07	78.00	7.27	77.00	9.02
Waist to Hip Ratio[d]	0.83	0.10	0.83	0.09	0.84	0.10
Pulse at rest[e]	73.31	9.47	75.57	8.93	71.20	9.77
Endurance-related variables						
VO_2max	32.20	6.65	33.40	7.14	30.94	6.05
Maximum lactate	7.73	1.88	8.21	1.87	7.22	1.80
performance per kg body weight at the individual anaerobic threshold	1.61	0.39	1.65	0.41	1.56	0.37
Clinical measures						
Ham-A	30.06	7.51	31.29	6.24	28.75	8.67
Ham-D	13.45	5.03	17.71	4.98	13.19	5.23
PSWQ-D	61.30	9.42	60.76	10.82	61.88	7.99
PSWQ-pw	58.76	16.36	56.94	15.53	60.69	17.49
SOMS-7	32.98	21.61	38.35	22.02	27.28	20.30
ACQ-R	67.33	22.18	72.00	19.91	62.38	23.99

sBP = systolic blood pressure; dBP = diastolic blood pressure.
[a] n = 31.
[b] n = 31.
[c] n = 30.
[d] n = 26.
[e] n = 29.

SOMS-7 (r = −.03, p = .87) or ACQ-R (r = .−.18, p = .37) from baseline to post.

4. Discussion

In the first RCT addressing HIIT in anxiety disorders, we were able to demonstrate large treatment effects on worrying, stress-related somatic symptoms, unspecific anxiety and comorbid depression in GAD. As indicated by the PSWQ-pw and SOMS-7 at post as well as at follow-up, its impact on core symptoms of GAD already took place during the intervention and was still detectable 18 days after the end of the active study period. Considering that medication and psychotherapy achieve small and medium to large effects within several weeks or months in this condition (Carl et al., 2019), HIIT showed not only a comparable or even higher disorder-specific efficacy but also a substantially faster onset of action than first-line treatment approaches. Moreover, we were able to confirm the high acceptability of HIIT in our sample which had already been observed in previous trials. Studies focused on either healthy subjects volunteers or patients with distinct somatic and mental illness reported a mean adherence rate of higher than 80 %, respectively (Korman et al., 2019; Martland, Mondelli, Gaughran, & Stubbs, 2019). Importantly, HIIT in the present trial showed a substantially lower cancellation rate compared to a rate of up to 24 %, which is observed for CBT and antidepressants in anxiety disorders in general (Taylor, Abramowitz, & McKay, 2012).

These findings of a high tolerability and substantial effects on disorder-specific anxiety are in line with pre-post data of a current pilot study that addressed the efficacy of HIIT in AG/PD (Plag et al., 2019). In this trial, 12 patients also underwent six sessions of HIIT within 12 days

Table 2
Results from ANOVAs from baseline to post (n = 33) for secondary clinical outcomes/endurance-related variables.

Parameter	ANOVA results[a]	*F*(*df*)	*p*	η_p^2
**Clinical measures**				
Ham-A	Group	0.07 (1,31)	.82	.00
	Time	65.56 (1,31)	**<.01**[**]	**.68**
	Group x time	5.33 (1,31)	**.03**[*]	**.15**
ACQ-R	Group	2.8 (1,31)	.10	.08
	Time	12.35 (1,31)	**<.01**[**]	**.28**
	Group x time	1.07 (1,31)	.31	.03
PSWQ-PW	Group	1.02 (1,31)	.32	.03
	Time	9.12 (1,31)	**<.01**[**]	**.23**
	Group x time	0.56 (1,31)	.47	.02
SOMS-7	Group	1.772 (1,31)	.19	.05
	Time	17.28 (1,31)	**<.01**[**]	**.41**
	Group x time	1.52 (1,31)	.23	.05
Ham-D	Group	1.14 (1, 31)	.30	.035
	Time	49.33 (1,31)	**<.01**[**]	**.61**
	Group x time	5.95 (1,31)	**.02**[*]	**.16**
**Cardiorespiratory fitness**				
VO_{2Max}	Group	5.45 (1,31)	**.028**[*]	**.15**
	Time	13.47 (1,31)	**<.01**[**]	**.30**
	Group x time	6.89 (1,31)	**.02**[*]	**.18**

[*] Significant at .05-level (two-tailed).
[**] Significant at .01-level (two-tailed).
[a] Main effects of time and group and interaction effects group x time of repeated-measurements ANOVAs with time (baseline, post) as within-subject factor and group (HIIT, LIT) as between-subject factor.

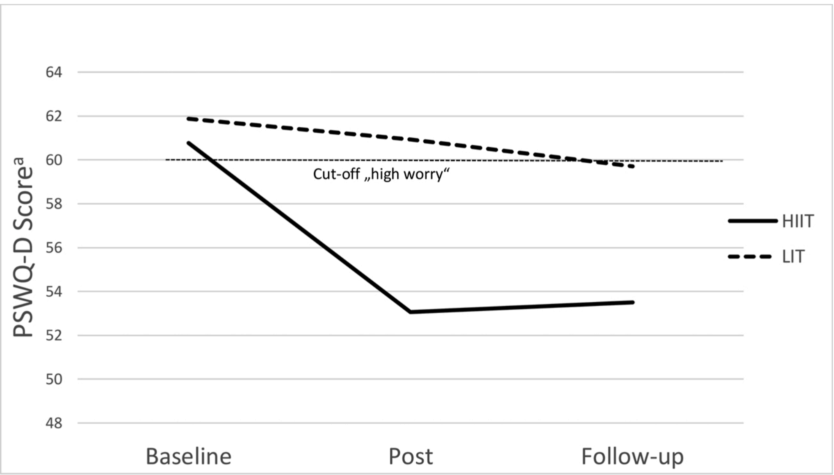

Fig. 2. Means for PSWQ-D for intervention and control group at three points of measurement.

Table 3
Means and post-hoc tests for secondary clinical outcomes/endurance-related variables at all points of measurement (N = 33).

Parameter	HIIT-LIT	Means(M) and 95 %-Confidence intervals at the time points baseline, post and follow-up, results of post hoc comparisons for time[a], and corresponding effect sizes[b]				
		baseline	post		follow-up	
		M (95 %-CI)	M (95 %-CI)	d	M (95 %-CI)	d
**Clinical measures**						
Ham-A	HIIT	31.29 (27.42–34.50)	**17.04**** (12.81–21.81)	1.80	**15.82****(12.01–19.98)	2.1
	LIT	28.75 (24.12–33.37)	**20.83**** (15.50–26.17)	0.85	**18.98**** (13.71–24.26)	1.05
ACQ-R	HIIT	72.00 (59.73–82.24)	**83.38**** (69.17–95.15)	0.53	**83.10*** (67.18–96.85)	0.47
	LIT	62.39 (49.59 – 5.16)	68.60 (57.16–80.04)	0.28	68.42 (56.11 – 8ß.74)	0.24
PSWQ-PW	HIIT	56.94 (49.36–64.92)	**44.50*** (34.31–55.89)	0.65	**38.70****(29.74–49.74)	0.97
	LIT	60.69 (51.37–70.00)	53.15 (40.40–65.90)	0.36	51.11 (39.98–62.23)	0.50
SOMS-7	HIIT	38.35 (25.25–49.67)	**24.04**** (14.26–33.42)	0.71	**19.93**** (12.37–26.65)	1.02
	LIT	27.28 (16.46–38.09)	18.99 (12.03–25.93)	0.48	**16.24**** (8.18–24.30)	0.62
Ham-D	HIIT	13.71 (11.02–16.27)	**5.59**** (4.04–7.60)	1.81	**5.41**** (3.99–7.13)	1.95
	LIT	13.19 (10.40–15.97)	**9.25*** (6.48–12.13)	0.74	**8.14**** (5.55–10.73)	1.01
**Cardiorespiratory fitness**						
VO_{2Max}	HIIT	33.40 (29.40–37.07)	**38.16**** (34.32–41.02)	0.74		
	LIT	32.31 (27.71–34.16)	35.05 (29.38–34.11)	0.15		

[*] Significant at .05-level (two-tailed).
[**] Significant at .01-level (two-tailed).
[a] Asterisks represent significances from post-hoc comparisons for time (baseline to post and baseline to follow-up) using Bonferroni correction; [b]Cohens *d* as effect size for difference between baseline and post or baseline and follow-up within the groups.

in a group setting and instructed by an experienced trainer. After calculating the maximum heart rate by subtracting the age of the patients from 220 (Weineck, 2010), six sessions of ten high-intensity one-minute intervals at 77 %–95 % of HR_{max} were performed every second day without any additional technical equipment. As a result, HIIT led to a significant reduction of PD/AG severity, agoraphobic avoidance and comorbid depression with moderate to strong effects (r = 0.30–0.55) (Plag et al., 2019). Moreover, our finding of a significant effect of HIIT on comorbid depression was also observed in the former study and further supports the results of a recent meta-analysis that reported an overall large effect of HIIT on depression (Martland, Mondelli, Gaughran, & Stubbs, 2019).

Although the impact of HIIT on AG/PD in our former trial already exceeded the overall effects found for several-week moderate intensity exercise training in anxiety disorders (Aylett et al., 2018; Stubbs et al., 2017), the pre-post effect of HIIT on GAD-severity in the present study was even more pronounced. This finding might be explained by several aspects. First, the substantial impact of our intervention on hallmark symptoms of GAD might be related to its effects on distinct pathomechanisms that are particularly important for this condition. In the present sample, baseline values of the ACQ and the PSWQ-D as well as the PWSQ-pw were significantly negatively correlated in the entire sample. This finding is in line with previous research in this field since numerous studies found that PC is a distinct cognitive function with transdiagnostic relevance for the onset as well as the maintenance of anxiety disorders (Gallagher et al., 2014). However, meta-analytic data demonstrated that the association between PC and symptom severity is significantly larger for GAD than for panic disorder or phobic conditions (Gallagher et al., 2014). There is further some evidence that PC may be improved by interventions originally focused on disorder-specific symptoms in different anxiety disorders. Gallagher and coworkers treated about 600 patients that suffered from panic disorder, social phobia or GAD with a several-week CBT. Compared to a waitlist condition, PC significantly increased until the 12-month follow-up with large effects and the change of PC strongly correlated with the improvement of disorder-specific symptoms particularly in GAD (Gallagher et al., 2013). Therefore, our findings of a significant association between the pre-post changes of ACQ and PSWQ-D/PSWQ-pw in the HIIT-group indicate that modification of PC is a common and relevant effect mechanism not only for CBT but also for exercise in (certain) anxiety disorders. The absence of this relationship in the control group, however, is quite important in this context. This observation provides some evidence for the necessity of a certain intensity of aerobic exercise to alter PC (and potentially further pathomechanisms of anxiety disorders) and may explain at least to some extent the "intensity-response" correlation found by former trials in the field of anxiety disorders (Aylett et al., 2018). The higher impact of HIIT on disorder-specific anxiety in the present trial might further be related to different aspects of the study design. In contrast to our pilot trial addressing AG/PD (Plag et al., 2019), HIIT was performed in a standardized one-to-one setting on a bicycle ergometer and the individual training rate was determined using a comprehensive CPET instead of a more simple formula-based calculation. In sum, these measures should be eligible to provide a more personalized training and therefore might further increase the efficacy of HIIT in this sample.

The significant pre-post increase of VO_{2max} in the HIIT group is in line with already existing evidence as the majority of previous trials reported about a HIIT-induced improvement of CRF in healthy subjects or patients with distinct somatic or mental diseases (Martland et al., 2019b; Vancampfort et al., 2017; Wen et al., 2019). Until now, no former trials have addressed the link between changes in VO_{2max} and clinical symptoms in anxiety disorders and we are therefore not able to reflect on our results in this respect. Nevertheless, some previous studies focused on the relationship between CRF and disorder-specific symptoms in other "stress related" mental disorders such as MDD or PTSD. A negative association between symptom severity and CRF was found in several of these trials (Papasavvas et al., 2016; Whitworth et al., 2020), however, the relevance of changes in CRF for the treatment effect of AE is still unclear. To our knowledge, no studies directly addressed this question in MDD until today and RCT that investigated HITT in this condition unanimously did not report a correlation between improvements of symptoms and VO2max (Gerber, Minghetti, Beck, Zahner, & Donath, 2018; Hanssen et al., 2017, 2018; Minghetti et al., 2018). In PTSD, some evidence indicate that lower CRF is associated with a stronger symptom reduction after regular AE in PTSD (LeBouthillier, Fetzner, & Asmundson, 2016) but only one trial directly investigated the interrelationship between CRF and symptomatology so far. More than 100 patients with PTSD underwent eight sessions of physical exercise in addition to a two-week short-term trauma focused therapy. Although CRF and symptom severity were significantly improved at the end of the active study period, no association was found between these parameters (Voorendonk, Sanches, de Jongh, & van Minnen, 2019). In order to get a

better insight into the significance of CRF as an effect factor of AE in mental conditions, future research should specifically address this topic within an appropriate study design.

Finally, it should be noted that LIT in our study also significantly impacted worrying, unspecific anxiety and comorbid depression from baseline to post and follow-up, respectively. These findings agree with results of former studies focused on the effects of AE in patients with depression or GAD (Herring et al., 2012; Schuch et al., 2016). Until today, more than 20 RCT have investigated the effect of different forms of AE on depression and found an overall large effect on symptom severity (Schuch et al., 2016). In line with our findings, however, meta-analytic data demonstrated that HIIT was significantly more effective than moderate intensity AE in this condition (Korman et al., 2019). In contrast, only one trial has already addressed the effect of this procedure in GAD a few years ago. In 2012, the effects of a six-week AE program (leg cycling) or resistance training (pressing, curling and extension of legs) were investigated in 30 female patients (Herring et al., 2012). Two supervised weekly 16-minute sessions of the exercise conditions were performed with a maximum heart rate of around 120 beats per minute in each group. Compared to a waitlist control, both types of exercise led to a significant pre-post reduction of the PSWQ-D with a moderate effect ($p = 0.45$), respectively. Interestingly, a higher rate of remission was observed in the resistance training group which was rated as significantly more exhausting by the patients (Herring et al., 2012). The latter finding underlines our results concerning intensity as a relevant factor particularly for the effect of physical exercise in GAD. However, the control group in the study at hand showed a substantially larger reduction of worrying at the post than both active intervention groups (cycling and resistance training) in the study of Herring and collaborators. Additionally, this larger reduction was achieved within a shorter period of time. This difference might be due to a higher physical strain resulting from the design of our control condition. Instead of exclusively training the lower limbs we combined different exercise elements such as yoga figures and stretching which involve different parts of the body and may therefore be more strenuous than resistance training. This fact may cause a relatively higher efficacy of LIT in our study compared to simple resistance training (Herring et al., 2012) while still remaining inferior to HIIT.

4.1. *Strengths and limitations*

Some limitations have to be considered with respect to the present trial. First, the small sample size may limit the generalizability of our findings. However, we calculated effect sizes for each of our groups to emphasize the relevance of our results. We further argued that not only intensity but also subjective exhaustion may be significantly associated with the efficacy of AE in our sample. Although it seems to be quite likely that more intense or more demanding exercise causes more exhaustion, we were not able to provide immediate evidence for this statement since we did not assess training-related exhaustion in our trial (e.g. by using the Borg Rating of Perceived Exertion (Borg, 1998)). Finally, our findings regarding the sustained effects of HIIT is restricted to a relatively short follow-up period of 18 days and therefore we are not able to estimate the long-term efficacy of this training.

5. Conclusion

The present RCT confirmed the overall role of AE as a treatment option for GAD because we found pronounced, fast-acting and sustained effects on its hallmark symptoms as well as on comorbid depression in both study conditions. For the first time, however, HIIT was demonstrated to be particularly effective and well tolerated in these patients. HIIT therefore holds some potential to complement psychotherapy as well as medication in GAD and may provide the advantage of a relatively fast onset of action. Since there is also preliminary evidence for the impact of HIIT in AG/PD, future RCT may expand the research concerning the disorder-specific efficacy of HIIT on other anxiety disorders such as social anxiety disorder or phobic conditions. As observed for CBT, our results further point to the change of PC as a relevant treatment factor of HIIT in GAD. Upcoming studies may therefore address the link between HIIT-induced treatment effects and further known pathogenetic factors of GAD as well as other anxiety disorders in order to comprehensively elucidate the "intensity-response" relationship of AE in these conditions.

Role of the funding source

This research did not receive any specific grant from funding agencies in the public, commercial, or not-for-profit sectors.

Contributors

Jens Plag and Andreas Ströhle designed the study. Jens Plag wrote the protocol, coordinated the trial, conducted the third-party assessments and wrote the major part of the manuscript. Moritz B. Petzold carried out the main part of the statistical analysis and wrote the result section of the paper. Paul Schmidt-Hellinger and Bernd Wolfarth performed the cardiopulmonary exercise testing. Theresa Klippstein and Jennifer L. Mumm provided support for the preparation and the analysis of the data.

Acknowledgments

The authors would like to thank Nora Jung, Tonja Leonie Kühnel, Jouline Petzka and Monique Strittmatter for the supervision of the exercise units.

References

American Psychiatric Association. (2013). *Diagnostic and statistical manual of mental disorders: Dsm-5* (5th ed.). Washington, DC: American Psychiatric Publishing.

Aylett, E., Small, N., & Bower, P. (2018). Exercise in the treatment of clinical anxiety in general practice—A systematic review and meta-analysis. *BMC Health Services Research, 18*(1), 559. https://doi.org/10.1186/s12913-018-3313-5

Bandelow, B. B., Reitt, M., Röver, C., Michaelis, S., Görlich, Y., & Wedekind, D. (2015). Efficacy of treatments for anxiety disorders: A meta-analysis. *International Clinical Psychopharmacology, 30*(4), 183–192. https://doi.org/10.1097/YIC.0000000000000078

Behar, E., Alcaine, O., Zuellig, A. R., & Borkovec, T. D. (2003). Screening for generalized anxiety disorder using the Penn State Worry Questionnaire: A receiver operating characteristic analysis. *Journal of Behavior Therapy and Experimental Psychiatry, 34* (1), 25–43. https://doi.org/10.1016/s0005-7916(03)00004-1

Bischoff, S., Wieder, G., Einsle, F., Petzold, M. B., Janßen, C., Mumm, J. L. M., & Ströhle, A. (2018). Running for extinction? Aerobic exercise as an augmentation of exposure therapy in panic disorder with agoraphobia. *Journal of Psychiatric Research, 101*, 34–41. https://doi.org/10.1016/j.jpsychires.2018.03.001

Borg, G. (1998). *Borg's perceived exertion and pain scales*. Champaign, IL: Human Kinetics.

Broocks, A., Bandelow, B. B., Pekrun, G., George, A., Meyer, T., Bartmann, U., … Rüther, E. (1998). Comparison of aerobic exercise, clomipramine, and placebo in the treatment of panic disorder. *The American Journal of Psychiatry, 155*(5), 603–609. https://doi.org/10.1176/ajp.155.5.603

Brown, T. A., White, K. S., Forsyth, J. P., & Barlow, D. H. (2004). The structure of perceived emotional control: Psychometric properties of a revised anxiety control questionnaire. *Behavior Therapy, 35*(1), 75–99. https://doi.org/10.1016/S0005-7894(04)80005-4

Carl, E., Witcraft, S. M., Kauffman, B. Y., Gillespie, E. M., Becker, E. S., Cuijpers, P., & Powers, M. B. (2019). Psychological and pharmacological treatments for generalized anxiety disorder (GAD): A meta-analysis of randomized controlled trials. *Cognitive Behaviour Therapy*, 1–21. https://doi.org/10.1080/16506073.2018.1560358

Cuijpers, P., Gentili, C., Banos, R. M., Garcia-Campayo, J., Botella, C., & Cristea, I. A. (2016). Relative effects of cognitive and behavioral therapies on generalized anxiety disorder, social anxiety disorder and panic disorder: A meta-analysis. *Journal of Anxiety Disorders, 43*, 79–89. https://doi.org/10.1016/j.janxdis.2016.09.003

Dickhuth, H.-H., Huonker, M., Münzel, T., Drexler, H., Berg, A., & Keul, J. (1991). Individual anaerobic threshold for evaluation of competitive athletes and patients with left ventricular dysfunction. In N. Bachl, T. E. Graham, & H. Löllgen (Eds.), *Advances in ergometry* (Vol. 60, pp. 173–179). Berlin, Heidelberg: Springer. https://doi.org/10.1007/978-3-642-76442-4_26. Berlin Heidelberg.

Enders, C. K. (2010). *Applied missing data analysis. Methodology in the social sciences*. Retrieved from. New York: Guilford Press http://site.ebrary.com/lib/alltitles/docDetail.action?docID=10389908.

Gallagher, M. W., Bentley, K. H., & Barlow, D. H. (2014). Perceived control and vulnerability to anxiety disorders: A meta-analytic review. *Cognitive Therapy and Research, 38*(6), 571–584. https://doi.org/10.1007/s10608-014-9624-x

Gallagher, M. W., Naragon-Gainey, K., & Brown, T. A. (2013). Perceived control is a transdiagnostic predictor of cognitive-behavior therapy outcome for anxiety disorders. *Cognitive Therapy and Research, 38*(1), 10–22. https://doi.org/10.1007/s10608-013-9587-3

Gaudlitz, K., Plag, J., Dimeo, F., & Ströhle, A. (2015). Aerobic exercise training facilitates the effectiveness of cognitive behavioral therapy in panic disorder. *Depression and Anxiety, 32*(3), 221–228. https://doi.org/10.1002/da.22337

Gerber, M., Minghetti, A., Beck, J., Zahner, L., & Donath, L. (2018). Sprint interval training and continuous aerobic exercise training have similar effects on exercise motivation and affective responses to exercise in patients with major depressive disorders: A randomized controlled trial. *Frontiers in Psychiatry, 9*, 694. https://doi.org/10.3389/fpsyt.2018.00694

Gillen, J. B., & Gibala, M. J. (2014). Is high-intensity interval training a time-efficient exercise strategy to improve health and fitness? *Applied Physiology, Nutrition, and Metabolism = Physiologie Appliquee, Nutrition et Metabolisme, 39*(3), 409–412. https://doi.org/10.1139/apnm-2013-0187

Hamilton, M. (1959). The assessment of anxiety states by rating. *The British Journal of Medical Psychology, 32*(1), 50–55. https://doi.org/10.1111/j.2044-8341.1959.tb00467.x

Hamilton, M. (1960). A rating scale for depression. *Journal of Neurology, Neurosurgery, and Psychiatry, 23*(1), 56–62.

Hanssen, H., Minghetti, A., Faude, O., Schmidt-Trucksäss, A., Zahner, L., Beck, J., … Donath, L. (2017). Effects of endurance exercise modalities on arterial stiffness in patients suffering from unipolar depression: A randomized controlled trial. *Frontiers in Psychiatry, 8*, 311. https://doi.org/10.3389/fpsyt.2017.00311

Hanssen, H., Minghetti, A., Faude, O., Schmidt-Trucksäss, A., Zahner, L., Beck, J., … Donath, L. (2018). Effects of different endurance exercise modalities on retinal vessel diameters in unipolar depression. *Microvascular Research, 120*, 111–116. https://doi.org/10.1016/j.mvr.2018.07.003

Herring, M. P., Jacob, M. L., Suveg, C., Dishman, R. K., & O'Connor, P. J. (2012). Feasibility of exercise training for the short-term treatment of generalized anxiety disorder: A randomized controlled trial. *Psychotherapy and Psychosomatics, 81*(1), 21–28. https://doi.org/10.1159/000327898

Korman, N., Armour, M., Chapman, J., Rosenbaum, S., Kisely, S., Suetani, S., & Siskind, D. (2019). High Intensity Interval training (HIIT) for people with severe mental illness: A systematic review & meta-analysis of intervention studies-considering diverse approaches for mental and physical recovery. *Psychiatry Research*, 112601. https://doi.org/10.1016/j.psychres.2019.112601

Laursen, P. B., & Jenkins, D. G. (2002). The scientific basis for high-intensity interval training: Optimising training programmes and maximising performance in highly trained endurance athletes. *Sports Medicine (Auckland, NZ), 32*(1), 53–73. https://doi.org/10.2165/00007256-200232010-00003

LeBouthillier, D. M., & Asmundson, G. J. G. (2015). A single bout of aerobic exercise reduces anxiety sensitivity but not intolerance of uncertainty or distress tolerance: A randomized controlled trial. *Cognitive Behaviour Therapy, 44*(4), 252–263. https://doi.org/10.1080/16506073.2015.1028094

LeBouthillier, D. M., Fetzner, M. G., & Asmundson, G. J. G. (2016). Lower cardiorespiratory fitness is associated with greater reduction in PTSD symptoms and anxiety sensitivity following aerobic exercise. *Mental Health and Physical Activity, 10*, 33–39. https://doi.org/10.1016/j.mhpa.2015.11.001

Lindenberger, B. L., Plag, J., Schumacher, S., Gaudlitz, K., Bischoff, S., Bobbert, T., & Ströhle, A. (2017). Clinical and neurobiological effects of aerobic exercise in dental phobia: A randomized controlled trial. *Depression and Anxiety, 34*(11), 1040–1048. https://doi.org/10.1002/da.22659

Martland, R., Mondelli, V., Gaughran, F., & Stubbs, B. (2019a). Can high intensity interval training improve health outcomes among people with mental illness? A systematic review and preliminary meta-analysis of intervention studies across a range of mental illnesses. *Journal of Affective Disorders, 263*, 629–660. https://doi.org/10.1016/j.jad.2019.11.039

Martland, R., Mondelli, V., Gaughran, F., & Stubbs, B. (2019b). Can high-intensity interval training improve physical and mental health outcomes? A meta-review of 33 systematic reviews across the lifespan. *Journal of Sports Sciences, 38*(4), 430–469. https://doi.org/10.1080/02640414.2019.1706829

Merom, D., Phongsavan, P., Wagner, R., Chey, T., Marnane, C., Steel, Z., & Bauman, A. (2008). Promoting walking as an adjunct intervention to group cognitive behavioral therapy for anxiety disorders—A pilot group randomized trial. *Journal of Anxiety Disorders, 22*(6), 959–968. https://doi.org/10.1016/j.janxdis.2007.09.010

Meyer, T. J., Miller, M. L., Metzger, R. L., & Borkovec, T. D. (1990). Development and validation of the Penn State Worry Questionnaire. *Behaviour Research and Therapy, 28*(6), 487–495. https://doi.org/10.1016/0005-7967(90)90135-6

Minghetti, A., Faude, O., Hanssen, H., Zahner, L., Gerber, M., & Donath, L. (2018). Sprint interval training (SIT) substantially reduces depressive symptoms in major depressive disorder (MDD): A randomized controlled trial. *Psychiatry Research, 265*, 292–297. https://doi.org/10.1016/j.psychres.2018.04.053

Nardi, A. T., Tolves, T., Lenzi, T. L., Signori, L. U., & Silva, A. M. V.d. (2018). High-intensity interval training versus continuous training on physiological and metabolic variables in prediabetes and type 2 diabetes: A meta-analysis. *Diabetes Research and Clinical Practice, 137*, 149–159. https://doi.org/10.1016/j.diabres.2017.12.017

Papasavvas, T., Bonow, R. O., Alhashemi, M., & Micklewright, D. (2016). Depression symptom severity and cardiorespiratory fitness in healthy and depressed adults: A systematic review and meta-analysis. *Sports Medicine (Auckland, NZ), 46*(2), 219–230. https://doi.org/10.1007/s40279-015-0409-5

Plag, J., Ergec, D.-L., Fydrich, T., & Ströhle, A. (2019). High-intensity interval training in panic disorder patients: A pilot study. *The Journal of Nervous and Mental Disease, 207*(3), 184–187. https://doi.org/10.1097/NMD.0000000000000944

Reljic, D., Lampe, D., Wolf, F., Zopf, Y., Herrmann, H. J., & Fischer, J. (2019). Prevalence and predictors of dropout from high-intensity interval training in sedentary individuals: A meta-analysis. *Scandinavian Journal of Medicine & Science in Sports, 29*(9), 1288–1304. https://doi.org/10.1111/sms.13452

Rief, W., & Hiller, W. (2003). A new approach to the assessment of the treatment effects of somatoform disorders. *Psychosomatics, 44*(6), 492–498. https://doi.org/10.1176/appi.psy.44.6.492

Saghaei, M., & Saghaei, S. (2011). Implementation of an open-source customizable minimization program for allocation of patients to parallel groups in clinical trials. *Journal of Biomedical Science and Engineering, 04*(11), 734–739. https://doi.org/10.4236/jbise.2011.411090

Schuch, F. B., Vancampfort, D., Richards, J., Rosenbaum, S., Ward, P. B., & Stubbs, B. (2016). Exercise as a treatment for depression: A meta-analysis adjusting for publication bias. *Journal of Psychiatric Research, 77*, 42–51. https://doi.org/10.1016/j.jpsychires.2016.02.023

Sheehan, D. V., Lecrubier, Y., Sheehan, K. H., Amorim, P., Janavs, J., Weiller, E., & Dunbar, G. C. (1998). The Mini-International Neuropsychiatric Interview (M.I.N.I.): The development and validation of a structured diagnostic psychiatric interview for DSM-IV and ICD-10. *The Journal of Clinical Psychiatry, 59*(Suppl 20), 22–33. quiz 34-57.

Stöber, J., & Bittencourt, J. (1998). Weekly assessment of worry: An adaptation of the Penn State Worry Questionnaire for monitoring changes during treatment. *Behaviour Research and Therapy, 36*(6), 645–656. https://doi.org/10.1016/s0005-7967(98)00031-x

Stubbs, B., Vancampfort, D., Rosenbaum, S., Firth, J., Cosco, T., Veronese, N., & Schuch, F. B. (2017). An examination of the anxiolytic effects of exercise for people with anxiety and stress-related disorders: A meta-analysis. *Psychiatry Research, 249*, 102–108. https://doi.org/10.1016/j.psychres.2016.12.020

Taylor, S., Abramowitz, J. S., & McKay, D. (2012). Non-adherence and non-response in the treatment of anxiety disorders. *Journal of Anxiety Disorders, 26*(5), 583–589. https://doi.org/10.1016/j.janxdis.2012.02.010

Vancampfort, D., Rosenbaum, S., Schuch, F., Ward, P. B., Richards, J., Mugisha, J., … Stubbs, B. (2017). Cardiorespiratory fitness in severe mental illness: A systematic review and meta-analysis. *Sports Medicine (Auckland, NZ), 47*(2), 343–352. https://doi.org/10.1007/s40279-016-0574-1

Voorendonk, E. M., Sanches, S. A., de Jongh, A., & van Minnen, A. (2019). Improvements in cardiorespiratory fitness are not significantly associated with post-traumatic stress disorder symptom reduction in intensive treatment. *European Journal of Psychotraumatology, 10*(1), 1654783. https://doi.org/10.1080/20008198.2019.1654783

Way, K. L., Sultana, R. N., Sabag, A., Baker, M. K., & Johnson, N. A. (2019). The effect of high intensity interval training versus moderate intensity continuous training on arterial stiffness and 24h blood pressure responses: A systematic review and meta-analysis. *Journal of Science and Medicine in Sport, 22*(4), 385–391. https://doi.org/10.1016/j.jsams.2018.09.228

Weineck, J. (2010). *Optimales Training: Leistungsphysiologische Trainingslehre unter besonderer Berücksichtigung des Kinder- und Jugendtrainings (16., durchgesehene Auflage)*. Retrieved from. Balingen: Spitta http://medizin.spitta.de/Sport/145_index.html.

Wen, D., Utesch, T., Wu, J., Robertson, S., Liu, J., Hu, G., … Chen, H. (2019). Effects of different protocols of high intensity interval training for VO2max improvements in adults: A meta-analysis of randomised controlled trials. *Journal of Science and Medicine in Sport, 22*(8), 941–947. https://doi.org/10.1016/j.jsams.2019.01.013

Wewege, M., van den Berg, R., Ward, R. E., & Keech, A. (2017). The effects of high-intensity interval training vs. moderate-intensity continuous training on body composition in overweight and obese adults: A systematic review and meta-analysis. *Obesity Reviews, 18*(6), 635–646. https://doi.org/10.1111/obr.12532

Whitworth, J. W., Hayes, S. M., Andrews, R. J., Fonda, J. R., Beck, B. M., Hanlon, L. B., & McGlinchey, R. E. (2020). Cardiorespiratory fitness is associated with better cardiometabolic health and lower PTSD severity in Post-9/11 veterans. *Military Medicine, 185*(5–6), e592–e596. https://doi.org/10.1093/milmed/usaa005
