# Supplementary material for: Does physical activity-based intervention decrease repetitive negative thinking? A systematic review
Source: PLoS One. 2025 Apr 1;20(4):e0319806. doi: 10.1371/journal.pone.0319806 (PMC11960971; doi:10.1371/journal.pone.0319806)
Supplement: S1 File — https://doi.org/10.6084/m9.figshare.25711734. (ZIP) [file pone.0319806.s001.zip › supporting information/paper file/McDowell 2016.pdf]

Medicine & Science in Sports & Exercise

The Official Journal of the American College of Sports Medicine
www.acsm-msse.org

. . . Published ahead of Print

Sex-Related Differences in Mood Responses to Acute Aerobic Exercise

Cillian P. McDowell,[1] Mark J. Campbell,[1] and Matthew P. Herring[1,2]

[1]Department of Physical Education and Sport Sciences, University of Limerick, Ireland
[2]Health Research Institute, University of Limerick, Ireland

ACCEPTED

Accepted for Publication: 20 April 2016

***Medicine & Science in Sports & Exercise*® Published ahead of Print** contains articles in unedited manuscript form that have been peer reviewed and accepted for publication. This manuscript will undergo copyediting, page composition, and review of the resulting proof before it is published in its final form. Please note that during the production process errors may be discovered that could affect the content.

Copyright © 2016 American College of Sports Medicine

Medicine & Science in Sports & Exercise, Publish Ahead of Print
DOI: 10.1249/MSS.0000000000000969

Sex-Related Differences in Mood Responses to Acute Aerobic Exercise

Cillian P. McDowell,[1] Mark J. Campbell,[1] and Matthew P. Herring[1,2]

[1]Department of Physical Education and Sport Sciences, University of Limerick, Ireland

[2]Health Research Institute, University of Limerick, Ireland

Corresponding Author:

Matthew P. Herring, PhD

PESS 1045

Department of Physical Education and Sport Sciences

University of Limerick

Limerick, Ireland

Phone: +353 061 23 4762

Email: matthew.herring@ul.ie

The authors disclose no conflicts of interest. The results of the present study do not constitute endorsement by the American College of Sports Medicine. This study is unfunded.

Running title: Sex-Related Differences, Mood, and Exercise

ACCEPTED

Copyright © 2016 by the American College of Sports Medicine. Unauthorized reproduction of this article is prohibited.

Abstract

Though some evidence supports stronger mood improvements in response to acute exercise among women, sex-related differences remain understudied. **Purpose:** To quantify and compare differences in baseline mood and the magnitude of mood responses to either acute aerobic exercise or quiet rest among young adult men and women. **Methods:** Fifty-three (27 M; 26 F) young adults completed two counterbalanced conditions: 30-min of vigorous treadmill exercise or 30-min of quiet rest. Outcomes included state anxiety, worry symptoms, and feelings of tension, depression, vigor, fatigue, anger, and confusion. ANOVA and RM-ANOVA examined sex-related differences at baseline and across condition and time, respectively. Hedges' *d* (95%Cis) were calculated to quantify and compare the magnitude of change in response to exercise compared to control. **Results:** Females were more likely to report scores indicative of depression (QIDS>5; 38.5% vs. 18.5%) and high trait anxiety (≥1SD above age- and sex-related norm on STAI-Y2; 26.9% vs. 3.7%). Baseline worry symptoms and trait anxiety were significantly higher among females ($p<0.02$). Though repeated measures models did not support statistically significant differences between sexes, the magnitude of improvement in mood outcomes was larger among females than males for all outcomes other than feelings of tension. Compared to quiet rest, exercise significantly improved feelings of fatigue (*d*=0.59 [0.01, 1.17]), confusion (*d*=0.83 [0.24, 1.41]), and energy (*d*=1.67 [1.02, 2.33]), and total mood disturbance (*d*=1.09 [0.49, 1.70]), and resulted in a non-significant moderate-sized improvement in state anxiety (*d*=0.51 [-0.07, 1.08]) among females. **Conclusion:** Findings support potential sex-related differences in mood response to acute aerobic exercise, with larger improvements found among females. Future research should confirm findings and examine putative mechanisms of sex-related differences in mood responses to exercise. **Keywords:** Acute exercise; mood states; energy; fatigue; state anxiety; sex-related differences

ACCEPTED

Copyright © 2016 by the American College of Sports Medicine. Unauthorized reproduction of this article is prohibited.

Introduction

The positive effects of an acute bout of exercise on mood states are well-documented. Quantitative syntheses of the available evidence support reduced state anxiety (10), enhanced cognitive function (7) and sleep (39), and improved feelings of energy and fatigue in response to acute exercise (21). Though less well-documented, acute exercise also has been shown to improve anger (36), depressive mood state (1), pain perception (15), and panic in both patients with panic disorder and healthy controls (35).

Several factors are proposed to influence the effects of exercise on mood, including participant characteristics, features of the exercise stimulus, and neurobiological and psychological factors. Rodent models and human studies have supported favourable biological adaptations, including alterations in 5-HT, norepinephrine (NE), galanin, and brain-derived neurotrophic factor (BDNF), following acute and chronic PA (11, 17, 18, 26). Moreover, improved mood states have been reported in response to both acute aerobic (4) and resistance (27) exercise, among healthy (16) and chronically-ill samples, and in response to varying intensities (10, 29).

Potential sex-related differences in mood response to acute exercise have remained relatively understudied. Some evidence supports stronger effects of exercise on mood among women compared to men, findings which some authors have postulated may arise from poorer baseline mood profiles and larger subsequent benefits in response to exercise, and/or greater motivation for exercise as a strategy to enhance mood among women (30, 38). However, overall findings regarding sex-related differences in mood response have been mixed and the majority of

ACCEPTED

Copyright © 2016 by the American College of Sports Medicine. Unauthorized reproduction of this article is prohibited.

studies have either not examined or not reported potential sex-related differences. Thus, the present study examined differences in baseline mood and responses to 30-min of vigorous intensity aerobic exercise between young adult men and women.

Methods

Design & Participants. A within-subjects, repeated measures design was used, and the study protocol was approved by the University's Research Ethics Board. Based on *a priori* power analysis performed with G*Power, a sample size of 52 would provide >80% statistical power to detect differences in mood assuming a two-tailed α=0.05, a conservative correlation between repeated measures (r=0.5), and a moderate-sized effect of acute exercise on mood (f=0.2) based on previous evidence (10, 13, 21). Thus, 53 young adult men and women, aged 21.2±1.5y, were recruited from the university and surrounding populations. Prior to participation, interested potential participants provided written informed consent and completed a medical history screening questionnaire. Potential participants, aged 18-35y, who were free of any medical contraindication to safe participation in vigorous aerobic exercise and who were not pregnant or lactating were randomized to one of two counterbalanced conditions: an acute 30-min bout of vigorous treadmill running or 30-min of seated quiet rest.

Baseline Measures. Prior to the beginning of testing on day 1, each participant completed electronically administered measures of self-reported physical activity (Seven Day Physical Activity Recall) (3), trait anxiety (trait subscale of the State-Trait Anxiety Inventory, STAI-Y2) (33), depressive symptoms (Quick Inventory of Depressive Symptoms, QIDS) (31), and sleep quality (Pittsburgh Sleep Quality Index, PSQI) (6).

ACCEPTED

Copyright © 2016 by the American College of Sports Medicine. Unauthorized reproduction of this article is prohibited.

Outcome Measures. Immediately before and 10 minutes following each condition, participants completed electronically-administered measures of state anxiety (state subscale of the State-Trait Anxiety Inventory, STAI-Y1) (33), worry symptoms (Penn State Worry Questionnaire, PSWQ) (24), and the intensity of feelings of tension, depression, vigor, fatigue, anger, and confusion (Profile of Mood States – Brief Form, POMS-B) (22). Participants were instructed to respond to the POMS-B "based on how you feel RIGHT NOW."

Conditions

Acute Exercise. Participants completed a 30-min supervised bout of treadmill running at ≥65% of maximal heart rate reserve (%HRR) calculated using the normative values for resting HR for this population age-group (i.e., 63 for males and 73 for females). Heart rate was monitored continuously using Polar HR monitors. Each participant was instructed to perform a 5-min warm-up, progressively increasing the treadmill speed and/or grade to achieve 65%HRR, and to then complete 30 min at ≥65%HRR. When 30 min at or above 65%HRR had been completed, each participant was asked to walk until he/she felt sufficiently cooled down. Each participant then provided a session RPE using Borg's 6-20 RPE scale. Standard instructions for rating perceived exertion were provided to each participant. Given the potential for mood effects of social interaction (23), extraneous conversation was minimized during the exercise session.

Control: Seated Quiet Rest. During the control condition, each participant completed a 30-min bout of seated quiet rest, a widely used and well-established control condition in studies of acute exercise and mood. For example, in a recent meta-analytic update of randomized controlled trials of acute exercise effects on state anxiety published over the past 25 years, 23 of the 36 included

ACCEPTED

Copyright © 2016 by the American College of Sports Medicine. Unauthorized reproduction of this article is prohibited.

studies (~64%) compared acute exercise to quiet rest (10). Procedures were consistent with the exercise session except that each participant sat in an upright chair in a quiet area for 30 min. Consistent with the exercise session, extraneous conversation with each participant was limited during the quiet rest session. Participants were not allowed to read, study, or listen to music given the potential for altered mood state responses.

Analyses. Data analyses were performed using SPSS 22.0. One-way ANOVA examined baseline (Day 1) sex-related differences in demographic variables, trait anxiety, depression symptoms, physical activity level, sleep quality, and outcome measures. Chi-square tests examined sex-related differences in smoking status, high trait anxiety (>1SD above the age-related norm), and poor sleep (PSQI>5).

ANOVA examined sex-related differences in total exercise time and average session HR between males and females. For males and females, Pearson correlation coefficients quantified associations between total exercise time and average session HR and percent change in mood outcomes. Linear regression examined associations between baseline physical activity level and mood responses to acute exercise.

Repeated measures ANOVA examined differences between exercise and the quiet rest control and potential sex-related differences. However, given increased calls across the psychological literature to move beyond acknowledged flaws of null-hypothesis significance testing in favor of estimation based on effect sizes and confidence intervals (9), we also sought to quantify and compare the magnitude of change in mood responses among men and women using

ACCEPTED

Copyright © 2016 by the American College of Sports Medicine. Unauthorized reproduction of this article is prohibited.

Hedges' *d* effect sizes and associated 95% confidence intervals (95%CI). For each outcome measure, the mean pre-condition to post-condition change for the quiet rest condition was subtracted from the mean pre-condition to post-condition change for the exercise condition and divided by the pooled pre-condition standard deviation (12). Effect sizes were adjusted for small sample bias and calculated such that improved moods resulted in positive effect sizes (12).

Results

Table 1 shows baseline participant characteristics. Baseline ANOVAs revealed sex-related differences in age ($F_{(1,49)}$=7.22, $p \leq 0.01$), weight ($F_{(1,47)}$=52.77, $p \leq 0.001$), BMI ($F_{(1,45)}$=4.45, $p \leq 0.05$, trait anxiety ($F_{(1,46)}$=6.68, $p \leq 0.05$), and worry symptoms ($F_{(1,46)}$=12.51, $p \leq 0.001$). There was no significant difference in self-reported physical activity ($F_{(1,49)}$=0.01, $p>0.92$). Women, however, were significantly more likely to report high trait anxiety ($X^2_{(53)}$=5.57, $p \leq 0.02$) and poor sleep ($X^2_{(52)}$=4.06, $p \leq 0.05$).

Participants averaged 34.9±6.2 min of total exercise at an average HR of 162.2±5.4 bpm. Total exercise time did not significantly differ between males and females ($p>0.60$). However, session average HR was significantly higher among females (164.2±4.9) compared to males (160.2±5.1; $F_{(1,45)}$=7.40, $p \leq 0.009$). Session ratings of perceived exertion ranged from 9 to 17 (13±2), and did not significantly differ between males and females ($F_{(1,38)}$=1.00, $p>0.32$). Among males, total exercise time was significantly associated with percent change in worry symptoms (r=-0.58, $p \leq 0.005$) and anger (r=-0.92, $p \leq 0.001$); no significant associations were found among women. Baseline physical activity level was not significantly associated with change in any outcome (all $p>0.08$).

ACCEPTED

Copyright © 2016 by the American College of Sports Medicine. Unauthorized reproduction of this article is prohibited.

A statistically significant sex by condition by time interaction was found for feelings of energy ($F_{(1,86)}$ =6.77, $p \leq 0.011$). Simple effects analyses were used to decompose this interaction. Compared to females, feelings of energy were significantly higher among males at pre-exercise (mean difference: 2.81, $p \leq 0.028$) and non-significantly higher among males at post-control (mean difference: 2.32, $p \leq 0.057$). Feelings of energy were significantly higher at post-exercise than post-control among both males (mean difference: 3.36, $p \leq 0.007$) and females (mean difference: 4.39, $p \leq 0.001$). Sex by condition by time interactions were not statistically significant for other mood outcomes (all $p > 0.07$). Compared to control, acute aerobic exercise significantly improved state anxiety ($F_{(1,92)}$ =12.52, $p \leq 0.001$), feelings of depression ($F_{(1,86)}$ =5.05, $p \leq 0.027$), fatigue ($F_{(1,86)}$ =15.39, $p \leq 0.001$), and confusion ($F_{(1,86)}$=10.58, $p \leq 0.002$, and total mood disturbance $F_{(1,86)}$ =36.91, $p \leq 0.001$). Statistically non-significant improvements were found for worry ($p > 0.07$) and feelings of tension ($p > 0.19$) and anger ($p > 0.05$).

Pre- and post-condition means (SD) and Hedges' *d* effect sizes (95%CI) are presented in Table 2. Among females, compared to quiet rest, acute exercise significantly decreased feelings of fatigue (*d*=0.59; 95%CI: [0.01-1.17]), confusion (*d*=0.83; [0.24-1.41]), and total mood disturbance (*d*=1.09; [0.49-1.70]), and significantly increased feelings of energy (*d*=1.67; [1.02-2.33]). Non-significant moderate-sized improvements were found for state anxiety (*d*=0.51; [-0.07-1.08]). No statistically significant effects were found for males. However, non-significant moderate-sized improvements were found for feelings of confusion (*d*=0.42), total mood disturbance (*d*=0.47), and feelings of energy (*d*=0.50). The magnitude of improvement in response to acute exercise was larger (i.e., effect sizes were greater) among women than men for all outcomes other than feelings of tension.

Copyright © 2016 by the American College of Sports Medicine. Unauthorized reproduction of this article is prohibited.

Discussion

The present findings supported the positive effect of exercise on mood and provide support for sex-related differences in mood responses to an acute bout of vigorous intensity aerobic exercise. Compared to a quiet rest control, exercise significantly improved state anxiety, feelings of depression and fatigue, and total mood disturbance, and non-significantly improved worry symptoms and feelings of tension and anger in the full combined sample of men and women. Though a statistically significant sex by condition by time interaction was only found for feelings of energy, the magnitude of improvement in mood response to acute exercise compared to quiet rest, quantified by Hedges' *d* effect sizes, was significantly larger among women than among men for all mood outcomes other than feelings of tension.

To our knowledge this is the first methodologically rigorous, counterbalanced, controlled investigation to show larger mood improvements among women in response to vigorous intensity aerobic exercise. Though overall findings regarding sex-related differences in mood response have been mixed and the majority of studies have either not examined or not reported potential sex-related differences, the current findings support a limited amount of previous evidence (30, 38). For example, a previous study reported lower negative mood among women following an acute bout of resistance exercise (30). However, the previous study lacked a control condition and did not standardize exercise intensity, limitations which were addressed by the design of the study reported here.

The magnitude of the current improvements in mood among both males and females are comparable to previously reported evidence. Improvements in feelings of energy and fatigue are

ACCEPTED

Copyright © 2016 by the American College of Sports Medicine. Unauthorized reproduction of this article is prohibited.

comparable to, if not larger than, previously reported effects during and following acute resistance exercise (d=-0.09 to -0.63 and d=0.05 to 0.73 for fatigue and energy, respectively) (14), and the mean effect from 16 trials of acute exercise (Δ=0.03 and Δ=0.47 for fatigue and energy, respectively) (21). Small-to-moderate improvements in state anxiety are comparable to the recently reported mean effect of g=0.16 from 36 trials published since 1990 (10). Importantly, the present findings provide the first evidence of the effects of acute aerobic exercise on worry symptoms. The small improvement in worry symptoms among women (d=0.25) is comparable to the previously reported effect of four bouts (findings from week two of six-week trial) of moderate-intensity cycling exercise among young women with Generalized Anxiety Disorder (d=0.23) (13). These novel findings warrant further investigation.

Women in the current study reported greater improvement in all mood outcomes other than feelings of tension. The magnitude of the differences in mood between sexes are similar to those reported in previous longitudinal studies (2, 19, 25, 28). However, women still reported worse mood than men following exercise, as evidenced by higher reported state anxiety, feelings of tension, fatigue, confusion, and total mood disturbance and lower feelings of energy. This is plausibly due to their somewhat poorer baseline mood profile. Specifically, women reported significantly higher baseline trait anxiety and worry scores than men, and marginally higher depressive symptoms, state anxiety, total mood disturbance and feelings of tension and lower feelings of energy. Previous evidence suggests that individuals with worse mood prior to exercise generally derive the most benefit (29). Thus, it is plausible that larger improvements in mood observed in women were due to their somewhat higher negative mood at baseline. However, given that effect size calculations consider change from baseline for exercise and

ACCEPTED

Copyright © 2016 by the American College of Sports Medicine. Unauthorized reproduction of this article is prohibited.

control as well as the pooled baseline standard deviation, equally plausible explanations include a potential floor effect among males and a slight worsening of some mood outcomes following quiet rest among females.

Though not measured in the current study, it is plausible that sex-related differences in neurobiological responses to exercise, including exercise-induced changes in the synthesis, metabolism, and cellular transit of 5-HT, NE, galanin, and/or BDNF, could underlie greater mood enhancing effects of exercise in women. Indeed, Schuch and colleagues (32) reported that serum BDNF significantly increased only in women with bipolar disorder in response to a single session of maximal aerobic exercise; no changes were observed in men with bipolar disorder. Psychological factors, including anxiety sensitivity (20), distraction (5), and changes in attentional resources (37), may also influence exercise-induced changes in mood. Sex-related differences have also been suggested for factors such as anxiety sensitivity (34), but how potential sex-related differences in these psychological factors may influence mood responses to exercise have remained understudied and warrant further investigation.

Potential limitations include small sample size, the timing of mood measurements, and a focus on young adults recruited from the university and surrounding communities. Mood was measured 10-min post-conditions in the current study. However, some previous evidence has suggested that mood may improve to a larger magnitude 20-30 minutes following acute exercise (8, 14). It is plausible that greater effects would have been observed had there been a greater delay between completion of each condition and measurement of mood.

ACCEPTED

Copyright © 2016 by the American College of Sports Medicine. Unauthorized reproduction of this article is prohibited.

Conclusions. The present findings supported the positive effect of exercise on mood, and provided support for sex-related differences in mood responses to acute aerobic exercise, such that larger magnitude improvements were found among young women. Future research with larger sample sizes and varying exercise intensities and modes is warranted. For example, future research should examine the plausible mechanisms that may underlie sex-related differences in the exercise-mood relationship. Psychophysiological methods such as EEG and eye-tracking paradigms may offer more objective and precise measurements of plausible markers of sex-related differences in mood responses to acute exercise.

ACCEPTED

Copyright © 2016 by the American College of Sports Medicine. Unauthorized reproduction of this article is prohibited.

Acknowledgements

The authors wish to thank Rachel Geil for her contributions to the processing and management of data included in this manuscript. This study is unfunded.

Conflict of Interest

The authors disclose no conflicts of interest. The results of the present study do not constitute endorsement by the American College of Sports Medicine.

ACCEPTED

Copyright © 2016 by the American College of Sports Medicine. Unauthorized reproduction of this article is prohibited.

References

1. Bartholomew JB, Morrison D, Ciccolo JT. Effects of acute exercise on mood and well-being in patients with major depressive disorder. *Med Sci Sports Exerc*. 2005;37(12):2032-7.

2. Birkeland MS, Torsheim T, Wold B. A longitudinal study of the relationship between leisure-time physical activity and depressed mood among adolescents. *Psychol Sport Exerc*. 2009;10(1):25-34.

3. Blair SN, Haskell WL, Ho P, et al. Assessment of habitual physical activity by a sevenday recall in a community survey and controlled experiments. *Am J Epidemiol*. 1985;122(5):794-804.

4. Blake H, Mo P, Malik S, Thomas S. How effective are physical activity interventions for alleviating depressive symptoms in older people? A systematic review. *Clin Rehabil*. 2009;23(10):873-87.

5. Breus MJ, O'Connor PJ. Exercise-induced anxiolysis: a test of the" time out" hypothesis in high anxious females. *Med Sci Sport Exerc*. 1998;30(7):1107-12.

6. Buysse DJ, Reynolds CF, Monk TH, Berman SR, Kupfer DJ. The Pittsburgh Sleep Quality Index: A new instrument for psychiatric practice and research. *Psychiatry Res*. 1989;28(2):193-213.

7. Chang Y-K, Labban J, Gapin J, Etnier J. The effects of acute exercise on cognitive performance: A meta-analysis. *Brain Res*. 2012;1453:87-101.

8. Cox RH, Thomas TR, Hinton PS, Donahue OM. Effects of acute 60 and 80% VO2max bouts of aerobic exercise on state anxiety of women of different age groups across time. *Res Q Exerc Sport*. 2004;75(2):165-75.

9. Cumming G. The new statistics: Why and how. *Psychol Sci*. 2014;25(1):7-29.

ACCEPTED

Copyright © 2016 by the American College of Sports Medicine. Unauthorized reproduction of this article is prohibited.

10. Ensari I, Greenlee TA, Motl RW, Petruzzello SJ. Meta-analysis of acute exercise effects on state anxiety: An update of randomized controlled trials over the past 25 years. *Depress Anxiety*. 2015;32:624-34.

11. Greenwood BN, Fleshner M. Exercise, stress resistance, and central serotonergic systems. *Exerc Sport Sci Rev*. 2011;39(3):140-9.

12. Hedges LV, Olkin I. *Statistical methods for meta-analysis*. New York: Academic Press; 1985.

13. Herring MP, Jacob ML, Suveg C, Dishman RK, O'Connor PJ. Feasibility of exercise training for the short-term treatment of generalized anxiety disorder: A randomized controlled trial. *Psychother Psychosom*. 2012;81(1):21-8.

14. Herring MP, O'Connor PJ. The effect of acute resistance exercise on feelings of energy and fatigue. *J Sports Sci*. 2009;27(7):701-9.

15. Hoffman MD, Hoffman DR. Does aerobic exercise improve pain perception and mood? A review of the evidence related to healthy and chronic pain subjects. *Curr Pain Headache Rep*. 2007;11(2):93-7.

16. Hoffman MD, Hoffman DR. Exercisers achieve greater acute exercise-induced mood enhancement than nonexercisers. *Archives of physical medicine and rehabilitation*. 2008;89(2):358-63.

17. Holmes PV, Yoo HS, Dishman RK. Voluntary exercise and clomipramine treatment elevate prepro-galanin mRNA levels in the locus coeruleus in rats. *Neurosci Lett*. 2006;408(1):1-4.

ACCEPTED

Copyright © 2016 by the American College of Sports Medicine. Unauthorized reproduction of this article is prohibited.

18. Hopkins ME, Bucci DJ. BDNF expression in perirhinal cortex is associated with exercise-induced improvement in object recognition memory. *Neurobiol Learn Mem*. 2010;94(2):278-84.

19. Kearney PM, Cronin H, O'Regan C, et al. Cohort profile: The Irish longitudinal study on ageing. *Int J Epidemiol*. 2011;40(4):877-84.

20. LeBouthillier DM, Asmundson GJ. A single bout of aerobic exercise reduces anxiety sensitivity but not intolerance of uncertainty or distress tolerance: A randomized controlled trial. *Cogn Behav Ther*. 2015;44(4):252-63.

21. Loy BD, O'Connor PJ, Dishman RK. The effect of a single bout of exercise on energy and fatigue states: A systematic review and meta-analysis. *Fatigue*. 2013;1(4):223-42.

22. McNair DM, Droppleman LF, Lorr M. *Edits manual for the profile of mood states: POMS*. Edits; 1992.

23. McNeil JK, LeBlanc EM, Joyner M. The effect of exercise on depressive symptoms in the moderately depressed elderly. *Psychol Aging*. 1991;6(3):487.

24. Meyer TJ, Miller ML, Metzger RL, Borkovec TD. Development and validation of the penn state worry questionnaire. *Behav Res Ther*. 1990;28(6):487-95.

25. Monin JK, Levy B, Chen B, et al. Husbands' and wives' physical activity and depressive symptoms: Longitudinal findings from the Cardiovascular Health Study. *Ann Behav Med*. 2015;49(5):704-14.

26. Morgan JA, Corrigan F, Baune BT. Effects of physical exercise on central nervous system functions: a review of brain region specific adaptations. *J Mol Psychiatry*. 2015;3(3). Available from: http://jmolecularpsychiatry.biomedcentral.com/articles/10.1186/s40303-015-0010-8. doi: 10.1186/s40303-015-0010-8.

ACCEPTED

Copyright © 2016 by the American College of Sports Medicine. Unauthorized reproduction of this article is prohibited.

27. O'Connor PJ, Herring MP, Caravalho A. Mental health benefits of strength training in adults. *Am J Lifestyle Med*. 2010;4(5):377-96.

28. Pereira SMP, Geoffroy M-C, Power C. Depressive symptoms and physical activity during 3 decades in adult life: bidirectional associations in a prospective cohort study. *JAMA Psychiatry*. 2014;71(12):1373-80.

29. Reed J, Ones DS. The effect of acute aerobic exercise on positive activated affect: A meta-analysis. *Psychol Sport Exerc*. 2006;7(5):477-514.

30. Rocheleau CA, Webster GD, Bryan A, Frazier J. Moderators of the relationship between exercise and mood changes: Gender, exertion level, and workout duration. *Psychol Health*. 2004;19(4):491-506.

31. Rush AJ, Trivedi MH, Ibrahim HM et al. The 16-Item Quick Inventory of Depressive Symptomatology (QIDS), clinician rating (QIDS-C), and self-report (QIDS-SR): A psychometric evaluation in patients with chronic major depression. *Biol Psychiatry*. 2003;54(5):573-83.

32. Schuch FB, da Silveira LE, de Zeni TC et al. Effects of a single bout of maximal aerobic exercise on BDNF in bipolar disorder: A gender-based response. *Psychiatry Res*. 2015;229(1):57-62.

33. Spielberger CD, et al. *Manual for the State-Trait Anxiety Inventory*. Palo Alto, CA: Consulting Psychologists Press, Inc., 1983, pp. 5.

34. Stewart SH, Taylor S, Baker JM. Gender differences in dimensions of anxiety sensitivity. *J Anxiety Disord*. 1997;11(2):179-200.

35. Ströhle A, Graetz B, Scheel M, et al. The acute antipanic and anxiolytic activity of aerobic exercise in patients with panic disorder and healthy control subjects. *J Psychiatric Res*. 2009;43(12):1013-7.

ACCEPTED

Copyright © 2016 by the American College of Sports Medicine. Unauthorized reproduction of this article is prohibited.

36. Thom NJ, O'Connor PJ, Clementz BA, Dishman RK. The Effects of an Acute Bout of Moderate Intensity Exercise on Anger and EEG Responses During Elicitation of Angry Emotions. *Med Sci Sports Exerc*. 2010;42(5(Supplement 1)):59.

37. Van Rensburg KJ, Taylor A, Hodgson T. The effects of acute exercise on attentional bias towards smoking-related stimuli during temporary abstinence from smoking. *Addiction*. 2009;104(11):1910-7.

38. Yeung RR. The acute effects of exercise on mood state. *J Psychosomatic Res*. 1996;40(2):123-41.

39. Youngstedt SD. Effects of exercise on sleep. *Clin Sports Med*. 2005;24(2):355-65.

ACCEPTED

Copyright © 2016 by the American College of Sports Medicine. Unauthorized reproduction of this article is prohibited.

Table 1. Baseline Participant Characteristics

	**Male** (N=27)	**Female** (N=26)	**Total**
**Age (y)***	21.7 ± 1.4	20.6 ± 1.47	21.2 ± 1.5
**Weight (kg)***	77.6 ± 6.8	64.4 ± 5.9	71.1 ± 9.2
**BMI (kg/m^2)***	24.1 ± 1.9	22.9 ± 1.9	23.5 ± 2.0
**Underweight (%)**	0	3.8	1.9
**Normal (%)**	66.7	84.6	75.5
**Overweight (%)**	18.5	3.8	11.3
**Smoker (%)***	22.2	3.8	13.2
**Pain (%)**	3.7	0	1.9
**Contraceptive (%)***	0	38.5	18.9
**Physical Activity (kcal/wk)**	305.3 ± 51.3	307.3 ± 95.9	306.3 ± 75.7
**Sleep (PSQI)**	5.1 ± 2.3	5.6 ± 2.8	5.4 ± 2.5
**Poor sleeper (PSQI>5; %)***	22.2	50.0	35.8
**Low trait anxious (%)**	25.9	15.4	20.8
**Intermediate trait anxious (%)**	59.3	50.0	54.7
**High trait anxious (%)***	3.7	26.9	15.1
**Depressed (QIDS>5; %)**	18.5	38.5	28.3
**None (%)**	70.4	50.0	60.4
**Mild depression (%)**	14.8	34.6	24.5
**Moderate depression (%)**	3.7	3.8	3.8
**Trait anxiety (STAI-Y2)***	35.0 ± 6.7	41.6 ± 10.4	38.3 ± 9.3
**State anxiety (STAI-Y1)**	30.1 ± 7.8	33.2 ± 8.9	31.7 ± 8.4
**Tension (POMS)**	1.3 ± 1.7	2.0 ± 2.7	1.7 ± 2.3
**Anger (POMS)**	1.2 ± 2.3	1.2 ± 2.0	1.2 ± 2.1
**Vigour (POMS)**	8.6 ± 4.0	7.4 ± 3.7	8.0 ± 3.8
**Fatigue (POMS)**	2.8 ± 2.6	4.5 ± 3.9	3.7 ± 3.4
**Confusion (POMS)**	4.4 ± 2.3	3.9 ± 1.7	4.2 ± 2.0
**Total mood disturbance (POMS)**	2.2 ± 10.3	5.4 ± 11.3	3.8 ± 10.8
**Depression (QIDS)**	4.3 ± 2.9	5.1 ± 3.5	4.7 ± 3.2
**Worry (PSWQ)***	39.8 ± 12.9	53.2 ± 12.6	46.5 ± 14.3

*Significantly different between genders (p≤0.05)

Abbreviations: y: years; kg: kilograms; BMI: Body Mass Index; kg/m^2: kilograms per meter-squared; kcal/wk: kilocalories per week; PSQI: Pittsburgh Sleep Quality Index; QIDS: Quick Inventory of Depressive Symptomatology; STAI-Y2: Trait Subscale of the State-Trait Anxiety Inventory; STAI-Y1: State Subscale of the State-Trait Anxiety Inventory; POMS: Profile of Mood States; PSWQ: Penn State Worry Questionnaire

ACCEPTED

Copyright © 2016 by the American College of Sports Medicine. Unauthorized reproduction of this article is prohibited.

Table 2. Pre- and Post-Condition Means (SD) and Hedges' *d* effect sizes (95%CI) for Male and Female Participants

	Male					Female				
	Pre-EX	Post-EX	Pre-CON	Post-CON	Hedges' d (95% CI)	Pre-EX	Post-EX	Pre-CON	Post-CON	Hedges' d (95% CI)
**State anxiety (STAI-Y1)**	29.4 (7.6)	27.6 (6.3)	31.8 (11.3)	31.9 (11.3)	0.19 (-0.37, 0.76)	34.3 (8.1)	31.8 (7.6)	32.9 (10.1)	35.1 (10.7)	0.51 (-0.07, 1.08)
**Tension (POMS)**	0.9 (1.5)	0.4 (1.0)	1.4 (2.0)	1.2 (2.2)	0.17 (-0.40, 0.73)	1.8 (2.1)	1.0 (1.3)	1.9 (2.6)	1.3 (1.9)	0.08 (-0.48, 0.65)
**Depression (POMS)**	1.0 (2.0)	0.5 (1.4)	1.4 (3.4)	1.4 (3.4)	0.18 (-0.39, 0.74)	1.0 (2.1)	0.5 (1.3)	1.4 (2.4)	1.7 (2.6)	0.35 (-0.22, 0.92)
**Anger (POMS)**	1.1 (2.2)	0.4 (1.5)	1.2 (2.9)	1.2 (2.7)	0.27 (-0.30, 0.84)	1.0 (1.8)	0.2 (0.5)	1.4 (2.0)	1.4 (2.2)	0.41 (-0.16, 0.99)
**Vigour (POMS)**	8.6 (4.8)	10.6 (3.6)	7.7 (4.3)	7.4 (5.1)	0.50 (-0.08, 1.07)	5.7 (3.4)	9.5 (3.8)	7.5 (4.2)	4.8 (3.5)	1.67 (1.02, 2.33)*
**Fatigue (POMS)**	3.1 (2.6)	2.1 (2.0)	3.8 (3.5)	3.4 (4.3)	0.19 (-0.38, 0.76)	4.3 (3.1)	2.8 (3.0)	4.8 (4.7)	5.7 (5.5)	0.59 (0.01, 1.17)*
**Confusion (POMS)**	3.7 (1.9)	2.3 (1.6)	4.4 (2.7)	4.0 (2.9)	0.42 (-0.15, 0.99)	3.8 (1.4)	2.8 (1.1)	4.0 (1.9)	4.4 (2.0)	0.83 (0.24, 1.41)*
**TMD (POMS)**	1.2 (9.8)	-4.9 (8.2)	4.6 (14.1)	4.3 (16.6)	0.47 (-0.10, 1.04)	6.0 (8.6)	-2.3 (7.1)	6.0 (12.6)	9.7 (12.2)	1.09 (0.49, 1.70)*
**Worry (PSWQ)**	40.0 (13.6)	37.8 (14.8)	39.4 (13.8)	37.0 (14.6)	-0.01 (-0.58, 0.55)	54.2 (12.0)	52.1 (12.8)	52.0 (13.2)	53.1 (11.9)	0.25 (-0.32, 0.82)

*$p < 0.05$

Abbreviations: STAI-Y1: State Subscale of the State-Trait Anxiety Inventory; POMS: Profile of Mood States; PSWQ: Penn State Worry Questionnaire; TMD: Total Mood Disturbance

ACCEPTED

Copyright © 2016 by the American College of Sports Medicine. Unauthorized reproduction of this article is prohibited.
