# Supplementary material for: Does physical activity-based intervention decrease repetitive negative thinking? A systematic review
Source: PLoS One. 2025 Apr 1;20(4):e0319806. doi: 10.1371/journal.pone.0319806 (PMC11960971; doi:10.1371/journal.pone.0319806)
Supplement: S1 File — https://doi.org/10.6084/m9.figshare.25711734. (ZIP) [file pone.0319806.s001.zip › supporting information/paper file/Herring 2019.pdf]

Medicine & Science in Sports & Exercise

The Official Journal of the American College of Sports Medicine
www.acsm-msse.org

. . . Published ahead of Print

Acute Exercise Effects among Young Adults with Analog Generalized Anxiety Disorder

Matthew P. Herring,[1,2] Derek C. Monroe,[3] Brett R. Gordon,[1,2] Mats Hallgren,[4] and Mark J. Campbell[1,5]

[1]Department of Physical Education and Sport Sciences, University of Limerick, Limerick, Ireland; [2]Health Research Institute, University of Limerick, Limerick, Ireland; [3]Department of Neurology, University of California-Irvine, Irvine, CA; [4]Epidemiology of Psychiatric Conditions, Substance Use and Social Environment (EPiCSS), Department of Public Health Sciences, Karolinska Institute, Solna, Sweden; [5]Lero—the Irish Software Research Centre, University of Limerick, Limerick, Ireland

Accepted for Publication: 27 November 2018

Medicine & Science in Sports & Exercise® **Published ahead of Print** contains articles in unedited manuscript form that have been peer reviewed and accepted for publication. This manuscript will undergo copyediting, page composition, and review of the resulting proof before it is published in its final form. Please note that during the production process errors may be discovered that could affect the content.

Copyright © 2018 American College of Sports Medicine

ACCEPTED

Medicine & Science in Sports & Exercise, Publish Ahead of Print
DOI: 10.1249/MSS.0000000000001860

Acute Exercise Effects among Young Adults with Analog Generalized Anxiety Disorder

Matthew P. Herring,[1,2] Derek C. Monroe,[3] Brett R. Gordon,[1,2]
Mats Hallgren,[4] and Mark J. Campbell[1,5]

[1]Department of Physical Education and Sport Sciences, University of Limerick, Limerick, Ireland; [2]Health Research Institute, University of Limerick, Limerick, Ireland; [3]Department of Neurology, University of California-Irvine, Irvine, CA; [4]Epidemiology of Psychiatric Conditions, Substance Use and Social Environment (EPiCSS), Department of Public Health Sciences, Karolinska Institute, Solna, Sweden; [5]Lero—the Irish Software Research Centre, University of Limerick, Limerick, Ireland

Corresponding Author:

Matthew P. Herring, PhD

Department of Physical Education and Sport Sciences and Health Research Institute

University of Limerick

Limerick, Ireland

Email: matthew.herring@ul.ie

+353 061 23 4762

ACCEPTED

Copyright © 2018 by the American College of Sports Medicine. Unauthorized reproduction of this article is prohibited.

Brett R. Gordon is funded by the Irish Research Council under the Government of Ireland Postgraduate Programme. This research did not receive any specific grant from funding agencies in the public, commercial, or not-for-profit sectors. The results of the study are presented clearly, honestly, and without fabrication, falsification, or inappropriate data manipulation.

Conflict of Interest: The authors disclose no conflicts of interest. The results of the present study do not constitute endorsement by the American College of Sports Medicine.

ACCEPTED

Copyright © 2018 by the American College of Sports Medicine. Unauthorized reproduction of this article is prohibited.

Abstract

Recent findings support positive effects of acute aerobic exercise on worry, state anxiety, and feelings of energy and fatigue among young adult women with subclinical, or analogue, Generalized Anxiety Disorder (GAD). However, exercise effects among young adult men with analogue GAD are unstudied. **Purpose:** This study replicated initial findings of positive effects of acute vigorous-intensity aerobic exercise on worry, state anxiety, and feelings of energy and fatigue among young adult women with analogue GAD, examined responses among young adult men with analogue GAD, and explored sex-related differences and moderation by physical activity level, trait anxiety, depression, and poor sleep status. **Methods:** Thirty-five young adults (21.4±2.3y; 19M; 16F) with Penn State Worry Questionnaire scores ≥45 (60±8) completed two counterbalanced 30-min conditions: treadmill running at ~71.2%±0.04% heart rate reserve and seated quiet rest. Outcomes included worry, worry engagement, absence of worry, state anxiety, and feelings of energy and fatigue. **Results:** No outcome or moderator differed at baseline between sexes. Exercise significantly improved state anxiety ($p<0.04$; $d=0.27$) and feelings of energy ($p<0.001$; $d=1.09$). Small non-significant improvements were found for worry ($d=0.22$), worry engagement ($d=0.18$), and feelings of fatigue ($d=0.21$). The magnitude of improvements in worry, worry engagement, absence of worry, and feelings of energy were stronger among females. Significant large, potentially clinically meaningful increases in feelings of energy were found among women ($d=1.35$) and men ($d=0.92$). A non-significant, but potentially clinically meaningful, moderate reduction in worry ($d=0.53$) was found among women. High trait anxiety and poor sleep quality were supported as moderators. **Conclusions:** Findings replicated positive

ACCEPTED

Copyright © 2018 by the American College of Sports Medicine. Unauthorized reproduction of this article is prohibited.

effects of acute aerobic exercise among young adult women with analogue GAD, and extended to support for positive effects among young adult men with analogue GAD.

Keywords: Acute exercise; Worry; Anxiety; Fatigue; Energy; Generalized Anxiety Disorder

ACCEPTED

Copyright © 2018 by the American College of Sports Medicine. Unauthorized reproduction of this article is prohibited.

Introduction

Previous evidence supported positive effects of both aerobic and resistance exercise training among young adult women with Generalized Anxiety Disorder (GAD), including improved clinical severity (1), sleep quality and quantity (2), quality of life (3), and associated symptoms (e.g., worry, anxiety, and feelings of energy and fatigue) (4). More recent evidence highlighted the benefits of a single bout of aerobic exercise among a small sample of young women with subclinical, or analogue, GAD (i.e., elevated worry, the hallmark of GAD) (5). Compared to a 30-min bout of seated rest, a 30-min bout of vigorous-intensity treadmill running completed at ~73% heart rate reserve significantly improved worry engagement, state anxiety, and feelings of energy and fatigue (5). A ¼ standard deviation improvement for worry, approximating a 3.5% change, and moderate-to-large improvements in state anxiety and feelings of energy and fatigue were found (5).

People who report elevated symptom scores are more likely to develop clinically significant psychopathologies (6). It is plausible that exercise-induced attenuation of elevated symptoms may reduce the likelihood of symptom persistence and exacerbation. Thus, investigating exercise effects on the symptom profile of GAD and identifying traits that moderate these effects among individuals with analogue GAD has the potential to inform precision medicine prevention strategies. To the authors' knowledge, the initial findings of positive effects of acute exercise among females with analogue GAD has neither been replicated, nor extended to males.

ACCEPTED

Copyright © 2018 by the American College of Sports Medicine. Unauthorized reproduction of this article is prohibited.

Lifetime prevalence of GAD is nearly twice as high among women (7), who also have a higher likelihood of reporting symptoms that have been improved by exercise (i.e., fatigue, irritability, muscle tension, and somatic symptoms) (4, 8). Therefore, it is perhaps not surprising that the effects of acute exercise on worry, state anxiety, and feelings of energy and fatigue among young adult men have not been investigated. However, GAD is also prevalent among young adult men, and evidence supports small-to-moderate positive effects of acute exercise on state anxiety and feelings of energy and fatigue among otherwise healthy young adult men (9).

Thus, this study tested the effects of acute aerobic exercise compared to quiet rest on worry, state anxiety, and feelings of energy and fatigue among young adult women and men with worry indicative of analogue GAD to: (1) replicate and extend initial positive findings among young adult women with analogue GAD to include young adult men with analogue GAD; (2) to explore potential sex-related differences; and, (3) to replicate preliminary findings that elevated trait anxiety, depression, and poor sleep status moderate responses to exercise or quiet rest conditions. The authors hypothesized that, compared to quiet rest, an acute bout of aerobic exercise would significantly improve worry, state anxiety, and feelings of energy and fatigue among young adult women and men with worry indicative of GAD.

Methods

Design & Participants. The methods reported here are consistent with the authors' previously reported acute aerobic exercise studies (5, 9). The study protocol was approved by the University's Research Ethics Board. Prior to participation, interested potential participants

ACCEPTED

Copyright © 2018 by the American College of Sports Medicine. Unauthorized reproduction of this article is prohibited.

provided written informed consent and completed a medical history screening questionnaire that included the Physical Activity Readiness Questionnaire. Potential participants were screened based on the Penn State Worry Questionnaire (PSWQ) (10). Inclusion criteria were : i) PSWQ score ≥45, which has demonstrated high sensitivity and specificity as a cut-score to identify individuals with elevated worry indicative of GAD (11); ii) age 18-35y; iii) no medical contraindication to safe participation in vigorous aerobic exercise; and, iv) no current pregnancy or lactation. Self-reported physical activity indicative of an active lifestyle was not an exclusion criterion. Of 64 individuals consecutively recruited from the University and surrounding communities using leaflets, recruitment emails to approved listservs, and word-of-mouth, 35 young adults (19 male; 16 female), aged 21.4±2.3y, met inclusion criteria. Based on power analysis performed with G*Power, a sample size of 35 would provide >80% statistical power to detect differences in worry assuming a two-tailed $\alpha=0.05$, a correlation between repeated measures of $r=0.8$, and a small effect of exercise on worry ($f=0.13$, $d=0.26$) based on previous evidence (1, 5, 9). Participants were randomized (www.randomizer.org) by a researcher who did not perform assessments to complete two conditions in counterbalanced order at approximately the same time of day, with approximately 48h between conditions: i) 30-min of vigorous treadmill running, or ii) 30-min seated quiet rest (5, 9, 12).

Baseline Measures. Before testing on day 1, each participant completed electronic versions (www.surveymonkey.com) of a Seven Day Physical Activity Recall (13), the trait subscale of the State-Trait Anxiety Inventory (STAI-Y2) (14), the Quick Inventory of Depressive Symptoms (QIDS) (15), and the Pittsburgh Sleep Quality Index (PSQI) (16). Established cut-scores were

ACCEPTED

Copyright © 2018 by the American College of Sports Medicine. Unauthorized reproduction of this article is prohibited.

used to classify high trait anxious status (STAI-Y2≥50), depression status (QIDS≥6 (15)) and poor sleep quality (PSQI>5 (16)).

Outcomes. Using laboratory desktop PCs, immediately before and 10 minutes following exercise or quiet rest participants completed electronic assessments (www.surveymonkey.com) of worry, state anxiety, and the intensity of feelings of energy and fatigue. This process required approximately 10-15 minutes.

Worry. Worry was assessed using the 16-item PSWQ (10). Item responses are summed after reverse scoring; total scores range from 16-80. Worry engagement (11 items worded in the direction of pathological worry) and absence of worry (5 items reverse-worded to combat acquiescence) subscales were also calculated (10, 17); higher scores indicate worse symptom severity. The PSWQ demonstrated adequate internal consistency (α=0.85), and correlations between repeated measures were r=0.69 and r=0.89 for exercise and quiet rest, respectively. Worry engagement demonstrated similar internal consistency (α=0.86), and correlations between repeated measures were r=0.86 and r=0.67 for exercise and quiet rest, respectively. Internal consistency for absence of worry was poor (α=0.57), and correlations between repeated measures were lower for both exercise (r=0.64) and quiet rest (r=0.60). Recent evidence supported that the PSWQ is sensitive to change in response to acute aerobic exercise (5, 9).

State Anxiety. State anxiety was measured with the 20-item state subscale of the State-Trait Anxiety Inventory (STAI-Y1) (14). Item responses are summed after reverse scoring; total scores range from 20-80. The STAI-Y1 demonstrated adequate internal consistency (α=0.94),

ACCEPTED

Copyright © 2018 by the American College of Sports Medicine. Unauthorized reproduction of this article is prohibited.

and correlations between repeated measures were *r*=0.80 and *r*=0.92 for exercise and control, respectively.

Feelings of Energy and Fatigue. Feelings of energy and fatigue were measured with the vigor and fatigue subscales of the Profile of Mood States – Brief Form (POMS-B). These subscales reliably measure the intensity of feelings of energy and fatigue (18, 19). Participants were instructed to respond "based on how you feel RIGHT NOW." Subscale scores range from 0-20. Internal consistency was adequate for fatigue (α=0.84) and vigor (α=0.88). For the fatigue subscale, correlations between repeated measures were *r*=0.41 and *r*=0.75 for exercise and control, respectively. For the vigor subscale, correlations between repeated measures were *r*=0.50 and *r*=0.81, respectively.

Conditions

Acute Aerobic Exercise. Participants completed a vigorous (20) 30-min supervised bout of running on a Woodway Pro® treadmill at 65%-85% of maximal heart rate reserve (%HRR). Intensity was estimated as a function of predicted maximal heart rate and an age-related norm resting heart rate of 73bpm for females and 63bpm for males. This method has demonstrated validity for estimating vigorous-intensity exercise dose in field-based exercise studies (21). Polar FT60® HR monitors were used to set the heart rate training zone and to continuously monitor HR and in-zone exercise minutes during exercise. Each participant performed a 5-min warm-up, continued running while investigators progressively increased the treadmill speed and/or grade to achieve 65%HRR, and then completed 30 min at a heart rate between 65%-

ACCEPTED

Copyright © 2018 by the American College of Sports Medicine. Unauthorized reproduction of this article is prohibited.

85%HRR. Most participants achieved 65%HRR within five minutes; therefore, warm-up periods ranged from 1-5min. After completing 30 min at 65%-85%HRR, each participant walked until sufficiently cooled down. Again, some participants cooled down more rapidly than others; therefore, the cool-down period ranged from 1-5min. Following completion of cool-down, each participant provided a session Rating of Perceived Exertion (RPE) for the full exercise bout using Borg's 6-20 RPE scale (22). Standard instructions for RPE were provided to each individual. Muscle pain intensity was assessed with the 0-10 pain intensity scale developed and validated by Cook and colleagues (23). Given the potential mood effects of social interaction (24), extraneous conversation was minimized during the exercise session.

Control: Seated Quiet Rest. Each participant completed 30 min of seated quiet rest in the same laboratory. Procedures were consistent with the exercise session except that each participant sat in an upright chair in a quiet area for 30 min. Participants were not allowed to read or listen to music given the potential for altered mood responses.

Analyses. Data analyses were performed using SPSS 22.0. Missing data for state anxiety (n=5), worry (n=6), feelings of fatigue (n=4), and feelings of energy (n=4) were imputed by multiple imputation: gender and time-variant responses for each variable were entered as predictors into separate multiple linear regression models and predicted values were retained. Independent samples *t*-tests examined baseline differences in outcomes based on gender and high trait anxious, depression, and poor sleep status.

ACCEPTED

Copyright © 2018 by the American College of Sports Medicine. Unauthorized reproduction of this article is prohibited.

Two condition (exercise/quiet rest) X two time (pre/post) repeated measures ANOVA examined differences between exercise and quiet rest. Significant interactions were decomposed with simple effects analyses Bonferroni-corrected for multiple testing. Given increased calls to move beyond null-hypothesis significance testing in favor of effect sizes and confidence intervals (25), we also quantified and compared the magnitude of outcome change using Hedges' *d* and associated 95% confidence intervals (95%CI) such that improved outcomes resulted in positive effect sizes (26). Effect sizes were judged as small (0.2), moderate (0.5), and large (0.8) based on Cohen's thresholds.

Potential sex-related differences were examined using two group (male/female) X two condition (exercise/quiet rest) X two time (pre/post) repeated measures ANOVA. Hedges' *d* (95%CI) quantified magnitude of outcome change for males and females.

Independent samples *t*-tests of pre-post exercise and pre-post quiet rest change scores analyzed within-condition differences in outcomes based on high trait anxious status, depression status, and poor sleep status. Pearson's correlation coefficients quantified associations between physical activity and outcome change scores for each condition.

Results

Baseline Characteristics and Differences. Table 1 presents baseline participant characteristics.

ACCEPTED

Copyright © 2018 by the American College of Sports Medicine. Unauthorized reproduction of this article is prohibited.

***Sex-Related Differences*.** No outcome or potential moderator significantly differed between sexes at baseline: worry (t_{33}=1.80, p>0.08), worry engagement (t_{33}=1.46, p>0.15), absence of worry ($t_{27.3}$=1.67, p>0.10), state anxiety ($t_{19.6}$=1.94, p>0.06), and feelings of energy (t_{33}=1.86, p>0.12) and fatigue (t_{33}=1.80, p>0.07), prior week physical activity (t_{33}=0.63, p>0.53), self-reported minutes of moderate-to-vigorous physical activity (t_{33}=0.29, p>0.77), STAI-Y2 ($t_{26.81}$=1.81, p>0.08), high trait anxious status (X_1=3.33, p>0.06), PSQI total score (t_{32}=0.20, p>0.84), and poor sleep quality status (X_1=1.80, p>0.17).

***Differences Based on High Trait Anxious, Depression, and Poor Sleep Status*.** High trait anxious participants reported significantly higher worry (t_{33}=-3.77, $p \leq 0.001$), worry engagement (t_{33}=-4.11, p<0.001), state anxiety ($t_{11.5}$=-5.32, p<0.001), and feelings of fatigue ($t_{11.2}$=-2.36, p<0.03). Depressed participants reported significantly higher state anxiety (t_{33}=-3.22, $p \leq 0.003$) and feelings of fatigue ($t_{27.97}$=-3.00, $p \leq 0.006$). Poor sleepers reported significantly higher worry (t_{32}=-2.38, p<0.03) and absence of worry (t_{32}=-3.27, $p \leq 0.003$).

Exercise Session Variables. Participants averaged 35.8±2.4 min of exercise, 30.4±0.4 min in-zone. Participants exercised at ~71.2±0.04 %HRR, approximating an average session heart rate of 160.8±5.2 bpm, an average session RPE of 13±2, and an average session pain rating of 3±2. All participants completed >30min at 65%-85%HRR; there was little variability in in-zone minutes, speed, or gradient, and differences in total exercise time and exact %HRR at which participants exercised were not significantly associated with outcome responses (all p>0.09).

ACCEPTED

Copyright © 2018 by the American College of Sports Medicine. Unauthorized reproduction of this article is prohibited.

Effects of Exercise Compared to Quiet Rest. Table 2 presents descriptives, standardized mean differences, paired *t*-tests, and Hedges' *d* (95%CI). Statistically significant Condition X Time interactions were found for state anxiety ($F_{(1,34)}$=4.64, p<0.04; d=0.27, 95%CI: [-0.20, 0.74]) and feelings of energy ($F_{(1,34)}$=42.30, p<0.001; d=1.09, [0.59, 1.59]). State anxiety was significantly reduced by exercise (mean difference=-3.92, $p \leq 0.001$), and significantly lower for exercise compared to quiet rest post-condition (mean difference=-4.62, $p \leq 0.004$). Feelings of energy were significantly increased by exercise (mean difference=2.86, p<0.001), significantly decreased by quiet rest (mean difference=-1.82, p<0.001), and significantly greater for exercise compared to quiet rest post-condition (mean difference=3.94, p<0.001). Condition X Time interactions were not significant for worry ($F_{(1,34)}$=1.46, p>0.23; d=0.22, [-0.25, 0.69]), absence of worry ($F_{(1,34)}$=0.01, p>0.91; d=0.03, [-0.44, 0.50]), worry engagement ($F_{(1,34)}$=0.80, p>0.37; d=0.18, [-0.29, 0.65]), or feelings of fatigue ($F_{(1,34)}$=0.75, p>0.39; d=0.21, [-0.26, 0.68]). Significant main effects for time were found for worry ($F_{(1,34)}$=6.11, p<0.02) and worry engagement ($F_{(1,34)}$=4.15, p<0.05). Worry (mean difference=-1.85, p<0.02) and worry engagement (mean difference=-1.14, p<0.05) were significantly lower post-condition.

Sex-Related Differences. Table 3 presents descriptives and standardized mean differences across gender and condition. Sex X Condition X Time interactions were not significant for any outcome (all p>0.11). As shown in Figure 1, compared to quiet rest, exercise resulted in a significant large increase in feelings of energy, non-significant moderate improvements in worry and absence of worry, and non-significant small reductions in worry engagement and state anxiety among women. Among men, compared to quiet rest, exercise resulted in significant large increases in feelings of energy and small-to-moderate reductions in feelings of fatigue, and

ACCEPTED

Copyright © 2018 by the American College of Sports Medicine. Unauthorized reproduction of this article is prohibited.

non-significant small-to-moderate reductions in state anxiety. Effect sizes were larger among women than men for worry, worry engagement, absence of worry, and feelings of energy.

Potential Moderators of Exercise Effects. Significantly larger improvements in absence of worry (t_{33}=2.15, $p \leq 0.039$; d=0.76) and state anxiety (t_{33}=2.25, $p \leq 0.031$; d=0.70) were found among high trait anxious participants. Significantly larger reductions in state anxiety were found among poor sleepers (t_{32}=2.77, $p \leq 0.009$; d=0.57). No significant differences were found based on depression status or between potential moderator levels for quiet rest. Estimated energy expenditure and minutes of MVPA were not significantly associated with any outcome response to exercise or quiet rest.

Discussion

Compared to 30 minutes of quiet rest, a 30-minute bout of treadmill running completed at an average intensity of ~71%HRR significantly improved state anxiety and feelings of energy among young adult men and women with analogue GAD. Though not statistically significant, small magnitude improvements were found for worry, worry engagement, and feelings of fatigue. The magnitude of these improvements is consistent with previously reported responses to acute exercise among otherwise healthy adults (9, 12, 27), people with Multiple Sclerosis (28), low-income adults with elevated depressive symptoms (29), and adult women diagnosed with Major Depressive Disorder (30). Though not significantly different, the magnitude of exercise-induced changes in worry, worry engagement, and absence of worry was larger among women than men.

Copyright © 2018 by the American College of Sports Medicine. Unauthorized reproduction of this article is prohibited.

Feelings of Energy. Given that low energy is a prevalent associated symptom of GAD that may contribute to negative outcomes like lower health-related quality of life (3), automobile accidents (31), and reduced achievement and productivity (32), the large, significant improvements in feelings of energy found here may be statistically and clinically meaningful. The magnitude of improvement in feelings of energy is consistent with previous reports of moderate-to-large improvements in feelings of energy following acute exercise and exercise training among otherwise healthy adults (9, 27), young women with persistent fatigue (33), and young women with analogue (5) and clinical GAD (4). Though debates continue regarding interpretations of clinical meaningfulness, based on the suggested minimal important difference of ½ standard deviation (34), the 1.09 standard deviation improvement in feelings of energy here may represent a clinically meaningful finding.

Comparability of Findings for Worry & Plausible Explanations. The small magnitude improvement in worry (d=0.22), the hallmark of GAD, found for the full sample was comparable to the previously reported effect among young adult women with analogue GAD (d=0.25) (5). However, the magnitude of the overall effect can be attributed to a null effect among men and a moderate, but potentially clinically meaningful (34), effect among women (d=0.53) that was twice the size of the previously reported effect (5). A poorer baseline symptom profile here may have contributed to this larger magnitude effect; the current sample of young adult women reported worse worry, state anxiety, and feelings of energy and fatigue than both the current sample of men and the previous sample with analogue GAD (5).

ACCEPTED

Copyright © 2018 by the American College of Sports Medicine. Unauthorized reproduction of this article is prohibited.

Consistent with a previous report that acute vigorous-intensity exercise is not sufficient to reduce worry among otherwise healthy young adult men (9), exercise trivially influenced worry and response did not differ to quiet rest. It is plausible that worry is not as sensitive to acute exercise among males, and/or that different neurobiological mechanisms underlie response and non-response to exercise among females and males, respectively. One plausible explanation is greater cardiac vagal tone expressed by females relative to males (35). A model of neurovisceral integration suggests that a relationship between poor vagal tone and elevated worry in GAD patients (36) may be explained by deficits in cognitive control and inhibition (37). This explanation is supported by recent evidence of an association between sex-specific attentional biases and GAD symptom profiles (38). However, because no study has examined exercise effects on autonomic function or cognitive control in GAD, how those effects influence worry or related outcome responses remains unclear.

Differential Responses to Quiet Rest. Consistent with previous findings (5, 9), the magnitude of between-condition differences was influenced by differential responses to quiet rest. Hedges' *d* effect sizes were larger for nearly all outcomes because of trivial or negative responses to quiet rest. It is somewhat surprising that imposed seated rest would worsen outcomes, but these findings are consistent with reported worsened mood outcomes following imposed sedentary behavior among physically active adults (39). These findings encourage future investigations of factors that may underlie worsening of mood states during imposed resting conditions (e.g., sedentary behavior and rumination) and the most appropriate comparison condition for acute exercise (e.g., minimal intervention or placebo-control conditions such as light-intensity walking or passive cycling).

ACCEPTED

Copyright © 2018 by the American College of Sports Medicine. Unauthorized reproduction of this article is prohibited.

Susceptibility Profiles: Plausible Role of High Trait Anxious & Poor Sleep Status. Identifying susceptibility profiles that inform the use of exercise as a precision medicine strategy is important; thus, it is notable that improvements in state anxiety and absence of worry following exercise were significantly larger among high trait anxious participants and poor sleepers. Because trait anxiety and state anxiety are strongly correlated (14), it is unsurprising that baseline state anxiety was significantly higher among high trait anxious participants compared to non-high trait anxious. High state anxiety among high trait anxious individuals and elevated worry symptoms among poor sleepers likely contributed to the larger magnitude change among these participants. Although adults with mild GAD frequently experience prolonged sleep latency, lower total sleep time, and poor sleep efficiency (40, 41), no previous study has documented anxiolytic effects of acute exercise among young adults reporting poor sleep quality and analogue GAD. These findings are consistent with previous evidence that anxiety accounts for significant variability in the sleep quality of young adults (42). It is possible that post-exercise symptom trajectories (43) for state anxiety and absence of worry are at least partly dependent on trait anxiety and sleep quality. Future research should leverage advanced mobile technologies (e.g., ecological momentary assessment) to probe the time-course of these effects.

Limitations. One potential limitation was the somewhat favorable baseline profile among men, characterized by lower state anxiety and feelings of fatigue and higher feelings of energy, which may not be reflective of population heterogeneity among young men with analogue or clinical GAD. Although an active lifestyle was not an exclusion criterion and physical activity was not significantly associated with outcome responses, the activity profile of included participants may

ACCEPTED

Copyright © 2018 by the American College of Sports Medicine. Unauthorized reproduction of this article is prohibited.

be a limitation such that both exercise intensity and exercise effects may have been underestimated among highly active participants. Because physical activity is lower among other groups with elevated anxiety and/or depressive symptoms and disorders, the active group here may not accurately represent physical activity levels in analogue or clinical GAD. However, the large-scale evidence regarding physical activity among young adults with analogue or clinical GAD needed to place the current levels into context is limited, highlighting the need for future reports of physical activity levels among large samples. Using gender-based age-related norms for resting heart rate to estimate vigorous-intensity exercise dose has demonstrated validity (21). However, it is plausible that resting heart rate may be higher among high-anxious individuals and lower among the active participants here, which could have resulted in over- or underestimation of exercise intensity. Nonetheless, intensity did not significantly vary and was not associated with outcome change. Future research can benefit from larger sample sizes, comparisons of varying degrees of subclinical and clinical GAD severity, the comparison of multiple exercise modes and intensities, the use of advanced mobile technologies to elucidate the time-course of exercise response, and examination of plausible cognitive and biological mechanisms governing exercise response.

Conclusions. Notwithstanding potential limitations, these findings support the authors' initial report of positive effects of acute aerobic exercise on worry, state anxiety, and feelings of energy and fatigue among young adult women with analogue GAD. These findings also provide initial support for these positive effects among young adult men with analogue GAD.

ACCEPTED

Copyright © 2018 by the American College of Sports Medicine. Unauthorized reproduction of this article is prohibited.

Acknowledgements

Brett R. Gordon is funded by the Irish Research Council under the Government of Ireland Postgraduate Programme. This research did not receive any specific grant from funding agencies in the public, commercial, or not-for-profit sectors. The results of the study are presented clearly, honestly, and without fabrication, falsification, or inappropriate data manipulation.

Conflict of Interest

The authors disclose no conflicts of interest. The results of the present study do not constitute endorsement by the American College of Sports Medicine.

ACCEPTED

Copyright © 2018 by the American College of Sports Medicine. Unauthorized reproduction of this article is prohibited.

References

1. Herring MP, Jacob ML, Suveg C, Dishman RK, O'Connor PJ. Feasibility of exercise training for the short-term treatment of generalized anxiety disorder: a randomized controlled trial. *Psychother Psychosom*. 2012;81(1):21-8.

2. Herring MP, Kline CE, O'Connor PJ. Effects of exercise on sleep among young women with Generalized Anxiety Disorder. *Ment Health Phys Act*. 2015;9:59-66.

3. Herring MP, Johnson KE, O'Connor PJ. Exercise training and health-related quality of life in generalized anxiety disorder. *Psychol Sport Exerc*. 2016;27:138-41.

4. Herring MP, Jacob ML, Suveg C, O'Connor PJ. Effects of short-term exercise training on signs and symptoms of generalized anxiety disorder. *Ment Health Phys Act*. 2011;4(2):71-7.

5. Herring MP, Hallgren M, Campbell MJ. Acute exercise effects on worry, state anxiety, and feelings of energy and fatigue among young women with probable Generalized Anxiety Disorder: A pilot study. *Psychol Sport Exerc*. 2017;33:31-6.

6. Wolitzky- Taylor K, Dour H, Zinbarg R, Mineka S, Vrshek- Schallhorn S, Epstein A, et al. Experiencing core symptoms of anxiety and unipolar mood disorders in late adolescence predicts disorder onset in early adulthood. *Depress Anxiety*. 2014;31(3):207-13.

7. Bandelow B, Michaelis S. Epidemiology of anxiety disorders in the 21st century. *Dialogues Clin Neurosci*. 2015;17(3):327.

8. Vesga-López O, Schneier F, Wang S, et al. Gender differences in generalized anxiety disorder: results from the National Epidemiologic Survey on Alcohol and Related Conditions (NESARC). *J Clin Psychiatry*. 2008;69(10):1606.

9. McDowell CP, Campbell MJ, Herring MP. Sex-related differences in mood responses to acute aerobic exercise. *Med Sci Sports Exerc*. 2016;48(9):1798-802.

ACCEPTED

Copyright © 2018 by the American College of Sports Medicine. Unauthorized reproduction of this article is prohibited.

10. Meyer TJ, Miller ML, Metzger RL, Borkovec TD. Development and validation of the penn state worry questionnaire. *Behav Res Ther*. 1990;28(6):487-95.

11. Behar E, Alcaine O, Zuellig AR, Borkovec T. Screening for generalized anxiety disorder using the Penn State Worry Questionnaire: A receiver operating characteristic analysis. *J Behav Ther Exp Psychiatry*. 2003;34(1):25-43.

12. Ensari I, Greenlee TA, Motl RW, Petruzzello SJ. Meta-analysis of acute exercise effects on state anxiety: An update of randomized controlled trials over the past 25 years. *Depress Anxiety*. 2015;32(8):624-634..

13. Blair SN, Haskell WL, Ho P, et al. Assessment of habitual physical activity by a sevenday recall in a community survey and controlled experiments. *Am J Epidemiol*. 1985;122(5):794-804.

14. Spielberger CD, Gorsuch RL, Lushene RE. *Manual for the state-trait anxiety inventory (form Y): self-evaluation questionnaire*. 1st ed. Palo Alto: Consulting Psychologists Press; 1983.

15. Rush AJ, Trivedi MH, Ibrahim HM, et al. The 16-Item Quick Inventory of Depressive Symptomatology (QIDS), clinician rating (QIDS-C), and self-report (QIDS-SR): a psychometric evaluation in patients with chronic major depression. *Biol Psychiatry*. 2003;54(5):573-583.

16. Buysse DJ, Reynolds III CF, Monk TH, Berman SR, Kupfer DJ. The Pittsburgh Sleep Quality Index: a new instrument for psychiatric practice and research. *Psychiatry Res*. 1989;28(2):193-213.

17. Fresco DM, Heimberg RG, Mennin DS, Turk CL. Confirmatory factor analysis of the Penn State worry questionnaire. *Behav Res Ther*. 2002;40(3):313-23.

18. McNair DM, Droppleman LF, Lorr M. *Edits manual for the profile of mood states: POMS*. Edits; 1992.

ACCEPTED

Copyright © 2018 by the American College of Sports Medicine. Unauthorized reproduction of this article is prohibited.

19. O'Connor PJ. Evaluation of four highly cited energy and fatigue mood measures. *J Psychosom Med*. 2004;57(5):435-41.

20. Physical Activity Guidelines Advisory Committee. *Physical activity guidelines advisory committee report, 2008*. Washington, DC: US Department of Health and Human Services; 2008.

21. Miller FL, O'Connor DP, Herring MP, et al. Exercise Dose, Exercise adherence, and associated health outcomes in the TIGER Study. *Med Sci Sports Exerc*. 2014;46(1):69.

22. Borg GA. Psychophysical bases of perceived exertion. *Med Sci Sports Exerc*. 1982;14(5):377-81.

23. Cook DB, O'Connor PJ, Eubanks SA, Smith JC, Lee M. Naturally occurring muscle pain during exercise: assessment and experimental evidence. *Med Sci Sports Exerc*. 1997;29(8):999-1012.

24. McNeil JK, LeBlanc EM, Joyner M. The effect of exercise on depressive symptoms in the moderately depressed elderly. *Psychol Aging*. 1991;6(3):487.

25. Cumming G. The new statistics why and how. *Psychol Sci*. 2013:0956797613504966.

26. Hedges LV, Olkin I. *Statistical methods for meta-analysis*. New York: Academic Press; 1985.

27. Loy BD, O'Connor PJ, Dishman RK. The effect of a single bout of exercise on energy and fatigue states: a systematic review and meta-analysis. *Fatigue*. 2013;1(4):223-42.

28. Ensari I, Sandroff BM, Motl RW. Intensity of treadmill walking exercise on acute mood symptoms in persons with multiple sclerosis. *Anxiety Stress Coping*. 2016:30(1)15-25.

29. Legrand FD, Race M, Herring MP. Acute effects of outdoor and indoor exercise on feelings of energy and fatigue in people with depressive symptoms. *J Environ Psychol*. 2018;56:91-6.

ACCEPTED

Copyright © 2018 by the American College of Sports Medicine. Unauthorized reproduction of this article is prohibited.

30. Meyer JD, Koltyn KF, Stegner AJ, Kim J-S, Cook DB. Influence of exercise intensity for improving depressed mood in depression: A dose-response study. *Behav Ther*. 2016;47(4):527-37.

31. Martiniuk AL, Senserrick T, Lo S, Williamson A, Du W, Grunstein RR, et al. Sleep-deprived young drivers and the risk for crash: the DRIVE prospective cohort study. *JAMA Pediatrics*. 2013;167(7):647-55.

32. Fukuda S, Yamano E, Joudoi T, Mizuno K, Tanaka M, Kawatani J, et al. Effort-reward imbalance for learning is associated with fatigue in school children. *Behav Med*. 2010;36(2):53-62.

33. Herring MP, O'Connor PJ. The effect of acute resistance exercise on feelings of energy and fatigue. *J Sports Sci*. 2009;27(7):701-9.

34. Norman GR, Sloan JA, Wyrwich KW. Interpretation of changes in health-related quality of life: the remarkable universality of half a standard deviation. *Med Care*. 2003;41(5):582-92.

35. Abhishekh HA, Nisarga P, Kisan R, Meghana A, Chandran S, Raju T, et al. Influence of age and gender on autonomic regulation of heart. *J Clin Monit*. 2013;27(3):259-64.

36. Thayer JF, Friedman BH, Borkovec TD. Autonomic characteristics of generalized anxiety disorder and worry. *Biol Psychiatry*. 1996;39(4):255-66.

37. Thayer JF, Lane RD. Claude Bernard and the heart–brain connection: Further elaboration of a model of neurovisceral integration. *Neurosci Biobehav Rev*. 2009;33(2):81-8.

38. Kinney K, Boffa J, Amir N. Gender difference in attentional bias toward negative and positive stimuli in Generalized Anxiety Disorder. *Behav Ther*. 2017;48(3):277-84.

39. Edwards MK, Loprinzi PD. Experimentally increasing sedentary behavior results in increased anxiety in an active young adult population. *J Affect Disord*. 2016;204:166-73.

ACCEPTED

Copyright © 2018 by the American College of Sports Medicine. Unauthorized reproduction of this article is prohibited.

40. Monti JM, Monti D. Sleep disturbance in generalized anxiety disorder and its treatment. *Sleep Med Rev*. 2000;4(3):263-76.

41. Papadimitriou GN, Linkowski P. Sleep disturbance in anxiety disorders. *Int Rev Psychiatry*. 2005;17(4):229-36.

42. Lund HG, Reider BD, Whiting AB, Prichard JR. Sleep patterns and predictors of disturbed sleep in a large population of college students. *J Adolesc Health*. 2010;46(2):124-32.

43. Dyer III JB, Crouch JG. Effects of running on moods: A time series study. *Percept Mot Skills*. 1987;64(3):783-9.

ACCEPTED

Copyright © 2018 by the American College of Sports Medicine. Unauthorized reproduction of this article is prohibited.

Figure Legends

Figure 1. Hedges' *d* Effect Sizes for Outcome Measures by Sex; asterisks indicate significant improvement for exercise compared to quiet rest based on 95%CI not encompassing 0.

ACCEPTED

Copyright © 2018 by the American College of Sports Medicine. Unauthorized reproduction of this article is prohibited.

Figure 1

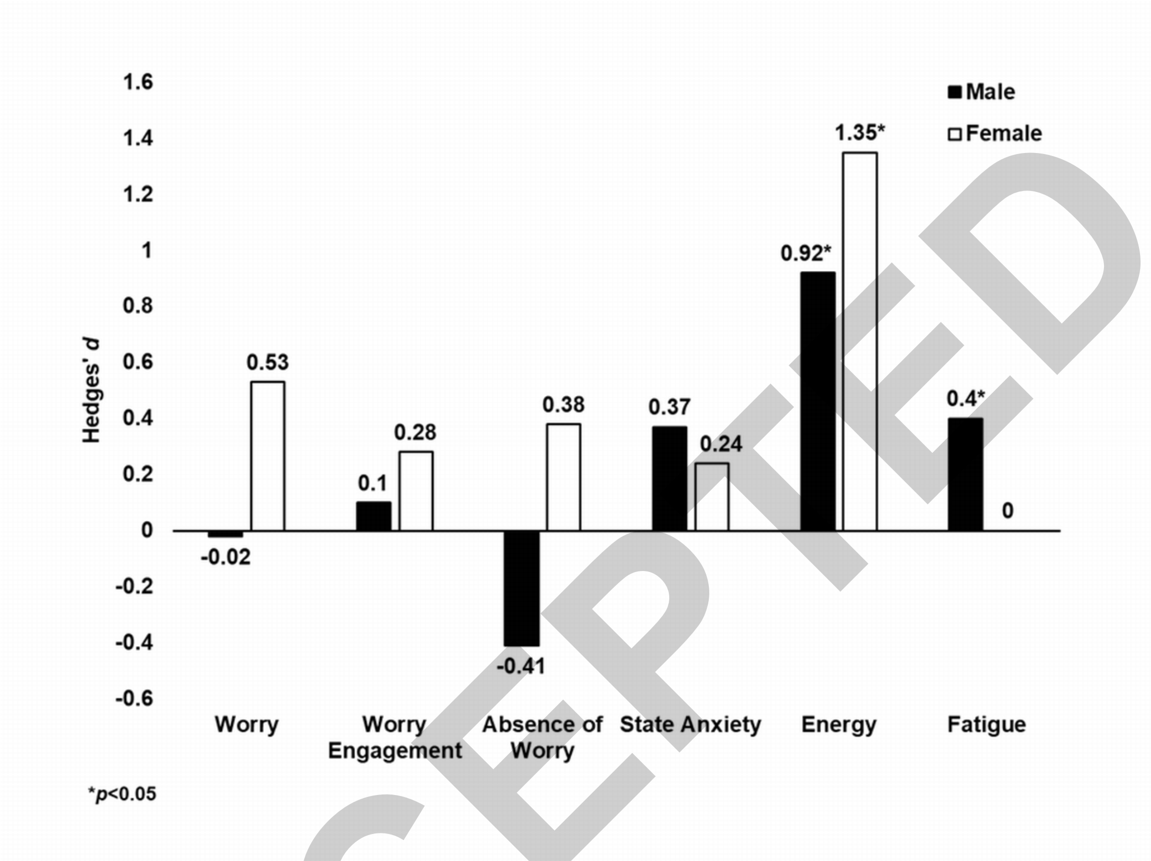

Copyright © 2018 by the American College of Sports Medicine. Unauthorized reproduction of this article is prohibited.

Table 1. Baseline Participant Characteristics

	Male (n=19)	Female (n=16)	Total (n=35)
**Age (y)**	21.6±1.2	21.2±3.2	21.4±2.3
**Weight (kg)**	78.7±8.3	65.4±9.0	72.8±10.8
**BMI (kg/m^2)**	24.1±1.8	23.1±2.8	23.6±2.3
**Overweight (%)**	5 (26.3%)	3 (18.8%)	8 (22.9%)
**Physical Activity (kcal/wk)**	284.4±45.4	293.3±35.7	288.5±40.9
**Moderate-to-Vigorous Physical Activity (min/wk)**	811.6±583.3	864.4±463.4	835.7±525.0
**Sleep (PSQI)**	5.5±2.7	5.7±2.9	5.6±2.7
**Poor sleeper (PSQI>5; %)**	6 (31.6%)	9 (56.3%)	15 (42.9%)
**High trait anxious (%)**	3 (15.8%)	7 (43.8%)	10 (28.6%)
**Depressed (QIDS>5; %)**	9 (47.4%)	10 (62.5%)	19 (54.3%)
**None (%)**	10 (52.6%)	6 (37.5%)	16 (45.7%)
**Mild depression (%)**	8 (42.1%)	6 (37.5%)	14 (40.0%)
**Moderate depression (%)**	1 (5.3%)	4 (25.0%)	5 (14.3%)
**Trait anxiety (STAI-Y2)**	41.9±7.8	47.8±10.8	44.6±9.6
**State anxiety (STAI-Y1)**	35.6±6.6	43.6±15.3	39.3±12.0
**Feelings of Energy (POMS-B)**	8.8±3.6	6.6±4.5	7.8±4.1
**Feelings of Fatigue (POMS-B)**	3.5±2.5	5.3±3.3	4.3±3.0
**Depression (QIDS)**	5.7±3.4	7.4±3.8	6.5±3.7
**Worry (PSWQ)**	57.6±8.0	62.6±8.1	59.9±8.3
**Worry Engagement (PSWQ)**	38.5±7.3	41.9±6.1	40.1±6.9
**Absence of Worry (PSWQ)**	19.1±2.3	20.7±3.1	19.8±2.8

Abbreviations: y: years; kg: kilograms; BMI: Body Mass Index; kg/m^2: kilograms per meter-squared; kcal/wk: kilocalories per week; PSQI: Pittsburgh Sleep Quality Index; QIDS: Quick Inventory of Depressive Symptomatology; STAI-Y2: Trait Subscale of the State-Trait Anxiety Inventory; STAI-Y1: State Subscale of the State-Trait Anxiety Inventory; POMS-B: Profile of Mood States-Brief; PSWQ: Penn State Worry Questionnaire

ACCEPTED

Copyright © 2018 by the American College of Sports Medicine. Unauthorized reproduction of this article is prohibited.

Table 2. Pre- and Post-Condition Means (SD), Standardized Mean Differences (d), Within-Condition Change, and Hedges' *d* (95%CI)

	Pre-EX	Post-EX	SMD (d)	*t*	*p*	Pre-CON	Post-CON	SMD (d)	*t*	*p*	Hedges' *d* (95% CI)
**Worry (PSWQ)**	61.0±8.1	58.2±10.8	0.34	2.08	**<0.05**	60.2±8.7	59.3±9.1	0.11	1.30	>0.20	0.22 (-0.25, 0.69)
**Worry Engagement (PSWQ)**	40.6±7.0	38.9±9.0	0.26	2.26	**≤0.03**	40.0±7.1	39.5±7.7	0.07	0.48	>0.63	0.18 (-0.29, 0.65)
**Absence of Worry (PSWQ)**	19.8±2.6	19.4±3.0	0.18	1.16	>0.25	20.0±3.0	19.6±3.0	0.14	0.89	>0.38	0.03 (-0.44, 0.50)
**State Anxiety (STAI-Y1)**	37.9±10.3	34.0±9.9	0.38	3.62	**≤0.001**	39.3±13.4	38.6±13.8	0.05	0.72	>0.47	0.27 (-0.20, 0.74)
**Feelings of Energy (POMS-B)**	6.9±4.0	9.8±3.7	0.71	4.35	**≤0.001**	7.7±4.5	5.8±4.0	-0.41	4.10	**<0.001**	1.09 (0.59, 1.59)
**Feelings of Fatigue (POMS-B)**	4.9±3.9	4.2±4.1	0.20	1.04	>0.30	5.1±4.1	5.2±4.3	-0.02	-0.20	>0.84	0.21 (-0.26, 0.68)

Bolded *p*-values are statistically significant at $p<0.05$ based on paired *t*-tests.

Abbreviations: STAI-Y1: State Subscale of the State-Trait Anxiety Inventory; POMS: Profile of Mood States-Brief; PSWQ: Penn State Worry Questionnaire

ACCEPTED

Copyright © 2018 by the American College of Sports Medicine. Unauthorized reproduction of this article is prohibited.

Table 3. Pre- and Post-Condition Means (SD), Standardized Mean Differences (d), and Hedges' *d* effect sizes (95%CI) for Male and Female Participants

	Male (n=19)							Female (n=16)						
	Pre-EX	Post-EX	SMD (d)	Pre-CON	Post-CON	SMD (d)	Hedges' *d* (95% CI)	Pre-EX	Post-EX	SMD (d)	Pre-CON	Post-CON	SMD (d)	Hedges' *d* (95% CI)
**Worry (PSWQ)**	57.7±7.1	57.2±10.0	0.07	58.0±8.7	57.3±9.6	0.08	-0.02 (-0.66, 0.61)	64.9±7.7	59.4±11.9	0.71	62.9±8.1	61.7±8.1	0.15	0.53 (-0.17, 1.24)
**Worry Engagement (PSWQ)**	39.0±6.7	38.1±8.6	0.13	38.1±7.3	37.9±8.4	0.03	0.10 (-0.54, 0.73)	42.6±7.0	39.8±9.6	0.40	42.2±6.2	41.3±6.5	0.15	0.28 (-0.41, 0.98)
**Absence of Worry (PSWQ)**	18.7±1.6	19.1±2.3	-0.25	19.4±2.6	18.9±2.6	0.19	-0.41 (-1.05, 0.23)	21.2±2.9	19.7±3.8	0.52	20.7±3.2	20.4±3.4	0.09	0.38 (-0.32, 1.08)
**State Anxiety (STAI-Y1)**	34.6±6.2	31.2±5.1	0.55	35.5±8.9	35.0±9.7	0.06	0.37 (-0.27, 1.01)	41.9±12.7	37.3±13.0	0.36	43.8±16.6	42.9±16.7	0.05	0.24 (-0.45, 0.94)
**Feelings of Energy (POMS-B)**	8.4±3.8	10.7±3.1	0.61	8.3±4.3	6.8±4.1	-0.35	0.92 (0.25, 1.59)*	5.1±3.6	8.7±4.2	1.00	6.9±4.7	4.7±3.6	-0.47	1.35 (0.58, 2.12)*
**Feelings of Fatigue (POMS-B)**	4.5±3.8	3.6±3.5	0.24	4.1±3.6	4.7±4.5	0.17	0.40 (0.25, 1.04)*	5.4±4.0	4.9±4.7	0.13	6.4±4.5	5.9±4.0	0.11	0.00 (-0.69, 0.69)

* $p < 0.05$; significant improvement for exercise compared to quiet rest based on 95%CI not encompassing 0.

Abbreviations: STAI-Y1: State Subscale of the State-Trait Anxiety Inventory; POMS: Profile of Mood States-Brief; PSWQ: Penn State Worry Questionnaire

ACCEPTED

Copyright © 2018 by the American College of Sports Medicine. Unauthorized reproduction of this article is prohibited.
