# Supplementary material for: Does physical activity-based intervention decrease repetitive negative thinking? A systematic review
Source: PLoS One. 2025 Apr 1;20(4):e0319806. doi: 10.1371/journal.pone.0319806 (PMC11960971; doi:10.1371/journal.pone.0319806)
Supplement: S1 File — https://doi.org/10.6084/m9.figshare.25711734. (ZIP) [file pone.0319806.s001.zip › supporting information/paper file/Alderman 2016.pdf]

OPEN

Citation: Transl Psychiatry (2016) **6**, e726; doi:10.1038/tp.2015.225

www.nature.com/tp

ORIGINAL ARTICLE

MAP training: combining meditation and aerobic exercise reduces depression and rumination while enhancing synchronized brain activity

BL Alderman[1], RL Olson[1,2], CJ Brush[1] and TJ Shors[3]

Mental and physical (MAP) training is a novel clinical intervention that combines mental training through meditation and physical training through aerobic exercise. The intervention was translated from neuroscientific studies indicating that MAP training increases neurogenesis in the adult brain. Each session consisted of 30 min of focused-attention (FA) meditation and 30 min of moderate-intensity aerobic exercise. Fifty-two participants completed the 8-week intervention, which consisted of two sessions per week. Following the intervention, individuals with major depressive disorder (MDD; $n=22$) reported significantly less depressive symptoms and ruminative thoughts. Typical healthy individuals ($n=30$) also reported less depressive symptoms at follow-up. Behavioral and event-related potential indices of cognitive control were collected at baseline and follow-up during a modified flanker task. Following MAP training, N2 and P3 component amplitudes increased relative to baseline, especially among individuals with MDD. These data indicate enhanced neural responses during the detection and resolution of conflicting stimuli. Although previous research has supported the individual beneficial effects of aerobic exercise and meditation for depression, these findings indicate that a combination of the two may be particularly effective in increasing cognitive control processes and decreasing ruminative thought patterns.

Translational Psychiatry (2016) **6,** e726; doi:10.1038/tp.2015.225; published online 2 February 2016

INTRODUCTION

Thousands of new neurons are produced each day in a ‘normal’ healthy brain.[1,2] Many of these cells are produced in the hippocampus, a brain region necessary for various types of learning. These cells are produced in a part of the hippocampus known as the dentate gyrus, whose primary neuronal phenotype is the granule neuron. In animal models, newly generated neurons in the dentate gyrus are especially responsive to environmental conditions that humans often experience. For example, stressful life events tend to decrease neurogenesis, whereas antidepressants can increase cell production.[3–5] These findings, among others, led to a neurogenesis hypothesis of depression, which proposes that depression is accompanied by a loss of new granule neurons, while a renewal of these same cells can reverse depressive symptomatology.[4–6] Antidepressants are not the only depression-related therapies known to increase neurogenesis. Most notably, aerobic exercise can greatly increase the number of cells that are produced in the hippocampus. Animals given the opportunity to run on a daily basis can produce nearly twice as many new cells as sedentary controls.[7–9] Importantly, however, these new neurons are not necessarily permanent. Even under ‘healthy’ conditions, many of these new cells can die within several weeks of being born, often before differentiating into mature neurons.[10] Nevertheless, many of the newly born neurons can be rescued from death by new learning experiences and they may even be involved in learning itself.[11,12]

Not all types of learning keep new neurons alive. Rather the learning experiences must be effortful—requiring more trials of practice to learn.[13–15] These tasks bear some resemblance to effortful mental training in humans.[16,17] Accordingly, mental training can rescue new neurons from death as long as the learning experience is new and effortful. Collectively, these findings suggest that aerobic exercise increases the production of new neurons in the adult brain, while effortful mental training experiences keep a significant number of those cells alive.[7] On the basis of these neurogenic mechanisms, we developed a novel neurobehavioral intervention, referred to as mental and physical (MAP) training. Focused-attention (FA) meditation was selected as the mental training component because it requires significant mental effort to perform and each session of practice represents a new learning opportunity.[18,19] The physical training component consists of aerobic exercise, which has been shown to be beneficial for brain structure and function as well as for promoting overall physical and mental health.[20,21] Theoretically, the combination of aerobic exercise and FA meditation may increase the number of newborn cells in the hippocampus and rescue these newly generated cells, which may be integrated into the brain circuitry.

In the present study, we tested the efficacy of MAP training in improving symptoms of depression and rumination in individuals with major depressive disorder (MDD). Depression is a debilitating disorder that affects nearly one in five Americans in their lifetime, many experiencing their first episodes as adolescents and young adults. The inability to focus, concentrate or make decisions is a hallmark symptom of depression.[22,23] This clinical presentation is indicative of deficits in cognitive control, a broad term that

[1]Department of Exercise Science, Rutgers, The State University of New Jersey, New Brunswick, NJ, USA; [2]Department of Nutritional Sciences, Rutgers University, New Brunswick, NJ, USA and [3]Behavioral and Systems Neuroscience Graduate Program, Department of Psychology, Center for Collaborative Neuroscience, Rutgers University, Piscataway, NJ, USA. Correspondence: Professor BL Alderman, Department of Exercise Science, Rutgers, The State University of New Jersey, 70 Lipman Drive, New Brunswick, NJ 08901, USA. E-mail: alderman@rutgers.edu
Received 5 October 2015; revised 1 December 2015; accepted 5 December 2015

encompasses core cognitive processes of planning, inhibition (including self-control), problem solving and mental flexibility.[24] Because cognitive control processes are important for neurocognitive and affective functioning, this impairment may contribute to other symptoms in depression, particularly rumination.[23,25] Indeed, rumination may be a key cognitive and affective process in depression, wherein individuals retrieve and repetitively rehearse autobiographical memories about past and current problems. This passive and perseverative process likely disrupts the ability to concentrate, learn and engage in healthy decision-making processes.[26,27]

A number of studies report that hippocampal volume is reduced in individuals with MDD,[28,29] although this finding is not consistent enough to be a biomarker. Nonetheless, we do know that the acquisition and short-term retention of autobiographical memories activate the hippocampus and depend on neuronal activity within the hippocampus. Therefore, we hypothesized that MAP training, because it is designed to maximally activate hippocampal neurons, would be especially effective in reducing rumination. We further propose that a decrease in rumination will be reflected by an increase in synchronized neural activity in cortical regions linked to cognitive control since previous research suggests important and possibly reciprocal relationships between cognitive control impairments and rumination, which may be related to increased vulnerability for depression.[30] Thus, we hypothesized that MAP training would not only reduce ruminative thoughts, but would also ameliorate deficits in cognitive control processes. To assess this change, event-related brain potentials (ERPs) were recorded during a cognitive control task that involves conflict monitoring and inhibitory control (that is, a modified Eriksen flanker task).

It is widely accepted that either aerobic exercise or FA meditation alone can reduce symptoms of depression in individuals with MDD.[31,32] However, to date no one has directly assessed the combined effects of these two behavioral interventions. Therefore, the aim of this study was to examine the efficacy of a combined 8-week MAP training intervention on depressive symptoms, ruminative thought patterns and cognitive control processes in individuals with and without a clinical diagnosis of MDD. It was hypothesized that before MAP training, individuals with MDD would report greater rumination levels and display reduced conflict-related neural activity relative to typical healthy individuals, but these deficits would be remediated following the intervention.

MATERIALS AND METHODS

Participants

Men and women with a diagnosis of nonpsychotic MDD were recruited from a university counseling and psychiatric services clinic. Participants in the MDD group were included if they met diagnostic (DSM-IV-TR) criteria for current MDD,[33] confirmed by the Mini International Neuropsychiatric Interview (MINI[34]). The MINI is an appropriate tool for assigning threshold-level psychiatric diagnoses in research settings and has strong diagnostic agreement with other clinical interviews such as the Structured Clinical Interview for the DSM.[35] Exclusion criteria included bipolar or psychotic disorders, self-injurious or suicidal behavior, or a history of neurological disorders or head injuries resulting in a loss of consciousness. Healthy comparison participants who did not meet criteria for MDD via the MINI were also recruited and included if they reported no previous or current history of neuropsychiatric disorders, neurological disorders or head injuries. In addition, handedness (Edinburgh Handedness Inventory) was assessed to minimize heterogeneity in neurocognitive measures, but was not used as an exclusionary factor. All eligible individuals were invited to visit the laboratory for a clinical interview and neurophysiological testing at baseline before beginning the MAP training intervention. Sixty-nine participants (33 depressed, 36 nondepressed) who met the inclusion criteria were initially enrolled in the study,[30] and 22 depressed and 30 nondepressed individuals completed the 8-week MAP training intervention with complete baseline and post-intervention data (~75% retention rate). No significant differences were observed in the demographic, behavioral or cognitive status between those who finished the MAP intervention and those who did not. Before participation in this study, participants provided written informed consent that was approved by the Institutional Review Board at Rutgers, The State University of New Jersey.

Recruited participants were scheduled for study entry and baseline assessments by trained clinical research staff. Potential participants were given contact information, including a phone number and email address for the study staff/coordinator, who provided study information and scheduled an initial screening to determine eligibility. After determining initial eligibility, the study coordinator scheduled a secondary screening appointment for the informed consent process and baseline assessments, during which they completed a structured diagnostic interview, neurocognitive assessment and a maximal aerobic fitness test (VO_2 peak). Subsequently, participants were assigned to the 8-week MAP training intervention. At the end of the 8-week program, all the participants were invited back to the laboratory to complete the same battery of assessments, including measures of clinical symptoms, neurocognitive function and aerobic fitness (VO_2 peak) following the same protocol.

MAP training intervention

MAP training is a neurobehavioral intervention developed from basic neuroscientific studies suggesting that MAP training may work synergistically to improve cognitive and brain health.[17,36] For the mental component, participants engaged in FA meditation, which is challenging to learn and practice.[37,38] During FA meditation, participants sat in silence in a cross-legged or otherwise comfortable upright posture. For the meditation practice, participants were instructed to bring their full focus of attention to the breath and to count each breath if that helped to maintain focus on the breath. If their attention drifted to thoughts about the past or future, participants were instructed to acknowledge this change and return their attention to the breath. With practice, one recognizes the transient nature of thoughts and learns to monitor and accept moment-to-moment changes in attention.[39] After 20 min of sitting meditation, participants engaged in 10 min of slow-walking meditation, this time focusing their attention on their feet as they transitioned from one foot to the other in a slow walk with other participants. The 10 min walking portion of the meditation session was not only a secondary form of FA meditation training, but also a chance to return blood flow to the extremities before the upcoming session of aerobic exercise. Immediately following the meditation session, participants prepared for the exercise component.

For the physical component, participants performed aerobic exercise at a moderate intensity for 30 min. Following a 5-min warm-up, participants exercised either on a treadmill or cycle ergometer at a heart rate (HR) intensity range corresponding to 50–70% VO_2 peak as determined by their individual baseline fitness assessment before MAP training. Trained research staff supervised all exercise sessions and monitored intensity by assessing HR throughout exercise. This dose of exercise (that is, intensity and duration) is consistent with public health recommendations and is known to reduce depressive symptoms among individuals with MDD.[40,41] After 30 min of aerobic exercise, participants cooled down for ~5 min.

MDD diagnosis and assessment of depression symptom severity

At baseline, the Mini Neuropsychiatric Diagnostic Interview (MINI; manic/hypomanic episodes, obsessive-compulsive disorder, substance and alcohol use disorders) was used to confirm clinical diagnosis of MDD. The MINI is a brief structured interview that has been used extensively to aid in making diagnoses of Diagnostic and Statistical Manual of Mental Disorders, Fourth Edition (DSM-IV) and International Classification of Diseases-10 (ICD-10) psychiatric disorders. The reliability and validity of this instrument has been previously established.[34,42,43]

Depressive symptoms

The Beck Depression Inventory-II (BDI-II[44]) is a 21-item, self-report inventory of the severity of current depressive symptoms. The BDI was used to assess the severity of depressive symptoms at baseline and at post-intervention. Higher total scores reflect greater subjectively perceived depressive symptomatology. The BDI-II in this sample demonstrated good internal consistency ($\alpha = 0.92$) at baseline and the point biserial correlation coefficient of MDD diagnosis with BDI-II scores was 0.80, $P < 0.01$.

Ruminative thought patterns

Participants completed the Ruminative Responses Scale (RRS[45]) before and after MAP training. The RRS includes 22 items describing thoughts and responses that are focused on the self, the symptoms and the potential consequences and causes of their depressed mood. In addition, the scale is further defined by three subscales, which include depression, brooding and reflection.[46] Participants were asked to indicate how often they exhibit a certain behavior on a scale from 1 (almost never) to 4 (almost always) when they feel down, sad or depressed. The RRS scale demonstrated appropriate internal consistency ($\alpha = 0.93$).

Cardiorespiratory fitness and physical activity assessment

Cardiorespiratory fitness (VO_2 peak) was assessed by a maximal oxygen consumption test using a motor-driven treadmill and a modified Bruce protocol.[47] During this test, participants began walking on a treadmill, which increased in speed and incline every 2 min until volitional exhaustion or VO_2 peak criteria were met. A Polar HR monitor (Polar Electro, Kempele, Finland) was used to measure HR throughout the test, and a rating of perceived exertion[48] was taken 1 min into each stage. Rating of perceived exertion allows participants to rate their perceived physical effort on a numerical scale ranging from 6 to 20, which correlates with HR during exercise.[49] Relative VO_2 peak (ml kg^{-1} min^{-1}) was determined from direct expired gas exchange data from an indirect calorimetry metabolic system and was established as the maximal average oxygen consumption when at least three of the following criteria were met: (1) a plateau in VO_2 values despite a progressive increase in workload, (2) a maximal HR within 10 beats per minute (b.p.m.) of age-predicted maximal values (220 b.p.m. minus age in years), (3) a respiratory exchange ratio greater than 1.10 or (4) a rating of perceived exertion greater than or equal to 17. Oxygen consumption was measured through indirect calorimetry using a ParvoMedics True Max 2400 Metabolic Measurement Cart (ParvoMedics, Sandy, UT, USA) and was averaged over 15-s intervals. A 3–5 min cool-down was then performed at 2.5 m.p.h. and 0% grade to ensure participants returned to near baseline cardiovascular values. HR was recorded during the fitness assessment and was used to define individual training zones during the intervention. Fitness was reassessed upon completion of the 8-week intervention using the same procedures.

In addition, participants were also asked to recall the amount of physical activity that they engaged in at baseline and following MAP training using the International Physical Activity Questionnaire.[50] The International Physical Activity Questionnaire-short-form assesses the frequency and duration of moderate and vigorous intensity activity and walking physical activity. These data were summarized to report physical activity by weighting the energy expenditure for these categories of activity to produce MET-minutes of physical activity.

Cognitive control

A modified arrow version of the Eriksen flanker task[30,51] was presented using E-Prime version 2.0 software (Psychology Software Tools, Pittsburgh, PA, USA). Participants completed the flanker task at baseline and post-intervention to assess changes in behavioral and neurophysiological cognitive control processes. The flanker task is composed of two conditions, congruent and incongruent, during which participants are asked to press either a left or right button corresponding to the direction of a centrally located target arrow. The congruent trials consisted of the central target being flanked by arrows pointing in the same direction (for example, «« <), while incongruent trials involved the target being flanked by arrows pointing in the opposing direction (for example, « > «). A set of instructions preceded the first trial that explained which button press would be used to indicate the direction of the central or target arrow. Participants performed a button press with their left thumb when the target arrow pointed to the left (<) and a button press with their right thumb when the target arrow pointed to the right (>). In addition, participants were instructed to respond as quickly and accurately as possible for each trial. Each trial began with a black fixation cross (+) in the center of a white screen for 500 ms, followed by 1.5 cm tall × 8 cm long black arrows centered focally on a white background for 100 ms with a response window of 1500 ms. A random inter-stimulus time interval of 1100, 1300, or 1500 ms was used between each visual fixation (+) and the stimulus in order to reduce potential anticipatory responses. Following the instructions, participants completed 20 practice trials, including equiprobable congruent and incongruent trials. Performance feedback was provided and any remaining questions were resolved during the practice trials to ensure a sufficient familiarization and understanding of the task. After the practice, the participants completed two blocks of 110 trials with equiprobable congruency and directionality of the stimuli. The stimuli were presented on a monitor at a distance of 70 cm centered to the nasion and the vertical and horizontal visual angles were 1.2° and 6.6°, respectively. In addition to behavioral measures of accuracy and reaction time, continuous electroencephalographic data were collected during the flanker task to derive N2 and P3 component amplitudes.

Event-related potentials

Continuous electroencephalographic activity was recorded from 64 scalp sites using a HydroCel Geodesic Sensor Net and Electrical Geodesics (Eugene, OR, USA) amplifier system (20 K nominal gain, bandpass = 0.1–100 Hz) and arranged according to the International 10-10 system.[52] The electro-oculogram was recorded from electrodes placed above and below each eye. Continuous data were initially referenced to the vertex electrode (Cz) and digitized continuously at 250 Hz with a 24-bit analog-to-digital converter. Impedances were maintained below 50 kΩ. Although lower impedances are typically recommended, previous research has produced acceptable electroencephalographic signals when data were collected with higher scalp impedances,[53] and similar values have been used in previous studies of cognitive control and depression.[54,55]

Following collection, data were re-referenced to the average of the left and right mastoids[56,57] and bandpass filtered with a low-pass frequency of 30 Hz and high-pass frequency of 0.1 Hz. The continuous electroencephalographic data were manually inspected and periods with large movement-related artifacts (eye blinks, eye movement and muscle activity) were removed using NetStation 4.0 (Electrical Geodesic). Stimulus-locked epochs were created from 100 ms pre-stimulus to 1000 ms post stimulus and baseline-adjusted using the 100 ms pre-stimulus period. NetStation detection software, which allows for the adjustment of settings for detecting and marking artifacts and contaminated segments, was used to detect eye blinks, vertical and horizontal eye movements, and bad channels. Marked segments were visually inspected and rejected if they contained (1) eye movements exceeding 55 μV, (2) eye blinks exceeding 14 μV or (3) greater than or equal to 10 bad channels exceeding 200 μV. In each case, a moving average of 20 samples combined with threshold values was used. Using spherical spline interpolation, bad channels were then replaced from the remaining channels in 'good' segments. Trials were also visually inspected for remaining artifacts, and data from individual channels containing artifacts were rejected on a trial-by-trial basis. Only correct trials were used for the corresponding ERP analyses. Using a mean amplitude approach,[58] the N2 component was defined as the mean amplitude within a 200–350 ms window post-stimulus onset,[30,59] whereas the P3 component was defined as the mean amplitude within a 250–500 ms window post-stimulus onset.[60]

Data analysis

Descriptive statistics were first performed on participant demographic and fitness data. Using the G*Power 3 program, with a Cohen's $d = 0.56$, significance level $\alpha = 0.05$, required power $(1 - \beta) = 0.08$, and number of groups = 2, the sample of 22 depressed and 30 nondepressed individuals who completed the 8-week MAP training intervention resulted in sufficient power for this study. Before conducting analyses, cognitive outcome measures were assessed for normality. To test the effects of MAP training intervention on depressive symptoms, rumination and aerobic fitness, we used repeated-measures analyses of variance (RM ANOVAs) with group status (MDD or healthy) as a between-subjects variable and time (pre- and post-intervention) as a within-subjects variable. For behavioral performance (accuracy and reaction time) and ERP measures (N2 and P3), we used three-way RM ANOVAs with group status as a between-subjects variable and time (pre- and post-intervention) and congruency (congruent and incongruent) serving as within-subjects variables. To reduce the potential effect of outliers, trials with RTs beyond the individual mean ± 3 s.d. for each trial type were excluded. Statistical analyses for the N2 and P3 ERP components were performed using five electrode sites across the midline (N2: Fz, FCz and Cz; P3: Cz, CPz and Pz). The anterior N2 is most robust and frequently examined at frontocentral midline electrode sites,[59,61] while the posterior P3 component is most prominent and commonly studied at centroparietal midline electrode sites.[62,63] Thus, Fz, FCz and Cz electrodes were used for N2 analyses and Cz, CPz and Pz electrode sites for P3 analyses. All the tests were conducted as two-tailed and the family-wise alpha level of probability was set at $P < 0.05$ before

Table 1. Demographic and fitness characteristics of participants by group status at baseline

Characteristic	*Healthy*	*Depressed*	*Total*
n	30	22	52
Age (years)	21.2 ± 3.3	20.7 ± 3.1	21.0 ± 3.2
Gender (male/female)	10/20	5/17	15/37
Height (cm)	168.0 ± 7.9	163.5 ± 10.3	166.1 ± 9.2
Weight (kg)	66.0 ± 13.2	63.6 ± 12.6	65.0 ± 12.9
BMI (kg/m^2)	23.3 ± 4.2	23.7 ± 3.4	23.5 ± 3.9
VO_2 peak (ml kg^{-1} min^{-1})	38.6 ± 9.2	37.2 ± 7.1	38.0 ± 8.3

Abbreviations: BMI, body mass index; VO_2 peak, peak aerobic fitness. Values equal means ± s.d.

Bonferroni correction and adjusted when appropriate with the Greenhouse–Geisser epsilon correction for non-sphericity.[64] Planned comparisons and *post hoc* analyses were conducted using Bonferroni correction for multiple comparisons. Last, bivariate correlations were conducted to examine the relationship between change scores in ERP component amplitudes and self-reported rumination. Partial eta squared (η_p^2) values are presented as effect sizes.

RESULTS

Consistent with higher incidence of MDD among women,[65,66] more women with MDD volunteered to participate and enrolled in the current study. Overall, self-reported symptoms of depression on the BDI were significantly higher for women than for men, F(1,48) = 6.67, $P < 0.01$. However, we did not detect sex differences in behavioral task performance measures (accuracy and response time) or ERP component amplitudes. Therefore, task performance and ERP data were collapsed across sex for subsequent analyses. As expected, men had higher VO_2 peak values than women (47.3 ± 6.4 ml kg^{-1} min^{-1} vs 34.2 ± 5.6 ml kg^{-1} min^{-1}). Depressed and healthy groups did not differ significantly on any demographic or fitness measures before MAP Training (*P*-values > 0.19; see Table 1 for demographic and fitness data). Meta-analytic findings report moderate-sized effects for the treatment of depression with exercise.

BDI-II analysis—depressive symptoms

Participants with a clinical diagnosis of MDD reported higher levels of depressive symptoms before MAP training, F(1,50) = 79.20; $P < 0.001$, $\eta_p^2 = 0.61$. The two-way RM ANOVA on BDI-scores also revealed a significant main effect of time (pre-post), F(1,50) = 58.8, $P < 0.001$, $\eta_p^2 = 0.54$, indicating that both groups reported significantly lower symptoms of depression following the 8-week intervention. However, these main effects were superseded by a significant group × time interaction, F(1,50) = 18.73, $P < 0.001$, $\eta_p^2 = 0.27$, such that the decrease in depressive symptoms was significantly greater for the depressed group than for the healthy control group, see Figure 1.

Ruminative responses scale

The RM ANOVA for overall rumination (RRS) scores revealed a significant main effect of group, F(1,50) = 31.6, $P < 0.001$, $\eta_p^2 = 0.39$, such that the MDD group ($M = 55.8 \pm 2.0$) reported significantly greater self-reported rumination relative to the healthy comparison group, ($M = 41.0 \pm 1.6$; see Figure 2) at baseline, F(1,48) = 19.89, $P < 0.001$. A significant main effect of time was observed, F(1,50) = 16.6, $P < 0.001$, $\eta_p^2 = 0.25$, indicating that both the groups demonstrated a significant pre-to-post intervention decrease in ruminative thought patterns. No significant interaction between group and time on rumination was found, $P > 0.05$.

Cardiorespiratory fitness—VO_2 peak

As expected, males had higher VO_2 peak values than females, F(1,50) = 52.78, $P < 0.001$. A two-way RM ANOVA was conducted to determine whether changes in aerobic fitness occurred following the 8-week MAP intervention. No significant main effect of group emerged, indicating no between-group differences in aerobic fitness. In addition, MAP training was not associated with an increase in aerobic fitness levels, $P > 0.05$. With respect to changes in physical activity, however, participants reported an overall increase in daily physical activity following MAP training, F(1,50) = 4.29, $P < 0.05$, $\eta_p^2 = 0.19$, above the amount of physical activity required during the intervention. These data suggest that participation in the MAP training intervention was insufficient to alter fitness levels, but resulted in an increase in daily physical activity.

Behavioral performance data

For accuracy on the flanker task, the mixed 2 (group) × 2 (task congruency) ANOVA revealed a main effect of congruency, F(1,50) = 29.28, $P < 0.001$, $\eta_p^2 = 0.37$, indicating worse performance on incongruent relative to congruent trials. No main effect of group or group by congruency interaction was observed, indicating that MDD and healthy controls did not differ in terms of overall performance accuracy. However, this analysis also confirmed that a comparable number of data points were available in each group for subsequent ERP analysis. The RM ANOVA for RT similarly showed a main effect of congruency, F (1,50) = 391.72, $P < 0.001$, $\eta_p^2 = 0.89$, owing to faster response times for congruent vs incongruent trials. Similar to accuracy, the main effect of group and the group × congruency interaction were not statistically significant. Overall, there were minimal effects of depression status, sex and MAP training on behavioral task performance measures (Table 2).

ERP component amplitudes

In a previous study,[30] we reported that the N2 component of the ERP elicited by the flanker task is reduced in individuals with MDD. Many of these same individuals ($n = 50$) participated in the current intervention, and the N2 and P3 component amplitude responses were thus re-analyzed here with MAP training serving as a repeated-measures variable (pre-to-post intervention). The mixed model three-way ANOVA for N2 averaged across Fz, FCz and Cz electrode sites revealed a significant time main effect, F(1,50) = 48.53, $P < 0.001$, $\eta_p^2 = 0.49$, with significantly larger (that is, more negative) N2 values occurring following MAP training relative to values at baseline, $P < 0.001$. A significant congruency effect was also observed, F(1,50) = 28.45, $P < 0.001$, $\eta_p^2 = 0.36$, such that more negative N2 values were observed during the more challenging incongruent task trials. The significant main effect of MAP training was superseded by a significant time × group interaction, F(1,50) = 34.39, $P < 0.001$, $\eta_p^2 = 0.41$. Decomposition of this interaction revealed that the depressed group had a larger increase in N2 amplitude from pre- (0.87 ± .55 μV) to post intervention (−0.68 ± 0.43 μV) relative to their nondepressed counterparts (−0.62 ± 0.47 μV, − 0.76 ± 0.37 μV), see Figure 2. No other significant main effects or interactions were found for N2 component amplitude.

The RM ANOVA for P3 component amplitude revealed a significant congruency main effect, F(1,50) = 131.52, $P < 0.001$, $\eta_p^2 = 0.73$, with larger component amplitudes found for incongruent relative to congruent task trials. This main effect was superseded by a significant congruency by group interaction, F(1,50) = 5.41, $P < 0.05$, $\eta_p^2 = 0.10$, and a congruency × time interaction, F(1,50) = 13.59, $P < 0.001$, $\eta_p^2 = 0.21$. There was also a significant three-way interaction of time × congruency × group status, F(1,50) = 9.28, $P < 0.01$, $\eta_p^2 = 0.16$. Although depressed individuals exhibited a nonsignificant trend towards smaller P3

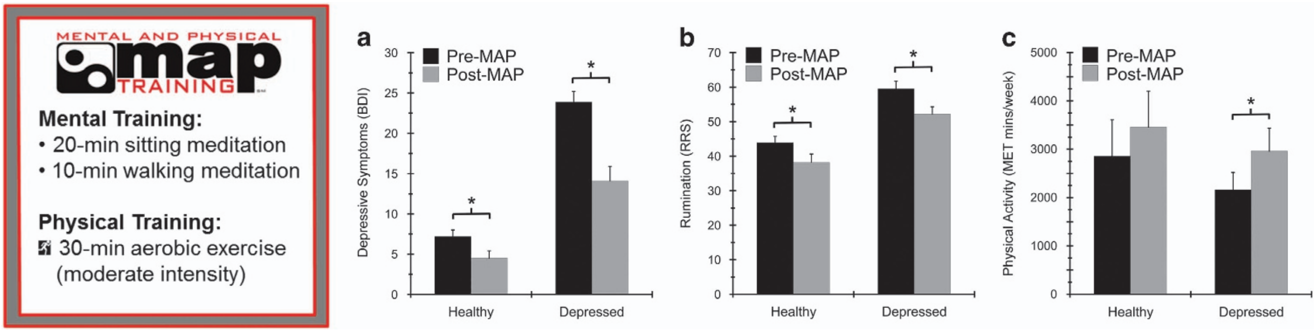

Figure 1. (**a**) After MAP training, there were significant decreases in the severity of depression (BDI-II) in individuals with MDD and healthy controls. (**b**) Scores on the ruminative responses scale (RRS) were significantly decreased at post-intervention for both groups. (**c**) The International Physical Activity Questionnaire (IPAQ) was used to assess physical activity levels in both groups. At post-intervention, both groups reported increases in physical activity, although the increase was only significant for the depressed group. *Indicates a significant difference from pre-to-post intervention. BDI, Beck Depression Inventory; MAP, mental and physical; MDD, major depressive disorder.

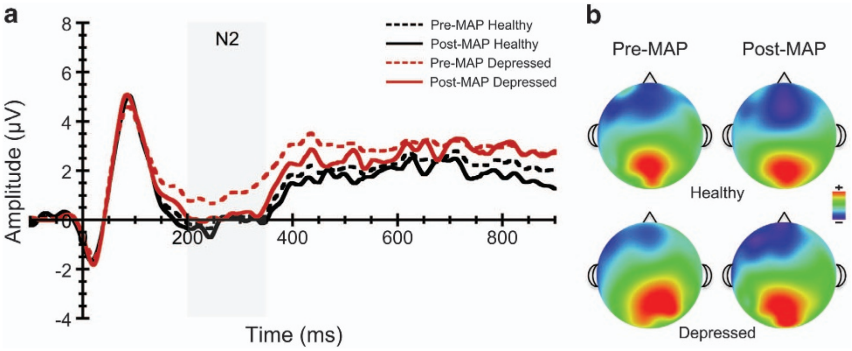

Figure 2. Stimulus-locked grand average N2 ERP waveforms (**a**) and topographic plots (**b**) averaged across flanker task conditions before and after MAP training. ERPs were averaged across frontocentral midline electrode sites (Fz, FCz and Cz). ERP, event-related potential; MAP, mental and physical.

Table 2. Mean (95% CI) values for psychological and behavioral task performance measures before and after MAP training

	Healthy (n = 30)		*Depressed* (n = 22)	
	Pre-MAP	*Post-MAP*	*Pre-MAP*	*Post-MAP*
Response accuracy (%)				
Congruent trials	97.1 (94.8–99.5)	98.0 (97.0–99.1)	96.8 (92.8–100.8)	98.5 (96.6–100.3)
Incongruent trials	85.9 (79.3–95.1)	90.0 (85.0–95.1)	86.1 (76.2–96.1)	89.2 (81.8–96.6)
Reaction time (ms)				
Congruent trials	344.4 (318.7–370.1)	330.0 (310.0–350.1)	319.5 (290.5–348.4)	301.2 (280.8–321.5)
Incongruent trials	422.2 (393.6–450.8)	407.1 (384.3–430.0)	390.0 (356.2–423.8)	373.0 (347.0–399.0)

Abbreviations: CI, confidence interval; MAP, mental and physical.

amplitude at baseline relative to the nondepressed group, (5.82 vs 6.28 μV), they had a significantly larger increase in P3 amplitude to incongruent trials from pre-to-post intervention compared with typical healthy undergraduates, $P<0.01$, see Figure 3. As expected, these data suggest that depressed participants displayed enhanced neuronal activity during the conflict monitoring task following the intervention. Further, this increase was observed during task trials that require the greatest amount of conflict and cognitive control.

Correlational analysis

Last, to assess whether the change in ruminative thought patterns was related to enhanced neural responses during conflict monitoring, bivariate correlations were conducted between change scores in ERP component amplitudes and rumination scores from pre-to-post MAP training. The change score in N2 amplitude was significantly correlated with RRS change scores, $r = 0.30$, $P<0.05$, see Figure 4. Thus, decreased rumination at post-intervention was associated with larger N2 amplitudes, suggesting a possible neurophysiological mechanism linking cognitive control processes with ruminative thoughts, and how these mechanisms might be strengthened across the 8-week intervention period.

DISCUSSION

It is widely accepted that aerobic exercise and meditation training are useful behavioral therapies for remediating clinical symptoms

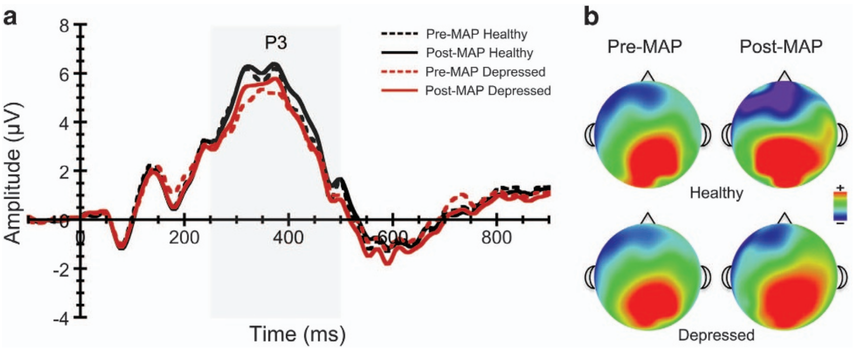

Figure 3. Stimulus-locked grand average P3 ERP waveforms (**a**) and topographic plots (**b**) averaged across flanker task conditions before and after MAP training. ERPs were averaged across centroparietal midline electrode sites (Cz, CPz and Pz). ERP, event-related potential; MAP, mental and physical.

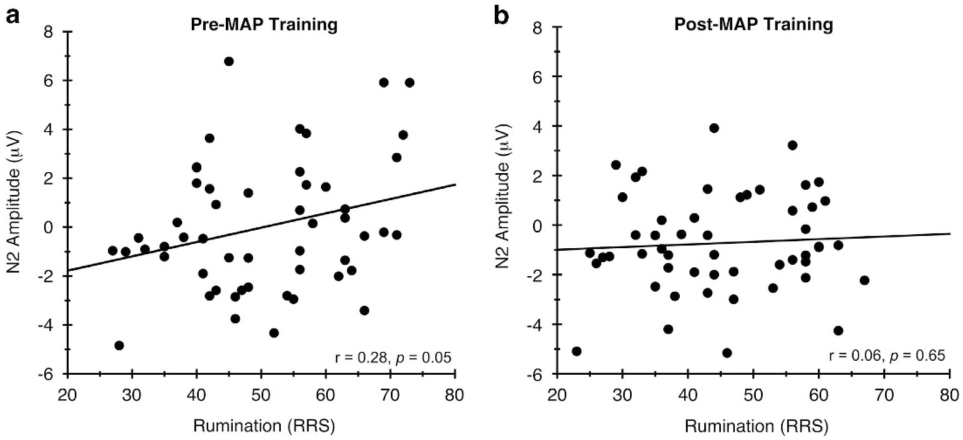

Figure 4. Correlational analysis between pre-to-post intervention change in ruminative thoughts and N2 amplitude. At baseline (**a**), RRS scores were significantly correlated with N2 amplitude. Following the intervention (**b**), this relationship was no longer significant. MAP, mental and physical; RRS, ruminative responses scale.

of depression.[31,32] However, no study to date has assessed the combined effects of the two behavioral interventions. Here, we present data indicating that a combination of FA meditation and moderate-intensity aerobic exercise significantly reduces symptoms of depression in individuals with MDD. The effects were robust, as evident by the nearly 40% reduction in depressive symptoms after only 8 weeks of training. Interestingly, individuals without a clinical diagnosis of MDD also reported significant reductions in depressive symptoms. Participants with MDD also exhibited a significant decrease in self-reported ruminative thoughts, which typically involve repetitive thinking about autobiographical memories and negatively valenced content from the past. Along with these positive changes in psychological outcomes, significant increases in synchronized neural activity were found following MAP training. In particular, N2 amplitude was lower in the depressed group at baseline and N2 amplitudes increased at post-intervention in both depressed and healthy groups. For the P3 component, an increase in amplitude was observed for the depressed group following the intervention for task trials that result in the greatest amount of conflict. This resulted in an enhancement of P3 in the depressed group that approached those observed in the healthy comparison group at post-intervention. These ERPs have previously been used to reflect neural activity during conflict monitoring and cognitive control.[67] Importantly, we failed to detect differences in behavioral task performance measures between depressed and healthy individuals at baseline or following MAP training. Therefore, these MAP-induced changes in ERPs reflect an overall increase in the synchronous neuronal responses during a task that requires an upregulation of cognitive control during conflict monitoring. Moreover, ERPs may be more sensitive to a neurobehavioral intervention among young adults with and without MDD than more traditional overt behavioral performance measures alone. In our study, individuals with MDD initially exhibited impairments in both N2 and P3 amplitudes relative to healthy individuals. Therefore, the increase in component amplitudes reflects a return to neurophysiological levels more consistent with their otherwise healthy counterparts. From these results, we propose that the MAP training intervention reduces rumination, which is associated with enhanced neural representations of conflict monitoring and cognitive control, particularly among individuals with MDD.

Previous evidence suggests that regular aerobic exercise improves mental health and neurocognitive function, including processes related to learning and memory. The evidence for mental health benefits, although impressive for healthy individuals, is even stronger for clinical and psychiatric populations.[40,68–70] Indeed, randomized controlled trials indicate that aerobic exercise is as effective as common antidepressants (for example, selective serotonin reuptake inhibitors and monoamine oxidase inhibitors) in reducing depression,[69,71] as well as in reducing relapse rates.[68] With respect to mechanisms, there are many potential candidates. One involves the autonomic nervous system, which regulates cardiovascular and stress responses, and has been implicated in various lifestyle choices and health risk behaviors.[72–74] For example, we recently reported that aerobic fitness positively relates to neurocardiac balance (an index of autonomic function);

individuals with higher cardiorespiratory fitness (VO_2 peak) displayed greater neurocardiac balance between sympathetic and parasympathetic branches of the autonomic nervous system.[75] A second candidate mechanism is neurogenesis, specifically the production of new neurons in the hippocampal formation of the adult brain.[6,76,77] A number of studies have reported that exercise in laboratory animals increases neurogenesis and exposure to stressful conditions that mimic stressful life conditions in humans reduce cell production. Because individuals with MDD often exhibit reduced hippocampal volumes,[28] some have speculated that aerobic exercise alleviates depression through an increase in cell proliferation.[78] However, it is not yet possible to monitor neurogenesis in humans and therefore, these theories remain premature.

Similar to aerobic exercise, the practice of meditation has garnered increasing attention as an effective therapy for reducing symptoms of depression.[79] Mindfulness meditation, the practice of attending to the present moment and allowing thoughts and emotions to pass without judgment, has received the most attention as an effective therapy for reducing symptoms of depression.[32,80–82] One variant of mindfulness-based practice is FA meditation, which is an effortful learning process during which an individual maintains focus on a selected object (for example, the breath) and when their focus becomes distracted, they learn to disengage attention from the source of distraction, and redirect and engage their attention to the intended object.[19] With practice, the person learns to recognize deviations of attention, thereby acquiring new skills that can help direct attention to the present moment, not only during meditation but also in everyday life. FA meditation is often used as a stepping-stone to other practices, as well as an end in itself.[83] As with most mindfulness-based practices, FA meditation is associated with clarity of thoughts, recognition of feelings, the ability to control anger[84] and an improved overall sense of well-being and positive emotion.[84–86] Mechanistically, FA meditation induces significant changes in autonomic nervous system regulation such as heart rate and respiration[87] together with central autonomic network control.[88] Studies have also started to document the potential brain regions and substrates influenced by meditation training. Oft-cited neural structures include the hippocampus and cortical regions, including prefrontal and anterior cingulate regions, which are most often associated with change. For example, a recent study reported that just 8 weeks of mindfulness-based meditation increased the volume of the hippocampus.[89] FA on the breath has also been found to recruit an attention network that includes parietal and prefrontal structures.[90] We propose that meditation practice coupled with aerobic exercise maximally increases the integration of networks involved with cognitive control and learning to those necessary for autonomic nervous system regulation. This theory is generally consistent with others that address the mediating factors that disrupt cognitive and affective processes during depression.[28,91–93]

Previously, we tested the efficacy of MAP training with women in the local community who are young mothers and were recently homeless on the streets of New Jersey. They had recently been accepted to live full time at a residential treatment center, where we provided the intervention. The female participants ($n = 8$) began the study with severe depressive symptoms, as well as elevated anxiety levels indexed by Beck Anxiety Inventory. After 8 weeks of MAP training, BDI and Beck Anxiety Inventory scores dropped nearly in half. Importantly, the young women also demonstrated significant improvements in their physical health profile. Before MAP training, their aerobic fitness levels (VO_2 peak) were in the low range. After MAP training, fitness levels increased into the normal-to-excellent range.[47] In the current study, we did not observe an increase in fitness levels, perhaps because the sample was composed largely of university undergraduate students, most of whom were in relatively in good physical condition before committing to the study. However, participants in this study reported an increase in physical activity engagement following the intervention. This is important because it may indicate that the participants were either more motivated to exercise or were taking advantage of opportunities to be physically active as they realized the positive benefits and opportunities following MAP training. Notably, reductions in symptoms of depression and enhanced neural responses occurred despite a change in aerobic capacity, suggesting other potential candidates as mechanisms that may underlie the beneficial effects of combining FA meditation with aerobic exercise.

How does the combination of meditation and aerobic exercise meaningfully decrease depression and/or depressive symptoms? As previously discussed, the MAP training intervention was translated from basic neuroscientific research demonstrating that aerobic exercise and mental training increase neurogenesis in the adult hippocampus.[11,17,36] Indeed, these studies were the first to report that new neurons in the hippocampus may be involved in the acquisition and short-term recall of associative memories.[12] In those initial studies, an effortful learning task was used because of its dependence on the hippocampus for learning and its association with declarative memories.[94–97] In humans, tasks such as these are often associated with the acquisition of autobiographical memories. Although the hippocampus is 'activated' during the acquisition and retrieval of autobiographical and/or episodic memories, it is only necessary for learning new episodic memories and recollecting recent memories.[95] In other words, the hippocampus is generally not necessary for the long-term storage of memories. These findings are consistent with our hypothesis that rumination preferentially activates the hippocampus because ruminative thoughts often reflect feelings and/or thoughts about autobiographical events in the present or recent past. Of course, the hippocampus does not act alone in these learning processes, but rather interacts directly and indirectly with prefrontal and cingulate cortical regions.[98,99] Indeed, for many types of learning, interactions among hippocampal and prefrontal cortices are necessary to associate memories from the past with the present context.[100] These anatomical and neurophysiological interactions are likewise necessary for imagining future scenarios and making adaptive decisions when faced with challenging life situations.[101] When individuals are clinically depressed and have difficulties inhibiting or filtering out ruminative thoughts, it may become challenging to efficiently 'time-travel' without becoming engaged in memory rehearsal. Therefore, we suggest that individuals with MDD who engage in excessive rumination are activating distributed neuronal circuits within the hippocampus and frontal cortices, which impairs their ability to acquire new associative memories. By learning to focus their attention, the participants acquire new cognitive skills that reduce interference from negatively biased recollections. These neuronal mechanisms activated during mental training with meditation are perhaps further strengthened and even 'consolidated' by the physical training with aerobic exercise that occurs immediately afterwards in this neurobehavioral intervention.

CONCLUSION

Until recently, the most common and accepted line of treatment for depression has been psychotropic medications, most notably the selective serotonin reuptake inhibitors and mood stabilizers. However, recent studies indicate that these drugs may not be as effective as once thought and even when they are, relapse often occurs.[102–104] Various forms of psychotherapy, such as cognitive behavioral therapy, can be efficacious but require considerable time and commitment on the part of the patient, not to mention trained professionals to institute. There are two behavioral therapies, aerobic exercise and meditation, which have demonstrated benefits for individuals suffering with depression, are not

accompanied by profound side effects and can be practiced across the lifespan. Here, we provide evidence that demonstrates the effectiveness of a combined behavioral approach in improving mental and cognitive health outcomes in individuals with MDD and otherwise healthy individuals.

CONFLICT OF INTEREST

The authors declare no conflict of interest.

ACKNOWLEDGMENTS

We gratefully acknowledge the contributions of all volunteers who participated in this study as well as the undergraduate research assistants who contributed to data collection. We also acknowledge the Rutgers University Counseling and Psychiatric Services (CAPS) program for assistance in participant recruitment as well as the Rutgers University Recreation Department. A special acknowledgment of Myong Ahn Sunim and Moo Sang Sunim from the Soshimsa Zen Center for their meditation training and the translation of MAP training into a clinically relevant intervention. We thank Michelle Chang, Marsha Bates and Edward Selby for early participation in the study. We also thank Peter Ehmann and Emma Millon for helpful comments to the manuscript. This research was supported by The Charles and Johanna Busch Memorial Fund at the Rutgers University, and the Brain and Behavior Research Foundation.

REFERENCES

1 Shors TJ. The adult brain makes new neurons and effortful learning keeps them alive. *Curr Dir Psychol Sci* 2014; **23**: 311–318.
2 Shors TJ, Olson RL, Bates ME, Selby EA, Alderman BL. Mental and physical (MAP) training: a neurogenesis-inspired intervention that combines aerobic exercise and meditation to enhance brain health in humans. *Neurobiol Learn Mem* 2014; **115**: 3–9.
3 Mirescu C, Gould E. Stress and adult neurogenesis. *Hippocampus* 2006; **16**: 233–238.
4 Warner-Schmidt JL, Duman RS. Hippocampal neurogenesis: opposing effects of stress and antidepressant treatment. *Hippocampus* 2006; **16**: 239–249.
5 Boldrini M, Hen R, Underwood MD, Rosoklija GB, Dwork AJ, Mann JJ *et al*. Hippocampal angiogenesis and progenitor cell proliferation are increased with antidepressant use in major depression. *Biol Psychiatry* 2012; **72**: 562–571.
6 Eisch AJ, Petrik D. Depression and hippocampal neurogenesis: a road to remission? *Science* 2012; **338**: 72–75.
7 Curlik DM 2nd, Maeng LY, Agarwal PR, Shors TJ. Physical skill training increases the number of surviving new cells in the adult hippocampus. *PLoS One* 2013; **8**: e55850.
8 van Praag H. Neurogenesis and exercise: past and future directions. *Neuromol Med* 2008; **10**: 128–140.
9 van Praag H, Kempermann G, Gage FH. Running increases cell proliferation and neurogenesis in the adult mouse dentate gyrus. *Nat Neurosci* 1999; **2**: 266–270.
10 Anderson ML, Sisti HM, Curlik DM 2nd, Shors TJ. Associative learning increases adult neurogenesis during a critical period. *Eur J Neurosci* 2011; **33**: 175–181.
11 Gould E, Beylin A, Tanapat P, Reeves A, Shors TJ. Learning enhances adult neurogenesis in the hippocampal formation. *Nat Neurosci* 1999; **2**: 260–265.
12 Shors TJ, Miesegaes G, Beylin A, Zhao M, Rydel T, Gould E. Neurogenesis in the adult is involved in the formation of trace memories. *Nature* 2001; **410**: 372–376.
13 Curlik DM 2nd, Shors TJ. Learning increases the survival of newborn neurons provided that learning is difficult to achieve and successful. *J Cogn Neurosci* 2011; **23**: 2159–2170.
14 Waddell J, Anderson ML, Shors TJ. Changing the rate and hippocampal dependence of trace eyeblink conditioning: Slow learning enhances survival of new neurons. *Neurobiol Learn Mem* 2011; **95**: 159–165.
15 Waddell J, Shors TJ. Neurogenesis, learning and associative strength. *Eur J Neurosci* 2008; **27**: 3020–3028.
16 Shors TJ, Anderson ML, Curlik DM 2nd, Nokia MS. Use it or lose it: how neurogenesis keeps the brain fit for learning. *Behav Brain Res* 2012; **227**: 450–488.
17 Shors TJ, Olson RL, Bates ME, Selby EA, Alderman BL. Mental and Physical (MAP) Training: a neurogenesis-inspired intervention that enhances health in humans. *Neurobiol Learn Mem* 2014; **115**: 3–9.
18 Lippelt DP, Hommel B, Colzato LS. Focused attention, open monitoring and loving kindness meditation: effects on attention, conflict monitoring, and creativity—a review. *Front Psychol* 2014; **5**: 1083.
19 Lutz A, Slagter HA, Rawlings NB, Francis AD, Greischar LL, Davidson RJ. Mental training enhances attentional stability: neural and behavioral evidence. *J Neurosci* 2009; **29**: 13418–13427.
20 Erickson KI, Voss MW, Prakash RS, Basak C, Szabo A, Chaddock L *et al*. Exercise training increases size of hippocampus and improves memory. *Proc Natl Acad Sci USA* 2011; **108**: 3017–3022.
21 Prakash RS, Voss MW, Erickson KI, Kramer AF. Physical activity and cognitive vitality. *Annu Rev Psychol* 2015; **66**: 769–797.
22 Davidson RJ, Pizzagalli D, Nitschke JB, Putnam K. Depression: perspectives from affective neuroscience. *Annu Rev Psychol* 2002; **53**: 545–574.
23 Krompinger JW, Simons RF. Cognitive inefficiency in depressive undergraduates: stroop processing and ERPs. *Biol Psychol* 2011; **86**: 239–246.
24 Botvinick MM, Braver TS, Barch DM, Carter CS, Cohen JD. Conflict monitoring and cognitive control. *Psychol Rev* 2001; **108**: 624–652.
25 Levens SM, Muhtadie L, Gotlib IH. Rumination and impaired resource allocation in depression. *J Abnorm Psychol* 2009; **118**: 757–766.
26 Disner SG, Beevers CG, Haigh EA, Beck AT. Neural mechanisms of the cognitive model of depression. *Nat Rev Neurosci* 2011; **12**: 467–477.
27 Nolen-Hoeksema S. The role of rumination in depressive disorders and mixed anxiety/depressive symptoms. *J Abnorm Psychol* 2000; **109**: 504–511.
28 Bremner JD, Narayan M, Anderson ER, Staib LH, Miller HL, Charney DS. Hippocampal volume reduction in major depression. *Am J Psychiatry* 2000; **157**: 115–118.
29 Campbell S, Marriott M, Nahmias C, MacQueen GM. Lower hippocampal volume in patients suffering from depression: a meta-analysis. *Am J Psychiatry* 2004; **161**: 598–607.
30 Alderman BL, Olson RL, Bates ME, Selby EA, Buckman JF, Brush CJ *et al*. Rumination in major depressive disorder is associated with impaired neural activation during conflict monitoring. *Front Hum Neurosci* 2015; **9**: 269.
31 Rethorst CD, Trivedi MH. Evidence-based recommendations for the prescription of exercise for major depressive disorder. *J Psychiatr Pract* 2013; **19**: 204–212.
32 Segal ZV, Williams JMG, Teasdale JD. *Mindfulness-Based Cognitive Therapy for Depression*. Guilford Press: New York, NY, USA, 2012.
33 American Psychiatric Association. *Diagnostic and Statistical Manual of Mental Disorders*. American Psychiatric Association: Washington, DC, USA, 2000.
34 Sheehan DV, Lecrubier Y, Sheehan KH, Amorim P, Janavs J, Weiller E *et al*. The Mini-International Neuropsychiatric Interview (M.I.N.I.): the development and validation of a structured diagnostic psychiatric interview for DSM-IV and ICD-10. *J Clin Psychiatry* 1998; **59**: 22–33.
35 First MB, Williams JB, Karg RS, Spitzer RL. Structured clinical interview for DSM-5 disorders (SCID-5), clinician version. American Psychiatric Association: Arlington, VA, USA, 2016.
36 Curlik DM 2nd, Shors TJ. Training your brain: do mental and physical (MAP) training enhance cognition through the process of neurogenesis in the hippocampus? *Neuropharmacology* 2013; **64**: 506–514.
37 Farb NA, Segal ZV, Mayberg H, Bean J, McKeon D, Fatima Z *et al*. Attending to the present: mindfulness meditation reveals distinct neural modes of self--reference. *Soc Cogn Affect Neurosci* 2007; **2**: 313–322.
38 Lutz A, Slagter HA, Dunne JD, Davidson RJ. Attention regulation and monitoring in meditation. *Trends Cogn Sci* 2008; **12**: 163–169.
39 Paulson S, Davidson R, Jha A, Kabat-Zinn J. Becoming conscious: the science of mindfulness. *Ann N Y Acad Sci* 2013; **1303**: 87–104.
40 Dunn AL, Trivedi MH, Kampert JB, Clark CG, Chambliss HO. Exercise treatment for depression: efficacy and dose response. *Am J Prev Med* 2005; **28**: 1–8.
41 Trivedi MH, Greer TL, Grannemann BD, Chambliss HO, Jordan AN. Exercise as an augmentation strategy for treatment of major depression. *J Psychiatr Pract* 2006; **12**: 205–213.
42 Lecrubier Y, Sheehan D, Weiller E, Amorim P, Bonora I, Harnett Sheehan K *et al*. The Mini International Neuropsychiatric Interview (MINI). A short diagnostic structured interview: reliability and validity according to the CIDI. *Eur Psychiatry* 1997; **12**: 224–231.
43 Sheehan D, Lecrubier Y, Harnett Sheehan K, Janavs J, Weiller E, Keskiner A *et al*. The validity of the Mini International Neuropsychiatric Interview (MINI) according to the SCID-P and its reliability. *Eur Psychiatry* 1997; **12**: 232–241.
44 Beck AT, Steer RA, Brown GK. *Manual for the Beck Depression Inventory-II*. Psychological Corporation: San Antonio, TX, USA, 1996.
45 Nolen-Hoeksema S, Davis CG. "Thanks for sharing that": ruminators and their social support networks. *J Pers Soc Psychol* 1999; **77**: 801–814.
46 Treynor W, Gonzalez R, Nolen-Hoeksema S. Rumination reconsidered: a psychometric analysis. *Cognitive Ther Res* 2003; **27**: 247–259.
47 American College of Sports Medicine. *ACSM's guidelines for exercise testing and prescription*, 9th edn. Wolters Kluwer Health/Lippincott Williams & Wilkins: Philadelphia, PA, USA, 2013.
48 Borg G. Perceived exertion as an indicator of somatic stress. *Scand J Rehabil Med* 1970; **2**: 92–98.
49 Borg GA. *Borg's perceived exertion and pain scales*. Human Kinetics: Champaign, IL, USA, 1998.
50 Craig CL, Marshall AL, Sjostrom M, Bauman AE, Booth ML, Ainsworth BE *et al*. International physical activity questionnaire (IPAQ): 12-country reliability and validity. *Med Sci Sports Exerc* 2003; **35**: 1381–1395.

51 Eriksen BA, Eriksen CW. Effects of noise letters upon the identification of a target letter in a nonsearch task. *Atten Percept Psychophys* 1974; **16**: 143–149.
52 Chatrian GE, Lettich E, Nelson PL. Modified nomenclature for the "10%" electrode system. *J Clin Neurophysiol* 1988; **5**: 183–186.
53 Ferree TC, Luu P, Russell GS, Tucker DM. Scalp electrode impedance, infection risk, and EEG data quality. *Clin Neurophysiol* 2001; **112**: 536–544.
54 Clayson PE, Larson MJ. Psychometric properties of conflict monitoring and conflict adaptation indices: response time and conflict N2 event-related potentials. *Psychophysiology* 2013; **50**: 1209–1219.
55 Millner AJ, Jaroszewski AC, Chamarthi H, Pizzagalli DA. Behavioral and electrophysiological correlates of training-induced cognitive control improvements. *Neuroimage* 2012; **63**: 742–753.
56 Bertrand O, Perrin F, Pernier J. A theoretical justification of the average reference in topographic evoked potential studies. *Electroencephalogr Clin Neurophysiol* 1985; **62**: 462–464.
57 Tucker DM, Liotti M, Potts GF, Russell GS, Posner MI. Spatiotemporal analysis of brain electrical fields. *Hum Brain Mapp* 1994; **1**: 134–152.
58 Luck SJ. *An Introduction to the Event-Related Potential Technique*. The MIT Press: Cambridge, MA, USA, 2014.
59 Folstein JR, Van Petten C. Influence of cognitive control and mismatch on the N2 component of the ERP: a review. *Psychophysiology* 2008; **45**: 152–170.
60 Polich J. Updating P300: an integrative theory of P3a and P3b. *Clin Neurophysiol* 2007; **118**: 2128–2148.
61 Gehring WJ, Gratton G, Coles MGH, Donchin E. Probability effects on stimulus evaluation and response processes. *J Exp Psychol Hum Percept Perform* 1992; **18**: 198–216.
62 Donchin E. Presidential address, 1980. Surprise!...Surprise? *Psychophysiology* 1981; **18**: 493–513.
63 Johnson R. On the neural generators of the P300 component of the event-related potential. *Psychophysiology* 1993; **30**: 90–97.
64 Jennings JR, Wood CC. Letter: The epsilon-adjustment procedure for repeated-measures analyses of variance. *Psychophysiology* 1976; **13**: 277–278.
65 Kessler RC, Berglund P, Demler O, Jin R, Koretz D, Merikangas KR *et al.* The epidemiology of major depressive disorder: results from the National Comorbidity Survey Replication (NCS-R). *JAMA* 2003; **289**: 3095–3105.
66 Kessler RC, Chiu WT, Demler O, Merikangas KR, Walters EE. Prevalence, severity, and comorbidity of 12-month DSM-IV disorders in the National Comorbidity Survey Replication. *Arch Gen Psychiatry* 2005; **62**: 617–627.
67 Larson MJ, Clayson PE, Clawson A. Making sense of all the conflict: a theoretical review and critique of conflict-related ERPs. *Int J Psychophysiol* 2014; **93**: 283–297.
68 Babyak M, Blumenthal JA, Herman S, Khatri P, Doraiswamy M, Moore K *et al.* Exercise treatment for major depression: maintenance of therapeutic benefit at 10 months. *Psychosom Med* 2000; **62**: 633–638.
69 Blumenthal JA, Babyak MA, Moore KA, Craighead WE, Herman S, Khatri P *et al.* Effects of exercise training on older patients with major depression. *Arch Intern Med* 1999; **159**: 2349–2356.
70 Trivedi MH, Greer TL, Church TS, Carmody TJ, Grannemann BD, Galper DI *et al.* Exercise as an augmentation treatment for nonremitted major depressive disorder: a randomized, parallel dose comparison. *J Clin Psychiatry* 2011; **72**: 677–684.
71 Brosse AL, Sheets ES, Lett HS, Blumenthal JA. Exercise and the treatment of clinical depression in adults: recent findings and future directions. *Sports Med* 2002; **32**: 741–760.
72 Bates ME, Barry D, Labouvie EW, Fals-Stewart W, Voelbel G, Buckman JF. Risk factors and neuropsychological recovery in clients with alcohol use disorders who were exposed to different treatments. *J Consult Clin Psychol* 2004; **72**: 1073–1080.
73 Thayer JF, Yamamoto SS, Brosschot JF. The relationship of autonomic imbalance, heart rate variability and cardiovascular disease risk factors. *Int J Cardiol* 2010; **141**: 122–131.
74 O'Connor M-F, Allen JJB, Kaszniak AW. Autonomic and emotion regulation in bereavement and depression. *J Psychosom Res* 2002; **52**: 183–185.
75 Alderman BL, Olson RL. The relation of aerobic fitness to cognitive control and heart rate variability: a neurovisceral integration study. *Biol Psychol* 2014; **99**: 26–33.
76 Dery N, Pilgrim M, Gibala M, Gillen J, Wojtowicz JM, Macqueen G *et al.* Adult hippocampal neurogenesis reduces memory interference in humans: opposing effects of aerobic exercise and depression. *Front Neurosci* 2013; **7**: 66.
77 Wang Z, van Praag H. Exercise and the brain: neurogenesis, synaptic plasticity, spine density, and angiogenesis. In: *Functional Neuroimaging in Exercise and Sport Sciences*. Springer: New York, NY, USA, 2012, pp 3–24.
78 Ernst C, Olson AK, Pinel JP, Lam RW, Christie BR. Antidepressant effects of exercise: evidence for an adult-neurogenesis hypothesis? *J Psychiatry Neurosci* 2006; **31**: 84–92.
79 Marchand WR. Mindfulness-based stress reduction, mindfulness-based cognitive therapy, and Zen meditation for depression, anxiety, pain, and psychological distress. *J Psychiatr Pract* 2012; **18**: 233–252.
80 Goyal M, Singh S, Sibinga EM, Gould NF, Rowland-Seymour A, Sharma R *et al.* Meditation programs for psychological stress and well-being: a systematic review and meta-analysis. *JAMA Intern Med* 2014; **174**: 357–368.
81 Hofmann SG, Sawyer AT, Witt AA, Oh D. The effect of mindfulness-based therapy on anxiety and depression: a meta-analytic review. *J Consult Clin Psychol* 2010; **78**: 169–183.
82 Mars TS, Abbey H. Mindfulness meditation practise as a healthcare intervention: a systematic review. *Int J Osteopath Med* 2010; **13**: 56–66.
83 Kok BE, Waugh CE, Fredrickson BL. Meditation and health: the search for mechanisms of action. *Soc Personal Psychol Compass* 2013; **7**: 27–39.
84 Kabat-Zinn J. Mindfulness-based interventions in context: past, present, and future. *Clin Psychol Sci Pract* 2003; **10**: 144–155.
85 Davidson RJ, Kabat-Zinn J, Schumacher J, Rosenkranz M, Muller D, Santorelli SF *et al.* Alterations in brain and immune function produced by mindfulness meditation. *Psychosom Med* 2003; **65**: 564–570.
86 Jha AP, Krompinger J, Baime MJ. Mindfulness training modifies subsystems of attention. *Cogn Affect Behav Neurosci* 2007; **7**: 109–119.
87 Iversen S, Kupfermann I, Kandel ER. Emotional states and feelings. In: Kandel ER, Schwartz J, Jessel TM (eds). *Principles of Neural Science*, 4th edn. McGraw-Hill: New York, NY, USA, 2000, pp 982–997.
88 Benarroch EE. The central autonomic network. In: Low PA (ed). *Clinical Autonomic Disorders*, 2nd edn. Lippincott-Raven: Philadelphia, PA, USA, 1997, pp 17–28.
89 Holzel BK, Carmody J, Vangel M, Congleton C, Yerramsetti SM, Gard T *et al.* Mindfulness practice leads to increases in regional brain gray matter density. *Psychiatry Res* 2011; **191**: 36–43.
90 Dickenson J, Berkman ET, Arch J, Lieberman MD. Neural correlates of focused attention during a brief mindfulness induction. *Soc Cogn Affect Neurosci* 2013; **8**: 40–47.
91 Gotlib IH, Joormann J. Cognition and depression: current status and future directions. *Annu Rev Clin Psychol* 2010; **6**: 285–312.
92 Bangasser DA, Shors TJ. The hippocampus is necessary for enhancements and impairments of learning following stress. *Nat Neurosci* 2007; **10**: 1401–1403.
93 Pizzagalli DA. Frontocingulate dysfunction in depression: toward biomarkers of treatment response. *Neuropsychopharmacology* 2011; **36**: 183–206.
94 Beylin AV, Gandhi CC, Wood GE, Talk AC, Matzel LD, Shors TJ. The role of the hippocampus in trace conditioning: temporal discontinuity or task difficulty? *Neurobiol Learn Mem* 2001; **76**: 447–461.
95 Clark RE, Squire LR. Classical conditioning and brain systems: the role of awareness. *Science* 1998; **280**: 77–81.
96 Stone SSD, Teixeira CM, DeVito LM, Zaslavsky K, Josselyn SA, Lozano AM *et al.* Stimulation of entorhinal cortex promotes adult neurogenesis and facilitates spatial memory. *J Neurosci* 2011; **31**: 13469–13484.
97 Baars BJ, Gage NM. *Cognition Brain and Consciousness: An Introduction to Cognitive Neuroscience, 2nd edn.* Academic Press: New York, NY, USA, 2010.
98 Cooney RE, Joormann J, Eugene F, Dennis EL, Gotlib IH. Neural correlates of rumination in depression. *Cogn Affect Behav Neurosci* 2010; **10**: 470–478.
99 Siegle GJ, Thayer JF. Physiological aspects of depressive rumination. In: Papageorgiou C, Wells A (eds). *Depressive Rumination: Nature, Theory and Treatment*. John Wiley & Sons, Ltd.: Chichester, UK, 2003, pp 79–104.
100 *Clinical Perspectives on Autobiographical Memory*. Cambridge University Press: Cambridge, UK, 2015.
101 Squire LR, van der Horst AS, McDuff SG, Frascino JC, Hopkins RO, Mauldin KN. Role of the hippocampus in remembering the past and imagining the future. *Proc Natl Acad Sci USA* 2010; **107**: 19044–19048.
102 Kirsch I. *Emperor's New Drugs: Exploding the Antidepressant Myth*. The Random House Group, Ltd: London, UK, 2009.
103 Kirsch I, Deacon BJ, Huedo-Medina TB, Scoboria A, Moore TJ, Johnson BT. Initial severity and antidepressant benefits: a meta-analysis of data submitted to the Food and Drug Administration. *PLoS Med* 2008; **5**: e45.
104 Kirsch I, Moore TJ, Scoboria A, Nicholls SS. The emperor's new drugs: an analysis of antidepressant medication data submitted to the US Food and Drug Administration. *Prev Treat* 2002; **5**: 23a.

This work is licensed under a Creative Commons Attribution 4.0 International License. The images or other third party material in this article are included in the article's Creative Commons license, unless indicated otherwise in the credit line; if the material is not included under the Creative Commons license, users will need to obtain permission from the license holder to reproduce the material. To view a copy of this license, visit http://creativecommons.org/licenses/by/4.0/
