# Supplementary material for: Does physical activity-based intervention decrease repetitive negative thinking? A systematic review
Source: PLoS One. 2025 Apr 1;20(4):e0319806. doi: 10.1371/journal.pone.0319806 (PMC11960971; doi:10.1371/journal.pone.0319806)
Supplement: S1 File — https://doi.org/10.6084/m9.figshare.25711734. (ZIP) [file pone.0319806.s001.zip › supporting information/paper file/Shors 2018.pdf]

frontiers in Neuroscience

ORIGINAL RESEARCH
published: 23 April 2018
doi: 10.3389/fnins.2018.00211

Check for updates

MAP Training My Brain™: Meditation Plus Aerobic Exercise Lessens Trauma of Sexual Violence More Than Either Activity Alone

Tracey J. Shors*, Han Y. M. Chang and Emma M. Millon

Behavioral and Systems Neuroscience, Department of Psychology, Center for Collaborative Neuroscience, Rutgers University, Piscataway, NJ, United States

Sexual violence against women often leads to post-traumatic stress disorder (PTSD), a mental illness characterized by intrusive thoughts and memories about the traumatic event (Shors and Millon, 2016). These mental processes are obviously generated by the brain but often felt in the body. *MAP Training My Brain*™ is a novel clinical intervention that combines mental training of the brain with physical training of the body (Curlik and Shors, 2013; Shors et al., 2014). Each training session begins with 20-min of sitting meditation, followed by 10-min of slow-walking meditation, and ending with 30-min of aerobic exercise at 60–80% of the maximum heart rate (see maptrainmybrain.com). In previous studies, the combination of mental and physical (MAP) training together significantly reduced symptoms of depression and ruminative thoughts, while reducing anxiety (Shors et al., 2014, 2017; Alderman et al., 2016). We also documented positive changes in brain activity during cognitive control and whole-body oxygen consumption in various populations. In the present pilot study, we asked whether the combination of meditation and aerobic exercise during MAP Training would reduce trauma-related thoughts, ruminations, and memories in women and if so, whether the combination would be more effective than either activity alone. To test this hypothesis, interventions were provided to a group of women ($n = 105$), many of whom had a history of sexual violence ($n = 32$). Groups were trained with (1) MAP Training, (2) meditation alone, (3) aerobic exercise alone, or (4) not trained. Individuals in training groups completed two sessions a week for at least 6 weeks. *MAP Training My Brain*™ significantly reduced post-traumatic cognitions and ruminative thoughts in women with a history of sexual violence, whereas meditation alone, and exercise alone did not. MAP Training significantly enhanced a measure of self-worth, whereas meditation and exercise alone did not. Similar positive effects were observed for all participants, although meditation alone was also effective in reducing trauma-related thoughts. Overall, these data indicate the combination of meditation and exercise is synergistic. As a consequence, MAP Training is preferable and especially so for women who have experienced sexual violence in their past. Simply put, the whole is greater than the sum of its parts.

Keywords: violence, trauma, fear, memory, PTSD, Mental and Physical (MAP) Training, meditation, exercise

OPEN ACCESS

Edited by:
Mardi A. Crane-Godreau, Geisel School of Medicine at Dartmouth, United States

Reviewed by:
Bruno Bonaz, Centre Hospitalier Universitaire de Grenoble, France
Lisa D. Miller, Independent Researcher, Los Gatos, CA, United States

****Correspondence:***
Tracey J. Shors
shors@rutgers.edu

Specialty section:
This article was submitted to Autonomic Neuroscience, a section of the journal Frontiers in Neuroscience

Received: *08 January 2018*
Accepted: *16 March 2018*
Published: *23 April 2018*

Citation:
Shors TJ, Chang HYM and Millon EM (2018) MAP Training My Brain™: Meditation Plus Aerobic Exercise Lessens Trauma of Sexual Violence More Than Either Activity Alone. Front. Neurosci. 12:211. doi: 10.3389/fnins.2018.00211

INTRODUCTION

More than 25% of women worldwide experience physical or sexual violence (SV) in their lifetime, with similar estimates in the United States (Kessler, 2000; WHO, 2013; Kessler et al., 2017). Exact statistics are difficult to obtain because many women do not report the event, and many fail to seek medical assistance. Sexual assault often induces post-traumatic stress disorder (PTSD) (Kessler, 2000; Ozer et al., 2003), a category of mental illness characterized by extreme fear, helplessness and horror during the traumatic event, followed by months and sometimes years of symptoms which include re-experiencing the trauma, avoiding reminders, and hyperarousal, along with impaired cognition and mood (American Psychiatric Association, 2013). Because women are four times as likely as men to experience sexual assault and more than ten times as likely to experience rape (Kessler, 2000), PTSD is common among women who have experienced sexual violence. Most resources focus on preventing sexual violence and helping women with PTSD; not enough target women who are not actively seeking help and do not have any diagnosable disorder. The pressing issue is: what can we do to reduce PTSD and stress-related cognition among women with a history of sexual violence and assault? In the present study, we provided three different interventions to a large group of women, many of whom had experienced sexual trauma in their lives.

MAP Training stands for Mental and Physical Training (Curlik and Shors, 2013; Shors et al., 2014, 2017; Alderman et al., 2016). The intervention, known as *MAP Training My Brain*™ was inspired by laboratory studies connecting changes in the brain with mental and physical exercise (Gould et al., 1999; van Praag et al., 1999; Shors, 2009; Curlik et al., 2013; Nokia et al., 2016). We chose meditation for the mental training component because it is effortful for most human beings and presents a new opportunity for learning upon each session. For the physical training, we chose aerobic exercise. The two activities are done one after the other, and easily remembered as SIT, WALK, SWEAT (**Figure 1**). Each session of MAP Training consists of 20-min of sitting meditation, followed by 10-min of very slow walking meditation and then ending with 30-min of aerobic exercise. Exercise can be accomplished in any mode (running, spinning, aerobic dancing, rowing, etc.), as long as heart rate is maintained at 60–80% of a person's maximum. For logistical reasons, the meditation component is immediately followed by the aerobic exercise component. Importantly, each session is only 1 h and therefore easily incorporated into most lifestyles.

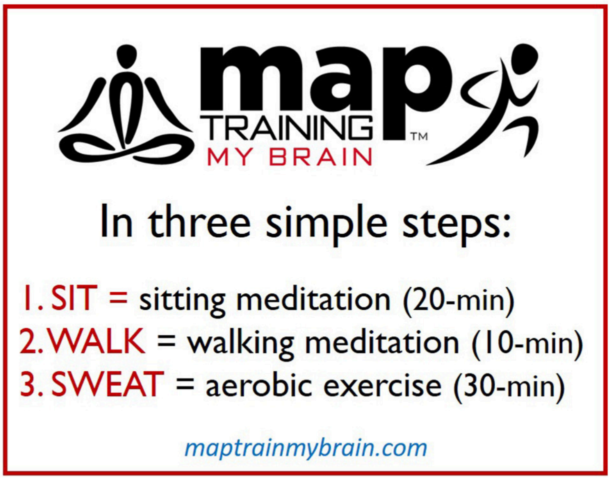

FIGURE 1 | Each session of Mental and Physical (MAP) Training consists of 20-min of sitting meditation, followed by 10-min of very slow walking meditation, ending with 30-min of aerobic exercise with heart rate maintained at 60–80% of the participant's maximum.

In previous and ongoing studies, participants engaged in two sessions of MAP Training per week. In our first published study, we provided MAP Training to a group of young women who had experienced untold trauma while homeless and many came from impoverished childhoods. After 8 weeks of MAP Training, symptoms of depression and anxiety decreased significantly (Shors et al., 2014). MAP Training also produced a significant and relatively large increase in VO_2, which is a direct measure of oxygen consumption by the body. In a second study, we provided MAP Training to adult men and women diagnosed with major depressive disorder (MDD). After MAP Training, participants with MDD were much less depressed with an average 40% decrease in symptoms (Alderman et al., 2016). Even otherwise healthy controls were less depressed. We also evaluated ruminations, which are repeated thoughts, typically autobiographical and negative in nature. These thoughts have been associated with depression and as expected, they decreased after MAP Training along with symptoms of depression in individuals with and without MDD (Shors et al., 2017). MAP Training also produced positive changes in brain activity related to cognitive control (Alderman et al., 2016). In summary, MAP Training has proven effective in reducing symptoms of mental health and in some individuals, increasing brain and whole-body health.

Women are much more likely than men to be diagnosed with PTSD. Of all traumas, sexual assault and violence is the most likely to induce PTSD, when compared to other trauma such as war (Kessler et al., 2017). Because sexual violence is most often inflicted on women, they are at high risk. Indeed, women with PTSD often report past experiences of sexual harassment, violence and abuse. Because MAP Training is so effective in reducing symptoms associated with depression and anxiety as well as ruminations about the past, we hypothesized that women who have a history of sexual violence and the trauma associated with this experience would respond especially well to MAP Training. We also wanted to make the intervention available to women who might not otherwise seek help for their symptoms and/or who do not even know they have symptoms. To accomplish our objectives, we recruited young adult women using flyers and through an online process. After giving their written informed consent, women were interviewed using the Structured Clinical Interview Diagnosis (SCID) to detect major mental illness and document trauma-related symptoms. They then completed questionnaires about thoughts and feelings surrounding the trauma experience.

The primary goal of this pilot study was to determine whether *MAP Training My Brain*™ would help young adult women learn to recover from trauma-related thoughts and cognitions. A second goal was to determine whether the combination of meditation and aerobic exercise was better in this regard than either meditation alone or exercise alone. Therefore, we randomly assigned the participants into three treatment arms: (1) meditation plus aerobic exercise, (2) meditation alone, and (3) aerobic exercise alone. They completed two sessions a week for at least 6 weeks and then we retested them with the same battery of questionnaires and tasks. We included a fourth group of women (no training) who completed all the testing procedures but did not participate in any of the interventions. They were given the opportunity to participate in MAP Training at a later date (wait-list control).

METHODS

Participants

Adult women ($M_{\text{age}} = 20$ years, *range* = 18–32 years) were recruited with flyers from the student body of a northeastern university. All participants were provided written informed consent in accordance with the Declaration of Helsinki. This study was carried out in accordance with the recommendations of the Institutional Review Board at Rutgers University. All research staff were certified by the Collaborative Institutional Training Initiative (CITI) for Human Subjects Research in conducting proper clinical trial procedures for human subjects research.

During session one, recruited participants were given study information and asked for their informed consent. Participants then received a clinical interview (Structured Clinical Interview for DSM-5; SCID-5-RV; First et al., 2015). Psychological and health history taken from the SCID and questionnaires were used to determine whether a participant was included or excluded from the study. Inclusion criteria include: (1) women aged 18–40 years; (2) not engaged in a regular exercise program (<3 days/wk for 20-min or less per session over the past month); (3) not engaged in any formal meditation practice (not meditating more than 30-min total per week and less than 200-h in lifetime); (4) free from physical limitations or contraindications to exercise; and (5) able and willing to provide informed consent. The age restriction takes into account the effects of aging on cardiovascular and cognitive function (Stein et al., 1997; Clark et al., 2006). Exclusion criteria included those with severe psychopathology, including bipolar spectrum disorders, schizophrenia spectrum disorders, and substance use disorders and those at high risk for suicide (Joiner et al., 1999), determined from the SCID. All participants were given contact information for counseling and violence assistance at the university during consent and at the end of the testing session. To facilitate recruitment and retention, we provided appropriate compensation. If an individual met any of the exclusion criteria, she was compensated for her screening time but did not continue further in the study. If an individual met all of the inclusion criteria, she continued with the session one assessments, during which staff obtain health and medical histories, prior and current medication use with the Health History Questionnaire (HHQ; Sadock et al., 2014) and physical behaviors with the International Physical Activity Questionnaire (IPAQ; Booth, 2000; Craig et al., 2003) or Physical Activity Readiness Questionnaire (PAR-Q; Chisholm et al., 1975). After consent and screening measures were completed, potential recruits were led into testing rooms to assess outcome measures as follows:

1) The Post-traumatic Cognitions Inventory (PTCI; Foa et al., 1999) was used to assess thoughts and emotions related to stressful life events. The 33-item questionnaire asks the participant to rate items such as "*I have to be on guard all the time*" and "*I feel like I don't know myself anymore*." Scores on the PTCI indicate to what extent the participant has negative cognitions about the self or the world, or feels self-blame as a result of a stressful event. Higher PTCI scores are indicative of more negative post-traumatic cognitions and are associated with greater numbers of PTSD symptoms (Foa and Rauch, 2004). The instructions in this questionnaire were altered to reflect responses to a very stressful event rather than a traumatic event, because not all participants had a traumatic experience.
2) The Ruminative Responses Scale (RRS; Treynor et al., 2003) is a 22-item scale used to assess repeated thoughts which relate to depression, brooding and reflection (min 22; max 88).
3) The Best Self Scale was used to assess self-worth (Ogilvie and Clark, 1992). It asks the participant to mark how close or far one feels to her ideal self on a scale from 1 (*far from being at my best*) to 10 (*close to being at my best*). Lower scores have been shown to be associated with depression, particularly in women (Ogilvie and Clark, 1992; Carver et al., 1999).
4) The Autobiographical Memory Questionnaire (AMQ; Rubin et al., 2003) was used to assess the strength of a stressful life memory. The questionnaire includes 19 items, which are typically scored individually or in clusters, depending on the research question of interest (Rubin et al., 2011). We were especially interested in the strength of the memory rather than accuracy. To protect their privacy, we did not ask participants to identify the event but rather to just reflect on the most stressful event in their life. We included 12 individual items which assessed features of the memory: sensory details ("*As I remember the event, I can hear it in my mind*" or "*see it in my mind*"), temporal and spatial details ("*As I remember the event, I know its temporal and spatial layout*"), emotional details ("*As I remember the event, I feel the emotions now that I felt then*"), and significance ("*the memory is significant in my life*"). Total scores were calculated, with greater scores representing a stronger autobiographical memory.

Meditation and aerobic exercise components (alone and together) were provided in a recreational facility and/or a large otherwise empty meditation room. The MAP Training staff supervised all intervention sessions. Meditation sessions were facilitated by trained practitioners. Aerobic exercise sessions were supervised and/or led by trained aerobics instructors. Meditation and aerobic exercise sessions were completed in

the group exercise room. Aerobic exercises were completed in the fully-equipped exercise facility or during 30-min aerobic exercise classes. To be compensated, participants had to complete two sessions per week for 6 weeks. They were allowed to miss sessions, provided that they attended at least 12 sessions and a missed session was made up within 2-weeks. We sent email reminders a day before, which we have used in the past to produce positive adherence results (∼75%; Alderman et al., 2016). Based on individual availabilities, participants were randomly placed in one of three intervention groups or a no-training wait-list condition, as described below.

1) **Mental and Physical (MAP) Training** *(MAP Training My Brain*™). Participants first engaged in 20-min of focused-attention (FA) meditation (Farb et al., 2007; Lutz et al., 2008). In traditional circles, this practice is most similar to Zen meditation. During the first session, participants were first instructed to sit, either cross-legged on the floor or in a straight-back chair. They were instructed to place the palms of their hands one on top of the other, with the dominant hand on the bottom and the thumb tips loosely touching. They were instructed to hold their arms slightly away from the body but relaxed at the sides, with eyes either closed or half-open and focused three feet in front of the legs. Participants were then instructed to begin focusing their attention on their breath, noticing as the air goes in and out. They were instructed to especially focus attention on that space in time between the inhale and exhale. They were then instructed to count these spaces in time from 1 to 10 and beyond. If the participant lost count, she was instructed to mentally "let go" of intervening thoughts without judgment and return to counting the breath, beginning again at one. This training procedure provided a context in which the participant can learn to recognize the presence of an interfering thought and further learn to regain attention to the focus, which in this case is the breath. This first component of FA meditation was conducted in complete silence and continued for 20-min until a standardized bell rang (using a smartphone meditation application). After the bell, participants stretched out their legs and arms to regain blood flow. They then stood up slowly to begin the 10-min walking meditation. Participants gathered in a large circle with about 3-ft between each of them. Facing in one direction, they were instructed to clasp their hands gently behind their back and direct their eyes about 3-ft on the floor in front of them. They were guided to focus their mental attention onto their feet as they walked very slowly (about 10 steps per min) in the circle, paying attention to the bodily sensations as body weight shifted from one foot onto the other and as it shifted through the heel to the ball of the foot. As attention drifts, the participants were instructed to recognize the interruption and without judgment, bring attention back to the feet. This component of meditation training was also conducted in complete silence until a standardized bell rang (smartphone meditation application). Immediately after the 30-min meditation component was complete, participants were instructed to put on their exercise shoes and walk directly to the exercise room facility (<500-ft from the meditation room) or remain in the same room for an organized group exercise class. Participants either exercised on a treadmill or elliptical machines at moderate-intensity for 30-min or completed the 30-min aerobic exercise class. After 5-min of supervised warm-up, participants were instructed to maintain their heart rate at 60–80% of their individual maximum, which was recorded halfway through (at minute 15) and at the end of each session. Maximum heart rate was calculated by subtracting the participant's age from 220. After the aerobic exercise component, participants were supervised through a 5-min cool down, exercising at low intensity until heart rate values returned to a pre-exercise range, at which point the MAP training session ended.
2) **Mental Training Alone**. Participants engaging in the meditation alone condition meditated for a total of 30-min (20-min sitting followed by 10-min slow walking), as described above.
3) **Physical Training Alone**. Participants engaging in the aerobic exercise alone condition exercised at moderate-intensity for 30-min, as described above.
4) **No Training**. Participants without training were assigned to a wait-list.

After training, all participants attended a follow-up session, which included all steps from the first session except the clinical interview. Participants who did not attend training were asked to return for the follow-up session at least 6 weeks after their initial session.

The data were analyzed with analysis of variance with time (pre-training to post-training) as the within-subject independent variable and training group (MAP Training, meditation alone, exercise alone, and no training) as the between-subject independent variable. The following outcome measures are presented as follows: post-traumatic cognitions (PTCI scores), rumination (RRS scores), self-worth (Best Self Scale scores) and the memories about a very stressful life event (AMQ scores). Data were analyzed according to the complete sample of participants with N's as follows: MAP ($n = 25$), meditation alone ($n = 24$), exercise alone ($n = 21$) and no training ($n = 35$). The groups with sexual violence were smaller with N's as follows: MAP ($n = 8$), meditation alone ($n = 4$), exercise alone ($n = 8$), no training ($n = 12$). Because of low numbers in some groups, we analyzed data for all participants ($n = 105$) and for those with SV history ($n = 32$) separately.

RESULTS

Post-traumatic thoughts and cognitions were assessed with the PTCI. With respect to ANOVA, pairwise comparisons indicated a significant decrease in trauma-related cognitions after MAP Training, [$F_{(1, 101)} = 4.06$, $p < 0.05$] and meditation alone, [$F_{(1, 101)} = 8.40$, $p = 0.005$], but not for exercise alone ($p > 0.05$). They did not change in those who were not trained ($p > 0.05$). These data suggest that all participants benefited by MAP Training and meditation alone when asked to reflect on a very stressful event in their life (**Figure 2A**). We also analyzed

the subgroup of women with trauma history of sexual violence, as indicated by the Structured Clinical Interview (SCID). In this group, MAP Training significantly reduced trauma-related cognitions, [$F_{(1, 28)} = 4.84$, $p < 0.05$], whereas meditation alone did not change these scores, $p > 0.05$ (**Figure 3A**). Exercise alone also was ineffective ($p > 0.05$) and scores did not change in those who were not trained ($p > 0.05$). The percent change was 16% for trauma-related cognitions in the MAP Training group, whereas all other training conditions did not reach 10% change. Thus, MAP Training was effective in reducing trauma-related cognitions in women with SV history whereas exercise and meditation alone were not.

Ruminative thoughts were assessed with the RRS. As shown in **Figure 2B**, RRS scores decreased by more than 5 points after MAP Training when assessing all participants. This decrease was highly significant, [$F_{(1, 101)} = 9.18$, $p < 0.005$]. Surprisingly, neither exercise alone nor meditation alone reduced these types of thoughts. Not surprisingly, there was no change in the women who did not participate in any training (p's > 0.05). In women with sexual violence history, RRS scores were reduced after MAP Training, [$F_{(1, 28)} = 4.54$, $p < 0.05$] and again, not by any of the other training procedures (**Figure 3B**). These data suggest that MAP Training is especially and perhaps uniquely effective in reducing ruminations when compared to meditation or exercise alone.

Changes in self-worth were assessed before and after the interventions using the Best Self Scale across all participants. As shown in **Figure 2C**, self-worth scores increased significantly after MAP Training, [$F_{(1, 101)} = 6.30$, $p < 0.05$], but not after meditation only or exercise only (p's > 0.05). Scores did not change in the group who did not participate in any training ($p > 0.05$). In women with sexual trauma history, self-worth scores were significantly enhanced after MAP Training, [$F_{(1, 28)} = 5.83$, $p < 0.05$], but not by the other two training procedures. Scores did not change in the groups who did not participate in any training (**Figure 3C**).

Participants were asked to report on details of a very stressful event from the past with the AMQ. These reports were quantified and assessed across participants according to training group. Interestingly, the strength of the memory significantly increased in the group with no training, [$F_{(1, 101)} = 9.42$, $p < 0.01$], but did not change for any of the groups that did participate in training ($p > 0.05$). There were no significant differences between training groups. To illustrate these changes, we present the means for the three training groups compared to the untrained group in **Figure 4**. We also analyzed items within the AMQ that changed (i.e., increased in strength) for participants who did not participate in the training. The memory items that tended to increase in strength over the 6-week interval included (1) reliving the event, (2) seeing, and (3) hearing the event and (4) hearing people's voices during the event.

Relationships among rumination, trauma-related cognitions and strength of a stressful autobiographical memory were also assessed before training. Across the entire sample, scores on the AMQ were significantly associated with PTCI scores, $r = 0.45$, $p < 0.01$, as well as scores on the RRS, $r = 0.47$, $p < 0.01$. Correlations were similar after training ($p < 0.01$). These data indicate that those women who reported more sensory-related and contextual details of a stressful past memory had a greater tendency to ruminate and had more trauma-related thoughts.

We further tested relationships among rumination, trauma-related thoughts and self-worth scores across the entire sample. Before training, post-traumatic cognitions as assessed by the PTCI were highly correlated with ruminative thoughts as assessed by the RRS, $r = 0.67$, $p < 0.01$. The number of ruminative thoughts negatively correlated with self-worth as assessed by the Best Self Scale, $r = -0.41$, $p < 0.01$. Additionally, post-traumatic cognitions negatively correlated with self-worth, $r = -0.51$, $p < 0.01$. These relationships among outcomes remained significant ($p < 0.01$) and did not change as a result of training, as would be expected given the group data. These data indicate that those participants who rated themselves as closer to their best (ideal) self also reported fewer ruminative and post-traumatic thoughts and vice versa.

DISCUSSION

Despite the undeniable connection between sexual trauma and mental illness, few interventions are tailored for women who experience sexual violence. In this pilot study, we present the clinical intervention of *MAP Training My Brain*™ as an especially effective intervention for decreasing trauma-related thoughts in women in general and especially in women with SV history. These data further suggest that the combination of the mental and physical training is more effective than either activity alone (**Figures 3**, **5**). When analyzing all participants, irrespective of SV history, both MAP Training and meditation alone significantly decreased trauma-related cognitions (**Figure 2**). This is important information because it suggests that the meditation component of MAP Training is especially useful for targeting trauma-related memories and cognitions. However, MAP Training but not meditation or exercise alone was effective in women who had experienced sexual violence. Thus, there appears to be a synergistic effect of the two activities that specifically helps women learn to recover from sexual violence, at least with respect to negative thoughts about trauma.

The present data further indicate that rumination responds best to the combination of meditation and aerobic exercise. Specifically, MAP Training significantly reduced ruminative thoughts in all participants and in women with SV history, whereas meditation alone or exercise alone did not. Together, the decrease in ruminative thoughts coupled with a decrease in trauma-related thoughts could be transformative for a woman as she learns to recover from the stress of life and the even more traumatic experience of sexual violence. Finally, we report an increase in self-worth (less discrepancy between real and ideal self) after MAP Training, but not after meditation or exercise alone. Thus, the training program not only decreased negative thoughts but also increased positive thoughts and feelings about one's self. As a whole, these data indicate the combination of meditation and exercise can induce a relatively wide range of beneficial cognitive and mental health responses in women with and without trauma history.

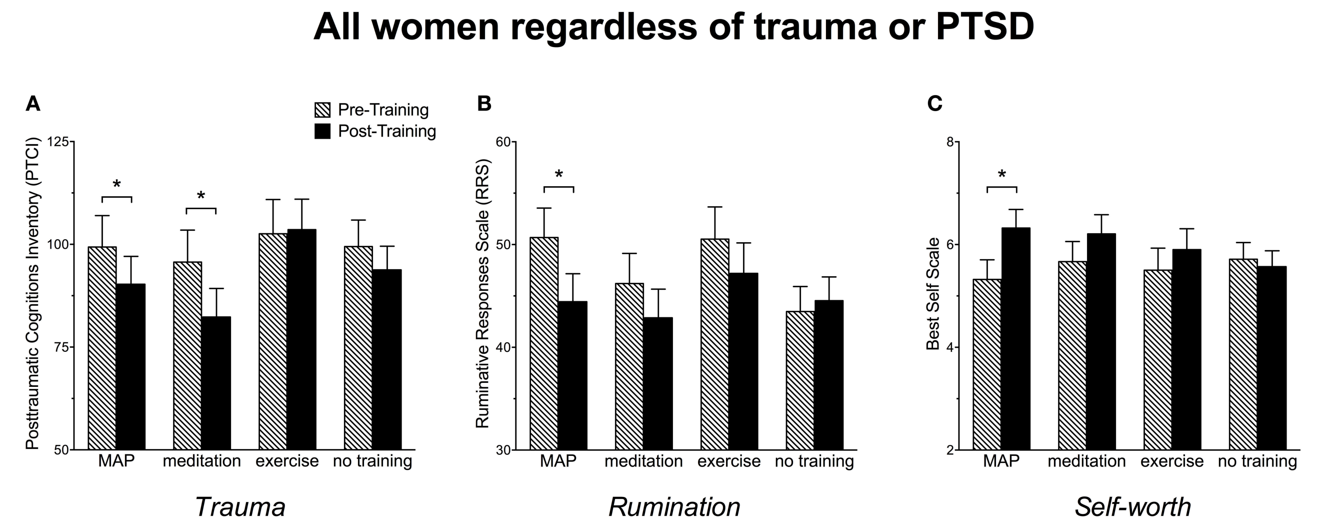

FIGURE 2 | (A) Women reported a significant decrease in trauma-related cognitions as assessed by the Post-traumatic Cognitions Inventory (PTCI) following MAP Training and after meditation alone but not after exercise alone or in those with no training. **(B)** Women reported a significant decrease in ruminative thoughts as assessed by the Ruminative Responses Scale (RRS) following MAP Training, but not after meditation alone, exercise alone, or in those with no training. **(C)** Women reported a significant enhancement in self-worth as assessed by the Best Self Scale following MAP Training but not after meditation alone, exercise alone, or in those with no training. Bars reflect means and standard error. Asterisks (*) indicate $p < 0.05$.

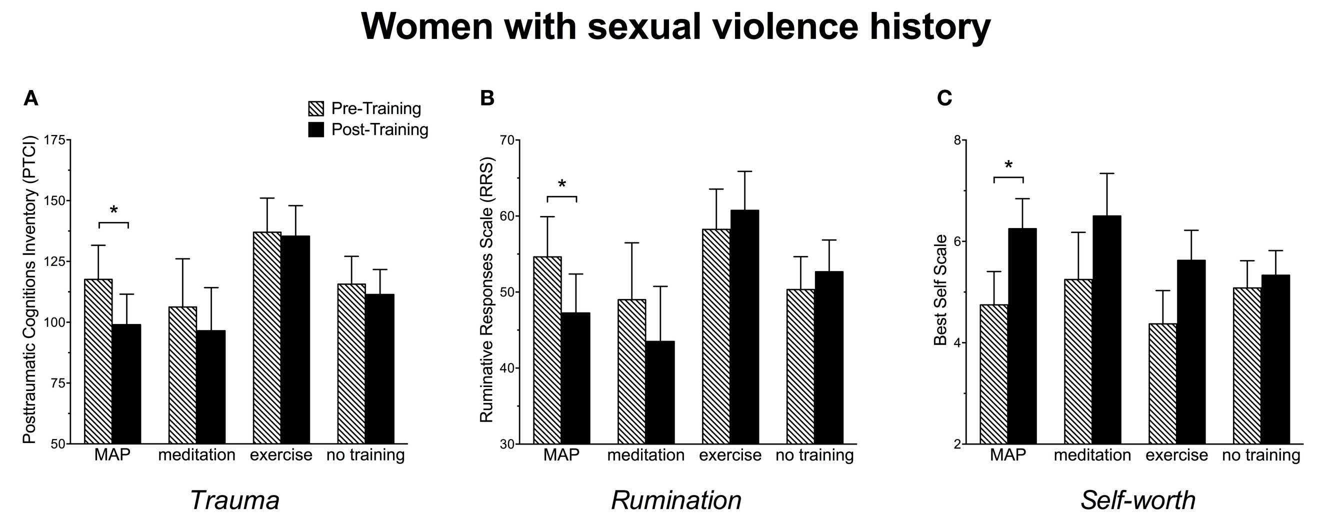

FIGURE 3 | (A) Women with a history of sexual violence reported a significant decrease in trauma-related cognitions as assessed by the Post-traumatic Cognitions Inventory (PTCI) following MAP Training but not after meditation alone, exercise alone or in those with no training. **(B)** Women with a history of sexual violence reported a significant decrease in ruminative thoughts as assessed by the Ruminative Responses Scale (RRS) following MAP Training, but not after meditation alone, exercise alone, or in those with no training. **(C)** Women with a history of sexual violence reported a significant enhancement in self-worth as assessed by the Best Self Scale following MAP Training but not after meditation alone, exercise alone, or in those with no training. Bars reflect means and standard error. Asterisks (*) indicate $p < 0.05$.

Trauma-Related Thoughts Reduced by MAP Training

MAP Training and meditation alone significantly decreased trauma-related cognitions for all participants. However, only MAP Training significantly lessened these types of thoughts for women with SV history. Taken together, these data suggest that meditation is an effective means for reducing trauma-related thoughts, which is then facilitated by the aerobic exercise component in women with SV history. During the silent meditation component, the participant was instructed to let thoughts "come and go." In other words, as thoughts arise in the mental space, the person should not follow the thought into other thoughts nor attach emotions to the thought. But even if this does happen, and it does, the person is instructed to let the thought

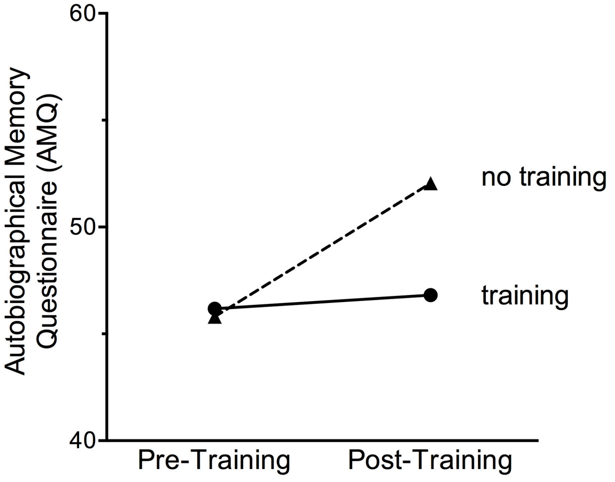

FIGURE 4 | The strength of an autobiographical memory of a very stressful event did not change after any of the training sessions (MAP Training, meditation or exercise alone). However, the strength of the personal stressful memory was enhanced in women who did not participate in any training procedures.

map TRAINING MY BRAIN	Trauma	Rumination	Self-worth
MAP Training	✓	✓	✓
Meditation	∅	∅	∅
Exercise	∅	∅	∅
No training	∅	∅	∅

FIGURE 5 | The combination of meditation and aerobic exercise during MAP Training was more effective than either meditation or exercise alone, especially in reducing trauma-related cognitions and ruminative thoughts, and enhancing self-worth in women with a history of sexual violence.

go. This process is often referred to as "nonattachment." In a previous study, we documented that women with sexual trauma history ruminate more and are especially likely to rehearse brooding and depressive thoughts about the past (Millon et al., under review). These same women also reported the trauma memories as more vivid, remembered more sensory details about the event and considered the event a significant part of their life story. Despite these reports, we cannot know whether participants were thinking about the traumatic event during meditation. Assuming they were, it is possible that meditation serves as a natural means of exposure therapy, which may be facilitated by the state of the body during meditation. It is generally assumed that meditation activates the parasympathetic nervous system, reducing heart rate and blood pressure, slowing the breath, etc. Learning to let the negative thoughts go while in a relaxed parasympathetic state appears to be especially effective for reducing some symptoms of trauma, as detected with the PTCI and RRS. It is noted that only MAP Training significantly decreased reports of these types of thoughts in women with SV history. Thus, meditation plus aerobic exercise is necessary to significantly induce these changes. We speculate that the aerobic exercise, because it increases blood flow to the brain, consolidates the learning which occurred during the mental training component. It would be difficult to prove, but such a mechanism would explain the synergistic effect that was evident after MAP Training but not after meditation or exercise alone.

Ruminative Thoughts Reduced by MAP Training

Rumination is a potential consequence of sexual trauma and abuse. During these thought processes, an individual impulsively and repeatedly rehearses thoughts and memories, most of which are autobiographical and negative in content. Ruminative thoughts are especially evident in women with stress-related illnesses such as depression and in some reports, PTSD (Shors et al., 2017). Despite a growing appreciation for their presence, we know less about how to reduce them. Exposure therapy, during which an individual is exposed to trauma-related stimuli, can reduce ruminative thoughts through learning processes related to extinction (Foa et al., 2002; Resick et al., 2002). However, there is some concern these therapies may exacerbate the problem because the rehearsal of the event creates yet more trauma-related memories—the so-called "multiple trace theory" (Nadel and Hardt, 2011). Regardless, it is generally accepted that ruminations interfere with the "normal" processing capacity of the hippocampus, a brain region necessary for acquisition and rehearsal of autobiographical memories. Indeed, high ruminators expressed less hippocampal activation to loss events with corresponding deficits in functional connectivity (Hach et al., 2014; Johnston et al., 2015). In the present study, ruminative thoughts were significantly reduced after MAP Training, but were not reduced after meditation alone or exercise alone. Again, these data indicate the combination of meditation and exercise is especially effective in reducing these types of negative thought processes. Fewer ruminations may contribute to fewer trauma-related thoughts or vice versa. In either case, fewer negative thought patterns likely help women recover from the traumas in their life, especially those related to sexual violence.

Enhanced Self-Worth After MAP Training

In the middle of the last century, Rogers (1954) published an instrument intended to quantify the discrepancy between images of an "ideal self" and one's "now" or "real self." Scores were negatively correlated with a host of other variables, e.g., symptoms of anxiety and depression, especially in women. They correlated with self-worth and responded to psychotherapy. In the present study, we used an updated measure, referred to as the Best Self Scale (Ogilvie and Clark, 1992). Before any training, low discrepancies scores correlated negatively with trauma-related thoughts and ruminations. Therefore, participants who rated

themselves as further from their best (ideal) self also reported more ruminative and post-traumatic thoughts and vice versa. Interestingly, women who completed MAP Training reported themselves as feeling closer to their "best selves" when compared to women who completed the same number of sessions of meditation alone or exercise alone. These results could be clinically important because women with trauma history often report negative cognitions about themselves, such as self-blame and guilt, which are sensitive to therapeutic change. For example, a recent study reported that fewer negative cognitions about the self were associated with fewer symptoms of PTSD when compared to thoughts about others or the world (Kumpula et al., 2017). Our results could reflect a change in body image in response to exercise, although exercise alone was not sufficient to significantly change the scores. In summary, the combination of meditation and aerobic exercise was similarly synergistic for self-worth as it was for ruminative and trauma-related thoughts.

Autobiographical Memories for Stressful Life Events

All participants were asked to reflect on the most stressful experience in their life and then to answer questions about the sensory details and emotions connected to the memory with the AMQ. In general, the strength of the stressful memory did not change after MAP Training nor did it change significantly after meditation or exercise alone. Interestingly, the strength of the memory and select sensory details associated with it did enhance in women who did not participate in any training. It is possible that trauma memories were reactivated by the reminder cues in the interview session (i.e., the SCID) or by filling out the questionnaires and continued to increase over the weeks between the two testing sessions. Because the strength of the memory did not increase in the trained groups, engaging in meditation or exercise, regardless of whether they were combined or not, was sufficient to keep the memories similar in strength to what they were before training. Overall, these data suggest MAP Training does not necessarily alter the quality of stressful memories as much as it does the quantities of intrusive thoughts and ruminations.

PTSD and Women With Sexual Violence History

Post-traumatic stress disorder is a mental disorder that can emerge after an extremely stressful life event, with serious repercussions for the person and society at large (Kessler, 2000). A person diagnosed with PTSD must have direct exposure, witness or learn of a traumatic event, which induces a combination of trauma-related thoughts and memories, negative thoughts about the self, others and the world, hypervigilance and heightened reactivity to environmental stimuli. These responses must persist and be accompanied by functional impairment. About a third (28%) of the women with SV history had current PTSD, whereas none of the women without SV history were similarly diagnosed. This is not surprising because women were recruited for SV history and trauma-related symptoms. Even so, many of the participants without SV history did report traumatic life experiences, such as car accidents, family deaths, physical abuse, etc. We did not conduct a structured interview after training and therefore do not know whether women with PTSD were less likely to be diagnosed as a result of MAP Training. Because MAP Training reduced post-traumatic cognitions, it is likely that many would have been less likely to incur a PTSD diagnosis. It has been reported that these negative cognitions fully mediate the severity of PTSD symptoms in women with SV history (Shin et al., 2017).

Mind- vs. Body-Focused Therapies for Trauma

Prolonged Exposure (PE) therapy is the most accepted evidence-based form of therapy for helping women recover from sexual violence and assault. This type of intervention evolved from animal learning studies of extinction. Through Pavlovian conditioning, animals including humans learn very quickly to associate a stressful event with its context and other cues that are present. These cues come to induce learned or "conditioned" responses in and of themselves. These learned responses often persist and may even become exaggerated over time. As such, they mimic some symptoms of PTSD. However, if the animal is repeatedly exposed to the cues (the conditioned stimulus), the learned response can lessen in magnitude and may even be extinguished altogether. Of course, this response is not forgotten and will often reemerge in another context or when presented with salient reminders. Nonetheless, the expression of fearful responses to conditioned stimuli can be reduced as the animal learns they do not necessarily predict a stressful event.

The purpose of PE is to reactivate fear networks and learn new associations with the conditioned stimuli. To do this, the client is guided through imagined, virtual and/or real-life exposure to traumatic memories, along with cognitive restructuring and at-home practice exercises. PE is often effective but has its share of detractors and problems (Pitman et al., 1991; Jaycox and Foa, 1996; Schnurr et al., 2007). As expected from learning theory, fear responses often reemerge when the client is confronted with trauma-related cues outside the therapeutic context. The main problem however is adherence, with nearly 40% drop-out rates in some studies (Hembree et al., 2003; Schnurr et al., 2007; Schottenbauer et al., 2008).

Other trauma interventions incorporate more body-centered approaches (Porges, 1995; van der Kolk et al., 2014). These approaches vary but generally involve some combination of meditation, body scanning and/or mindfulness exercises. Mindfulness-Based Stress Reduction (MBSR) (Davidson et al., 2003) incorporates mindfulness meditation with body scanning and yoga, whereas Mindfulness and Metta Based Trauma Therapy (MMTT) includes breathing meditation along with compassion mantras (Frewen et al., 2015). Their efficacy for PTSD are supported by clinical trials and meta-analyses (Metcalf et al., 2016; Cushing and Braun, 2017; Gallegos et al., 2017; Hilton et al., 2017). Some approaches focus specifically on the peripheral nervous system. For example, Polyvagal Theory (PVT) focuses on trauma-related processes which engage the vagus nerve (Sullivan et al., 2018). Somatic Experiencing®

(SE^{TM}) intervenes using interoceptive and somatic cues to restore balance of the autonomic nervous system (Payne et al., 2015; Briggs et al., 2018). Other therapies for trauma include Eye Movement Desensitization (EMDR), during which the client focuses attentional processes on external stimuli while engaging with memories of trauma (van der Kolk et al., 2007).

Group therapy and supportive counseling are not as efficacious as evidence-based therapies, but better than no treatment at all (Foa and Street, 2001; Sayin et al., 2013). Many individuals with PTSD are prescribed antidepressants and anxiolytic medications, sometimes augmented with anti-psychotics. Importantly, women with SV history reportedly prefer psychotherapy over pharmacotherapy (Feeny et al., 2009).

MAP Training as a Brain and Body Intervention

MAP Training activates both branches of the autonomic nervous system—sympathetic and parasympathetic—in one session. During meditation, the participant activates the parasympathetic nervous system to reduce heart rate and blood pressure, while increasing salivation and digestion. During aerobic exercise, heart rate increases dramatically through activation of the sympathetic nervous system, bringing blood and oxygen to the muscles and brain. These two systems interact in a healthy person to maintain balance between stress and recovery from stress. We propose here that extinction and/or exposure learning occurs while the participant meditates on her breath in a safe nonthreatening state and then consolidates the learning upon activation of the sympathetic nervous system during aerobic exercise. Theoretically, the combination of meditation and exercise during MAP Training is effective, at least in part because it activates both branches of the ANS.

The *MAP Training My Brain*^{TM} intervention was inspired by laboratory studies about neurogenesis, which is the production and survival of new neurons (Curlik and Shors, 2013; Shors et al., 2014). Early studies in animal models reported that many new neurons die within weeks unless new and effortful learning occurs (Gould et al., 1999; Shors, 2014). It was later reported that pubescent female rats exposed to an aggressive adult male did not learn well and as such retained fewer cells (Shors et al., 2016). Because learning and aerobic exercise increase these neurogenic processes, again in rodent models (DiFeo and Shors, 2017; Vivar and van Praag, 2017), we proposed that mental and physical training may be particularly effective when done together (Curlik and Shors, 2013; Shors et al., 2014). Importantly however, neurogenesis cannot be measured accurately in humans while they are alive and their detection in postmortem tissue is limited. Therefore, any meaningful connection to neurogenesis in humans is tenuous at best. But exercise and meditation have many effects throughout the hippocampus. For example, aerobic exercise reportedly increases the volume of the human hippocampus (Pereira et al., 2007; Erickson et al., 2011), with similar observations after MBSR (Hölzel et al., 2011). To be sure, meditation and aerobic exercise are generally beneficial for both brain and body. The point here was to develop a mind/body intervention that would help humans recover from traumatic experiences and the associated memories, especially women who have suffered sexual violence.

Dissemination of MAP Training

MAP Training significantly reduced trauma-related thoughts and ruminations while increasing self-worth in women with and without sexual violence history. Importantly, the combination of meditation plus exercise was especially effective for women with SV history whereas the individual components of exercise alone and meditation alone were not. We have previously reported similarly positive outcomes in individuals without preexisting conditions or trauma, thereby suggesting this intervention as a way to strengthen mental and physical health and perhaps even resilience against future traumas. It is not only effective; it is easy to learn, portable and relatively inexpensive to administer and do (see maptrainmybrain.com). Our next goal is to disseminate *MAP Training My Brain*^{TM} for others to use, either on its own or in conjunction with other therapies.

AUTHOR CONTRIBUTIONS

TS designed project. TS, EM, and HC carried out experiments, analyzed results, and wrote the manuscript.

FUNDING

Brain Health Institute, Rutgers University.

ACKNOWLEDGMENTS

We want to thank our research assistants Abigail Harriman, Christina Mesa, Katlyn Schroder, Ahua Traore, and Jacqueline Ugwuneri for their help with this project.

REFERENCES

Alderman, B. L., Olson, R. L., Brush, C. J., and Shors, T. J. (2016). MAP Training: combining meditation and aerobic exercise reduces depression and rumination while enhancing synchronized brain activity. *Transl. Psychiatry* 6, 1–9. doi: 10.1038/tp.2015.225

American Psychiatric Association (2013). *Diagnostic and Statistical Manual of Mental Disorders, 5th Edn*. Arlington, VA: American Psychiatric Association.

Booth, M. (2000). Assessment of physical activity: an international perspective. *Res. Q. Exerc. Sport* 71, S114–S120. doi: 10.1080/02701367.2000.11082794

Briggs, P., Hayes, S., and Changaris, M. (2018). Somatic Experiencing® informated therapeutic group for the care and treatment of biopsychosocial effects upon a gender diverse identity. *Front. Psychiatry* 9:53. doi: 10.3389/fpsyt.2018.00053

Carver, C. S., Lawrence, J. W., and Scheier, M. F. (1999). Self-discrepancies and affect: incorporating the role of feared selves. *Pers. Soc. Psychol. Bull* 25, 783–792.

Chisholm, D., Collis, M., Kulak, L., Davenport, W., and Gruber, N. (1975). Physical activity readiness. *BC Med. J.* 17, 375–378.

Clark, C. R., Paul, R. H., Williams, L. M., Arns, M., Fallahpour, K., Handmer, C., et al. (2006). Standardized assessment of cognitive functioning during

development and aging using an automated touchscreen battery. *Arch. Clin. Neuropsychol.* 21, 449–467. doi: 10.1016/j.acn.2006.06.005

Craig, C. L., Marshall, A. L., Sjöström, M., Bauman, A. E., Booth, M. L., Ainsworth, B. E., et al. (2003). International physical activity questionnaire: 12-country reliability and validity. *Med. Sci. Sports Exerc.* 35, 1381–1395. doi: 10.1249/01.MSS.0000078924.61453.FB

Curlik, D. M., and Shors, T. J. (2013). Training your brain: do mental and physical (MAP) training enhance cognition through the process of neurogenesis in the hippocampus? *Neuropharmacology* 64, 506–514. doi: 10.1016/j.neuropharm.2012.07.027

Curlik, D. M., Maeng, L. Y., Agarwal, P. R., and Shors, T. J. (2013). Physical skill training increases the number of surviving new cells in the adult hippocampus. *PLoS ONE* 8:e55850. doi: 10.1371/journal.pone.0055850

Cushing, R. E., and Braun, K. L. (2017). Mind–body therapy for military veterans with post-traumatic stress disorder: a systematic review. *J. Altern. Complement. Med.* 24, 106–114. doi: 10.1089/acm.2017.0176

Davidson, R. J., Kabat-Zinn, J., Schumacher, J., Rosenkranz, M., Muller, D., Santorelli, S. F., et al. (2003). Alterations in brain and immune function produced by mindfulness meditation. *Psychosom. Med.* 65, 564–570. doi: 10.1097/01.PSY.0000077505.67574.E3

DiFeo, G., and Shors, T. J. (2017). Mental and physical skill training increases neurogenesis via cell survival in the adolescent hippocampus. *Brain Res.* 1654, 95–101. doi: 10.1016/j.brainres.2016.08.015

Erickson, K. I., Voss, M. W., Prakash, R. S., Basak, C., Szabo, A., Chaddock, L., et al. (2011). Exercise training increases size of hippocampus and improves memory. *Proc. Natl. Acad. Sci. U.S.A.* 108, 3017–3022. doi: 10.1073/pnas.1015950108

Farb, N. A. S., Segal, Z. V., Mayberg, H., Bean, J., McKeon, D., Fatima, Z., et al. (2007). Attending to the present: mindfulness meditation reveals distinct neural modes of self-reference. *Soc. Cogn. Affect. Neurosci.* 2, 313–322. doi: 10.1093/scan/nsm030

Feeny, N. C., Zoellner, L. A., and Kahana, S. Y. (2009). Providing a treatment rationale for PTSD: does what we say matter? *Behav. Res. Ther.* 47, 752–760. doi: 10.1016/j.brat.2009.06.007

First, M. B., Williams, J. B. W., Karg, R. S., and Spitzer, R. L. (2015). *Structured Clinical Interview for DSM-5 (SCID-5), Research Version*. Arlington, VA: American Psychiatric Association.

Foa, E. B., and Rauch, S. A. M. (2004). Cognitive changes during prolonged exposure versus prolonged exposure plus cognitive restructuring in female assault survivors with posttraumatic stress disorder. *J. Consult. Clin. Psychol.* 72, 879–884. doi: 10.1037/0022-006X.72.5.879

Foa, E. B., and Street, G. P. (2001). Women and traumatic events. *J. Clin. Psychiatry* 62(Suppl. 17), 29–34.

Foa, E. B., Ehlers, A., Clark, D. M., Tolin, D. F., and Orsillo, S. M. (1999). The Posttraumatic Cognitions Inventory (PTCI): development and validation. *Psychol. Assess.* 11, 303–314. doi: 10.1037/1040-3590.11.3.303

Foa, E. B., Zoellner, L. A., Feeny, N. C., Hembree, E. A., and Alvarez-Conrad, J. (2002). Does imaginal exposure exacerbate PTSD symptoms? *J. Consult. Clin. Psychol.* 70, 1022–1028. doi: 10.1037/0022-006X.70.4.1022

Frewen, P., Rogers, N., Flodrowski, L., and Lanius, R. (2015). Mindfulness and metta-based trauma therapy (MMTT): initial development and proof-of-concept of an internet resource. *Mindfulness* 6, 1322–1334. doi: 10.1007/s12671-015-0402-y

Gallegos, A. M., Crean, H. F., Pigeon, W. R., and Heffner, K. L. (2017). Meditation and yoga for posttraumatic stress disorder: a meta-analytic review of randomized controlled trials. *Clin. Psychol. Rev.* 58, 115–124. doi: 10.1016/j.cpr.2017.10.004

Gould, E., Beylin, A., Tanapat, P., Reeves, A., and Shors, T. (1999). Learning enhances adult neurogenesis in the hippocampal formation. *Nat. Neurosci.* 2, 260–265. doi: 10.1038/6365

Hach, S., Tippett, L. J., and Addis, D. R. (2014). Neural changes associated with the generation of specific past and future events in depression. *Neuropsychologia* 65, 41–55. doi: 10.1016/j.neuropsychologia.2014.10.003

Hembree, E. A., Foa, E. B., Dorfan, N. M., Street, G. P., Kowalski, J., and Tu, X. (2003). Do patients drop out prematurely from exposure therapy for PTSD? *J. Trauma. Stress* 16, 555–562. doi: 10.1023/B:JOTS.0000004078.93012.7d

Hilton, L., Maher, A. R., Colaiaco, B., Apaydin, E., Sorbero, M. E., Booth, M., et al. (2017). Meditation for posttraumatic stress: systematic review and meta-analysis. *Psychol. Trauma* 9, 453–460. doi: 10.1037/tra0000180

Hölzel, B. K., Carmody, J., Vangel, M., Congleton, C., Yerramsetti, S. M., Gard, T., et al. (2011). Mindfulness practice leads to increases in regional brain gray matter density. *Psychiatry Res.* 191, 36–43. doi: 10.1016/j.pscychresns.2010.08.006

Jaycox, L. H., and Foa, E. B. (1996). Obstacles in implementing exposure therapy for PTSD: case discussions and practical solutions. *Clin. Psychol. Psychother.* 3, 176–184. doi: 10.1002/(SICI)1099-0879(199609)3:3<176::AID-CPP100>3.0.CO;2-1

Johnston, B. A., Tolomeo, S., Gradin, V., Christmas, D., Matthews, K., and Steele, J. D. (2015). Failure of hippocampal deactivation during loss events in treatment-resistant depression. *Brain* 138, 2766–2776. doi: 10.1093/brain/awv177

Joiner, T. E., Walker, R. L., Rudd, M. D., and Jobes, D. A. (1999). Scientizing and routinizing the assessment of suicidality in outpatient practice. *Prof. Psychol. Res. Pract.* 30, 447–453. doi: 10.1037/0735-7028.30.5.447

Kessler, R. C. (2000). Posttraumatic stress disorder: the burden to the individual and to society. *J. Clin. Psychiatry* 61(Suppl 5), 4–14.

Kessler, R., Aguilar-Gaxiola, S., Alonso, J., Benjet, C., Bromet, E., Cardoso, G., et al. (2017). Trauma and PTSD in the WHO world mental health surveys. *Eur. J. Psychotraumatol.* 8:1353383. doi: 10.1080/20008198.2017.1353383

Kumpula, M. J., Pentel, K. Z., Foa, E. B., LeBlanc, N. J., Bui, E., McSweeney, L. B., et al. (2017). Temporal sequencing of change in posttraumatic cognitions and PTSD symptom reduction during prolonged exposure therapy. *Behav. Ther.* 48, 156–165. doi: 10.1016/j.beth.2016.02.008

Lutz, A., Slagter, H. A., Dunne, J. D., and Davidson, R. J. (2008). Attention regulation and monitoring in meditation. *Trends Cogn. Sci.* 12, 163–169. doi: 10.1016/j.tics.2008.01.005

Metcalf, O., Varker, T., Forbes, D., Phelps, A., Dell, L., DiBattista, A., et al. (2016). Efficacy of fifteen emerging interventions for the treatment of posttraumatic stress disorder: a systematic review. *J. Trauma. Stress* 29, 88–92. doi: 10.1002/jts.22070

Nadel, L., and Hardt, O. (2011). Update on memory systems and processes. *Neuropsychopharmacology* 36, 251–273. doi: 10.1038/npp.2010.169

Nokia, M. S., Lensu, S., Ahtiainen, J. P., Johansson, P. P., Koch, L. G., Britton, S. L., et al. (2016). Physical exercise increases adult hippocampal neurogenesis in male rats provided it is aerobic and sustained. *J. Physiol.* 594, 1855–1873. doi: 10.1113/JP271552

Ogilvie, D., and Clark, M. (1992). "The best and worst of it: age and sex differences in self-discrepancy research," in *Self-perspectives Across the Life Span*, eds R. P. Albany Lipka and T. M. Brinthaupt (New York, NY: SUNY Press), 186–222.

Ozer, E. J., Best, S. R., Lipsey, T. L., and Weiss, D. S. (2003). Predictors of posttraumatic stress disorder and symptoms in adults: a meta-analysis. *Psychol. Bull.* 129, 52–73. doi: 10.1037/0033-2909.129.1.52

Payne, P., Levine, P. A., and Crane-Godreau, M. A. (2015). Somatic Experiencing: using interoception and proprioception as core elements of trauma therapy. *Front. Psychol.* 6:93. doi: 10.3389/fpsyg.2015.00093

Pereira, A. C., Huddleston, D. E., Brickman, A. M., Sosunov, A. A., Hen, R., McKhann, G. M., et al. (2007). An *in vivo* correlate of exercise-induced neurogenesis in the adult dentate gyrus. *Proc. Natl. Acad. Sci. U.S.A.* 104, 5638–5643. doi: 10.1073/pnas.0611721104

Pitman, R. K., Altman, B., Greenwald, E., Longpre, R. E., Macklin, M. L., Poiré, R. E., et al. (1991). Psychiatric complications during flooding therapy for posttraumatic stress disorder. *J. Clin. Psychiatry* 52, 17–20.

Porges, S. W. (1995). Orienting in a defensive world: mammalian modifications of our evolutionary heritage. a polyvagal theory. *Psychophysiology* 32, 301–318. doi: 10.1111/j.1469-8986.1995.tb01213.x

Resick, P. A., Nishith, P., Weaver, T. L., Astin, M. C., and Feuer, C. A. (2002). A comparison of cognitive-processing therapy with prolonged exposure and a waiting condition for the treatment of chronic posttraumatic stress disorder in female rape victims. *J. Consult. Clin. Psychol.* 70, 867–879. doi: 10.1037/0022-006X.70.4.867

Rogers. C. R. (1954). "The case of Mrs. Oaks: a research analysis," in *Psychotherapy and Personality Change: Coordinated Research Studies in the Client-Centered Approach*, eds C. R. Rogers and R. F. Dymond (Chicago, IL: Chicago University Press), 259–348.

Rubin, D. C., Dennis, M. F., and Beckham, J. C. (2011). Autobiographical memory for stressful events: the role of autobiographical memory in posttraumatic stress disorder. *Conscious. Cogn.* 20, 840–856. doi: 10.1016/j.concog.2011.03.015

Rubin, D. C., Schrauf, R. W., and Greenberg, D. L. (2003). Belief and recollection of autobiographical memories. *Mem. Cogn.* 31, 887–901. doi: 10.3758/BF03196443

Sadock, B. J., Sadock, V. A., and Ruiz, P. (2014). *Synopsis of Psychiatry: Behavioral Sciences/Clinical Psychiatry*, *11th Edn*. Philadelphia, PA: Wolters Kluwer.

Sayin, A., Candansayar, S., and Welkin, L. (2013). Group psychotherapy in women with a history of sexual abuse: what did they find helpful? *J. Clin. Nurs.* 22, 3249–3258. doi: 10.1111/jocn.12168

Schnurr, P. P., Friedman, M. J., Engel, C. C., Foa, E. B., Shea, M. T., Chow, B. K., et al. (2007). Cognitive behavioral therapy for posttraumatic stress disorder in women: a randomized controlled trial. *JAMA* 297, 820–830. doi: 10.1001/jama.297.8.820

Schottenbauer, M. A., Glass, C. R., Arnkoff, D. B., Tendick, V., and Gray, S. H. (2008). Nonresponse and dropout rates in outcome studies on PTSD: review and methodological considerations. *Psychiatry Interpers. Biol. Process* 71, 134–168. doi: 10.1521/psyc.2008.71.2.134

Shin, K. M., Chung, Y. K., Shin, Y. J., Kim, M., Kim, N. H., Kim, K. A., et al. (2017). Post-traumatic cognition mediates the relationship between a history of sexual abuse and the post-traumatic stress symptoms in sexual assault victims. *J. Korean Med. Sci.* 32, 1680–1686. doi: 10.3346/jkms.2017.32.10.1680

Shors, T. J. (2009). Saving new brain cells. *Sci. Am.* 300, 46–54. doi: 10.1038/scientificamerican0309-46

Shors, T. J. (2014). The adult brain makes new neurons, and effortful learning keeps them alive. *Curr. Dir. Psychol. Sci.* 23, 311–318. doi: 10.1177/0963721414540167

Shors, T. J., and Millon, E. M. (2016). Sexual trauma and the female brain. *Front. Neuroendocrinol.* 41, 87–98. doi: 10.1016/j.yfrne.2016.04.001

Shors, T. J., Millon, E. M., Chang, H. Y. M., Olson, R. L., and Alderman, B. L. (2017). Do sex differences in rumination explain sex differences in depression? *J. Neurosci. Res.* 95, 711–718. doi: 10.1002/jnr.23976

Shors, T. J., Olson, R. L., Bates, M. E., Selby, E. A., and Alderman, B. L. (2014). Mental and Physical (MAP) Training: a neurogenesis-inspired intervention that enhances health in humans. *Neurobiol. Learn. Mem.* 115, 3–9. doi: 10.1016/j.nlm.2014.08.012

Shors, T. J., Tobón, K., DiFeo, G., Durham, D. M., and Chang, H. Y. M. (2016). Sexual Conspecific Aggressive Response (SCAR): a model of sexual trauma that disrupts maternal learning and plasticity in the female brain. *Sci. Rep.* 6:18960. doi: 10.1038/srep18960

Stein, P. K., Kleiger, R. E., and Rottman, J. N. (1997). Differing effects of age on heart rate variability in men and women. *Am. J. Cardiol.* 80, 302–305. doi: 10.1016/S0002-9149(97)00350-0

Sullivan, M. B., Erb, M., Schmalzl, L., Moonaz, S., Noggle Taylor, J., and Porges, S. W. (2018). Yoga therapy and polyvagal theory: the convergence of traditional wisdom and contemporary neuroscience for self-regulation and resilience. *Front. Hum. Neurosci.* 12:67. doi: 10.3389/fnhum.2018.00067

Treynor, W., Gonzalez, R., and Nolen-Hoeksema, S. (2003). Rumination reconsidered: a psychometric analysis. *Cognit. Ther. Res.* 27, 247–259. doi: 10.1023/A:1023910315561

van der Kolk, B. A., Spinazzola, J., Blaustein, M. E., Hopper, J. W., Hopper, E. K., Korn, D. L., et al. (2007). A randomized clinical trial of eye movement desensitization and reprocessing (EMDR), fluoxetine, and pill placebo in the treatment of posttraumatic stress disorder: treatment effects and long-term maintenance. *J. Clin. Psychiatry* 68, 37–46. doi: 10.4088/JCP.v68n0105

van der Kolk, B. A., Stone, L., West, J., Rhodes, A., Emerson, D., Suvak, M., et al. (2014). Yoga as an adjunctive treatment for posttraumatic stress disorder: a randomized controlled trial. *J. Clin. Psychiatry* 75, e559–e565. doi: 10.4088/JCP.13m08561

van Praag, H., Kempermann, G., and Gage, F. H. (1999). Running increases cell proliferation and neurogenesis in the adult mouse dentate gyrus. *Nat. Neurosci.* 2, 266–270. doi: 10.1038/6368

Vivar, C., and van Praag, H. (2017). Running changes the brain: the long and short of it. *Physiology* 32, 410–424. doi: 10.1152/physiol.00017.2017

World Health Organization (2013). *Global and Regional Estimates of Violence Against Women: Prevalence and Health Effects of Intimate Partner Violence and Nonpartner Sexual Violence* (Geneva: World Health Organization), 1–50.

Conflict of Interest Statement: The authors declare that the research was conducted in the absence of any commercial or financial relationships that could be construed as a potential conflict of interest.

Copyright © 2018 Shors, Chang and Millon. This is an open-access article distributed under the terms of the Creative Commons Attribution License (CC BY). The use, distribution or reproduction in other forums is permitted, provided the original author(s) and the copyright owner are credited and that the original publication in this journal is cited, in accordance with accepted academic practice. No use, distribution or reproduction is permitted which does not comply with these terms.
