# Supplementary material for: Does physical activity-based intervention decrease repetitive negative thinking? A systematic review
Source: PLoS One. 2025 Apr 1;20(4):e0319806. doi: 10.1371/journal.pone.0319806 (PMC11960971; doi:10.1371/journal.pone.0319806)
Supplement: S1 File — https://doi.org/10.6084/m9.figshare.25711734. (ZIP) [file pone.0319806.s001.zip › supporting information/paper file/Schuver 2016.pdf]

Complementary Therapies in Medicine 26 (2016) 85–91

Contents lists available at ScienceDirect

Complementary Therapies in Medicine

journal homepage: www.elsevierhealth.com/journals/ctim

Mindfulness-based yoga intervention for women with depression

Katie J. Schuver[a,*], Beth A. Lewis[b]

[a] University of Minnesota, School of Kinesiology and Center for Spirituality & Healing, 1900 University Ave SE & 420 Delaware St. SE C592, Minneapolis, MN 55455, USA
[b] University of Minnesota, School of Kinesiology, 1900 University Ave SE, Minneapolis, MN 55455, USA

ARTICLE INFO

Article history:
Received 16 December 2015
Received in revised form 3 March 2016
Accepted 4 March 2016
Available online 14 March 2016

Keywords:
Depression
Hatha yoga
Mindfulness

ClinicalTrials.gov identifier:
NCT02630758

ABSTRACT

Objectives: The purpose of this study was to examine the efficacy of a 12-week mindfulness-based yoga intervention on depressive symptoms and rumination among depressed women.
Design: Prospective, randomized, controlled 12 week intervention pilot study. Depressive symptoms were assessed at baseline, post-intervention (12 weeks), and one-month follow-up.
Setting: Women with a history of diagnosed depression and currently depressed were randomized to a mindfulness-based yoga condition or a walking control.
Interventions: The mindfulness-based yoga intervention consisted of a home-based yoga asana, pranayama and meditation practice with mindfulness education sessions delivered over the telephone. The walking control condition consisted of home-based walking sessions and health education sessions delivered over the phone.
Main outcome measures: The Beck Depression Inventory (BDI) and Ruminative Responses Scale (RRS).
Results: Both groups reported decreases in depressive symptoms from baseline to post-intervention, $f(1,33) = 34.83$, $p < 0.001$, and from baseline to one-month follow-up, $f(1,33) = 37.01$, $p < 0.001$. After controlling for baseline, there were no significant between group differences on depression scores at post-intervention and the one-month follow-up assessment. The mindfulness-based yoga condition reported significantly lower levels of rumination than the control condition at post-intervention, after controlling for baseline levels of rumination, $f(1,31) = 6.23$, $p < 0.01$.
Conclusions: These findings suggest that mindfulness-based yoga may provide tools to manage ruminative thoughts among women with elevated depressive symptoms. Future studies, with larger samples are needed to address the effect of yoga on depression and further explore the impact on rumination.
© 2016 Elsevier Ltd. All rights reserved.

1. Introduction

Depression is the most prevalent of all psychiatric disorders affecting up to 25% of women and 12% of men during their lifetimes.[1–3] Over the next 20 years, depression is projected to be the leading cause of disability in the United States.[4] Depression is commonly treated with antidepressants or psychotherapy, or a combination of both antidepressants and psychotherapy. Both are effective for treating depression[5]; however, a recent review found high dropout rates, low remission rates, and placebo responses for these therapies.[6–9]

1.1. *Efficacy of mindfulness-based hatha yoga interventions for depression*

Preliminary findings indicate that Yoga and mindfulness (i.e., meditation) may be an effective ancillary intervention option for the treatment of depression.[10–13] The increased development of mindfulness[14,15] and physical components (yoga postures)[16] have been proposed as the mechanism for decreasing depression. The literature examining the effect of yoga and mindfulness-based therapies on depression has been criticized methodologically.[10,17–20] Limitations included short interventions, small sample sizes, no randomization in some studies, lack of standardized interviews to diagnose depression, and some studies lacked a control or comparison group.

The purpose of this study was to examine the efficacy of a mindfulness-based yoga intervention for the reduction of depressive symptoms among adult women. Women with depression were randomized to a mindfulness-based yoga intervention or a walk-

* Corresponding author.
E-mail address: schuv007@umn.edu (K.J. Schuver).

http://dx.doi.org/10.1016/j.ctim.2016.03.003
0965-2299/© 2016 Elsevier Ltd. All rights reserved.

ing control group. Our study improved upon previous research by addressing some of the most significant methodological issues in previous studies including lack of standardized interviews to diagnose depression, use of non-standardized depression outcome measures, short treatment length, lack of a control or comparison group, and lack of follow-up assessments. Hatha yoga was chosen because it is the most widely practiced style of yoga in the United States.[20] Adult women were targeted for this study given they are two times more likely than men to report major depressive symptoms[1] and the majority of individuals practicing yoga in the United States are women.[21,22] Walking was chosen as the control condition given it has been shown to be a comparable MET-matched activity to hatha yoga. We hypothesized that women in the mindfulness-based yoga intervention would exhibit greater decreases in depressive symptoms (based on the Beck Depression Inventory) and rumination (based on the Ruminative Responses Scale) than participants in the walking control condition.

2. Methods

2.1. Overview of study design

This study was a prospective, randomized controlled intervention pilot study conducted in a metropolitan city in the upper Midwest of the United States. Forty women who met the criteria for depression based on the Structured Clinical Interview for Diagnostic and Statistical Manual of Mental Disorders IV (SCID-I)[23] were randomized to an experimental mindfulness-based yoga condition (n = 20) or a walking control condition (n = 20). Both programs were home-based motivational sessions delivered over the telephone and lasted 12 weeks. Assessments were conducted at baseline, post-intervention (i.e., three months following baseline), one-month follow-up (i.e., one month after the intervention ended). Participants were instructed to continue their usual depression care. The primary dependent variable was depressive symptoms based on the Beck Depression Inventory (BDI). The secondary dependent variable was rumination scores on the Ruminative Responses Scale (RRS). This protocol was approved by the University of Minnesota's Institutional Review Board (IRB). Participants completed informed consent forms sent through the mail; consent to participate in physical activity was also obtained from the participant's healthcare provider. Recruitment for this trial occurred from February 2013 to January 2014 and follow-up assessments were completed by May 2014.

2.2. Participants

Forty women between the ages of 20 and 64 were recruited and randomly assigned to one of the two study conditions. Participants were recruited primarily online using targeted emails through a local newspaper (email was sent to individuals who had given their email address to the newspaper) and Craig's List (i.e., online classifieds). The overall recruitment into the study is summarized in Fig. 1 and demographic information for the sample is summarized in Table 1.

2.3. Eligibility criteria

Inclusion criteria were assessed via a telephone screening interview and included: (1) 18 years of age or older; (2) a personal history of depression (i.e., ever being told by a healthcare provider that they had depression or had been given an antidepressant for depression); (3) able to read and write in the English language; (4) able to commit to two sessions (yoga or walking) per week for 12 weeks; (5) demonstrate a willingness to be randomly assigned to either of the study arms; and (6) be yoga naïve, defined as not taking

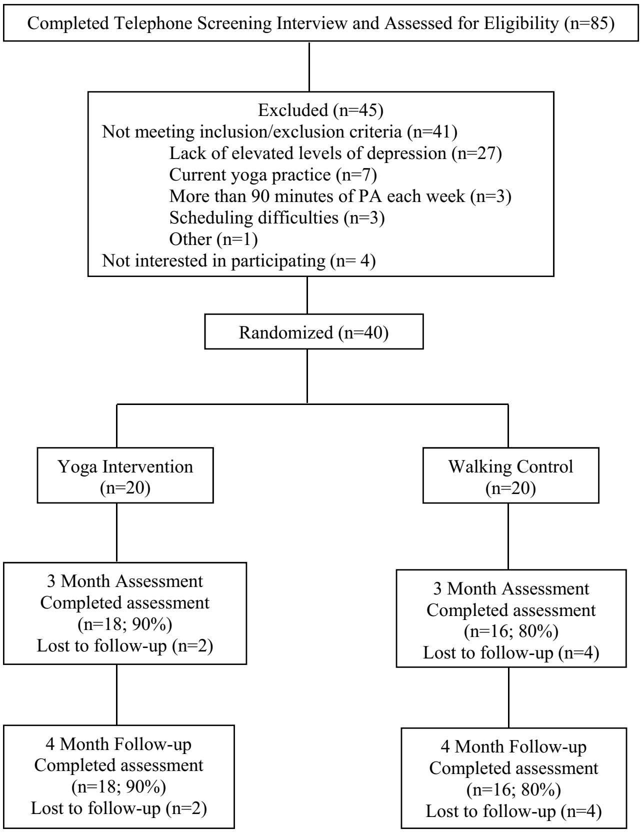

Fig. 1. Sampling and flow of participants from January 2013-May 2014.

more than four yoga classes in the past two years and not more than one class in the past month. The Structured Clinical Interview for Diagnostic and Statistical Manual of Mental Disorders IV was used for screening purposes to identify a depressive episode.[23] All participants met the criteria for major depressive disorder. Additionally, at the time of eligibility screening, participants needed to have a score of ≥14 on the Beck Depression Inventory,[24] which is indicative of at least mild to moderate levels of depressive symptoms. Participants currently using other forms of treatment for depressive symptoms (i.e., pharmacotherapy and/or psychotherapy) were considered eligible for the study, assuming there were no changes in antidepressant medications and/or dosing during the previous month or any expected changes during the intervention period.

Specific exclusion criteria included: (1) Individuals with a history of bipolar disorder or schizophrenia and/or who had been hospitalized for a psychiatric disorder within six months prior to the eligibility screening; (2) individuals not providing signed informed consent; (3) individuals reporting any other health problems that would interfere with regular yoga practice or walking sessions; and (4) individuals who engaged in more than 90 min of moderate intensity physical activity per week during the month prior to the eligibility screening.

2.4. Randomization

Participants were randomly assigned in a 1:1 ratio to either the mindfulness-based yoga intervention or walking control condition utilizing a random numbers table generated by excel. Participants received an explanation of each group prior to randomization and only participants who agreed to participate in either of the two groups were randomized. At the end of the randomization session, participants were told by the primary investigator which group they had been randomly assigned to. The primary investigator

Table 1
Baseline characteristics by study condition.

Variable	Total sample (n = 40)	Yoga (n = 20)	Walking Control (n = 20)	P-value
Age (average in years)	42.68(4.95)	45.55(12.30)	39.8(11.23)	0.13
Race (%)				0.12
Caucasion	80.0%	90.0%	70.0%	
American-Indian	2.5%	0.0%	5.0%	
Asian	2.5%	0.0%	5.0%	
African-American	10.0%	10.0%	10.0%	
Other	5.0%	0.0%	10.0%	
Ethnicity (%)				0.55
Non-hispanic	95.0%	100.0%	95.0%	
Hispanic	5.0%	0.0%	5.0%	
Marital Status (% Married)	42.5%	50.0%	35.0%	0.35
Education (% College Grad)	52.5%	45.0%	60.0%	0.35
High School Graduate	7.5%	5.0%	10.0%	
Some College	40.0%	50.0%	30.0%	
College Graduate	32.5%	35.0%	30.0%	
Post-graduate Work	20.0%	10.0%	30.0%	
Income (% over $40,000)	57.5%	60.0%	55.0%	0.76
Under 10,000	15.0%	15.0%	15.0%	
Between $10,000–19,999	7.5%	10.0%	5.0%	
Between $20,000–29,999	17.5%	10.0%	25.0%	
Between $30,000–39,999	2.5%	5.0%	0.0%	
Between $40,000–49,999	12.5%	10.0%	15.0%	
Over $50,000	45.0%	50.0%	40.0%	
Currently Employed (%)	77.5%	85.0%	70.0%	0.27
1+ Children Under 18years (%)	30.0%	25.0%	35.0%	0.50
Family History of Depression (%)	80.0%	20.0%	20.0%	1.00
Current Antidepressant Usage(%)	52.5%	45.0%	60.0%	0.36
Current Psychotherapy	2.5%	5.0%	0.0%	0.16
BDI	26.35(5.50)	26.20(6.60)	26.50(4.31)	0.87

Note. BDI = Beck Depression Inventory.

administering the randomization and counseling sessions was not involved in post-intervention or follow-up data collection. Additionally, the research assistant conducting the post-intervention and follow-up assessment session was blinded to the participant's treatment assignment.

2.5. Measures

2.5.1. Primary dependent variable: beck depression inventory (BDI)

The BDI was used to measure depressive symptoms and was the primary dependent variable for this study.[24] The BDI is a 21-item questionnaire that addresses the severity of both cognitive and somatic aspects of depression and is a commonly used self-report scale for the measurement of depressive symptoms. The BDI serves as a standard measure of the presence and severity of depressive symptoms as consistent with the criteria of the DSM-IV-TR.[25] A meta-analysis of the BDI's internal consistency indicated a mean coefficient alpha of 0.81 for non-psychiatric individuals.[26]

2.5.2. Secondary dependent measure: the ruminative responses scale (RSS)

The RSS was used to measure ruminating thoughts.[27] The RRS is a 22-item self-report questionnaire that examines reflection, brooding, and depression-related characteristics. The RRS has demonstrated high internal validity, with Cronbach's α ranging from 0.88 to 0.92.[27–30]

2.6. Procedure

Potential participants were screened for eligibility using a standard script to determine if they met the inclusion and/or exclusion criteria. Existence and severity of depressive symptoms was assessed at baseline using the BDI[24] and the SCID.[23] Participants were also given information regarding the study, including the voluntary nature of the study and assurance of confidential procedures. Following determination of eligibility and interest, the participant was sent an informed consent letter, along with a demographics form to be signed, completed, and returned prior to randomization. The participant was instructed to return this consent form within one week to minimize time between screening and randomization.

Once written consent was obtained from the participant, baseline assessments were completed and the participant was randomized into the mindfulness-based yoga (i.e., yoga) or walking control condition. Following the 12-week intervention period, participants in both the mindfulness-based yoga group and walking control group completed the post-intervention assessment questionnaires over the telephone. The follow-up assessment was completed one month later (four months from the time of randomization). Questionnaires administered at baseline were repeated at both post-intervention and one-month follow-up assessments. Assessments were completed by an individual trained in administering all questionnaires and who was blinded to the participants' group assignment. Upon completion of the one-month follow-up assessment, each participant was sent a thank you letter and a $25.00 gift card for their participation in the study.

2.7. Mindfulness-based yoga condition

Participants in the 12-week mindfulness-based yoga condition were guided by a gentle yoga DVD that included asana (postures), pranayama (breathing exercises), and relaxation (meditation). The DVD was a commercially developed yoga program specifically designed for the management of depression and anxiety symptoms.[31] Participants were asked to complete 60–75 min of the DVD twice per week and were encouraged to do more if they were interested. Once they were familiar with the activities in the

Table 2
Timeline and content of mindfulness-based telephone sessions.

Session	Content	Mindfulness Principle Focus
1	Overview of yoga intervention/assessing initial questions on yoga & preparing plan for practicing yoga at home	Mindful Movement Strategies
2	History of yoga & introduction to mindfulness	Overview of all 7 principles- Non-Judging, Patience, Beginner's Mind, Trust, Non-striving, Acceptance, Letting Go
3	Introduction to mindful breathing practices	Non-Judging, Patience, Beginner's Mind, Trust, Non-striving, Acceptance, Letting Go
4	Mindfulness of mind (thoughts, judgments, reactions) & body-scan & observation journals	Trust, Non-Judging, Acceptance, Letting Go
5	Mindfulness of body, mind, thoughts, emotions in everyday activities-mindful eating exercise	Patience, Trust, Acceptance, Beginner's Mind
6	Practice of mindfulness of mind-sitting meditation	Letting Go, Patience, Non-striving, Acceptance
7	Mindfulness of mind-nine dots exercise & mindfulness lists	Beginner's Mind, Letting Go
8	Overview of mindfulness skills & wrap-up	Review all 7 Principles

Note. Sessions 1–4 were scheduled weekly during the first month of the intervention. Sessions 5–8 were scheduled bi-weekly during the second and third month of the intervention.

DVD, participants were allowed to do the practices on their own (self-led). Questions and/or concerns regarding the practices introduced in the DVD were addressed during the telephone sessions. Weekly yoga frequency and time was reported and recorded at each phone session.

Following the initial baseline assessment and randomization telephone interview, participants in the yoga group completed weekly 15-min telephone sessions for the first month and bi-weekly telephone sessions for the second and third months for a total of eight sessions over the 12 weeks. In general, each session started with a brief check-in regarding the previous week's experience with the yoga DVD and mindfulness-based goal. The participant reported the frequency and duration of the week's yoga practice. Then the current week's mindfulness topic was introduced and discussed. Before ending the call, the participant was asked to set a goal for implementing the week's mindfulness theme into their daily life and a goal related to their yoga practice with the DVD.

The mindfulness telephone sessions were modified from the Mindfulness-Based Stress Reduction program[32] which has demonstrated to be effective for managing depressive[33–35] and anxiety symptoms[35–37] ; in addition to improvement of general mental health.[38] Table 2 summarizes the topics discussed at each mindfulness-based telephone session.

2.8. Walking control condition

The 12-week walking control condition included twice-weekly home practice with a 65-min walking DVD[39] and eight telephone

Table 3
Timeline and content of wellness telephone sessions.

Session	Content
1	Overview of walking intervention/assessing initial questions & preparing plan for walking at home
2	Understanding stress
3	Stress prevention & management
4	Time management
5	Sleep management
6	Developing/maintaining strategies for safe weight loss and weight gain prevention
7	Nutrition overview & superfoods
8	Review of stress prevention and management and wrap-up

Note. Sessions 1–4 were scheduled weekly during the first month. Sessions 5–8 were scheduled bi-weekly during the second and third month.

sessions with the telephone counselor. The DVD guided participants through a walking-based workout that could be completed in a single room at home. Specifically, participants walked forwards and backwards based on the video that was set to music. Participants were asked to use a calendar to track walking and any other physical activity. Weekly walking frequency and time were reported and recorded at each telephone session.

Participants were asked to complete 60 min of the DVD (or other walking) twice weekly and encouraged to do more if they were interested. Questions and/or concerns regarding walking were addressed during the telephone sessions.

Participants received telephone sessions on the same schedule as the yoga condition. In general, each session started with a brief check-in regarding the previous week's experience with the walking DVD and health-related goal. The participant reported the frequency and duration of the week's walking. Then the current week's health-related topic was introduced and discussed. Before ending the call, the participant was asked to set a wellness-related goal. The education sessions covered a variety of health and wellness related topics (see Table 3).

2.9. Data analysis

Independent *t*-tests for the continuous dependent variables and chi-square tests for categorical dependent variables were used to examine between group differences on the demographic variables. Between groups analysis of covariance (ANCOVA) was used to examine the effect of the intervention on the BDI (primary dependent variable), and the secondary variable of interest, RRS at post-intervention and one-month follow-up, controlling for baseline level of depressive symptoms and rumination. To examine the magnitude of the difference between the mean scores of each variable for the two conditions, effect sizes were calculated using Cohen's D. ANCOVA's were also used to examine between group differences on retention and adherence. All data were analyzed using SPSS (v21.0) for Windows and Microsoft Excel (Windows 2010). The statistical significance level was set at 0.05.

3. Results

3.1. Participants

Recruitment and randomization are summarized in Fig. 1. The final sample consisted of 40 women with depression. The retention rate (defined as completing both the post-intervention and

one-month follow-up) was 90% for the mindfulness-based yoga condition and 80% for the walking control condition.

The demographic data by study arms are summarized in Table 1. There were no between groups differences for any of the demographic or baseline variables. Additionally there were no differences on the demographic variables between study completers (n = 34) and dropouts (n = 6) with the exception of marital status $f(1,39) = 5.70$, ($p < 0.05$). Specifically, 50% of study completers reported being married whereas none of the non-completers were married. All participants who were taking an antidepressant at baseline continued their antidepressant use at post-intervention and the one-month follow-up. Additionally, none of the participants who were not taking an antidepressant at baseline reported taking an antidepressant at post-intervention or the one-month follow-up.

3.2. Retention and adherence

The recommended "dose" of the mindfulness-based yoga or walking was minimum twice-weekly sessions or approximately 120 min per week of the assigned activity. Participants in the mindfulness-based yoga participated in yoga 2.30 ($sd = 1.03$) times a week and 50.25 ($sd = 17.05$) minutes per session for a total of 119.75 ($sd = 58.95$) minutes per week on average during the three month intervention. Participants in the walking group engaged in walking sessions 3.55 ($sd = 3.22$) times a week and 23.90 ($sd = 14.25$) minutes per session for a total of 78.25 ($sd = 52.50$) minutes per week on average during the three month intervention. The yoga condition reported more minutes per session, $f(1,39) = 28.7$, $p < 0.001$; $d = 1.67$, and more weekly minutes of assigned activity, $f(1,39) = 5.53$, $p < 0.05$; $d = 0.74$, than the walking condition during the three month intervention. At the one-month follow-up, participants in the yoga condition reported more assigned activity during the assessment week than the walking condition, $f(1,39) = 4.228$, $p < 0.05$; $d = 0.65$. Specifically, yoga participants reported an average of 105.00 ($sd = 50.52$) minutes per week of yoga activity and walking participants reported 72.00 ($sd = 50.96$) minutes per week of walking.

Participants in the mindfulness-based yoga condition completed a mean of 6.25 ($sd = 2.19$) telephone counseling sessions (out of eight) with a duration of 17.10 ($sd = 5.65$) minutes per session on average during the three month intervention. Participants in the walking control condition completed an average of 5.70 ($sd = 2.23$) telephone counseling sessions with each session averaging 17.35 ($sd = 5.50$) minutes during the three month intervention (see Tables 4 and 3). There were no differences between groups on average number or length for the telephone sessions.

3.3. Primary dependent variable: beck depression inventory (BDI)

Participants significantly decreased their depressive symptoms from baseline to post-intervention $f(1,33) = 34.83$, $p < 0.001$, and from baseline to one-month follow-up, $f(1,33) = 37.01$, $p < 0.001$. Participants in the mindfulness-based yoga group exhibited significantly decreased depressive scores from baseline to post-intervention, $f(1,17) = 18.51$, $p < 0.001$ and from baseline to one-month follow-up, $f(1,17) = 21.27$, $p < 0.001$. Participants in the walking control similarly reported significant decreases of depressive symptoms from baseline to post-intervention, $f(1,15) = 16.5$, $p < 0.01$ and from baseline to one-month follow-up, $f(1,15) = 15.5$, $p < 0.01$. Means and standard deviations are presented in Table 4.

Between groups analysis of covariance (ANCOVA) was used to analyze BDI mean scores between the mindfulness-based yoga and walking control conditions at post-intervention and one-month follow-up. The baseline BDI score was entered as a covariate in order to control for baseline variability. There were no significant between group differences on depression scores at post-intervention, $f(1,31) = 0.61$, $p = 0.44$; $d = 0.25$, and one-month follow-up $f(1,31) = 0.80$, $p = 0.78$; $d = 0.08$.

3.4. Secondary dependent variable: the ruminative responses scale

Between groups analysis of covariance (ANCOVA) was used to analyze rumination scores between the mindfulness-based yoga and walking control conditions at post-intervention and one-month follow-up. The RRS score at baseline was entered as a covariate in order to control for baseline variability. The mindfulness-based yoga condition reported significantly lower levels of rumination than the walking control condition, after controlling for baseline levels of rumination, at post-intervention ($f(1,31) = 6.23$, $p < 0.01$; $d = 0.55$. There were no significant between group differences on rumination scores at the one-month follow-up; $d = 0.40$. Means and standard deviations are presented in Table 5.

4. Discussion

Participants in both conditions reported a comparable decrease in depressive scores over three months in that the mean depression scores decreased from "moderate depression" to "mild depression" for both groups at 12 weeks. The decreased depression scores were maintained at the one-month follow-up. Consistent with previous studies, in the current study there were no between group differences on depression scores at post-intervention or one-month follow-up.[13,17,19,40] In contrast, a study by Streeter and colleagues reported lower mood and anxiety scores among participants in a three month yoga intervention relative to a walking intervention.[41] One limitation of the Streeter study was that the participants were not depressed at baseline. Additionally, mood was measured using the Exercise-Induced Feeling Inventory (EFI) in the Streeter study, which is a general assessment of feeling states (e.g., refreshed, peaceful, fatigued), but not depressive symptoms.

Rumination significantly decreased in the yoga group at post-intervention relative to the walking group (effect size calculations indicated a moderate effect). This is consistent with Kinser and colleagues[19] who found that depressed women did not report a decrease on depressive symptoms relative to an active control group; however, did report a greater reduction in rumination scores. Treynor, Gonzalez, and Nolen-Hoeksema[27] suggested that a low sense of mastery contributes to brooding, a factor of rumination, which involves repeatedly contemplating what is wrong in one's life. During yoga, mindfulness may decrease rumination by giving participants an opportunity to focus on alternative thoughts or sensations (i.e., breathing, physical activity of the poses). Additionally, the mindfulness-based education sessions in the yoga group were adapted from the Mindfulness-Based Stress Reduction Program,[32] which has been shown to decrease rumination in both clinical and non-clinical populations.[42,43] Uebelacker et al.[44] suggests that the mindfulness component of yoga (and MBSR) may change the content of thoughts or cognitions. The teachings of self-acceptance, contentment, and non-judgment may lead to increased self-mastery and self-efficacy, which then lead to more positive, affirming thoughts. These cognitions may assist in the management and/or prevention of depressive symptoms. There were no between group differences for rumination at the one-month follow-up, however.

The increase in both yoga and walking is promising given physical activity has the dual benefit of improving overall health and alleviating depression and anxiety.[45] Similar to aerobic exercise, hatha yoga is associated with improved cardiopulmonary fitness,

Table 4
Means and standard deviations for BDI by study arm.

Study Arm	BDI Baseline	Post	Follow-up
Yoga (n = 18)	26.20(6.60)	18.06 (10.86)	17.28 (11.23)
Walking (n = 16)	26.50(5.50)	15.69 (8.20)	16.50 (8.03)

Note. BDI = Beck Depression Inventory. Standard deviations are in parentheses.

Table 5
Means and standard deviations for RRS by study arm.

Study Arm	RRS Baseline	Post	Follow-up
Yoga (n = 18)	60.20 (10.38)	41.94 (8.04)	40.44 (7.76)
Walking (n = 16)	58.45 (9.61)	47.56 (11.93)	44.50 (12.19)

Note. RRS = Ruminative Responses Scale. Standard deviations in parentheses.

muscular strength, and endurance.[46–48] Perhaps the improvement in depression scores was a result of overall increased moderate intensity physical activity rather than yoga or walking in particular. Regular contact with the telephone counselor and completion of exercise logs may also have contributed to the increase of physical activity and decrease in depressive symptoms in both study conditions.

The present study design has several strengths and addressed some of the most significant methodological issues in previous studies. Well validated, reliable measurement tools were used to assess both the presence and severity of depressive symptoms. Additionally, the current study utilized a 12-week intervention, which is longer than most studies in the yoga and depression literature. The inclusion of an active comparison condition, which included both MET-matched walking and time-matched wellness telephone sessions controlled for the possible effect of exercise and non-specific therapeutic effect of contact with a counselor on depression symptoms. The only differential treatment factor between the two conditions was the focus on mindfulness training for the intervention (yoga) condition. Additionally, the individual conducting the assessments at post-intervention and the one-month follow-up was blind to the condition assignment. The random allocation procedure used in the current study allowed for a more robust examination of the cause-effect relationship between mindfulness-based hatha yoga and depression. Finally, this study had high adherence to the study protocol (i.e., yoga or walking) and low attrition numbers, which has been shown to be problematic in previous depression and yoga intervention studies.[45,49]

Despite the strengths discussed above, there were some limitations. First, the majority of participants were Caucasian, educated, employed, and were of mid-high socioeconomic status. The current study included a small sample size, which may have lowered the statistical power to find significance differences between groups. Additionally, the measures were self-report, which is a limitation, particularly when evaluating adherence to the protocols and physical activity behavior. The participants were aware of the purpose of the study and therefore, social desirability bias may have also influenced the results of the study.

Future research in yoga and depression should include diverse and larger sample sizes, longer interventions, and long-term follow-ups. The dose response of yoga with varying frequency, duration, intensity, and type should also be explored. Yoga relative to traditional treatments should also be examined. Researchers should examine if severity of depressive symptoms plays a role in the efficacy of yoga interventions. This study incorporated a gentler form of hatha yoga. Perhaps vigorous styles of yoga would be more efficacious for mild to moderate levels of depression than gentler forms of yoga. Finally, future research should include biomarker measures (e.g., cortisol) of depression and anxiety symptoms and objective measurements of physical activity. Although the results of the current study are compelling and there is an increased interest in alternative methods for treating depression, it remains unclear the effect yoga has on depressive symptoms. This study supports the need for continued large-scale research studies to evaluate the effect of yoga on depressive symptoms relative to an active comparison condition.

Conflict of interest

None declared.

Acknowledgements

This study was submitted in partial fulfillment of the requirements for the Degree of Doctor of Philosophy at the University of Minnesota. We would like to thank Dr. Miriam Cameron, Dr. Maureen Weiss, Dr. John Romano, Dr. Leslie Scibora, Dr. Amanda Bonikowske, and Laura Polikowsky for their significant contributions to the conduct of this study. We would also like to thank Amy Weintraub for contributing to the study protocol and providing the DVD's at a discounted rate. This study would not have been possible without our study participants and we are very grateful for their participation.

References

1. Kessler RC, Berglund P, Demler O, et al. The epidemiology of major depressive disorder: results from the National Comorbidity Survey Replication (NCS-R). *JAMA*. 2003;289(23):3095–3105.
2. Moussavi S, Chatterji S, Verdes E, Tandon A, Patel V, Ustun B. Depression, chronic diseases, and decrements in health: results from the World Health Surveys. *Lancet*. 2007;370(9590):851–858.
3. Rubio JM, Markowitz JC, Alegría A, et al. Epidemiology of chronic and nonchronic major depressive disorder: results from the national epidemiologic survey on alcohol and related conditions. *Depress Anxiety*. 2011;28(8):622–631.
4. Mathers CD, Loncar D. Projections of global mortality and burden of disease from 2002 to 2030. *PLoS Med*. 2006;3(11):e442.
5. Kupfer DJ. Long-term treatment of depression. *J. Clin. Psychiatry*. 1991;5:28–34.
6. Mathew S, Charney D. Publication bias and the efficacy of antidepressants. *Am. J. Psychiatry*. 2009;166(2):140–145.
7. Pigott HE, Leventhal AM, Alter G, Boren J. Efficacy and effectiveness of antidepressants: current status of research. *Psychother. Psychosom*. 2010;79(5):267–279.
8. Rief W, Nestoriuc Y, Weiss S, Welzel E, Barsky AJ, Hofmann SG. Meta-analysis of the placebo response in antidepressant trials. *J. Affect. Disord*. 2009;118(1):1–8.
9. Turner EH, Matthews AM, Linardatos E, Tell RA, Rosenthal R. Selective publication of antidepressant trials and its influence on apparent efficacy. *N. Engl. J. Med*. 2008;358(3):252–260.
10. Cramer H, Lauche R, Langhorst J, Dobos G. Yoga for depression: a systematic review and meta-analysis. *Depress Anxiety*. 2013;30(11):1068–1083.

11. Pilkington K, Kirkwood G, Rampes H, Richardson J. Yoga for depression: the research evidence. *J. Affect. Disord.* 2005;89(1):13–24.
12. Shapiro D, Cook IA, Davydov DM, Ottaviani C, Leuchter AF, Abrams M. Yoga as a complementary treatment of depression: effects of traits and moods on treatment outcome. *Evid. Based Complement. Alternat. Med.* 2007;4(4):493–502.
13. Woolery A, Myers H, Sternlieb B, Zeltzer L. A yoga intervention for young adults with elevated symptoms of depression. *Altern. Ther.* 2004;10(2):60–63.
14. Mathew KL, Whitford HS, Kenny MA, Denson LA. The long-term effects of mindfulness-based cognitive therapy as a relapse prevention treatment for major depressive disorder. *Behav. Cognit. Psychother.* 2010;38(05):561–576.
15. Teasdale JD, Segal ZV, Williams JMG, Ridgeway VA, Soulsby JM, Lau MA. Prevention of relapse/recurrence in major depression by mindfulness-based cognitive therapy. *J. Consult. Clin. Psychol.* 2000;68(4):615.
16. Phillips WT, Kiernan M, King AC. Physical activity as a nonpharmacological treatment for depression: a review. *Comp. Health Prac. Rev.* 2003;8(2):139–152.
17. Dhananjai S, Sadashiv ST, Dutt K, Kumar R. Reducing psychological distress and obesity through Yoga practice. *Int. J. Yoga.* 2013;6(1):66.
18. Hofmann SG, Sawyer AT, Witt AA, Oh D. The effect of mindfulness-based therapy on anxiety and depression: a meta-analytic review. *J. Consult. Clin. Psychol.* 2010;78(2):169–183.
19. Kinser PA, Bourguignon C, Whaley D, Hauenstein E, Taylor AG. Feasibility, acceptability, and effects of gentle hatha yoga for women with major depression: findings from a randomized controlled mixed-methods study. *Arch. Psychiatr. Nurs.* 2013;27(3):137–147.
20. Uebelacker LA, Tremont G, Epstein-Lubow G, et al. Open trial of Vinyasa yoga for persistently depressed individuals: evidence of feasibility and acceptability. *Behav. Modif.* 2010;34(3):247–264.
21. Quilty MT, Saper RB, Goldstein R, Khalsa SBS. Yoga in the real world: perceptions, motivators, barriers, and patterns of use. *Glob. Adv. Health Med.* 2013;2(1):44–49.
22. Ross A, Friedmann E, Bevans M, Thomas S. National survey of yoga practitioners: mental and physical health benefits. *Complement. Ther. Med.* 2013;21(4):313–323.
23. First MB, Spitzer RL, Gibbons M, Williams JB. *Structured Clinical Interview for DSM-IV—ClinicalVersion (SCID-CV) (User's Guide and Interview).* Washington, D.C: American Psychiatric Press, Inc.; 1997.
24. Beck AT, Ward CH, Mendelson M, Mock J, Erbaugh JK. An inventory for measuring depression. *Arch. Gen. Psychiatry.* 1961;4(6):561–571.
25. American Psychiatric Association. Diagnostic And Statistical Manual Of Mental Disorders DSM-IV-TR Fourth Edition (Text Revision) Author: American Psychiatric Association (2000).
26. Beck AT, Steer RA, Carbin MG. Psychometric properties of the Beck Depression Inventory: twenty-five years of evaluation. *Clin. Psychol. Rev.* 1988;8(1):77–100.
27. Treynor W, Gonzalez R, Nolen-Hoeksema S. Rumination reconsidered: a psychometric analysis. *Cognit. Ther. Res.* 2003;27(3):247–259.
28. Bagby RM, Rector NA, Segal ZV, et al. Rumination and distraction in major depression: assessing response to pharmacological treatment. *J. Affect. Disord.* 1999;55(2):225–229.
29. Nolen-Hoeksema S, Davis CG. Thanks for sharing that: ruminators and their social support networks. *J. Pers. Soc. Psychol.* 1999;77(4):801.
30. Nolen-Hoeksema S, Parker LE, Larson J. Ruminative coping with depressed mood following loss. *J. Pers. Soc. Psychol.* 1994;67(1):92.
31. Weintraub, A., Duncan, D. (Producers). LifeForce yoga to beat the blues: Level 1. [Video/DVD]. Pennsauken, NJ : Disc Makers (2007).
32. Kabat-Zinn J. *Full Catastrophe Living: Using the Wisdom of Your Body and Mind to Face Stress, Pain, and Illness.* New York, NY: Random House, Inc.; 1990.
33. Goldin PR, Gross JJ. Effects of mindfulness-based stress reduction (MBSR) on emotion regulation in social anxiety disorder. *Emotion.* 2010;10(1):83.
34. Grossman P, Niemann L, Schmidt S, Walach H. Mindfulness-based stress reduction and health benefits: a meta-analysis. *J. Psychosom. Res.* 2004;57(1):35–43.
35. Lengacher CA, Johnson-Mallard V, Post-White J, et al. Randomized controlled trial of mindfulness-based stress reduction (MBSR) for survivors of breast cancer. *Psycho-oncology.* 2009;18(12):1261–1272.
36. Bohlmeijer E, Prenger R, Taal E, Cuijpers P. The effects of mindfulness-based stress reduction therapy on mental health of adults with a chronic medical disease: a meta-analysis. *J. Psychomsom. Res.* 2010;68(6):539–544.
37. Vøllestad J, Sivertsen B, Nielsen GH. Mindfulness-based stress reduction for patients with anxiety disorders: evaluation in a randomized controlled trial. *Behav. Res. Ther.* 2011;49(4):281–288.
38. Fjorback LO, Arendt M, Ørnbøl E, Fink P, Walach H. Mindfulness-based stress reduction and mindfulness-based cognitive therapy—a systematic review of randomized controlled trials. *Acta Psychiatr. Scand.* 2011;124(2):102–119.
39. Sansone, L. (2008). Leslie Sansone: Walk at home-5 mile fat burning walk. [Video/DVD].
40. Javnbakht M, Hejazi Kenari R, Ghasemi M. Effects of yoga on depression and anxiety of women. *Complement. Ther. Clin. Pract.* 2009;15(2):102–104.
41. Streeter CC, Whitfield TH, Owen L, et al. Effects of yoga versus walking on mood, anxiety, and brain GABA levels: a randomized controlled MRS study. *J. Altern. Complement. Med.* 2010;16(11):1145–1152.
42. Campbell TS, Labelle LE, Bacon SL, Faris P, Carlson LE. Impact of mindfulness-based stress reduction (MBSR) on attention, rumination and resting blood pressure in women with cancer: a waitlist-controlled study. *J. Behav. Med.* 2012;35(3):262–271.
43. Jain S, Shapiro SL, Swanick S, et al. A randomized controlled trial of mindfulness meditation versus relaxation training: effects on distress, positive states of mind, rumination, and distraction. *Ann. Behav. Med.* 2007;33(1):11–21.
44. Uebelacker LA, Epstein-Lubow G, Gaudiano BA, Tremont G, Battle CL, Miller IW. Hatha yoga for depression: critical review of the evidence for efficacy, plausible mechanisms of action, and directions for future research. *J. Psychiatr. Pract.* 2010;16(1):22–33.
45. Dunn AL, Trivedi MH, Kampert JB, Clark CG, Chambliss HO. Exercise treatment for depression: efficacy and dose response. *Am. J. Prev. Med.* 2005;28(1):1–8.
46. Raub JA. Psychophysiologic effects of Hatha Yoga on musculoskeletal and cardiopulmonary function: a literature review. *J. Altern. Complement. Med.* 2002;8(6):797–812.
47. Ross A, Thomas S. The health benefits of yoga and exercise: a review of comparison studies. *J. Altern. Complement. Med.* 2010;16(1):3–12.
48. Tran MD, Holly RG, Lashbrook J, Amsterdam EA. Effects of Hatha Yoga practice on the health-related aspects of physical fitness. *Prev. Cardiol.* 2001;4(4):165–170.
49. Alexander G, Innes KE, Bourguignon C, Bovbjerg VE, Kulbok P, Taylor AG. Patterns of yoga practice and physical activity following a yoga intervention for adults with or at risk for type 2 diabetes. *J. Phys. Activity Health.* 2012;9(1):53.
