# Supplementary material for: Does physical activity-based intervention decrease repetitive negative thinking? A systematic review
Source: PLoS One. 2025 Apr 1;20(4):e0319806. doi: 10.1371/journal.pone.0319806 (PMC11960971; doi:10.1371/journal.pone.0319806)
Supplement: S1 File — https://doi.org/10.6084/m9.figshare.25711734. (ZIP) [file pone.0319806.s001.zip › supporting information/paper file/La Rocque 2021.pdf]

Journal Pre-proof

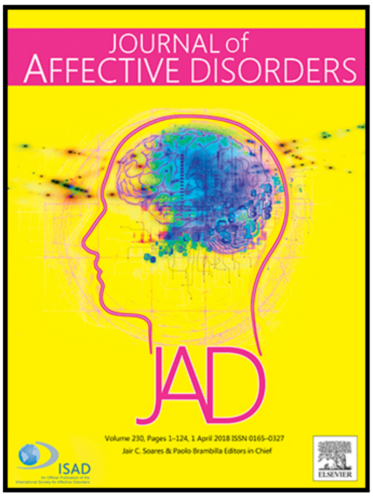

Randomized Controlled Trial of Bikram Yoga and Aerobic Exercise for Depression in Women: Efficacy and Stress-Based Mechanisms

Cherie L. La Rocque , Raegan Mazurka , Troy J.R. Stuckless , Kyra Pyke , Kate L. Harkness

PII: S0165-0327(20)32912-8
DOI: https://doi.org/10.1016/j.jad.2020.10.067
Reference: JAD 12609

To appear in: *Journal of Affective Disorders*

Received date: 28 April 2020
Revised date: 13 September 2020
Accepted date: 31 October 2020

Please cite this article as: Cherie L. La Rocque , Raegan Mazurka , Troy J.R. Stuckless , Kyra Pyke , Kate L. Harkness , Randomized Controlled Trial of Bikram Yoga and Aerobic Exercise for Depression in Women: Efficacy and Stress-Based Mechanisms, *Journal of Affective Disorders* (2020), doi: https://doi.org/10.1016/j.jad.2020.10.067

This is a PDF file of an article that has undergone enhancements after acceptance, such as the addition of a cover page and metadata, and formatting for readability, but it is not yet the definitive version of record. This version will undergo additional copyediting, typesetting and review before it is published in its final form, but we are providing this version to give early visibility of the article. Please note that, during the production process, errors may be discovered which could affect the content, and all legal disclaimers that apply to the journal pertain.

© 2020 Published by Elsevier B.V.

Journal Pre-proof

Randomized Controlled Trial of Bikram Yoga and Aerobic Exercise for Depression in Women: Efficacy and Stress-Based Mechanisms

Cherie L. La Rocque[a], Raegan Mazurka[a], Troy J. R. Stuckless[b], Kyra Pyke[c], Kate L. Harkness[c,*1]

[a]Queen's University

[b]University of Toronto

[c]Queen's University

Highlights

- Bikram yoga and aerobic exercise led to greater depression response than a wait-list condition
- Bikram yoga and aerobic exercise led to decreased levels of rumination
- Decreases in rumination drove the symptom change seen in Bikram yoga and exercise
- Increases in mindful acceptance drove the symptom change seen in aerobic exercise

Journal Pre-proof

Abstract

Background: The current study presents a randomized controlled 8-week trial of Bikram yoga, aerobic exercise, and waitlist for depression. Bikram yoga was chosen specifically for its standardized nature. Further, we examined changes in three stress-related constructs—perceived stress, rumination, and mindfulness—as mediators of antidepressant effects.

Method: Fifty-three women (age 18-65; 74% White) with a unipolar depressive disorder were randomly assigned to one of the three conditions. Response was defined as >50% reduction on the Hamilton Rating Scale for Depression (HAM-D). Remission was defined as no longer meeting criteria for

[1] Corresponding Author Address: Department of Psychology, 62 Arch St, Queen's University, Kingston, ON, K7L 3N6; Phone: 613-533-2886; email: harkness@queensu.ca

Journal Pre-proof

depression and a HAM-D ≤ 7. Self-reported perceived stress, rumination, and mindfulness were assessed weekly.

Results: In the intention-to-treat sample (n = 53), response rates were significantly higher in the Bikram yoga (61.1%; χ^2=10.48, p = .001) and aerobic exercise (60.0%; χ^2=10.44, p = .001) conditions relative to waitlist (6.7%). In the completer sample (n = 42), 73.3% (χ^2=11.41, p = .001) of women in yoga and 80.0% (χ^2=13.72, p < .001) in exercise achieved response compared to 8.3% in waitlist. Reductions in rumination significantly mediated HAM-D change for both active treatments, and mindful acceptance was a partial mediator in the exercise condition.

Limitations: The sample was small in size, consisted of women only, and was ethnically homogenous. Inter-rater reliability was not assessed, aerobic exercise was not standardized, and mediators were assessed by self-report.

Conclusions: Bikram yoga showed descriptively similar efficacy to aerobic exercise and both may work, in part, by helping individuals interrupt negative thinking.

Keywords

Depression; Bikram yoga; aerobic exercise; rumination; mindfulness.

Journal Pre-proof

Introduction

Major depressive disorder affects 300 million people globally and is a leading cause of disability (World Health Organization, 2017). Only about half of those suffering from depression seek conventional treatment (Patten et al., 2015), and among those who do, adherence rates are low, often due to lack of response and/or unpleasant side effects (Rush & Thase, 2018). These factors limit the promise of conventional treatments to fully address the burden of depression.

Journal Pre-proof

Up to 20% of individuals with mood disorders have used one or more complementary, integrative treatments in the past 12 months, with the most frequent being physical or mind-body therapies, such as aerobic exercise and yoga (de Jonge et al., 2018). Current treatment guidelines for mild to moderate depression recommend aerobic exercise as a first-line monotherapy, and yoga as a second-line adjunctive therapy (NICE, 2016; Ravindran et al., 2016).

The primary goal of the current study was to examine the efficacy of Bikram yoga and aerobic exercise relative to a waitlist condition in an 8-week randomized controlled trial (RCT) in women with depression. Our second goal was to examine changes in three stress-related constructs—perceived stress, rumination, and mindfulness—as mediators of the effects of Bikram yoga and aerobic exercise on depression symptoms.

Antidepressant Efficacy of Exercise and Yoga

Exercise is defined as structured and repetitive bodily movement done to improve or maintain physical fitness (American College of Sports Medicine, 2013). Most studies in depression employ aerobic exercise (e.g., jogging, cycling, or other cardiovascular training), practiced for 30-60 minutes, 2-3 times per week for at least 8 weeks (Perraton et al., 2010). In a meta-analysis of 23 RCTs, Kvam et al. (2016) found a large effect of exercise on depression compared to wait-list or no treatment (g = -1.24), moderate effects relative to treatment-as-usual (g = -0.48), and comparable effects to psychotherapy (g = -0.22) and antidepressant medication (g = -0.08; see also Schuch et al., 2016).

Yoga is an ancient Indian system of philosophy and practice that promotes the interconnection of mind, body, and spirit (Salmon et al., 2009). A meta-analysis of 12 RCTs of yoga for depression symptoms revealed a moderate effect compared to treatment-as-usual, and comparable efficacy to relaxation and aerobic exercise (Cramer et al., 2013). In a subsequent systematic review of seven RCTs in depressive disorder, similar response rates were seen between yoga and both aerobic exercise and

Journal Pre-proof

antidepressant medication (Cramer et al., 2017). However, the authors identified several limitations with the existing evidence base for yoga, including a lack of standardized instruments to diagnose depression, insufficient randomization and blinding procedures, and failure to report attrition rates. Studies also have varied widely in the type, length, and frequency of yoga. As such, researchers have highlighted the importance of using standardized styles of yoga, which would mirror manualized psychotherapy protocols (Uebelacker et al., 2010, 2017). A small number of methodologically rigorous RCTs of yoga have since been conducted that include structured interviews, such as the Structured Clinical Interview for DSM-IV Axis I Disorders (SCID; First et al., 1997) to diagnose depression and standardized instruments, such as the Hamilton Rating Scale for Depression (HAM-D; Hamilton, 1960), to assess symptom change (see Hewett et al., 2018; Kwok et al., 2019; Streeter et al., 2017; Uebelacker et al., 2017). In particular, Streeter et al. (2017) and Uebelacker et al. (2017) have utilized manualized yoga protocols for depression.

Bikram yoga is the most standardized form of yoga. It consists of an unchanging 90-minute sequence of 26 physical postures and two breathing exercises (Choudhury, 2007), and is practiced in a heated environment (~40°C, 40% humidity) to increase active and passive range of motion (Bleakley & Costello, 2013). All Bikram yoga instructors undergo an intense certification course and must instruct the class with a scripted dialogue. As such, Bikram yoga meets the call for examining standardized forms of yoga in research. Given proper medical screening, Bikram yoga is well-tolerated, even in samples of obese older adults (see Hewett et al., 2015). A comparison among 15 styles of yoga revealed that Bikram yoga scored highest on ‘physicality’(Park et al., 2018). Therefore, this form of yoga also provides a good comparison to aerobic exercise. A recent uncontrolled 8-week pilot trial of Bikram yoga for mild depression reported encouraging results, with 56.5% of patients meeting criteria for remission based on standardized depression instruments (Nyer et al., 2019).

Mechanisms Underlying the Effects of Exercise and Yoga

Journal Pre-proof

Journal Pre-proof

Exercise and yoga are theorized to convey mental and physical health benefits, in part, via changes in the stress response (e.g., Riley & Park, 2015), and changes in key psychological constructs associated with the stress response, specifically perceived stress, rumination, and mindfulness, are seen following these interventions (reviewed below). To our knowledge, the current study is the first to empirically examine whether the effects of Bikram yoga or aerobic exercise on depression symptoms are *mediated* by these changes.

In previous trials for depression, both aerobic exercise (Askari et al., 2017) and a variety of styles of yoga (Chu et al., 2017; Hewett et al., 2018; Michalsen et al., 2005) were associated with significantly larger reductions in perceived stress than control conditions, and comparable reductions in perceived stress compared to medication-only. Rumination is one of the most robust prospective predictors of depression and involves repetitive and passive past-oriented thoughts about stress without engagement in active coping (Aldao et al., 2010). In trials for depression, both aerobic exercise (Craft, 2005) and Hatha yoga (Kinser et al., 2013, 2014) are associated with significantly greater decreases in rumination relative to control conditions.

Finally, mindfulness is defined as "the awareness that emerges through paying attention on purpose, in the present moment, and nonjudgmentally to the unfolding of experience moment by moment" (Kabat-Zinn, 2003, p. 145). A nonjudgmental present-moment focus is believed to help free the mind from excessive orientation toward the past or future when dealing with stressors. Several investigators have theorized that aerobic exercise fosters mindfulness through promotion of a mind-body connection and increased capacity to self-regulate attention (see Salmon et al., 2010). Indeed, cross-sectional studies have reported a significant positive relation between engaging in physical activity and levels of mindfulness (e.g., Kangasniemi et al., 2014; Ulmer et al., 2010), and RCTs have shown that aerobic exercise results in significant improvements in mindfulness (de Bruin et al., 2016; Mothes et al.,

Journal Pre-proof

Journal Pre-proof

2014). Several trials have also reported significant increases in mindfulness following yoga practice (e.g., Hewett et al., 2011), including in individuals with elevated depression symptoms (e.g., Falsafi, 2016; Uebelacker et al., 2010).

Objectives and Hypotheses

The current study improved upon many previous investigations of yoga by using randomization procedures, standardized instruments to diagnose depression and assess symptom change, outcome assessors blind to treatment condition, and an intention-to-treat (ITT) analysis. Our first goal was to examine the efficacy of Bikram yoga and aerobic exercise relative to a waitlist condition in an 8-week RCT in women meeting diagnostic criteria for unipolar depression. Depression is at least twice as common in women than in men (Salk et al., 2017). Further, national surveys suggest that over three-quarters of yoga practitioners across yoga types are female (Riley & Park, 2015). Therefore, given that this is a preliminary trial that is not sufficiently powered to detect potential moderation by sex or gender, we recruited women only.

We hypothesized that women in the Bikram yoga and exercise conditions would experience significantly greater improvements in depression symptoms from pre- to post-treatment, and a higher likelihood of response and remission, than those in waitlist. Our second goal was to test the hypothesis that both active treatments would show larger reductions in perceived stress and rumination, and greater increases in mindful awareness and acceptance than the waitlist group, and these changes would mediate improvements in depression symptoms in the active treatment groups, but not the waitlist group. This trial did not have the statistical power to provide a test of equivalency (Läkens et al., 2018). Therefore, the comparison between the yoga and exercise conditions is descriptive in order to support future large-scale tests of equivalency.

Method

Journal Pre-proof

Journal Pre-proof

Participants

Women were recruited from a small city in Southeastern Ontario via advertisements. Ethical approval was obtained from the Queen's University Health Sciences Research Ethics Board and the trial was registered with Clinicaltrials.gov [NCT04058080]. All participants met current DSM-5 (APA, 2013) criteria for a non-chronic, unipolar depressive disorder. Exclusion criteria were lifetime psychotic, bipolar, or substance disorder; suicidal intent; and a medical condition interfering with physical activity. Pregnant women, and those participating bi-weekly or more in yoga or group exercise, were excluded. If women were engaged in traditional treatment, they were excluded if they had a change in the type or dose of medication or frequency of psychotherapy in the previous 3 months.[2]

Of the 217 women who expressed interest, 98 completed a telephone screening, and 56 were invited to the pre-treatment assessment (see CONSORT diagram, Figure 1). Three women declined, leaving a sample of 53 (see Table 1). GPower (Erdfelder et al., 1996) was used to estimate the sample size required to detect an omnibus 3(group) by 2(time) interaction. Given the meta-analytic findings in the literature comparing *either* yoga *or* exercise to wait-list, we estimated that the effect in our data would be large, although not as large as in the previous literature given our inclusion of 3 treatment groups. Therefore, we set eta-squared at 0.10, alpha at .05, power at .95, and the correlation between repeated measures at 0.35. This yielded a sample size of 51.

Measures

Physical health screening

---

[2] Anti-depressant medications taken by women included bupropion, duloxetine, escitalopram, nortriptyline, sertraline, trazodone, and venlafaxine. Medication status was not significantly associated with post-treatment HAM-D scores (p = .340) or response status (p = .276). Its inclusion as a covariate did not change the pattern of results. Therefore, to facilitate interpretation, the uncontrolled models are presented below.

Journal Pre-proof

Journal Pre-proof

Screening recommendations from the Canadian Society for Exercise Physiology and the American College of Sports Medicine included the 7-item Physical Activity Readiness Questionnaire (PAR-Q; Thomas et al., 1992) and questions assessing cardiovascular disease risk. All questions were answered in a yes/no format. Individuals at "moderate" (≥2 'yes' answers on the PAR-Q) or "high" (diagnosis of a cardiovascular, metabolic, or pulmonary disease) risk required medical clearance from their family physician and approval from the 4th author (a PhD kinesiologist with expertise in cardiovascular function during exercise). Two women were deemed at risk but withdrew before randomization.

Depression

The SCID-I/P (First et al., 2002) and the 17-item HAM-D (Hamilton, 1960) were administered by the first or second authors who were blind to intervention assignment throughout the study. Interviewers were senior doctoral students in clinical psychology who were trained and supervised by the last author, a registered clinical psychologist with over 25 years' experience conducting research in depression treatment trials and supervising psychodiagnostic assessment. Training involved first observing interviews and matching the diagnosis, or symptom ratings, of the supervisor on at least three consecutive interviews. They then conducted interviews with the supervisor sitting in and had to match ratings on an additional three consecutive interviews (see Grove et al., 1981). The training also included regular case conference.

All women met criteria for a unipolar depressive disorder (46 MDD, 4 depressive disorder not otherwise specified, and 3 adjustment disorder with depressed mood).[3] Participants were not excluded based on their HAM-D scores, although as noted in Table 1, the average HAM-D score in the current

[3] The pattern of results below was identical when limiting the sample to women with major depressive disorder.

Journal Pre-proof

Journal Pre-proof

study was in the mild-moderate range of severity. Response to treatment was defined as a 50% reduction from baseline HAM-D scores, and remission was defined as no longer meeting diagnostic criteria for depression at the post-treatment assessment based on the SCID-I/P *and* scoring 7 or lower on the HAM-D (Rush et al., 2006). In the sample of participants who completed the full 8 weeks of treatment and the post-treatment assessment ('completer' sample), all participants who met criteria for response also met criteria for remission. Therefore, the term 'response' will be used throughout the results.

Perceived stress

The 53-item Hassles and Uplifts Scale (HUS; DeLongis et al., 1988) asks participants to rate 53 items (e.g., "health or well-being of a family member") on a 3-point scale based on how much each was a hassle and an uplift in the previous week. Only items on the hassles scale were summed (α = 0.94). A mean score of 28.28 (*SD* = 15.79) on the HUS was reported in a previous healthy normative sample (Uhrlass & Gibb, 2007).

Rumination

The 22-item Ruminative Responses Scale (RRS; Nolen-Hoeksema & Morrow, 1991) assesses the tendency to ruminate in response to depression symptoms. Items are rated from 1(*almost never)* to 4 (*almost always)* and were summed (α = 0.89). The initial validation sample for the RRS (Nolen-Hoeksema et al., 1999) provided a mean score of 42.01 (*SD* = 10.64) for healthy women.

Acceptance and Awareness

The 20-item Philadelphia Mindfulness Scale (PHLMS; Cardaciotto et al., 2008) assesses awareness (i.e., the ongoing monitoring of current internal and external experiences) and acceptance (i.e., an attitude of openness and compassion about one's experience). Items are rated from 1 (*Never*) to

Journal Pre-proof

5 *(Very Often*) and were summed separately for awareness (α = 0.77) and acceptance (α = 0.76). The initial validation sample for the PHLMS (Cardaciotto et al., 2008) provided mean scores for Awareness and Acceptance of 36.65 (*SD* = 4.93) and 30.19 (*SD* = 5.84), respectively, for the normative sample, and 35.11 (*SD* = 5.39) and 24.62 (*SD* = 5.48), respectively, for the general psychiatric sample.

Treatment Participation

Attendance was tracked objectively. At the yoga studio, participants wrote down their name on a class list and were then signed in by studio personnel through an online system. At the YMCA, participants scanned their YMCA membership card.

Procedure

At the baseline in-person assessment, participants provided written informed consent and completed the above measures. Participants were then assigned via a computer-generated random sequence to their treatment condition, which was revealed via previously prepared sealed envelopes. No participants refused their assignment. Throughout the intervention period, participants received a weekly email with a link to complete, in random order, the HUS, RRS, and PHLMS online.

Intervention

Classes were free of charge to participants, and participants were instructed to start within a week of randomization. They were required to sign a waiver that absolved the establishment of liability in the case of injury. Participants were given an information sheet by study personnel prior to participation that was also verbally discussed with them. Possible physical difficulties with Bikram yoga and exercise were highlighted, and it was emphasized that participation was completely voluntary and under the participant’s control. Taking breaks during class was normalized.

Journal Pre-proof

Journal Pre-proof

Women in the Bikram yoga group were asked to attend two 90-minute classes per week for 8 weeks at a specific Bikram yoga studio with certified Bikram teachers. The standard 90-minute Bikram class was the only yoga class available at this studio. Classes were held in a temperature-controlled room (~40°C, 40% humidity). Classes opened with a deep breathing exercise and continued with 50 min of standing *asanas* (i.e., body poses or positions) and 40 min of floor-based *asanas*, including a forceful breathing exercise to finish. All but the last *asana* were performed twice. *Savasana* – a posture of gradually relaxing the body parts – was performed between *asanas* throughout the floor series and at the end of class (Choudhury, 2007). The yoga studio offered 22 class times per week, all of which were available.

Participants in the aerobic exercise group were asked to attend two 50-60-min group classes per week for 8 weeks at the local YMCA. Classes involving the following components were available: choreography-based cardio, aerobics, light muscular conditioning, and stretching; cardio, plyometric, and strength training; high intensity aerobic exercise with intermittent rest periods; circuit-based cardio and strength training; stepper-based exercises; and Latin-inspired dance/fitness. Participants could choose any of the 18-22 classes offered each week.

Women in the waitlist condition were not able to access yoga or exercise classes throughout the intervention period but participated in the rest of the study protocol. Following the post-treatment assessment, they received their choice of 8 weeks of either yoga or aerobic exercise classes at the above-noted studios for free.

Post-Treatment Assessment

The post-treatment session included administration of the Mood Module of the SCID-I/P and the HAM-D. Participants were compensated $50 at the end of this appointment for completing the study

Journal Pre-proof

Journal Pre-proof

assessments and were referred back to their treatment provider or were provided with a list of clinical resources.

Data Analysis

Analyses were conducted using the Statistical Program for the Social Sciences 24 (SPSS) and HLM 7 (Raudenbush et al., 2017). To address Goal 1, we conducted analyses separately with the completer sample (n = 42) and with the full randomized sample (*n* = 53). Completers included participants who completed 8 weeks of treatment (or wait-list) and attended the final in-person assessment. The last observation carried forward (LOCF) method was used to address missing post-treatment data in the ITT sample (see Blumenthal et al., 2007; de Manincor et al., 2016). The LOCF method assumes no change from pre-treatment HAM-D scores for noncompleters; thus, this method was chosen over multiple imputation as it provides the most conservative test of treatment efficacy.

In both samples, 2(time: pre-, post-intervention) x 3(group: exercise, yoga, waitlist) mixed-model ANOVAs were performed with HAM-D scores as the dependent variable. Significant interactions were followed up with pairwise comparisons between means using a Bonferroni correction. Clinically significant change was assessed using the Reliable Change Index (RCI) and associated effect sizes calculated using the ACORN tool (ACORN Organization Inc.). The meta-analytic reliability of the HAM-D was used (α = 0.789; Trajković et al., 2011). Chi-square tests were used to examine the relation of treatment group to response. Follow-up pairwise comparisons, using a Bonferroni correction, evaluated differences among proportions.

To address Goal 2, first, multilevel modeling (MLM) assessed group differences in changes in perceived hassles, rumination, awareness, and acceptance over the 8 weeks. Random intercepts and slopes were specified to allow for individual growth trajectories. Parameters were estimated by restricted maximum likelihood. Repeated measurements of each psychological variable (Level 1) were

Journal Pre-proof

nested within participants (Level 2). Pairwise comparisons between groups were dummy coded, and two iterations of each model were specified to enable all comparisons (i.e., yoga vs. waitlist, exercise vs. waitlist, yoga vs. exercise). Time was entered as a fixed effect at Level 1 and coded such that the intercept represented values of the psychological variable at post-treatment. The slopes represented the rate of change in the psychological variable from pre- to post-treatment. Between subject predictors were included at Level 2.

Second, mediation models were tested using PROCESS (Hayes, 2012). Post-treatment values of each psychological variable and HAM-D were entered as the mediator and dependent variable, respectively, and pre-treatment values were entered as covariates (Hayes & Rockwood, 2017). Mediation was assessed by the indirect effect of treatment group on changes in HAM-D scores, while accounting for the direct effects of treatment group on changes in the psychological variable and HAM-D scores.

Journal Pre-proof

Results

Goal 1: Treatment Outcome

The three groups did not differ significantly on any baseline descriptive characteristic (Table 1). No serious adverse events were reported, and no participants withdrew due to adverse events. Minor adverse events included transient muscle soreness or stiffness. Lower pre-treatment HAM-D scores were significantly associated with lower post-treatment HAM-D scores, $r = .51$, $p < .001$. Similarly, responders had significantly lower pre-treatment HAM-D scores than non-responders (*M*s = 11.92, 13.93; *SD*s = 3.30, 3.57; $t[51] = 2.12$, $p = .039$).

There were no significant differences across groups in attrition ($\chi^2[2] = 0.816$, $p = .66$). Non-completers were more likely than completers to have a comorbid diagnosis (72.7% vs. 35.7%, χ^2 [1, N = 53] = 4.86, $p = .027$), but they did not differ on any other descriptive characteristic (all *p*s > .178).

Journal Pre-proof

Adherence rates for the yoga and exercise groups, respectively, were 68.8% and 66.9% in the ITT sample, and 80% and 85.8% in the completer sample. Among completers, the number of classes attended did not differ significantly between groups (*M*s = 12.80, 13.73; *SD*s = 2.27, 2.31; $t[28] = 1.11$, $p = .275$). Further, the correlation between total classes attended and change in HAM-D scores was not significant ($p = .350$), and total classes did not differ significantly between responders and non-responders ($p = .478$).

Primary Analyses. In the ITT sample, there was a significant group by time interaction ($F[2, 50] = 11.41$, $p < .001$, partial $\eta^2 = .31$). HAM-D scores significantly decreased from pre- to post-treatment in the yoga ($t[17] = 3.70$, $p =.001$, $d = .89$, CI_{95} [2.31, 8.13]) and exercise ($t[19] = 4.38$, $p < .001$, $d = .98$, CI_{95} [2.74, 7.76]) groups, but increased in the waitlist group ($t[14] = -2.21$, $p =.044$, $d = .57$, CI_{95} [-4.73, -0.07]) (see Table 1). For the yoga and exercise groups, these were clinically significant changes (*RCIs* = 3.92, 5.25; effect sizes = 2.03, 1.70, respectively). Similarly, response rates differed significantly across groups (χ^2 [2, $N = 53$] = 12.60, $p = .002$, Cramer's $V = .49$). The proportion of responders in the waitlist condition (6.7%) was significantly lower than in the yoga (61.1%, χ^2 [1, $N = 33$] = 10.48, $p = .001$, Cramer's $V = .56$) and exercise (60.0%, χ^2 [1, $N = 35$] = 10.44, $p = .001$, Cramer's $V = .55$) groups.

In the completer sample, the group by time interaction was significant ($F[2, 39] = 12.73$, $p < .001$, partial $\eta^2 = .40$). HAM-D scores significantly decreased from pre- to post-treatment in the yoga ($t[14] = 4.13$, $p =.001$, $d = 1.07$, CI_{95} [3.01, 9.52]) and exercise ($t[14] = 5.33$, $p < .001$, $d = 1.38$, CI_{95} [4.18, 9.82]) groups, but not waitlist ($t[11] = -1.87$, $p =.089$, $d = .63$, CI_{95} [-5.44, -0.10]) (See Table 2). Response rates differed across groups (χ^2 [2, $N = 42$] = 16.48, $p < .001$, Cramer's $V = .63$). The proportion of responders in waitlist (8.3%) was significantly lower than in the yoga (73.3%, χ^2 [1, $N = 27$] = 11.41, $p = .001$, Cramer's $V = .65$) and exercise (80.0%, χ^2 [1, $N = 27$] = 13.72, $p < .001$, Cramer's $V = .71$) groups.

Goal 2: Mechanisms of Action

The treatment groups did not differ significantly on the four mediators pre-treatment (Table 2).

Journal Pre-proof

Journal Pre-proof

Older age was significantly associated with lower pre-treatment rumination (*r* = -.39, *p* = .011), higher pre-treatment acceptance (*r* = .37, *p* = .017), and higher post-treatment hassles in the MLM sample (*n* = 36; *r* = .48, *p* = .007) and the sample used for mediation, which used the LOCF (*n* = 42; *r* = .35, *p* = .025). Including age in the models below did not change the pattern of findings; therefore, for ease of interpretation, we present the uncontrolled analyses.

Multilevel Models of Change. Table 3 presents fixed effect estimates of the pairwise comparisons between groups on post-treatment intercepts and slopes. Estimates from the comparison of yoga versus exercise from the second iteration of the model are in the text.

For perceived hassles, the exercise group had significantly lower post-treatment scores than the waitlist group, and the yoga group differed from waitlist at a trend. The overall rate of linear change in hassles from pre- to post-treatment was significantly different from zero; however, there were no significant pairwise group differences in post-treatment slopes.

For rumination, the yoga and exercise groups had significantly lower post-treatment scores than the waitlist group. Yoga and exercise had rumination slopes that were negative relative to the waitlist group. The yoga and exercise groups did not differ from each other in post-treatment intercept (coefficient = 5.76, *p* = .219) or slope (coefficient = 0.57, *p* = .689; Figure 2a). [4]

For awareness, participants in the yoga group reported significantly higher scores at post-treatment, and had a significantly steeper slope, than those in the waitlist group. There were no significant differences between yoga and exercise in post-treatment intercept or slope (*p*s > .230; Figure 2b).

For acceptance, participants in the exercise group had significantly higher post-treatment acceptance scores than those in the waitlist group. There were no significant pairwise differences in

[4] The estimates presented in this figure were derived from the multilevel model results. Therefore, they do not reflect the actual means from Table 2.

Journal Pre-proof

Journal Pre-proof

slopes between the exercise and waitlist groups. Further, the yoga and exercise groups did not differ significantly in either post-treatment acceptance scores or slopes (*p*s > .250).

Mediation Models

The mediation models for perceived hassles and awareness were not significant and, thus, are not reported further. For rumination, the indirect effects of both active treatment groups versus the waitlist group on post-treatment HAM-D scores were significant (top panel, Table 4): For every 1 unit decrease in post-treatment rumination, post-treatment HAM-D scores decreased by 0.26. For acceptance, the indirect effect of the exercise group versus the waitlist group on post-treatment HAM-D scores was also significant (bottom panel, Table 4): For every 1 unit increase in post-treatment acceptance, post-treatment HAM- D scores decreased by 0.42.

Discussion

Bikram yoga and aerobic exercise resulted in similarly large effects on symptom improvement and rates of response relative to a waitlist condition. RCI analyses indicated that symptom changes within the Bikram yoga and aerobic exercise conditions were clinically significant. Further, perceived stress and rumination decreased significantly to within normative levels in the yoga and exercise groups. Risk of bias was minimized by using proper randomization procedures and blinding of outcome assessors, describing attrition rates, and conducting an ITT analysis. Thus, the current findings provide promising preliminary evidence that Bikram yoga is an efficacious complementary treatment for mild to moderate depression.

Our effect size (mean standard difference) in the current study for Bikram yoga (-1.36 in the ITT sample) is comparable to effect sizes reported in previous trials of yoga in comparison with a wait-list control in young women (e.g., -1.92 in Kumar et al. [1993] and -1.54 in Woolery et al. [2004], reported in Cramer et al. [2013] meta-analysis). Similarly, our effect size for aerobic

Journal Pre-proof

Journal Pre-proof

exercise (-1.60 in the ITT sample) is comparable to the previous meta-analytic effect size for exercise versus wait-list (Kvam et al., 2016; -1.24). Further, 61% and 60% of those who started treatment (ITT sample) in the current Bikram yoga and exercise groups, respectively, met strict clinical criteria for remission at treatment end. These remission rates are comparable to those reported in previous trials using other forms of yoga in similar depressed samples (e.g., 56.5% remission in Nyer et al., 2019), as well as the rates seen for aerobic exercise (Kvam et al., 2016). It should be noted, however, that depression symptoms *increased* by about two scale points on the Ham-D from pre- to post-treatment in the wait-list condition. Therefore, it is possible that the current study is an over-estimate of the efficacy of Bikram yoga and aerobic exercise, and future studies with more rigorous comparison conditions are required.

Completion rates in the current study were high (79% in the trial overall) relative to a recent uncontrolled trial of Bikram yoga (50% in Nyer et al., 2019), and were similar to those reported in studies that used a higher degree of supervision (Blumenthal et al., 2007; Uebelacker et al., 2017). Importantly, completion rate and class attendance did not differ significantly between the active conditions, and were not significantly associated with outcome. Nevertheless, an important question for future research with larger samples is whether potential differences in treatment outcome between aerobic exercise and Bikram yoga might be *moderated* by number of sessions completed (see Streeter et al., 2017).

Levels of hassles and rumination decreased significantly over the course of the 8-week trial. In particular, patients in the exercise and Bikram yoga groups went from scores that were between 1.5 to over 2.0 SDs above the normative mean to falling within 0.5 SDs of the norm. These results are consistent with those of previous studies showing strong effects of both treatments on these psychological constructs (Askari et al., 2017; Chu et al., 2017; Craft, 2005;

Journal Pre-proof

Journal Pre-proof

Hewett et al., 2018; Kinser et al., 2013, 2014; Michalsen et al., 2005). Novel to the current study, changes in rumination significantly *mediated* the effects of both active treatments relative to waitlist on depression. These results suggest that Bikram yoga and exercise may work, at least in part, by helping depressed individuals interrupt the repetitive elaboration of negative past and/or future cognitive content.

Mindful awareness scores increased significantly in the Bikram yoga group, whereas mindful acceptance scores increased significantly in the exercise group. These results raise the tentative intriguing hypothesis that Bikram yoga and aerobic exercise may interrupt rumination through these differential mindfulness processes – Bikram yoga through raising awareness of the body, and aerobic exercise through fostering acceptance. In the current sample, acceptance was a significant mediator of the effects of exercise on depression, but awareness did not mediate the effects of yoga. This dissociation should be interpreted with caution given low power. Nevertheless, the overall pattern is consistent with previous findings that baseline acceptance, but not awareness, prospectively predicted decreases in depression symptoms and rumination (Long & Hayes, 2014). Future research with larger samples is required to examine the causal paths involving mindfulness and rumination, and their potential interaction, in differentially mediating the antidepressant effects of Bikram yoga versus exercise.

Limitations

The results of this study should be considered in light of the following limitations. First, the sample size was small, and low power can lead to overestimates of true effect sizes when results are statistically significant (Button et al., 2013). The small sample also prevented equivalency tests between yoga and aerobic exercise. Further, the sample consisted of women only, was ethnically homogenous, and included volunteers with average pre-treatment HAM-D

Journal Pre-proof

Journal Pre-proof

scores in the mild-moderate range, thereby limiting generalizability. Second, we did not collect inter-rater reliability data on the diagnostic and Ham-D interviews. Therefore, while the two interviewers received rigorous training and supervision, were blind to condition, and were distributed randomly across conditions, we cannot rule out concerns with interview validity as a confound. Third, women in the wait-list condition experienced a worsening of their symptoms from pre- to post-treatment and, thus, the current results should be interpreted only with reference to other studies that also used a wait-list control and future studies should include an active comparison condition to avoid iatrogenic effects.

Fourth, for ethical reasons we allowed women to maintain their current level of treatment. Therefore, future studies are required to determine whether Bikram yoga should be recommended as a monotherapy or as adjunctive to conventional treatments for mild-moderate depression. Fifth, the aerobic exercise included a wide variety of activities and was not matched to the yoga condition for metabolic equivalents. Relatedly, while Bikram yoga does have a standardized protocol, and instructors were required to meet the stringent criteria for certification, we did not do fidelity ratings. Sixth, the mediating variables were assessed by self-report, thus studies using behavioral and/or physiological measures of stress are needed to validate the current results. Finally, an important limitation of Bikram yoga and aerobic exercise is that they are physically challenging and, thus, the current results may be limited by selection biases related to athletic ability and/or motivation. Therefore, an important future question is whether individual differences in personality and/or motivation contribute to differences in adherence to aerobic exercise versus Bikram yoga (e.g., Bagby et al., 2008). Both aerobic exercise and Bikram yoga, specifically, are well-tolerated even in samples of older adults with health challenges (Hewett et al., 2015). Nevertheless, generalization to other forms of yoga that do not involve strenuous heat conditions (e.g., Asthanga, Hatha) is an important next step.

Journal Pre-proof

Journal Pre-proof

The current preliminary results suggest that, relative to a waitlist condition, Bikram yoga is efficacious in the treatment of mild-to-moderate depression, and is associated with clinically significant symptom change. Further, descriptively, rates of response and change in symptoms and stress-related constructs are similar to those seen in aerobic exercise, an established first-line treatment for mild to moderate depression. Both Bikram yoga and aerobic exercise may work, at least in part, by helping individuals interrupt the ruminative thinking processes that maintain depression. Yoga and exercise are readily accessible in most communities. While the current preliminary results await replication, they add to a growing body of research suggesting that increased use of these complementary approaches may help reduce delays for effective treatment, thereby reducing the burden of this devastating disorder.

Author Statement

Contributors

The authors in order are:

Cherie L. La Rocque, Ph.D., Queen's University

Raegan Mazurka, M.Sc., Queen's University

Troy J. R. Stuckless, M.Sc., University of Toronto

Kyra Pyke, Ph.D., Queen's University

Kate L. Harkness, Ph.D., C. Psych., Queen's University

C. La Rocque, K. Pyke, and K. Harkness developed the research questions and design of the current report. C. La Rocque, R. Mazurka, and T. Stuckless collected the data. C. La Rocque conducted the statistical analyses and wrote the first draft of the manuscript under the supervision of K. Harkness. All authors provides edits and approved the final manuscript.

Role of the Funding Source

Journal Pre-proof

Journal Pre-proof

This study was funded by a Canadian Institutes of Health Research (CIHR) Doctoral Canada Graduate Scholarship awarded to C. La Rocque, and a Queen's University Senate Advisory Research Council grant awarded to K. Harkness and K. Pyke. The funding source provided funds for all research costs. The funders had no role in the design and conduct of the study; collection, management, analysis, and interpretation of the data; preparation, review, or approval of the manuscript; and decision to submit the manuscript for publication.

Declaration of Competing Interest

Dr. La Rocque, Raegan Mazurka, Troy Stuckless, Dr. Pyke, and Dr. Harkness have no conflicts of interest to report.

Acknowledgements

The authors gratefully acknowledge the Kingston YMCA and Feel Yoga studio for opening their facilities to our participants. We also thank Dustin Washburn, Amanda Shamblaw, Mateya Dimnik, Jennifer Gillies, Chloe Hudson, Stephanie Clayton, Hailey Ventola, Alexa Scarcello, Trevor King, Meghan Plotnick, Katrina D'Urzo, Jennifer Williams, Sarah Schmitter, and Shannen Murray for help with data collection, coding, and management, and Jasmine Chananna for help with manuscript preparation. Finally, we are grateful to Dr. Zindel Segal, Dr. Ian Janssen, Dr. Tom Hollenstein, and Dr. Chris Bowie for their insightful comments on previous drafts of this paper.

Journal Pre-proof

References

ACORN Organization Inc. RCI and clinical cutoff calculator. http://www.psychoutcomes.org

Aldao, A., Nolen-Hoeksema, S., Schweizer, S. 2010. Emotion-regulation strategies across psychopathology: a meta-analytic review. Clin Psychol Rev 30, 217-237

American College of Sports Medicine. 2013. ACSM's Health-Related Physical Fitness Assessment Manual (4th ed.). Philadelphia, PA: Lippincott Williams & Wilkins.

American Psychiatric Association. 2013. Diagnostic and Statistical Manual of Mental Disorders (5th ed.). Washington, DC: Author.

Journal Pre-proof

Askari, J., Saberi-Kakhki, A., Taheri, H., Yassini, S. M. 2017. The effect of aerobic exercise on different symptoms of depression: an investigation of psychological mechanism of stress and coping. Open J Med Psychol. 6, 86-102.

Bagby, R. M., Quilty, L. C., Segal, Z. V., McBride, C. C., Kennedy, S. H., Costa, P. T. 2008. Personality and Differential Treatment Response in Major Depression: A Randomized Controlled Trial Comparing Cognitive-Behavioural Therapy and Pharmacotherapy. Can J Psychiat. 53, 361-370.

Bleakley, C. M., Costello, J. T. 2013. Do thermal agents affect range of movement and mechanical properties in soft tissues? A systematic review. Arch Phys Med Rehab. 94, 149-163.

Blumenthal, J. A., Babyak, M. A., Doraiswamy, M., Watkins, L., Hoffman, B. M., Barbour, K. A., …Sherwood, A. 2007. Exercise and pharmacotherapy in the treatment of major depressive disorder. Psychosom Med. 69, 587-596.

Button, K. S., Ioannidis, J. P. A., Mokrysz, C., Nosek, B. A., Flint, J., Robinson, E. S. J., Munafò, M. R. 2013. Power failure: why small sample size undermines the reliability of neuroscience. Nature Rev Neurosci. 14, 365-376.

Cardaciotto, L., Herbert, J. D., Forman, E. M., Moitra, E., Victoria, F. 2008. The assessment of present-moment awareness and acceptance. Assess. 15, 204-223.

Choudhury, B. 2007. Bikram Yoga. New York: Harper Collins.

Chu, I.-H., Wu, W.-L., Lin, I.-M., Chang, Y., Lin, Y., Yang, P.-C. 2017. Effects of Yoga on Heart Rate Variability and Depressive Symptoms in Women. J Altern Complement Med. 23, 310–316. https://doi.org/10.1089/acm.2016.0135

Craft, L. L. 2005. Exercise and clinical depression: Examining two psychological mechanisms. Psychol

Journal Pre-proof

Sport Exerc. 6, 151-171.

Cramer, H., Anheyer, D., Lauche, R., Dobos, G. 2017. A systematic review of yoga for major depressive disorder. J Aff Dis. 213, 70-77.

Cramer, H., Lauche, R., Langhorst, J., Dobos, G. 2013. Yoga for depression: a systematic review and meta-analysis. Depress Anxiety. 30, 1068-1083.

de Bruin, E. I., van der Zwan, J. E., Bogels, S. M. 2016. A RCT comparing daily mindfulness meditations, biofeedback exercises, and daily physical exercise on attention control, executive functioning, mindful awareness, self-compassion, and worrying in stressed young adults. Mindfulness. 7, 1182-1192.

de Jonge, P., Wardenaar, K. J., Hoenders, H. R., Evans-Lacko, S., Kovess-Masfety, V., Aguilar-Gaxiola, S.,…Thornicroft, G. (2018). Complementary and alternative medicine contacts by persons with mental disorders in 25 countries: results from the World Mental Health Surveys. Epid Psychiatr Sci. *27, 552-567.*

DeLongis, A., Folkman, S., Lazarus, R. S. 1988. The impact of daily stress on health and mood: psychiatric and social resources as mediators. J Pers Soc Psychol. 54, 486-495.

de Manincor, M., Bensoussan, A., Smith, C. A., Fahey, P., Bourchier, S. 2015. Establishing key components of yoga interventions for reducing depression and anxiety, and improving well-being: a Delphi method study. BMC Compl Alt Med. 15, 1-5.

Erdfelder, E., Faul, F., Buchner, A. 1996. GPOWER: A general power analysis program. Behav Res Methods Instrum Comput. 28, 1–11.

Falsafi, N. 2016. A randomized controlled trial of mindfulness versus yoga: effects on depression and/or anxiety in college students. J Am Psychiatr Nurses Assoc. *22*, 483-497.

Journal Pre-proof

Journal Pre-proof

First, M. B., Spitzer, R. L, Gibbon, M., & Williams, J. B. W. 2002. Structured Clinical Interview for DSM-IV-TR Axis I Disorders, Research Version, Patient Edition. (SCID-I/P). New York: Biometrics Research.

Grove, W., Andreasen, N., McDonald-Scott, P., Keller, M. Shapiro, R. 1981. Reliability studies of psychiatric diagnosis. Arch General Psychiat. 38, 408-413.

Hamilton, M. 1960. A rating scale for depression. J Neurol Neurosur Ps. 25, 56–62.

Hayes, A. F. 2012. PROCESS: A versatile computational tool for observed variable mediation, moderation, and conditional process modeling. Retrieved from http://www.afhayes.com/ public/process2012.pdf

Hayes, A. F., & Rockwood, N. J. 2017. Regression-based statistical mediation and moderation analysis in clinical research: observations, recommendations, and implementation. Beh Res Ther. 98, 39-57.

Hewett, Z. L., Ransdell, L. B., Gao, Y., Petlichkoff, L. M., Lucas, S. 2011. An examination of the effectiveness of an 8-week Bikram yoga program on mindfulness, perceived stress, and physical fitness. J Exer Sci Fitness. *9*, 87-92.

Hewett, Z. L., Cheema, B. S., Pumpa, K. L., Smith, C. A. 2015. The effects of Bikram yoga on health: critical review and clinical trial recommendations. Evid-Based Compl Alt. 428427.

Hewett, Z. L., Pumpa, K. L., Smith, C. A., Fahey, P. P., Cheema, B. S. 2018. Effect of a 16-week Bikram yoga program on perceived stress, self-efficacy and health-related quality of life in stressed and sedentary adults: a randomised controlled trial. J Sci Med Sport. 21, 352-357.

Kabat-Zinn, J. 2003. Mindfulness-based interventions in context: past, present, and future. Clin Psychol-Sci Prac. 10, 144-156.

Kangasniemi, A., Lappalainen, R., Kankaanpää, A., Tammelin, T. 2014. Mindfulness skills, psychological flexibility, and psychological symptoms among physically less active and active adults. Mental Health and Physical Activity. 7, 121-127.

Journal Pre-proof

Kinser, P. A., Bourguignon, C., Whaley, D., Hauenstein, E., Taylor, A. G. 2013. Feasibility, acceptability, and effects of gentle Hatha yoga for women with major depression: findings from a randomized controlled mixed-methods study. Arch Psychiat Nurs. 27, 137-147.

Kinser, P. A., Elswick, R. K., Kornstein, S. 2014. Potential long-term effects of a mind-body intervention for women with major depressive disorder: sustained mental health improvements with a pilot yoga intervention. Arch Psychiat Nurs. 28, 377-383.

Kvam, S., Kleppe, C. L., Nordhus, I. H., Hovland, A. 2016. Exercise as a treatment for depression: a meta-analysis. J Aff Dis. 202, 67-86.

Kwok, J. Y. Y., Kwan, J. C. Y., Auyeung, M., Mok, V. C. T., Lau, C. K. Y., Choi, K. C., Chan, H. Y. L. 2019. Effects of Mindfulness Yoga vs Stretching and Resistance Training Exercises on Anxiety and Depression for People With Parkinson Disease: A Randomized Clinical Trial. JAMA Neurology. 76, 755-753. doi:10.1001/jamaneurol.2019.0534

Lakens, D., Scheel, A. M., Isager, P. M. 2018. Equivalence Testing for Psychological Research: A Tutorial. Advances in Methods and Practices in Psychological Science. 1, 259-269.

Long, D. M., Hayes, S. C. 2014. Acceptance, mindfulness, and cognitive reappraisal as longitudinal predictors of depression and quality of life in educators. J Contextual Beh Sci. 3, 38-44.

Michalsen, A., Grossman, P., Acil, A., Langhorst, J., Ludtke, R., Esch, T., …Dobos, G. J. 2005. Rapid stress reduction and anxiolysis among distressed women as a consequence of a three-month intensive yoga program. Med Sci Monitor. 11, 555-561.

Mothes, H., Klaperski, S., Seelig, H., Schmidt, S., Fuchs, R. 2014. Regular aerobic exercise increases dispositional mindfulness in men: a randomized controlled trial. Mental Health and Physical Activity. *7*, 111-119.

Journal Pre-proof

National Institute for Clinical Excellence. 2018. Depression in Adults: Recognition and Management. Clinical Guideline 90. http://www.nice. org.uk/guidance/cg90.

Nolen-Hoeksema, S. Morrow, J. 1991. A prospective study of depression and posttraumatic stress symptoms after a natural disaster: the 1989 Loma Prieta earthquake. J Pers Soc Psychol. 61, 115–121.

Nolen-Hoeksema, S., Larson, J., & Grayson, C. (1999). Explaining the gender difference in depressive symptoms. J Pers Soc Psychol. 77, 1061–1072.

Nyer, M., Hopkins, L. B., Farabaugh, A., Nauphal, M., Parkin, S., McKee, M. M., Miller, K. K., Streeter, C., Uebelacker, L. A., Fava, M., Alpert, J. E., Pedrelli, P., Mischoulon, D. J. (2019). Community-Delivered Heated Hatha Yoga as a Treatment for Depressive Symptoms: An Uncontrolled Pilot Study. Altern Complement Med. 25, 814-823. doi: 10.1089/acm.2018.0365.

Park, C. L., Elwy, A. R., Maiya, M., Sarkin, A. J., Riley, K. E., Eisen, S. v.,…Groessl, E. J. 2018. The Essential Properties of Yoga Questionnaire (EPYQ): Psychometric Properties. Int J Yoga Therap. 28, 23-38. doi: 10.17761/2018-00016R2.

Patten, S. B., Williams, J. V. A., Lavorato, D. H., Wang, J. L., McDonald, K., Bulloch, A. G. M. 2015. Descriptive epidemiology of major depressive disorder in Canada in 2012. Can J Psychiat. 60, 23-30.

Perraton, L. G., Kumar, S., Machotka, Z. 2010. Exercise parameters in the treatment of clinical depression: a systematic review of randomized controlled trials. J Eval Clin Prac. 16, 597-604.

Raudenbush, S. W., Bryk, A. S., Congdon, R. 2017. HLM 7.03 for Windows. Skokie, IL: Scientific Software International, Inc.

Ravindran, A. V., Balneaves, L. G., Faulkner, G., Ortiz, A., McIntosh, D., Morehouse, R. L., …Parikh, S. V. 2016. Canadian Network for Mood and Anxiety Treatments (CANMAT) 2016 clinical guidelines for

Journal Pre-proof

Journal Pre-proof

the management of adults with major depressive disorder: 5. Complementary and alternative medicine treatments. Can J Psychiat. 61, 576-587.

Riley, K. E., Park, C. L. 2015. How does yoga reduce stress? A systematic review of mechanisms of change and guide to future inquiry. Health Psychol Rev. 9, 379-396.

Rush, A. J., Trivedi, M. H., Wisniewski, S. R., Nierenberg, A. A., Stewart, J. W., Warden, D.,…Fava, M. 2006. Acute and longer-term outcomes in depressed outpatients requiring one or several treatment steps: a STAR*D report. Am J Psychiat. 163, 1905-1917.

Rush, A. J., Thase, M. E. 2018. Improving Depression Outcome by Patient-Centered Medical Management. Am J Psychiat. 175, 1187-1198.

Salk, R. H., Hyde, J. S., Abramson, L. Y. 2017. Gender differences in depression in representative national samples: Meta-analyses of diagnoses and symptoms. Psychol Bull. 143, 783-822.

Salmon, P., Hanneman, S., Harwood, B. 2010. Associative/dissociative cognitive strategies in sustained physical activity: literature review and proposal for a mindfulness-based conceptual model. Sport Psychol. 24, 127-156.

Salmon, P., Lush, E., Jablonski, M., Sephton, S. E. 2009. Yoga and mindfulness: clinical aspects of an ancient mind/body practice. Cog Beh Prac. 16, 59-72.

Schuch, F. B., Vancampfort, D., Richards, J., Rosenbaum, S., Ward, P. B., Stubbs, B. 2016. Exercise as a treatment for depression: a meta-analysis adjusting for publication bias. J Psychiat Res. 77, 42-51.

Streeter, C. C., Gerbarg, P. L., Whitfield, T. H., Owen, L., Johnston, J., Silveri, M. M., …Jensen, J. E. 2017. Treatment of Major Depressive Disorder with Iyengar Yoga and Coherent Breathing: A Randomized Controlled Dosing Study. J Altern Complement Med. 23, 201–207.

Thomas, S., Reading, I., Shephard, R. J. 1992. Revision of the Physical Activity Readiness Questionnaire

Journal Pre-proof

(PAR-Q). Can Journal Sport Sci. 17, 338-345.

Trajković, G., Starčević, V., Latas, M., Leštarević, M., Ille, T., Bukumirić, Z., Marinkovic, J. 2011. Reliability of the Hamilton Rating Scale for Depression: a meta-analysis over a period of 49 years. Psychiat Res. 189, 1-9. doi: 10.1016/j.psychres.2010.12.007.

Uebelacker et al., 2010

Uebelacker, L. A., Epstein-Lubow, G., Gaudiano, B. A., Tremont, G., Battle, C. L., Miller, I. W. 2010. Hatha yoga for depression: critical review of the evidence for efficacy, plausible mechanisms of action, and future research. J Psychiatr Pract. 16, 22-33.

Uebelacker, L. A., Tremont, G., Gillette, L. T., Epstein-Lubow, G., Strong, D. R., Abrantes, A. M., …Miller, I. W. 2017. Adjunctive yoga v. health education for persistent major depression: a randomized controlled trial. Psychol Med. 47, 2130-2142.

Uhrlass, D. J., Gibb, B. E. 2008. Childhood emotional maltreatment and the stress generation model of depression. J Soc Clin Psychol. 26, 119-130.

Ulmer, C. S., Stetson, B. A., & Salmon, P. G. (2010). Mindfulness and acceptance are associated with exercise maintenance in YMCA exercisers. *Behaviour Research and Therapy, 48*, 805-809.

World Health Organization. 2017. Depression and Other Common Mental Disorders: Global Health Estimates. Geneva: World Health Organization.

Journal Pre-proof

Table 1

Demographic and Clinical Characteristics of Full Sample Stratified by Group (N=53)

	Yoga<br>(*n* = 18)	Exercise<br>(*n* = 20)	Waitlist<br>(*n* = 15)	F/χ^2

Journal Pre-proof

Age (*M*/*SD*)	34.17/15.75	34.85/15.15	29.40/13.08	0.65
Ethnicity (*n*/%)				0.47
White	13/72.2	14/70.0	12/80.0	
Other	5/27.8	6/30.0	3/20.0	
Highest education level (*n*/%)				0.35
Grade 12/Some university	10/55.6	10/50.0	9/60.0	
University degree	8/44.4	10/50.0	6/40.0	
Relationship status (*n*/%)				0.77
Unmarried	11/61.1	12/60.0	11/73.3	
Married	7/38.9	8/40.0	4/26.7	
Age of onset (*M*/*SD*)	19.78/5.00	19.35/7.09	18.80/6.27	0.10
Total # of episodes (*M*/*SD*)	4.11/3.39	2.80/1.32	3.53/2.88	1.19
Current treatment: Yes (*n*/%)	8/44.4	9/45.0	7/46.7	0.02
Medication	4/22.2	4/20.0	7/46.7	
Psychotherapy	3/16.7	4/20.0	0/0	
Both	1/5.6	1/5.0	0/0	
Comorbid disorder: Yes (*n*/%)	8/44.4	10/50.0	5/33.3	0.98
Panic Disorder**	0/0	2/10.0	1/6.7	
Social Phobia	3/16.7	4/20.0	4/26.7	
Specific Phobia	2/11.1	3/15.0	3/20.0	
Generalized Anxiety Disorder	2/11.1	3/15.0	1/6.7	
Anxiety Disorder NOS	1/5.6	1/5.0	0/0	
Obsessive Compulsive Disorder	0/0	1/5.0	0	
Pre-T_X HAM-D (*M*/*SD*)	13.39/3.07	12.00/3.77	13.93/3.71	1.44
Post-T_X HAM-D (*M*/*SD*)	8.17/6.15	6.75/6.05	16.33/5.84	12.02*

Note: NOS = Not Otherwise Specified; Pre-Tx = Pre-Treatment; Post-Tx = Post-Treatment; HAM-D = Hamilton Rating Scale for Depression; * Last observation carried forward ($p < .001$); ** Frequencies do not add up to total as some individuals have more than one comorbid disorder.

Table 2

Pre- and Post-Treatment Depression Severity and Psychological Variables Stratified by Treatment Group in the Completer Sample

Journal Pre-proof

	Yoga	Exercise	Waitlist	*F*
Pre-T_X HAM-D (*M*/*SD*)	12.93/3.08	11.80/4.12	14.00/4.13	1.13
Post-T_X HAM-D (*M*/*SD*)	6.67/5.55	4.80/5.60	16.17/6.31	14.31**
Pre-T_X hassles (*M*/*SD*)	61.47/30.03	52.43/25.31	63.25/24.80	0.63
Post-T_X hassles (*M*/*SD*)	26.00/17.89	24.50/4.51	30.58/15.72	0.45
Pre-T_X rumination (*M*/*SD*)	61.60/12.10	53.93/15.10	61.42/9.01	1.76
Post-T_X rumination (*M*/*SD*)	44.07/13.51	36.67/8.66	55.92/13.55	9.60**
Pre-T_X awareness (*M*/*SD*)	36.13/4.70	35.80/6.37	36.17/7.09	0.02
Post-T_X awareness (*M*/*SD*)	37.53/8.11	35.47/8.09	33.58/5.18	0.96
Pre-T_X acceptance (*M*/*SD*)	22.80/5.10	25.47/6.59	21.25/5.40	1.88
Post-T_X acceptance (*M*/*SD*)	28.20/7.74	31.33/6.76	23.75/4.81	4.32*

Note. * $p < .05$; ** $p < .001$; Pre-Tx = Pre-Treatment; Post-Tx = Post-Treatment; HAM-D = Hamilton Rating Scale for Depression.

Table 3

Parameter Estimates for the Multilevel Model of Hassles, Rumination, Awareness, and Acceptance.

	Coefficient	*SE*	*t*
**Hassles**: Post-T_X intercept			
Intercept	33.84	4.55	7.44***
Yoga vs. waitlist	-10.85	5.94	-1.83+
Exercise vs. waitlist	-16.24	6.23	-2.61*
Pre- to Post-T_X slope			
Intercept	-2.27	0.65	-3.49***
Yoga vs. waitlist	-0.45	0.84	-0.54
Exercise vs. waitlist	-0.45	0.90	-0.50
**Rumination**: Post-T_X intercept			
Intercept	57.86	3.69	15.68***
Yoga vs. waitlist	-15.84	4.75	-3.33**
Exercise vs. waitlist	-21.61	5.15	-4.20***

Journal Pre-proof

Pre- to Post-T_X slope			
Intercept	-0.39	1.13	-0.35
Yoga vs. waitlist	-3.48	1.45	-2.40*
Exercise vs. waitlist	-4.05	1.57	-2.58*
**Awareness**: Post-T_X intercept			
Intercept	32.17	2.10	15.29***
Yoga vs. waitlist	5.73	2.81	2.04*
Exercise vs. waitlist	2.48	2.85	0.87
Pre- to Post-T_X slope			
Intercept	-0.35	0.25	-1.41
Yoga vs. waitlist	0.70	0.33	2.10*
Exercise vs. waitlist	0.32	0.34	0.95
**Acceptance**: Post-T_X intercept			
Intercept	23.77	2.05	11.60***
Yoga vs. waitlist	2.82	2.70	1.05
Exercise vs. waitlist	7.44	2.84	2.62*
Pre- to Post-T_X slope			
Intercept	0.16	0.29	0.55
Yoga vs. waitlist	0.09	0.39	0.22
Exercise vs. waitlist	0.69	0.41	1.69

Note. + $p < .10$; * $p < .05$; ** $p < .01$; *** $p < .001$; Pre-Tx = Pre-treatment; Post-Tx = Post-treatment. The models for Hassles, Rumination, and Acceptance controlled for age.

Table 4

Regression Coefficients, Standard Errors, and Model Summary Information for Mediating Effects of Rumination and Mindful Acceptance

	Post-T_X rumination (*M*)			Post-T_X HAM-D (*Y*)			Indirect effects		
	Coeff	*SE*	*p*	Coeff	*SE*	*p*	Coeff	*SE*	[illegible]
Constant	47.18	11.71	.003	-7.57	4.98	.138			
Yoga vs. waitlist (D_1)	-11.77	4.52	.013	-5.87	1.74	.002	-3.02	1.33	[illegible]
Exercise vs. waitlist (D_2)	-17.28	4.91	.000	-5.85	2.01	.006	-4.43	1.34	[illegible]
Pre-T_X rumination (C_1)	0.12	0.15	.425	0.03	0.05	.625			
Pre-T_X HAM-D (C_2)	0.10	0.54	.858	0.56	0.19	.006			
Post-T_X rumination (*M*)				0.26	0.06	.000			
		$R^2 = .33$			$R^2 = .73$				

Journal Pre-proof

Journal Pre-proof

	$F(4, 36) = 4.36$, $p = .006$			$F(5,35) = 18.85$, $p < .001$					
	Post-T_X acceptance (*M*)			Post-T_X HAM-D (*Y*)			Indirect effects		
	Coeff	*SE*	*p*	Coeff	*SE*	*p*	Coeff	*SE*	[illegible]
Constant	8.11	4.88	.106	10.19	4.46	.029			
Yoga vs. waitlist (D_1)	3.95	2.06	.064	-7.17	1.90	.001	-1.67	1.08	[illegible]
Exercise vs. waitlist (D_2)	6.32	2.37	.012	-7.53	2.29	.002	-2.67	1.20	[illegible]
Pre-T_X acceptance (C_1)	0.53	0.15	.001	0.24	0.16	.133			
Pre-T_X HAM-D (C_2)	0.31	0.25	.223	0.78	0.22	.001			
Post-T_X acceptance (*M*)				-0.42	0.15	.008			
	$R^2 = .45$			$R^2 = .66$					
	$F(4, 35) = 7.19$, $p < .001$			$F(5, 34) = 13.01$, $p < .001$					

Note. *M* = mediator; *Y* = dependent variable; D_1 and D_2 = dummy variables (waitlist coded as reference group); *C* = covariates; Coeff = unstandardized regression coefficient; Pre-T_X = Pre-treatment; Post-T_X = Post-treatment; HAM-D = Hamilton Rating Scale for Depression.

Journal Pre-proof
