# Supplementary figures and images for: Does physical activity-based intervention decrease repetitive negative thinking? A systematic review
Source: PLoS One. 2025 Apr 1;20(4):e0319806. doi: 10.1371/journal.pone.0319806 (PMC11960971; doi:10.1371/journal.pone.0319806)

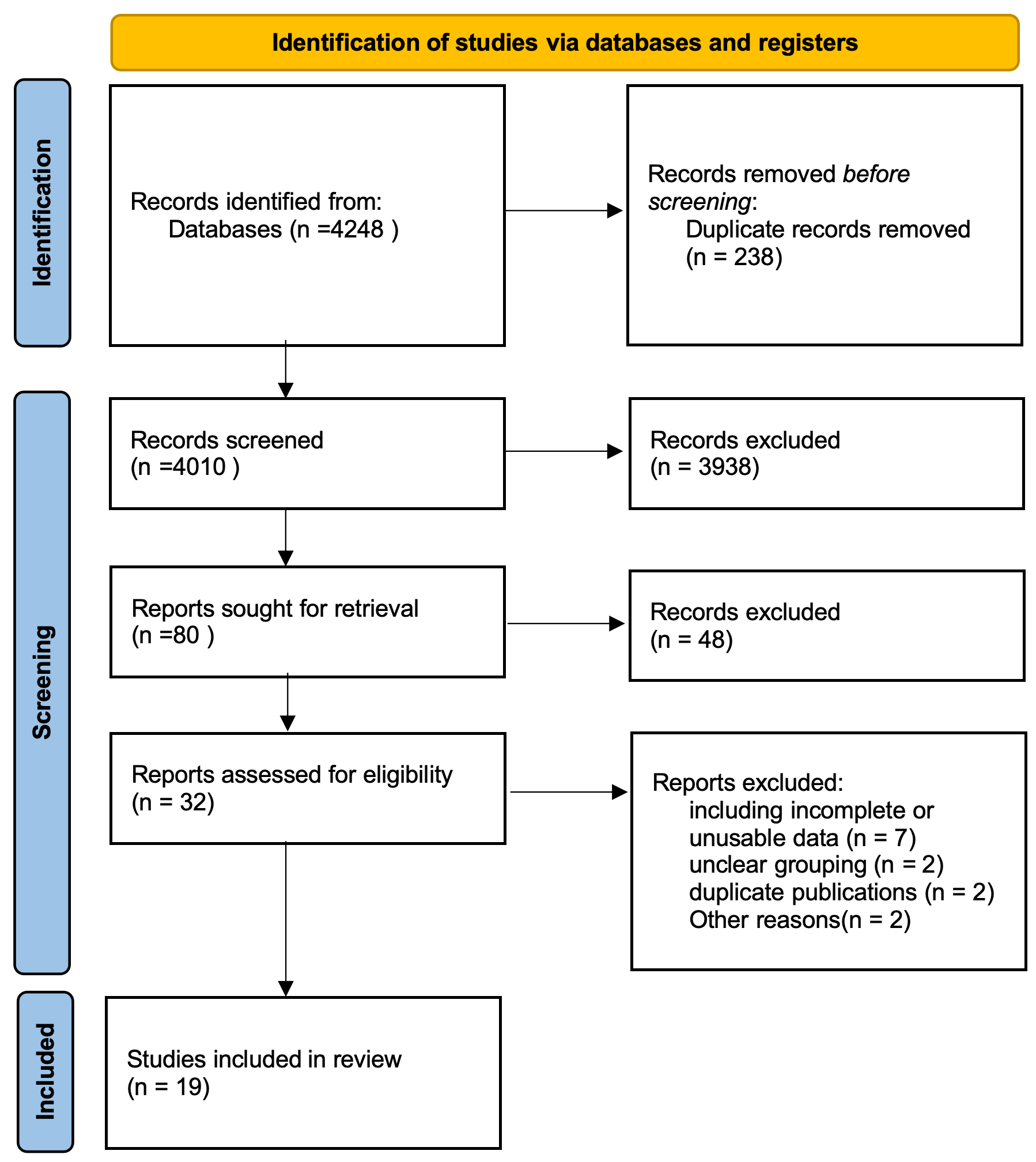

Supplement: S1 Fig — (TIF) [file pone.0319806.s002.tif]

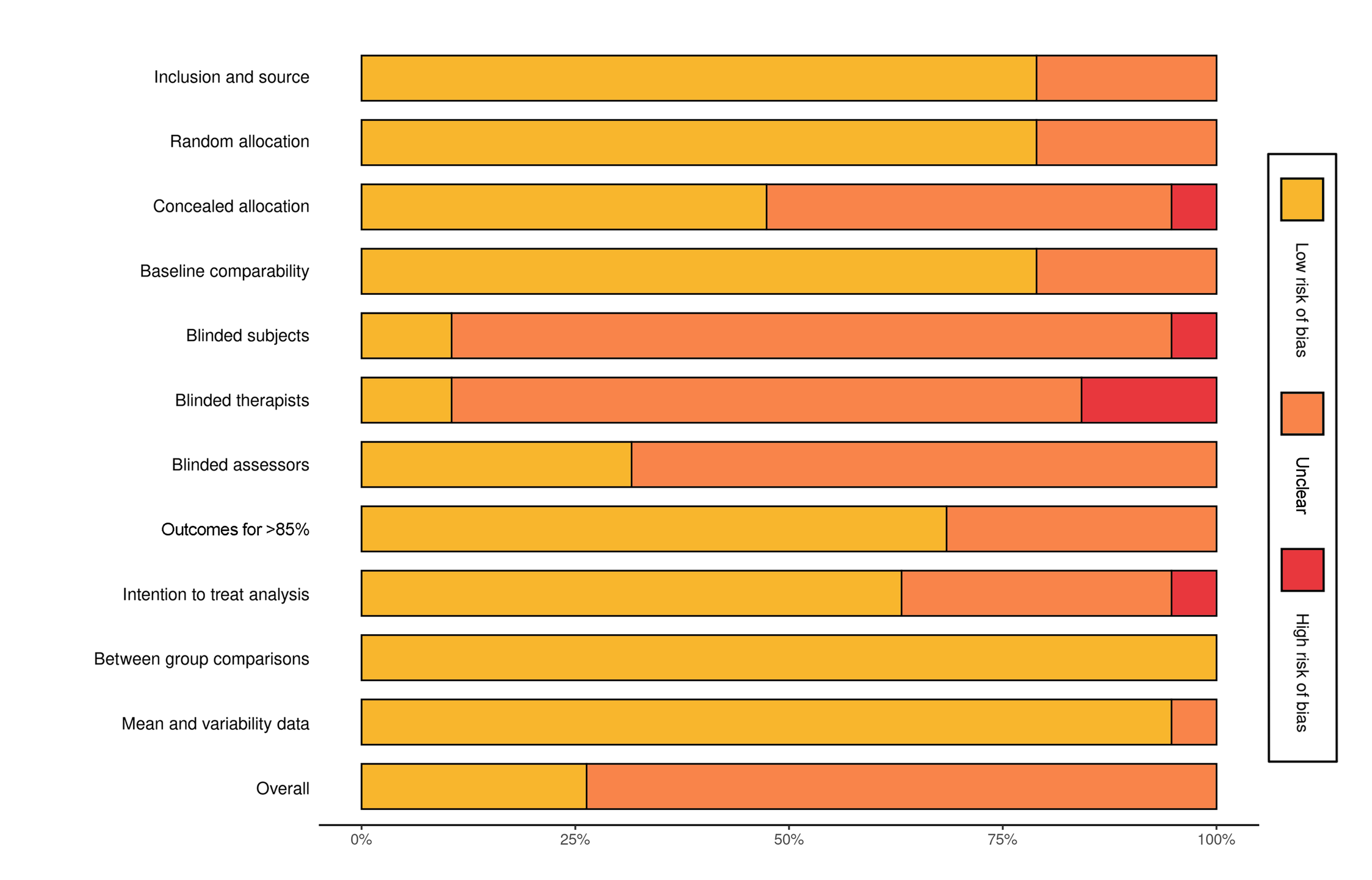

Supplement: S2 Fig — (TIF) [file pone.0319806.s003.tif]

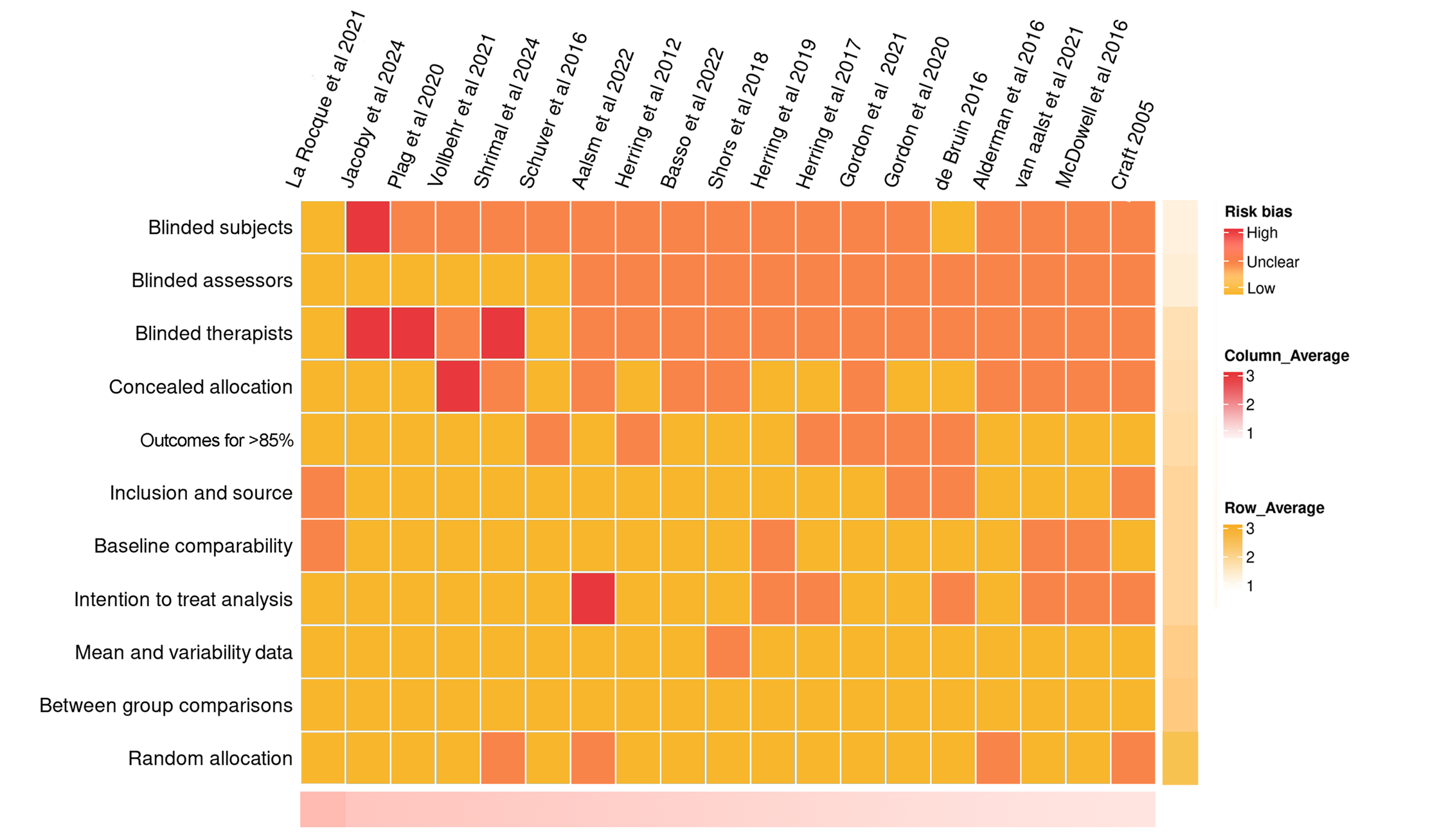

Supplement: S3 Fig — (TIF) [file pone.0319806.s004.tif]
